# Cyclopropane-Fused *N*-Heterocycles via Aza-Heck-Triggered C(sp^3^)–H Functionalization Cascades

**DOI:** 10.1021/jacs.2c08304

**Published:** 2022-09-09

**Authors:** Changcheng Jing, Benjamin T. Jones, Ross J. Adams, John F. Bower

**Affiliations:** † Department of Chemistry, University of Liverpool, Crown Street, Liverpool L69 7ZD, United Kingdom; ‡ School of Chemistry, University of Bristol, Bristol BS8 1TS, United Kingdom

## Abstract

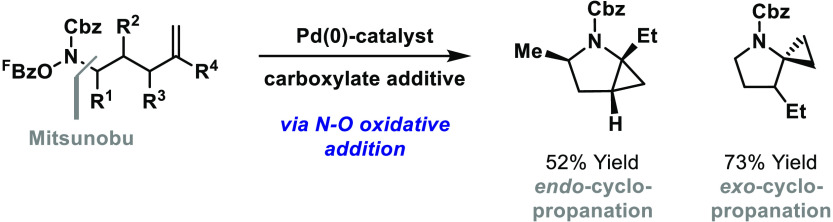

Unique examples of aza-Heck-based C(sp^3^)–H functionalization cascades are described. Under Pd(0)-catalyzed conditions, the aza-Heck-type cyclization of *N*-(pentafluorobenzoyloxy)carbamates generates alkyl–Pd(II) intermediates that effect C(sp^3^)–H palladation en route to cyclopropanes. Key factors that control the site selectivity of the cyclopropanation process have been elucidated such that selective access to a wide range of ring- or spiro-fused systems can be achieved.

Cyclopropanes are routinely employed in pharmaceutical design to moderate compound lipophilicity or N-centered basicity.^1^ Reflecting their relative ease of synthesis, *peripheral* cyclopropane units are featured in many marketed drugs, whereas *core* cyclopropane units are encountered less often (Scheme 1A).^2^ An example of the latter is the DPP-4 inhibitor saxagliptin, which possesses a cyclopropane-fused pyrrolidine.^3a^ This subunit is derived from pyroglutamic acid via a lengthy Simmons–Smith-based route, making access to more complex derivatives challenging.^3b^ Accordingly, direct and flexible methods that can address these issues are likely to be of interest. A powerful but underdeveloped option involves the intramolecular aza-palladation of an alkene (step a) in advance of C–H palladation-initiated cyclopropanation (step b) (Scheme 1B). Yang and co-workers have demonstrated such processes under oxidative conditions (Scheme 1C).^4^ Although conceptually important, specific constraints hamper both steps; for example, step a requires a conformationally biasing and acidifying anilide unit and is not well suited to six-ring cyclizations, whereas step b suffers from limited scope and selectivity.

**Scheme 1:**
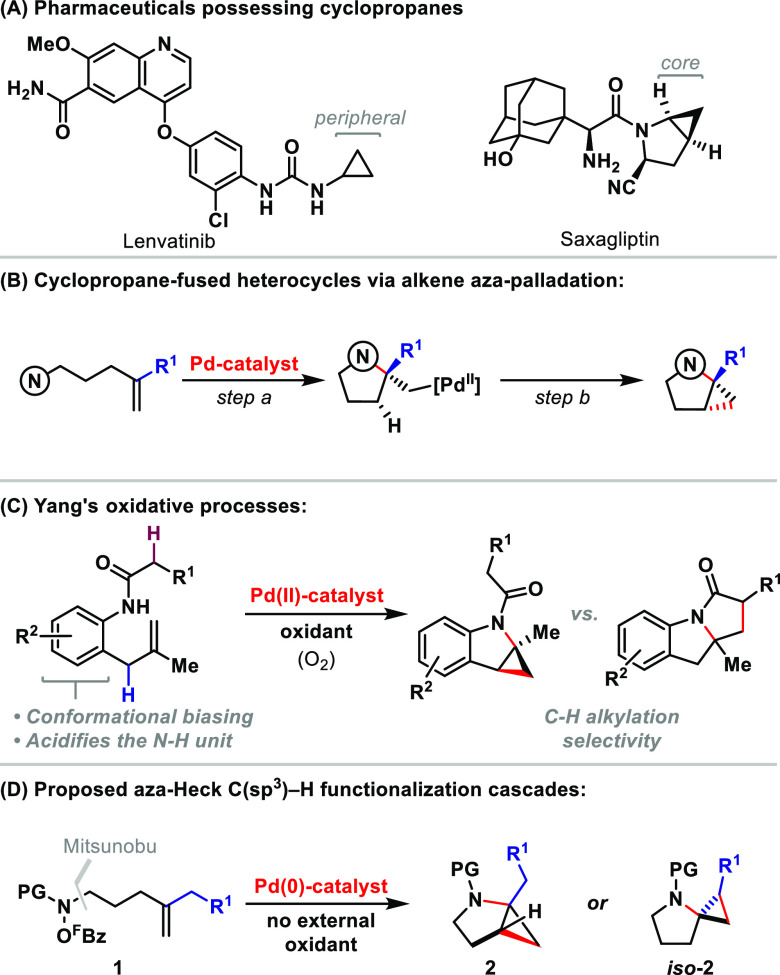
Introduction

We and others have demonstrated that the efficiency of alkene aza-palladation-based processes can be enhanced substantially by replacing an external oxidant (e.g., O_2_ in Scheme 1C) with a N–O bond.^5^ In this approach, N–O oxidative addition unites the key processes of substrate binding and catalyst oxidation, so the catalysis is often more robust. The non-oxidative conditions also mean that highly tunable P-based ligands can be used to moderate the properties of the Pd center. Indeed, aza-Heck-based approaches of this type now allow an expanding range of nonconformationally biased cyclizations and cascades involving sterically and electronically diverse alkenes.^5a−5f,5i,5j^ This includes asymmetric aza-Heck cyclizations^5a,5d^ and cascade reactions involving aryl C(sp^2^)–H palladation.^5a,5j^ In the present study, we outline aza-Heck-triggered cyclopropanation processes that involve a more challenging C(sp^3^)–H palladation step (Scheme 1D). To the best of our knowledge, these are the first examples of C(sp^3^)–H functionalization cascades that use newer classes of N–O units (i.e., nonoxime ester-based).^5l,5m,6^ Compared to Scheme 1C, notable features of these new processes include (a) the efficient participation of sterically encumbered alkenes, (b) efficient 5-*exo* cyclizations in the absence of a conformationally biasing and acidifying anilide unit, (c) efficient 6-*exo* cyclizations, and (d) no requirement for the benzylic activation of the target C(sp^3^)–H bond. We also demonstrate that either steric or electronic control can be used to enforce the regioselectivity of the cyclopropanation event, thereby providing selective access to ring- or spiro-fused systems. In broader terms, these studies offer rare examples of C(sp^3^)–H cyclopropanation processes that are triggered by alkene heteropalladation, a sequence that is likely challenging because of the reversibility of the migratory insertion step (vide infra).^4,7,8^

A mechanistic analysis of the processes developed here is outlined in Scheme 2A. The N–O oxidative addition of **1** is expected to provide the aza-Pd(II) intermediate **Int-I**. Prior work has indicated that efficient alkene aza-palladation requires the dissociation of pentafluorobenzoate from **Int-I** to give **Int-I′**.^5b^**Int-I′** undergoes cyclization and carboxylate association to give the alkyl–Pd(II) intermediate **Int-II**, which can provide palladacyclobutane **Int-III** via concerted metalation deprotonation-type metalation. Reductive elimination from **Int-III** then releases the cyclopropane product **2**. Depending on the nature of R (vide infra), alternative palladacyclobutanes may be accessible. The carboxylic acid released during the cyclpropanation sequence is expected to undergo deprotonation by triethylamine, and the resulting triethylammonium salt will triger the facile protodecarboxylation of pentafluorobenzoate to release C_6_F_5_H.^9^ Previous studies indicated that alkene aza-palladation is reversible under cationic conditions (vide infra),^5j,10^ so the success of the process is likely to dependent on the efficiency of the carboxylate-mediated C(sp^3^)–H metalation step (**Int-II** to **Int-III**). Here, external carboxylate additives (R′CO_2_M) are likely required because the pentafluorobenzoate released during N–O oxidative addition is highly dissociative and a relatively weak base. Note that the optimal mechanistic scenario requires a cationic species for aza-palladation (**Int-I′**) and a neutral intermediate for C(sp^3^)–H metalation (**Int-II**). As such, optimal conditions require an appropriate trade-off because the complete partitioning of pathways at each stage is likely unattainable.

**Scheme 2:**
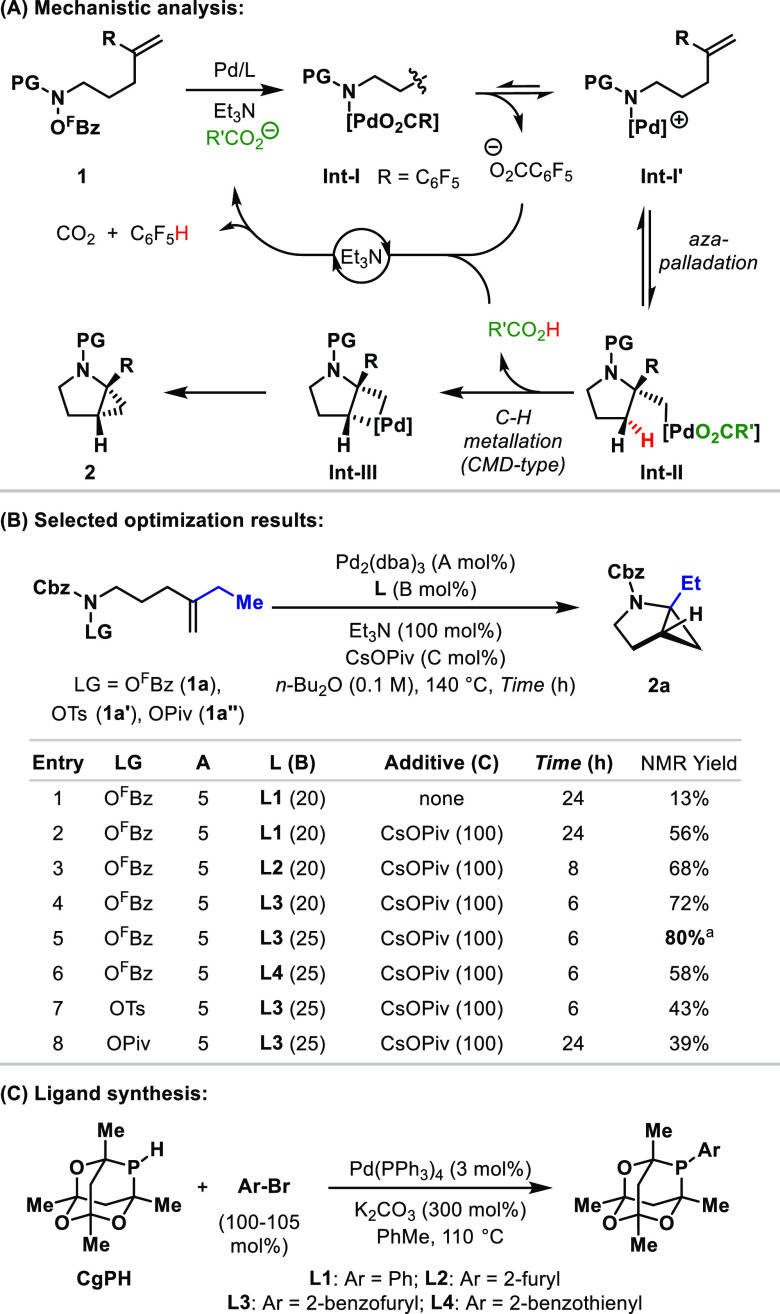
Mechanistic Analysis and Optimization of the Cascade Process Isolated yield. Further optimization results, including the evaluation of other ligands, are given in the SI.

Proof-of-concept studies focused initially on the cyclization of the *O*-pentafluorobenzoyl system **1a** to target **2a** (Scheme 2B). Under the indicated conditions and in the absence of a carboxylate additive, target **2a** was generated in only a 13% yield (entry 1). The addition of 100 mol% CsOPiv markedly increased the efficiency such that **2a** was formed in a 56% yield (entry 2). During these initial studies, other optimal parameters were established. Most significantly, from a screen of P ligands commonly employed in aza-Heck processes, it was found that **L1** (CgPPh) was by far the most efficient.^5b^ Note that P ligands were specifically noted as being incompatible with the method in Scheme 1C.^4^ To optimize the process further, a library of approximately 15 derivatives was prepared from CgPH (Scheme 2C and the SI).^11^ Selected evaluation results are shown in entries 3–6, with the key finding being that the benzofuryl system **L3** could provide **2a** in an 80% yield over 6 h. At this stage we evaluated the choice of the O-based leaving group (entries 7 and 8), which confirmed that the −O^F^Bz system (**1a**) was superior to both −OTs and −OPiv variants **1a′** and **1a″**, respectively. The latter result is consistent with the idea that a cationic aza-palladium intermediate offers an optimal aza-palladation efficiency (**Int-I′** to **Int-II**).

The optimized conditions were proven to be applicable to the synthesis of a wide range of cyclopropane-fused pyrrolidines (Table 1A). For example, a variety of alkyl substituents were tolerated at R^1^, as evidenced by the efficient formation of **2b**–**f**. Note that the bulky secondary alkyl substituents of **2b** and **2f** did not significantly diminish the reaction efficiency relative to, for example, that of **2c**. The electronically distinct styrenyl system **1g** also participated to provide adduct **2g**, albeit with a more modest efficiency. Substituents can be introduced at C4 and C5 of the targets, as demonstrated by the formation of **2h** and **2i**, respectively. For the former, minimal diastereoselectivity was observed, whereas the latter was generated as a single diastereomer. The hydrogenolytic N–Cbz deprotection of **2i** allowed access to the N–DNs derivative **2i′**. **2i′** was characterized by single-crystal X-ray diffraction, which revealed a *syn*-relationship between the methyl and ethyl substituents.^12^ This outcome is consistent with the alkene aza-palladation step being reversible because the productive palladacyclobutane **Int-2i** is formed via the disfavored cyclization mode (**TS-2i**) (Table 1B).^13^ For system **1j**, where R^1^ = Me, the expected product **2j** was formed in a 55% yield alongside smaller quantities of spiro-fused cyclopropane *iso***-2j** (14% yield) (Table 1C). The formation of the latter is presumably facilitated by the C(sp^3^)–H metalation of the more sterically accessible (versus Table 1A) methyl group of **Int-II′**.^14^ To enforce this selectivity, systems possessing a substitution at the internal allylic position (C3) were investigated (Table 1D). In the reaction, the cyclization of the ethyl-substituted system **1k** provided *iso-***2k** in a 73% yield, and the corresponding ring-fused cyclopropane was not observed. This process required an increased reaction temperature (160 °C) and an alternate carboxylate additive (KOAc). These modified conditions were also effective for the selective formation of *iso-***2l** and the intriguing vicinally dispirofused adduct *iso-***2m**.^15^

**Table 1:**
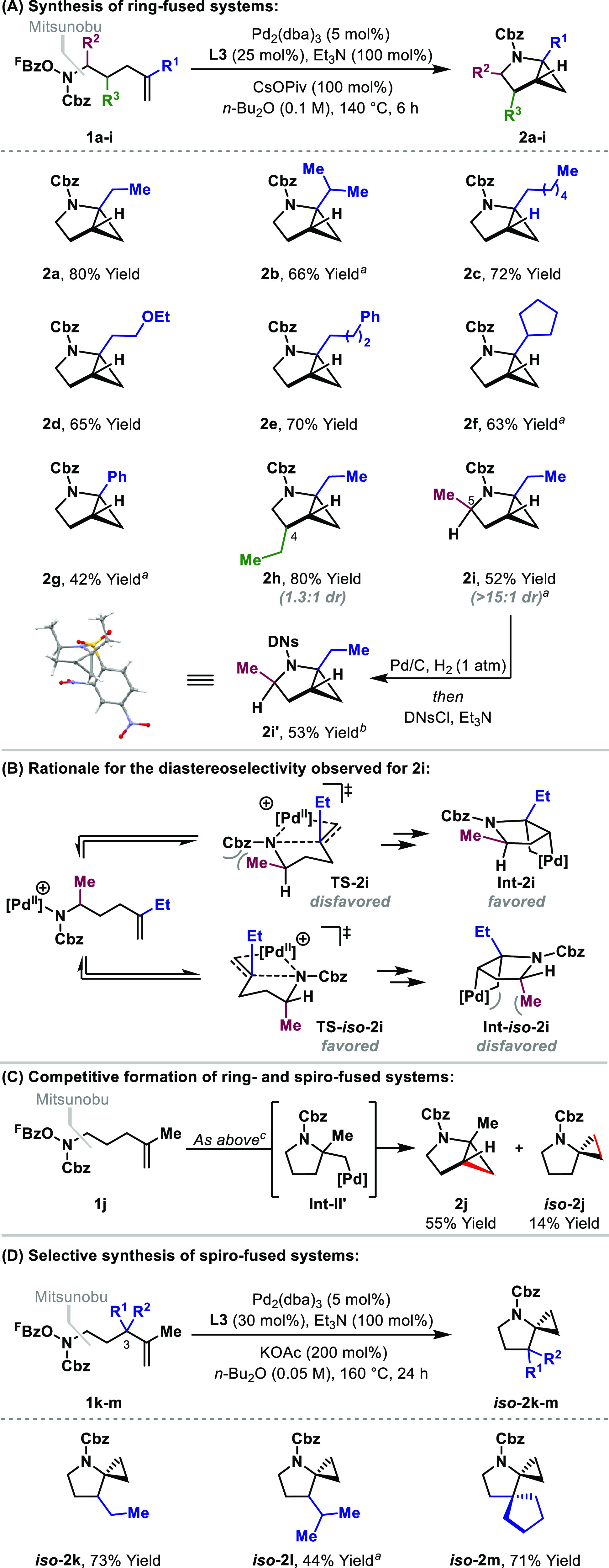
Cyclopropane-Fused Pyrrolidines

a **L3** (50 mol %) was used.

b DNs = 2,4-dinitrophenylsulfonyl.

c **L3** (30 mol %) was used, and the reaction time was 24 h.

Applying the reaction conditions to, for example, the homologue of **1a** did not provide the corresponding cyclopropane-fused piperidine (see the SI), presumably because 6-*exo* aza-palladation is relatively demanding. This limitation can be circumvented by instead using systems that possess a degree of conformational bias (Table 2). Indeed, the cascade cyclization of **1n**, which possesses an aromatic linker, was efficient, delivering the cyclopropane-fused tetrahydroisoquinoline **2n** in a 76% yield. As mentioned earlier, the process is relatively insensitive to the steric demands of the alkene substituent (R^1^), so the bulky isopropyl group of **2p** was well-tolerated. The protocol offers a useful scope with respect to the aromatic component, with both electron-rich (**2q**, **2r**, and **2t**) and electron-poor (**2s**) units participating. Diastereoselective processes are achievable for systems where R^2^ ≠ H; this was demonstrated by the highly stereocontrolled cyclization of **1u** to **2u**, which favored the *syn*-diastereomer (>15:1 dr). For these processes, spiro-fused cyclopropanes (cf. *iso***-2j**) were not observed, which likely reflects the inherent preference for C(sp^3^)–H palladation at the benzylic position. For many of the examples in Table 2, the use of Pd_2_(dba)_3_ as the precatalyst resulted in purification problems because dba coeluted with the product during chromatography. This issue was alleviated by instead using Pd_2_(*p*-MeO-dba)_3_.^16,17^

**Table 2:**
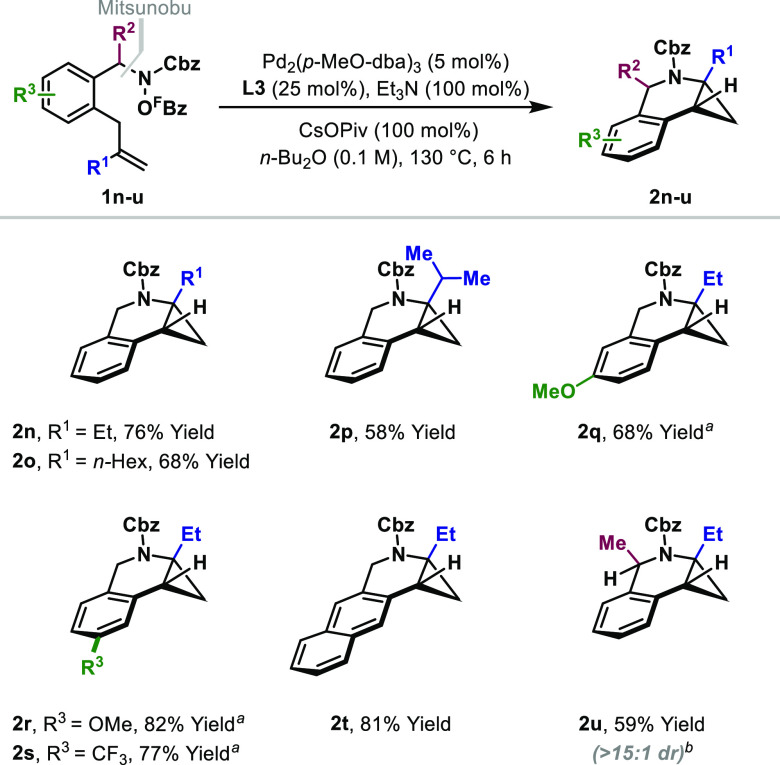
Cyclopropane-Fused Tetrahydroisoquinolines

a Pd_2_(dba)_3_ (5 mol %) was used as the precatalyst.

b **L3** (50 mol %) was used.

We have established that the cascade cyclopropanation procedure can be used to generate reactive donor–acceptor cyclopropanes. A preliminary example process involves the conversion **3a** to piperidine **4a** via initial (nonconformationally biased) 5-*exo* aza-palladation (Table 3A). This generates **Int-II″** in which there is a choice of three different C(sp^3^)–H bonds for metalation, leading to either four- or six-membered palladacycles (not depicted).^18^ The productive pathway involves metalation at the C3 position en route to donor–acceptor cyclopropane **Int-IV**. This is primed for thermally promoted ring opening to provide piperidine **4a**.^19,20^ Accordingly, the rearrangement process transfers the methylidene CH_2_ unit of the starting material (**3a**) to C3 of the target. The metalation selectivity at the stage of **Int-II″** contrasts with the processes in Table 1D; this is presumably due to the electronic activation provided by the ketone substituent, which also allows the process to operate at a lower temperature (110 °C versus 140–160 °C). To improve the efficiency, optimization studies were undertaken, resulting in the conditions shown in Table 3B. The protocol was applicable to a range of systems **3a**–**g** with different substituents at R^1^, R^2^, or R^3^. In general, the processes were efficient, and a mixture of alkene regioisomers was obtained in each case. Based on the mechanistic analysis in Table 3A, the C3–C4 regioisomers of **4a**–**g** result from the isomerization of the initially generated C2–C3 regioisomer under the reaction conditions. Attempts to intercept the donor–acceptor cyclopropane intermediates (**Int-IV**) in cycloaddition processes using either internal (**4d**) or external π-unsaturates (e.g., activated ketones) have so far been unsuccessful.^20^

**Table 3:**
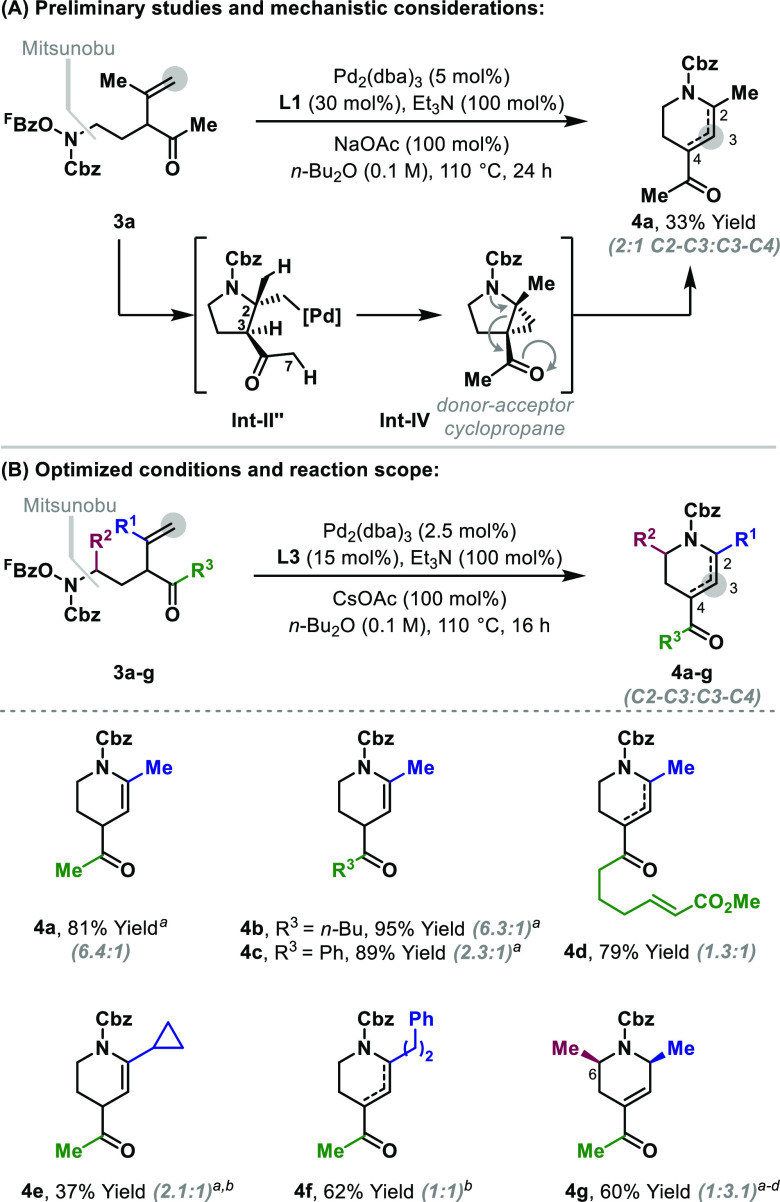
Rearrangement Processes via the Generation of Donor–Acceptor Cyclopropanes

a The major regioisomer is depicted.

b **L3** (25 mol %) was used.

c The reaction time was 42 h, and the regioisomers were isolated in 6:1 and >15:1 d.r., respectively (see the SI).

d The substrate was prepared from (*S*)-propylene oxide.

In summary, we demonstrate the first examples of aza-Heck-triggered C(sp^3^)–H functionalization cascades that lead to ring- or spiro-fused cyclopropanes. To enable these processes, a library of largely novel P ligands was designed and evaluated, from which **L3** emerged as the optimal ligand. The resulting methodology provides an attractive approach to the synthesis of diverse heterocycles containing core cyclopropanes. These are medicinally valuable scaffolds that are challenging to access by other means. From a reactivity viewpoint, our observations have elucidated how steric or electronic control can be used to govern the C(sp^3^)–H metalation selectivity. In broader terms, the processes described here are unusual because they exploit alkene heteropalladation to trigger C(sp^3^)–H cyclopropanation.

## Supporting Information Available

Experimental details, characterization data, and crystallographic data (PDF)
