# Supplementary material for: Cyclopropane-Fused N-Heterocycles via Aza-Heck-Triggered C(sp3)–H Functionalization Cascades
Source: J Am Chem Soc. 2022 Sep 9;144(37):16749–54. doi: 10.1021/jacs.2c08304 (PMC9501755; doi:10.1021/jacs.2c08304)
Supplement: Supplementary file 1 — ja2c08304_si_001.pdf [file ja2c08304_si_001.pdf]

Cyclopropane-Fused N-Heterocycles via Aza-Heck Triggered $C(sp^3)$-H Functionalization Cascades

Changcheng Jing,† Benjamin T. Jones,‡ Ross J. Adams,† and John F. Bower*,†

† Department of Chemistry, University of Liverpool, Crown Street, Liverpool, L69 7ZD, United Kingdom
‡ School of Chemistry, University of Bristol, Bristol, BS8 1TS, United Kingdom

Supporting Information

Table of Contents

General Information ……………………..………………………………………….…….…………….S2

Experimental Procedures and Data ……………………………………….………..…………….…..….S4

Preactivated Reagents for Mitsunobu Alkylation …………………….……..…………..……..S8

Phosphaadamantane Ligand Synthesis…………………………...….….………..….….….……S8

Optimization of the Aza-Heck Cascade ………………………………….…………………….S16

Substrate Synthesis and Catalysis……………………………………….………..……….……S21

Studies on the homologue of **1a**………………………………….……………..………S91

Deuterium-labeling experiment for **1k** to ***iso*-2k**…………………..……………..…….…S92

Representative 2D NMR studies……………..………………………..……………..…..……S93

NMR Spectra for Novel Compounds………………..………………………………………..….S99

Notes and References ………..…..……………………………………….………..………..S225

General Information

Reagents, Solvents and Reactions. Starting materials were purchased from commercial sources (Acros, Aldrich, Alfa Aesar, Fluorochem, TCI, Apollo Scientific) and used without further purification unless otherwise stated. Anhydrous solvents were obtained by passage through drying columns supplied by Anhydrous Engineering Ltd. The removal of solvents in vacuo was achieved using both a Büchi rotary evaporator (bath temperatures up to 40 °C) at a pressure of either 15 mmHg (diaphragm pump) or 0.1 mmHg (oil pump), as appropriate, and a high vacuum line at room temperature. Reactions requiring anhydrous conditions were run under a dry atmosphere of nitrogen or argon; glassware was either flame dried immediately prior to use or placed in an oven (200 °C) for at least 2 hours and allowed to cool either in a desiccator or under an atmosphere of nitrogen or argon; liquid reagents, solutions or solvents were added *via* syringe through rubber septa.

Chromatography. Flash column chromatography (FCC) was performed using Sigma-Aldrich silica gel (60 Å, 230-400 mesh, 40-63 μm). Thin-layer chromatography was performed using aluminium backed 60F254 silica plates. Visualisation was achieved by UV fluorescence or a basic $KMnO_4$ solution and heat.

Spectroscopy. NMR spectra were recorded on Bruker Nano 400, Varian VNMRS500 and Bruker Avance III HD 500 Cryo spectrometers. Chemical shifts (δ) are given in parts per million (ppm) and referenced to the appropriate residual solvent peak. Peaks are described as singlets (s), doublets (d), triplets (t), quartets (q), pentets (pent), sextets (sext), heptets (hept), multiplets (m) and broad (br). Coupling constants (J) are quoted to the nearest 0.5 Hz. Assignments of ^{1}H NMR and ^{13}C NMR signals were made, where possible, using COSY, HSQC, HMBC, and NOE experiments. *Numbering systems for NMR signal assignments are specified on the structure and are not related to those used for the compound names.* In situ yields were determined by integration of the ^{1}H NMR spectrum of the crude material employing 1,3,5-trimethoxybenzene as internal standard. Infra-red (IR) spectra were recorded on a Perkin Elmer Spectrum Two FTIR spectrometer as either neat films or solids compressed on a diamond plate. Only selected absorption maxima (ν_{max}) are reported in wavenumbers (cm^{-1}). Abbreviations used are: weak (w), medium (m), strong (s) and broad (br). High resolution mass spectra (HRMS) were recorded on a VG Analytical Autospec spectrometer by Electron Ionisation (EI) or Chemical Ionisation (CI), a Bruker micrOTOF instrument or a 6200 series TOF/6500 series Q-TOF instrument by Electrospray Ionisation (ESI) or matrix-assisted laser desorption/ionization (MALDI). Mass spectra were determined by a Shimadzu GCMS QP2010+ (EI+ mode), Bruker ultrafleXtreme II TOF/TOF (MALDI, using a colloidal graphite matrix), Brüker Daltonics FT-ICR-MS Apex 4e 7.0T FT-MS (ESI+ mode), Thermo Scientific Orbitrap Elite (APCI mode). Melting points were determined using a Stuart SMP30 melting point apparatus and temperature controller and are uncorrected. Optical

rotation ($[\alpha]_D^T$) were measured using an ADP440+ polarimeter at the concentration and temperature stated.

Naming of Compounds. Compound names are generated by ChemDraw 16.0 software (PerkinElmer), following IUPAC nomenclature.

Experimental Procedures and Data

General Procedure A: Oxidation of primary alcohols

Dess–Martin periodinane (1.4 eq.) was added carefully in portions to a stirred solution of alcohol (1.0 eq.) in DCM (0.5 M) at 0 °C. The reaction was stirred at 0 °C for 20 min. The reaction was quenched with sat. $Na_2S_2O_3$ solution (*approx.* 2 mL/mmol) and sat. $NaHCO_3$ solution (*approx.* 2 mL/mmol). The phases were separated, and the aqueous phase was extracted with DCM (*approx.* 3 × 5 mL/mmol). The combined organic phase was washed with brine (*approx.* 5 mL/mmol), dried over Na_2SO_4, filtered and concentrated *in vacuo*. The residue was purified by FCC under the conditions noted.

General Procedure B: Mannich reaction

A mixture of aldehyde (1.0 eq.), methylamine hydrochloride (0.93 to 1.0 eq.) and formaldehyde (37% wt. in H_2O, 1.0 eq.) was stirred at 70 °C for 48 h. The reaction was diluted with Et_2O (*approx.* 5 mL/mmol) and the aqueous phase was extracted with Et_2O (*approx.* 2 × 5 mL/mmol). The combined organic phase was washed with brine (*approx.* 5 mL/mmol), dried over Na_2SO_4, filtered and concentrated *in vacuo*. The residue was purified by vacuum distillation.

General Procedure C: Reduction of aldehydes

$NaBH_4$ (1.0 eq.) was added carefully in portions to a stirred solution of aldehyde (1.0 eq.) in MeOH (1.5 M) at 0 °C. The reaction was stirred at 0 °C for 30 min, and another 30 min at room temperature. The reaction was quenched with sat. NH_4Cl solution (*approx.* 2 mL/mmol), diluted with water (*approx.* 5 mL/mmol) and extracted with Et_2O (*approx.* 3 × 5 mL/mmol). The organic phase was washed with brine (*approx.* 5 mL/mmol), dried over Na_2SO_4, filtered and concentrated *in vacuo*. The residue was purified by the technique specified in each case.

General Procedure D: Johnson-Claisen rearrangement followed by hydrolysis

A solution of allylic alcohol (1.0 eq.) and propionic acid (0.1 eq.) in triethyl orthoacetate (5.0 eq.) equipped with a short-path distillation apparatus was heated at 140 °C or 160 °C for the time noted (typically 5 h). Upon completion, the reaction mixture was cooled to room temperature, and 1 M aq. HCl solution (*approx.* 1 mL/mmol) was added. The resulted mixture was stirred for 30 min and extracted with Et_2O (*approx.* 3 × 5 mL/mmol). (*If required, the combined organic phase was washed with brine (approx. 5 mL/mmol), dried over Na_2SO_4, filtered and concentrated in vacuo to afford the crude product, which was purified by FCC under the conditions noted.*) The combined organic phase was concentrated *in vacuo*. The residue was then dissolved in MeOH (2.0 M), and an aq. solution of KOH (4.0 M, 10.0 eq.) was added. The reaction was stirred at 60 °C overnight. MeOH was then removed *in vacuo*, and the aqueous phase was washed with Et_2O (*approx.* 2 × 10 mL/mmol) before being acidified (pH = 4~5) with 1.0 M aq. HCl solution. The aqueous phase was extracted with Et_2O

(*approx.* 3 × 10 mL/mmol. The combined organic phase was washed with brine (*approx.* 5 mL/mmol), dried over Na_2SO_4, filtered and concentrated *in vacuo*. The residue was purified by FCC under the conditions noted.

<u>General Procedure E</u>: Reduction of carboxylic acids/esters/amides using $LiAlH_4$

$LiAlH_4$ (2.0 to 3.0 eq.) was added carefully in portions to a solution of carboxylic acid/ester/amide (1.0 eq.) in anhydrous THF (0.7 M) or Et_2O (0.2 to 0.3 M) at 0 °C. The reaction mixture was stirred at the temperature noted and monitored by TLC. Upon completion, the reaction mixture was diluted with Et_2O (*approx.* 10 mL/mmol) and quenched at 0 °C with the addition of water (1 mL/g of $LiAlH_4$), 15% aqueous NaOH (1 mL/g of $LiAlH_4$) and a final portion of water (3 mL/g of $LiAlH_4$). The reaction mixture was stirred at room temperature for around 15 minutes before being dried over Na_2SO_4, filtered and concentrated *in vacuo*. The residue was purified by FCC under the conditions noted.

<u>General Procedure F</u>: Reaction of aliphatic aldehydes with Grignard reagents

The Grignard reagent (1.5 to 2.0 eq.) was added slowly to a solution of aldehyde (1.0 eq.) in anhydrous Et_2O (0.2 M) or THF (0.35 M) at 0 ºC. The reaction mixture was stirred at the temperature noted and monitored by TLC before the addition of sat. NH_4Cl solution (*approx.* 1.5 mL/mmol). The aqueous phase was extracted with Et_2O (*approx.* 3 × 10 mL/mmol). The combined organic phase was washed with brine (*approx.* 5 mL/mmol), dried over Na_2SO_4, filtered and concentrated *in vacuo*. The residue was purified by FCC under the conditions noted.

<u>General Procedure G</u>: Iodination of 2-aminobenzoic acids followed by esterification

A solution of sodium nitrite (2.0 eq.) in water (1.0 M) was added dropwise to a solution of 2-aminobenzoic acid (1.0 eq.) in a mixture of water (0.4 M), acetone (1.27 M), and concentrated HCl (~37% w/w, 0.53 mL/mmol) at 0 °C. After stirring for 2 h, potassium iodide (2.0 eq.) was added to the mixture. The resulting solution was stirred at 0 ºC for 0.5 h, heated at 90 ºC for 10 min, and then allowed to cool to room temperature. The mixture was extracted with $CHCl_3$. The organic layer was washed with sat. $Na_2S_2O_3$ solution, dried over Na_2SO_4, filtered and concentrated *in vacuo*. The resulted 2-iodobenzoic acid was dissolved in CH_2Cl_2 (0.5 M), and oxalyl chloride (2.0 eq.) was added at 0 ºC. Then drops of DMF was added to catalyze the forming of benzoyl chloride. The mixture was stirred at room temperature for another 2 h, then the solvent and excess oxalyl chloride was removed under reduce pressure and methanol (0.33 M) was added to the residue in CH_2Cl_2 (0.33 M). After the mixture was stirred at room temperature for 30 min, the solvents were removed under reduce pressure. The residue was diluted with Et_2O (*approx.* 10 mL/mmol), washed with sat. $NaHCO_3$ solution (*approx.* 2 mL/mmol) and extracted with Et_2O (*approx.* 2 × 10 mL/mmol). The combined organic phase was washed with brine (*approx.* 5 mL/mmol), dried over Na_2SO_4, filtered and concentrated *in vacuo*. The residue was purified by FCC under the conditions noted.

<u>General Procedure H</u>: Reaction of allylic halides with aryl cuprates generated from Grignard reagents

Bromobutane (2.0 eq.) was added dropwisely to a mixture of magnesium turnings (2.4 eq.) and a crystal of iodine in THF (0.25 M). The reaction mixture was stirred for 15 minutes then cooled to -40 °C before dropwise addition of a solution of methyl-2-iodobenzoate (1.0 eq.) in THF (0.25 M). The mixture was stirred at -40 °C for 1.5 h. A freshly prepared solution of LiCl (2.4 eq.) and CuCN (1.2 eq.) in THF (0.25 M) was added and the mixture was stirred for a further 15 min, followed by the addition of allylic halides (3.0 to 4.0 eq.). The mixture was stirred at -40 °C for a further 10 min, then warmed to room temperature. The mixture was diluted with EtOAc (*approx.* 4.5 mL/mmol) and filtered over Celite®. The filtrate was washed with 25% aq. NH_4OH (10 mL/mmol). The aqueous layer was further extracted with EtOAc (*approx.* 2 × 10 mL/mmol). The combined organic phase was washed with brine (*approx.* 5 mL/mmol), dried over Na_2SO_4, filtered and concentrated *in vacuo*. The residue was purified by FCC under the conditions noted.

<u>General Procedure I: Synthesis of l-trimethylsilyl-2-ketones</u>

Freshly prepared $TMSCH_2MgCl$ (1.1 eq.) was added slowly at -78 °C to a suspension of acyl chloride (1.0 eq.) and copper iodide (1.1 eq.) in anhydrous Et_2O (0.33 M). The resulting mixture was stirred for 4 h during which time the temperature was slowly raised to 0 °C. Water was then added to the reaction mixture and the aqueous layer was further extracted with Et_2O (*approx.* 2 × 10 mL/mmol). The combined organic phase was washed with brine (*approx.* 5 mL/mmol), dried over Na_2SO_4, filtered and concentrated *in vacuo* to give the corresponding l-trimethylsilyl-2-ketone which was used without any further purification.

<u>General Procedure J</u>: Cross-aldol reaction of lactones with l-trimethylsilyl-2-ketones

LiHMDS (1.0 M in THF, 1.1 to 1.2 eq.) was added dropwisely to a solution of butyrolactone (1.0 eq.) in THF (0.15 M) at -78 ° C. After stirring for 1 h at this temperature, 1-(trimethylsilyl)-propan-2-one (1.4 eq.) was added rapidly followed by a sat. NH_4Cl solution (approx. 1.5 mL/mmol) after 20 min. The aqueous phase was extracted with Et_2O (approx. 3 × 10 mL/mmol). The combined organic phase was washed with brine (approx. 5 mL/mmol), dried over Na_2SO_4, filtered and concentrated in *vacuo*. The residue was purified by FCC under the conditions noted.

<u>General Procedure K</u>: Peterson elimination to terminal alkenes

HF-pyridine (amount specified) was added to a stirring solution of the preceding lactone (1.0 eq.) in THF (0.083 M) or CH_3CN (0.060 M) at 0 °C. After 40 minutes the reaction was allowed to warm to room temperature and stirred for a further 10 h. The reaction was quenched by pouring it into a 1:1 mixture of Et_2O and a saturated aqueous solution of $NaHCO_3$ (approx. 7.2 mL/mmol) followed by vigorous stirring for 10 min. The aqueous phase was extracted with Et_2O (approx. 2 × 10 mL/mmol.

The combined organic phase was washed with brine (approx. 5 mL/mmol), dried over Na_2SO_4, filtered and concentrated in *vacuo*. The residue was purified by FCC under the conditions noted.

<u>General Procedure L</u>: Reaction of lactones or esters, *N*-methoxy-*N*-methylamine with Grignard reagents

A slurry of lactone or ester (1.0 eq.), HN(OMe)Me·HCl (1.2 eq.) and NaOMe (0.25 eq.) in THF (0.05 M) was cooled to -20 ° C. A solution of Grignard reagent (8.0 eq.) was added slowly. After 2 h the reaction mixture was allowed to warm to room temperature and stirred for 8 h. The reaction was quenched with sat. NH_4Cl solution (approx. 1.5 mL/mmol). The aqueous phase was extracted with Et_2O (approx. 3 × 10 mL/mmol). The combined organic phase was washed with brine (approx. 5 mL/mmol), dried over Na_2SO_4, filtered and concentrated in *vacuo*. The residue was purified by FCC under the conditions noted.

<u>General Procedure M</u>: TBS deprotection to alcohols using TBAF or TBAF/AcOH

A solution of TBAF (equivalents specified, 1.0 M in THF) or 1:1 TBAF/AcOH (equivalents specified, 1.0 M in THF) was added to a solution of silyl ether (1.0 eq.) in THF (0.25 M) at 0 °C. The reaction mixture was stirred at room temperature and monitored by TLC. Upon completion, the reaction mixture was quenched with water (approx. 1.5 mL/mmol) and the aqueous phase was extracted with Et_2O (approx. 3 × 10 mL/mmol). The combined organic phase was washed with brine (approx. 5 mL/mmol), dried over Na_2SO_4, filtered and concentrated in *vacuo*. The residue was purified by FCC under the conditions noted.

<u>General Procedure N</u>: Mitsunobu reactions

Triphenylphosphine (1.1 to 1.5 eq.) and carbamate (0.9 to 1.2 eq.) were added to a flame dried flask under nitrogen. Anhydrous solvent (0.1 to 0.4 M) was added (THF for OTs and OPiv systems, toluene/THF 4:1 for O^FBz systems) and the reaction was cooled to 0 °C before adding the alcohol (1.0 eq.) *via* syringe. Diisopropyl azodicarboxylate (1.1 to 1.5 eq.) was then added and the reaction was stirred at room temperature for 16 h. Upon completion, the reaction mixture was concentrated *in vacuo* and the residue was purified by FCC under the conditions noted.

<u>General Procedure O</u>: Aza-Heck/C-H functionalization cascade reactions

A flame-dried Schlenk tube, fitted with a rubber septum, was charged with cyclization substrate, $Pd_2(dba)_3$, phosphine ligand and the carboxylate salt additive. The tube was purged with nitrogen, and anhydrous solvent and triethylamine were added *via* syringe. The tube was sealed with a plastic cap and stirred at the specified temperature for the time noted. The reaction mixture was concentrated *in vacuo* and the crude mixture was purified by FCC under the conditions noted to afford the pure product.

Preactivated Substrates for Mitsunobu Alkylation

Benzyl ((perfluorobenzoyl)oxy)carbamate

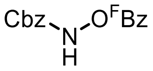

This compound was prepared according to our previously reported literature procedure.[1]

The spectroscopic properties were consistent with the data available in the literature.[1]

Benzyl (tosyloxy)carbamate

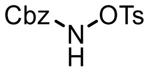

This compound was prepared according to our previously reported literature procedure.[2]

The spectroscopic properties were consistent with the data available in the literature.[2]

Benzyl (pivaloyloxy)carbamate

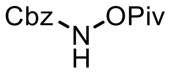

Pivaloyl chloride (0.62 mL, 5.00 mmol) and triethylamine (1.05 mL, 7.50 mmol) were added to a solution of benzyl hydroxycarbamate (836 mg, 5.00 mmol) in CH_2Cl_2 (30 mL) at 0 °C. The reaction was stirred at room temperature for 24 h. Upon completion, the reaction was diluted with water (100 mL), and the aqueous layer was extracted with CH_2Cl_2 (3 × 50 mL). The combined organic extracts were washed with brine (50 mL), dried over Na_2SO_4, filtered and concentrated in *vacuo*. The residue was purified by FCC (Hexane/EtOAc = 85:15) to give the title compound (1.08 g, 86%) as a colorless oil. IR (thin film) ν_{max}/cm^{-1}: 3281, 2976, 1778, 1748, 1242, 1105, 736; ^{1}H NMR (400 MHz, $CDCl_3$) δ_H = 8.02 (br s, 1H, N$\underline{H}$), 7.44 – 7.31 (m, 5H, ArC$\underline{H}$), 5.21 (s, 2H, Cbz C$\underline{H_2}$), 1.29 (s, 9H, Piv C(C$\underline{H_3}$)$_3$); ^{13}C NMR (101 MHz, $CDCl_3$) δ_C = 177.7 (Piv $\underline{C}$=O), 156.6 (Cbz $\underline{C}$=O), 135.2 (Cbz Ar$\underline{C}$), 128.8 (Ar$\underline{C}$H), 128.8 (Ar$\underline{C}$H), 128.5 (Ar$\underline{C}$H), 68.5 (Cbz $\underline{C}H_2$), 38.4 (Piv $\underline{C}(CH_3)_3$), 27.1 (Piv C($\underline{C}H_3)_3$); HRMS (ESI$^+$) calculated for $C_{13}H_{17}NNaO_4$ [M+Na]$^+$ = 274.1050, found 274.1061.

Phosphaadamantane Ligand synthesis

8-(4-Methoxyphenyl)-1,3,5,7-tetramethyl-2,4,6-trioxa-8-phosphaadamantane

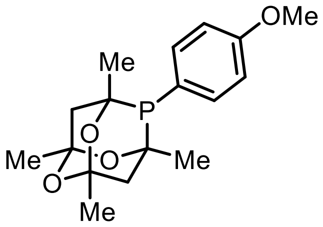

This compound was prepared according to our previously reported literature procedure.[1]

The spectroscopic properties were consistent with the data available in the literature.[1]

8-(3,5-Bis(trifluoromethyl)phenyl)-1,3,5,7-tetramethyl-2,4,6-trioxa-8-phosphaadamantane

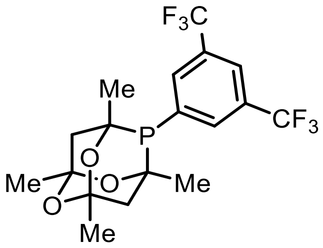

This compound was prepared according to our previously reported literature procedure.[3]

The spectroscopic properties were consistent with the data available in the literature.[3]

Ethyl 4-(1,3,5,7-tetramethyl-2,4,6-trioxa-8-phosphaadamantan-8-yl)benzoate

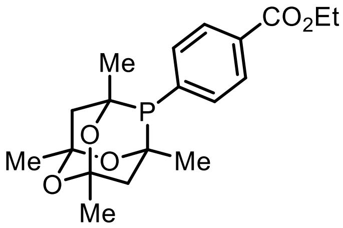

This compound was prepared according to our previously reported literature procedure.[1]

The spectroscopic properties were consistent with the data available in the literature.[1]

1,3,5,7-Tetramethyl-8-(4-nitrophenyl)-2,4,6-trioxa-8-phosphaadamantane

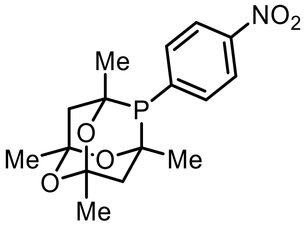

This compound was prepared according to our previously reported literature procedure.[1]

The spectroscopic properties were consistent with the data available in the literature.[1]

8-(3,5-Difluoro-4-nitrophenyl)-1,3,5,7-tetramethyl-2,4,6-trioxa-8-phosphaadamantane

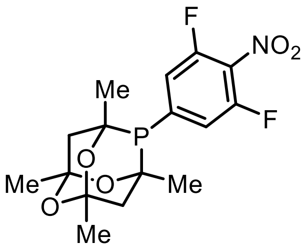

This compound was prepared according to our previously reported literature procedure.[3]

The spectroscopic properties were consistent with the data available in the literature.[3]

1,3,5,7-Tetramethyl-8-(thiophen-2-yl)-2,4,6-trioxa-8-phosphaadamantane

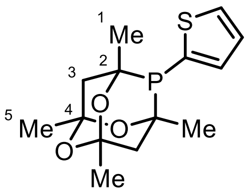

A suspension of 1,3,5,7-tetramethyl-2,4,6-trioxa-8-phosphaadamantane (432 mg, 2.00 mmol), $Pd(PPh_3)_4$ (69.3 mg, 0.06 mmol), 2-bromothiophene (326 mg, 2.00 mmol) and K_2CO_3 (829 mg, 6.00 mmol) in anhydrous toluene (10.0 mL) was heated at 110 °C for 17 h. The reaction mixture was cooled to room temperature before being filtered through silica and rinsed with Et_2O. The filtrate was concentrated *in vacuo*. The residue was purified by FCC (Toluene) to afford the title compound (323 mg, 54 %) as a colorless solid. m.p. 92-94 °C (CH_2Cl_2:petrol); IR (thin film) ν_{max}/cm^{-1}: 3100, 2962, 2911, 1450, 1380 , 1343, 1212; ^{13}C NMR (500 MHz, $CDCl_3$) δ_c = 7.63 (dd, *J* = 4.9, 1.2 Hz, 1H, ArC<u>H</u>), 7.53 (ddd, *J* = 7.2, 3.5, 1.2 Hz, 1H, ArC<u>H</u>), 7.13 (ddd, *J* = 4.9, 3.5, 1.3 Hz, 1H, ArC<u>H</u>), 2.11 (d, *J* = 13.5 Hz, 1H, C**3'**-<u>H</u>), 2.05 (dd, *J* = 13.3, 7.4 Hz, 1H, C**3**-<u>H</u>), 1.96 (dd, *J* = 25.0, 13.3 Hz, 1H, C**3**-<u>H</u>'), 1.54 (dd, *J* = 13.5, 4.4 Hz, 1H, C**3'**-<u>H</u>'), 1.45 (s, 3H, C**5**-<u>H3</u>), 1.42 (s, 3H, C**5'**-<u>H3</u>), 1.40 (3H, d, *J* = 12.9 Hz, C**1**-<u>H3</u>), 1.26 (d, *J* = 13.9 Hz, 3H, C**1'**-<u>H3</u>); ^{13}C NMR (125 MHz, $CDCl_3$) δ_c = 139.2 (d, *J* = 29.1 Hz, Ar<u>C</u>H), 132.9 (Ar<u>C</u>H), 132.0 (d, *J* = 45.4 Hz, Ar<u>C</u>), 127.5 (d, *J* = 9.2 Hz, Ar<u>C</u>H), 97.0 (C**4**), 96.4 (C**4'**), 73.2 (d, *J* = 16.7 Hz, C**2**), 73.2 (d, *J* = 8.4 Hz, C**2'**), 45.0 (d, *J* = 17.8 Hz, C**3**), 37.0 (d, *J* = 2.1 Hz, C**3'**), 28.1 (C**5'**), 27.9 (C**5**), 27.7 (d, *J* = 23.4 Hz, C**1'**), 27.1 (d, *J* = 10.9 Hz, C**1**); ^{31}P NMR (202 MHz, $CDCl_3$) δ_P = -34.3; HRMS (ESI$^+$) calculated for $C_{14}H_{20}O_3SP$ $[M+H]^+$ = 299.0865, found 299.0874.

1,3,5,7-Tetramethyl-8-(5-acetylthiophen-2-yl)-2,4,6-trioxa-8-phosphaadamantane

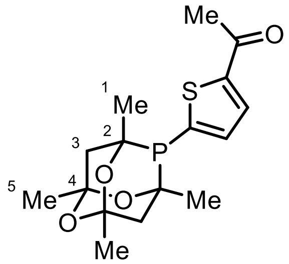

A suspension of 1,3,5,7-tetramethyl-2,4,6-trioxa-8-phosphaadamantane (432 mg, 2.00 mmol), $Pd(PPh_3)_4$ (69.3 mg, 0.06 mmol), 2-acetyl-5-bromothiophene (410 mg, 2.00 mmol) and K_2CO_3 (829 mg, 6.00 mmol) in anhydrous toluene (10.0 mL) was heated at 110 °C for 22 h. The reaction mixture was cooled to room temperature before being filtered through silica and rinsed with Et_2O. The filtrate was concentrated *in vacuo*. The residue was purified by FCC (Toluene:acetone = 99:1 to 98:2) to afford the title compound (377 mg, 55 %) as a colorless solid. m.p. 104-105 °C (CH_2Cl_2:petrol); IR (thin film) ν_{max}/cm^{-1}: 2969, 2915, 1662, 1514, 1213; ^{1}H NMR (500 MHz, $CDCl_3$) δ_H = 7.66 (dd, *J* = 3.8, 1.2 Hz, 1H, ArCH), 7.50 (dd, *J* = 6.3, 3.8 Hz, 1H, ArCH), 2.57 (s, 3H, C(O)CH_3), 2.04 – 1.94 (m, 3H, C**3**-H_2 + C**3'**-H), 1.56 (dd, *J* = 13.6, 4.5 Hz, 1H, C**3'**-H'), 1.45 (s, 3H, C**5**-H_3), 1.43 (d, *J* = 13.6 Hz, 3H, C**1**-H_3), 1.42 (s, 3H, C**5'**-H_3), 1.29 (d, *J* = 14.0 Hz, 3H, C**1'**-H_3). ^{13}C NMR (125 MHz, $CDCl_3$) δ_c = 190.5 (C(O)CH_3), 150.1 (ArC), 142.4 (d, *J* = 51.2 Hz, ArC), 138.9 (d, *J* = 27.9 Hz, ArCH), 132.1 (d, *J* = 8.5 Hz, ArCH), 97.0 (C**4**), 96.4 (C**4'**), 73.4 (d, *J* = 7.9 Hz, C**2'**), 73.1 (d, *J* = 18.7 Hz, C**2**), 44.6 (d, *J* = 17.7 Hz, C**3**), 37.0 (d, *J* = 2.1 Hz, C**3'**), 27.9 (d, *J* = 22.6 Hz, C**1'**), 27.8 (C**5'**), 27.7 (C**5**), 27.3 (C(O)CH_3), 27.1 (d, *J* = 10.8 Hz, C**1**); ^{31}P NMR (202 MHz, $CDCl_3$) δ_P = -32.5; HRMS (ESI$^+$) calculated for $C_{16}H_{22}O_4SP$ $[M+H]^+$ = 341.0710, found 341.0980.

8-(Furan-2-yl)-1,3,5,7-tetramethyl-2,4,6-trioxa-8-phosphaadamantane

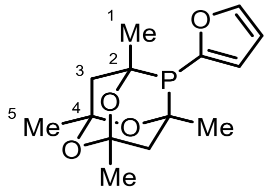

A suspension of 1,3,5,7-tetramethyl-2,4,6-trioxa-8-phosphaadamantane (324 mg, 1.50 mmol), $Pd(PPh_3)_4$ (52.0 mg, 0.045 mmol), 2-bromofuran (133 µL, 1.50 mmol) and K_2CO_3 (622 mg, 4.50 mmol) in anhydrous toluene (7.50 mL) was heated at 110 °C for 20 h. The reaction mixture was cooled to room temperature before being filtered through silica and rinsed with Et_2O. The filtrate was concentrated *in vacuo*. The residue was purified by FCC (Hexane/Et_2O = 90:10) to afford the title compound (377 mg, 89%) as a colorless solid. m.p. 90 - 92 °C (CH_2Cl_2/hexane); IR (thin film) ν_{max}/cm^{-1}: 2991, 2920, 1449, 1378, 1213, 978; ^{1}H NMR (400 MHz, $CDCl_3$) δ_H = 7.67 (ddd, *J* = 1.6, 0.8, 0.8 Hz, 1H, ArCH), 6.93 (ddd, *J* = 3.4, 1.6, 0.8 Hz, 1H, ArCH), 6.45 (ddd, *J* = 3.4, 1.6, 0.8 Hz, 1H, ArCH), 2.04 (d, *J* = 13.2 Hz, 1H, C**3**-H), 2.02 – 1.88 (m, 2H, C**3'**-H_2), 1.60 (dd, *J* = 13.2, 4.4 Hz, 1H, C**3**-H'), 1.43 (s, 3H, C**5**-H_3),

1.41 (s, 3H, C**5’**-H3), 1.38 (d, *J* = 13.1 Hz, 3H, C**1**-H3), 1.31 (d, *J* = 13.8 Hz, 3H, C**1’**-H3); ^{13}C NMR (101 MHz, $CDCl_3$) δ_C = 150.2 (d, *J* = 29.9 Hz, ArC), 147.5 (d, *J* = 2.7 Hz, ArCH), 122.7 (d, *J* = 13.8 Hz, ArCH), 110.7 (d, *J* = 4.1 Hz, ArCH), 97.0 (C**4**), 96.4 (C**4’**), 72.9 (d, *J* = 8.6 Hz, C**2**), 72.9 (d, *J* = 13.2 Hz, C**2’**), 45.0 (d, *J* = 18.3 Hz, C**3’**), 38.1 (d, *J* = 2.4 Hz, C**3**), 28.2 (d, *J* = 22.4 Hz, C**1’**), 28.2 (C**5**), 27.9 (C**5’**), 27.1 (d, *J* = 10.8 Hz, C**1**); ^{31}P NMR (162 MHz, $CDCl_3$) δ_P = -44.6; HRMS (ESI$^+$) calculated for $C_{14}H_{20}O_5P$ [M+O+H]$^+$ = 299.1048, found 299.1047 (*phosphine oxide peak found*).

8-(5-Ethylfuran-2-yl)-1,3,5,7-tetramethyl-2,4,6-trioxa-8-phosphaadamantane

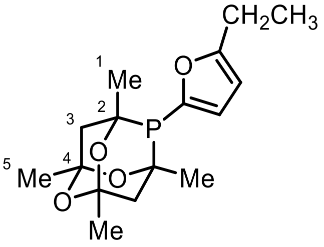

A suspension of 1,3,5,7-tetramethyl-2,4,6-trioxa-8-phosphaadamantane (324 mg, 1.50 mmol), $Pd(PPh_3)_4$ (52.0 mg, 0.045 mmol), 2-bromo-5-ethylfuran (263 mg, 1.50 mmol) and K_2CO_3 (622 mg, 4.50 mmol) in anhydrous toluene (7.50 mL) was heated at 110 °C for 20 h. The reaction mixture was cooled to room temperature before being filtered through silica and rinsed with Et_2O. The filtrate was concentrated *in vacuo*. The residue was purified by FCC (Hexane/Et_2O = 90:10) to afford the title compound (366 mg, 79%) as a colorless solid. m.p. 60 - 62 °C (Et_2O/hexane); IR (thin film) ν_{max}/cm^{-1}: 2970, 2919, 1586, 1439, 1377, 1214, 978; ^{1}H NMR (400 MHz, $CDCl_3$) δ_H = 6.95 – 6.68 (m, 1H, ArCH), 6.15 – 5.89 (m, 1H, ArCH), 2.67 (q, *J* = 7.6 Hz, 2H, CH_2CH_3), 2.09 (d, *J* = 13.1 Hz, 1H, C**3**-H), 2.03 – 1.84 (m, 2H, C**3’**-H2), 1.57 (dd, *J* = 13.1, 4.3 Hz, 1H, C**3**-H’), 1.41 (s, 3H, C**5**-H3), 1.39 (s, 3H, C**5’**-H3), 1.35 (d, *J* = 13.4 Hz, 3H, C**1**-H3), 1.29 (d, *J* = 13.4 Hz, 3H, C**1’**-H3), 1.22 (t, *J* = 7.6 Hz, 3H, CH_2CH_3); ^{13}C NMR (101 MHz, $CDCl_3$) δ_C = 163.1 (d, *J* = 2.5 Hz, ArC), 147.8 (d, *J* = 26.5 Hz, ArC), 123.6 (d, *J* = 13.2 Hz, ArCH), 105.5 (d, *J* = 4.1 Hz, ArCH), 96.9 (C**4**), 96.3 (C**4’**), 72.9 (d, *J* = 11.0 Hz, C**2**), 72.8 (d, *J* = 10.4 Hz, C**2’**), 45.0 (d, *J* = 18.3 Hz, C**3’**), 38.0 (d, *J* = 2.3 Hz, C**3**), 28.2 (d, *J* = 22.4 Hz, C**1’**), 28.2 (C**5**), 27.9 (C**5’**), 27.1 (d, *J* = 10.9 Hz, C**1**), 21.8 (CH_2CH_3), 12.1 (CH_2CH_3); ^{31}P NMR (162 MHz, $CDCl_3$) δ_P = -44.7; HRMS (ESI$^+$) calculated for $C_{16}H_{24}O_4P$ [M+H]$^+$ = 311.1408, found 311.1411.

1-(5-(1,3,5,7-Tetramethyl-2,4,6-trioxa-8-phosphaadamantan-8-yl)furan-2-yl)ethan-1-one

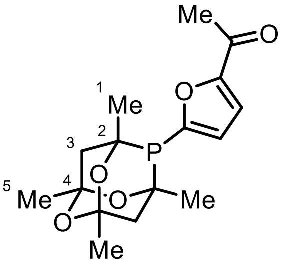

A suspension of 1,3,5,7-tetramethyl-2,4,6-trioxa-8-phosphaadamantane (162 mg, 0.75 mmol), $Pd(PPh_3)_4$ (26.0 mg, 0.0225 mmol), 1-(5-bromofuran-2-yl)ethan-1-one (142 mg, 0.75 mmol) and K_2CO_3 (311 mg, 2.25 mmol) in anhydrous toluene (3.80 mL) was heated at 110 °C for 20 h. The reaction mixture was cooled to room temperature before being filtered through silica and rinsed with Et_2O. The filtrate was concentrated *in vacuo*. The residue was purified by FCC (Hexane/Et_2O = 90:10) to afford the title compound (207 mg, 85%) as a colorless solid. m.p. 95 - 97 °C (Et_2O/hexane); IR (thin film) ν_{max}/cm^{-1}: 2964, 2917, 1682, 1451, 1378, 1215, 978; ^{1}H NMR (400 MHz, $CDCl_3$) δ_H = 7.18 (d, *J* = 3.4 Hz, 1H, ArC<u>H</u>), 7.00 (d, *J* = 3.4 Hz, 1H, ArC<u>H</u>), 2.49 (s, 3H, ArCOC<u>H</u>$_3$), 2.01 – 1.86 (m, 3H, C**3**-<u>H</u> + C**3'**-<u>H</u>$_2$), 1.61 (dd, *J* = 13.3, 4.4 Hz, 1H, C**3**-<u>H</u>'), 1.41 (d, *J* = 13.1 Hz, 3H, C**1**-<u>H</u>$_3$), 1.40 (s, 3H, C**5**-<u>H</u>$_3$), 1.40 (s, 3H, C**5'**-<u>H</u>$_3$), 1.36 (d, *J* = 13.9 Hz, 3H, C**1'**-<u>H</u>$_3$); ^{13}C NMR (101 MHz, $CDCl_3$) δ_C = 186.9 (Ar<u>C</u>OCH$_3$), 156.7 (d, *J* = 2.5 Hz, Ar<u>C</u>), 156.4 (d, *J* = 34.9 Hz, Ar<u>C</u>), 123.1 (d, *J* = 7.9 Hz, Ar<u>C</u>H), 116.9 (d, *J* = 2.4 Hz, Ar<u>C</u>H), 97.0 (C**4**), 96.3 (C**4'**), 72.9 (d, *J* = 23.5 Hz, C**2'**), 72.8 (d, *J* = 35.5 Hz, C**2**), 44.5 (d, *J* = 18.2 Hz, C**3'**), 38.1 (d, *J* = 2.3 Hz, C**3**), 28.4 (d, *J* = 22.2 Hz, C**1'**), 28.1 (C**5**), 27.8 (C**5'**), 27.2 (d, *J* = 10.7 Hz, C**1**), 26.4 (ArCO<u>C</u>H$_3$); ^{31}P NMR (162 MHz, $CDCl_3$) δ_P = -43.3; HRMS (ESI$^+$) calculated for $C_{16}H_{22}O_5P$ $[M+H]^+$ = 325.1199, found 325.1198.

Methyl 5-(1,3,5,7-tetramethyl-2,4,6-trioxa-8-phosphaadamantan-8-yl)furan-2-carboxylate

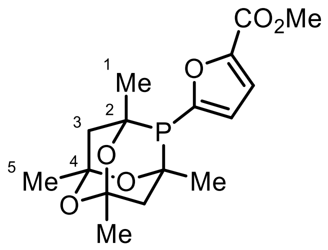

A suspension of 1,3,5,7-tetramethyl-2,4,6-trioxa-8-phosphaadamantane (324 mg, 1.50 mmol), $Pd(PPh_3)_4$ (52.0 mg, 0.045 mmol), methyl-5-bromo-2-furoate (308 mg, 1.50 mmol) and K_2CO_3 (622 mg, 4.50 mmol) in anhydrous toluene (7.50 mL) was heated at 110 °C for 20 h. The reaction mixture was cooled to room temperature before being filtered through silica and rinsed with Et_2O. The filtrate was concentrated *in vacuo*. The residue was purified by FCC (Hexane/Et_2O = 85:15) to afford the title compound (457 mg, 90%) as a colorless solid. m.p. 114 – 115 °C (CH_2Cl_2/hexane); IR (thin film) ν_{max}/cm^{-1}: 2966, 1723, 1293, 1199, 1132; ^{1}H NMR (500 MHz, $CDCl_3$) δ_H = 7.19 (d, *J* = 3.5 Hz, 1H, ArC<u>H</u>), 7.00 (d, *J* = 3.5 Hz, 1H, ArC<u>H</u>), 3.89 (s, 3H, CO$_2$C<u>H</u>$_3$), 2.03 – 1.86 (m, 3H, C**3**-<u>H</u> + C**3**-<u>H</u>' + C**3'**-<u>H</u>), 1. 61 (dd, *J* = 13.3, 4.4 Hz, 1H, C**3'**-<u>H</u>'), 1.42 (s, 3H, C**5**-<u>H</u>$_3$), 1.41 (s, 3H, C**5'**-<u>H</u>$_3$), 1.40 (d, *J* = 13.2 Hz, 3H, C**1**-<u>H</u>$_3$), 1.36 (d, *J* = 13.9 Hz, 3H, C**1'**-<u>H</u>$_3$); ^{13}C NMR (125 MHz, $CDCl_3$) δ_C = 158.9 (<u>C</u>O$_2$CH$_3$), 156.5 (d, *J* = 33.3 Hz, Ar<u>C</u>), 148.6 (d, *J* = 3.3 Hz, Ar<u>C</u>), 122.6 (d, *J* = 6.4 Hz, Ar<u>C</u>H), 118.3 (d, *J* = 2.1 Hz, Ar<u>C</u>H), 97.0 (C**4**), 96.3 (C**4'**), 73.0 (d, *J* = 5.9 Hz, C**2**), 72.7 (d, *J* = 18.1 Hz, C**2'**), 52.2 (CO$_2$<u>C</u>H$_3$), 44.6 (d, *J* = 18.2 Hz, C**3'**), 38.0 (d, *J* = 2.2 Hz, C**3**), 28.5 (d, *J* = 22.2 Hz, C**1'**), 28.1 (C**5**),

27.8 (C**5'**), 27.2 (d, *J* = 10.8 Hz, C**1**); ^{31}P NMR (202 MHz, $CDCl_3$) δ_P = -43.9; HRMS (ESI$^+$) calculated for $C_{16}H_{21}O_6PNa$ $[M+Na]^+$ = 363.0973, found 363.0969.

2-(1,3,5,7-Tetramethyl-2,4,6-trioxa-8-phosphaadamantan-8-yl)pyridine

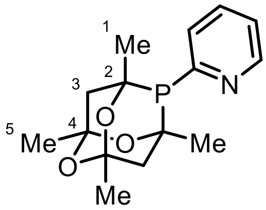

A suspension of 1,3,5,7-tetramethyl-2,4,6-trioxa-8-phosphaadamantane (324 mg, 1.50 mmol), $Pd(PPh_3)_4$ (52.0 mg, 0.045 mmol), 2-bromo pyridine (0.14 mL, 1.50 mmol) and K_2CO_3 (622 mg, 4.50 mmol) in anhydrous toluene (7.50 mL) was heated at 110 °C for 20 h. The reaction mixture was cooled to room temperature before being filtered through silica and rinsed with Et_2O. The filtrate was concentrated *in vacuo*. The residue was purified by FCC (Hexane/Et_2O = 85:15) to afford the title compound (400 mg, 91%) as a colorless solid. m.p. 100 – 102 °C (CH_2Cl_2/hexane) [Lit.: 99.5 – 102.5 °C][4]; ^{1}H NMR (400 MHz, $CDCl_3$) δ_H = 8.72 (m, 1H, ArC<u>H</u>), 8.03 (d, *J* = 8.0 Hz, 1H, ArC<u>H</u>), 7.69 (m, 1H, ArC<u>H</u>), 7.26 (m, 1H, ArC<u>H</u>), 2.09 (dd, *J* = 13.5, 7.0 Hz, 1H, C**3**-<u>H</u>), 1.92 (dd, *J* = 23.5, 13.5 Hz, 1H, C**3**-<u>H</u>'), 1.75 (d, *J* = 13.5 Hz, 1H, C**3'**-<u>H</u>), 1.58 (d, *J* = 12.5 Hz, 3H, C**1**-<u>H₃</u>), 1.52 (dd, *J* = 13.5, 4.0 Hz, 1H, C**3'**-<u>H</u>'), 1.43 (d, *J* = 12.5 Hz, 3H, C**1'**-<u>H₃</u>), 1.42 (s, 3H, C**5**-<u>H₃</u>), 1.38 (s, 3H, C**5'**-<u>H₃</u>); ^{13}C NMR (101 MHz, $CDCl_3$) δ_c = 160.7 (m, Ar<u>C</u>), 149.8 (m, Ar<u>C</u>H), 136.1 (Ar<u>C</u>H), 129.6 (d, *J* = 8.5 Hz, Ar<u>C</u>H), 123.2 (Ar<u>C</u>H), 96.9 (C**4**), 96.3 (C**4'**), 73.6 (d, *J* = 10.0 Hz, C**2**), 73.1 (d, *J* = 23.0 Hz, C**2'**), 45.1 (d, *J* = 17.0 Hz, C**3**), 37.5 (d, *J* = 2.0 Hz, C**3'**), 28.1 (C**5'**), 28.0 (d, *J* = 20.5 Hz, C**1'**), 27.9 (C**5**), 27.2 (d, *J* = 11.5 Hz, C**1**); ^{31}P NMR (162 MHz, $CDCl_3$) δ_p = -25.8.

The spectroscopic properties were consistent with the data available in the literature.[4]

8-(Benzofuran-2-yl)-1,3,5,7-tetramethyl-2,4,6-trioxa-8-phosphaadamantane

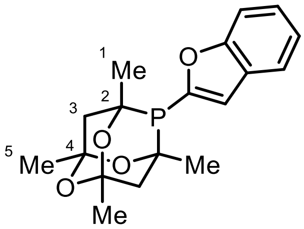

A suspension of 1,3,5,7-tetramethyl-2,4,6-trioxa-8-phosphaadamantane (260 mg, 1.20 mmol), $Pd(PPh_3)_4$ (41.6 mg, 0.036 mmol), 2-bromobenzofuran (248 mg, 1.26 mmol) and K_2CO_3 (498 mg, 3.60 mmol) in anhydrous toluene (6.00 mL) was heated at 110 °C for 20 h. The reaction mixture was cooled to room temperature before being filtered through silica and rinsed with Et_2O. The filtrate was concentrated *in vacuo*. The residue was purified by FCC (Hexane/Et_2O = 90:10) to afford the title

compound (371 mg, 93%) as a colorless solid. m.p. 93 - 95 °C (Et_2O/hexane); IR (thin film) ν_{max}/cm^{-1}: 2966, 2940, 2917, 1442, 1374, 1214, 978; ^{1}H NMR (500 MHz, $CDCl_3$) δ_H = 7.60 (d, *J* = 7.7 Hz, 1H, ArC<u>H</u>), 7.55 – 7.49 (m, 1H, ArC<u>H</u>), 7.32 (ddd, *J* = 8.4, 7.2, 1.3 Hz, 1H, ArC<u>H</u>), 7.31 – 7,29 (m, 1H, ArC<u>H</u>), 7.26 – 7.22 (m, 1H, ArC<u>H</u>), 2.10 (d, *J* = 13.3 Hz, 1H, C**3**-<u>H</u>), 2.07 – 1.93 (m, 2H, C**3'**-<u>H</u>$_2$), 1.62 (dd, *J* = 13.3, 4.4 Hz, 1H, C**3**-<u>H</u>'), 1.49 (d, *J* = 13.1 Hz, 3H, C**1'**-<u>H</u>$_3$), 1.46 (s, 3H, C**5'**-<u>H</u>$_3$), 1.44 (s, 3H, C**5**-<u>H</u>$_3$), 1.40 (d, *J* = 13.8 Hz, 3H, C**1**-<u>H</u>$_3$); ^{13}C NMR (125 MHz, $CDCl_3$) δ_C = 157.6 (d, *J* = 3.7 Hz, Ar<u>C</u>), 154.2 (d, *J* = 30.9 Hz, Ar<u>C</u>), 127.9 (d, *J* = 3.6 Hz, Ar<u>C</u>), 125.3 (Ar<u>C</u>H), 123.0 (Ar<u>C</u>H), 121.4 (Ar<u>C</u>H), 111.6 (Ar<u>C</u>H), 118.2 (d, *J* = 9.6 Hz, Ar<u>C</u>H), 97.1 (C**4**), 96.4 (C**4'**), 73.1 (d, *J* = 30.4 Hz, C**2**), 72.9 (d, *J* = 42.9 Hz, C**2'**), 44.8 (d, *J* = 18.2 Hz, C**3'**), 38.1 (d, *J* = 2.2 Hz, C**3**), 28.4 (d, *J* = 22.2 Hz, C**1**), 28.2 (C**5**), 27.9 (C**5'**), 27.2 (d, *J* = 10.8 Hz, C**1'**); ^{31}P NMR (202 MHz, $CDCl_3$) δ_P = -42.7; HRMS (ESI$^+$) calculated for $C_{18}H_{22}O_4P$ $[M+H]^+$ = 333.1250, found 333.1250.

8-(Benzo[*b*]thiophen-2-yl)-1,3,5,7-tetramethyl-2,4,6-trioxa-8-phosphaadamantane

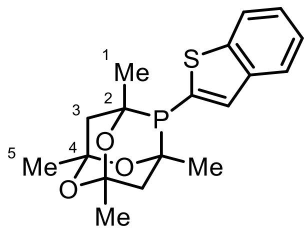

A suspension of 1,3,5,7-tetramethyl-2,4,6-trioxa-8-phosphaadamantane (324 mg, 1.50 mmol), $Pd(PPh_3)_4$ (52.0 mg, 0.045 mmol), 2-bromobenzo[*b*]thiophene (320 mg, 1.50 mmol) and K_2CO_3 (622 mg, 4.50 mmol) in anhydrous toluene (7.50 mL) was heated at 110 °C for 20 h. The reaction mixture was cooled to room temperature before being filtered through silica and rinsed with Et_2O. The filtrate was concentrated *in vacuo*. The residue was purified by FCC (Hexane/Et_2O = 90:10) to afford the title compound (471 mg, 90%) as a colorless solid. m.p. 125 - 127 °C (Et_2O/hexane); IR (thin film) ν_{max}/cm^{-1}: 2991, 2966, 1917, 1452, 1377, 1214, 978; ^{1}H NMR (500 MHz, $CDCl_3$) δ_H = 7.91 – 7.83 (m, 1H, ArC<u>H</u>), 7.83 – 7.80 (m, 1H, ArC<u>H</u>), 7.79 (d, *J* = 8.4 Hz, 1H, ArC<u>H</u>), 7.45 – 7.27 (m, 2H, ArC<u>H</u>), 2.22 (d, *J* = 13.4 Hz, 1H, C**3**-<u>H</u>), 2.10 – 1.94 (m, 2H, C**3'**-<u>H</u>$_2$), 1.58 (dd, *J* = 13.4, 4.3 Hz, 1H, C**3**-<u>H</u>'), 1.51 (s, 3H, C**5'**-<u>H</u>$_3$), 1.49 (d, *J* = 13.0 Hz, 3H, C**1'**-<u>H</u>$_3$), 1.44 (s, 3H, C**5**-<u>H</u>$_3$), 1.33 (d, *J* = 14.0 Hz, 3H, C**1**-<u>H</u>$_3$); ^{13}C NMR (125 MHz, $CDCl_3$) δ_C = 144.3 (Ar<u>C</u>), 139.4 (d, *J* = 10.8 Hz, Ar<u>C</u>), 136.8 (d, *J* = 32.3 Hz, Ar<u>C</u>H), 134.3 (d, *J* = 48.6 Hz, Ar<u>C</u>), 125.4 (Ar<u>C</u>), 124.4 (Ar<u>C</u>H), 123.8 (Ar<u>C</u>H), 122.2 (Ar<u>C</u>H), 97.0 (C**4**), 96.5 (C**4'**), 73.4 (d, *J* = 36.1 Hz, C**2**), 73.3 (d, *J* = 46.6 Hz, C**2'**), 44.7 (d, *J* = 17.5 Hz, C**3'**), 37.5 (d, *J* = 2.2 Hz, C**3**), 28.1 (C**5**), 27.9 (C**5'**), 27.8 (d, *J* = 23.6 Hz, C**1**), 27.3 (d, *J* = 11.2 Hz, C**1'**); ^{31}P NMR (202 MHz, $CDCl_3$) δ_P = -31.5; HRMS (ESI$^+$) calculated for $C_{18}H_{22}O_3PS$ $[M+H]^+$ = 349.1021, found 349.1022.

Optimization of the Aza-Heck Cascade

Ligands for optimization experiments

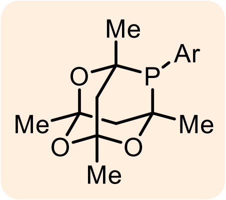

L1: Ar = Ph
L2: Ar = 2-furyl
L3: Ar = 2-benzofuryl
L4: Ar = 2-benzothienyl
L5: Ar = 4-OMePh
L6: Ar = 3,5-$(CF_3)_2$Ph
L7: Ar = 4-CO_2EtPh
L8: Ar = 4-NO_2Ph
L9: Ar = 3,5-F_2-4-NO_2Ph
L10: Ar = 2-thienyl
L11: Ar = 5-Ac-2-thienyl
L12: Ar = 2-pyridyl
L13: Ar = 5-CO_2Me-2-furyl
L14: Ar = 5-Ac-2-furyl
L15: Ar = 5-Et-2-furyl

P[3,5-$(CF_3)_2C_6H_3]_3$
P[2,4,6-$(MeO)_3C_6H_2]_3$
P(2-furyl)$_3$
dppp
CyPAd-Dalphos
PAd_2-Dalphos

Selected additional optimization experiments for Scheme 2B are presented below:

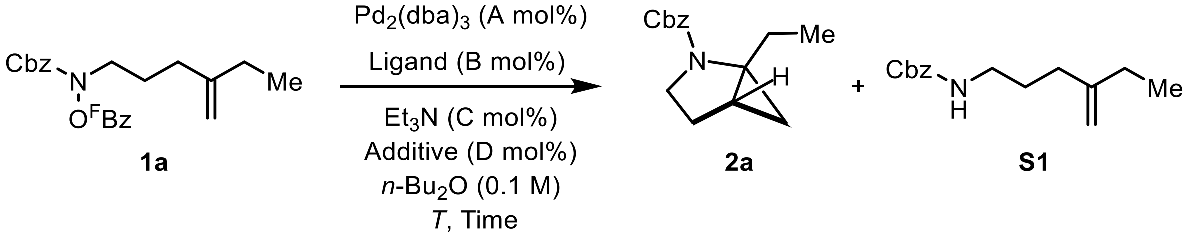

Entry	Pd (A mol%)	Ligand (B mol%)	Et_3N (C mol%)	Additive (D mol%)	*T* *(ºC)*	*Time (h)*	NMR Yield (isolated yield)% for 2a
**1**	5	**L1** (20)	100	none	140	24	13
**2**	5	**L1** (20)	100	NaOBz (100)	140	24	22
**3**	5	**L1** (20)	100	NaOAc (100)	140	24	34
**4**	5	**L1** (20)	100	$AdCO_2Na$ (100)	140	24	30
**5**	5	**L1** (20)	100	CsOPiv (100)	140	24	56
**6**	5	P[3,5-$(CF3)_2C_6H_3]_3$ (20)	100	CsOPiv (100)	140	24	n.d.[a]
**7**	5	P[2,4,6-$(MeO)_3C_6H_2]_3$ (20)	100	CsOPiv (100)	140	24	n.d.[a]
**8**	5	P(2-furyl)$_3$ (20)	100	CsOPiv (100)	140	24	n.d.[a]
**9**	5	dppp (20)	100	CsOPiv (100)	140	24	n.d.[a]
**10**	5	CyPAd-Dalphos (20)	100	CsOPiv (100)	140	24	n.d.[a]
**11**	5	PAd_2-Dalphos (20)	100	CsOPiv (100)	140	24	n.d.[a]
**12**	5	**L5** (20)	100	CsOPiv (100)	140	24	62
**13**	5	**L6** (20)	100	CsOPiv (100)	140	24	52

**14**	5	**L7** (20)	100	CsOPiv (100)	140	24	55
**15**	5	**L8** (20)	100	CsOPiv (100)	140	24	59
**16**	5	**L9** (20)	100	CsOPiv (100)	140	24	47
**17**	5	**L10** (20)	100	CsOPiv (100)	140	24	59
**18**	5	**L11** (20)	100	CsOPiv (100)	140	24	61
**19**	5	**L12** (20)	100	CsOPiv (100)	140	6	50
**20**	5	**L2** (20)	100	CsOPiv (100)	140	8	68 (65)
**21**	5	**L13** (20)	100	CsOPiv (100)	140	8	57
**22**	5	**L2**(25)	100	CsOPiv (100)	140	6	70 (67)
**23**	5	**L14** (25)	100	CsOPiv (100)	140	6	72
**24**	5	**L15** (25)	100	CsOPiv (100)	140	6	67
**25**	5	**L3** (25)	100	CsOPiv (100)	140	6	83 (80)
**26**	5	**L4** (25)	100	CsOPiv (100)	140	6	58
**27**	5	**L3** (20)	100	CsOPiv (100)	140	6	65
**28**	5	**L3** (30)	100	CsOPiv (100)	140	6	82
**29**	5	**L3** (25)	200	CsOPiv (200)	140	6	77
**30**	5	**L3** (25)	50	CsOPiv (50)	140	6	47
**31**	5	**L3** (25)	100	CsOPiv (50)	140	6	59
**32**	5	**L3** (25)	100	CsOPiv (200)	140	6	69
**33**	5	**L3** (25)	100	CsOPiv (100)	150	6	71
**34**	5	**L3** (25)	100	CsOPiv (100)	130	8	64
**35**	2.5	**L3**(12.5)	50	CsOPiv (50)	140	6	63
**36**	3.75	**L3** (18.75)	75	CsOPiv (75)	140	6	67
**37**	3.75	**L3** (18.75)	100	CsOPiv (100)	140	6	69
**38**	5	**L3** (25)	no	CsOPiv (100)	140	6	67 (68)
**39**	5	**L3** (25)	no	CsOPiv (100)/collidine (100)	140	6	73
**40**	5	**L3** (25)	no	CsOPiv (100)/Cs_2CO_3 (100)	140	6	64

a **S1** was detected after the reaction was completed.

Selected optimization experiments for Table 1D (1k to *iso*-2k) are presented below:

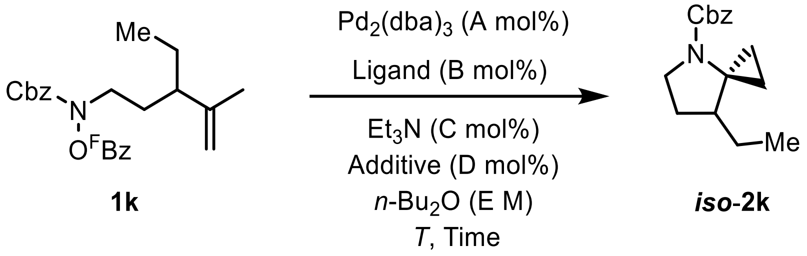

Entry	Pd (A mol%)	Ligand (B mol%)	Base (C mol%)	Additive (D mol%)	*n*-Bu$_2$O (E M)	*T* (ºC)	*Time* (h)	NMR Yield (isolated yield)%
**1**	5	**L1** (30)	Et$_3$N (100)	NaOAc (100)	*n*-Bu$_2$O (0.1)	150	24	14
**2**	5	**L1** (30)	Et$_3$N (100)	NaOBz (100)	*n*-Bu$_2$O (0.1)	150	24	0
**3**	5	**L1** (30)	Et$_3$N (100)	NaOAc (100)	*n*-Bu$_2$O (0.1)	160	24	18
**4**	5	**L1** (30)	Et$_3$N (100)	KOAc (100)	*n*-Bu$_2$O (0.1)	150	24	39
**5**	5	**L3** (25)	Et$_3$N (100)	CsOPiv (100)	*n*-Bu$_2$O (0.1)	150	6	39
**6**	5	**L5** (30)	Et$_3$N (100)	KOAc (100)	*n*-Bu$_2$O (0.1)	150	24	12
**7**	5	**L6** (30)	Et$_3$N (100)	KOAc (100)	*n*-Bu$_2$O (0.1)	150	24	15
**8**	5	**L7** (30)	Et$_3$N (100)	KOAc (100)	*n*-Bu$_2$O (0.1)	150	24	21
**9**	5	**L11** (30)	Et$_3$N (100)	KOAc (100)	*n*-Bu$_2$O (0.1)	150	24	18
**10**	5	**L3** (30)	Et$_3$N (100)	KOAc (100)	*n*-Bu$_2$O (0.1)	150	24	47
**11**	5	**L3** (30)	Et$_3$N (100)	CsOAc (100)	*n*-Bu$_2$O (0.1)	150	24	30
**12**	5	**L3** (30)	Et$_3$N (100)	LiOAc (100)	*n*-Bu$_2$O (0.1)	150	24	13
**13**	5	**L3** (30)	Et$_3$N (100)	CsOPiv (100)	*n*-Bu$_2$O (0.1)	150	24	37

**14**	5	**L3** (30)	Et_3N (100)	KOAc (100)	*n*-Bu_2O (0.1)	160	24	61
**15**	5	**L3** (30)	Et_3N (100)	KOAc (100)	*n*-Bu_2O (0.1)	170	24	56
**16**	5	**L3** (30)	Et_3N (50)	KOAc (100)	*n*-Bu_2O (0.1)	160	24	56
**17**	5	**L3** (30)	Et_3N (200)	KOAc (100)	*n*-Bu_2O (0.1)	160	24	53
**18**	5	**L3** (30)	Et_3N (100)	KOAc (200)	*n*-Bu_2O (0.1)	160	24	69
**19**	5	**L3** (30)	Et_3N (100)	KOAc (50)	*n*-Bu_2O (0.1)	160	24	56
**20**	5	**L3** (30)	Et_3N (100)	KOAc (100)	*n*-Bu_2O (0.2)	160	24	53
**21**	5	**L3** (30)	Et_3N (100)	KOAc (100)	*n*-Bu_2O (0.05)	160	24	65
**22**	5	**L3** (30)	Et_3N (100)	KOAc (200)	*n*-Bu_2O (0.05)	160	24	74 (73)
**23**	5	**L3** (30)	Et_3N (100)	KOAc (300)	*n*-Bu_2O (0.05)	160	24	56
**24**	5	**L3** (25)	Et_3N (100)	KOAc (200)	*n*-Bu_2O (0.05)	160	24	68
**25**	2.5	**L3** (15)	Et_3N (100)	KOAc (200)	*n*-Bu_2O (0.05)	160	24	30

Selected optimization experiments for Table 3B (3a to 4a) are presented below:

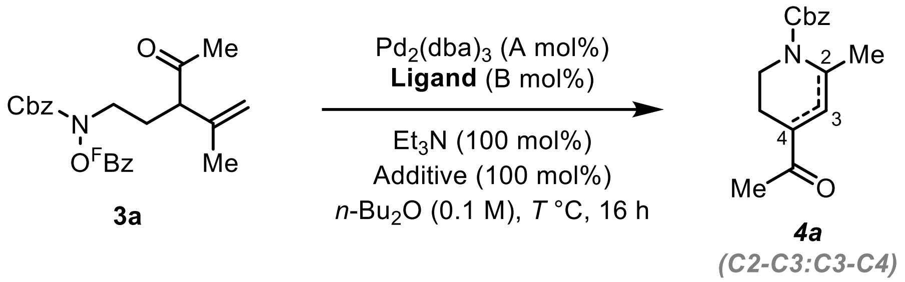

Entry	Pd (A mol%)	Ligand (B mol%)	Additive (100 mol%)	*T* (ºC)	NMR Yield (isolated yield)% [*C2-C3:C3-C4*]
**1**	5	**L1** (30)	NaOAc	110	33% [1.8:1]
**2**	5	**L1** (30)	KOAc	110	29% [2:1]
**3**	5	**L1** (30)	CsOAc	110	57% [7.1:1]
**4**	5	**L1** (30)	CsOPiv	110	46% [1:2.5]
**5**	5	**L5** (30)	CsOAc	110	64% [5:1]
**6**	5	**L6** (30)	CsOAc	110	60% [5:1]
**7**	5	**L10** (30)	CsOAc	110	71% [2.6:1]
**8**	5	**L2** (30)	CsOAc	110	70% [4.4:1]
**9**	5	**L3** (30)	CsOAc	110	(80% [5.4:1])
**10**	5	**L4** (30)	CsOAc	110	58% [4.3:1]
**11**	5	**L3** (30)	CsOAc	100	(71% [6.0:1])
**12**	2.5	**L3** (15)	CsOAc	110	(81% [6.4:1])

Substrate Synthesis and Catalysis

4-Methylenehexanoic acid

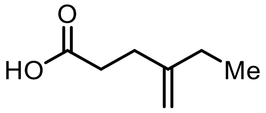

General procedure D: 2-Methylenebutan-1-ol (1.72 g, 20.0 mmol) was employed with propionic acid (148 mg, 149 µL, 2.00 mmol) and triethyl orthoacetate (16.2 g, 18.3 mL, 100 mmol) for 5 h at 140 °C; KOH (4.0 M, 50.0 mL, 200 mmol) was used. Purification by flash column chromatography (Hexane/EtOAc = 85/15 to 75/25) afforded the title compound (1.85 g, 72%) as a colorless oil. ^{1}H NMR (500 MHz, $CDCl_3$) δ_H = 11.25 (br s, 1H), 4.77 (d, J = 1.2 Hz, 1H), 4.72 (d, J = 1.2 Hz, 1H), 2.56 – 2.47 (m, 2H), 2.36 (t, J = 7.7 Hz, 2H), 2.04 (q, J = 7.4 Hz, 2H), 1.04 (t, J = 7.4 Hz, 3H).

The spectroscopic properties were consistent with the data available in the literature.[5]

4-Methylenehexan-1-ol

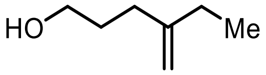

General procedure E: The preceding carboxylic acid (1.79 g, 14.0 mmol) was employed with $LiAlH_4$ (1.06 g, 28.0 mmol) in THF at r.t. for 1 h. Purification by flash column chromatography (Hexane/EtOAc = 90/10 to 85/15) afforded the title compound (1.16 g, 73%) as a colorless oil. ^{1}H NMR (500 MHz, C_6D_6) δ_H = 4.84 – 4.75 (m, 2H), 3.39 (t, J = 6.5 Hz, 2H), 2.04 – 1.97 (m, 2H), 1.92 (q, J = 7.4 Hz, 2H), 1.61 – 1.52 (m, 2H), 1.49 (br s, 1H), 0.96 (t, J = 7.4 Hz, 3H); ^{13}C NMR (125 MHz, C_6D_6) δ_C = 151.1, 108.2, 62.4, 32.8, 31.3, 29.1, 12.6.

The spectroscopic properties were consistent with the data available in the literature.[6]

Benzyl (4-methylenehexyl)(tosyloxy)carbamate (1a')

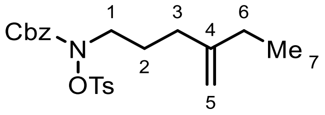

General procedure N: The preceding alcohol (125 mg, 1.09 mmol) was employed with CbzNHOTs (421 mg, 1.31 mmol), triphenylphosphine (344 mg, 1.31 mmol) and diisopropyl azodicarboxylate (265 mg, 260 µL, 1.31 mmol). Purification by flash column chromatography (Hexane/EtOAc = 100/0 to 92.5/7.5) afforded the title compound (403 mg, 89%) as a colorless oil. IR (thin film) ν_{max}/cm^{-1}: 2965, 1756, 1725, 1383, 1191, 751; ^{1}H NMR (400 MHz, $CDCl_3$) δ_H = 7.98 – 7.73 (m, 2H, Ts ArCH), 7.46 – 7.31 (m, 3H, 2 × Ts ArCH + Cbz ArCH), 7.22 – 7.08 (m, 4H, Cbz ArCH), 4.91 (s, 2H, Cbz CH_2), 4.77 – 4.70 (m, 1H, C**5**-H), 4.69 – 4.64 (m, 1H, C**5**-H'), 3.74 – 3.49 (m, 2H, C**1**-H_2), 2.40 (s, 3H, Ts CH_3), 2.03 – 1.89 (m, 4H, C**3**-H_2 + C**6**-H_2), 1.83 – 1.71 (m, 2H, C**2**-H_2), 1.00 (t, J = 7.4 Hz, 3H, C**7**-H_3); ^{13}C NMR (101 MHz, $CDCl_3$) δ_C = 156.7 (C=O), 149.9 (C**4**), 145.8 (Ts ArC), 135.1 (Cbz ArC), 131.1 (Ts

ArC), 129.6 (Cbz ArCH), 129.6 (Ts ArCH), 128.6 (Cbz ArCH + Ts ArCH), 128.3 (Cbz ArCH), 108.5 (C**5**), 68.8 (Cbz CH_2), 53.1 (C**1**), 33.1 (C**3**), 28.7 (C**6**), 24.0 (C**2**), 21.9 (Ts CH_3), 12.4 (C**7**); HRMS (ESI^+) calculated for $C_{22}H_{28}NO_5S$ $[M+H]^+$ = 418.1683, found 418.1696.

Benzyl (4-methylenehexyl)(pivaloyloxy)carbamate (1a'')

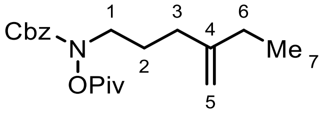

General procedure N: The preceding alcohol (125 mg, 1.09 mmol) was employed with CbzNHOPiv (329 mg, 1.31 mmol), triphenylphosphine (344 mg, 1.31 mmol) and diisopropyl azodicarboxylate (265 mg, 260 µL, 1.31 mmol). Purification by flash column chromatography (Hexane/EtOAc = 95/5 to 90/10) afforded the title compound (355 mg, 94%) as a colorless oil. IR (thin film) ν_{max}/cm^{-1}: 2971, 1780, 1721, 1405, 1077; ^1H NMR (400 MHz, $CDCl_3$) δ_H = 7.38 – 7.29 (m, 5H, ArCH), 5.15 (s, 2H, Cbz CH_2), 4.77 – 4.72 (m, 1H, C**5**-H), 4.71 – 4.67 (m, 1H, C**5**-H'), 3.64 (t, J = 7.2 Hz, 2H, C**1**-H_2), 2.08 (t, J = 7.6 Hz, 2H, C**3**-H_2), 2.00 (q, J = 7.4 Hz, 2H, C**6**-H_2), 1.76 – 1.68 (m, 2H, C**2**-H_2), 1.23 (s, 9H, Piv $C(CH_3)_3$), 1.01 (t, J = 7.4 Hz, 3H, C**7**-H_3); ^{13}C NMR (101 MHz, $CDCl_3$) δ_C = 175.9 (Piv C=O), 155.6 (Cbz C=O), 150.3 (C**4**), 135.8 (Cbz ArC), 128.6 (Cbz ArCH), 128.4 (Cbz ArCH), 128.2 (Cbz ArCH), 108.3 (C**5**), 68.3 (Cbz CH_2), 50.3 (C**1**), 38.4 (Piv $C(CH_3)_3$), 33.1 (C**3**), 28.9 (C**6**), 27.1 (Piv $C(CH_3)_3$), 25.3 (C**2**), 12.5 (C**7**); HRMS (ESI^+) calculated for $C_{20}H_{29}NNaO_4$ $[M+Na]^+$ = 370.1989, found 370.1992.

Benzyl (4-methylenehexyl)((perfluorobenzoyl)oxy)carbamate (1a)

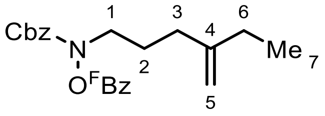

General procedure N: The preceding alcohol (685 mg, 6.00 mmol) was employed with $CbzNHO^FBz$ (2.60 g, 7.20 mmol), triphenylphosphine (1.89 g, 7.20 mmol) and diisopropyl azodicarboxylate (1.33 g, 1.31 mL, 6.60 mmol). Purification by flash column chromatography (Hexane/EtOAc = 100/0 to 97/3) afforded the title compound (1.98 g, 72%) as a colorless oil. IR (thin film) ν_{max}/cm^{-1}: 2967, 1786, 1730, 1524, 1500, 1172; ^1H NMR (500 MHz, $CDCl_3$) δ_H = 7.46 – 7.27 (m, 5H, Cbz ArCH), 5.22 (s, 2H, Cbz CH_2), 4.77 – 4.73 (m, 1H, C**5**-H), 4.72 – 4.68 (m, 1H, C**5**-H'), 3.75 (t, J = 7.2 Hz, 2H, C**1**-H_2), 2.10 (t, J = 7.6 Hz, 2H, C**3**-H_2), 2.00 (q, J = 7.4 Hz, 2H, C**6**-H_2), 1.85 – 1.75 (m, 2H, C**2**-H_2), 1.01 (t, J = 7.4 Hz, 3H, C**7**-H_3); ^{19}F NMR (377 MHz, $CDCl_3$) δ_F = -136.0 – -136.2 (m, 2F), -146.1 (tt, J = 21.0, 5.5 Hz, 1F), -159.2 – -159.4 (m, 2F); ^{13}C NMR (125 MHz, $CDCl_3$) δ_C = 155.5 (C=O), 150.0 (C**4**), 135.3 (Cbz ArC), 128.7 (Cbz ArCH), 128.6 (Cbz ArCH), 128.3 (Cbz ArCH), 108.5 (C**5**), 68.9 (Cbz CH_2), 51.1 (C**1**), 32.9 (C**3**), 28.8 (C**6**), 25.1 (C**2**), 12.4 (C**7**); HRMS (ESI^+) calculated for $C_{22}H_{20}F_5NNaO_4$ $[M+Na]^+$ = 480.1205, found 480.1202.

The carbon signals corresponding to the pentafluorobenzoyl group could not be resolved due to their weak intensity.

Benzyl 1-ethyl-2-azabicyclo[3.1.0]hexane-2-carboxylate (2a)

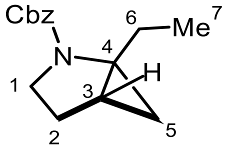

General procedure O: The preceding substrate (45.7 mg, 0.10 mmol) was employed with $Pd_2(dba)_3$ (4.58 mg, 0.005 mmol), CgP(2-benzofuryl) (**L3**) (8.31 mg, 0.025 mmol), CsOPiv (23.4 mg, 0.10 mmol), Et_3N (14.0 µL, 0.10 mmol) and dibutyl ether (1.0 mL). The reaction was stirred at 140 °C for 6 h. Purification by flash column chromatography (Hexane/EtOAc = 100/0 to 95/5) afforded the title compound (19.8 mg, 81%) as a colorless oil. IR (thin film) ν_{max}/cm^{-1}: 2967, 2923, 2868, 1703, 1407, 1340, 1054, 1013; ^{1}H NMR (500 MHz, DMSO-d^6, 100 °C) δ_H = 7.60 – 7.19 (m, 5H, Cbz ArC<u>H</u>), 5.10 (d, J = 12.7 Hz, 1H, Cbz C<u>H</u>), 5.07 (d, J = 12.7 Hz, 1H, Cbz C<u>H</u>'), 3.63 (ddd, J = 11.3, 9.6, 5.7 Hz, 1H, C**1**-<u>H</u>), 3.40 (ddd, J = 11.3, 8.7, 6.0 Hz, 1H, C**1**-<u>H</u>'), 2.43 (dq, J = 14.5, 7.3 Hz, 1H, C**6**-<u>H</u>), 2.17 – 2.03 (m, 1H, C**2**-<u>H</u>), 1.84 – 1.72 (m, 1H, C**2**-<u>H</u>'), 1.43 – 1.35 (m, 1H, C**3**-<u>H</u>), 1.28 (dq, J = 14.5, 7.3 Hz, 1H, C**6**-<u>H</u>'), 0.90 (1H, dd, J = 8.7, 5.1 Hz, C**5**-<u>H</u>), 0.86 (dd, J = 7.3, 7.3 Hz, 3H, C**7**-<u>H$_3$</u>), 0.66 (dd, J = 5.1, 5.1 Hz, 1H, C**5**-<u>H</u>'); ^{13}C NMR (125 MHz, DMSO-d^6, 100 °C) δ_C = 154.9 (<u>C</u>=O), 136.8 (Cbz Ar<u>C</u>),127.8 (Cbz Ar<u>C</u>H), 127.1 (Cbz Ar<u>C</u>H), 126.8 (Cbz Ar<u>C</u>H), 65.3 (Cbz <u>C</u>H$_2$), 49.5 (C**1**), 47.9 (C**4**), 25.3 (C**2**), 24.8 (C**6**), 22.3 (C**3**), 21.3 (C**5**), 9.6 (C**7**); HRMS (ESI$^+$) calculated for $C_{15}H_{20}NO_2$ [M+H]$^+$ = 246.1489, found 246.1491.

3-Methyl-2-methylenebutanal

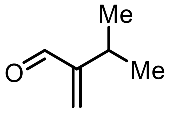

General procedure B: 3-Methylbutanal (3.45 g, 4.29 mL, 40.0 mmol) was employed with methylamine hydrochloride (3.24 g, 40.0 mmol) and formaldehyde (3.26 g, 40.0 mmol). Purification by vacuum distillation afforded the title compound (2.87 g, 73%) as a colorless oil. ^{1}H NMR (500 MHz, $CDCl_3$) δ_H = 9.53 (s, 1H), 6.23 (d, J = 1.0 Hz, 1H), 5.94 (s, 1H), 2.79 (heptd, J = 6.8, 1.0 Hz, 1H), 1.07 (d, J = 6.8 Hz, 6H); ^{13}C NMR (125 MHz, $CDCl_3$) δ_C = 194.8, 156.6, 132.3, 26.3, 21.5.

The spectroscopic properties were consistent with the data available in the literature.[7]

3-Methyl-2-methylenebutan-1-ol

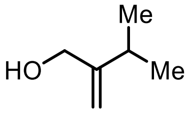

General procedure C: The preceding aldehyde (2.74 g, 28.0 mmol) was employed with $NaBH_4$ (3.13 g, 28.0 mmol). Purification by vacuum distillation afforded the title compound (2.28 g, 81%) as a colorless oil. ^{1}H NMR (500 MHz, $CDCl_3$) δ_H = 5.03 – 4.94 (m, 1H), 4.93 – 4.85 (m, 1H), 4.11 (s, 2H), 2.31 (hept, J = 6.8 Hz, 1H), 1.75 (br s, 1H), 1.06 (d, J = 6.8 Hz, 6H); ^{13}C NMR (125 MHz, $CDCl_3$) δ_C = 155.4, 107.1, 65.0, 31.1, 21.9.

The spectroscopic properties were consistent with the data available in the literature [8]

5-Methyl-4-methylenehexanoic acid

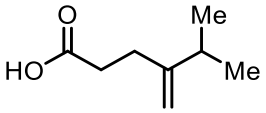

General procedure D: The preceding alcohol (2.20 g, 22.0 mmol) was employed with propionic acid (163 mg, 164 µL, 2.20 mmol) and triethyl orthoacetate (17.9 g, 20.2 mL, 110 mmol) for 5 h at 140 °C; KOH (4.0 M, 55.0 mL, 220 mmol) was used. Purification by flash column chromatography (Hexane/EtOAc = 90/10 to 85/15) afforded the title compound (1.71 g, 55%) as a colorless oil. ^{1}H NMR (500 MHz, $CDCl_3$) δ_H = 10.01 (br s, 1H), 4.83 – 4.79 (m, 1H), 4.72 – 4.63 (m, 1H), 2.59– 2.50 (m, 2H), 2.42 – 2.34 (m, 2H), 2.26 (hept, J = 6.8 Hz, 1H), 1.04 (d, J = 6.8 Hz, 6H); ^{13}C NMR (125 MHz, $CDCl_3$) δ_C = 179.8, 154.0, 107.0, 77.4, 77.2, 76.9, 34.3, 32.8, 28.7, 21.9.

The spectroscopic properties were consistent with the data available in the literature.[9]

5-Methyl-4-methylenehexan-1-ol

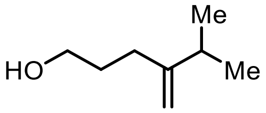

General procedure E: The preceding carboxylic acid (1.42 g, 10.0 mmol) was employed with $LiAlH_4$ (760 mg, 20.0 mmol) in THF at r.t. for 1 h. Purification by flash column chromatography (Hexane/EtOAc = 90/10 to 85/15) afforded the title compound (917 mg, 72%) as a colorless oil. IR (thin film) ν_{max}/cm^{-1}: 3315, 2960, 2935, 1542, 1378, 1057, 888; ^{1}H NMR (400 MHz, C_6D_6) δ_H = 4.84 (s, 1H), 4.76 (d, J = 1.5 Hz, 1H), 3.50 – 3.29 (m, 2H), 2.14 (hept, J = 6.8 Hz, 1H), 2.08 – 1.91 (m, 2H), 1.63 – 1.48 (m, 2H), 1.36 (br s, 1H), 0.99 (d, J = 6.8 Hz, 6H); ^{13}C NMR (101 MHz, C_6D_6) δ_C = 155.6, 106.8, 62.5, 34.2, 31.6, 30.9, 22.0; HRMS (ESI$^+$) calculated for $C_{16}H_{36}NO_2$ $[2M+NH_4]^+$ = 274.2741, found 274.2744.

Benzyl (5-methyl-4-methylenehexyl)((perfluorobenzoyl)oxy)carbamate (1b)

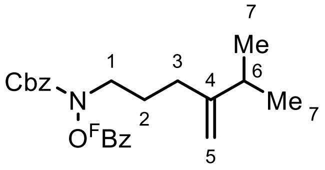

<u>General procedure N</u>: The preceding alcohol (256 mg, 2.00 mmol) was employed with CbzNHO^{F}Bz (795 mg, 2.20 mmol), triphenylphosphine (629 mg, 2.40 mmol) and diisopropyl azodicarboxylate (404 mg, 396 µL, 2.00 mmol). Purification by flash column chromatography (Hexane/EtOAc = 100/0 to 97/3) afforded the title compound (775 mg, 82%) as a colorless oil. IR (thin film) ν_{max}/cm^{-1}: 2967, 2876, 1784, 1727, 1552, 1172, 997; ^{1}H NMR (400 MHz, $CDCl_3$) δ_H = 7.52 – 7.27 (m, 5H, Cbz ArC<u>H</u>), 5.23 (s, 2H, Cbz C<u>H</u>$_2$), 4.78 (s, 1H, C**5**-<u>H</u>), 4.68 (s, 1H, C**5**-<u>H</u>’), 3.77 (t, *J* = 7.0 Hz, 2H, C**1**-<u>H</u>$_2$), 2.21 (hept, *J* = 6.8 Hz, 1H, C**6**-<u>H</u>), 2.11 (t, *J* = 7.6 Hz, 2H, C**3**-<u>H</u>$_2$), 1.87 – 1.75 (m, 2H, C**2**-<u>H</u>$_2$), 1.01 (d, *J* = 6.8 Hz, 6H, C**7**-<u>H</u>$_6$); ^{19}F NMR (377 MHz, $CDCl_3$) δ_F = -134.8 – -137.9 (m, 2F), -143.5 – -148.3 (m, 1F), -157.1 – -162.6 (m, 2F); ^{13}C NMR (101 MHz, $CDCl_3$) δ_C = 155.5 (<u>C</u>=O), 154.5 (C**4**), 135.3 (Cbz Ar<u>C</u>), 128.7 (Cbz Ar<u>C</u>H), 128.6 (Cbz Ar<u>C</u>H), 128.3 (Cbz Ar<u>C</u>H), 107.1 (C**5**), 68.9 (Cbz <u>C</u>H$_2$), 51.2 (C**1**), 33.9 (C**6**), 31.1 (C**3**), 25.4 (C**2**), 21.9 (C**7**); HRMS (ESI$^+$) calculated for $C_{23}H_{22}F_5NNaO_4$ [M+Na]$^+$ = 494.1361, found 494.1365.

The carbon signals corresponding to the pentafluorobenzoyl group could not be resolved due to their weak intensity.

Benzyl 1-isopropyl-2-azabicyclo[3.1.0]hexane-2-carboxylate (2b)

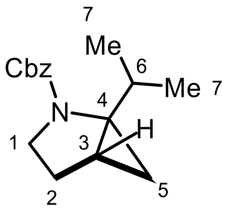

<u>General procedure O</u>: The preceding substrate (47.1 mg, 0.10 mmol) was employed with $Pd_2(dba)_3$ (4.58 mg, 0.005 mmol), CgP(2-benzofuryl) (**L3**) (16.6 mg, 0.05 mmol), CsOPiv (23.4 mg, 0.10 mmol), Et_3N (14.0 µL, 0.10 mmol) and dibutyl ether (1.0 mL). The reaction was stirred at 140 °C for 6 h. Purification by flash column chromatography (Hexane/EtOAc = 100/0 to 95/5) afforded the title compound (17.2 mg, 66%) as a colorless oil. IR (thin film) ν_{max}/cm^{-1}: 2960, 2920, 1704, 1456, 1358, 1142, 1079; ^{1}H NMR (400 MHz, DMSO-*d*^{6}, 100 °C) δ_H = 7.57 – 7.05 (m, 5H, Cbz ArC<u>H</u>), 5.09 (s, 2H, Cbz C<u>H</u>$_2$), 3.69 – 3.45 (m, 2H, C**1**-<u>H</u>$_2$), 2.62 (hept, *J* = 6.8 Hz, 1H, C**6**-<u>H</u>), 2.15 – 2.01 (m, 1H, C**2**-<u>H</u>), 1.80 – 1.74 (m, 1H, C**2**-<u>H</u>’), 1.52 – 1.39 (m, 1H, C**3**-<u>H</u>), 1.11 (dd, *J* = 8.8, 5.4 Hz, 1H, C**5**-<u>H</u>), 0.94 (d, *J* = 6.8 Hz, 3H, C**7**-<u>H</u>$_3$), 0.68 (d, *J* = 6.8 Hz, 3H, C**7**-<u>H</u>$_3$’), 0.56 (dd, *J* = 5.4, 5.4 Hz, 1H, C**5**-<u>H</u>’); ^{13}C NMR (101 MHz, DMSO-*d*^{6}, 100 °C) δ_C = 154.9 (<u>C</u>=O), 136.8 (Cbz Ar<u>C</u>),127.6 (Cbz Ar<u>C</u>H), 126.9 (Cbz Ar<u>C</u>H), 126.7 (Cbz Ar<u>C</u>H), 65.2 (Cbz <u>C</u>H$_2$), 52.1 (C**1**), 50.4 (C**4**), 26.6 (C**6**), 25.7 (C**2**), 20.7 (C**5**), 19.2 (C**3**), 19.0 (C**7**), 17.5 (C**7’**); HRMS (ESI$^+$) calculated for $C_{16}H_{21}NNaO_2$ [M+Na]$^+$ = 282.1464, found 282.1463.

2-Methyleneoctanal

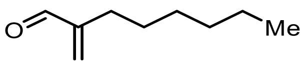

General procedure B: Octanal (2.56 g, 3.12 mL, 20.0 mmol) was employed with methylamine hydrochloride (1.52 g, 18.6 mmol) and formaldehyde (1.62 g, 20.0 mmol). Purification by vacuum distillation afforded the title compound (1.68 g, 60%) as a colorless oil. ^{1}H NMR (500 MHz, $CDCl_3$) δ_H = 9.53 (s, 1H), 6.33 – 6.11 (m, 1H), 5.97 (s, 1H), 2.35 – 2.16 (m, 2H), 1.49 – 1.39 (m, 2H), 1.34 – 1.23 (m, 6H), 0.86 (t, J = 6.9 Hz, 3H); ^{13}C NMR (125 MHz, $CDCl_3$) δ_C = 195.0, 150.6, 134.0, 31.7, 29.1, 27.9, 27.8, 22.7, 14.2.

The spectroscopic properties were consistent with the data available in the literature. [10]

2-Methyleneoctan-1-ol

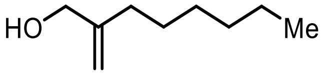

General procedure C: The preceding aldehyde (1.05 g, 7.50 mmol) was employed with $NaBH_4$ (284 mg, 7.50 mmol). The title compound (1.06 g, 99%) was afforded as a colorless oil without further purification. ^{1}H NMR (500 MHz, $CDCl_3$) δ_H = 5.01 (d, J = 1.5 Hz, 1H), 4.86 (d, J = 1.5 Hz, 1H), 4.07 (s, 2H), 2.05 (t, J = 7.8 Hz, 2H), 1.50 (br s, 1H), 1.46 – 1.42 (m, 2H), 1.31 – 1.27 (m, 6H), 0.89 (t, J = 7.0 Hz, 3H); ^{13}C NMR (125 MHz, $CDCl_3$) δ_C = 149.4, 109.1, 66.1, 33.2, 31.9, 29.2, 27.9, 22.8, 14.2.

The spectroscopic properties were consistent with the data available in the literature.[11]

4-Methylenedecanoic acid

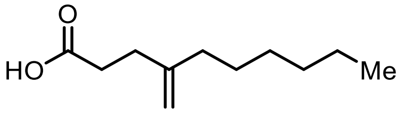

General procedure D: The preceding alcohol (1.05 g, 7.40 mmol) was employed with propionic acid (54.8 mg, 55.2 µL, 0.74 mmol) and triethyl orthoacetate (6.00 g, 6.80 mL, 37.0 mmol) for 5 h at 140 °C; KOH (4.0 M, 18.5 mL, 74.0 mmol) was used. Purification by flash column chromatography (Hexane/EtOAc = 95/5 to 85/15) afforded the title compound (1.00 g, 73%) as a colorless oil. ^{1}H NMR (500 MHz, $CDCl_3$) δ_H = 4.82 – 4.75 (m, 1H), 4.74 – 4.65 (m, 1H), 2.58 – 2.42 (m, 2H), 2.34 (t, J = 7.7 Hz, 2H), 2.09 – 1.93 (m, 2H), 1.48 – 1.36 (m, 2H), 1.36 – 1.22 (m, 6H), 0.88 (t, J = 7.0 Hz, 3H); δ_C = ^{13}C NMR (126 MHz, $CDCl_3$) δ 179.6, 148.1, 109.4, 36.4, 32.6, 31.9, 30.6, 29.2, 27.8, 22.8, 14.2.

The spectroscopic properties were consistent with the data available in the literature.[12]

4-Methylenedecan-1-ol

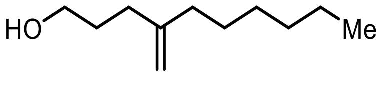

General procedure E: The preceding carboxylic acid (849 mg, 4.60 mmol) was employed with $LiAlH_4$ (349 mg, 9.20 mmol) in THF at r.t. for 1 h. Purification by flash column chromatography (Hexane/EtOAc = 90/10 to 85/15) afforded the title compound (711 mg, 91%) as a colorless oil. ^{1}H NMR (400 MHz, C_6D_6) δ_H = 4.82 (s, 2H), 3.53 – 3.26 (m, 2H), 2.13 – 1.88 (m, 4H), 1.69 – 1.50 (m,

2H), 1.49 – 1.36 (m, 2H), 1.32 – 1.21 (m, 6H), 0.90 (t, J = 6.8 Hz, 3H); ^{13}C NMR (101 MHz, C_6D_6) δ_C = 149.8, 109.2, 62.5, 36.5, 32.7, 32.2, 31.3, 29.5, 28.2, 23.1, 14.4.

The spectroscopic properties were consistent with the data available in the literature.[13]

Benzyl (4-methylenedecyl)((perfluorobenzoyl)oxy)carbamate (1c)

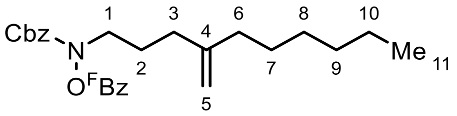

General procedure N: The preceding alcohol (341 mg, 2.00 mmol) was employed with CbzNHO^{F}Bz (795 mg, 2.20 mmol), triphenylphosphine (629 mg, 2.40 mmol) and diisopropyl azodicarboxylate (404 mg, 396 µL, 2.00 mmol). Purification by flash column chromatography (Hexane/EtOAc = 100/0 to 97/3) afforded the title compound (858 mg, 84%) as a colorless oil. IR (thin film) ν_{max}/cm^{-1}: 2931, 2858, 1787, 1552, 1327, 1172, 1004; ^{1}H NMR (400 MHz, $CDCl_3$) δ_H = 7.35 (s, 5H, Cbz ArC$\underline{H}$), 5.22 (s, 2H, Cbz C$\underline{H_2}$), 4.73 (s, 1H, C**5**-$\underline{H}$), 4.70 (s, 1H, C**5**-$\underline{H}$'), 3.75 (t, J = 7.2 Hz, 2H, C**1**-$\underline{H_2}$), 2.08 (t, J = 7.7 Hz, 2H, C**3**-$\underline{H_2}$), 1.98 (t, J = 7.7 Hz, 2H, C**6**-$\underline{H_2}$), 1.85 – 1.74 (m, 2H, C**2**-$\underline{H_2}$), 1.45 – 1.35 (m, 2H, C**7**-$\underline{H_2}$), 1.32 – 1.21 (m, 6H, C**8**-$\underline{H_2}$ + C**9**-$\underline{H_2}$ + C**10**-$\underline{H_2}$), 0.88 (t, J = 6.5 Hz, 3H, C**11**-$\underline{H_3}$); ^{19}F NMR (377 MHz, $CDCl_3$) δ_F = -135.9 – 136.2 (m, 2F), -146.1 (tt, J = 20.8, 5.5 Hz, 1F), -159.2 – -159.6 (m, 2F); ^{13}C NMR (101 MHz, $CDCl_3$) δ_C = 155.5 ($\underline{C}$=O), 148.6 (C**4**), 135.4 (Cbz Ar$\underline{C}$), 128.7 (Cbz Ar$\underline{C}$H), 128.6 (Cbz Ar$\underline{C}$H), 128.3 (Cbz Ar$\underline{C}$H), 109.6 (C**5**), 68.9 (Cbz $\underline{C}H_2$), 51.1 (C**1**), 36.1 (C**6**), 32.8 (C**3**), 31.9 (C**9**), 29.2 (C**8**), 27.8 (C**7**), 25.0 (C**2**), 22.8 (C**10**), 14.2 (C**11**); HRMS (ESI$^+$) calculated for $C_{26}H_{28}F_5NNaO_4$ $[M+Na]^+$ = 536.1830, found 536.1825.

The carbon signals corresponding to the pentafluorobenzoyl group could not be resolved due to their weak intensity.

Benzyl 1-hexyl-2-azabicyclo[3.1.0]hexane-2-carboxylate (2c)

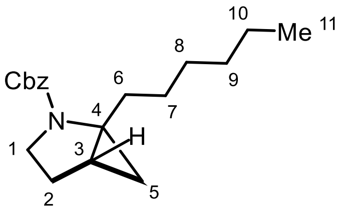

General procedure O: The preceding substrate (51.4 mg, 0.10 mmol) was employed with $Pd_2(dba)_3$ (4.58 mg, 0.005 mmol), CgP(2-benzofuryl) (**L3**) (8.31 mg, 0.025 mmol), CsOPiv (23.4 mg, 0.10 mmol), Et_3N (14.0 µL, 0.10 mmol) and dibutyl ether (1.0 mL). The reaction was stirred at 140 °C for 6 h. Purification by flash column chromatography (Hexane/EtOAc = 100/0 to 96/4 for the first column, then toluene/EtOAc = 100/0 to 99/1 for the second column) afforded the title compound (21.8 mg, 72%) as a colorless oil. IR (thin film) ν_{max}/cm^{-1}: 2954, 2925, 2858, 1704, 1408, 1077; ^{1}H NMR (400 MHz, DMSO-d^6, 100 °C) δ_H = 7.90 – 6.78 (m, 5H, Cbz ArC$\underline{H}$), 5.11 (d, J = 12.8 Hz, 1H, Cbz C$\underline{H}$), 5.07 (d, J = 12.8 Hz, 1H, Cbz C$\underline{H}$'), 3.63 (ddd, J = 10.8, 10.3, 5.8 Hz, 1H, C**1**-$\underline{H}$), 3.39 (ddd, J = 10.8, 8.8, 6.1

Hz, 1H, C**1**-H'), 2.49 – 2.40 (m, 1H, C**6**-H), 2.18 – 2.01 (m, 1H, C**2**-H), 1.84 – 1.72 (m, 1H, C**2**-H'), 1.49 – 1.36 (m, 1H, C**3**-H), 1.34 – 1.21 (m, 8H, C**7**-H_2 + C**8**-H_2 + C**9**-H_2 + C**10**-H_2), 1.20 – 1.13 (m, 1H, C**6**-H'), 0.91 – 0.81 (m, 4H, C**5**-H + C**11**-H_3), 0.63 (dd, *J* = 5.1, 5.1 Hz, 1H, C**5**-H'); ^{13}C NMR (101 MHz, DMSO-d^6, 100 °C) δ_C = 154.8 (C=O), 136.8 (Cbz ArC), 127.6 (Cbz ArCH), 127.0 (Cbz ArCH), 126.7 (Cbz ArCH), 65.2 (Cbz CH_2), 49.2 (C**1**), 46.7 (C**4**), 31.8 (C**6**), 30.6 (C**9**), 27.8 (C**8**), 25.1 (C**2**), 25.0 (C**7**), 23.0 (C**3**), 21.2 (C**5**), 21.1 (C**10**), 13.0 (C**11**); HRMS (ESI$^+$) calculated for $C_{19}H_{27}NNaO_2$ $[M+Na]^+$ = 324.1934, found 324.1936.

4-Ethoxy-2-methylenebutanal

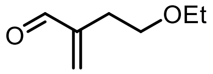

General procedure B: 4-Ethoxybutanal (1.40 g, 12.0 mmol) was employed with methylamine hydrochloride (979 mg, 12.0 mmol) and formaldehyde (974 mg, 12.0 mmol). Purification by vacuum distillation afforded the title compound (650 mg, 42%) as a colorless oil. IR (thin film) ν_{max}/cm^{-1}: 2970, 2923, 2866, 1054, 1012; ^{1}H NMR (500 MHz, $CDCl_3$) δ_H = 9.53 (s, 1H), 6.48 – 6.28 (m, 1H), 6.06 (s, 1H), 3.52 (t, *J* = 6.5 Hz, 2H), 3.46 (q, *J* = 7.0 Hz, 2H), 2.52 (t, *J* = 6.5 Hz, 2H), 1.17 (t, *J* = 7.0 Hz, 3H); ^{13}C NMR (125 MHz, $CDCl_3$) δ_C = 194.6, 147.2, 135.7, 68.3, 66.2, 28.4, 15.2; HRMS (CI$^+$) calculated for $C_7H_{13}O_2$ $[M+H]^+$ = 129.0910, found 129.0928.

4-Ethoxy-2-methylenebutan-1-ol

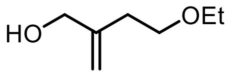

General procedure C: The preceding aldehyde (603 mg, 4.70 mmol) was employed with $NaBH_4$ (178 mg, 4.70 mmol). Purification by vacuum distillation afforded the title compound (569 mg, 93%) as a colorless oil. IR (thin film) ν_{max}/cm^{-1}: 3382, 2976, 2927, 1654, 1377, 1105, 897; ^{1}H NMR (500 MHz, $CDCl_3$) δ_H = 5.05 (d, *J* = 1.5 Hz, 1H), 4.91 (d, *J* = 1.5 Hz, 1H), 4.06 (s, 2H), 3.55 (t, *J* = 6.1 Hz, 2H), 3.51 (q, *J* = 7.0 Hz, 1H), 2.48 (br s, 1H), 2.38 (t, *J* = 6.1 Hz, 2H), 1.20 (t, *J* = 7.0 Hz, 3H); ^{13}C NMR (125 MHz, $CDCl_3$) δ_C = 147.2, 112.7, 70.4, 66.6, 66.6, 34.4, 15.2; HRMS (ESI$^+$) calculated for $C_7H_{15}O_2$ $[M+H]^+$ = 131.1066, found 131.1065.

6-Ethoxy-4-methylenehexanoic acid

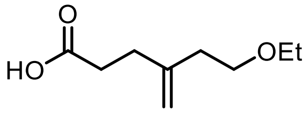

General procedure D: The preceding alcohol (503 mg, 3.80 mmol) was employed with propionic acid (28.2 mg, 28.3 µL, 0.38 mmol) and triethyl orthoacetate (3.08 g, 3.48 mL, 19.0 mmol) for 5 h at 140 °C; KOH (4.0 M, 9.50 mL, 38.0 mmol) was used. Purification by flash column chromatography (Hexane/EtOAc = 90/10 to 75/25) afforded the title compound (293 mg, 45%) as a colorless oil. IR

(thin film) ν_{max}/cm^{-1}: 2975, 2935, 1708, 1548, 1378, 1105, 891; ^{1}H NMR (400 MHz, $CDCl_3$) δ_H = 9.70 (br s, 1H), 4.82 (s, 1H), 4.80 (s, 1H), 3.53 (t, J = 7.0 Hz, 2H), 3.49 (q, J = 7.0 Hz, 2H), 2.58 – 2.46 (m, 2H), 2.36 (t, J = 7.7 Hz, 2H), 2.32 (t, J = 7.0 Hz, 2H), 1.19 (t, J = 7.0 Hz, 3H); ^{13}C NMR (101 MHz, $CDCl_3$) δ_C = 179.2, 144.9, 111.1, 69.2, 66.3, 36.4, 32.5, 31.0, 15.2; HRMS (ESI$^+$) calculated for $C_9H_{16}NaO_3$ $[M+Na]^+$ = 195.0991, found 195.0992.

6-Ethoxy-4-methylenehexan-1-ol

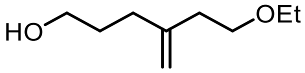

<u>General procedure E</u>: The preceding carboxylic acid (277 mg, 1.61 mmol) was employed with $LiAlH_4$ (122 mg, 3.22 mmol) in THF at r.t. for 1 h. Purification by flash column chromatography (Hexane/EtOAc = 85/15 to 75/25) afforded the title compound (198 mg, 78%) as a colorless oil. IR (thin film) ν_{max}/cm^{-1}: 3366, 2975, 2937, 1645, 1442, 1108, 890; ^{1}H NMR (500 MHz, C_6D_6) δ_H = 4.83 (s, 2H), 3.48 – 3.35 (m, 4H), 3.26 (q, J = 6.9 Hz, 2H), 2.29 (t, J = 6.9 Hz, 2H), 2.04 (t, J = 7.7 Hz, 2H), 1.65 – 1.52 (m, 2H), 1.51 (br s, 1H), 1.10 (t, J = 6.9 Hz, 3H); ^{13}C NMR (101 MHz, C_6D_6) δ_C = 147.0, 110.7, 69.7, 66.2, 62.2, 36.7, 33.0, 31.2, 15.5; HRMS (ESI$^+$) calculated for $C_9H_{19}O_2$ $[M+H]^+$ = 159.1380, found 159.1382.

Benzyl (6-ethoxy-4-methylenehexyl)((perfluorobenzoyl)oxy)carbamate (1d)

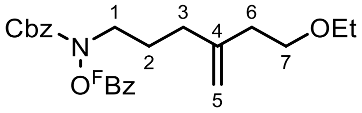

<u>General procedure N</u>: The preceding alcohol (158 mg, 1.00 mmol) was employed with CbzNHO^{F}Bz (397 mg, 1.10 mmol), triphenylphosphine (315 mg, 1.20 mmol) and diisopropyl azodicarboxylate (202 mg, 198 µL, 1.00 mmol). Purification by flash column chromatography (Hexane/EtOAc = 95/5 to 90/10) afforded the title compound (359 mg, 72%) as a colorless oil. IR (thin film) ν_{max}/cm^{-1}: 2976, 2937, 2866, 1785, 1524, 1326, 1170, 1107, 907; ^{1}H NMR (400 MHz, $CDCl_3$) δ_H = 7.50 – 7.28 (m, 5H, Cbz ArCH), 5.22 (s, 2H, Cbz C<u>H$_2$</u>), 4.91 – 4.74 (m, 2H, C**5**-<u>H$_2$</u>), 3.76 (t, J = 7.1 Hz, 2H, C**1**-<u>H$_2$</u>), 3.60 – 3.38 (m, 4H, C**7**-<u>H$_2$</u> + Et C<u>H$_2$</u>), 2.28 (t, J = 7.0 Hz, 2H, C**6**-<u>H$_2$</u>), 2.12 (t, J = 7.6 Hz, 2H, C**3**-<u>H$_2$</u>), 1.94 – 1.72 (m, 2H, C**2**-<u>H$_2$</u>), 1.19 (t, J = 7.0 Hz, 3H, Et C<u>H$_3$</u>); ^{19}F NMR (377 MHz, $CDCl_3$) δ_F = -136.0 – -136.1 (m, 2F), -146.1 (tt, J = 21.1, 5.6 Hz, 1F), -159.2 – -159.5 (m, 2F); ^{13}C NMR (101 MHz, $CDCl_3$) δ_C = 155.5 (<u>C</u>=O), 145.4 (C**4**), 135.3 (Cbz Ar<u>C</u>), 128.7 (Cbz Ar<u>C</u>H), 128.6 (Cbz Ar<u>C</u>H), 128.3 (Cbz Ar<u>C</u>H), 111.2 (C**5**), 69.3 (C**7**), 68.9 (Cbz <u>C</u>H$_2$), 66.3 (Et <u>C</u>H$_2$), 51.0 (C**1**), 36.2 (C**6**), 33.2 (C**3**), 25.0 (C**2**), 15.3 (Et <u>C</u>H$_3$); HRMS (ESI$^+$) calculated for $C_{24}H_{24}F_5NNaO_5$ $[M+Na]^+$ = 524.1467, found 524.1469.

The carbon signals corresponding to the pentafluorobenzoyl group could not be resolved due to their weak intensity.

Benzyl 1-(2-ethoxyethyl)-2-azabicyclo[3.1.0]hexane-2-carboxylate (2d)

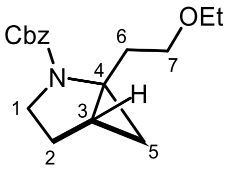

General procedure O: The preceding substrate (50.1 mg, 0.10 mmol) was employed with $Pd_2(dba)_3$ (4.58 mg, 0.005 mmol), CgP(2-benzofuryl) (**L3**) (8.31 mg, 0.025 mmol), CsOPiv (23.4 mg, 0.10 mmol), Et_3N (14.0 µL, 0.10 mmol) and dibutyl ether (1.0 mL). The reaction was stirred at 140 °C for 6 h. Purification by flash column chromatography (Hexane/EtOAc = 100/0 to 95/5) afforded the title compound (18.8 mg, 65%) as a colorless oil. IR (thin film) ν_{max}/cm^{-1}: 2931, 2868, 1703, 1410, 1331, 1112, 1081; ^{1}H NMR (400 MHz, DMSO-d^6, 100 °C) δ_H = 7.52 – 7.19 (m, 5H, Cbz ArCH), 5.11 (d, J = 13.4 Hz, 1H, Cbz CH), 5.08 (d, J = 13.4 Hz, 1H, Cbz CH'), 3.62 (ddd, J = 11.1, 9.5, 5.8 Hz, 1H, C**1**-H), 3.52 – 3.33 (m, 5H, C**1**-H' + C**7**-H_2 + Et CH_2), 2.62 (dt, J = 14.2, 6.0 Hz, 1H, C**6**-H), 2.20 – 2,03 (m, 1H, C**2**-H), 1.87 – 1.70 (m, 1H, C**2**-H'), 1.55 (dt, J = 14.2, 7.2 Hz, 1H, C**6**-H'), 1.48 (dddd, J = 8.4, 6.4, 5.2, 1.4 Hz, 1H, C**3**-H), 1.09 (t, J = 7.0 Hz, 3H, Et CH_3), 0.90 (dd, J = 8.4, 5.2 Hz, 1H, C**5**-H), 0.62 (dd, J = 5.2, 5.2 Hz, 1H, C**5**-H'); ^{13}C NMR (101 MHz, DMSO-d^6, 100 °C) δ_C = 154.9 (C=O), 136.7 (Cbz ArC), 127.7 (Cbz ArCH), 127.0 (Cbz ArCH), 126.7 (Cbz ArCH), 66.7 (C**7**), 65.3 (Cbz CH_2), 64.5 (Et CH_2), 49.1 (C**1**), 44.6 (C**4**), 31.9 (C**6**), 25.1 (C**2**), 23.1 (C**3**), 20.7 (C**5**), 14.3 (Et CH_3); HRMS (ESI$^+$) calculated for $C_{17}H_{23}NNaO_3$ $[M+Na]^+$ = 312.1570, found 312.1573.

2-Methylene-5-phenylpentanal

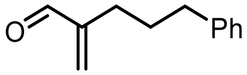

General procedure B: 5-Phenylpentanal (2.43 g, 15.0 mmol) was employed with methylamine hydrochloride (1.22 g, 15.0 mmol) and formaldehyde (1.22 g, 15.0 mmol). Purification by vacuum distillation afforded the title compound (2.15 g, 82%) as a colorless oil. ^{1}H NMR (500 MHz, $CDCl_3$) δ_H = 9.55 (s, 1H), 7.32 – 7.26 (m, 2H), 7.23 – 7.14 (m, 3H), 6.26 (d, J = 1.0 Hz, 1H), 6.07 – 5.95 (m, 1H), 2.72 – 2.58 (m, 2H), 2.38 – 2.28 (m, 2H), 1.84 – 1.76 (m, 2H); ^{13}C NMR (125 MHz, $CDCl_3$) δ_C = 194.8, 150.2, 142.0, 134.3, 128.5, 128.5, 126.0, 35.7, 29.6, 27.6.

The spectroscopic properties were consistent with the data available in the literature. [14]

2-Methylene-5-phenylpentan-1-ol

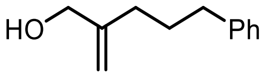

General procedure C: The preceding aldehyde (1.92 g, 11.0 mmol) was employed with $NaBH_4$ (416 mg, 11.0 mmol). The title compound (1.91 g, 99%) was afforded as a colorless oil without further purification. ^{1}H NMR (500 MHz, $CDCl_3$) δ_H = 7.34 – 7. 28 (m, 2H), 7.24 – 7.18 (m, 3H), 5.07 (d, J =

1.5 Hz, 1H), 4.92 (d, *J* = 1.5 Hz, 1H), 4.08 (s, 2H), 2.69 – 2.63 (m, 2H), 2.19 – 2.10 (m, 2H), 1.87 – 1.79 (m, 2H), 1.76 (br s, 1H); ^{13}C NMR (125 MHz, $CDCl_3$) δ_C = 148.8, 142.3, 128.5, 128.4, 125.9, 109.5, 66.0, 35.7, 32.6, 29.5.

The spectroscopic properties were consistent with the data available in the literature.[15]

4-Methylene-7-phenylheptanoic acid

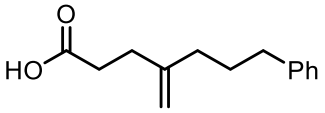

<u>General procedure D</u>: The preceding alcohol (1.94 g, 11.0 mmol) was employed with propionic acid (81.5 mg, 82.1 µL, 1.10 mmol) and triethyl orthoacetate (8.92 g, 10.1 mL, 55.0 mmol) for 5 h at 140 °C; KOH (4.0 M, 27.5 mL, 110 mmol) was used. Purification by flash column chromatography (Hexane/EtOAc = 95/5 to 85/15) afforded the title compound (1.80 g, 75%) as a colorless oil. ^{1}H NMR (500 MHz, $CDCl_3$) δ_H = 11.45 (br s, 1H), 7.33 – 7.27 (m, 2H), 7.23 – 7.17 (m, 3H), 4.83 – 4.80 (m, 1H), 4.79 – 4.76 (m, 1H), 2.66 – 2.60 (m, 2H), 2.55 – 2.49 (m, 2H), 2.36 (t, *J* = 7.7 Hz, 2H), 2.13 – 2.07 (m, 2H), 1.79 (tt, *J* = 9.1, 6.9 Hz, 2H); ^{13}C NMR (125 MHz, $CDCl_3$) δ_C = 179.8, 147.5, 142.4, 128.6, 128.4, 125.9, 109.8, 35.9, 35.6, 32.6, 30.6, 29.5.

The spectroscopic properties were consistent with the data available in the literature.[16]

4-Methylene-7-phenylheptan-1-ol

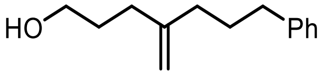

<u>General procedure E</u>: The preceding carboxylic acid (1.09 g, 5.00 mmol) was employed with $LiAlH_4$ (380 mg, 10.0 mmol) in THF at r.t. for 1 h. Purification by flash column chromatography (Hexane/EtOAc = 90/10 to 85/15) afforded the title compound (896 mg, 88%) as a colorless oil. IR (thin film) ν_{max}/cm^{-1}: 3340, 2938, 2862, 1628, 1401, 1055, 888; ^{1}H NMR (500 MHz, C_6D_6) δ_H = 7.19 – 7.13 (m, 3H), 7.11 – 7.03 (m, 2H), 4.83 – 4. 78 (m, 1H), 4.78 – 4.76 (m, 1H), 3.33 (t, *J* = 6.4 Hz, 2H), 2.53 – 2.43 (m, 2H), 2.01 – 1.90 (m, 4H), 1.67 (tt, *J* = 8.8, 6.7 Hz, 2H), 1.55 – 1.45 (m, 2H), 0.89 (br s, 1H); ^{13}C NMR (125 MHz, C_6D_6) δ_C = 149.3, 142.7, 128.4, 127.9, 126.1, 109.5, 62.4, 35.9, 35.9, 32.6, 31.2, 29.9; HRMS (ESI$^+$) calculated for $C_{14}H_{20}NaO$ $[M+Na]^+$ = 227.1406, found 227.1411.

Benzyl (4-methylene-7-phenylheptyl)((perfluorobenzoyl)oxy)carbamate (1e)

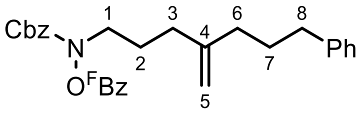

<u>General procedure N</u>: The preceding alcohol (409 mg, 2.00 mmol) was employed with CbzNHO^{F}Bz (795 mg, 2.20 mmol), triphenylphosphine (629 mg, 2.40 mmol) and diisopropyl azodicarboxylate (404 mg, 396 µL, 2.00 mmol). Purification by flash column chromatography (Hexane/EtOAc = 100/0 to

97/3) afforded the title compound (854 mg, 78%) as a colorless oil. IR (thin film) ν_{max}/cm^{-1}: 2944, 2858, 1784, 1651, 1522, 1327, 1003; ^{1}H NMR (400 MHz, CDCl$_3$) δ_H = 7.41 – 7.32 (m, 5H, Cbz ArCH), 7.31 – 7, 26 (m, 2H, Ph ArCH), 7.24 – 7.11 (m, 3H, Ph ArCH), 5.23 (s, 2H, Cbz CH$_2$), 4.78 (s, 1H, C**5**-H), 4.76 (s, 1H, C**5**-H'), 3.76 (t, J = 7.1 Hz, 2H, C**1**-H$_2$), 2.60 (t, J = 7.8 Hz, 2H, C**8**-H$_2$), 2.11 (t, J = 7.7 Hz, 2H, C**3**-H$_2$), 2.05 (t, J = 7.7 Hz, 2H, C**6**-H$_2$), 1.88 – 1.66 (m, 4H, C**2**-H$_2$ + C**7**-H$_2$); ^{19}F NMR (377 MHz, CDCl$_3$) δ_F = -133.7 – -137.5 (m, 2F), -146.04 (tt, J = 20.8, 5.7 Hz, 1F), -156.7 – -161.7 (m, 2F); ^{13}C NMR (101 MHz, CDCl$_3$) δ_C = 155.5 (C=O), 148.0 (C**4**), 142.5 (Ph ArC), 135.3 (Cbz ArC), 128.7 (Cbz ArCH), 128.6 (Cbz ArCH), 128.5 (Ph ArCH), 128.4 (Ph ArCH), 128.3 (Cbz ArCH), 125.8 (Ph ArCH), 110.0 (C**5**), 68.9 (Cbz CH$_2$), 51.1 (C**1**), 35.7 (C**6**), 35.7 (C**8**), 32.8 (C**3**), 29.5 (C**7**), 25.0 (C**2**); HRMS (ESI$^+$) calculated for $C_{29}H_{26}F_5NNaO_4$ [M+Na]$^+$ = 570.1674, found 570.1676.

The carbon signals corresponding to the pentafluorobenzoyl group could not be resolved due to their weak intensity.

Benzyl 1-(3-phenylpropyl)-2-azabicyclo[3.1.0]hexane-2-carboxylate (2e)

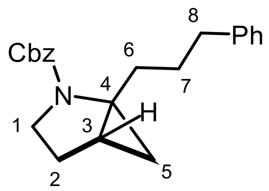

General procedure O: The preceding substrate (54.8 mg, 0.10 mmol) was employed with Pd$_2$(dba)$_3$ (4.58 mg, 0.005 mmol), CgP(2-benzofuryl) (**L3**) (8.31 mg, 0.025 mmol), CsOPiv (23.4 mg, 0.10 mmol), Et$_3$N (14.0 µL, 0.10 mmol) and dibutyl ether (1.0 mL). The reaction was stirred at 140 °C for 6 h. Purification by flash column chromatography (Hexane/EtOAc = 100/0 to 95/5) afforded the title compound (23.5 mg, 70%) as a colorless oil. IR (thin film) ν_{max}/cm^{-1}: 3029, 2935, 1703, 1454, 1404, 1081; ^{1}H NMR (400 MHz, DMSO-d^6, 100 °C) δ_H = 7.60 – 7.27 (m, 5H, Cbz ArCH), 7.27 – 7.23 (m, 2H, Ph ArCH), 7.23 – 7.05 (m, 3H, Ph ArCH), 5.08 (s, 2H, Cbz CH$_2$), 3.64 (ddd, J = 11.5, 9.5, 6.0 Hz, 1H, C**1**-H), 3.40 (ddd, J = 11.3, 8.8, 6.1 Hz, 1H, C**1**-H'), 2.62 – 2.51 (m, 3H, C**6**-H + C**8**-H$_2$), 2.19 – 2.05 (m, 1H, C**2**-H), 1.85 – 1.73 (m, 1H, C**2**-H'), 1.71 – 1.58 (m, 2H, C**7**-H$_2$), 1.43 – 1.34 (m, 1H, C**3**-H), 1.29 – 1.22 (m, 1H, C**6**-H'), 0.86 (dd, J = 8.3, 5.2 Hz, 1H, C**5**-H), 0.64 (dd, J = 5.2, 5.0 Hz, 1H, C**5**-H'); ^{13}C NMR (101 MHz, DMSO-d^6, 100 °C) δ_C = 154.9 (C=O), 141.7 (Ph ArC), 136.7 (Cbz ArC), 127.7 (Cbz ArCH), 127.5 (Ph ArCH), 127.5 (Ph ArCH), 127.0 (Cbz ArCH), 126.8 (Cbz ArCH), 124.9 (Ph ArCH), 65.3 (Cbz CH$_2$), 49.2 (C**1**), 46.6 (C**4**), 34.5 (C**8**), 31.6 (C**6**), 26.9 (C**7**), 25.0 (C**2**), 23.0 (C**3**), 21.1 (C**5**); HRMS (ESI$^+$) calculated for $C_{22}H_{25}NNaO_2$ [M+Na]$^+$ = 358.1777, found 358.1778.

2-Cyclopentylacrylaldehyde

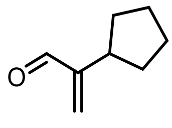

General procedure B: 2-Cyclopentylacetaldehyde (2.02 g, 18.0 mmol) was employed with methylamine hydrochloride (1.47 g, 18.0 mmol) and formaldehyde (1.46 g, 18.0 mmol). Purification by vacuum distillation afforded the title compound (1.66 g, 74%) as a colorless oil. ^{1}H NMR (500 MHz, $CDCl_3$) δ_H = 9.54 (br s, 1H), 6.28 – 6.17 (m, 1H), 5.94 (s, 1H), 2.83 (p, J = 8.3 Hz, 1H), 1.97 – 1.87 (m, 2H), 1.75 – 1.66 (m, 2H), 1.64 – 1.56 (m, 2H), 1.41 – 1.32 (m, 2H); ^{13}C NMR (125 MHz, $CDCl_3$) δ_C = 195.2, 154.3, 132.2, 38.2, 31.7, 25.1.

The spectroscopic properties were consistent with the data available in the literature.[17]

2-Cyclopentylprop-2-en-1-ol

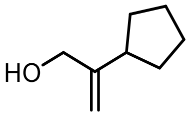

General procedure C: The preceding aldehyde (1.37 g, 11.0 mmol) was employed with $NaBH_4$ (416 mg, 11.0 mmol). Purification by vacuum distillation afforded the title compound (1.21 g, 87%) as a colorless oil. ^{1}H NMR (500 MHz, $CDCl_3$) δ_H = 4.99 – 4.97 (m, 1H), 4.89 – 4.87 (m, 1H), 4.09 (s, 2H), 2.41 (p, J = 8.5 Hz, 1H), 1.88 (br s, 1H), 1.84 – 1.79 (m, 2H), 1.69 – 1.63 (m, 2H), 1.58 – 1.54 (m, 2H), 1.41 – 1.35 (m, 2H); ^{13}C NMR (125 MHz, $CDCl_3$) δ_C = 152.8, 106.8, 66.0, 43.3, 31.6, 25.0;

The spectroscopic properties were consistent with the data available in the literature.[18]

4-Cyclopentylpent-4-enoic acid

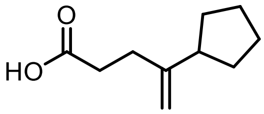

General procedure D: The preceding alcohol (1.39 g, 11.0 mmol) was employed with propionic acid (81.5 mg, 82.1 µL, 1.10 mmol) and triethyl orthoacetate (8.92 g, 10.1 mL, 55.0 mmol) for 12 h at 140 °C; KOH (4.0 M, 27.5 mL, 110 mmol) was used. Purification by flash column chromatography (Hexane/EtOAc = 90/10 to 85/15) afforded the title compound (985 mg, 55%) as a colorless oil. IR (thin film) ν_{max}/cm^{-1}: 2953, 2869, 1709, 1415, 1294, 890; ^{1}H NMR (500 MHz, $CDCl_3$) δ_H = 11.32 (br s, 1H), 4.82 –4.79 (m, 1H), 4.72 – 4.66 (m, 1H), 2.59 – 2.50 (m, 2H), 2.43 – 2.33 (m, 3H), 1.88 – 1.76 (m, 2H), 1.73 – 1.63 (m, 2H), 1.62 – 1.51 (m, 2H), 1.45 – 1.32 (m, 2H); ^{13}C NMR (125 MHz, $CDCl_3$) δ_C = 179.9, 151.3, 107.2, 46.4, 32.8, 31.6, 29.9, 25.1; HRMS (ESI$^+$) calculated for $C_{10}H_{17}O_2$ $[M+H]^+$ = 169.1223, found 169.1221.

4-Cyclopentylpent-4-en-1-ol

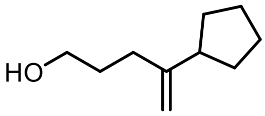

General procedure E: The preceding carboxylic acid (841 mg, 5.00 mmol) was employed with $LiAlH_4$ (380 mg, 10.0 mmol) in THF at r.t. for 1 h. Purification by flash column chromatography (Hexane/EtOAc = 90/10 to 85/15) afforded the title compound (696 mg, 90%) as a colorless oil. IR (thin film) ν_{max}/cm^{-1}: 3323, 2948, 2866, 1641, 1060, 1038, 888; ^{1}H NMR (500 MHz, C_6D_6) δ_H = 4.87 (s, 1H), 4.82 – 4.77 (m, 1H), 3.40 (t, J = 6.4 Hz, 2H), 2.29 (p, J = 8.5 Hz, 1H), 2.09 – 2.01 (m, 2H), 1.78 – 1.68 (m, 2H), 1.65 – 1.54 (m, 4H), 1.52 – 1.42 (m, 2H), 1.40 – 1.31 (m, 2H), 1.13 (br s, 1H); ^{13}C NMR (125 MHz, C_6D_6) δ_C = 152.9, 107.2, 62.5, 46.5, 32.1, 31.9, 31.6, 25.3; HRMS (ESI$^+$) calculated for $C_{10}H_{19}O$ [M+H]$^+$ = 155.1430, found 155.1428.

Benzyl (4-cyclopentylpent-4-en-1-yl)((perfluorobenzoyl)oxy)carbamate (1f)

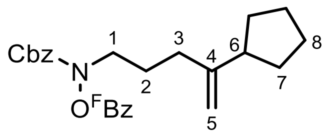

General procedure N: The preceding alcohol (309 mg, 2.00 mmol) was employed with CbzNHO^{F}Bz (795 mg, 2.20 mmol), triphenylphosphine (629 mg, 2.40 mmol) and diisopropyl azodicarboxylate (404 mg, 396 µL, 2.00 mmol). Purification by flash column chromatography (Hexane/EtOAc = 100/0 to 97/3) afforded the title compound (783 mg, 79%) as a colorless solid. m.p. 42 - 44 °C (EtOAc/hexane); IR (thin film) ν_{max}/cm^{-1}: 2951, 2868, 1790, 1524, 1328, 1173, 1006; ^{1}H NMR (400 MHz, $CDCl_3$) δ_H = 7.47 – 7.30 (m, 5H, Cbz ArC<u>H</u>), 5.22 (s, 2H, Cbz C<u>H</u>$_2$), 4.79 (s, 1H, C**5**-<u>H</u>), 4.68 (s, 1H, C**5**-<u>H</u>’), 3.77 (t, J = 7.2 Hz, 2H, C**1**-<u>H</u>$_2$), 2.35 (p, J = 8.5 Hz, 1H, C**6**-<u>H</u>), 2.11 (t, J = 7.8 Hz, 2H, C**3**-<u>H</u>$_2$), 1.90 – 1.74 (m, 4H, C**2**-<u>H</u>$_2$ + C**7**-<u>H</u>$_2$), 1.71– 1.62 (m, 2H, C**8**-<u>H</u>$_2$), 1.60 – 1.51 (m, 2H, C**8**-<u>H</u>$_2$’), 1.40 – 1.28 (m, 2H, C**7**-<u>H</u>$_2$’); ^{19}F NMR (377 MHz, $CDCl_3$) δ_F = -133.5 – -133.2 (m, 2F), -146.1 (tt, J = 20.9, 5.5 Hz, 1F), -157.8 – -160.7 (m, 2F); ^{13}C NMR (101 MHz, $CDCl_3$) δ_C = 155.5 (<u>C</u>=O), 151.9 (C**4**), 135.4 (Cbz Ar<u>C</u>), 128.7 (Cbz Ar<u>C</u>H), 128.6 (Cbz Ar<u>C</u>H), 128.3 (Cbz Ar<u>C</u>H), 107.4 (C**5**), 68.9 (Cbz <u>C</u>H$_2$), 51.2 (C**1**), 46.1 (C**6**), 32.3 (C**3**), 31.6 (C**7**), 25.4 (C**2**), 25.1 (C**8**); HRMS (ESI$^+$) calculated for $C_{25}H_{24}F_5NNaO_4$ [M+Na]$^+$ = 520.1517, found 520.1515.

The carbon signals corresponding to the pentafluorobenzoyl group could not be resolved due to their weak intensity.

Benzyl 1-cyclopentyl-2-azabicyclo[3.1.0]hexane-2-carboxylate (2f)

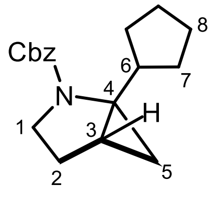

General procedure O: The preceding substrate (49.8 mg, 0.10 mmol) was employed with $Pd_2(dba)_3$ (4.58 mg, 0.005 mmol), CgP(2-benzofuryl) (**L3**) (16.6 mg, 0.05 mmol), CsOPiv (23.4 mg, 0.10 mmol), Et_3N (14.0 µL, 0.10 mmol) and dibutyl ether (1.0 mL). The reaction was stirred at 140 °C for 6 h.

Purification by flash column chromatography (Hexane/EtOAc = 100/0 to 96/4 for the first column, then toluene/EtOAc = 100/0 to 99/1 for the second column) afforded the title compound (17.9 mg, 63%) as a colorless oil. IR (thin film) ν_{max}/cm^{-1}: 2982, 2945, 1702, 1406, 1199, 1087, 947; ^{1}H NMR (400 MHz, DMSO-d^{6}, 100 °C) δ_{H} = 7.57 – 7.14 (m, 5H, Cbz ArCH), 5.11 (d, J = 14.8 Hz, 1H, Cbz CH), 5.08 (d, J = 14.8 Hz, 1H, Cbz CH'), 3.70 – 3.57 (m, 1H, C**1**-H), 3.49 (ddd, J = 11.2, 8.7, 5.3 Hz, 1H, C**1**-H'), 2.92 – 2.84 (m, 1H, C**6**-H), 2.15 – 2.03 (m, 1H, C**2**-H), 1.83 – 1.68 (m, 2H, C**2**-H' + C**7**-H), 1.62 – 1.43 (m, 6H, C**3**-H + C**7**'-H + C**8**-H_2 + C**8**'-H_2), 1.34 – 1.26 (m, 1H, C**7**-H), 0.99 (dd, J = 8.9, 5.2 Hz, 1H, C**5**-H), 0.87 – 0.76 (m, 1H, C**7**'-H'), 0.61 (dd, J = 5.2, 5.2 Hz, 1H, C**5**-H'); ^{13}C NMR (101MHz, DMSO-d^{6}, 100 °C) δ_{C} = 154.8 (C=O), 136.8 (Cbz ArC), 127.6 (Cbz ArCH), 127.0 (Cbz ArCH), 126.7 (Cbz ArCH), 65.2 (Cbz CH_2), 50.1 (C**1**), 50.0 (C**4**), 39.1 (C**6**), 29.5 (C**7**), 27.7 (C**7**'), 25.5 (C**2**), 24.3 (C**8**), 24.1 (C**8**'), 21.0 (C**5**), 19.9 (C**3**); HRMS (ESI^{+}) calculated for $C_{18}H_{23}NNaO_2$ [M+Na]$^{+}$ = 308.1621, found 308.1619.

4-Phenylpent-4-enoic acid

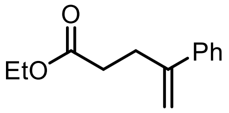

<u>**General procedure D**</u>: 2-Phenylprop-2-en-1-ol (1.34 g, 10.0 mmol) was employed with propionic acid (74.1 mg, 74.5 µL, 1.00 mmol) and triethyl orthoacetate (8.11 g, 9.20 mL, 50.0 mmol) for 5 h at 140 °C. Purification by flash column chromatography (Hexane/EtOAc = 100/0 to 95/5) afforded the title compound (1.56 g, 76%) as a colorless oil. ^{1}H NMR (500 MHz, $CDCl_3$) δ_{H} = 7.47 – 7.39 (m, 2H), 7.36 – 7.31 (m, 2H), 7.30 – 7.26 (m, 1H), 5.31 (s, 1H), 5.15 – 5.05 (m, 1H), 4.12 (q, J = 7.1 Hz, 2H), 2.85 (td, J = 7.6, 1.3 Hz, 2H), 2.51 – 2.41 (m, 2H), 1.24 (t, J = 7.1 Hz, 3H); ^{13}C NMR (125 MHz, $CDCl_3$) δ_{C} = 173.2, 147.1, 140.7, 128.5, 127.7, 126.2, 112.9, 60.5, 33.4, 30.6, 14.4.

The spectroscopic properties were consistent with the data available in the literature.[19]

4-Phenylpent-4-en-1-ol

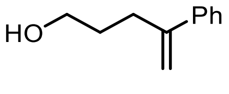

<u>**General procedure E**</u>: The preceding carboxylic acid (1.02 g, 5.00 mmol) was employed with $LiAlH_4$ (380 mg, 10.0 mmol) in Et_2O at 0 °C for 1 h and r.t. for 1 h. Purification by flash column chromatography (Hexane/EtOAc = 90/10 to 85/15) afforded the title compound (645 mg, 80%) as a colorless oil. ^{1}H NMR (400 MHz, C_6D_6) δ_{H} = 7.42 – 7.27 (m, 2H), 7.16 – 7.04 (m, 3H), 5.27 (s, 1H), 5.01 (s, 1H), 3.41 – 3.23 (m, 2H), 2.45 (t, J = 7.7 Hz, 2H), 1.63 – 1.43 (m, 2H), 0.81 (br s, 1H); ^{13}C NMR (101 MHz, C_6D_6) δ_{C} = 148.7, 141.6, 128.6, 127.7, 126.5, 112.5, 62.1, 31.9, 31.6.

The spectroscopic properties were consistent with the data available in the literature.[20]

Benzyl ((perfluorobenzoyl)oxy)(4-phenylpent-4-en-1-yl)carbamate (1g)

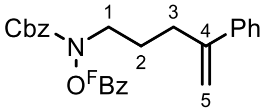

General procedure N: The preceding alcohol (308 mg, 1.90 mmol) was employed with CbzNHO^{F}Bz (755 mg, 2.09 mmol), triphenylphosphine (598 mg, 2.28 mmol) and diisopropyl azodicarboxylate (384 mg, 377 μL, 1.90 mmol). Purification by flash column chromatography (Hexane/EtOAc = 100/0 to 95/5) afforded the title compound (795 mg, 83%) as a colorless oil. IR (thin film) ν_{max}/cm^{-1}: 2949, 1783, 1729, 1653, 1524, 1498, 1326, 1172, 1002, 907; ^{1}H NMR (400 MHz, $CDCl_3$) δ_H = 7.40 – 7.24 (m, 10H, Cbz ArCH + Ph ArCH), 5.28 (d, J = 1.3 Hz, 1H, C**5**-H), 5.21 (s, 2H, Cbz CH_2), 5.11 – 5.05 (m, 1H, C**5**-H'), 3.78 (t, J = 7.0 Hz, 2H, C**1**-H_2), 2.60 (t, J = 7.5 Hz, 2H, C**3**-H_2), 1.88 – 1.75 (m, C**2**-H_2); ^{19}F NMR (377 MHz, $CDCl_3$) δ_F = -135.8 – -136.0 (m, 2F), -146.1 (tt, J = 21.0, 5.5 Hz, 1F), -159.3 – -159.5 (m, 2F); ^{13}C NMR (101 MHz, $CDCl_3$) δ_C = 155.6 (C=O), 147.3 (C**4**), 140.8 (Ph ArC), 135.3 (Cbz ArC), 128.7 (Cbz ArCH), 128.6 (Ph ArCH), 128.4 (Ph ArCH), 128.3 (Cbz ArCH), 127.6 (Cbz ArCH), 126.2 (Ph ArCH), 113.3 (C**5**), 68.9 (Cbz CH_2), 50.8 (C**1**), 32.2 (C**3**), 25.5 (C**2**); HRMS (ESI$^+$) calculated for $C_{26}H_{20}F_5NNaO_4$ [M+Na]$^+$ = 528.1205, found 528.1204.

The carbon signals corresponding to the pentafluorobenzoyl group could not be resolved due to their weak intensity.

Benzyl 1-phenyl-2-azabicyclo[3.1.0]hexane-2-carboxylate (2g)

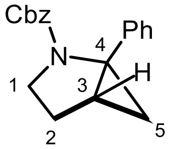

General procedure O: The preceding substrate (50.5 mg, 0.10 mmol) was employed with $Pd_2(dba)_3$ (4.58 mg, 0.005 mmol), CgP(2-benzofuryl) (**L3**) (16.6 mg, 0.05 mmol), CsOPiv (23.4 mg, 0.10 mmol), Et_3N (14.0 μL, 0.10 mmol) and dibutyl ether (1.0 mL). The reaction was stirred at 140 °C for 6 h. Purification by flash column chromatography (Toluene/Et_2O = 100/0 to 95/5) afforded the title compound (12.4 mg, 42%) as a colorless oil. IR (thin film) ν_{max}/cm^{-1}: 3020, 2944, 2879, 1701, 1405, 696; ^{1}H NMR (500 MHz, DMSO-d^6, 100 °C) δ_H = 7.32 – 7.21 (m, 7H, ArCH), 7.20 – 7.15 (m, 1H, ArCH), 7.13 – 7.03 (m, 2H, ArCH), 4.99 (d, 1H, J = 13.0 Hz, Cbz CH), 4.95 (d, 1H, J = 13.0 Hz, Cbz CH'), 3.91 – 3.84 (m, 1H, C**1**-H), 3.69 – 3.63 (m, 1H, C**1**-H'), 2.37 – 2.29 (m, 1H, C**2**-H), 1.97 – 1.90 (m, 1H, C**2**-H), 1.88 – 1.83 (m, 1H, C**5**-H), 1.66 – 1.60 (m, 1H, C**3**-H), 1.08 – 1.04 (m, 1H, C**5**-H'); ^{13}C NMR (125 MHz, DMSO-d^6, 100 °C) δ_C = 155.3 (C=O), 140.7 (Ph ArC), 136.6 (Cbz ArC), 127.8 (Cbz ArCH), 127.6 (Ph ArCH), 127.1 (Ph ArCH), 126.7 (Cbz ArCH), 125.5 (Cbz ArCH), 125.4 (Ph ArCH), 65.6 (Cbz CH_2), 50.6 (C**1**), 49.4 (C**4**), 30.0 (C**3**), 26.3 (C**2**), 23.0 (C**5**); HRMS (ESI$^+$) calculated for $C_{19}H_{19}NNaO_2$ [M+Na]$^+$ = 316.1308, found 316.1311.

(*S*)-4-Benzyl-3-butyryloxazolidin-2-one

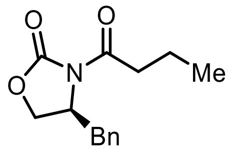

This compound was prepared according to our previously reported literature procedure.[2]

The spectroscopic properties were consistent with the data available in the literature.[2]

(*S*)-4-Benzyl-3-((*R*)-2-ethyl-4-methylenehexanoyl)oxazolidin-2-one

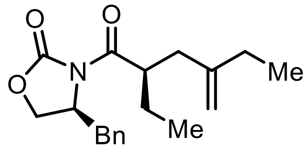

LDA (1.0 M in THF, 8.4 mL, 8.4 mmol) was cooled to -78 °C under N_2. A solution of (*S*)-4-benzyl-3-butyryloxazolidin-2-one (1.73 g, 7.00 mmol) in THF (4.40 mL) was added dropwise. After 30 min, the reaction mixture was warmed to room temperature over 20 min. Then a solution of 2-(iodomethyl)but-1-ene (1.51 g, 7.70 mmol) in THF (4.40 mL) was added dropwise at -78 °C. The resulting yellow solution was stirred at -78 °C for 2 h and warmed to room temperature over 3 h. Upon completion, sat. NH_4Cl solution (10 mL) was added and THF was removed in *vacuo*. The residue was diluted with Et_2O (50 mL) and washed with water (20 mL). The organic phase was washed with brine (20 mL), dried over Na_2SO_4, filtered and concentrated *in vacuo*. The crude product was purified by flash column chromatography (Hexane/EtOAc = 95/5 to 85/15) to afford the title compound (1.41 g, 64%) as a colorless oil. IR (thin film) ν_{max}/cm^{-1}: 2966, 2931, 1779, 1697, 1455, 1388, 1106; $[\alpha]_D^{23}$ = +53.07 (*c* 0.10, $CHCl_3$); ^{1}H NMR (400 MHz, $CDCl_3$) δ_H = 7.38 – 7.31 (m, 2H), 7.31 – 7.26 (m, 1H), 7.23 (d, *J* = 7.4 Hz, 2H), 4.81 (s, 2H), 4.71 (ddt, *J* = 10.3, 6.8, 3.8 Hz, 1H), 4.22 – 4.13 (m, 2H), 4.12 – 4.01 (m, 1H), 3.28 (dd, *J* = 13.3, 3.3 Hz, 1H), 2.69 (dd, *J* = 13.3, 9.9 Hz, 1H), 2.55 (dd, *J* = 14.2, 8.6 Hz, 1H), 2.22 (dd, *J* = 14.2, 6.1 Hz, 1H), 2.18 – 2.04 (m, 2H), 1.81 – 1.66 (m, 1H), 1.63 – 1.54 (m, 1H), 1.06 (dd, *J* = 7.4, 7.4 Hz, 3H), 0.94 (t, *J* = 7.4 Hz, 3H); ^{13}C NMR (101 MHz, $CDCl_3$) δ_C = 176.5, 153.3, 148.8, 135.5, 129.5, 129.0, 127.4, 110.0, 65.9, 55.5, 42.4, 38.9, 38.1, 28.8, 25.5, 12.4, 11.7; HRMS (ESI$^+$) calculated for $C_{19}H_{25}NNaO_3$ $[M+Na]^+$ = 338.1727, found 338.1730.

(*R*)-2-Ethyl-4-methylenehexan-1-ol

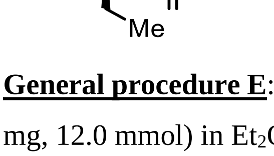

General procedure E: The preceding compound (1.26 g, 4.00 mmol) was employed with $LiAlH_4$ (455 mg, 12.0 mmol) in Et_2O at 0 ºC for 1 h. Purification by flash column chromatography (Hexane/EtOAc = 95/5 to 85/15) afforded the title compound (339 mg, 60%) as a colorless oil. IR (thin film) ν_{max}/cm^{-1}: 3337, 2964, 2923, 2876, 1645, 1461, 889; $[\alpha]_D^{23}$ = +5.79 (*c* 0.10, $CHCl_3$); ^{1}H NMR (400 MHz, $CDCl_3$) δ_H = 4.83 (s, 1H), 4.79 (s, 1H), 3.36 (d, *J* = 5.3 Hz, 2H), 2.11 – 2.03 (m, 1H), 1.99 – 1.87 (m, 3H), 1.54

– 1.40 (m, 1H), 1.40 – 1.28 (m, 2H), 1.26 (br s, 1H), 0.98 (dd, J = 7.4, 7.4 Hz, 3H), 0.85 (t, J = 7.4 Hz, 3H); ^{13}C NMR (101 MHz, $CDCl_3$) δ_C = 150.3, 109.7, 64.9, 40.3, 38.7, 28.8, 23.9, 12.5, 11.5; HRMS (CI^+) calculated for $C_9H_{19}O$ $[M+H]^+$ = 143.1431, found 143.1448.

Benzyl (*R*)-(2-ethyl-4-methylenehexyl)((perfluorobenzoyl)oxy)carbamate (1h)

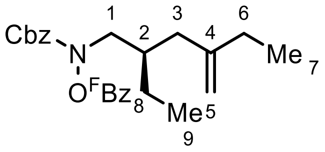

General procedure N: The preceding alcohol (199 mg, 1.40 mmol) was employed with CbzNHO^{F}Bz (556 mg, 1.54 mmol), triphenylphosphine (441 mg, 1.68 mmol) and diisopropyl azodicarboxylate (283 mg, 278 µL, 1.40 mmol). Purification by flash column chromatography (Hexane/EtOAc = 100/0 to 97/3) afforded the title compound (530 mg, 78%) as a colorless oil. IR (thin film) ν_{max}/cm^{-1}: 2967, 2925, 2872, 1784, 1507, 1327, 1033; $[\alpha]_D^{22}$ = +6.12 (c 0.10, $CHCl_3$); ^{1}H NMR (400 MHz, $CDCl_3$) δ_H = 7.52 – 7.28 (m, 5H, Cbz ArC<u>H</u>), 5.22 (s, 2H, Cbz C<u>H</u>$_2$), 4.77 (s, 1H, C**5**-<u>H</u>), 4.70 (s, 1H, C**5**-<u>H</u>'), 3.66 (d, J = 6.9 Hz, 2H, C**1**-<u>H</u>$_2$), 2.07 (d, J = 7.1 Hz, 2H, C**3**-<u>H</u>$_2$), 2.01 – 1.92 (m, 2H, C**8**-<u>H</u>$_2$), 1.91 – 1.82 (m, 1H, C**2**-<u>H</u>), 1.49 – 1.33 (m, 2H, C**6**-<u>H</u>$_2$), 1.00 (dd, J = 7.4, 7.4 Hz, 3H, C**9**-<u>H</u>$_3$), 0.88 (t, J = 7.2 Hz, 3H, C**7**-<u>H</u>$_3$); ^{19}F NMR (377 MHz, $CDCl_3$) δ_F = -133.4 – -137.5 (m, 2F), -146.1 (tt, J = 20.8, 5.6 Hz, 1F), -156.1 – -160.8 (m, 2F); ^{13}C NMR (101 MHz, $CDCl_3$) δ_C = 155.6 (<u>C</u>=O), 149.0 (C**4**), 135.4 (Cbz Ar<u>C</u>), 128.7 (Cbz Ar<u>C</u>H), 128.6 (Cbz Ar<u>C</u>H), 128.3 (Cbz Ar<u>C</u>H), 110.0 (C**5**), 68.9 (Cbz <u>C</u>H$_2$), 54.8 (C**1**), 38.6 (C**3**), 35.5 (C**2**), 28.4 (C**8**), 24.0 (C**6**), 12.3 (C**9**), 10.6 (C**7**); HRMS (ESI^+) calculated for $C_{24}H_{24}F_5NNaO_4$ $[M+Na]^+$ = 508.1518, found 508.1518.

The carbon signals corresponding to the pentafluorobenzoyl group could not be resolved due to their weak intensity.

Benzyl (4*R*)-1,4-diethyl-2-azabicyclo[3.1.0]hexane-2-carboxylate (2h)

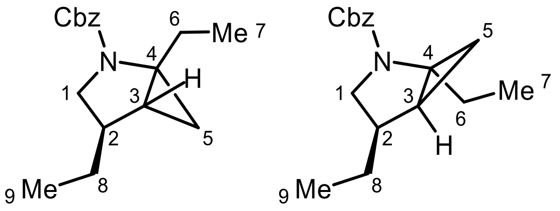

General procedure O: The preceding substrate (48.6 mg, 0.10 mmol) was employed with $Pd_2(dba)_3$ (4.58 mg, 0.005 mmol), CgP(2-benzofuryl) (**L3**) (16.6 mg, 0.025 mmol), CsOPiv (23.4 mg, 0.10 mmol), Et_3N (14.0 µL, 0.10 mmol) and dibutyl ether (1.0 mL). The reaction was stirred at 140 °C for 6 h. Purification by flash column chromatography (Toluene/EtOAc = 100/0 to 99.5/0.5 to remove dba firstly, then Hexane/EtOAc = 100/0 to 95/5) afforded the title compounds (*isomer 1*: 9.88 mg, 36%; *isomer 2*: 12.1 mg, 44%) as colorless oils. *Isomer 1:* IR (thin film) ν_{max}/cm^{-1}: 2967, 2925, 2872, 1707, 1402, 1340, 1055, 1013; ^{1}H NMR (400 MHz, DMSO-d^6, 100 °C) δ_H = 7.49 – 7.15 (m, 5H, Cbz ArC<u>H</u>),

5.08 (s, 2H, Cbz CH$_2$), 3.74 (dd, J = 11.3, 9.1 Hz, 1H, C**1**-H), 2.81 (dd, J = 11.3, 7.8 Hz, 1H, C**1**-H’), 2.50 – 2.43 (m, 1H, C**6**-H), 2.42 – 2.31 (m, 1H, C**2**-H), 1.42 – 1.29 (m, 4H, C**3**-H + C**6**-H’ + C**8**-H$_2$), 0.91 (dd, J = 7.4, 7.4 Hz, 3H, C**9**-H$_3$), 0.87 (t, J = 7.4 Hz, 3H, C**7**-H$_3$), 0.76 (dd, J = 5.4, 5.3 Hz, 1H, C**5**-H), 0.70 (dd, J = 8.8, 5.4 Hz, 1H, C**5**-H’); ^{13}C NMR (101 MHz, DMSO-d^6, 100 °C) δ_C = 155.1 (C=O), 136.7 (Cbz ArC), 127.7 (Cbz ArCH), 127.0 (Cbz ArCH), 126.7 (Cbz ArCH), 65.2 (Cbz CH$_2$), 53.4 (C**1**), 47.8 (C**4**), 37.5 (C**2**), 25.9 (C**6**), 24.8 (C**8**), 24.2 (C**3**), 14.9 (C**5**), 11.5 (C**9**), 9.4 (C**7**); HRMS (ESI$^+$) calculated for $C_{17}H_{23}NNaO_2$ [M+Na]$^+$ = 296.1621, found 296.1617. *Isomer 2:* IR (thin film) ν_{max}/cm^{-1}: 2971, 2925, 2873, 1705, 1405, 1340, 1055, 1033, 1013; ^{1}H NMR (400 MHz, DMSO-d^6, 100 °C) δ_H = 7.39 – 7.28 (m, 5H, Cbz ArCH), 5.11 (d, J = 13.0 Hz, 1H, Cbz CH), 5.07 (d, J = 13.0 Hz, 1H, Cbz CH’), 3.56 (dd, J = 11.4, 7.8 Hz, 1H, C**1**-H), 3.27 (dd, J = 11.4, 5.5 Hz, 1H, C**1**-H’), 2.44 (dt, J = 14.3, 7.3 Hz, 1H, C**6**-H), 2.03 – 1.95 (m, 1H, C**2**-H), 1.50 – 1.41 (m, 2H, C**8**-H$_2$), 1.29 – 1.23 (m, 1H, C**6**-H’), 1.14 (ddd, J = 6.6, 5.1, 2.1 Hz, 1H, C**3**-H), 0.93 (dd, J = 7.4, 7.4 Hz, 3H, C**9**-H$_3$), 0.87 (t, J = 7.4 Hz, 3H, C**7**-H$_3$), 0.67 (dd, J = 6.6, 5.1 Hz, 1H, C**5**-H), 0.67 (dd, J = 5.1, 5.1 Hz, 1H, C**5**-H’); ^{13}C NMR (101 MHz, DMSO-d^6, 100 °C) δ_C = 154.8 (C=O), 136.8 (Cbz ArC), 127.7 (Cbz ArCH), 127.0 (Cbz ArCH), 126.7 (Cbz ArCH), 65.2 (Cbz CH$_2$), 55.3 (C**1**), 47.8 (C**4**), 40.2 (C**2**), 27.2 (C**8**), 27.0 (C**3**), 24.7 (C**6**), 20.8 (C**5**), 11.0 (C**9**), 9.4 (C**7**); HRMS (ESI$^+$) calculated for $C_{17}H_{23}NNaO_2$ [M+Na]$^+$ = 296.1621, found 296.1624.

4-Methylenehexanal

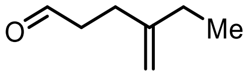

General Procedure A: 4-methylenehexan-1-ol (571 mg, 5.00 mmol) was employed with Dess–Martin periodinane (2.97 g, 7.00 mmol). Purification by flash column chromatography (Pentane/Et_2O = 95/5 to 90/10) afforded the title compound (434 mg, 76%) as a colorless oil. ^{1}H NMR (500 MHz, $CDCl_3$) δ_H = 9.77 (s, 1H), 4.85 – 4.73 (m, 1H), 4.72 – 4.61 (m, 1H), 2.57 (td, J = 7.5, 1.7 Hz, 2H), 2.36 (q, J = 7.5 Hz, 2H), 2.07 – 2.00 (m, 2H), 1.04 (t, J = 7.4 Hz, 3H); ^{13}C NMR (125 MHz, $CDCl_3$) δ_C = 202.4, 149.5, 108.5, 42.0, 29.1, 28.4, 12.4.

The spectroscopic properties were consistent with the data available in the literature.[21]

5-Methyleneheptan-2-ol

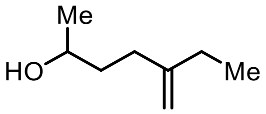

General procedure F: The preceding aldehyde (251 mg, 2.20 mmol) was employed with MeMgBr (3.0 M in Et_2O, 1.50 mL, 4.40 mmol) in Et_2O (11.0 mL) at 0 °C for 2 h. Purification by flash column chromatography (Pentane/Et_2O = 85/15 to 75/25) afforded the title compound (227 mg, 81%) as a colorless oil. IR (thin film) ν_{max}/cm^{-1}: 3338, 2966, 2933, 1646, 1374, 1038, 886; ^{1}H NMR (400 MHz, C_6D_6) δ_H = 4.80 (s, 2H), 3.65 – 3.43 (m, 1H), 2.21 – 2.05 (m, 1H), 2.03 – 1.89 (m, 3H), 1.55 – 1.37 (m,

2H), 1.31 (br s, 1H), 1.02 (d, J = 6.2 Hz, 3H), 0.98 (t, J = 8.0 Hz, 3H); ^{13}C NMR (101 MHz, C_6D_6) δ_C = 151.5, 108.0, 67.6, 37.8, 32.8, 29.2, 23.8, 12.6; HRMS (ESI$^+$) calculated for $C_{16}H_{36}NO_2$ [2M+NH$_4$]$^+$ = 274.2741, found 274.2744.

Benzyl (5-methyleneheptan-2-yl)((perfluorobenzoyl)oxy)carbamate (1i)

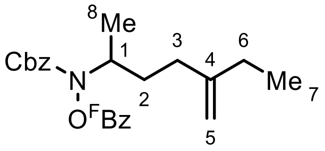

General procedure N: The preceding alcohol (180 mg, 1.40 mmol) was employed with CbzNHO^{F}Bz (556 mg, 1.54 mmol), triphenylphosphine (459 mg, 1.75 mmol) and diisopropyl azodicarboxylate (311 mg, 305 µL, 1.54 mmol). Purification by flash column chromatography (Hexane/EtOAc = 100/0 to 97/3) afforded the title compound (530 mg, 80%) as a colorless oil. IR (thin film) ν_{max}/cm^{-1}: 2968, 2942, 1785, 1523, 1325, 1174, 911; ^{1}H NMR (400 MHz, CDCl$_3$) δ_H = 7.41 – 7.30 (m, 5H, Cbz ArC$\underline{H}$), 5.23 (s, 2H, Cbz C$\underline{H_2}$), 4.72 (s, 1H, C**5**-$\underline{H}$), 4.69 (s, 1H, C**5**-$\underline{H}$'), 4.49 – 4.28 (m, 1H, C**1**-$\underline{H}$), 2.33 – 2.02 (m, 2H, C**3**-$\underline{H_2}$), 1.99 (q, J = 7.5 Hz, 2H, C**6**-$\underline{H_2}$), 1.84 – 1.69 (m, 1H, C**2**-$\underline{H}$), 1.68 – 1.57 (m, 1H, C**2**-$\underline{H}$'), 1.24 (d, J = 6.7 Hz, 3H, C**8**-$\underline{H_3}$), 1.00 (t, J = 7.5 Hz, 3H, C**7**-$\underline{H_3}$); [mixture of rotamers (A:B = 13:1)] ^{19}F NMR (377 MHz, CDCl$_3$) rotamer A δ_F = -135.0 – -136.4 (m, 2F), -145.9 – -146.5 (m, 1F), -158.61 – -159.88 (m, 2F); rotamer B δ_F = -137.1 – -138.3 (m, 2F), -148.5 – -149.2 (m, 1F), -160.0 – -161.5 (m, 2F); ^{13}C NMR (101 MHz, CDCl$_3$) δ_C = 157.3 ($\underline{C}$=O), 150.4 (C**4**), 135.4 (Cbz Ar$\underline{C}$), 128.7 (Cbz Ar$\underline{C}$H), 128.6 (Cbz Ar$\underline{C}$H), 128.2 (Cbz Ar$\underline{C}$H), 108.3 (C**5**), 68.9 (Cbz $\underline{C}$H$_2$), 56.6 (C**1**), 32.8 (C**3**), 31.8 (C**2**), 28.9 (C**6**), 17.6 (C**8**), 12.4 (C**7**); HRMS (ESI$^+$) calculated for $C_{23}H_{22}F_5NNaO_4$ [M+Na]$^+$ = 494.1361, found 494.1365.

The carbon signals corresponding to the pentafluorobenzoyl group could not be resolved due to their weak intensity.

Some signal broadening was observed due to amide-like resonance resulting in weak signal intensities in ^{13}C NMR spectrum.

Benzyl 1-ethyl-3-methyl-2-azabicyclo[3.1.0]hexane-2-carboxylate (2i)

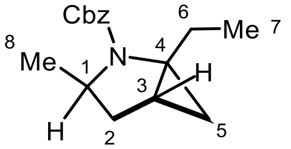

General procedure O: The preceding substrate (47.1 mg, 0.10 mmol) was employed with Pd$_2$(dba)$_3$ (4.58 mg, 0.005 mmol), CgP(2-benzofuryl) (**L3**) (16.6 mg, 0.05 mmol), CsOPiv (23.4 mg, 0.10 mmol), Et$_3$N (14.0 µL, 0.10 mmol) and dibutyl ether (1.0 mL). The reaction was stirred at 140 °C for 6 h. Purification by flash column chromatography (Hexane/EtOAc = 100/0 to 95/5) afforded the title compound (13.5 mg, >15:1 dr, 52%) as a colorless oil. IR (thin film) ν_{max}/cm^{-1}: 2964, 2931, 1700, 1454,

1345, 1091; ^{1}H NMR (400 MHz, DMSO-*d*^{6}, 100 °C) δ_H = 7.50 – 7.22 (m, 5H, Cbz ArCH), 5.09 (s, 2H, Cbz CH_2), 3.98 (dqd, *J* = 12.7, 6.4, 3.2 Hz, 1H, C**1**-H), 2.33 (dq, *J* = 14.6, 7.4 Hz, 1H, C**6**-H), 2.01 (dd, *J* = 13.0, 8.3 Hz, 1H, C**2**-H), 1.75 (ddd, *J* = 13.0, 7.1, 3.4 Hz, 1H, C**2**-H'), 1.44 – 1.37 (m, 1H, C**3**-H), 1.36 – 1.29 (m, 1H, C**6**-H'), 1.20 (d, *J* = 6.4 Hz, 2H, C**8**-H_3), 0.92 (dd, *J* = 8.8, 5.0 Hz, 1H, C**5**-H), 0.87 (t, *J* = 7.4 Hz, 3H, C**7**-H_3), 0.49 (dd, *J* = 4.9, 4.9 Hz, 1H, C**5**-H'); ^{13}C NMR (101 MHz, DMSO-*d*^{6}, 100 °C) δ_C = 154.8 (C=O), 136.8 (Cbz ArC), 127.7 (Cbz ArCH), 127.0 (Cbz ArCH), 126.7 (Cbz ArCH), 65.2 (Cbz CH_2), 58.5 (C**1**), 48.2 (C**4**), 34.6 (C**2**), 24.9 (C**6**), 23.3 (C**5**), 21.6 (C**8**), 21.0 (C**3**), 9.8 (C**7**); HRMS (ESI$^+$) calculated for $C_{16}H_{21}NNaO_2$ [M+Na]$^+$ = 282.1464, found 282.1463.

2-((2,4-Dinitrophenyl)sulfonyl)-1-ethyl-3-methyl-2-azabicyclo[3.1.0]hexane (2i')

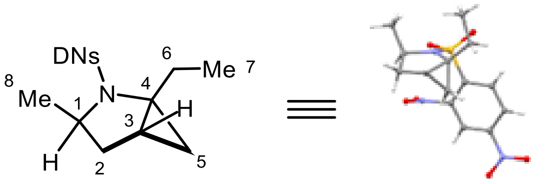

(**CCDC** 2189444)

A mixture of benzyl 1-ethyl-3-methyl-2-azabicyclo[3.1.0]hexane-2-carboxylate (25.9 mg, 0.10 mmol) and Palladium on carbon (10 wt. % loading, 10.7 mg, 0.01 mmol) in a solution of HCl in EtOAc (1.0 M, 0.35 mL) and EtOH (1.75 mL) was vigorously stirred for 24 h at room temperature under an atmosphere of H_2 (1 atm). The reaction mixture was filtered on a dicalite pad and the pad was washed with EtOH (3.00 mL). After the filtrate was concentrated *in vacuo*, the crude mixture was dissolved in DCM (1.0 mL) and cooled to 0 °C. Then 2,4-dinitrobenzenesulfonyl chloride (32.0 mg, 0.12 mmol) and Et_3N (30.4 mg, 41.8 µL, 0.30 mmol) were added at 0 °C, and the reaction mixture was stirred for 12 h at room temperature. The reaction mixture was purified by flash column chromatography (Hexane/EtOAc = 95/5 to 85/15) to afford the title compound (18.9 mg, 53%) as a colorless solid. m.p. 136 - 138 °C ($CHCl_3$/hexane); IR (thin film) ν_{max}/cm^{-1}: 2964, 2931, 1700, 1454, 1345, 1314, 1091; ^{1}H NMR (500 MHz, $CDCl_3$) δ_H = 8.48 (dd, *J* = 8.7, 2.2 Hz, 1H, DNs ArCH), 8.39 (d, *J* = 2.2 Hz, 1H, DNs ArCH), 8.24 (d, *J* = 8.7 Hz, 1H, DNs ArCH), 4.10 (dqd, *J* = 12.7, 6.5, 3.7 Hz, 1H, C**1**-H), 2.51 (dq, *J* = 14.5, 7.3 Hz, 1H, C**6**-H), 2.05 (ddd, *J* = 13.1, 12.7, 3.7 Hz, 1H, C**2**-H), 1.81 (ddd, *J* = 13.1, 7.0, 3.7 Hz, 1H, C**2**-H'), 1.45 – 1.40 (m, 1H, C**3**-H), 1.38 (d, *J* = 6.5 Hz, 3H, C**8**-H_3), 1.22 (dd, *J* = 14.5, 7.3 Hz, 1H, C**6**-H'), 1.07 (dd, *J* = 7.3 Hz, 3H, C**7**-H_3), 0.79 (dd, *J* = 8.8, 5.5 Hz, 1H, C**5**-H), 0.13 (dd, *J* = 5.5, 5.3 Hz, 1H, C**5**-H'); ^{13}C NMR (125 MHz, $CDCl_3$) δ_C = 149.8 (DNs ArC), 148.6 (DNs ArC), 138.1 (DNs ArC), 133.6 (DNs ArCH), 125.5 (DNs ArCH), 119.4 (DNs ArCH), 63.5 (C**1**), 51.2 (C**4**), 35.8 (C**2**), 27.4 (C**6**), 24.2 (C**8**), 23.0 (C**5**), 22.5 (C**3**), 10.9 (C**7**); HRMS (ESI$^+$) calculated for $C_{14}H_{17}N_3NaO_6S$ [M+Na]$^+$ = 378.0730, found 378.0727.

4-Methylpent-4-enoic acid

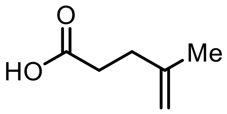

General procedure D: 2-Methylprop-2-en-1-ol (6.00 g, 83.2 mmol) was employed with propionic acid (431 mg, 434 µL, 5.82 mmol) and trimethyl orthoacetate (27.8 g, 29.4 mL, 231 mmol) for 3 h at 150 °C; KOH (4.0 M, 208 mL, 832 mmol) was used. Purification by flash column chromatography (Hexane/EtOAc = 95/5 to 85/15) afforded the title compound (6.09 g, 56%) as a colorless oil. ^{1}H NMR (400 MHz, $CDCl_3$) δ_H = 11.14 (br s, 1H), 4.76 (s, 1H), 4.71 (s, 1H), 2.51 (t, *J* = 7.7 Hz, 2H), 2.34 (t, *J* = 7.7 Hz, 2H), 1.75 (s, 3H).

The spectroscopic properties were consistent with the data available in the literature.[22]

4-Methylpent-4-en-1-ol

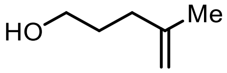

General procedure E: The preceding carboxylic acid (4.95 g, 38.0 mmol) was employed with $LiAlH_4$ (2.88 g, 76.0 mmol) in THF at r.t. for 1 h. Purification by flash column chromatography (Hexane/EtOAc = 90/10 to 85/15) afforded the title compound (2.75 g, 72%) as a colorless oil. ^{1}H NMR (400 MHz, $CDCl_3$) δ_H = 4.80 – 4.67 (m, 2H), 3.66 (t, *J* = 6.5 Hz, 2H), 2.10 (t, *J* = 7.7 Hz, 2H), 1.74 (s, 3H), 1.73 – 1.66 (m, 2H), 1.45 (br s, 1H); ^{13}C NMR (101 MHz, $CDCl_3$) δ_C = 145.6, 110.3, 62.8, 34.2, 30.6, 22.5.

The spectroscopic properties were consistent with the data available in the literature.[23]

Benzyl (4-methylpent-4-en-1-yl)((perfluorobenzoyl)oxy)carbamate (1j)

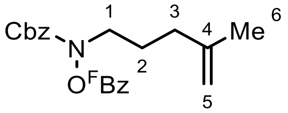

General procedure N: The preceding alcohol (1.50 g, 15.0 mmol) was employed with CbzNHO^{F}Bz (6.50 g, 18.0 mmol), triphenylphosphine (4.72 g, 18.0 mmol) and diisopropyl azodicarboxylate (3.34 g, 3.30 mL, 16.5 mmol). Purification by flash column chromatography (Hexane/EtOAc = 100/0 to 97/3) afforded the title compound (3.83 g, 58%) as a colorless oil. IR (thin film) ν_{max}/cm^{-1}: 2970, 1786, 1730, 1524, 1501, 1173; ^{1}H NMR (400 MHz, $CDCl_3$) δ_H = 7.44 – 7.27 (m, 5H, Cbz ArC<u>H</u>), 5.22 (s, 2H, Cbz C<u>H$_2$</u>), 4.77 – 4.71 (m, 1H, C**5**-<u>H</u>), 4.70 – 4.67 (m, 1H, C**5**-<u>H</u>’), 3.75 (t, *J* = 7.2 Hz, 2H, C**1**-<u>H$_2$</u>), 2.09 (t, *J* = 7.6 Hz, 2H, C**3**-<u>H$_2$</u>), 1.84 – 1.75 (m, 2H, C**2**-<u>H$_2$</u>), 1.70 (s, 3H, C**6**-<u>H$_3$</u>); ^{19}F NMR (377 MHz, $CDCl_3$) δ_F = -136.0 – -136.2 (m, 2F), -146.1 (tt, *J* = 21.0, 5.5 Hz, 1F), -159.2 – -159.4 (m, 2F); ^{13}C NMR (101 MHz, $CDCl_3$) δ_C = 155.6 (<u>C</u>=O), 144.4 (C**4**), 135.4 (Cbz Ar<u>C</u>), 128.7 (Cbz Ar<u>C</u>H), 128.6 (Cbz Ar<u>C</u>H), 128.3 (Cbz Ar<u>C</u>H), 110.9 (C**5**), 68.9 (Cbz <u>C</u>H$_2$), 51.0 (C**1**), 34.5 (C**3**), 24.9 (C**2**), 22.4 (C**6**); HRMS (ESI$^+$) calculated for $C_{21}H_{18}F_5NNaO_4$ $[M+Na]^+$ = 466.1048, found 466.4053.

The carbon signals corresponding to the pentafluorobenzoyl group could not be resolved due to their weak intensity.

Benzyl 1-methyl-2-azabicyclo[3.1.0]hexane-2-carboxylate (2j) and benzyl 4-azaspiro[2.4]heptane-4-carboxylate (*iso*-2j)

General procedure O: The preceding substrate (44.3 mg, 0.10 mmol) was employed with $Pd_2(dba)_3$ (4.58 mg, 0.005 mmol), CgP(2-benzofuryl) (**L3**) (8.31 mg, 0.025 mmol), CsOPiv (23.4 mg, 0.10 mmol), Et_3N (14.0 µL, 0.10 mmol) and dibutyl ether (1.0 mL). The reaction was stirred at 140 °C for 6 h. Purification by flash column chromatography (Hexane/EtOAc = 100/0 to 95/5) afforded the title compounds (**2j**: 12.8 mg, 55%; ***iso*-2j**: 3.3 mg, 14%) as colorless oils.

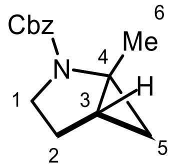

IR (thin film) ν_{max}/cm^{-1}: 2962, 2879, 1699, 1404; ^{1}H NMR (500 MHz, DMSO-d^6, 100 °C) δ_H = 7.40 – 7.34 (m, 4H, Cbz ArCH), 7.33 – 7.28 (m, 1H, Cbz ArCH), 5.10 (1H, d, J = 12.5 Hz, Cbz CH), 5.07 (1H, d, J = 12.5 Hz, Cbz CH'), 3.65 (ddd, J = 11.3, 9.7, 5.1 Hz, 1H, C**1**-H), 3.30 (ddd, J = 11.3, 8.8, 6.5 Hz, 1H, C**1**-H'), 2.11 (dddd, J = 12.9, 9.7, 6.5, 6.5 Hz, 1H, C**2**-H), 1.80 – 1.73 (m, 1H, C**2**-H'), 1.53 (s, 3H, C**6**-H_3), 1.36 – 1.31 (m, 1H, C**3**-H), 0.83 (dd, J = 8.6, 5.2 Hz, 1H, C**5**-H), 0.66 (dd, J = 5.2, 5.2 Hz, 1H, C**5**-H'); ^{13}C NMR (125 MHz, DMSO-d^6, 100 °C) δ_C = 155.2 (C=O), 136.8 (Cbz ArC), 127.9 (Cbz ArCH), 127.2 (Cbz ArCH), 126.9 (Cbz ArCH), 65.4 (Cbz CH_2), 48.4 (C**1**), 42.5 (C**4**), 24.9 (C**2**), 24.3 (C**3**), 21.1 (C**5**), 19.4 (C**6**); HRMS (ESI$^+$) calculated for $C_{14}H_{18}NO_2$ $[M+H]^+$ = 232.1332, found 232.1337.

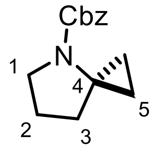

IR (thin film) ν_{max}/cm^{-1}: 2966, 2877, 1699, 1411; ^{1}H NMR (500 MHz, DMSO-d^6, 100°C) δ_C = 7.39 – 7.29 (5H, m, Cbz ArCH), 5.01 (s, 2H, Cbz CH_2), 3.53 (t, J = 6.8 Hz, 2H, C**1**-H_2), 1,91 – 1.84 (m, 4H, C**2**-H_2 + C**3**-H_2), 1.53 – 1.46 (m, 2H, C**5**-H_2), 0.51 – 0.46 (m, 2H, C**5**-H_2'); ^{13}C NMR (125 MHz, DMSO-d^6, 100°C) δ 152.0 (C=O), 136.7 (Cbz ArC), 127.8 (Cbz ArCH), 127.2 (Cbz ArCH), 126.9 (Cbz ArCH), 65.0 (Cbz CH_2), 47.6 (C**1**), 42.4 (C**4**), 35.2 (C**3**), 21.9 (C**2**), 8.2 (C**5**); HRMS (ESI$^+$) calculated for $C_{14}H_{18}NO_2$ $[M+H]^+$ = 232.1332, found 232.1332.

Some signal broadening was observed in the NMR spectra due to amide-like resonance, despite running at high temperature.

3-Ethyl-4-methylpent-4-enoic acid

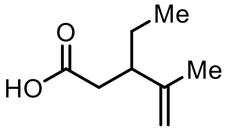

General procedure D: 2-Methylpent-2-en-1-ol (4.00 g, 40.0 mmol) was employed with propionic acid (296 mg, 298 µL, 4.00 mmol) and triethyl orthoacetate (32.5 g, 36.7 mL, 200 mmol) for 5 h at 140 °C; KOH (4.0 M, 100 mL, 400 mmol) was used. Purification by flash column chromatography (Hexane/EtOAc = 95/5 to 85/15) afforded the title compound (3.37 g, 59%) as a colorless oil. IR (thin film) ν_{max}/cm^{-1}: 2966, 2933, 1708, 1412, 1293, 893; ^{1}H NMR (400 MHz, $CDCl_3$) δ_H = 10.73 (br s, 1H), 4.82 – 4.78 (m, 1H), 4.78 – 4.73 (m, 1H), 2.54 – 2.44 (m, 1H), 2.43 – 2.38 (m, 2H), 1.66 (s, 3H), 1.51 – 1.37 (m, 2H), 0.84 (dd, J = 7.4, 7.4 Hz, 3H); ^{13}C NMR (101 MHz, $CDCl_3$) δ_C = 179.5, 145.9, 112.3, 45.1, 38.9, 25.9, 19.0, 11.6; HRMS (ESI$^+$) calculated for $C_8H_{15}O_2$ [M+H]$^+$ = 143.1067, found 143.1067.

3-Ethyl-4-methylpent-4-en-1-ol

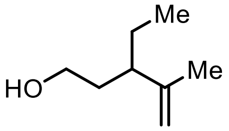

General procedure E: The preceding carboxylic acid (1.99 g, 14.0 mmol) was employed with $LiAlH_4$ (1.06 g, 28.0 mmol) in THF at r.t. for 1 h. Purification by flash column chromatography (Hexane/EtOAc = 90/10 to 85/15) afforded the title compound (1.58 g, 88%) as a colorless oil. IR (thin film) ν_{max}/cm^{-1}: 3324, 2961, 2930, 1055, 888; ^{1}H NMR (400 MHz, C_6D_6) δ_H = 4.80 – 4.76 (m, 1H), 4.77 – 4.73 (m, 1H), 3.52 – 3.41 (m, 2H), 2.13 – 2.00 (m, 1H), 2.06 (br s, 1H), 1.54 – 1.45 (m, 2H), 1.50 (s, 3H), 1.31 – 1.20 (m, 2H), 0.80 (dd, J = 7.4, 7.4 Hz, 3H); ^{13}C NMR (125 MHz, C_6D_6) δ_C = 147.4, 112.2, 61.1, 46.1, 36.5, 26.5, 17.9, 12.1; HRMS (APCI$^+$) calculated for $C_8H_{17}O$ [M+H]$^+$ = 129.1274, found 129.1266.

Benzyl (3-ethyl-4-methylpent-4-en-1-yl)((perfluorobenzoyl)oxy)carbamate (1k)

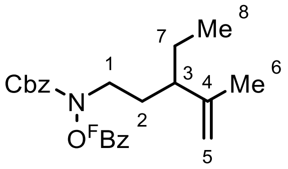

General procedure N: The preceding alcohol (769 mg, 6.00 mmol) was employed with CbzNHO^{F}Bz (2.38 g, 6.60 mmol), triphenylphosphine (1.89 g, 7.20 mmol) and diisopropyl azodicarboxylate (1.21 g, 1.19 mL, 6.00 mmol). Purification by flash column chromatography (Hexane/EtOAc = 100/0 to 97/3) afforded the title compound (2.02 g, 71%) as a colorless oil. IR (thin film): 2963, 2934, 1786, 1730, 1524, 1499, 1174; ^{1}H NMR (500 MHz, $CDCl_3$) δ_H = 7.54 – 7.29 (m, 5H, Cbz ArCH), 5.22 (s, 2H, Cbz CH_2), 4.86 – 4.75 (m, 1H, C**5**-H), 4.73 – 4.65 (m, 1H, C**5**-H’), 3.67 (t, J = 7.6 Hz, 2H, C**1**-H_2), 2.10 –

1.98 (m, 1H, C**3**-H), 1.78 – 1.62 (m, 2H, C**2**-H_2), 1.58 (s, 3H, C**6**-H_3), 1.44 – 1.28 (m, 2H, C**7**-H_2), 0.81 (dd, *J* = 7.4, 7.4 Hz, 3H, C**8**-H_3); ^{19}F NMR (377 MHz, $CDCl_3$) δ_F = -134.6 – -137.8 (m, 2F), -146.2 (tt, *J* = 20.8, 5.3 Hz, 1F), -157.8 – -161.9 (m, 2F); ^{13}C NMR (125 MHz, $CDCl_3$) δ_C = 155.5 (C=O), 145.9 (C**4**), 135.4 (Cbz ArC), 128.7 (Cbz ArCH), 128.6 (Cbz ArCH), 128.3 (Cbz ArCH), 112.8 (C**5**), 68.8 (Cbz CH_2), 50.1 (C**1**), 46.4 (C**3**), 29.9 (C**2**), 26.2 (C**7**), 17.8 (C**6**), 11.9 (C**8**); HRMS (ESI$^+$) calculated for $C_{23}H_{22}F_5NNaO_4$ $[M+Na]^+$ = 494.1361, found 494.1362.

The carbon signals corresponding to the pentafluorobenzoyl group could not be resolved due to their weak intensity.

Benzyl 7-ethyl-4-azaspiro[2.4]heptane-4-carboxylate (*iso*-2k)

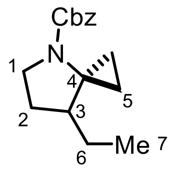

General procedure O: The preceding substrate (47.1 mg, 0.10 mmol) was employed with $Pd_2(dba)_3$ (4.58 mg, 0.005 mmol), CgP(2-benzofuryl) (**L3**) (9.97 mg, 0.03 mmol), KOAc (19.6 mg, 0.20 mmol), Et_3N (14.0 µL, 0.10 mmol) and dibutyl ether (2.0 mL). The reaction was stirred at 160 °C for 24 h. Purification by flash column chromatography (Hexane/EtOAc = 100/0 to 97/3) afforded the title compound (18.9 mg, 73%) as a colorless oil. IR (thin film) ν_{max}/cm^{-1}: 2962, 2932, 2876, 1700, 1408; ^{1}H NMR (500 MHz, DMSO-d^6, 100 °C) δ_H = 7.48 – 7.24 (m, 5H, Cbz ArCH), 5.01 (s, 2H, Cbz CH_2), 3.56 (ddd, *J* = 10.9, 8.0, 5.4 Hz, 1H, C**1**-H), 3.44 (ddd, *J* = 10.9, 7.4, 7.4 Hz, 1H, C**1**-H'), 2.08 – 2.01 (m, 1H, C**2**-H), 1.83 (dtd, *J* = 10.5, 6.7, 4.0 Hz, C**3**-H), 1.59 – 1.51 (m, 1H, C**2**-H'), 1.49 – 1.38 (m, 2H, C**5**-H + C**5'**-H), 1.29 – 1.23 (m, 1H, C**6**-H), 1.14 – 1.05 (m, 1H, C**6**-H'), 0.89 (dd, *J* = 7.4, 7.4 Hz, 3H, C**7**-H_3), 0.60 (ddd, *J* = 10.7, 5.6, 5.1 Hz, 1H, C**5**-H'), 0.41 (ddd, *J* = 9.6, 5.6, 3.9 Hz, 1H, C**5'**-H'); ^{13}C NMR (125 MHz, DMSO-d^6, 100 °C) δ_C = 152.2 (C=O), 136.7 (Cbz ArC), 127.8 (Cbz ArCH), 127.1 (Cbz ArCH), 126.9 (Cbz ArCH), 65.0 (Cbz CH_2), 45.9 (C**4**), 45.6 (C**1**), 45.0 (C**3**), 27.0 (C**2**), 22.4 (C**6**), 11.2 (C**7**), 6.9 (C**5'**), 4.8 (C**5**); HRMS (ESI$^+$) calculated for $C_{16}H_{21}NNaO_2$ $[M+Na]^+$ = 282.1465, found 282.1478.

3-Isopropyl-4-methylpent-4-enoic acid

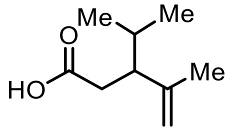

General procedure D: 2,4-Dimethylpent-2-en-1-ol (822 mg, 7.20 mmol) was employed with propionic acid (53.3 mg, 53.7 µL, 0.72 mmol) and triethyl orthoacetate (5.84 g, 6.60 mL, 36.0 mmol) for 5 h at 140 °C; KOH (4.0 M, 18.0 mL, 72.0 mmol) was used. Purification by flash column chromatography

(Hexane/EtOAc = 95/5 to 85/15) afforded the title compound (590 mg, 52%) as a colorless oil. ^{1}H NMR (500 MHz, $CDCl_3$) δ_H = 11.10 (br s, 1H), 4.82– 4.78 (m, 1H), 4.74 – 4.69 (m, 1H), 2.56 (dd, J = 14.1, 3.9 Hz, 1H), 2.36 – 2.23 (m, 2H), 1.68 (s, 3H), 1.68 – 1.61 (m, 1H), 0.89 (d, J = 9.6 Hz, 3H), 0.88 (d, J = 9.6 Hz, 3H); ^{13}C NMR (125 MHz, $CDCl_3$) δ_C = 179.8, 145.9, 112.7, 50.5, 36.4, 30.0, 21.2, 20.1, 20.0.

The spectroscopic properties were consistent with the data available in the literature.[24]

3-Isopropyl-4-methylpent-4-en-1-ol

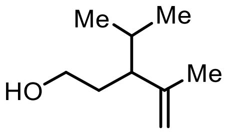

General procedure E: The preceding carboxylic acid (578 mg, 3.70 mmol) was employed with $LiAlH_4$ (281 mg, 7.40 mmol) in THF at r.t. for 1 h. Purification by flash column chromatography (Hexane/EtOAc = 90/10 to 85/15) afforded the title compound (338 mg, 64%) as a colorless oil. IR (thin film) ν_{max}/cm^{-1}: 3345, 2960, 2913, 1595, 1013, 887; ^{1}H NMR (500 MHz, $CDCl_3$) δ_H = 4.79 (dq, J = 2.9, 1.5 Hz, 1H), 4.72 (dq, J = 2.9, 0.7 Hz, 1H), 3.65 – 3.58 (m, 1H), 3.56 – 3.47 (m, 1H), 1.86 – 1.76 (m, 2H), 1.62 (dd, J = 1.5, 0.7 Hz, 3H), 1.56 – 1.47 (m, 2H), 1.48 (br s, 1H), 0.92 (d, J = 6.6 Hz, 3H), 0.82 (d, J = 6.7 Hz, 3H); ^{13}C NMR (125 MHz, $CDCl_3$) δ_C = 147.4, 112.7, 62.2, 52.1, 33.0, 30.1, 21.5, 20.8, 18.7; HRMS (CI$^+$) calculated for $C_9H_{19}O$ $[M+H]^+$ = 143.1431, found 143.1440.

Benzyl (3-isopropyl-4-methylpent-4-en-1-yl)((perfluorobenzoyl)oxy)carbamate (1l)

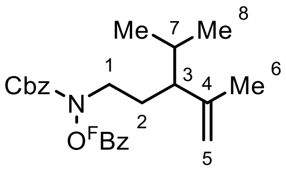

General procedure N: The preceding alcohol (228 mg, 1.60 mmol) was employed with CbzNHO^{F}Bz (636 mg, 1.76 mmol), triphenylphosphine (503 mg, 1.92 mmol) and diisopropyl azodicarboxylate (324 mg, 317 µL, 1.60 mmol). Purification by flash column chromatography (Hexane/EtOAc = 100/0 to 97/3) afforded the title compound (456 mg, 59%) as a colorless oil. IR (thin film) ν_{max}/cm^{-1}: 2960, 2941, 1786, 1523, 1326, 997, 905; ^{1}H NMR (500 MHz, $CDCl_3$) δ_H = 7.44 – 7.27 (m, 5H, Cbz ArC$\underline{H}$), 5.21 (s, 2H, Cbz C$\underline{H_2}$), 4.80 (dt, J = 3.0, 1.2 Hz, 1H, C**5**-$\underline{H}$), 4.71 – 4.63 (m, 1H, C**5**-$\underline{H}$'), 3.69 – 3.55 (m, 2H, C**1**-$\underline{H_2}$), 1.91 (dtd, J = 13.3, 8.2, 3.4 Hz, 1H, C**2**-$\underline{H}$), 1.78 – 1.68 (m, C**3**-$\underline{H}$), 1.58 (dd, J = 1.2, 1.0 Hz, 3H, C**6**-$\underline{H_3}$), 1.57 – 1.51 (m, 2H, C**2**-$\underline{H}$' + C**7**-$\underline{H}$), 0.89 (d, J = 6.5 Hz, 3H, C**8**-$\underline{H_3}$), 0.82 (d, J = 6.6 Hz, 3H, C**8'**-$\underline{H_3}$); ^{19}F NMR (377 MHz, $CDCl_3$) δ_F = -131.9 – -138.3 (m, 2F), -146.2 (tt, J = 20.8, 5.2 Hz, 1F), -156.4 – -161.3 (m, 2F); ^{13}C NMR (125 MHz, $CDCl_3$) δ_C = 155.5 ($\underline{C}$=O), 145.5 (C**4**), 135.4 (Cbz Ar$\underline{C}$), 128.7 (Cbz Ar$\underline{C}$H), 128.6 (Cbz Ar$\underline{C}$H), 128.3 (Cbz Ar$\underline{C}$H), 113.4 (C**5**), 68.8 (Cbz $\underline{C}H_2$), 52.1

(C**3**), 50.5 (C**1**), 30.1 (C**7**), 26.8 (C**2**), 21.4 (C**8'**), 20.5 (C**8**), 18.7 (C**6**); HRMS (ESI$^+$) calculated for $C_{24}H_{24}F_5NNaO_4$ [M+Na]$^+$ = 508.1518, found 508.1524.

The carbon signals corresponding to the pentafluorobenzoyl group could not be resolved due to their weak intensity.

Benzyl 7-isopropyl-4-azaspiro[2.4]heptane-4-carboxylate (*iso*-2l)

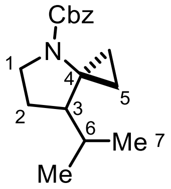

General procedure O: The preceding substrate (48.6 mg, 0.10 mmol) was employed with $Pd_2(dba)_3$ (4.58 mg, 0.005 mmol), CgP(2-benzofuryl) (**L3**) (16.6 mg, 0.05 mmol), KOAc (19.6 mg, 0.20 mmol), Et_3N (14.0 µL, 0.10 mmol) and dibutyl ether (2.0 mL). The reaction was stirred at 160 °C for 24 h. Purification by flash column chromatography (Hexane/EtOAc = 100/0 to 97/3) afforded the title compound (12.0 mg, 44%) as a colorless oil. IR (thin film) ν_{max}/cm^{-1}: 2981, 2883, 1702, 1406, 1250, 1055, 1013; ^{1}H NMR (400 MHz, DMSO-d^6, 100 °C) δ_H = 7.58 – 7.11 (m, 5H, Cbz ArC<u>H</u>), 5.02 (s, 2H, Cbz C<u>H₂</u>), 3.63 – 3.52 (m, 1H, C**1**-<u>H</u>), 3.42 (ddd, *J* = 8.9, 5.5, 5.5 Hz, 1H, C**1**-<u>H</u>'), 2.02 – 1.89 (m, 1H, C**2**-<u>H</u>), 1.84 (ddd, *J* = 10.8, 6.0, 6.0 Hz, 1H, C**5**-<u>H</u>), 1.79 – 1.67 (m, 2H, C**3**-<u>H</u> + C**2**-<u>H</u>'), 1.66 – 1.55 (m, 1H, C**6**-<u>H</u>), 1.15 (ddd, *J* = 10.7, 6.8, 4.4 Hz, 1H, C**5'**-<u>H</u>), 0.90 (d, *J* = 6.8 Hz, 3H, C**7**-<u>H₃</u>), 0.86 (d, *J* = 6.7 Hz, 3H, C**7'**-<u>H₃</u>), 0.75 (ddd, *J* = 10.8, 6.0, 6.0 Hz, 1H, C**5**-<u>H</u>'), 0.42 (ddd, *J* = 10.7, 6.8, 4.4 Hz, 1H, C**5'**-<u>H</u>'); ^{13}C NMR (101 MHz, DMSO-d^6, 100 °C) δ_C = 152.1 (<u>C</u>=O), 136.7 (Cbz Ar<u>C</u>), 127.7 (Cbz Ar<u>C</u>H), 127.0 (Cbz Ar<u>C</u>H), 126.7 (Cbz Ar<u>C</u>H), 64.9 (Cbz <u>C</u>H₂), 49.3 (C**3**), 46.0 (C**1**), 44.9 (C**4**), 27.6 (C**6**), 23.3 (C**2**), 20.8 (C**7**), 18.0 (C**7'**), 9.0 (C**5'**), 6.0 (C**5**); HRMS (ESI$^+$) calculated for $C_{17}H_{23}NNaO_2$ [M+Na]$^+$ = 296.1621, found 296.1617.

2-(1-(Prop-1-en-2-yl)cyclopentyl)acetic acid

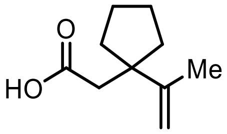

General procedure D: 2-Cyclopentylidenepropan-1-ol (379 mg, 3.00 mmol) was employed with propionic acid (22.2 mg, 22.4 µL, 0.30 mmol) and triethyl orthoacetate (2.43 g, 2.75 mL, 15.0 mmol) for 72 h at 180 °C in sealed tube; KOH (4.0 M, 7.50 mL, 30.0 mmol) was used. Purification by flash column chromatography (Hexane/EtOAc = 95/5 to 85/15) afforded the title compound (244 mg, 48%) as a colorless oil. IR (thin film) ν_{max}/cm^{-1}: 2955, 2921, 1694, 1447, 1319, 963, 892; ^{1}H NMR (500 MHz, $CDCl_3$) δ_H = 11.15 (br s, 1H), 4.83 – 4,79 (m, 1H), 4.78 – 4.75(m, 1H), 2.45 (s, 2H), 1.84 – 1.74 (m,

2H), 1.78 (s, 3H), 1.71 – 1.62 (m, 6H); ^{13}C NMR (101 MHz, $CDCl_3$) δ_C = 178.7, 148.6, 110.9, 51.0, 42.7, 36.5, 23.2, 20.3; HRMS (ESI$^-$) calculated for $C_{10}H_{15}O_2$ [M-H]$^-$ = 167.1078, found 167.1074.

2-(1-(Prop-1-en-2-yl)cyclopentyl)ethan-1-ol

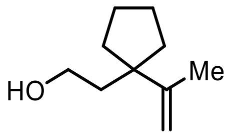

General procedure E: The preceding carboxylic acid (168 mg, 1.00 mmol) was employed with $LiAlH_4$ (75.9 mg, 2.00 mmol) in THF at r.t. for 1 h. Purification by flash column chromatography (Hexane/EtOAc = 90/10 to 85/15) afforded the title compound (90.3 mg, 59%) as a colorless oil. IR (thin film) ν_{max}/cm^{-1}: 3316, 2954, 1637, 1452, 1033, 889; ^{1}H NMR (500 MHz, C_6D_6) δ_H = 4.77 – 4.74 (m, 1H), 4.71 – 4.68 (m, 1H), 3.42 (t, *J* = 7.3 Hz, 2H), 1.63 (s, 3H), 1.62 – 1.54 (m, 2H), 1.56 (t, *J* = 7.3 Hz, 2H), 1.54 – 1.47 (m, 4H), 1.36 – 1.30 (m, 2H), 1.09 (br s, 1H); ^{13}C NMR (125 MHz, C_6D_6) δ_C = 150.4, 110.2, 60.5, 50.8, 40.3, 36.6, 23.4, 20.2; HRMS (ESI$^+$) calculated for $C_{10}H_{19}O$ [M+H]$^+$ = 155.1430, found 155.1428.

Benzyl ((perfluorobenzoyl)oxy)(2-(1-(prop-1-en-2-yl)cyclopentyl)ethyl)carbamate (1m)

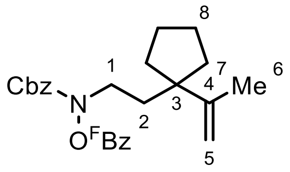

General procedure N: The preceding alcohol (77.1 mg, 0.50 mmol) was employed with CbzNHO^{F}Bz (199 mg, 0.55 mmol), triphenylphosphine (157 mg, 0.60 mmol) and diisopropyl azodicarboxylate (101 mg, 99.1 µL, 0.50 mmol). Purification by flash column chromatography (Hexane/EtOAc = 100/0 to 97/3) afforded the title compound (161 mg, 65%) as a colorless oil. IR (thin film) ν_{max}/cm^{-1}: 2957, 2875, 1786, 1652, 1499, 1175, 907; ^{1}H NMR (400 MHz, $CDCl_3$) δ_H = 7.42 – 7.30 (m, 5H, Cbz ArC<u>H</u>), 5.21 (s, 2H, Cbz C<u>H$_2$</u>), 4.82 – 4.78 (m, 1H, C**5**-<u>H</u>), 4,71 – 4.67 (m, 1H, C**5**-<u>H</u>’), 3.61 – 3.52 (m, 2H, C**1**-<u>H$_2$</u>), 1.85 – 1.70 (m, 4H, C**2**-<u>H$_2$</u> + C**7**-<u>H$_2$</u>), 1.69 (s, 3H, C**6**-<u>H$_3$</u>), 1.65 – 1.59 (m, 4H, C**8**-<u>H$_2$</u> + C**8’**-<u>H$_2$</u>), 1.48 – 1.40 (m, 2H, C**7’**-<u>H$_2$</u>); ^{19}F NMR (377 MHz, $CDCl_3$) δ_F = -135.6 – 136.7 (m, 2F), -146.2 (tt, *J* = 20.9, 5.4 Hz, 1F), -158.4 – -159.8 (m, 2F); ^{13}C NMR (101 MHz, $CDCl_3$) δ_C = 155.5 (<u>C</u>=O), 148.5 (C**4**), 135.4 (Cbz Ar<u>C</u>), 128.7 (Cbz Ar<u>C</u>H), 128.6 (Cbz Ar<u>C</u>H), 128.3 (Cbz Ar<u>C</u>H), 111.0 (C**5**), 68.8 (Cbz <u>C</u>H$_2$), 50.4 (C**3**), 49.1 (C**1**), 36.2 (C**7**), 36.2 (C**7’**), 33.7 (C**2**), 23.2 (C**8**), 23.2 (C**8’**), 19.9 (C**6**); HRMS (ESI$^+$) calculated for $C_{25}H_{24}F_5NNaO_4$ [M+Na]$^+$ = 520.1518, found 520.1517.

The carbon signals corresponding to the pentafluorobenzoyl group could not be resolved due to their weak intensity.

Benzyl 11-azadispiro[2.0.4^4.3^3]undecane-11-carboxylate (*iso*-2m)

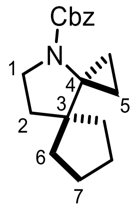

General procedure O: The preceding substrate (49.8 mg, 0.10 mmol) was employed with $Pd_2(dba)_3$ (4.58 mg, 0.005 mmol), CgP(2-benzofuryl) (**L3**) (9.97 mg, 0.03 mmol), KOAc (19.6 mg, 0.20 mmol), Et_3N (14.0 µL, 0.10 mmol) and dibutyl ether (2.0 mL). The reaction was stirred at 160 °C for 24 h. Purification by flash column chromatography (Hexane/EtOAc = 100/0 to 97/3) afforded the title compound (20.2 mg, 71%) as a colorless oil. IR (thin film) ν_{max}/cm^{-1}: 2956, 2927, 1706, 1407, 1359, 1014; ^{1}H NMR (400 MHz, DMSO-d^6, 100 °C) δ_H = 7.64 – 7.13 (m, 5H, Cbz ArC<u>H</u>), 5.03 (s, 2H, Cbz C<u>H</u>$_2$), 3.48 (t, J = 7.0 Hz, 2H, C**1**-<u>H</u>$_2$), 1.74 (t, J = 7.0 Hz, 2H, C**2**-<u>H</u>$_2$), 1.68 – 1.54 (m, 4H, C**7**-<u>H</u>$_2$ + C**7'**-<u>H</u>$_2$), 1.48 – 1.36 (m, 4H, C**5**-<u>H</u>$_2$ + C**6**-<u>H</u>$_2$), 1.28 – 1.16 (m, 2H, C**6'**-<u>H</u>$_2$), 0.50 – 0.44 (m, 2H, C**5'**-<u>H</u>$_2$); ^{13}C NMR (101 MHz, DMSO-d^6, 100 °C) δ_C = 152.3 (<u>C</u>=O), 136.7 (Cbz Ar<u>C</u>), 127.7 (Cbz Ar<u>C</u>H), 127.0 (Cbz Ar<u>C</u>H), 126.8 (Cbz Ar<u>C</u>H), 64.9 (Cbz <u>C</u>H$_2$), 50.8 (C**4**), 48.2 (C**3**) , 44.3 (C**1**), 34.7 (C**2**), 33.4 (C**6**), 23.2 (C**7**), 5.2 (C**5**); HRMS (ESI$^+$) calculated for $C_{18}H_{23}NNaO_2$ $[M+Na]^+$ = 308.1621, found 308.1619.

Methyl 2-(2-methylenebutyl)benzoate

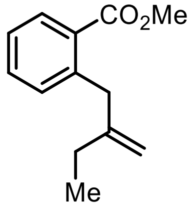

General Procedure H: Methyl-2-iodobenzoate (1.21 g, 4.60 mmol) was employed with magnesium turnings (268 mg, 11.0 mmol), bromobutane (1.26 g, 993 µL, 9.20 mmol), LiCl (468 mg, 11.0 mmol), CuCN (494 mg, 5.52 mmol) and 2-(bromomethyl)but-1-ene (2.06 g, 13.8 mmol). Purification by flash column chromatography (Hexane/Et_2O = 100/0 to 95/5) afforded the title compound (756 mg, 80%) as a colorless oil. IR (thin film) ν_{max}/cm^{-1}: 2966, 2878, 1721, 1433, 1258, 1081, 890; ^{1}H NMR (500 MHz, $CDCl_3$) δ_H = 7.85 (dd, J = 7.6, 1.4 Hz, 1H), 7.45 – 7.41 (m, 1H), 7.29 – 7.24 (m, 2H), 4.82 – 4.76 (m, 1H), 4.45 – 4.39 (m, 1H), 3.86 (s, 3H), 3.73 (s, 2H), 2.10 – 2.00 (m, 2H), 1.05 (t, J = 7.4 Hz, 3H); ^{13}C NMR (125 MHz, $CDCl_3$) δ_C = 168.4, 151.2, 141.2, 131.8, 131.6, 130.6, 130.5, 126.3, 109.4, 52.1, 40.6, 29.3, 12.4; HRMS (ESI$^+$) calculated for $C_{13}H_{17}O_2$ $[M+H]^+$ = 205.1223, found 205.1222.

(2-(2-Methylenebutyl)phenyl)methanol

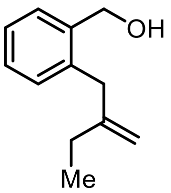

<u>General procedure E</u>: The preceding ester (449 mg, 2.20 mmol) was employed with $LiAlH_4$ (217 mg, 5.72 mmol) in Et_2O at r.t. for 3 h. Purification by flash column chromatography (Hexane/EtOAc = 90/10 to 85/15) afforded the title compound (344 mg, 89%) as a colorless oil. IR (thin film) ν_{max}/cm^{-1}: 3307, 2965, 1645, 1454, 1211, 1180, 890; ^{1}H NMR (400 MHz, C_6D_6) δ_H = 7.45 – 7.30 (m, 1H), 7.15 – 7.02 (m, 3H), 4.79 (s, 1H), 4.57 (s, 1H), 4.46 (d, *J* = 5.5 Hz, 2H), 3.25 (s, 2H), 1.89 (q, *J* = 7.5 Hz, 2H), 1.36 (br t, *J* = 5.5 Hz, 1H), 0.93 (t, *J* = 7.5 Hz, 3H); ^{13}C NMR (101 MHz, C_6D_6) δ_C = 150.9, 140.1, 137.6, 130.7, 128.3, 127.7, 126.8, 109.9, 63.0, 39.7, 29.2, 12.5; HRMS (CI$^+$) calculated for $C_{12}H_{17}O$ $[M+H]^+$ = 177.1274, found 177.1279.

Benzyl (2-(2-methylenebutyl)benzyl)((perfluorobenzoyl)oxy)carbamate (1n)

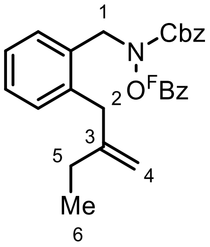

<u>General procedure N</u>: The preceding alcohol (317 mg, 1.80 mmol) was employed with CbzNHO^{F}Bz (780 mg, 2.16 mmol), triphenylphosphine (566 mg, 2.16 mmol) and diisopropyl azodicarboxylate (400 mg, 393 µL, 1.98 mmol). Purification by flash column chromatography (Hexane/EtOAc = 100/0 to 97/3) afforded the title compound (797 mg, 85%) as a colorless solid. m.p. 70 - 72 °C (EtOAc/hexane); IR (thin film) ν_{max}/cm^{-1}: 2992, 1796, 1654, 1520, 1201, 987; ^{1}H NMR (500 MHz, $CDCl_3$) δ_H = 7.38 – 7.31 (m, 5H, Cbz ArC<u>H</u>), 7.29 (dd, *J* = 7.5, 1.4 Hz, 1H, ArC<u>H</u>), 7.27 – 7.23 (m, 1H, ArC<u>H</u>), 7.20 – 7.14 (m, 2H, ArC<u>H</u>), 5.26 (s, 2H, Cbz C<u>H</u>$_2$), 4.92 (s, 2H, C**1**-<u>H</u>$_2$), 4.80 – 4.77 (m, 1H, C**4**-<u>H</u>), 4.44 – 4.40 (m, 1H, C**4**-<u>H</u>’), 3.42 (s, 2H, C**2**-<u>H</u>$_2$), 2.01 (q, *J* = 7.4 Hz, 2H, C**5**-<u>H</u>$_2$), 1.03 (t, *J* = 7.4 Hz, 3H, C**6**-<u>H</u>$_3$); ^{19}F NMR (377 MHz, $CDCl_3$) δ_F = -132.8 – -136.9 (m, 2F), -146.2 (tt, *J* = 20.9, 5.7 Hz, 1F), -156.1 – -163.1 (m, 2F); ^{13}C NMR (125 MHz, $CDCl_3$) δ_C = 155.4 (<u>C</u>=O), 150.0 (C**3**), 138.7 (Ar<u>C</u>), 135.2 (Cbz Ar<u>C</u>), 132.7 (Ar<u>C</u>), 130.9 (Ar<u>C</u>H), 129.5 (Ar<u>C</u>H), 128.7 (Cbz Ar<u>C</u>H), 128.7 (Cbz Ar<u>C</u>H), 128.5 (Ar<u>C</u>H), 128.3 (Cbz Ar<u>C</u>H), 126.6 (Ar<u>C</u>H), 109.9 (C**4**), 69.1 (Cbz <u>C</u>H$_2$), 52.6 (C**1**), 39.7 (C**2**), 29.1 (C**5**), 12.4 (C**6**); HRMS (ESI$^+$) calculated for $C_{27}H_{22}F_5NNaO_4$ $[M+Na]^+$ = 542.1361, found 542.1365.

The carbon signals corresponding to the pentafluorobenzoyl group could not be resolved due to their weak intensity.

Benzyl 1a-ethyl-1,1a,3,7b-tetrahydro-2*H*-cyclopropa[*c*]isoquinoline-2-carboxylate (2n)

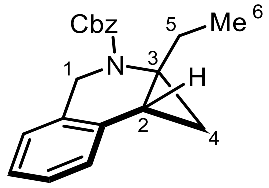

General procedure O: The preceding substrate (52.0 mg, 0.10 mmol) was employed with Pd_2(dba-MeO-*p*)$_3$ (5.48 mg, 0.005 mmol), CgP(2-benzofuryl) (**L3**) (8.31 mg, 0.025 mmol), CsOPiv (23.4 mg, 0.10 mmol), Et_3N (14.0 µL, 0.10 mmol) and dibutyl ether (1.0 mL). The reaction was stirred at 130 °C for 6 h. Purification by flash column chromatography (Hexane/EtOAc = 100/0 to 97/3) afforded the title compound (23.3 mg, 76%) as a colorless oil. IR (thin film) ν_{max}/cm^{-1}: 2963, 2927, 1701, 1409, 1222, 1087; [mixture of rotamers (A:B = 7:3)] ^{1}H NMR (500 MHz, $CDCl_3$) δ_H = 7.54 – 7.33 (m, 5H, Cbz ArCH, A + B), 7.33 – 7.26 (m, 1H, ArCH, A + B), 7.25 – 7.17 (m, 1H, ArCH, A + B), 7.16 – 6.96 (m, 2H, ArCH, A + B), 5.30 – 5.02 (m, 2H, Cbz CH_2, A + B), 4.91 (d, *J* = 15.6 Hz, 0.7H, C**1**-H, A), 4.78 (d, *J* = 15.5 Hz, 0.3H, C**1**-H, B), 4.24 (d, *J* = 15.5 Hz, 0.3H, C**1**-H', B), 4.15 (d, *J* = 15.6 Hz, 0.7H, C**1**-H', A), 2.55 – 2.32 (m, 1H, C**5**-H, A + B), 2.11 – 1.91 (m, 1H, C**2**-H, A + B), 1.42 – 1.31 (m, 1H, C**4**-H, A + B), 1.29 – 1.20 (m, 1H, C**5**-H', A + B), 1.14 – 0.96 (m, 3H, C**6**-H_3, A + B), 0.93 – 0.86 (m, 1H, C**4**-H', A + B); ^{13}C NMR (125 MHz, $CDCl_3$) δ_C = 156.8 (C=O, A), 155.0 (C=O, B), 137.1 (Cbz ArC, A), 136.9 (Cbz ArC, B), 136.4 (ArC, B), 136.0 (ArC, A), 135.3 (ArC, A), 135.1 (ArC, B), 129.5 (ArCH, B), 129.3 (ArCH, A), 128.6 (Cbz ArCH, A + B), 128.1 (Cbz ArCH, B), 128.0 (ArCH, B), 127.9 (ArCH, A), 127.7 (Cbz ArCH, A), 127.5 (Cbz ArCH, B), 127.4 (Cbz ArCH, A), 126.0 (ArCH, A), 126.0 (ArCH, A), 125.7 (ArCH, B), 125.7 (ArCH, B), 67.3 (Cbz CH_2, A), 67.1 (Cbz CH_2, B), 46.0 (C**1**, B), 45.6 (C**1**, A), 41.2 (C**3**, B), 40.5 (C**3**, A), 30.2 (C**5**, A), 28.9 (C**5**, B), 27.1 (C**4**, A), 26.4 (C**4**, B), 22.5 (C**2**, A), 22.2 (C**2**, B), 10.6 (C**6**, A + B); HRMS (ESI$^+$) calculated for $C_{20}H_{21}NNaO_2$ [M+Na]$^+$ = 330.1465, found 330.1470.

Methyl 2-(2-methyleneoctyl)benzoate

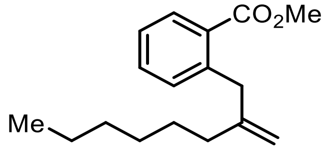

General Procedure H: Methyl-2-iodobenzoate (1.21 g, 4.60 mmol) was employed with magnesium turnings (268 mg, 11.0 mmol), bromobutane (1.26 g, 993 µL, 9.20 mmol), LiCl (468 mg, 11.0 mmol), CuCN (494 mg, 5.52 mmol) and 2-(bromomethyl)oct-1-ene (3.77 g, 18.4 mmol). Purification by flash column chromatography (Hexane/Et_2O = 100/0 to 95/5) afforded the title compound (1.06 g, 89%) as a colorless oil. IR (thin film) ν_{max}/cm^{-1}: 2954, 2928, 1726, 1433, 1261, 1079, 890; ^{1}H NMR (500 MHz, $CDCl_3$) δ_H = 7.85 (dd, *J* = 7.7, 1.4 Hz, 1H), 7.45 – 7.39 (m, 1H), 7.30 – 7.23 (m, 2H), 4.80 – 4.76 (m, 1H), 4.44 – 4.39 (m, 1H), 3.86 (s, 3H), 3.71 (s, 2H), 2.03 (t, *J* = 7.7 Hz, 2H), 1.49 – 1.42 (m, 2H), 1.31

– 1.26 (m, 6H), 0.88 (t, J = 6.8 Hz, 3H); ^{13}C NMR (125 MHz, $CDCl_3$) δ_C = 168.4, 149.7, 141.2, 131.8, 131.7, 130.6, 130.5, 126.2, 110.5, 52.0, 40.4, 36.6, 31.9, 29.2, 27.8, 22.8, 14.2; HRMS (ESI$^+$) calculated for $C_{17}H_{25}O_2$ [M+H]$^+$ = 261.1849, found 261.1849.

(2-(2-Methyleneoctyl)phenyl)methanol

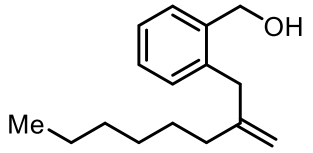

<u>General procedure E</u>: The preceding ester (911 mg, 3.50 mmol) was employed with $LiAlH_4$ (345 mg, 9.10 mmol) in Et_2O at r.t. for 3 h. Purification by flash column chromatography (Hexane/EtOAc = 90/10 to 85/15) afforded the title compound (706 mg, 87%) as a colorless oil. IR (thin film) ν_{max}/cm^{-1}: 3330, 2924, 2860, 1454, 1033, 880; ^{1}H NMR (400 MHz, C_6D_6) δ_H = 7.44 – 7.32 (m, 1H), 7.15 – 7.06 (m, 3H), 4.83 (s, 1H), 4.59 (s, 1H), 4.49 (s, 2H), 3.29 (s, 2H), 1.96 (t, J = 7.7 Hz, 2H), 1.50 – 1.34 (m, 2H), 1.30 – 1.16 (m, 6H), 1.21 (br s, 1H), 0.89 (t, J = 6.6 Hz, 3H); ^{13}C NMR (101 MHz, C_6D_6) δ_C = 149.6, 140.1, 137.6, 130.7, 128.4, 128.3, 126.9, 111.0, 63.1, 39.7, 36.6, 32.1, 29.4, 28.2, 23.1, 14.4; HRMS (CI$^+$) calculated for $C_{16}H_{25}O$ [M+H]$^+$ = 233.1900, found 233.1904.

Benzyl (2-(2-methyleneoctyl)benzyl)((perfluorobenzoyl)oxy)carbamate (1o)

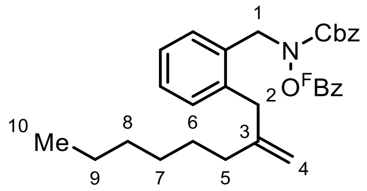

<u>General procedure N</u>: The preceding alcohol (418 mg, 1.80 mmol) was employed with CbzNHO^{F}Bz (780 mg, 2.16 mmol), triphenylphosphine (566 mg, 2.16 mmol) and diisopropyl azodicarboxylate (400 mg, 393 µL, 1.98 mmol). Purification by flash column chromatography (Hexane/EtOAc = 100/0 to 97/3) afforded the title compound (766 mg, 74%) as a colorless solid. m.p. 49 - 51 °C (EtOAc/hexane); IR (thin film) ν_{max}/cm^{-1}: 2924, 2862, 1784, 1733, 1522, 1499, 1327, 1174, 1054, 1033, 1006; ^{1}H NMR (400 MHz, $CDCl_3$) δ_H = 7.46 –7.33 (m, 5H, Cbz ArC<u>H</u>), 7.32 – 7.29 (m, 1H, ArC<u>H</u>), 7.29 – 7.23 (m, 1H, ArC<u>H</u>), 7.22 – 7.12 (m, 2H, ArC<u>H</u>), 5.27 (s, 2H, Cbz C<u>H</u>$_2$), 4.93 (s, 2H, C**1**-<u>H</u>$_2$), 4.79 (s, 1H, C**4**-<u>H</u>), 4.43 (s, 1H, C**4**-<u>H</u>'), 3.41 (s, 2H, C**2**-<u>H</u>$_2$), 2.01 (t, J = 7.7 Hz, 2H, C**5**-<u>H</u>$_2$), 1.50 – 1.40 (m, 2H. C**6**-<u>H</u>$_2$), 1.33 – 1.24 (m, 6H, C**7**-<u>H</u>$_2$ + C**8**-<u>H</u>$_2$ + C**9**-<u>H</u>$_2$), 0.90 (t, J = 6.3 Hz, 3H, C**10**-<u>H</u>$_3$); ^{19}F NMR (377 MHz, $CDCl_3$) δ_F = -134.6 – -136.9 (m, 2F), -146.2 (tt, J = 21.1, 5.3 Hz, 1F), -158.7 – -161.0 (m, 2F); ^{13}C NMR (101 MHz, $CDCl_3$) δ_C = 155.5 (<u>C</u>=O), 148.6 (C**3**), 138.6 (Ar<u>C</u>), 135.2 (Cbz Ar<u>C</u>), 132.8 (Ar<u>C</u>), 130.9 (Ar<u>C</u>H), 129.5 (Ar<u>C</u>H), 128.7 (Cbz Ar<u>C</u>H), 128.6 (Cbz Ar<u>C</u>H), 128.4 (Ar<u>C</u>H), 128.3 (Cbz Ar<u>C</u>H),, 126.6 (Ar<u>C</u>H), 111.0 (C**4**), 69.1 (Cbz <u>C</u>H$_2$), 52.6 (C**1**), 39.6 (C**2**), 36.5 (C**5**), 31.9 (C**8**), 29.2

(C**7**), 27.9 (C**6**), 22.8 (C**9**), 14.2 (C**10**); HRMS (ESI$^+$) calculated for $C_{31}H_{30}F_5NNaO_4$ [M+Na]$^+$ = 598.1987, found 598.1991.

The carbon signals corresponding to the pentafluorobenzoyl group could not be resolved due to their weak intensity.

Benzyl 1a-hexyl-1,1a,3,7b-tetrahydro-2*H*-cyclopropa[*c*]isoquinoline-2-carboxylate (2o)

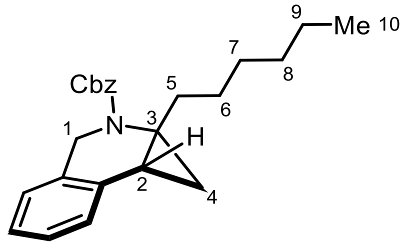

General procedure O: The preceding substrate (57.6 mg, 0.10 mmol) was employed with Pd_2(dba-MeO-*p*)$_3$ (5.48 mg, 0.005 mmol), CgP(2-benzofuryl) (**L3**) (8.31 mg, 0.025 mmol), CsOPiv (23.4 mg, 0.10 mmol), Et_3N (14.0 µL, 0.10 mmol) and dibutyl ether (1.0 mL). The reaction was stirred at 130 °C for 6 h. Purification by flash column chromatography (Hexane/EtOAc = 100/0 to 97/3) afforded the title compound (24.7 mg, 68%) as a colorless oil. IR (thin film) ν_{max}/cm^{-1}: 2956, 2929, 1703, 1408, 1347, 1069, 754; [mixture of rotamers (A:B = 7:3)] ^{1}H NMR (500 MHz, $CDCl_3$) δ_H = ^{1}H NMR (500 MHz, Chloroform-*d*) δ 7.50 – 7.33 (m, 5H, Cbz ArCH, A + B), 7.33 – 7.27 (m, 1H, ArCH, A + B), 7.24 – 7.18 (m, 1H, ArCH, A + B), 7.17 – 7.00 (m, 2H, ArCH, A + B), 5.26 – 5.05 (m, 2H, Cbz CH_2, A + B), 4.90 (d, *J* = 15.5 Hz, 0.7H, C**1**-H, A), 4.77 (d, *J* = 15.8 Hz, 1H, 0.3H, C**1**-H, B), 4.23 (d, *J* = 15.8 Hz, 1H, 0.3H, C**1**-H’, B), 4.14 (d, *J* = 15.5 Hz, 0.7H, C**1**-H’, A), 2.54 – 2.29 (m, 1H, C**5**-H, A + B), 2.12 – 2.01 (m, 1H, C**2**-H, A + B), 1.58 – 1.40 (m, 2H, C**6**-H_2, A + B), 1.35 – 1.21 (m, 7H, C**4**-H, A + B and C**7**-H_2, A + B and C**8**-H_2, A + B and C**9**-H_2, A + B), 1.19 – 1.03 (m, 1H, C**5**-H’, A + B), 0.92 – 0.82 (m, 4H, C**4**-H’, A + B and C**10**-H_3, A + B); ^{13}C NMR (125 MHz, $CDCl_3$) δ_C = 156.8 (C=O, A), 155.0 (C=O, B), 137.1 (Cbz ArC, A), 136.9 (Cbz ArC, B), 136.4 (ArC, B), 136.0 (ArC, A), 135.3 (ArC, A), 135.1 (ArC, B), 129.5 (ArCH, B), 129.3 (ArCH, A), 128.6 (Cbz ArCH, A + B), 128.0 (Cbz ArCH, B), 128.0 (ArCH, B), 127.9 (ArCH, A), 127.8 (Cbz ArCH, A), 127.5 (Cbz ArCH, B), 127.4 (Cbz ArCH, A), 126.0 (ArCH, A), 126.0 (ArCH, A), 125.7 (ArCH, B), 125.7 (ArCH, B), 67.2 (Cbz CH_2, A), 67.1 (Cbz CH_2, B), 45.9 (C**1**, B), 45.5 (C**1**, A), 40.3 (C**3**, B), 39.5 (C**3**, A), 37.5 (C**5**, A), 36.2 (C**5**, B), 32.0 (C**8**, A + B), 29.6 (C**7**, A + B), 27.2 (C**4**, A), 26.5 (C**4**, B), 26.3 (C**6**, A + B), 23.2 (C**2**, A), 22.8 (C**2,** B), 22.7 (C**9**, A + B), 14.2 (C**10**, A + B); HRMS (ESI$^+$) calculated for $C_{24}H_{29}NNaO_2$ [M+Na]$^+$ = 386.2091, found 386.2094.

Methyl 2-(3-methyl-2-methylenebutyl)benzoate

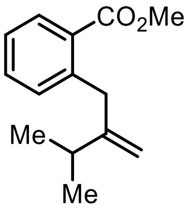

General Procedure H: Methyl-2-iodobenzoate (786 mg, 3.00 mmol) was employed with magnesium turnings (175 mg, 7.20 mmol), bromobutane (822 mg, 647 µL, 6.00 mmol), LiCl (305 mg, 7.20 mmol), CuCN (323 mg, 3.60 mmol) and 2-(bromomethyl)-3-methylbut-1-ene (1.96 g, 12.0 mmol). Purification by flash column chromatography (Hexane/Et_2O = 100/0 to 95/5) afforded the title compound (502 mg, 77%) as a colorless oil. IR (thin film) ν_{max}/cm^{-1}: 2960, 2872, 1725, 1434, 1130, 892; ^1H NMR (500 MHz, $CDCl_3$) δ_H = 7.85 (dd, J = 7.8, 1.5 Hz, 1H), 7.45 – 7.40 (m, 1H), 7.30 – 7.22 (m, 2H), 4.84 – 4.78 (m, 1H), 4.32 – 4.22 (m, 1H), 3.85 (s, 3H), 3.78 – 3.71 (m, 2H), 2.28 (hept, J = 6.9 Hz, 1H), 1.09 (d, J = 6.9 Hz, 6H); ; ^{13}C NMR (125 MHz, $CDCl_3$) δ_C = 168.4, 155.8, 141.3, 131.9, 131.8, 130.7, 130.5, 126.2, 108.4, 52.1, 38.9, 34.3, 22.0; HRMS (ESI^+) calculated for $C_{14}H_{19}O_2$ $[M+H]^+$ = 219.1380, found 219.1380.

(2-(3-Methyl-2-methylenebutyl)phenyl)methanol

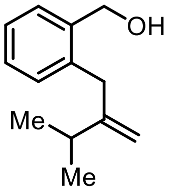

General procedure E: The preceding ester (437 mg, 2.00 mmol) was employed with $LiAlH_4$ (197 mg, 5.20 mmol) in Et_2O at r.t. for 3 h. Purification by flash column chromatography (Hexane/EtOAc = 90/10 to 85/15) afforded the title compound (350 mg, 92%) as a colorless oil. IR (thin film) ν_{max}/cm^{-1}: 3336, 2966, 2859, 1343, 1012; ^1H NMR (500 MHz, C_6D_6) δ_H = 7.38 – 7.33 (m, 1H), 7.14 – 7.09 (m, 2H), 7.09 – 7.05 (m, 1H), 4.83 – 4.80 (m, 1H), 4.47 – 4.42 (m, 3H), 3.28 (s, 2H), 2.15 (hept, J = 6.9 Hz, 1H), 1.17 (br s, 1H), 0.99 (d, J = 6.9 Hz, 6H); ^{13}C NMR (125 MHz, C_6D_6) δ_C = 155.6, 140.1, 137.8, 131.0, 128.4, 128.4, 127.8, 126.8, 108.9, 63.1, 37.9, 34.3, 21.9; HRMS (CI^+) calculated for $C_{13}H_{19}O$ $[M+H]^+$ = 191.1430, found 191.1428.

Benzyl (2-(3-methyl-2-methylenebutyl)benzyl)((perfluorobenzoyl)oxy)carbamate (1p)

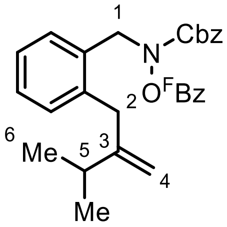

General procedure N: The preceding alcohol (285 mg, 1.50 mmol) was employed with $CbzNHO^FBz$ (650 mg, 1.80 mmol), triphenylphosphine (472 mg, 1.80 mmol) and diisopropyl azodicarboxylate (334 mg, 327 µL, 1.65 mmol). Purification by flash column chromatography (Hexane/EtOAc = 100/0 to 97/3) afforded the title compound (574 mg, 72%) as a colorless solid. m.p. 40 - 42 °C (EtOAc/hexane); IR (thin film) ν_{max}/cm^{-1}: 2966, 2868, 1786, 1499, 1175, 1033, 904; ^1H NMR (400 MHz, $CDCl_3$) δ_H = 7.48 – 7.35 (m, 5H, Cbz ArCH), 7.34 – 7.31 (m, 1H, ArCH), 7.31 – 7.25 (m, 1H, ArCH), 7.24 – 7.15 (m, 2H, ArCH), 5.29 (s, 2H, Cbz CH_2), 4.93 (s, 2H, C**1**-H_2), 4.83 (s, 1H, C**4**-H), 4.30 (s, 1H, C**4**-H'), 3.46 (s, 2H, C**2**-H_2), 2.29 (hept, *J* = 6.8 Hz, 1H, C**5**-H), 1.10 (d, *J* = 6.8 Hz, 6H, 2 × C**6**-H_3); ^{19}F NMR (377 MHz, $CDCl_3$) δ_F = -135.1 – -136.1 (m, 2F), -146.2 (tt, *J* = 20.8, 5.5 Hz, 1F), -159.2 – -160.1 (m, 2F); ^{13}C NMR (101 MHz, $CDCl_3$) δ_C = 155.5 (C=O), 154.6 (C**3**), 138.8 (ArC), 135.2 (Cbz ArC), 132.8 (ArC), 131.1 (ArCH), 129.4 (ArCH), 128.7 (Cbz ArCH), 128.6 (Cbz ArCH), 128.4 (ArCH), 128.3 (Cbz ArCH), 126.6 (ArCH), 108.9 (C**4**), 69.1 (Cbz CH_2), 52.6 (C**1**), 37.8 (C**2**), 34.2 (C**5**), 21.9 (C**6**); HRMS (ESI^+) calculated for $C_{28}H_{24}F_5NNaO_4$ $[M+Na]^+$ = 556.1518, found 556.1518.

The carbon signals corresponding to the pentafluorobenzoyl group could not be resolved due to their weak intensity.

Benzyl 1a-isopropyl-1,1a,3,7b-tetrahydro-2*H*-cyclopropa[*c*]isoquinoline-2-carboxylate (2p)

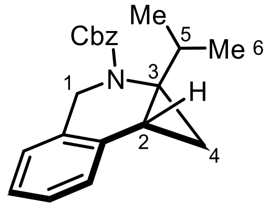

General procedure O: The preceding substrate (53.4 mg, 0.10 mmol) was employed with Pd_2(dba-MeO-*p*)$_3$ (5.48 mg, 0.005 mmol), CgP(2-benzofuryl) (**L3**) (8.31 mg, 0.025 mmol), CsOPiv (23.4 mg, 0.10 mmol), Et_3N (14.0 µL, 0.10 mmol) and dibutyl ether (1.0 mL). The reaction was stirred at 130 °C for 6 h. Purification by flash column chromatography (Hexane/EtOAc = 100/0 to 97/3) afforded the title compound (18.5 mg, 58%) as a colorless oil. IR (thin film) ν_{max}/cm^{-1}: 2962, 2929, 1699, 1494, 1347, 1052, 747; [mixture of rotamers (A:B = 7:3)] ^1H NMR (500 MHz, $CDCl_3$) δ_H = 7.56 – 7.32 (m, 5H, Cbz ArCH, A + B), 7.33 – 7.27 (m, 1H, ArCH, A + B), 7.25 – 7.18 (m, 1H, ArCH, A + B), 7.17 – 6.98 (m, 2H, ArCH, A + B), 5.18 (d, *J* = 12.6 Hz, 0.7H, Cbz CH, A), 5.18 (d, *J* = 12.5 Hz, 0.3H, Cbz CH, B), 5.12 (d, *J* = 12.6 Hz, 0.7H, Cbz CH', A), 5.04 (d, *J* = 12.5 Hz, 0.3H, Cbz CH', B), 4.95 (d, *J* = 15.6 Hz, 0.7H, C**1**-H, A), 4.81 (d, *J* = 15.7 Hz, 0.3H, C**1**-H, B), 4.24 (d, *J* = 15.7 Hz, 0.3H, C**1**-H', B), 4.16 (d, *J* = 15.6 Hz, 0.7H, C**1**-H', A), 2.21 – 1.93 (m, 2H, C**2**-H, A + B and C**5**-H, A + B), 1.52 (dd, *J* = 9.7, 5.4 Hz, 0.3H, C**4**-H, B), 1.45 (dd, *J* = 9.6, 5.5 Hz, 0.7H, C**4**-H, A), 1.11 (d, *J* = 6.8 Hz, 0.9H, C**6**-H_3, B), 1.05 (d, *J* = 6.8 Hz, 2.1H, C**6**-H_3, A), 0.94 (d, *J* = 6.9 Hz, 0.9H, C**6'**-H_3, B), 0.92 – 0.87 (m, 0.3H, C**4**-H', B), 0.90 (d, *J* = 6.9 Hz, 2.1H, C**6'**-H_3, A), 0.84 (dd, *J* = 5.8, 5.5 Hz, 0.7H, C**4**-H', A); ^{13}C

NMR (125 MHz, $CDCl_3$) δ_C = 157.2 (C=O, A), 155.0 (C=O, B), 136.9 (Cbz ArC, A + B), 136.5 (ArC, B), 136.0 (ArC, A), 135.5 (ArC, A), 135.4 (ArC, B), 129.6 (ArCH, B), 129.4 (ArCH, A), 128.6 (Cbz ArCH, B), 128.5 (Cbz ArCH, A), 128.0 (ArCH, B), 127.9 (ArCH, A), 127.9 (Cbz ArCH, A+ B), 127.5 (Cbz ArCH, B), 127.4 (Cbz ArCH, A), 126.0 (ArCH, A), 126.0 (ArCH, A + B), 125.6 (ArCH, B), 67.4 (Cbz CH_2, A), 67.1 (Cbz CH_2, B), 46.6 (C**1**, B), 46.1 (C**1**, A), 44.6 (C**3**, B), 43.8 (C**3**, A), 34.3 (C**5**, A), 33.6 (C**5**, B), 26.0 (C**4**, A), 25.3 (C**4**, B), 20.4 (C**6**, A), 20.3 (C**6**, B), 20.1 (C**2,** B), 20.1 (C**2,** A), 19.0 (C**6',** A + B); HRMS (ESI^+) calculated for $C_{21}H_{23}NNaO_2$ $[M+Na]^+$ = 344.1621, found 344.1623.

Methyl 2-iodo-5-methoxybenzoate

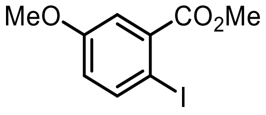

General Procedure G: 2-Iodo-5-methoxybenzoic acid (5.56 g, 20.0 mmol) was employed with oxalyl chloride (5.08 g, 3.43 mL, 40.0 mmol). Purification by flash column chromatography (Hexane/EtOAc = 90/10 to 85/15) afforded the title compound (5.42 g, 93%) as a yellow oil. ^1H NMR (400 MHz, $CDCl_3$) δ_H = 7.82 (d, J = 8.8 Hz, 1H), 7.33 (t, J = 2.4 Hz, 1H), 6.74 (dt, J = 8.8, 2.4 Hz, 1H), 3.92 (s, 3H), 3.80 (s, 3H); ^{13}C NMR (101 MHz, $CDCl_3$) δ_C = 166.8, 159.6, 142.0, 135.9, 119.5, 116.5, 82.5, 55.7, 52.6.

The spectroscopic properties were consistent with the data available in the literature. [25]

Methyl 5-methoxy-2-(2-methylenebutyl)benzoate

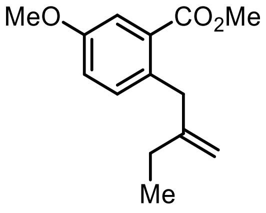

General Procedure H: The preceding iodobenzoate (1.34 g, 4.60 mmol) was employed with magnesium turnings (268 mg, 11.0 mmol), bromobutane (1.26 g, 993 µL, 9.20 mmol), LiCl (468 mg, 11.0 mmol), CuCN (494 mg, 5.52 mmol) and 2-(bromomethyl)but-1-ene (2.74 g, 18.4 mmol). Purification by flash column chromatography (Hexane/EtOAc = 100/0 to 90/10) afforded the title compound (878 mg, 82%) as a colorless oil. IR (thin film) ν_{max}/cm^{-1}: 2964, 2837, 1724, 1434, 1074, 1040, 890; ^1H NMR (500 MHz, $CDCl_3$) δ_H = 7.38 (d, J = 2.9 Hz, 1H), 7.15 (d, J = 8.4 Hz, 1H), 6.98 (dd, J = 8.4, 2.9 Hz, 1H), 4.77 – 4.75 (m, 1H), 4.43 – 4.40 (m, 1H), 3.85 (s, 3H), 3.82 (s, 3H), 3.64 (s, 2H), 2.03 (q, J = 7.4 Hz, 2H), 1.04 (d, J = 7.4 Hz, 3H); ^{13}C NMR (125 MHz, $CDCl_3$) δ_C = ^{13}C NMR (126 MHz, $CDCl_3$) δ 168.2, 157.8, 151.5, 133.2, 132.7, 131.2, 118.2, 115.1, 109.0, 55.6, 52.1, 39.9, 29.2, 12.4; HRMS (ESI^+) calculated for $C_{14}H_{19}O_3$ $[M+H]^+$ = 235.1329, found 235.1328.

(5-Methoxy-2-(2-methylenebutyl)phenyl)methanol

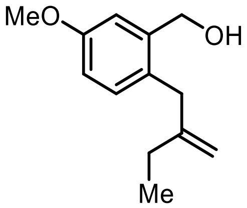

General procedure E: The preceding ester (515 mg, 2.20 mmol) was employed with $LiAlH_4$ (217 mg, 5.72 mmol) in Et_2O at r.t. for 3 h. Purification by flash column chromatography (Hexane/EtOAc = 90/10 to 85/15) afforded the title compound (405 mg, 89%) as a colorless oil. IR (thin film) ν_{max}/cm^{-1}: 3336, 2964, 2934, 1610, 1257, 1158, 890; ^1H NMR (500 MHz, C_6D_6) δ_H = 7.14 – 7.08 (m, 1H), 6.98 (dd, J = 8.3, 2.0 Hz, 1H), 6.80 – 6.71 (m, 1H), 4.87 – 4.76 (m, 1H), 4.65 – 4.54 (m, 1H), 4.51 – 4.39 (m, 2H), 3.48 – 3.34 (m, 3H), 3.21 (s, 2H), 1.91 (q, J = 7.4 Hz, 2H), 0.96 (t, J = 7.4 Hz, 3H); ^{13}C NMR (125 MHz, C_6D_6) δ_C = 159.1, 151.3, 141.4, 113.7, 113.0, 109.6, 63.0, 54.8, 39.1, 29.2, 12.5; HRMS (CI^+) calculated for $C_{13}H_{19}O_2$ $[M+H]^+$ = 207.1380, found 207.1382.

Benzyl (5-methoxy-2-(2-methylenebutyl)benzyl)((perfluorobenzoyl)oxy)carbamate (1q)

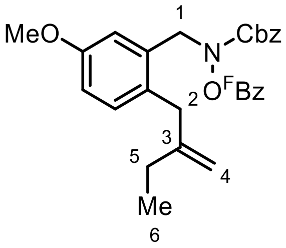

General procedure N: The preceding alcohol (371 mg, 1.80 mmol) was employed with CbzNHO^{F}Bz (780 mg, 2.16 mmol), triphenylphosphine (566 mg, 2.16 mmol) and diisopropyl azodicarboxylate (400 mg, 393 µL, 1.98 mmol). Purification by flash column chromatography (Hexane/EtOAc = 100/0 to 95/5) afforded the title compound (773 mg, 78%) as a colorless solid. m.p. 55 - 57 °C (EtOAc/hexane); IR (thin film) ν_{max}/cm^{-1}: 2968, 2936, 1786, 1501, 1177, 1004; ^1H NMR (400 MHz, $CDCl_3$) δ_H = 7.38 – 7.32 (m, 5H, Cbz ArC<u>H</u>), 7.06 (d, J = 8.3 Hz, 1H, ArC<u>H</u>), 6.91 (s, 1H, ArC<u>H</u>), 6.79 (d, J = 8.3 Hz, 1H, ArC<u>H</u>), 5.27 (s, 2H, Cbz C$\underline{H_2}$), 4.89 (s, 2H, C**1**-$\underline{H_2}$), 4.77 (s, 1H, C**4**-<u>H</u>), 4.42 (s, 1H, C**4**-<u>H</u>’), 3.75 (s, 3H, OC$\underline{H_3}$), 3.33 (s, 2H, C**2**-$\underline{H_2}$), 2.00 (q, J = 7.4 Hz, 2H, C**5**-$\underline{H_2}$), 1.03 (t, J = 7.4 Hz, 3H, C**6**-$\underline{H_3}$); ^{19}F NMR (377 MHz, $CDCl_3$) δ_F = -133.4 – -137.8 (m, 2F), -146.1 (tt, J = 21.0, 5.4 Hz, 1F), -157.3 – -161.3 (m, 2F); ^{13}C NMR (101 MHz, $CDCl_3$) δ_C = 158.3 (Ar<u>C</u>), 155.5 (<u>C</u>=O), 150.3 (C**3**), 135.2 (Cbz Ar<u>C</u>), 134.0 (Ar<u>C</u>), 131.9 (Ar<u>C</u>H), 130.3 (Ar<u>C</u>), 128.7 (Cbz Ar<u>C</u>H), 128.7 (Cbz Ar<u>C</u>H), 128.3 (Cbz Ar<u>C</u>H), 114.3 (Ar<u>C</u>H), 113.8 (Ar<u>C</u>H), 109.6 (C**4**), 69.1 (Cbz <u>C</u>H_2), 55.3 (O<u>C</u>H_3), 52.6 (C**1**), 39.0 (C**2**), 29.0 (C**5**), 12.4 (C**6**); HRMS (ESI^+) calculated for $C_{28}H_{24}F_5NNaO_5$ $[M+Na]^+$ = 572.1467, found 572.1470.

The carbon signals corresponding to the pentafluorobenzoyl group could not be resolved due to their weak intensity.

Benzyl 1a-ethyl-5-methoxy-1,1a,3,7b-tetrahydro-2*H*-cyclopropa[*c*]isoquinoline-2-carboxylate (2q)

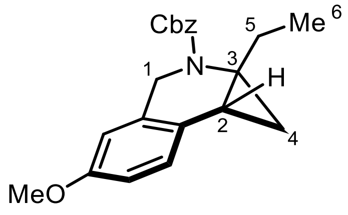

General procedure O: The preceding substrate (55.0 mg, 0.10 mmol) was employed with $Pd_2(dba)_3$ (4.58 mg, 0.005 mmol), CgP(2-benzofuryl) (**L3**) (8.31 mg, 0.025 mmol), CsOPiv (23.4 mg, 0.10 mmol), Et_3N (14.0 µL, 0.10 mmol) and dibutyl ether (1.0 mL). The reaction was stirred at 130 °C for 6 h. Purification by flash column chromatography (Hexane/EtOAc = 100/0 to 95/5) afforded the title compound (22.9 mg, 68%) as a colorless oil. IR (thin film) ν_{max}/cm^{-1}: 2961, 2923, 1699, 1409, 1038, 886; [mixture of rotamers (A:B = 7:3)] ^{1}H NMR (500 MHz, $CDCl_3$) δ_H = 7.44 – 7.27 (m, 5H, Cbz ArCH, A + B), 7.27 – 7.20 (m, 1H, ArCH, A + B), 6.83 – 6.73 (m, 1H, ArCH, A + B), 6.72 – 6.52 (m, 1H, ArCH, A + B), 5.24 – 5.04 (m, 2H, Cbz CH_2, A + B), 4.87 (d, *J* = 15.6 Hz, 0.7 H, C**1**-H, A), 4.74 (d, *J* = 15.6 Hz, 0.3 H, C**1**-H, B), 4.21 (d, *J* = 15.6 Hz, 0.3 H, C**1**-H’, B), 4.13 (d, *J* = 15.6 Hz, 0.7 H, C**1**-H’, A), 3.77 (s, 3H, OCH_3, A + B), 2.53 – 2.40 (m, 0.3H, C**5**-H, B), 2.39 – 2.29 (m, 0.7H, C**5**-H, A), 2.04 – 1.80 (m, 1H, C**2**-H, A + B), 1.39 – 1.27 (m, 1H, C**4**-H, A + B), 1.27 – 1.23 (m, 1H, C**5**-H’, A + B), 1.10 – 0.95 (m, 3H, C**6**-H_3, A + B), 0.89 (dd, *J* = 6.9, 6.9 Hz, 0.3H, C**4**-H’, B), 0.81 (dd, *J* = 5.4, 5.4 Hz, 0.7H, C**4**-H’, A); ^{13}C NMR (125 MHz, $CDCl_3$) δ_C = 157.7 (ArC, A + B), 156.9 (C=O, A), 155.0 (C=O, B), 137.1 (Cbz ArC, A), 136.9 (Cbz ArC, B), 136.4 (ArC, A), 136.4 (ArC, B), 130.5 (ArCH, B), 130.2 (ArCH, A), 128.6 (Cbz ArCH, A+ B), 128.4 (ArCH, B), 128.1 (Cbz ArCH, B), 128.1 (ArCH, A), 128.0 (Cbz ArCH, B), 127.9 (Cbz ArCH, A), 127.7 (Cbz ArCH, A), 113.6 (ArCH, A), 113.3 (ArCH, B), 111.0 (ArCH, B), 110.9 (ArCH, A), 67.3 (Cbz CH_2, A), 67.1 (Cbz CH_2, B), 55.5 (OCH_3, A + B), 46.3 (C**1**, B), 45.9 (C**1**, A), 41.0 (C**3**, B), 40.3 (C**3**, A), 30.2 (C**5**, A), 29.0 (C**5**, B), 27.0 (C**4**, A), 26.3 (C**4**, B), 21.9 (C**2**, A), 21.5 (C**2**, B), 10.6 (C**6**, A + B); HRMS (ESI$^+$) calculated for $C_{21}H_{23}NNaO_3$ $[M+Na]^+$ = 360.1570, found 360.1573.

Methyl 2-iodo-4-methoxybenzoate

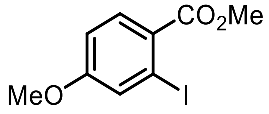

General Procedure G: 2-Amino-4-methoxybenzoic acid (4.18 g, 25.0 mmol) was employed with sodium nitrite (3.45 g, 50.0 mmol), concentrated HCl (~37% w/w, 13.2 mL), potassium iodide (8.30 g, 50.0 mmol) and oxalyl chloride (6.35 g, 4.29 mL, 50.0 mmol). Purification by flash column chromatography (Hexane/EtOAc = 95/5 to 85/15) afforded the title compound (4.56 g, 62%) as a

colorless solid. ^{1}H NMR (400 MHz, $CDCl_3$) δ_H = 7.85 (d, J = 8.8 Hz, 1H), 7.52 (s, 1H), 6.90 (d, J = 8.8 Hz, 1H), 3.88 (s, 3H), 3.82 (s, 3H); ^{13}C NMR (101 MHz, $CDCl_3$) δ_C = 166.1, 162.1, 132.7, 127.1, 126.2, 113.8, 95.8, 55.8, 52.3.

The spectroscopic properties were consistent with the data available in the literature.[26]

Methyl 4-methoxy-2-(2-methylenebutyl)benzoate

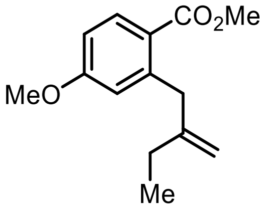

<u>General Procedure H</u>: The preceding iodobenzoate (1.34 g, 4.60 mmol) was employed with magnesium turnings (268 mg, 11.0 mmol), bromobutane (1.26 g, 993 µL, 9.20 mmol), LiCl (468 mg, 11.0 mmol), CuCN (494 mg, 5.52 mmol) and 2-(bromomethyl)but-1-ene (2.74 g, 18.4 mmol). Purification by flash column chromatography (Hexane/EtOAc = 100/0 to 90/10) afforded the title compound (802 mg, 74%) as a colorless oil. IR (thin film) ν_{max}/cm^{-1}: 2965, 2837, 1717, 1571, 1271, 1130, 888; ^{1}H NMR (400 MHz, $CDCl_3$) δ_H = 7.91 (d, J = 9.3 Hz, 1H), 6.83 – 6.73 (m, 2H), 4.79 (s, 1H), 4.46 (s, 1H), 3.83 (s, 3H), 3.83 (s, 3H), 3.75 (s, 2H), 2.07 (q, J = 7.4 Hz, 2H), 1.06 (t, J = 7.4 Hz, 3H); ^{13}C NMR (101 MHz, $CDCl_3$) δ_C = 167.7, 162.3, 151.0, 144.3, 133.1, 122.4, 116.9, 111.4, 109.4, 55.4, 51.8, 40.9, 29.3, 12.4; HRMS (ESI$^+$) calculated for $C_{14}H_{19}O_3$ [M+H]$^+$ = 235.1329, found 235.1330.

(4-Methoxy-2-(2-methylenebutyl)phenyl)methanol

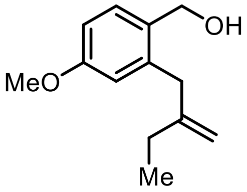

<u>General procedure E</u>: The preceding ester (703 mg, 3.00 mmol) was employed with $LiAlH_4$ (296 mg, 7.80 mmol) in Et_2O at r.t. for 3 h. Purification by flash column chromatography (Hexane/EtOAc = 90/10 to 85/15) afforded the title compound (600 mg, 97%) as a colorless oil. IR (thin film) ν_{max}/cm^{-1}: 3340, 2954, 2944, 1609, 1464, 1158, 890; ^{1}H NMR (500 MHz, C_6D_6) δ_H = 7.26 – 7.19 (m, 1H), 6.86 – 6.79 (m, 1H), 6.69 (dd, J = 8.4, 2.7 Hz, 1H), 4.84 – 4.76 (m, 1H), 4.68 – 4.68 (m, 1H), 4.49 – 4.45 (m, 2H), 3.36 – 3.34 (m, 3H), 3.30 (s, 2H), 1.91 (q, J = 7.4 Hz, 2H), 0.94 (t, J = 7.4 Hz, 3H); ^{13}C NMR (125 MHz, C_6D_6) δ_C = 159.8, 151.0, 139.6, 132.4, 130.2, 116.7, 111.6, 110.0, 62.9, 54.8, 39.9, 29.2, 12.5; HRMS (CI$^+$) calculated for $C_{13}H_{19}O_2$ [M+H]$^+$ = 207.1380, found 207.1384.

Benzyl (4-methoxy-2-(2-methylenebutyl)benzyl)((perfluorobenzoyl)oxy)carbamate (1r)

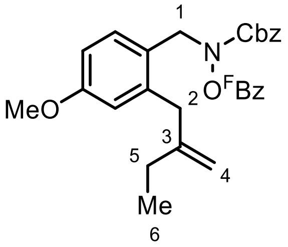

General procedure N: The preceding alcohol (371 mg, 1.80 mmol) was employed with $CbzNHO^FBz$ (780 mg, 2.16 mmol), triphenylphosphine (566 mg, 2.16 mmol) and diisopropyl azodicarboxylate (400 mg, 393 µL, 1.98 mmol). Purification by flash column chromatography (Hexane/EtOAc = 100/0 to 95/5) afforded the title compound (712 mg, 72%) as a colorless solid. m.p. 70 - 72 °C (EtOAc/hexane); IR (thin film) ν_{max}/cm^{-1}: 2967, 2923, 1788, 1524, 1327, 1176, 1006; ^1H NMR (500 MHz, $CDCl_3$) δ_H = 7.38 – 7.31 (m, 5H, Cbz ArCH), 7.19 (d, J = 8.3 Hz, 1H, ArCH), 6.74 – 6.67 (m, 2H, ArCH), 5.26 (s, 2H, Cbz CH_2), 4.85 (s, 2H, C**1**-H_2), 4.81 – 4.79 (m, 1H, C**4**-H), 4.51 – 4.45 (m, 1H, C**4**-H'), 3.78 (s, 3H, OCH_3), 3.41 (s, 2H, C**2**-H_2), 2.01 (q, J = 7.4 Hz, 2H, C**5**-H_2), 1.03 (t, J = 7.4 Hz, 3H, C**6**-H_3); ^{19}F NMR (377 MHz, $CDCl_3$) δ_F = -134.7 – -136.7 (m, 2F), -146.3 (tt, J = 21.0, 5.4 Hz, 1F), -158.8 – -160.4 (m, 2F); ^{13}C NMR (125 MHz, $CDCl_3$) δ_C = 159.7 (ArC), 155.4 (C=O), 149.8 (C**3**), 140.5 (ArC), 135.3 (Cbz ArC), 131.3 (ArCH), 128.7 (Cbz ArCH), 128.6 (Cbz ArCH), 128.3 (Cbz ArCH), 124.7 (ArC), 116.4 (ArCH), 111.5 (ArCH), 110.0 (C**4**), 69.0 (Cbz CH_2), 55.3 (OCH_3), 52.2 (C**1**), 39.8 (C**2**), 29.1 (C**5**), 12.4 (C**6**); HRMS (ESI^+) calculated for $C_{28}H_{24}F_5NNaO_5$ $[M+Na]^+$ = 572.1467, found 572.1462.

The carbon signals corresponding to the pentafluorobenzoyl group could not be resolved due to their weak intensity.

Benzyl 1a-ethyl-6-methoxy-1,1a,3,7b-tetrahydro-2*H*-cyclopropa[*c*]isoquinoline-2-carboxylate (2r)

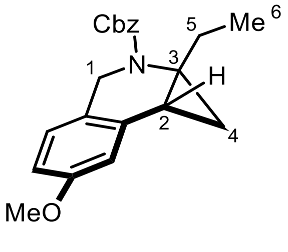

General procedure O: The preceding substrate (55.0 mg, 0.10 mmol) was employed with $Pd_2(dba)_3$ (4.58 mg, 0.005 mmol), CgP(2-benzofuryl) (**L3**) (8.31 mg, 0.025 mmol), CsOPiv (23.4 mg, 0.10 mmol), Et_3N (14.0 µL, 0.10 mmol) and dibutyl ether (1.0 mL). The reaction was stirred at 130 °C for 6 h. Purification by flash column chromatography (Hexane/EtOAc = 100/0 to 95/5) afforded the title compound (27.5 mg, 82%) as a colorless oil. IR (thin film) ν_{max}/cm^{-1}: 2978, 2889, 1700, 1504, 1215, 1038; [mixture of rotamers (A:B = 7:3)] ^1H NMR (500 MHz, $CDCl_3$) δ_H = 7.50 – 7.27 (m, 5H, Cbz ArCH, A + B), 7.11 – 7.97 (m, 1H, ArCH, A + B), 6.96 – 6.84 (m, 1H, ArCH, A + B), 6.74 – 6.61 (m, 1H, ArCH, A + B), 5.24 – 5.06 (m, 2H, Cbz CH_2, A + B), 4.86 (d, J = 15.3 Hz, 0.7H, C**1**-H, A), 4.73

(d, J = 15.4 Hz, 0.3H, C**1**-H, B), 4.17 (d, J = 15.4 Hz, 0.3H, C**1**-H', B), 4.09 (d, J = 15.3 Hz, 0.7H, C**1**-H', A), 3.79 (s, 3H, OCH_3, A + B), 2.54 – 2.41 (m, 0.3H, C**5**-H, B), 2.36 (dq, J = 14.5, 7.4 Hz, 0.7H, C**5**-H, A), 2.07 – 1.96 (m, 1H, C**2**-H, A + B), 1.42 – 1.31 (m, 1H, C**4**-H, A + B), 1.28 – 1.19 (m, 1H, C**5**-H', A + B), 1.09 – 0.97 (m, 3H, C**6**-H_3, A + B), 0.93 – 0.89 (m, C**4**-H', B), 0.86 (dd, J = 5.7, 5.7 Hz, 0.7H, C**4**-H', A); ^{13}C NMR (125 MHz, $CDCl_3$) δ_C = 159.1 (ArC, B), 159.0 (ArC, A), 156.8 (C=O, A), 155.0 (C=O, B), 137.6 (ArC, B), 137.2 (ArC, A), 137.1 (Cbz ArC, A), 137.0 (Cbz ArC, B), 128.6 (Cbz ArCH, A + B), 128.0 (ArCH, A + B), 128.0 (Cbz ArCH, B), 127.9 (Cbz ArCH, A), 127.7 (Cbz ArCH, A), 127.5 (Cbz ArC, B), 126.8 (ArCH, A), 126.5 (ArCH, B), 114.5 (ArCH, A + B), 111.9 (ArCH, A+ B), 67.2 (Cbz CH_2, A), 67.0 (Cbz CH_2, B), 55.4 (OCH_3, A + B), 45.5 (C**1**, B), 45.1 (C**1**, A), 41.0 (C**3**, B), 40.3 (C**3**, A), 30.1 (C**5**, A), 28.9 (C**5**, B), 27.1 (C**4**, A), 26.4 (C**4**, B), 22.9 (C**2**, A), 22.6 (C**2**, B), 10.6 (C**6**, A + B); HRMS (ESI$^+$) calculated for $C_{21}H_{23}NNaO_3$ $[M+Na]^+$ = 360.1570, found 360.1573.

Methyl 2-iodo-4-(trifluoromethyl)benzoate

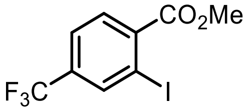

General Procedure G: 2-Iodo-4-(trifluoromethyl)benzoic acid (4.74 g, 15.0 mmol) was employed with oxalyl chloride (3.81 g, 2.57 mL, 30.0 mmol). Purification by flash column chromatography (Hexane/EtOAc = 95/5 to 85/15) afforded the title compound (4.45 g, 90%) as a colorless oil. ^{1}H NMR (400 MHz, $CDCl_3$) δ_H = 8.22 (s, 1H), 7.86 (d, J = 8.1 Hz, 1H), 7.66 (d, J = 8.1 Hz, 1H), 3.96 (s, 3H); ^{19}F NMR (377 MHz, $CDCl_3$) δ_F = -63.2 (s, 3F); ^{13}C NMR (101 MHz, $CDCl_3$) δ_C = 166.3, 138.8, 138.1 (q, $^3J_{CF}$ = 3.9 Hz), 134.1 (q, $^2J_{CF}$ = 33.2 Hz), 131.2, 124.9 (q, $^3J_{CF}$ = 3.7 Hz), 122.5 (q, $^1J_{CF}$ = 273.3 Hz), 93.9, 53.1.

The spectroscopic properties were consistent with the data available in the literature. [27]

Methyl 2-(2-methylenebutyl)-4-(trifluoromethyl)benzoate

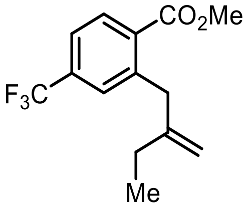

General Procedure H: The preceding iodobenzoate (1.52 g, 4.60 mmol) was employed with magnesium turnings (268 mg, 11.0 mmol), bromobutane (1.26 g, 993 µL, 9.20 mmol), LiCl (468 mg, 11.0 mmol), CuCN (494 mg, 5.52 mmol) and 2-(bromomethyl)but-1-ene (2.74 g, 18.4 mmol). Purification by flash column chromatography (Hexane/EtOAc = 100/0 to 90/10) afforded the title compound (980 mg, 78%) as a yellow oil. IR (thin film) ν_{max}/cm^{-1}: 2968, 1731, 1438, 1261, 1093, 1033, 884; ^{1}H NMR (500 MHz, $CDCl_3$) δ_H = 7.93 (d, J = 7.9 Hz, 1H), 7.57 – 7.47 (m, 2H), 4.86 – 4.81 (m,

1H), 4.46 – 4.36 (m, 1H), 3.89 (s, 3H), 3.75 (s, 2H), 2.05 (q, J = 7.3 Hz, 2H), 1.06 (t, J = 7.3 Hz, 3H); ^{19}F NMR (377 MHz, $CDCl_3$) δ_F = -63.1 (s, 3F); ^{13}C NMR (125 MHz, $CDCl_3$) δ_C = 167.4, 150.2, 142.0, 134.0, 133.3 (q, $^2J_{CF}$ = 32.5 Hz), 130.9, 128.3 (q, $^3J_{CF}$ = 3.7 Hz), 123.8 (q, $^1J_{CF}$ = 272.7 Hz), 123.1 (q, $^3J_{CF}$ = 3.7 Hz), 110.2, 52.5, 40.5, 29.3, 12.4.; HRMS (ESI$^+$) calculated for $C_{14}H_{16}F_3O_2$ $[M+H]^+$ = 273.1097, found 273.1096;

(2-(2-Methylenebutyl)-4-(trifluoromethyl)phenyl)methanol

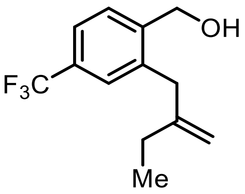

General procedure E: The preceding ester (272 mg, 1.00 mmol) was employed with $LiAlH_4$ (98.7 mg, 2.60 mmol) in Et_2O at r.t. for 3 h. Purification by flash column chromatography (Hexane/EtOAc = 90/10 to 85/15) afforded the title compound (203 mg, 83%) as a colorless oil. IR (thin film) ν_{max}/cm^{-1}: 3338, 2967, 2872, 1332, 1012; ^{1}H NMR (500 MHz, C_6D_6) δ_H = 7.42 – 7.31 (m, 2H), 7.27 (d, J = 7.9 Hz, 1H), 4.76 – 4.65 (m, 1H), 4.41– 4.35 (m, 1H), 4.26 (s, 2H), 3.00 (s, 2H), 1.77 (q, J = 7.4 Hz, 2H), 0.97 (br s, 1H), 0.87 (t, J = 7.4 Hz, 3H); ^{19}F NMR (377 MHz, C_6D_6) δ_F = -62.0 (s, 3F); ^{13}C NMR (125 MHz, C_6D_6) δ_C = 149.5, 144.0, 138.0, 129.7 (q, $^2J_{CF}$ = 31.9 Hz), 138.4, 127.1 (q, $^3J_{CF}$ = 3.7 Hz), 125.2 (q, $^1J_{CF}$ = 272.0 Hz), 123.6 (q, $^3J_{CF}$ = 3.7 Hz), 110.4, 62.1, 39.3, 29.1, 12.3; HRMS (CI$^+$) calculated for $C_{13}H_{15}F_3O$ $[M+H]^+$ = 245.1148, found 245.1153.

Benzyl (4-trifluoromethyl-2-(2-methylenebutyl)benzyl)((perfluorobenzoyl)oxy)carbamate (1s)

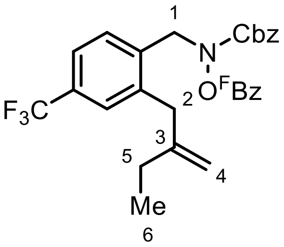

General procedure N: The preceding alcohol (186 mg, 0.76 mmol) was employed with CbzNHO^{F}Bz (329 mg, 0.91 mmol), triphenylphosphine (239 mg, 0.91 mmol) and diisopropyl azodicarboxylate (169 mg, 166 µL, 0.84 mmol). Purification by flash column chromatography (Hexane/EtOAc = 100/0 to 95/5) afforded the title compound (274 mg, 61%) as a colorless solid. m.p. 58 - 60 °C (EtOAc/hexane); IR (thin film) ν_{max}/cm^{-1}: 2968, 2868, 1784, 1503, 1329, 1007; ^{1}H NMR (500 MHz, $CDCl_3$) δ_H = 7.47 – 7.43 (m, 2H, ArC<u>H</u>), 7.42 (s, 1H, ArC<u>H</u>), 7.40 – 7.30 (m, 5H, Cbz ArC<u>H</u>), 5.27 (s, 2H, Cbz C<u>H₂</u>), 4.95 (s, 2H, C**1**-<u>H</u>$_2$), 4.86 – 4.80 (m, 1H, C**4**-<u>H</u>), 4.41 – 4.36 (m, 1H, C**4**-<u>H</u>’), 3.46 (s, 2H, C**2**-<u>H</u>$_2$), 2.03 (q, J = 7.4 Hz, 2H, C**5**-<u>H</u>$_2$), 1.05 (t, J = 7.4 Hz, 3H, C**6**-<u>H</u>$_3$); ^{19}F NMR (377 MHz, $CDCl_3$) δ_F = -62.6 (s, 3F), -133.1 – - 136.9 (m, 2F), -145.7 (tt, J = 20.8, 5.6 Hz, 1F), -156.9 – -161.9 (m, 2F); ^{13}C NMR (125 MHz,

CDCl$_3$) δ_C = 155.4 (C=O), 149.0 (C**3**), 139.4 (ArC), 137.0 (ArC), 135.0 (Cbz ArC), 130.6 (q, $^2J_{CF}$ = 32.4 Hz, ArC), 129.5 (ArCH), 128.8 (Cbz ArCH), 128.7 (Cbz ArCH), 128.4 (Cbz ArCH), 127.6 (q, $^3J_{CF}$ = 3.7 Hz, ArCH), 124.2 (q, $^1J_{CF}$ = 272.1 Hz, CF_3), 123.6 (q, $^3J_{CF}$ = 3.8 Hz, ArCH), 110.6 (C**4**), 69.4 (Cbz CH_2), 52.2 (C**1**), 39.6 (C**2**), 29.2 (C**5**), 12.3 (C**6**); HRMS (ESI$^+$) calculated for $C_{28}H_{21}F_8KNO_4$ $[M+K]^+$ = 626.0974, found 626.0983.

The carbon signals corresponding to the pentafluorobenzoyl group could not be resolved due to their weak intensity.

Benzyl 1a-ethyl-6-(trifluoromethyl)-1,1a,3,7b-tetrahydro-2*H*-cyclopropa[*c*]isoquinoline-2-carboxylate (2s)

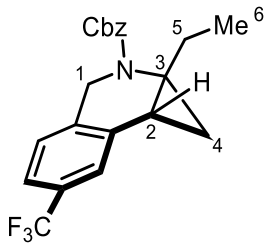

General procedure O: The preceding substrate (58.8 mg, 0.10 mmol) was employed with $Pd_2(dba)_3$ (4.58 mg, 0.005 mmol), CgP(2-benzofuryl) (**L3**) (8.31 mg, 0.025 mmol), CsOPiv (23.4 mg, 0.10 mmol), Et_3N (14.0 µL, 0.10 mmol) and dibutyl ether (1.0 mL). The reaction was stirred at 130 °C for 6 h. Purification by flash column chromatography (Hexane/EtOAc = 100/0 to 95/5) afforded the title compound (29.0 mg, 77%) as a colorless oil. IR (thin film) ν_{max}/cm^{-1}: 2980, 2883, 1702, 1409, 1332, 1272, 911; [mixture of rotamers (A:B = 7:3)] ^{1}H NMR (500 MHz, CDCl$_3$) δ_H = 7.68 – 7.58 (m, 1H, ArCH, A + B), 7.47 – 7.28 (m, 6H, ArCH, A + B and 5 × Cbz ArCH, A + B), 7.25 – 7.08 (m, 1H, ArCH, A + B), 5.23 – 5.04 (m, 2H, Cbz CH_2, A + B), 4.98 (d, *J* = 15.9 Hz, 0.7H, C**1**-H, A), 4.84 (d, *J* = 16.3 Hz, 0.3H, C**1**-H, B), 4.23 (d, *J* = 16.3 Hz, 0.3H, C**1**-H', B), 4.15 (d, *J* = 15.9 Hz, 0.7H, C**1**-H', A), 2.55 – 2.41 (m, 0.3H, C**5**-H, B), 2.36 (dt, *J* = 14.6, 7.3 Hz, 0.7H, C**5**-H, A), 2.13 – 2.02 (m, 1H, C**2**-H, A + B), 1.53 – 1.37 (m, 1H, C**4**-H, A + B), 1.29 – 1.24 (m, 1H, C**5**-H', A + B), 1.09 – 0.98 (m, 3H, C**6**-H_3, A + B), 0.95 – 0.88 (m, 1H, C**4**-H', A + B); ^{19}F NMR (471 MHz, CDCl$_3$) δ_F = -62.5 (s, 3F); ^{13}C NMR (125 MHz, CDCl$_3$) δ_C = 156.7 (C=O, A), 154.9 (C=O, B), 139.3 (ArC, B), 139.1(ArC, A), 137.4 (Cbz ArC, A), 137.0 (Cbz ArC, B), 136.9 (ArC, A), 136.7 (ArC, B), 129.8 (q, $^2J_{CF}$ = 32.1 Hz, ArC, A), 128.6 (Cbz ArCH, A + B), 128.0 (Cbz ArCH, A + B), 127.8 (Cbz ArCH, A + B), 126.4 (ArCH, A), 126.3 (ArCH, A), 126.1 (ArCH, B), 126.1 (ArCH, B), 124.2 (q, $^1J_{CF}$ = 272.1 Hz, CF_3, A + B), 122.8 (q, $^3J_{CF}$ = 3.8 Hz, ArCH, A), 67.5 (Cbz CH_2, A), 67.3 (Cbz CH_2, B), 45.8 (C**1**, B), 45.4 (C**1**, A), 41.4 (C**3**, B), 40.7 (C**3**, A), 30.0 (C**5**, A), 28.7 (C**5**, B), 27.3 (C**4**, A), 26.6 (C**4**, B), 22.4 (C**2**, A), 22.1 (C**2**, B), 10.5 (C**6**, A + B); HRMS (ESI$^+$) calculated for $C_{21}H_{20}F_3NNaO_2$ $[M+Na]^+$ = 398.1338, found 398.1332.

Some carbon signals corresponding to the trifluoromethyl group in rotamer B could not be resolved due to their weak intensity.

Methyl 3-iodo-2-naphthoate

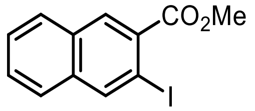

<u>General Procedure G</u>: 3-Amino-2-naphthoic acid (4.68 g, 25.0 mmol) was employed with sodium nitrite (3.45 g, 50.0 mmol), concentrated HCl (~37% w/w, 13.2 mL), potassium iodide (8.30 g, 50.0 mmol) and oxalyl chloride (6.35 g, 4.29 mL, 50.0 mmol). Purification by flash column chromatography (Hexane/EtOAc = 95/5 to 90/10) afforded the title compound (2.61 g, 33%) as a yellow solid. ^{1}H NMR (400 MHz, $CDCl_3$) δ_H = 8.50 (s, 1H), 8.35 (s, 1H), 7.86 (d, J = 7.8 Hz, 1H), 7.74 (d, J = 7.9 Hz, 1H), 7.61 – 7.52 (m, 2H), 3.99 (s, 3H); ^{13}C NMR (101 MHz, $CDCl_3$) δ_C = ^{13}C NMR (101 MHz, $CDCl_3$) δ 167.1, 140.8, 135.9, 131.8, 131.7, 131.3, 128.9, 128.9, 127.5, 126.8, 88.7, 52.7.

The spectroscopic properties were consistent with the data available in the literature.[28]

Methyl 3-(2-methylenebutyl)-2-naphthoate

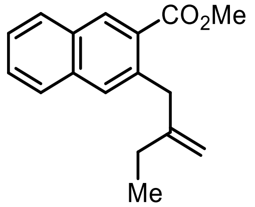

<u>General Procedure H</u>: The preceding iodobenzoate (1.44 g, 4.60 mmol) was employed with magnesium turnings (268 mg, 11.0 mmol), bromobutane (1.26 g, 993 µL, 9.20 mmol), LiCl (468 mg, 11.0 mmol), CuCN (494 mg, 5.52 mmol) and 2-(bromomethyl)but-1-ene (2.74 g, 18.4 mmol). Purification by flash column chromatography (Hexane/EtOAc = 100/0 to 96/4) afforded the title compound (1.05 g, 90%) as a yellow oil. IR (thin film) ν_{max}/cm^{-1}: 2965, 1723, 1430, 1280, 1131, 888; ^{1}H NMR (400 MHz, $CDCl_3$) δ_H = 8.42 (s, 1H), 7.89 (d, J = 8.1 Hz, 1H), 7.80 (d, J = 8.2 Hz, 1H), 7.68 (s, 1H), 7.60 – 7.53 (m, 1H), 7.52 – 7.46 (m, 1H), 4.82 (s, 1H), 4.44 (s, 1H), 3.92 (s, 3H), 3.87 (s, 2H), 2.11 (q, J = 7.4 Hz, 2H), 1.09 (t, J = 7.4 Hz, 3H); ^{13}C NMR (101 MHz, $CDCl_3$) δ_C = 168.6, 151.5, 136.8, 135.0, 131.9, 131.5, 130.2, 129.1, 128.9, 128.3, 127.4, 126.3, 109.6, 52.3, 41.0, 29.5, 12.6; HRMS (ESI$^+$) calculated for $C_{17}H_{19}O_2$ [M+H]$^+$ = 255.1380, found 255.1382.

(3-(2-Methylenebutyl)naphthalen-2-yl)methanol

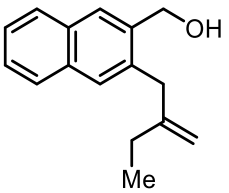

General procedure E: The preceding ester (763 mg, 3.00 mmol) was employed with $LiAlH_4$ (296 mg, 7.80 mmol) in Et_2O at r.t. for 3 h. Purification by flash column chromatography (Hexane/EtOAc = 90/10 to 85/15) afforded the title compound (619 mg, 91%) as a colorless oil. IR (thin film) ν_{max}/cm^{-1}: 3314, 2965, 2925, 1644, 1498, 1037, 888; ^1H NMR (400 MHz, C_6D_6) δ_H = 7.76 (s, 1H), 7.72 – 7.63 (m, 2H), 7.51 (s, 1H), 7.33 – 7.25 (m, 2H), 4.83 (s, 1H), 4.64 – 4.53 (m, 3H), 3.40 (s, 2H), 1.94 (q, *J* = 7.5 Hz, 2H), 1.28 (br s, 1H), 0.97 (t, *J* = 7.5 Hz, 3H); ^{13}C NMR (101 MHz, C_6D_6) δ_C = 150.8, 138.7, 135.8, 133.6, 133.0, 129.3, 128.0, 127.6, 127.0, 126.1, 125.8, 110.2, 63.4, 39.9, 29.4, 12.6; HRMS (CI^+) calculated for $C_{16}H_{19}O$ $[M+H]^+$ = 227.1430, found 227.1433.

Benzyl ((3-(2-methylenebutyl)naphthalen-2-yl)methyl)((perfluorobenzoyl)oxy)carbamate (1t)

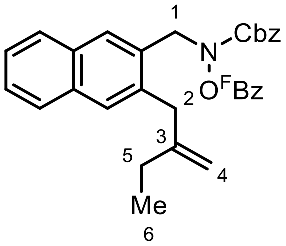

General procedure N: The preceding alcohol (407 mg, 1.80 mmol) was employed with CbzNHO^{F}Bz (780 mg, 2.16 mmol), triphenylphosphine (566 mg, 2.16 mmol) and diisopropyl azodicarboxylate (400 mg, 393 µL, 1.98 mmol). Purification by flash column chromatography (Hexane/EtOAc = 100/0 to 97/3) afforded the title compound (689 mg, 67%) as a colorless solid. m.p. 71 - 73 °C (EtOAc/hexane); IR (thin film) ν_{max}/cm^{-1}: 2966, 2921, 1785, 1326, 902; ^1H NMR (500 MHz, $CDCl_3$) δ_H = 7.78 – 7.74 (m, 3H, ArC<u>H</u>), 7.63 (s, 1H, ArC<u>H</u>), 7.47 – 7.41 (m, 2H, ArC<u>H</u>), 7.38 – 7.32 (m, 5H, Cbz ArC<u>H</u>), 5.29 (s, 2H, Cbz C$\underline{H_2}$), 5.08 (s, 2H, C**1**-$\underline{H_2}$), 4.87 – 4.81 (m, 1H, C**4**-<u>H</u>), 4.48 – 4.44 (m, 1H, C**4**-<u>H</u>’), 3.60 (s, 2H, C**2**-$\underline{H_2}$), 2.08 (q, *J* = 7.4 Hz, 2H, C**5**-$\underline{H_2}$), 1.07 (t, *J* = 7.4 Hz, 3H, C**6**-$\underline{H_3}$); ^{19}F NMR (471 MHz, $CDCl_3$) δ_F = -134.1 – -136.6 (m, 2F), -146.2 (tt, *J* = 20.9, 5.5 Hz, 1F), -157.8 – -161.9 (m, 2F); ^{13}C NMR (125 MHz, $CDCl_3$) δ_C = 155.5 (<u>C</u>=O), 150.2 (C**3**), 136.2 (Ar<u>C</u>), 135.2 (Cbz Ar<u>C</u>), 133.5 (Ar<u>C</u>), 132.2 (Ar<u>C</u>), 131.2 (Ar<u>C</u>), 129.5 (Ar<u>C</u>H), 128.9 (Ar<u>C</u>H), 128.7 (Cbz Ar<u>C</u>H), 128.7 (Cbz Ar<u>C</u>H), 128.3 (Cbz Ar<u>C</u>H), 127.7 (Ar<u>C</u>H), 127.3 (Ar<u>C</u>H), 126.4 (Ar<u>C</u>H), 125.8 (Ar<u>C</u>H), 110.1 (C**4**), 69.2 (Cbz <u>C</u>H_2), 53.2 (C**1**), 39.9 (C**2**), 29.3 (C**5**), 12.4 (C**6**); HRMS (ESI^+) calculated for $C_{31}H_{24}F_5NNaO_4$ $[M+Na]^+$ = 592.1518, found 592.1522.

The carbon signals corresponding to the pentafluorobenzoyl group could not be resolved due to their weak intensity.

Benzyl 1a-ethyl-1,1a,3,9b-tetrahydro-2*H*-benzo[*g*]cyclopropa[*c*]isoquinoline-2-carboxylate (2t)

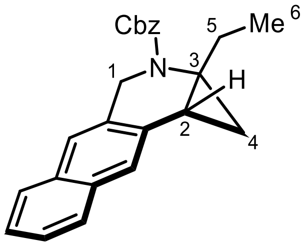

General procedure O: The preceding substrate (57.0 mg, 0.10 mmol) was employed with Pd_2(dba-MeO-*p*)$_3$ (5.48 mg, 0.005 mmol), CgP(2-benzofuryl) (**L3**) (8.31 mg, 0.025 mmol), CsOPiv (23.4 mg, 0.10 mmol), Et_3N (14.0 µL, 0.10 mmol) and dibutyl ether (1.0 mL). The reaction was stirred at 130 °C for 6 h. Purification by flash column chromatography (Hexane/EtOAc = 100/0 to 97/3) afforded the title compound (28.9 mg, 81%) as a colorless oil. IR (thin film) ν_{max}/cm^{-1}: 2982, 1700, 1446, 1353, 1215, 1088, 745; [mixture of rotamers (A:B = 7:3)] ^{1}H NMR (500 MHz, $CDCl_3$) δ_H = 7.91 – 7.83 (m, 1H, ArC<u>H</u>, A + B), 7.81 – 7.70 (m, 2H ArC<u>H</u>, A + B), 7.63 – 7.52 (m, 1H, ArC<u>H</u>, A + B), 7.50 – 7.41 (m, 2H, ArC<u>H</u>, A + B), 7.40 – 7.26 (m, 5H, Cbz ArC<u>H</u>, A + B), 5.29 – 5.04 (m, 2H, Cbz C<u>H</u>$_2$, A + B), 5.12 (d, *J* = 15.3 Hz, 0.7 H, C**1**-<u>H</u>, A), 4.98 (d, *J* = 15.3 Hz, 0.3 H, C**1**-<u>H</u>, B), 4.37 (d, *J* = 15.3 Hz, 0.3 H, C**1**-<u>H</u>', B), 4.29 (d, *J* = 15.3 Hz, 0.7 H, C**1**-<u>H</u>', A), 2.54 – 2.32 (m, 1H, C**5**-<u>H</u>, A + B), 2.31 – 2.20 (m, 1H, C**2**-<u>H</u>, A + B), 1.53 – 1.40 (m, 1H, C**4**-<u>H</u>, A + B), 1.40 – 1.32 (m, 1H, C**5**-<u>H</u>', A + B), 1.16 – 1.02 (m, 3H, C**6**-<u>H</u>$_3$, A + B), 0.98 – 0.90 (m, 1H, C**4**-<u>H</u>', A + B); ^{13}C NMR (125 MHz, $CDCl_3$) δ_C = 156.8 (<u>C</u>=O, A), 155.1 (<u>C</u>=O, B), 137.0 (Cbz Ar<u>C</u>, A), 136.9 (Cbz Ar<u>C</u>, B), 134.6 (Ar<u>C</u>, B), 134.3 (Ar<u>C</u>, A), 133.7 (Ar<u>C</u>, A + B), 133.2 (Ar<u>C</u>, A + B), 131.9 (Ar<u>C</u>, A + B), 128.6 (Cbz Ar<u>C</u>H, A + B), 128.3 (Ar<u>C</u>H, B), 128.1 (Ar<u>C</u>H, A), 127.9 (Cbz Ar<u>C</u>H, A + B), 127.7 (Cbz Ar<u>C</u>H, A + B), 127.7 (Ar<u>C</u>H, A), 127.6 (Ar<u>C</u>H, B), 127.2 (Ar<u>C</u>H, A+ B), 125.8 (Ar<u>C</u>H, A+ B), 125.7 (Ar<u>C</u>H, A+ B), 124.2 (Ar<u>C</u>H, A), 123.8 (Ar<u>C</u>H, B), 67.3 (Cbz <u>C</u>H$_2$, A), 67.1 (Cbz <u>C</u>H$_2$, B), 46.5 (C**1**, B), 46.0 (C**1**, A), 41.7 (C**3**, B), 41.0 (C**3**, A), 30.1 (C**5**, A), 28.9 (C**5**, B), 27.0 (C**4**, A), 26.3 (C**4**, B), 22.4 (C**2**, A), 22.1 (C**2**, B), 10.7 (C**6**, A + B); HRMS (ESI$^+$) calculated for $C_{24}H_{23}NNaO_2$ [M+Na]$^+$ = 380.1621, found 380.1623.

2-(2-Methylenebutyl)benzaldehyde

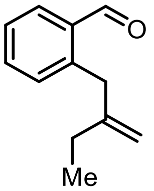

$PdCl_2(PPh_3)_2$ (123 mg, 0.18 mmol) and 1.0 M aq. Na_2CO_3 (14.0 mL, 14.0 mmol) were added to a solution of 2-(bromomethyl)but-1-ene (2.09 g, 14.0 mmol) in THF (35.0 mL) in a two neck round bottom flask. This solution was degassed with N_2. Then a degassed solution of 2-formylphenylboronic acid (525 mg, 3.50 mmol) was added and the resulting mixture was heated at reflux. After 1 h, the

reaction mixture was cooled to rt, and another degassed solution of 2-formylphenylboronic acid (525 mg, 3.50 mmol) was added. After 7 h, the reaction mixture was cooled and quenched with water (21.0 mL) and extracted with DCM (70.0 mL). The combined organic layer was washed with brine, dried over Na_2SO_4, filtered and concentrated in *vacuo*. The residue was purified by flash column chromatography (Hexane/Et_2O = 100/0 to 95/5) to afford the title compound (281 mg, 23%) as a yellow oil. ^{1}H NMR (500 MHz, C_6D_6) δ_H = 10.23 (s, 1H), 7.87 (dd, *J* = 7.6, 1.3 Hz, 1H), 7.52 (td, *J* = 7.6, 1.3 Hz, 1H), 7.39 (t, *J* = 7.4 Hz, 1H), 7.28 – 7.24 (m, 1H), 4.85 (s, 1H), 4.42 (s, 1H), 3.75 (s, 2H), 2.10 (q, *J* = 7.4 Hz, 2H), 1.08 (t, *J* = 7.4 Hz, 3H); ^{13}C NMR (125 MHz, $CDCl_3$) δ_C = 192.2, 151.1, 142.4, 134.5, 133.9, 131.8, 130.5, 127.1, 110.6, 39.0, 29.4, 12.4.

The spectroscopic properties were consistent with the data available in the literature.[29]

1-(2-(2-Methylenebutyl)phenyl)ethan-1-ol

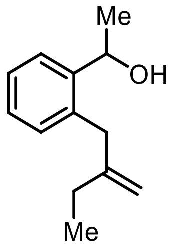

General procedure F: The preceding aldehyde (331 mg, 1.90 mmol) was employed with MeMgBr (3.0 M in Et_2O, 0.95 mL, 2.85 mmol) in THF (5.40 mL) at 0 ºC for 30 min and r.t. for 6 h. Purification by flash column chromatography (Hexane/EtOAc = 95/5 to 85/15) afforded the title compound (316 mg, 88%) as a colorless oil. IR (thin film) ν_{max}/cm^{-1}: 3340, 2968, 2923, 1646, 1368, 894, 757; ^{1}H NMR (400 MHz, C_6D_6) δ_H = 7.57 (d, *J* = 7.7 Hz, 1H), 7.19 – 7.13 (m, 1H), 7.13 – 7.00 (m, 2H), 4.94 (q, *J* = 6.5 Hz, 1H), 4.82 (s, 1H), 4.55 (s, 1H), 3.24 (s, 2H), 1.90 (q, *J* = 7.4 Hz, 2H), 1.35 (d, *J* = 6.5 Hz, 3H), 1.30 (br s, 1H), 0.95 (t, *J* = 7.4 Hz, 3H); ^{13}C NMR (101 MHz, C_6D_6) δ_C = 151.2, 145.1, 136.0, 130.7, 127.3, 127.2, 125.8, 110.1, 66.3, 39.7, 29.3, 24.7, 12.5; HRMS (CI$^+$) calculated for $C_{13}H_{19}O$ [M+H]$^+$ = 191.1430, found 191.1432.

Benzyl (1-(2-(2-methylenebutyl)phenyl)ethyl)((perfluorobenzoyl)oxy)carbamate (1u)

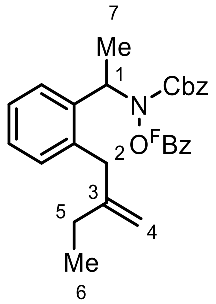

General procedure N: The preceding alcohol (285 mg, 1.50 mmol) was employed with CbzNHO^{F}Bz (650 mg, 1.80 mmol), triphenylphosphine (492 mg, 1.88 mmol) and diisopropyl azodicarboxylate (334 mg, 327 µL, 1.65 mmol). Purification by flash column chromatography (Hexane/EtOAc = 100/0 to 97/3) afforded the title compound (618 mg, 74%) as a colorless solid. m.p. 62 - 64 °C (EtOAc/hexane); IR (thin film) ν_{max}/cm^{-1}: 2968, 2936, 1789, 1524, 1326, 1175, 907; ^{1}H NMR (500 MHz, $CDCl_3$) δ_H = 7.57 – 7.27 (m, 5H, Cbz ArCH), 7.26 – 7.20 (m, 2H, ArCH), 7.20 – 7.01 (m, 2H, ArCH), 5.94 – 5.57 (m, 1H, C**1**-H), 5.46 – 5.00 (m, 2H, Cbz CH_2), 4.88 – 4.70 (m, 1H, C**4**-H), 4.52 – 4.33 (m, 1H, C**4**-H’), 3.84 – 3.56 (m, 1H, C**2**-H), 3.43 – 3.15 (m, 1H, C**2**-H’), 2.21 – 1.86 (m, 2H, C**5**-H_2), 1.64 – 1.51 (m, 3H, C**7**-H_3), 1.16 – 0.92 (m, 3H, C**6**-H_3); [mixture of rotamers (A:B = 2:1)] ^{19}F NMR (377 MHz, $CDCl_3$) rotamer A δ_F = -134.9 – -135.6 (m, 2F), -146.4 – -147.0 (m, 1F), -159.5 – -160.1 (m, 2F); rotamer B δ_F = -135.7– -136.1 (m, 2F), -145.9 – -146.4 (m, 1F), -158.9 – -159.5 (m, 2F); ^{13}C NMR (125 MHz, $CDCl_3$) δ_C = 155.2 (C=O), 150.7 (C**3**), 139.9 (ArC), 135.3 (Cbz ArC), 131.0 (ArC), 131.0 (ArCH), 128.7 (ArCH), 128.7 (Cbz ArCH), 128.7 (Cbz ArCH), 128.6 (ArCH), 128.2 (Cbz ArCH), 126.9 (ArCH), 109.6 (C**4**), 68.9 (Cbz CH_2), 55.3 (C**1**), 39.6 (C**2**), 29.3 (C**5**), 15.4 (C**7**), 12.4 (C**6**); HRMS (ESI$^+$) calculated for $C_{28}H_{24}F_5NNaO_4$ [M+Na]$^+$ = 556.1518, found 556.1514.

The carbon signals corresponding to the pentafluorobenzoyl group could not be resolved due to their weak intensity.

Some signal broadening and complexity was observed in ^{13}C NMR spectrum due to amide-like resonance and the pentafluorobenzoyl group effect.

Benzyl 1a-ethyl-3-methyl-1,1a,3,7b-tetrahydro-2*H*-cyclopropa[*c*]isoquinoline-2-carboxylate (2u)

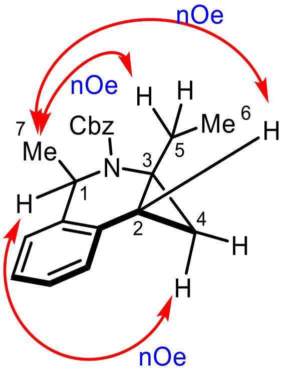

General procedure O: The preceding substrate (53.4 mg, 0.10 mmol) was employed with Pd_2(dba-MeO-*p*)$_3$ (5.48 mg, 0.005 mmol), CgP(2-benzofuryl) (**L3**) (16.6 mg, 0.050 mmol), CsOPiv (23.4 mg, 0.10 mmol), Et_3N (14.0 µL, 0.10 mmol) and dibutyl ether (1.0 mL). The reaction was stirred at 130 °C for 6 h. Purification by flash column chromatography (Hexane/EtOAc = 100/0 to 97/3) afforded the title compound (18.8 mg, 59%) as a colorless oil. IR (thin film) ν_{max}/cm^{-1}: 2981, 2921, 1708, 1413, 1293, 1137, 754; ^{1}H NMR (500 MHz, $CDCl_3$) δ_H = 7.38 – 7.30 (m, 5H, Cbz ArCH), 7.30 – 7.27 (m, 1H, ArCH), 7.23 (m, 2H, ArCH), 7.19 – 7.15 (m, 1H, ArCH), 5.06 (d, J = 12.6 Hz, 1H, Cbz CH), 5.02 (d,

J = 12.6 Hz, 1H, Cbz C<u>H</u>'), 4.56 (q, J = 7.1 Hz, 1H, C**1**-<u>H</u>), 2.11 – 2.05 (m, 1H, C**5**-<u>H</u>), 2.05 – 2.01 (m, 1H, C**2**-<u>H</u>), 1.98 – 1.75 (m, 3H, C**7**-<u>H</u>$_3$), 1.67 – 1.55 (m, 1H, C**5**-<u>H</u>'), 1.30 (dd, J = 9.4, 5.5 Hz, 1H, C**4**-<u>H</u>), 1.06 (t, J = 7.5 Hz, 3H, C**6**-<u>H</u>$_3$), 0.82 (dd, J = 5.6, 5.5 Hz, 1H, C**4**-<u>H</u>'); ^{13}C NMR (125 MHz, $CDCl_3$) δ_C = 156.2 (<u>C</u>=O), 140.8 (Ar<u>C</u>), 137.9 (Ar<u>C</u>), 137.1 (Cbz Ar<u>C</u>), 129.3 (Ar<u>C</u>H), 128.5 (Cbz Ar<u>C</u>H), 127.8 (Cbz Ar<u>C</u>H), 127.8 (Cbz Ar<u>C</u>H), 127.3 (Ar<u>C</u>H), 126.0 (Ar<u>C</u>H), 123.2 (Ar<u>C</u>H), 66.5 (Cbz <u>C</u>H$_2$), 53.5 (C**1**), 45.2 (C**3**), 30.2 (C**5**), 26.0 (C**4**), 22.9 (C**2**), 16.6 (C**7**), 10.4 (C**6**); HRMS (ESI$^+$) calculated for $C_{21}H_{23}NNaO_2$ $[M+Na]^+$ = 344.1621, found 344.1622.

Some signal broadening was observed due to amide-like resonance resulting in weak signal intensities in ^{13}C NMR spectrum.

The relative stereochemistry was assigned on the basis of the nOe enhancements outlined above.

1-(Trimethylsilyl)propan-2-one

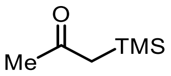

<u>**General Procedure I:**</u> $TMSCH_2MgCl$ (1.0 M in Et_2O, 44.0 mL, 44.0 mmol) was employed with acyl chloride (3.14 g, 40.0 mmol) and copper iodide (8.38 g, 44.0 mmol). 1-(trimethylsilyl)-propan-2-one (4.27 g, 82 %) was afforded as a colorless oil which was used without any further purification. ^{1}H NMR (500 MHz, C_6D_6) δ_H = 1.91 (s, 2H), 1.76 (s, 3H), -0.05 (s, 9H); ^{13}C NMR (125 MHz, C_6D_6) δ_C = 205.1, 38.5, 31.5, -1.3.

The spectroscopic properties were consistent with the data available in the literature.[30]

3-(2-Hydroxy-1-(trimethylsilyl)propan-2-yl)dihydrofuran-2(3*H*)-one

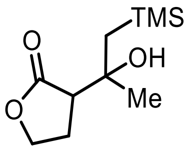

<u>**General Procedure J:**</u> Butyrolactone (2.07 g, 24.0 mmol) was employed with 1.0 M LiHMDS in THF (26.4 ml, 26.4 mmol) and 1-(trimethylsilyl)-propan-2-one (4.38 g, 33.6 mmol). Purification by flash column chromatography (Hexane/Et_2O = 80/20 to 50/50) afforded the title compounds (2.71 g, 70%) as a mixture of diastereomers (1.4:1) and colorless oils. IR (thin film) ν_{max}/cm^{-1}: 3496, 2950, 1747, 1376, 1247, 1183; *Diastereomer 1:* ^{1}H NMR (500 MHz, C_6D_6) δ_H = 3.85 (br s, 1H), 3.55 – 3.45 (m, 1H), 3.27 – 3.20 (m, 1H), 2.19 (dd, J = 11.2, 9.4 Hz, 1H), 1.33 – 1.21 (m, 2H), 1.07 (s, 3H), 0.78 – 0.70 (m, 2H), 0.18 (s, 9H); ^{1}H NMR (500 MHz, C_6D_6) δ_C = 178.7, 73.2, 65.5, 51.5, 30.9, 26.0, 25.5, 0.8; *Diastereomer 2:* ^{1}H NMR (500 MHz, C_6D_6) δ_H = 4.00 (br s, 1H), 3.55 – 3.45 (m, 1H), 3.27 – 3.20 (m, 1H), 2.10 (dd, J = 11.4, 9.2 Hz, 1H), 1.33 – 1.21 (m, 2H), 1.11 (s, 3H), 0.85 (d, J = 14.9 Hz, 1H), 0.64

(d, J = 14.9 Hz, 1H), 0.15 (s, 9H); ^{1}H NMR (500 MHz, C_6D_6) δ_C = 178.9, 73.2, 65.7, 52.0, 28.6, 27.2, 25.3, 0.7; HRMS (ESI$^+$) calculated for $C_{10}H_{20}NaO_3Si$ $[M+Na]^+$ = 239.1074, found 239.1074.

3-(Prop-1-en-2-yl)dihydrofuran-2(3*H*)-one

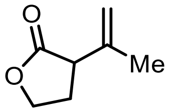

General Procedure K: The preceding lactone (2.70 g, 12.5 mmol) was employed with HF-pyridine (12.5 ml) in THF (150 mL). Purification by flash column chromatography (Pentane/Et_2O = 75/25 to 60/40) afforded the title compound (1.33 g, 84%) as a colorless oil. IR (thin film) ν_{max}/cm^{-1}: 3083, 2977, 1763, 1650, 1372, 1149; ^{1}H NMR (500 MHz, $CDCl_3$) δ_H = 5.02 – 4.98 (m, 1H), 4.96 – 4.92 (m, 1H), 4.36 (ddd, J = 8.6, 8.4, 4.1 Hz, 1H), 4.25 (ddd, J = 8.6, 8.4, 7.0 Hz, 1H), 3.24 (dd, J = 9.2, 9.1 Hz, 1H), 2.40 (dddd, J = 13.0, 9.1, 7.0, 4.1 Hz, 1H), 2.27 (dddd, J = 13.0, 9.2, 8.4, 8.4 Hz, 1H), 1.85 – 1.78 (m, 3H); ^{13}C NMR (125 MHz, $CDCl_3$) δ_C = 177.0, 139.8, 114.8, 66.6, 47.1, 28.0, 20.3; HRMS (ESI$^+$) calculated for $C_7H_{11}O_2$ $[M+H]^+$ = 127.0754, found 127.0748.

3-(2-Hydroxyethyl)-4-methylpent-4-en-2-one

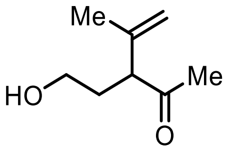

General Procedure L: The preceding lactone (1.39 g, 11.0 mmol) was employed with MeMgBr (3.0 M in Et_2O, 29.3 mL, 88.0 mmol), HN(OMe)Me·HCl (1.29 g, 13.2 mmol) and NaOMe (119 mg, 2.20 mmol). The residue was purified quickly by flash column chromatography (Hexane/Et_2O = 50/50 to 25/75) giving a crude product which was taken through to the next step without further purification.

Benzyl (3-acetyl-4-methylpent-4-en-1-yl)((perfluorobenzoyl)oxy)carbamate (3a)

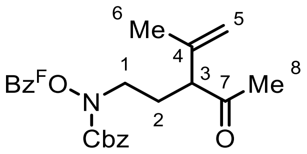

General procedure N: The preceding crude alcohol (625 mg, 4.00 mmol) was employed with CbzNHO^{F}Bz (1.45 g, 4.00 mmol), triphenylphosphine (1.15 g, 4.40 mmol) and diisopropyl azodicarboxylate (890 mg, 866 µL, 4.40 mmol). Purification by flash column chromatography (Toluene/EtOAc = 100/0 to 98/2) afforded the title compound (990 mg, 51% over 2 steps) as a colorless oil. IR (thin film) ν_{max}/cm^{-1}: 2970, 1785, 1715, 1525, 1501, 1177; ^{1}H NMR (500 MHz, $CDCl_3$) δ_H = 7.41 – 7.31 (m, 5H, Cbz ArC<u>H</u>), 5.21 (s, 2H, Cbz C<u>H$_2$</u>), 5.01 – 4.98 (m, 1H, C**5**-<u>H</u>), 4.95 – 4.92 (m, 1H, C**5**-

H'), 3.76 – 3.63 (m, 2H, C**1**-H$_2$), 3.34 (dd, *J* = 7.2, 7.2 Hz, 1H, C**3**-H), 2.17 – 2.08 (m, 1H, C**2**-H), 2.10 (s, 3H, C**8**-H$_3$), 1.81 – 1.73 (m, 1H, C**2**-H'), 1.65 – 1.62 (m, 3H, C**6**-H$_3$); ^{19}F NMR (471 MHz, $CDCl_3$) δ_F = -134.4 – -136.1 (m, 2F), -145.8 (tt, *J* = 21.1, 5.4 Hz, 1F), -158.9 – -159.4 (m, 2F); ^{13}C NMR (125 MHz, $CDCl_3$) δ_C = 207.9 (C**7**), 155.5 (Cbz C=O), 141.8 (C**4**), 135.2 (Cbz ArC), 128.7 (Cbz ArCH), 128.7 (Cbz ArCH), 128.3 (Cbz ArCH), 116.3 (C**5**), 69.0 (Cbz CH$_2$), 57.9 (C**3**), 49.4 (C**1**), 28.4 (C**8**), 25.8 (C**2**), 19.8 (C**6**); HRMS (ESI$^+$) calculated for $C_{23}H_{20}F_5NNaO_5$ [M+Na]$^+$ = 508.1154, found 508.1141.

The carbon signals corresponding to the pentafluorobenzoyl group could not be resolved due to their weak intensity.

Benzyl 4-acetyl-6-methyl-3,4-dihydropyridine-1(2*H*)-carboxylate and benzyl 4-acetyl-6-methyl-3,6-dihydropyridine-1(2*H*)-carboxylate (4a)

General procedure O: The preceding substrate (48.5 mg, 0.10 mmol) was employed with $Pd_2(dba)_3$ (2.29 mg, 0.0025 mmol), CgP(2-benzofuryl) (**L3**) (4.99 mg, 0.015 mmol), CsOAc (19.2 mg, 0.10 mmol), Et_3N (14.0 µL, 0.10 mmol) and dibutyl ether (1.0 mL). The reaction was stirred at 110 °C for 16 h. Purification by flash column chromatography (Hexane/EtOAc = 95/5 to 85/15) afforded the title compounds (benzyl 4-acetyl-6-methyl-3,4-dihydropyridine-1(2*H*)-carboxylate: 19.1 mg, 70%; benzyl 4-acetyl-6-methyl-3,6-dihydropyridine-1(2*H*)-carboxylate: 3.00 mg, 11%) as colorless oils.

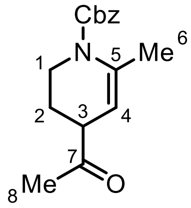

IR (thin film) ν_{max}/cm^{-1}: 2970, 1785, 1525, 1501, 1177; ^{1}H NMR (500 MHz, $CDCl_3$) δ_H = 7.38 – 7.32 (m, 5H, Cbz ArCH), 5.15 (s, 2H, Cbz CH$_2$), 4.96 (d, *J* = 3.3 Hz, 1H, C**4**-H), 3.71 (ddd, *J* = 12.8, 7.5, 3.2 Hz, 1H, C**1**-H), 3.57 (ddd, *J* = 12.8, 8.3, 3.1 Hz, 1H, C**1**-H'), 3.15 – 3.09 (m, 1H, C**3**-H), 2.18 (s, 3H, C**8**-H$_3$), 2.14 (s, 3H, C**6**-H$_3$), 2.11 – 2.05 (m, 1H, C**2**-H), 1.82 (dddd, *J* = 13.6, 8.3, 7.0, 3.2 Hz, 1H, C**2**-H'); ^{13}C NMR (125 MHz $CDCl_3$) δ_C = 208.1 (C**7**), 154.1 (Cbz C=O), 138.0 (C**5**), 136.3 (Cbz ArC), 128.7 (Cbz ArCH), 128.3 (Cbz ArCH), 128.3 (Cbz ArCH), 107.3 (C**4**), 67.7 (Cbz CH$_2$), 47.5 (C**3**), 43.5 (C**1**), 28.0 (C**8**), 24.5 (C**2**), 23.0 (C**6**); HRMS (ESI$^+$) calculated for $C_{16}H_{20}NO_3$ [M+Na]$^+$ = 274.1438, found 274.1427.

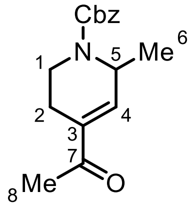

IR (thin film) ν_{max}/cm^{-1}: 2932, 1698, 1671, 1423; ^{1}H NMR (500 MHz, DMSO-d^6, 100°C) δ_H = 7.38 – 7.32 (m, 5H, Cbz ArC<u>H</u>), 6.87 – 6.82 (m, 1H, C**4**-<u>H</u>), 5.15 (d, *J* = 12.5 Hz, 1H, Cbz C<u>H</u>), 5.11 (d, *J* = 12.7 Hz, 1H, Cbz C<u>H</u>'), 4.71 – 4.64 (m, 1H, C**5**-<u>H</u>), 4.12 – 4.05 (m, 1H, C**1**-<u>H</u>), 2.88 (ddd, *J* = 12.5, 12.5, 3.9 Hz, 1H, C**1**-<u>H</u>'), 2.42 – 2.35 (m, 1H, C**2**-<u>H</u>), 2.26 (s, 3H, C**8**-$\underline{H_3}$), 2.09 – 2.03 (m, 1H, C**2**-<u>H</u>'), 1.26 (d, *J* = 6.8 Hz, 3H, C**6**-$\underline{H_3}$); ^{13}C NMR (125 MHz, DMSO-d^6, 100°C) δ_C = 196.9 (C**7**), 153.6 (Cbz <u>C</u>=O), 140.0 (C**4**), 136.5 (Cbz Ar<u>C</u>), 135.8 (C**3**), 127.9 (Cbz Ar<u>C</u>H), 127.3 (Cbz Ar<u>C</u>H), 127.0 (Cbz Ar<u>C</u>H), 65.9 (Cbz $\underline{C}H_2$), 47.5 (C**5**), 35.9 (C**1**), 24.6 (C**8**), 22.5 (C**2**), 17.4 (C**6**); HRMS (ESI$^+$) calculated for $C_{16}H_{20}NO_3$ [M+Na]$^+$ = 274.1438, found 274.1424.

Methyl 2-(2-((*tert*-butyldimethylsilyl)oxy)ethyl)-3-methylbut-3-enoate

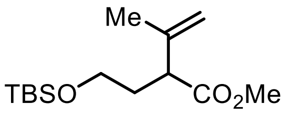

A solution of methyl senecioate (571 mg, 607 µL, 5.00 mmol) in THF (2.50 mL) was added to a stirring solution of LDA (1.0 M in THF, 6.00 mL, 6.00 mmol) in THF (5.0 mL) at −78 °C, and the reaction was stirred for 1 h at −78 °C. The reaction was allowed to warm to 0 °C over 1 h, and *tert*-butyl(2-iodoethoxy)dimethylsilane (2.00 g, 7.00 mmol) was added dropwise. The mixture was warmed to room temperature over 1 h, and stirred at room temperature for 2 h, before being poured into water (10 mL) and extracted with Et_2O (3 × 10 mL). The combined organic layer was washed with brine, dried over Na_2SO_4, filtered and concentrated in *vacuo*. The residue was purified by flash column chromatography (Hexane/EtOAc = 100/0 to 95/5) to afford the title compound (1.0 g, 73%) as a colorless oil. IR (thin film) ν_{max}/cm^{-1}: 2954, 2930, 1737, 1435, 1254, 834; ^{1}H NMR (4500 MHz, $CDCl_3$) δ_H = 4.93 – 4.89 (m, 1H), 4.89 – 4.86 (m, 1H), 3.67 (s, 3H), 3.58 (dd, *J* = 6.2, 6.2 Hz, 2H), 3.27 (dd, *J* = 7.6, 7.6 Hz, 1H), 2.05 (ddt, *J* = 14.1, 7.6, 6.2 Hz, 1H), 1.80 – 1.71 (m, 1H), 1.74 (s, 3H), 0.88 (s, 9H), 0.02 (s, 6H); ^{13}C NMR (101 MHz $CDCl_3$) δ_C = 174.2, 142.4, 114.0, 60.7, 51.9, 49.3, 33.2, 26.0, 20.4, 18.4, -5.3, -5.3; HRMS (ESI$^+$) calculated for $C_{14}H_{28}O_3NaSi$ [M+Na]$^+$ = 295.1700, found 295.1704.

3-(2-((*tert*-Butyldimethylsilyl)oxy)ethyl)-2-methyloct-1-en-4-one

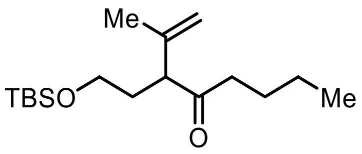

<u>**General Procedure L**</u>**:** Methyl 2-(2-((*tert*-butyldimethylsilyl)oxy)ethyl)-3-methylbut-3-enoate (681 mg, 2.50 mmol) was employed with ^{n}BuMgBr (20 mmol), HN(OMe)Me·HCl (293 mg, 3.00 mmol) and NaOMe (33.8 mg, 0.63 mmol). Purification by flash column chromatography (Hexane/EtOAc = 100/0 to 95/5) afforded the title compound (468 mg, 63%) as a colorless oil. IR (thin film) ν_{max}/cm^{-1}: 2956, 2930, 1714, 1471, 1254, 1099; ^{1}H NMR (500 MHz, $CDCl_3$) δ_H = 4.97 – 4.91 (m, 1H), 4.90 – 4.85 (m,

1H), 3.52 (dd, J = 6.3, 6.3 Hz, 2H), 3.38 (dd, J = 7.1, 7.1 Hz, 1H), 2.51 (ddd, J = 16.9, 7.9, 7.1 Hz, 1H), 2.37 (ddd, J = 16.9, 7.9, 6.3 Hz, 1H), 2.04 – 1,96 (m, 1H), 1.70 – 1.61 (m, 1H), 1.66 – 1.63 (m, 3H), 1.56 – 1.46 (m, 2H), 1.34 – 1.22 (m, 2H), 0.88 (t, J = 7.3 Hz, 3H), 0.87 (s, 9H), 0.01 (s, 6H); ^{13}C NMR (125 MHz CDCl$_3$) δ_C = 210.8, 142.9, 114.9, 60.8, 56.7, 41.0, 32.0, 26.1, 26.0, 22.4, 20.2, 18.4, 14.0, -5.2, -5.3; HRMS (ESI$^+$) calculated for $C_{17}H_{34}NaO_2Si$ [M+Na]$^+$ = 321.2220, found 321.2221.

3-(2-Hydroxyethyl)-2-methyloct-1-en-4-one

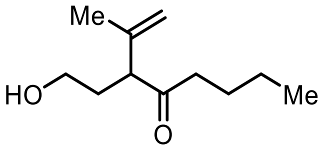

General Procedure M: The preceding silyl ether (367 mg, 1.23 mmol) was employed with TBAF (1.0 M in THF, 1.60 mL, 1.60 mmol). Purification by flash column chromatography (Hexane/EtOAc = 90/10 to 85/15) afforded the title compound (206 mg, 91%) as a colorless oil. IR (thin film) ν_{max}/cm^{-1}: 3416, 2958, 2933, 1710, 1378, 1128, 897; ^{1}H NMR (500 MHz, CDCl$_3$) δ_H = 4.98 – 4.93 (m, 1H), 4.91 (s, 1H), 3.62 – 3.51 (m, 2H), 3.36 (dd, J = 7.1, 7.1 Hz, 1H), 2.51 (ddd, J = 17.1, 7.8, 7.8 Hz, 1H), 2.38 (ddd, J = 17.1, 8.1, 6.6 Hz, 1H), 2.08 – 2.00 (m, 1H), 1.86 (br s, 1H), 1.74 – 1.67 (m, 1H), 1.64 (s, 3H), 1.54 – 1.45 (m, 2H), 1.31 – 1.22 (m, 2H), 0.86 (t, J = 7.3 Hz, 3H); ^{13}C NMR (125 MHz CDCl$_3$) δ_C = 211.3, 142.8, 115.2, 60.9, 57.4, 40.9, 31.7, 26.0, 22.4, 20.1, 14.0; HRMS (ESI$^+$) calculated for $C_{11}H_{20}NaO_2$ [M+Na]$^+$ = 207.1355, found 207.1354.

Benzyl (4-oxo-3-(prop-1-en-2-yl)octyl)((perfluorobenzoyl)oxy)carbamate (3b)

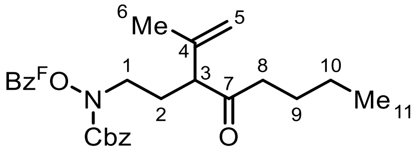

General procedure N: The preceding alcohol (203 mg, 1.10 mmol) was employed with CbzNHO^{F}Bz (361 mg, 1.00 mmol), triphenylphosphine (328 mg, 1.25 mmol) and diisopropyl azodicarboxylate (243 mg, 238 μL, 1.20 mmol). Purification by flash column chromatography (Hexane/EtOAc = 90/10 to 85/15) afforded the title compound (345 mg, 66%) as a colorless oil. IR (thin film) ν_{max}/cm^{-1}: 2967, 1786, 1730, 1500, 1172; ^{1}H NMR (500 MHz, CDCl$_3$) δ_H = 7.38 – 7.30 (m, 5H, Cbz ArC<u>H</u>), 5.21 (s, 2H, Cbz C<u>H$_2$</u>), 4.98 – 4.95 (m, 1H, C**5**-<u>H</u>), 4.93 – 4.90 (m, 1H, C**5**-<u>H</u>’), 3.76 – 3.62 (m, 2H, C**1**-<u>H$_2$</u>), 3.35 (dd, J = 7.2, 7.2 Hz, 1H, C**3**-<u>H</u>), 2.50 (ddd, J = 17.1, 8.0, 7.1 Hz, 1H, C**8**-<u>H</u>), 2.33 (ddd, J = 17.1, 7.9, 6.5 Hz, 1H, C**8**-<u>H</u>’), 2.11 (m, 1H, C**2**-<u>H</u>), 1.82 – 1.73 (m, 1H, C**2**-<u>H</u>’), 1.62 (s, 3H, C**6**-<u>H$_3$</u>), 1.53 – 1.45 (m, 2H, C**9**-<u>H$_2$</u>), 1.30 – 1.19 (m, 2H, C**10**-<u>H$_2$</u>), 0.87 (t, J = 7.4 Hz, 3H, C**11**-<u>H$_3$</u>); ^{19}F NMR (471 MHz, CDCl$_3$) δ_F = -127.5 – -138.7 (m, 2F), -145.9 (tt, J = 20.9, 5.3 Hz, 1F), -158.5 – - 160.4 (m, 2F); ^{13}C NMR (125 MHz, CDCl$_3$) δ_C = 210.0 (C**7**), 155.5 (Cbz <u>C</u>=O), 142.0 (C**4**), 135.2 (Cbz ArC), 128.7 (Cbz

ArCH), 128.7 (Cbz ArCH), 128.3 (Cbz ArCH), 116.1 (C**5**), 69.0 (Cbz C̲H$_2$), 57.1 (C**3**), 49.4 (C**1**), 40.9 (C**8**), 26.0 (C**2**), 25.9 (C**9**), 22.4 (C**10**), 19.8 (C**6**), 14.0 (C**11**); HRMS (ESI$^+$) calculated for $C_{26}H_{26}F_5NNaO_5$ [M+Na]$^+$ = 550.1623, found 550.1627.

The carbon signals corresponding to the pentafluorobenzoyl group could not be resolved due to their weak intensity.

Benzyl 6-methyl-4-pentanoyl-3,4-dihydropyridine-1(2*H*)-carboxylate and benzyl 6-methyl-4-pentanoyl-3,6-dihydropyridine-1(2*H*)-carboxylate (4b)

General procedure O: The preceding substrate (52.8 mg, 0.10 mmol) was employed with $Pd_2(dba)_3$ (2.29 mg, 0.0025 mmol), CgP(2-benzofuryl) (**L3**) (4.99 mg, 0.015 mmol), CsOAc (19.2 mg, 0.10 mmol), Et_3N (14.0 μL, 0.10 mmol) and dibutyl ether (1.0 mL). The reaction was stirred at 110 °C for 16 h. Purification by flash column chromatography (Hexane/EtOAc = 95/5 to 85/15) afforded the title compounds (benzyl 6-methyl-4-pentanoyl-3,4-dihydropyridine-1(2*H*)-carboxylate: 26.0 mg, 82%; benzyl 6-methyl-4-pentanoyl-3,6-dihydropyridine-1(2*H*)-carboxylate: 4.00 mg, 13%) as colorless oils.

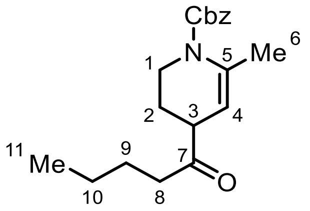

IR (thin film) ν_{max}/cm^{-1}: 2988, 2974, 2901, 1719, 1405, 1394, 1074, 891; ^{1}H NMR (500 MHz, $CDCl_3$) δ_H = 7.47 – 7.27 (m, 5H, Cbz ArCH̲), 5.15 (s, 2H, Cbz CH̲$_2$), 4.93 (dd, *J* = 3.8, 1.3 Hz, 1H, C**4**-H̲), 3.71 (ddd, *J* = 12.9, 7.5, 3.3 Hz, 1H, C**1**-H̲), 3.58 (ddd, *J* = 12.9, 8.5, 3.2 Hz, 1H, C**1**-H̲'), 3.19 – 2.99 (m, 1H, C**3**-H̲), 2.55 – 2.39 (m, 2H, C**8**-H̲$_2$), 2.16 – 2.12 (m, 3H, C**6**-H̲$_3$), 2.10 – 2.03 (m, 1H, C**2**-H̲), 1.80 (dddd, *J* = 13.6, 8.5, 6.9, 3.3 Hz, 1H, C**2**-H̲'), 1.59 – 1.50 (m, 2H, C**9**-H̲$_2$), 1.34 – 1.26 (m, 2H, C**10**-H̲$_2$), 0.90 (t, *J* = 7.3 Hz, 3H, C**11**-H̲$_3$); ^{13}C NMR (500 MHz, $CDCl_3$) δ_C = 210.4 (C**7**), 154.0 (Cbz C̲=O), 137.8 (C**5**), 136.3 (Cbz ArC̲), 128.7 (Cbz ArC̲H), 128.3 (Cbz ArC̲H), 128.2 (Cbz ArC̲H), 107.5 (C**4**), 67.6 (Cbz C̲H$_2$), 46.8 (C**3**), 43.5 (C**1**), 40.4 (C**8**), 26.0 (C**9**), 24.6 (C**2**), 22.9 (C**6**), 22.5 (C**10**), 14.0 (C**11**); HRMS (ESI$^+$) calculated for $C_{19}H_{25}NNaO_3$ [M+Na]$^+$ = 338.1727, found 338.1723.

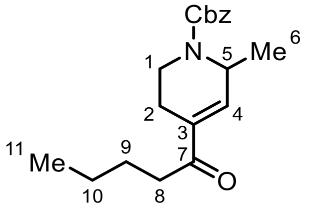

IR (thin film) ν_{max}/cm^{-1}: 2989, 2971, 2901, 1701, , 1407, 1215, 1066, 892; ^{1}H NMR (500 MHz, $CDCl_3$) δ_H = 7.46 – 7.27 (m, 5H, Cbz ArCH̲), 6.66 (d, *J* = 16.6 Hz, 1H, C**4**-H̲), 5.26 – 5.09 (m, 2H, Cbz CH̲$_2$), 4.89 – 4.63 (m, 1H, C**5**-H̲), 4.37 – 4.10 (m, 1H, C**1**-H̲), 2.98 – 2.75 (m, 1H, C**1**-H̲'), 2.63 (t, *J* = 7.5 Hz,

2H, C**8**-H̲$_2$), 2.54 – 2.42 (m, 1H, C**2**-H̲), 2.23 – 2.12 (m, 1H, C**2**-H̲'), 1.64 – 1.53 (m, 2H, C**9**-H̲$_2$), 1.44 – 1.18 (m, 5H, C**6**-H̲$_3$ + C**10**-H̲$_2$), 0.92 (t, *J* = 7.3 Hz, 3H, C**11**-H̲$_3$); ^{13}C NMR (125 MHz, $CDCl_3$) δ_C = 200.4 (C**7**), 154.8 (Cbz C̲=O), 139.8 (C**4**), 137.0 (Cbz ArC̲), 136.8 (C**3**), 128.7 (Cbz ArC̲H), 128.2 (Cbz ArC̲H), 128.1 (Cbz ArC̲H), 67.3 (Cbz C̲H$_2$), 48.3 (C**5**), 37.0 (C**8**), 36.6 (C**1**), 26.8 (C**9**), 23.5 (C**2**), 22.6 (C**10**), 18.2 (C**6**), 14.1 (C**11**); HRMS (ESI$^+$) calculated for $C_{19}H_{25}NNaO_3$ [M+Na]$^+$ = 338.1727, found 338.1724.

Some signal broadening was observed due to amide-like resonance resulting in weak signal intensities in ^{13}C NMR spectrum.

2-(2-((*tert*-Butyldimethylsilyl)oxy)ethyl)-3-methyl-1-phenylbut-3-en-1-one

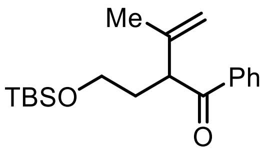

General Procedure L: Methyl 2-(2-((*tert*-butyldimethylsilyl)oxy)ethyl)-3-methylbut-3-enoate (681 mg, 2.50 mmol) was employed with PhMgBr (20 mmol), HN(OMe)Me·HCl (293 mg, 3.00 mmol) and NaOMe (33.8 mg, 0.63 mmol). Purification by flash column chromatography (Hexane/EtOAc = 100/0 to 95/5) afforded the title compound (595 mg, 75%) as a colorless oil. IR (thin film) ν_{max}/cm^{-1}: 2956, 2929, 1683, 1254, 1105, 834; ^{1}H NMR (500 MHz, $CDCl_3$) δ_H = 8.05 – 7.92 (m, 2H), 7.56 – 7.50 (m, 1H), 7.48 – 7.39 (m, 2H), 4.95 – 4.93 (m, 1H), 4.93 – 4.91 (m, 1H), 4.39 – 4.31 (m, 1H), 3.68 – 3.56 (m, 2H), 2.20 (dddd, *J* = 13.9, 7.6, 6.7, 5.2 Hz, 1H), 1.84 (ddd, *J* = 13.9, 6.5, 6.5, 5.4 Hz, 1H), 1.72 (s, 3H), 0.87 (s, 9H), 0.00 (s, 3H), -0.03 (s, 3H); ^{13}C NMR (125 MHz, $CDCl_3$) δ_C = 200.4, 143.3, 137.3, 132.9, 128.6, 128.6, 115.0, 60.8, 51.0, 33.3, 26.0, 20.6, 18.4, -5.3, -5.3; HRMS (ESI$^+$) calculated for $C_{19}H_{30}NaO_2Si$ [M+Na]$^+$ = 341.1907, found 341.1902.

2-(2-Hydroxyethyl)-3-methyl-1-phenylbut-3-en-1-one

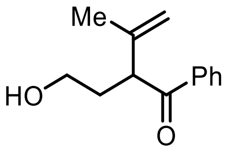

General Procedure M: The preceding silyl ether (478 mg, 1.50 mmol) was employed with 1:1 TBAF/AcOH (1.0 M in THF, 2.25 mL, 2.25 mmol). Purification by flash column chromatography (Hexane/EtOAc = 90/10 to 85/15) afforded the title compound (284 mg, 93%) as a colorless oil. IR (thin film) ν_{max}/cm^{-1}: 3414, 2972, 2937, 1680, 1447, 1229, 1052, 899; ^{1}H NMR (500 MHz, C_6D_6) δ_H = 8.08 – 7.95 (m, 2H), 7.14 – 7.00 (m, 3H), 4.96 – 4.89 (m, 1H), 4.82 – 4.74 (m, 1H), 4.28 (dd, *J* = 7.9, 6.1 Hz, 1H), 3.42 (dd, *J* = 6.1, 6.0 Hz, 2H), 2.26 (ddt, *J* = 14.0, 7.9, 6.0 Hz, 1H), 1.85 –1.77 (m, 1H), 1.60 (s, 3H), 1.15 (br s, 1H); ^{13}C NMR (125 MHz, C_6D_6) δ_C = 199.7, 143.8, 137.7, 132.8, 128.8, 128.6,

115.0, 60.5, 51.6, 33.5, 20.3; HRMS (ESI$^+$) calculated for $C_{13}H_{16}NaO_2$ $[M+Na]^+$ = 227.1042, found 227.1046.

Benzyl (3-benzoyl-4-methylpent-4-en-1-yl)((perfluorobenzoyl)oxy)carbamate (3c)

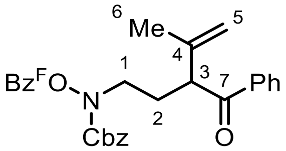

General procedure N: The preceding alcohol (284 mg, 1.39 mmol) was employed with CbzNHO^{F}Bz (502 mg, 1.39 mmol), triphenylphosphine (456 mg, 1.74 mmol) and diisopropyl azodicarboxylate (337 mg, 331 µL, 1.67 mmol). Purification by flash column chromatography (Hexane/EtOAc = 95/5 to 90/10) afforded the title compound (466 mg, 61%) as a colorless oil. IR (thin film) ν_{max}/cm^{-1}: 2988, 2901, 1735, 1507, 1228, 902; ^{1}H NMR (500 MHz, $CDCl_3$) δ_H = 7.98 – 7.92 (m, 2H, ArCH), 7.56 – 7.50 (m, 1H, ArCH), 7.44 – 7.39 (m, 2H, ArCH), 7.36 – 7.29 (m, 3H, Cbz ArCH), 7.29 – 7.26 (m, 2H, Cbz ArCH), 5.18 (d, J = 12.2 Hz, 1H, Cbz CH), 5.08 (d, J = 12.2 Hz, 1H, Cbz CH'), 4.97 – 4.92 (m, 2H, C**5**-H_2), 4.30 (dd, J = 8.0, 6.2 Hz, 1H, C**3**-H), 3.85 – 3.76 (m, 2H, C**1**-H_2), 2.36 – 2.26 (m, 1H, C**2**-H), 2.03 – 1.94 (m, 1H, C**2**-H'), 1.69 (s, 3H, C**6**-H_3); ^{19}F NMR (471 MHz, $CDCl_3$) δ_F = -135.2 – -136.7 (m, 2F), -145.9 (tt, J = 20.9, 5.5 Hz, 1F), -158.8 – -159.9 (m, 2F); ^{13}C NMR (125 MHz, $CDCl_3$) δ_C = 199.4 (C**7**), 155.5 (Cbz C=O), 142.6 (C**4**), 136.7 (ArC), 135.2 (Cbz ArC), 133.1 (ArCH), 128.7 (Cbz ArCH), 128.6 (Cbz ArCH), 128.6 (ArCH), 128.2 (Cbz ArCH), 128.2 (ArCH), 116.1 (C**5**), 68.9 (Cbz CH_2), 51.8 (C**3**), 49.6 (C**1**), 27.4 (C**2**), 20.2 (C**6**); HRMS (ESI$^+$) calculated for $C_{28}H_{22}F_5NNaO_5$ $[M+Na]^+$ = 570.1310, found 570.1312.

The carbon signals corresponding to the pentafluorobenzoyl group could not be resolved due to their weak intensity.

Benzyl 4-benzoyl-6-methyl-3,4-dihydropyridine-1(2*H*)-carboxylate and benzyl 4-benzoyl-6-methyl-3,6-dihydropyridine-1(2*H*)-carboxylate (4c)

General procedure O: The preceding substrate (54.8 mg, 0.10 mmol) was employed with $Pd_2(dba)_3$ (2.29 mg, 0.0025 mmol), CgP(2-benzofuryl) (**L3**) (4.99 mg, 0.015 mmol), CsOAc (19.2 mg, 0.10 mmol), Et_3N (14.0 µL, 0.10 mmol) and dibutyl ether (1.0 mL). The reaction was stirred at 110 °C for 16 h. Purification by flash column chromatography (Hexane/EtOAc = 95/5 to 85/15) afforded the title compounds (benzyl 4-benzoyl-6-methyl-3,4-dihydropyridine-1(2*H*)-carboxylate: 20.8 mg, 62%; benzyl 4-benzoyl-6-methyl-3,6-dihydropyridine-1(2*H*)-carboxylate: 9.10 mg, 27%) as colorless oils.

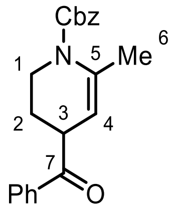

IR (thin film) ν_{max}/cm^{-1}: 2988, 2970, 1709, 1469, 1066, 892; ^{1}H NMR (500 MHz, $CDCl_3$) δ_H = 7.97 – 7.92 (m, 2H, ArCH), 7.60 – 7.56 (m, 1H, ArCH), 7.51 – 7.46 (m, 2H, ArCH), 7.40 – 7.31 (m, 5H, Cbz ArCH), 5.17 (s, 2H, Cbz CH_2), 4.92 (d, *J* = 3.5 Hz, 1H, C**4**-H), 4.10 – 4.03 (m, 1H, C**3**-H), 3.91 (ddd, *J* = 12.7, 6.8, 3.4 Hz, 1H, C**1**-H), 3.62 (ddd, *J* = 12.7, 9.2, 3.0 Hz, 1H, C**1**-H'), 2.27 – 2.21 (m, 1H, C**2**-H), 2.14 – 2.07 (m, 3H, C**6**-H_3), 1.99 – 1.91 (m, 1H, C**2**-H'); ^{13}C NMR (125 MHz, $CDCl_3$) δ_C = 199.8 (C**7**), 154.1 (Cbz C=O), 138.1 (C**5**), 136.3 (Cbz ArC), 135.9 (ArC), 133.2 (ArCH), 128.9 (ArCH), 128.7 (Cbz ArCH), 128.6 (ArCH), 128.3 (Cbz ArCH), 128.2 (Cbz ArCH), 108.1 (C**4**), 67.6 (Cbz CH_2), 43.4 (C**1**), 41.4 (C**3**), 25.2 (C**2**), 22.9 (C**6**); HRMS (ESI$^+$) calculated for $C_{21}H_{21}NNaO_3$ $[M+Na]^+$ = 358.1414, found 358.1416.

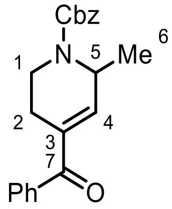

IR (thin film) ν_{max}/cm^{-1}: 2972, 2903, 1698, 1424, 1253, 1197, 892; ^{1}H NMR (500 MHz, $CDCl_3$) δ_H = 7.69 – 7.61 (m, 2H, ArCH), 7.56 – 7.52 (m, 1H, ArCH), 7.47 – 7.42 (m, 2H, ArCH), 7.40 – 7.31 (m, 5H, Cbz CH_2), 6.53 – 6.24 (m, 1H, C**4**-H), 5.23 – 5.13 (m, 2H, Cbz CH_2), 4.93 – 4.69 (m, 1H, C**5**-H), 4.48 – 4.17 (m, 1H, C**1**-H), 3.06 – 2.84 (m, 1H, C**1**-H'), 2.75 – 2.62 (m, 1H, C**2**-H), 2.48 – 2.32 (m, 1H, C**2**-H'), 1.28 (d, *J* = 6.9 Hz, 3H, C**6**-H_3); ^{13}C NMR (125 MHz, $CDCl_3$) δ_C = 196.6 (C**7**), 154.8 (Cbz C=O), 143.6 (C**4**), 137.8 (ArC), 136.8 (Cbz ArC), 136.2 (C**3**), 132.1 (ArCH), 129.4 (ArCH), 128.7 (Cbz ArCH), 128.4 (ArCH), 128.2 (Cbz ArCH), 128.1 (Cbz ArCH), 67.4 (Cbz CH_2), 48.4 (C**5**), 36.9 (C**1**), 24.4 (C**2**), 18.2 (C**6**); HRMS (ESI$^+$) calculated for $C_{21}H_{21}NNaO_3$ $[M+Na]^+$ = 358.1414, found 358.1415.

Some signal broadening was observed due to amide-like resonance resulting in weak signal intensities in ^{13}C NMR spectrum.

3-(2-((*tert*-Butyldimethylsilyl)oxy)ethyl)-2-methylnona-1,8-dien-4-one

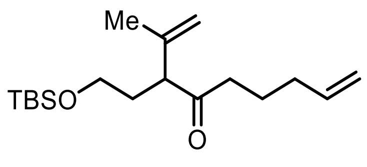

General Procedure L: Methyl 2-(2-((*tert*-butyldimethylsilyl)oxy)ethyl)-3-methylbut-3-enoate (1.36 g, 5.00 mmol) was employed with pent-4-en-1-ylmagnesium bromide (40.0 mmol), HN(OMe)Me·HCl (585 mg, 6.00 mmol) and NaOMe (67.5 mg, 1.25 mmol). Purification by flash column chromatography (Hexane/EtOAc = 100/0 to 95/5) afforded the title compound (1.11 g, 72%) as a colorless oil. IR (thin film) ν_{max}/cm^{-1}: 2953, 2929, 1713, 1361, 1254, 835; ^{1}H NMR (500 MHz, $CDCl_3$) δ_H = 5.75 (ddt, *J* = 17.0, 10.2, 6.7 Hz, 1H), 5.03 – 4.97 (m, 1H), 4.97 – 4.91 (m, 2H), 4.91 – 4.86 (m, 1H), 3.52 (dd, *J* = 6.1, 6.1 Hz, 2H), 3.37 (dd, *J* = 7.1, 7.1 Hz, 1H), 2.53 (ddd, *J* = 17.2, 8.0, 7.0 Hz, 1H), 2.38 (ddd, *J* = 17.2, 8.0, 6.5 Hz, 1H), 2.06 – 1.96 (m, 3H), 1.70 – 1.57 (m, 6H), 0.87 (s, 9H), 0.02 (s, 3H), 0.01 (s, 3H); ^{13}C NMR (125 MHz, $CDCl_3$) δ_C = 210.5, 142.8, 138.2, 115.2, 115.0, 60.8, 56.8, 40.4, 33.2, 32.0, 26.0, 23.0, 20.2, 18.4, -5.2, -5.3; HRMS (ESI$^+$) calculated for $C_{18}H_{34}NaO_2Si$ [M+Na]$^+$ = 333.2220, found 333.2219.

Methyl (*E*)-8-(2-((*tert*-butyldimethylsilyl)oxy)ethyl)-9-methyl-7-oxodeca-2,9-dienoate

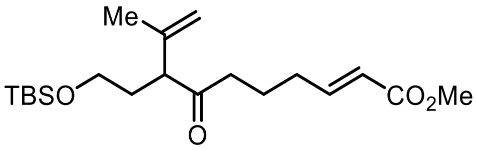

Methyl acrylate (1.29 g, 1.35 mL, 15.0 mmol) was added to a mixture of the preceding olefin (466 mg, 1.50 mmol) and Hoveyda-Grubbs catalyst 2nd generation (9.45 mg) in degassed (N_2) CH_2Cl_2 (24.0 mL) under N_2 atmosphere, and the reaction mixture was heated at 42 °C overnight. Upon completion, the solvent was removed in *vacuo*. The residue was purified by flash column chromatography (Hexane/EtOAc = 100/0 to 94/6) to afford the title compound (410 mg, 74%) as a colorless oil. IR (thin film) ν_{max}/cm^{-1}: 2952, 2929, 1726, 1362, 1256, 834; ^{1}H NMR (500 MHz, $CDCl_3$) δ_H = 6.90 (dt, *J* = 15.6, 6.9 Hz, 1H), 5.80 (dt, *J* = 15.6, 1.6 Hz, 1H), 4.95 – 4.90 (m, 1H), 4.89 – 4.84 (m, 1H), 3.70 (s, 3H), 3.58 – 3.49 (m, 2H), 3.35 (dd, *J* = 7.1, 7.1 Hz, 1H), 2.56 (dt, *J* = 17.5, 7.3 Hz, 1H), 2.38 (dt, *J* = 17.5, 7.0 Hz, 1H), 2.23 – 2.10 (m, 2H), 2.03 – 1.95 (m, 1H), 1.73 – 1.67 (m, 2H), 1.67 – 1.60 (m, 4H), 0.86 (s, 9H), 0.00 (s, 3H), -0.00 (s, 3H); ^{13}C NMR (125 MHz, $CDCl_3$) δ_C = 209.8, 167.1, 148.7, 142.7, 121.6, 115.1, 60.8, 56.9, 51.5, 40.1, 31.9, 31.5, 26.0, 22.0, 20.2, 18.4, -5.3, -5.3; HRMS (ESI$^+$) calculated for $C_{20}H_{36}NaO_4Si$ [M+Na]$^+$ = 391.2275, found 391.2273.

Methyl (*E*)-8-(2-hydroxyethyl)-9-methyl-7-oxodeca-2,9-dienoate

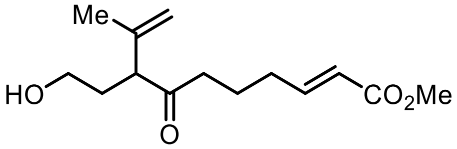

General Procedure M: The preceding silyl ether (369 mg, 1.00 mmol) was employed with 1:1 TBAF/AcOH (1.0 M in THF, 1.70 mL, 1.70 mmol). Purification by flash column chromatography

(Pentane/Et_2O = 50/50 to 25/75) afforded the title compound (223 mg, 88%) as a colorless oil. IR (thin film) ν_{max}/cm^{-1}: 3427, 2954, 2931, 1710, 1437, 1273, 902; ^{1}H NMR (500 MHz, C_6D_6) δ_H = 6.92 (dt, J = 15.5, 7.0 Hz, 1H), 5.81 (dt, J = 15.5, 1.6 Hz, 1H), 4.81 – 4.77 (m, 2H), 3.44 – 3.31 (m, 2H), 3.42 (s, 3H), 3.25 – 3.18 (m, 1H), 2.26 – 2.20 (m, 1H), 2.11 – 1.96 (m, 2H), 1.83 – 1.73 (m, 2H), 1.60 – 1.55 (m, 1H), 1.52 (s, 3H), 1.50 – 1.46 (m, 2H); ^{13}C NMR (125 MHz, C_6D_6) δ_C = 208.8, 166.7, 148.7, 143.3, 121.9, 114.8, 60.6, 57.3, 51.0, 40.1, 32.3, 31.5, 22.2, 20.0; HRMS (ESI$^+$) calculated for $C_{14}H_{22}NaO_4$ [M+Na]$^+$ = 277.1410, found 277.1413.

Methyl (*E*)-8-(2-(((benzyloxy)carbonyl)((perfluorobenzoyl)oxy)amino)ethyl)-9-methyl-7-oxodeca-2,9-dienoate (3d)

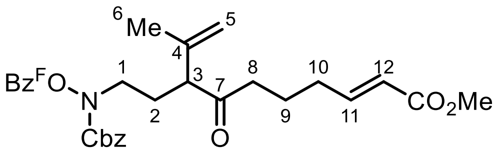

General procedure N: The preceding alcohol (204 mg, 0.80 mmol) was employed with CbzNHO^{F}Bz (289 mg, 0.80 mmol), triphenylphosphine (262 mg, 1.00 mmol) and diisopropyl azodicarboxylate (194 mg, 190 µL, 0.96 mmol). Purification by flash column chromatography (Hexane/EtOAc = 95/5 to 85/15) afforded the title compound (342 mg, 71%) as a colorless solid. m.p. 48 - 50 °C (EtOAc/hexane); IR (thin film) ν_{max}/cm^{-1}: 2951, 1784, 1654, 1327, 1176, 907; ^{1}H NMR (500 MHz, $CDCl_3$) δ_H = 7.43 – 7.28 (m, 5H, Cbz ArC<u>H</u>), 6.90 (dt, J = 15.6, 6.9 Hz, 1H, C**11**-<u>H</u>), 5.80 (dt, J = 15.7, 1.6 Hz, 1H, C**12**-<u>H</u>), 5.20 (s, 2H, Cbz C<u>H$_2$</u>), 5.01 – 4.94 (m, 1H, C**5**-<u>H</u>), 4.94 – 4.87 (m, 1H, C**5**-<u>H</u>'), 3.78 – 3.59 (m, 2H, C**1**-<u>H$_2$</u>), 3.72 (s, 3H, CO_2C<u>H$_3$</u>), 3.35 (dd, J = 7.2, 7.2 Hz, 1H, C**3**-<u>H</u>), 2.54 (dt, J = 17.5, 7.4 Hz, 1H, C**8**-<u>H</u>), 2.33 (dt, J = 17.5, 7.0 Hz, 1H, C**8**-<u>H</u>'), 2.20 – 2.06 (m, 3H, C**10**-<u>H$_2$</u> + C**2**-<u>H</u>), 1.80 – 1.72 (m, 1H, C**2**-<u>H</u>'), 1.71 – 1.64 (m, 2H, C**9**-<u>H$_2$</u>), 1.62 (s, 3H, C**6**-<u>H$_3$</u>); ^{19}F NMR (471 MHz, $CDCl_3$) δ_F = -133.8 – -138.3 (m, 2F), -145.8 (tt, J = 20.9, 5.3 Hz, 1F), -157.8 – -161.5 (m, 2F); ^{13}C NMR (125 MHz, $CDCl_3$) δ_C = 209.1 (C**7**), 167.1 (<u>C</u>O_2CH_3), 155.5 (Cbz <u>C</u>=O), 148.6 (C**11**), 141.8 (C**4**), 135.2 (Cbz ArC), 128.7 (Cbz ArCH), 128.7 (Cbz ArCH), 128.3 (Cbz ArCH), 121.6 (C**12**), 116.3 (C**5**), 69.0 (Cbz <u>C</u>H_2), 57.1 (C**3**), 51.6 (CO_2<u>C</u>H_3), 49.3 (C**1**), 40.1 (C**8**), 31.5 (C**10**), 25.9 (C**2**), 22.0 (C**9**), 19.9 (C**6**); HRMS (ESI$^+$) calculated for $C_{29}H_{28}F_5NNaO_7$ [M+Na]$^+$ = 620.1678, found 620.1680.

The carbon signals corresponding to the pentafluorobenzoyl group could not be resolved due to their weak intensity.

Benzyl (*E*)-4-(7-methoxy-7-oxohept-5-enoyl)-6-methyl-3,4-dihydropyridine-1(2*H*)-carboxylate and benzyl (*E*)-4-(7-methoxy-7-oxohept-5-enoyl)-6-methyl-3,6-dihydropyridine-1(2*H*)-carboxylate (4d)

General procedure O: The preceding substrate (59.8 mg, 0.10 mmol) was employed with $Pd_2(dba)_3$ (2.29 mg, 0.0025 mmol), CgP(2-benzofuryl) (**L3**) (4.99 mg, 0.015 mmol), CsOAc (19.2 mg, 0.10

mmol), Et_3N (14.0 µL, 0.10 mmol) and dibutyl ether (1.0 mL). The reaction was stirred at 110 °C for 16 h. Purification by flash column chromatography (Hexane/EtOAc = 95/5 to 80/20) afforded the title compounds (benzyl (*E*)-4-(7-methoxy-7-oxohept-5-enoyl)-6-methyl-3,4-dihydropyridine-1(2*H*)-carboxylate: 17.3 mg, 45%; benzyl (*E*)-4-(7-methoxy-7-oxohept-5-enoyl)-6-methyl-3,6-dihydropyridine-1(2*H*)-carboxylate: 13.2 mg, 34%) as colorless oils.

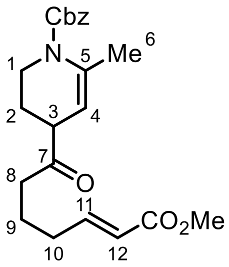

IR (thin film) ν_{max}/cm^{-1}: 2988, 2976, 1403, 1251, 1228, 1076, 892; ^{1}H NMR (500 MHz, $CDCl_3$) δ_H = 7.40 – 7.31 (m, 5H, Cbz ArC<u>H</u>), 6.91 (dt, *J* = 15.7, 6.9 Hz, 1H, C**11**-<u>H</u>), 5.82 (dt, *J* = 15.7, 1.6 Hz, 1H, C**12**-<u>H</u>), 5.15 (s, 2H, Cbz C<u>H</u>$_2$), 4.90 (d, *J* = 3.2 Hz, 1H, C**4**-<u>H</u>), 3.73 – 3.68 (m, 1H, C**1**-<u>H</u>), 3.72 (s, 3H, CO_2C<u>H</u>$_3$), 3.57 (ddd, *J* = 12.9, 8.5, 3.2 Hz, 1H, C**1**-<u>H</u>'), 3.13 – 3.05 (m, 1H, C**3**-<u>H</u>), 2.58 – 2.43 (m, 2H, C**8**-<u>H</u>$_2$), 2.25 – 2.17 (m, 2H, C**10**-<u>H</u>$_2$), 2.13 (dd, *J* = 2.0, 1.2 Hz, 3H, C**6**-<u>H</u>$_3$), 2.09 – 2.02 (m, 1H, C**2**-<u>H</u>), 1.83 – 1.78 (m, 1H, C**2**-<u>H</u>'), 1.77 – 1.71 (m, 2H, C**9**-<u>H</u>$_2$);^{13}C NMR (125 MHz, $CDCl_3$) δ_C = 209.4 (C**7**), 167.0 (<u>C</u>O_2CH_3), 154.0 (Cbz <u>C</u>=O), 148.5 (C**11**), 138.1 (C**5**), 136.3 (Cbz ArC), 128.7 (Cbz Ar<u>C</u>H), 128.3 (Cbz Ar<u>C</u>H), 128.2 (Cbz ArCH), 121.7 (C**12**), 107.1 (C**4**), 67.7 (Cbz <u>C</u>H$_2$), 51.6 (CO_2<u>C</u>H_3), 46.9 (C**3**), 43.5 (C**1**), 39.5 (C**8**), 31.5 (C**10**), 24.5 (C**2**), 23.0 (C**6**), 22.0 (C**9**); HRMS (ESI$^+$) calculated for $C_{22}H_{27}NNaO_5$ $[M+Na]^+$ = 408.1781, found 408.1782.

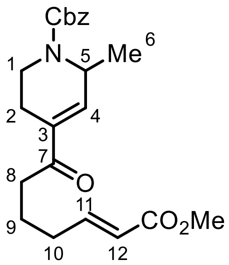

IR (thin film) ν_{max}/cm^{-1}: 2988, 2901, 1711, 1473, 1234, 894; ^{1}H NMR (500 MHz, $CDCl_3$) δ_H = 7.38 – 7.31 (m, 5H, Cbz ArC<u>H</u>), 6.93 (dt, *J* = 15.7, 6.9 Hz, 1H, C**11**-<u>H</u>), 6.78 – 6.50 (m, 1H, C**4**-<u>H</u>), 5.84 (dt, *J* = 15.7, 1.6 Hz, 1H, C**12**-<u>H</u>), 5.20 – 5.11 (m, 2H, Cbz C<u>H</u>$_2$), 4.86 – 4.65 (m, 1H, C**5**-<u>H</u>), 4.38 – 4.13 (m, 1H, C**1**-<u>H</u>), 3.72 (s, 3H, CO_2C<u>H</u>$_3$), 2.94 – 2.75 (m, 1H, C**1**-<u>H</u>'), 2.71 – 2.58 (m, 2H, C**8**-<u>H</u>$_2$), 2.52 – 2.45 (m, 1H, C**2**-<u>H</u>), 2.27 – 2.21 (m, 2H, C**10**-<u>H</u>$_2$), 2.19 – 2.12 (m, 1H, C**2**-<u>H</u>'), 1.85 – 1.76 (m, 2H, C**9**-<u>H</u>$_2$), 1.29 (d, *J* = 6.9 Hz, 3H, C**6**-<u>H</u>$_3$); ^{13}C NMR (125 MHz, $CDCl_3$) δ_C = 199.2 (C**7**), 167.1 (<u>C</u>O_2CH_3), 154.7 (Cbz <u>C</u>=O), 148.6 (C**11**), 140.1 (C**4**), 137.0 (C**3**), 136.7 (Cbz ArC), 128.7 (Cbz Ar<u>C</u>H), 128.2 (Cbz Ar<u>C</u>H), 128.0 (Cbz ArCH), 121.8 (C**12**), 67.3 (Cbz <u>C</u>H$_2$), 51.6 (CO_2<u>C</u>H_3), 48.3 (C**5**), 36.8 (C**1**),

36.1 (C**8**), 31.6 (C**10**), 23.5 (C**2**), 22.6 (C**9**), 18.7 (C**6**); HRMS (ESI$^+$) calculated for $C_{22}H_{27}NNaO_5$ $[M+Na]^+$ = 408.1781, found 408.1775.

Some signal broadening was observed due to amide-like resonance resulting in weak signal intensities in ^{13}C NMR spectrum.

1-Cyclopropyl-2-(trimethylsilyl)ethan-1-one

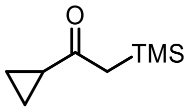

General Procedure I: $TMSCH_2MgCl$ (1.0 M in Et_2O, 38.5 mL, 38.5 mmol) was employed with cyclopropanecarbonyl chloride (3.66 g, 3.18 ml, 35.0 mmol) and copper iodide (7.33 g, 38.5 mmol). 1-(trimethylsilyl)-propan-2-one (4.45 g, 81 %) was afforded as a yellow oil which was used without any further purification.

3-(1-Cyclopropyl-1-hydroxy-2-(trimethylsilyl)ethyl)dihydrofuran-2(3*H*)-one

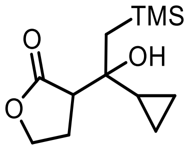

General Procedure J: Butyrolactone (1.29 g, 15.0 mmol) was employed with 1.0 M LiHMDS in THF (18.0 ml, 18.0 mmol) and 1-(trimethylsilyl)-propan-2-one (3.28 g, 21.0 mmol). Purification by flash column chromatography (Hexane/Et_2O = 85/15 to 75/25) afforded the title compounds (1.78 g, 49%) as a mixture of diastereomers (2.9:1) and yellow oils. IR (thin film) ν_{max}/cm^{-1}: 3500, 2952, 1743, 1376, 1246, 1182; *Diastereomer 1:* ^{1}H NMR (500 MHz, $CDCl_3$) δ_H = 4.43 – 4.35 (m, 1H), 4.26 – 4.18 (m, 1H), 3.59 (br s, 1H), 2.76 (dd, *J* = 11.1, 9.3 Hz, 1H), 2.45 – 2.34 (m, 1H), 2.24 – 2.15 (m, 1H), 1.02 (d, *J* = 14.5 Hz, 1H), 0.97 (d, *J* = 14.5 Hz, 1H), 0.88 – 0.82 (m, 1H), 0.76 (dddd, *J* = 13.5, 8.3, 5.1, 1.5 Hz, 1H), 0.47 – 0.42 (m, 2H), 0.40 – 0.30 (m, 1H), 0.09 (s, 9H); ^{13}C NMR (125 MHz $CDCl_3$) δ_C = 180.1, 72.9, 66.9, 51.8, 26.8, 25.4, 20.5, 3.1, 1.1, 0.7; *Diastereomer 2:* ^{1}H NMR (500 MHz, $CDCl_3$) δ_H = 4.43 – 4.35 (m, 1H), 4.26 – 4.18 (m, 1H), 3.28 (br s, 1H), 2.85 (dd, *J* = 9.5, 9.5 Hz, 1H), 2.45 – 2.34 (m, 2H), 1.22 (d, *J* = 14.6 Hz, 1H), 0.93 – 0.91 (m, 1H), 0.90 – 0.88 (m, 1H), 0.54 – 0.48 (m, 2H), 0.40 – 0.30 (m, 2H), 0.09 (s, 9H); ^{13}C NMR (125 MHz $CDCl_3$) δ_C = 179.3, 73.5, 66.8, 50.0, 29.8, 25.3, 19.2, 3.1, 1.8, 0.7; HRMS (ESI$^+$) calculated for $C_{12}H_{22}NaO_3Si$ $[M+Na]^+$ = 265.1230, found 265.1229.

3-(1-Cyclopropylvinyl)dihydrofuran-2(3*H*)-one

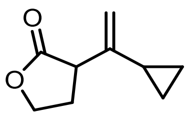

General Procedure K: The preceding lactone (1.72 g, 7.10 mmol) was employed with HF-pyridine (7.10 ml) in THF (85.5 mL). Purification by flash column chromatography (Hexane/Et_2O = 75/25 to 60/40) afforded the title compound (659 mg, 61%) as a colorless oil. IR (thin film) ν_{max}/cm^{-1}: 3004, 1764, 1642, 1455, 1371, 1148; ^{1}H NMR (500 MHz, $CDCl_3$) δ_H = 4.90 (s, 1H), 4.87 (s, 1H), 4.39 (ddd, *J* = 9.0, 7.6, 5.0 Hz, 1H), 4.31 – 4.26 (m, 1H), 3.33 (dd, *J* = 9.0, 9.0 Hz, 1H), 2.49 – 2.41 (m, 2H), 1.37 – 1.29 (m, 1H), 0.75 – 0.68 (m, 2H), 0.58 – 0.52 (m, 1H), 0.50 – 0.45 (m, 1H); ^{13}C NMR (125 MHz $CDCl_3$) δ_C = 177.4, 145.7, 110.9, 66.8, 47.0, 28.8, 14.5, 7.0, 6.2; HRMS (ESI$^+$) calculated for $C_9H_{13}O_2$ $[M+H]^+$ = 153.0910, found 153.0909.

4-Cyclopropyl-3-(2-hydroxyethyl)pent-4-en-2-one

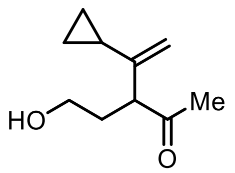

General Procedure L: The preceding lactone (304 mg, 2.00 mmol) was employed with MeMgBr (3.0 M in Et_2O, 5.30 mL, 16.0 mmol), HN(OMe)Me·HCl (234 mg, 2.40 mmol) and NaOMe (21.6 mg, 0.40 mmol). The residue was purified quickly by flash column chromatography (Pentane/Et_2O = 50/50 to 25/75) giving a crude product which was taken through to the next step without further purification.

Benzyl (3-acetyl-4-cyclopropylpent-4-en-1-yl)((perfluorobenzoyl)oxy)carbamate (3e)

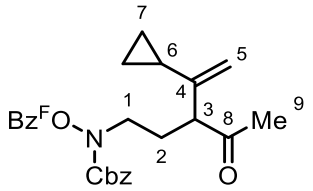

General procedure N: The preceding crude alcohol (92.5 mg, 0.55 mmol) was employed with CbzNHO^{F}Bz (179 mg, 0.50 mmol), triphenylphosphine (216 mg, 0.83 mmol) and diisopropyl azodicarboxylate (167 mg, 163 µL, 0.83 mmol). Purification by flash column chromatography (Toluene/EtOAc = 100/0 to 98/2) afforded the title compound (80.3 mg, 8% over 2 steps) as a colorless oil. IR (thin film) ν_{max}/cm^{-1}: 3086, 1782, 1713, 1653, 1498, 1172; ^{1}H NMR (500 MHz, $CDCl_3$) δ_H = 7.38 – 7.31 (m, 5H, Cbz ArC$\underline{H}$), 5.21 (s, 2H, Cbz C$\underline{H}_2$), 4.79 (s, 1H, C**5**-$\underline{H}$), 4.76 (s, 1H, C**5**-$\underline{H}$'), 3.78 – 3.66 (m, 2H, C**1**-$\underline{H}_2$), 3.38 (dd, *J* = 7.2, 7.2 Hz, 1H, C**3**-$\underline{H}$), 2.22 – 2.17 (m, 1H, C**2**-$\underline{H}$), 2.12 (s, 3H, C**9**-$\underline{H}_3$), 1.92 – 1.85 (m, 1H, C**2**-$\underline{H}$'), 1.18 – 1.12 (m, 1H, C**6**-$\underline{H}$), 0.72 – 0.63 (m, 2H, C**7**-H + C**7**'-$\underline{H}$), 0.49 – 0.40 (m, 2H, C**7**-H' + C**7**'-$\underline{H}$'); ^{19}F NMR (471 MHz, $CDCl_3$) δ_F = -134.4 – 136.2 (m, 2F), -145.8 (tt, *J* = 20.8, 5.2 Hz, 1F), -158.2 – -159.8 (m, 2F); ^{13}C NMR (125 MHz, $CDCl_3$) δ_c = 207.9 (C**8**), 155.5 (Cbz $\underline{C}$=O), 147.9 (C**4**), 135.3 (Cbz ArC), 128.7 (Cbz ArCH), 128.7 (Cbz ArCH), 128.3 (Cbz ArCH),

110.6 (C**5**), 69.0 (Cbz C̲H2), 57.5 (C**3**), 49.6 (C**1**), 28.6 (C**9**), 26.8 (C**2**), 14.0 (C**6**), 7.8 (C**7**), 6.9 (C**7**’); HRMS (ESI$^+$) calculated for $C_{25}H_{23}F_5NO_5$ $[M+H]^+$ = 512.1491, found 512.1485.

The carbon signals corresponding to the pentafluorobenzoyl group could not be resolved due to their weak intensity.

Benzyl 4-acetyl-6-cyclopropyl-3,4-dihydropyridine-1(2*H*)-carboxylate and benzyl 4-acetyl-6-cyclopropyl-3,6-dihydropyridine-1(2*H*)-carboxylate (4e)

General procedure O: The preceding substrate (51.1 mg, 0.10 mmol) was employed with $Pd_2(dba)_3$ (2.29 mg, 0.0025 mmol), CgP(2-benzofuryl) (**L3**) (8.31 mg, 0.025 mmol), CsOAc (19.2 mg, 0.10 mmol), Et_3N (14.0 µL, 0.10 mmol) and dibutyl ether (1.0 mL). The reaction was stirred at 110 °C for 16 h. Purification by flash column chromatography (Hexane/EtOAc = 95/5 to 85/15) afforded the title compounds (benzyl 4-acetyl-6-cyclopropyl-3,4-dihydropyridine-1(2*H*)-carboxylate: 7.51 mg, 25%; benzyl 4-acetyl-6-cyclopropyl-3,6-dihydropyridine-1(2*H*)-carboxylate: 3.68 mg, 12%) as colorless oils.

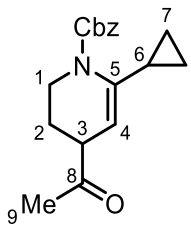

IR (thin film) ν_{max}/cm^{-1}: 2993, 1707, 1396, 1212; ^{1}H NMR (500 MHz, $CDCl_3$) δ_H = 7.37 – 7.32 (m, 5H, Cbz ArCH̲), 5.18 (s, 2H, Cbz CH̲2), 5.05 (d, *J* = 4.0 Hz, 1H, C**4**-H̲), 3.65 (ddd, *J* = 11.5, 7.6, 3.3 Hz, 1H, C**1**-H̲), 3.55 (ddd, *J* = 11.5, 8.1, 3.3 Hz, 1H, C**1**-H̲’), 3.12 – 3.07 (m, 1H, C**3**-H̲), 2.16 (s, 3H, C**9**-H̲3), 2.09 – 2.04 (m, 1H, C**2**-H̲), 1.99 – 1.93 (m, 1H, C**2**-H̲’), 1.85 – 1.79 (m, 1H, C**6**-H̲), 0.66 – 0.60 (m, 2H, C**7**-H + C**7**’-H̲), 0.46 – 0.40 (m, 2H, C**7**-H’ + C**7**’-H̲’); ^{13}C NMR (125 MHz CDCl3) δ_C = 207.9 (C**8**), 154.3 (Cbz C̲=O), 143.8 (C**5**), 136.4 (Cbz ArC̲), 128.7 (Cbz ArC̲H), 128.3 (Cbz ArC̲H), 128.3 (Cbz ArC̲H), 106.3 (C**4**), 67.7 (Cbz C̲H2), 47.5 (C**3**), 44.0 (C**1**), 28.0 (C**9**), 24.8 (C**2**), 16.0 (C**6**), 7.3 (C**7**), 7.2 (C**7**’); HRMS (ESI$^+$) calculated for $C_{18}H_{22}NO_3$ $[M+H]^+$ = 300.1594, found 300.1597.

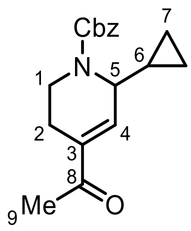

IR (thin film) ν_{max}/cm^{-1}: 2922, 1694, 1670, 1386; ^{1}H NMR (500 MHz, $CDCl_3$) δ_H = 7.37 – 7.32 (m, 5H, Cbz ArCH̲), 6.83 – 6.69 (m, 1H, C**4**-H̲), 5.18 – 5.09 (m, 2H, Cbz CH̲2), 4.40 – 4. 23 (m, 1H, C**1**-H̲), 4.14 – 3.97 (m, 1H, C**5**-H̲), 3.02 – 2.92 (m, 1H, C**1**-H̲’), 2.50 – 2.44 (m, 1H, C**2**-H̲), 2.32 (s, 3H, C**9**-H̲3), 2.20 – 2.13 (m, 1H, C**2**-H̲’), 1.04 – 0.99 (m, 1H, C**6**-H̲), 0.86 – 0.81 (m, 1H, C**7**-H̲), 0.64 – 0.60

(m, 1H, C**7**'-H), 0.52 – 0.46 (m, 1H, C**7**-H'), 0.43 – 0.31 (m, 1H, C**7**'-H'); ^{13}C NMR (125 MHz, $CDCl_3$) δ_c = 198.1 (C**8**), 155.2 (Cbz C=O), 139.1 (C**4**), 137.8 (Cbz ArC), 136.7 (C**3**), 128.7 (Cbz ArCH), 128.2 (Cbz ArCH), 128.1 (Cbz ArCH), 67.4 (Cbz CH_2), 56.4 (C**5**), 37.7 (C**1**), 25.4 (C**9**), 23.3 (C**2**), 14.6 (C**6**), 3.7 (C**7**), 3.1 (C**7**'); HRMS (ESI$^+$) calculated for $C_{18}H_{22}NO_3$ $[M+H]^+$ = 300.1594, found 300.1597.

Some signal broadening was observed due to amide-like resonance resulting in weak signal intensities in ^{13}C NMR spectrum.

4-Phenyl-1-(trimethylsilyl)butan-2-one

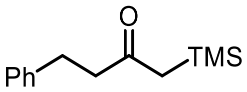

General Procedure I: $TMSCH_2MgCl$ (1.0 M in Et_2O, 19.2 mL, 19.2 mmol) was employed with 3-phenylpropanoyl chloride (2.70 g, 2.40 ml, 16.0 mmol) and copper iodide (3.35 g, 17.6 mmol). 4-Phenyl-1-(trimethylsilyl)butan-2-one (3.10 g, 88 %) was afforded as a colorless oil which was used without any further purification. IR (thin film) ν_{max}/cm^{-1}: 2955, 2898, 1689, 1496, 1453, 1407, 1250, 1185, 1102, 1010, 840; ^{1}H NMR (500 MHz, $CDCl_3$) δ_H = 7.16 – 7.12 (m, 2H), 7.12 – 7.08 (m, 2H), 7.07 – 7.02 (m, 1H), 2.87 (t, *J* = 7.5 Hz, 2H), 2.35 (t, *J* = 7.5 Hz, 2H), 1.87 (s, 2H), -0.09 (s, 9H); ^{13}C NMR (125 MHz, C_6D_6) δ_C = 205.8, 142.2, 128.8, 128.7, 126.2, 46.3, 37.6, 30.3, -1.2 ; HRMS (ESI$^+$) calculated for $C_{13}H_{20}NaOSi$ $[M+Na]^+$ = 243.1176, found 243.1173.

3-(2-Hydroxy-4-phenyl-1-(trimethylsilyl)butan-2-yl)dihydrofuran-2(3*H*)-one

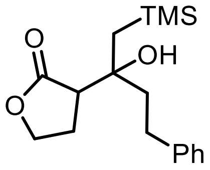

General Procedure J: Butyrolactone (431 mg, 5.00 mmol) was employed with 1.0 M LiHMDS in THF (5.50 ml, 5.50 mmol) and 1-(trimethylsilyl)-propan-2-one (1.54 g, 7.00 mmol). Purification by flash column chromatography (Hexane/Et_2O = 80/20 to 50/50) afforded the title compounds (1.08 g, 71%) as a mixture of diastereomers (5.9:1) and colorless oils. IR (thin film) ν_{max}/cm^{-1}: 3560, 2973, 2901, 1753, 1407, 1226, 867; *Diastereomer 1:* ^{1}H NMR (500 MHz, $CDCl_3$) δ_H = 7.34 – 7.26 (m, 2H), 7.24 – 7.14 (m, 3H), 4.38 (ddd, *J* = 9,0, 9.0, 2.1 Hz, 1H), 4.23 – 4.14 (m, 1H), 4.06 (d, *J* = 2.1 Hz, 1H), 3.00 – 2.86 (m, 2H), 2.60 – 2.50 (m, 1H), 2.36 – 2.18 (m, 1H), 2.16 – 2.04 (m, 2H), 1.68 – 1.60 (m, 1H), 1.06 – 0.84 (m, 2H), 0.11 (s, 9H); ^{13}C NMR (125 MHz, $CDCl_3$) δ_C = 180.3, 142.3, 128.6, 128.5, 126.0, 75.2, 66.6, 48.3, 42.3, 30.2, 27.3, 25.4, 0.7; *Diastereomer 2:* ^{1}H NMR (500 MHz, $CDCl_3$) δ_H = 7.34 – 7.26 (m, 2H), 7.24 – 7.14 (m, 3H), 4.33 (ddd, *J* = 9.0, 9.0, 2.6 Hz, 1H), 4.23 – 4.14 (m, 1H), 3.30 (d, *J* = 1.4 Hz, 1H), 3.00 – 2.86 (m, 1H), 2.84 – 2.75 (m, 1H), 2.74 – 2.62 (m, 1H), 2.36 – 2.18 (m, 2H), 1.90 – 1.71 (m, 2H), 1.06 – 0.84 (m, 2H), 0.14 (s, 9H); ^{13}C NMR (125 MHz, $CDCl_3$) δ_C = 179.0, 142.3, 128.6,

128.5, 126.0, 75.2, 66.3, 49.4, 41.7, 30.1, 27.1, 25.3, 0.7; HRMS (ESI$^+$) calculated for $C_{17}H_{26}NaO_3Si$ $[M+Na]^+$ = 329.1543, found 329.1542.

3-(4-Phenylbut-1-en-2-yl)dihydrofuran-2(3*H*)-one

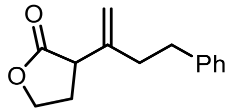

General Procedure K: The preceding lactone (1.17 g, 3.82 mmol) was employed with HF-pyridine (1.27 ml) in CH_3CN (63.7 mL). Purification by flash column chromatography (Hexane/Et_2O = 75/25 to 50/50) afforded the title compound (751 mg, 91%) as a colorless oil. IR (thin film) ν_{max}/cm^{-1}: 2988, 2907, 1768, 1373, 1214, 901; ^1H NMR (500 MHz, $CDCl_3$) δ_H = 7.32 – 7.27 (m, 2H), 7.25 – 7.16 (m, 3H), 5.12 – 5.08 (m, 1H), 5.07 – 5.04 (m, 1H), 4.34 (ddd, *J* = 8.5, 8.5, 4.5 Hz, 1H), 4.25 (ddd, *J* = 8.5, 8.5, 7.1 Hz, 1H), 3.25 (dd, *J* = 8.9, 8.9 Hz, 1H), 2.89 – 2.76 (m, 2H), 2.51 – 2.38 (m, 3H), 2.29 – 2.21 (m, 1H); ^{13}C NMR (125 MHz, $CDCl_3$) δ_C = 177.0, 143.7, 141.5, 128.5, 128.4, 126.1, 113.3, 66.6, 46.3, 36.0, 34.1, 28.6; HRMS (ESI$^+$) calculated for $C_{14}H_{16}NaO_2$ $[M+Na]^+$ = 239.1042, found 239.1041.

3-(2-Hydroxyethyl)-4-methylene-6-phenylhexan-2-one

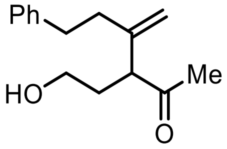

General Procedure L: The preceding lactone (663 mg, 3.07 mmol) was employed with MeMgBr (3.0 M in Et_2O, 8.20 mL, 24.6 mmol), HN(OMe)Me·HCl (359 mg, 3.68 mmol) and NaOMe (41.5 mg, 0.77 mmol). Purification by flash column chromatography (Hexane/EtOAc = 85/15 to 75/25) afforded the title compound (376 mg, 53%) as a colorless oil (alcohol:hemiketal = 6.7:1). IR (thin film) ν_{max}/cm^{-1}: 3398, 2931, 2888, 1709, 1496, 1052, 902; ^1H NMR (500 MHz, C_6D_6) δ_H = 7.16 – 7.12 (m, 2H), 7.09 – 7.02 (m, 3H), 4.89 – 4.85 (m, 1H), 4.85 – 4.83 (m, 1H), 3.45 – 3.31 (m, 2H), 3.18 (dd, *J* = 8.1, 5.9 Hz, 1H), 2.63 – 2.56 (m, 2H), 2.20 (t, *J* = 8.1 Hz, 2H), 2.10 – 2.03 (m, 1H), 1.82 (s, 3H), 1.61 – 1.54 (m, 1H), 1.57 (br s, 1H); ^{13}C NMR (125 MHz, C_6D_6) δ_C = 207.4, 147.0, 141.8, 128.7, 128.7, 126.3, 113.3, 60.7, 57.1, 36.4, 34.4, 33.1, 28.2; HRMS (ESI$^+$) calculated for $C_{15}H_{20}NaO_2$ $[M+Na]^+$ = 255.1355, found 255.1357.

Benzyl (3-acetyl-4-methylene-6-phenylhexyl)((perfluorobenzoyl)oxy)carbamate (3f)

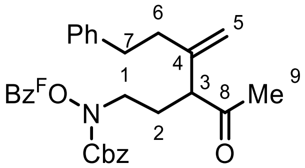

General procedure N: The preceding alcohol (372 mg, 1.60 mmol) was employed with CbzNHO^{F}Bz (524 mg, 1.45 mmol), triphenylphosphine (524 mg, 2.00 mmol) and diisopropyl azodicarboxylate (388 mg, 381 µL, 1.92 mmol). Purification by flash column chromatography (Hexane/EtOAc = 95/5 to 85/15) afforded the title compound (377 mg, 45%) as a colorless oil. IR (thin film) ν_{max}/cm^{-1}: 2988, 2968, 1713, 1499, 1229, 892; ^{1}H NMR (500 MHz, $CDCl_3$) δ_H = 7.42 – 7.29 (m, 5H, Cbz ArCH), 7.29 – 7.22 (m, 2H, ArCH), 7.21 – 7.13 (m, 3H, ArCH), 5.21 (s, 2H, Cbz CH_2), 5.07 (s, 1H, C**5**-H), 4.98 (s, 1H, C**5**-H'), 3.75 (ddd, *J* = 14.5, 7.0, 7.0 Hz, 1H, C**1**-H), 3.65 (ddd, *J* = 14.5, 6.3, 6.3 Hz, 1H, C**1**-H'), 3.34 (dd, *J* = 7.5, 7.1 Hz, 1H, C**3**-H), 2.82 – 2.68 (m, 2H, C**7**-H_2), 2.35 – 2.18 (m, 2H, C**6**-H_2), 2.13 (dddd, *J* = 14.2, 7.5, 7.0, 6.3 Hz, 1H, C**2**-H), 2.05 (s, 3H, C**9**-H_3), 1.83 – 1.72 (m, 1H, C**2**-H'); ^{19}F NMR (471 MHz, $CDCl_3$) δ_F = -134.9 – -136.6 (m, 2F), -145.8 (tt, *J* = 21.0, 5.6 Hz, 2F), -158.7 – -159.8 (m, 2F); ^{13}C NMR (125 MHz, $CDCl_3$) δ_C = 207.6 (C**8**), 155.5 (Cbz C=O), 145.7 (C**4**), 141.4 (ArC), 135.2 (Cbz ArC), 128.7 (Cbz ArCH), 128.7 (Cbz ArCH), 128.5 (ArCH), 128.4 (ArCH), 128.3 3 (Cbz ArCH), 126.2 (ArCH), 114.5 (C**5**), 69.0 (Cbz CH_2), 57.0 (C**3**), 49.5 (C**1**), 35.8 (C**6**), 34.1 (C**7**), 28.6 (C**9**), 26.9 (C**2**); HRMS (ESI$^+$) calculated for $C_{30}H_{26}F_5NNaO_5$ [M+Na]$^+$ = 598.1623, found 598.1624.

The carbon signals corresponding to the pentafluorobenzoyl group could not be resolved due to their weak intensity.

Benzyl 4-acetyl-6-phenethyl-3,4-dihydropyridine-1(2*H*)-carboxylate and benzyl 4-acetyl-6-phenethyl-3,6-dihydropyridine-1(2*H*)-carboxylate (4f)

General procedure O: The preceding substrate (57.6 mg, 0.10 mmol) was employed with $Pd_2(dba)_3$ (2.29 mg, 0.0025 mmol), CgP(2-benzofuryl) (**L3**) (4.99 mg, 0.015 mmol), CsOAc (19.2 mg, 0.10 mmol), Et_3N (14.0 µL, 0.10 mmol) and dibutyl ether (1.0 mL). The reaction was stirred at 110 °C for 16 h. Purification by flash column chromatography (Hexane/EtOAc = 95/5 to 85/15) afforded the title compounds (benzyl 4-acetyl-6-phenethyl-3,4-dihydropyridine-1(2*H*)-carboxylate: 11.4 mg, 31%; benzyl 4-acetyl-6-phenethyl-3,6-dihydropyridine-1(2*H*)-carboxylate: 11.0 mg, 31%) as colorless oils.

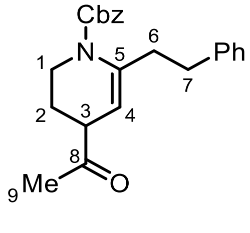

IR (thin film) ν_{max}/cm^{-1}: 2970, 2901, 1706, 1454, 1214, 913; ^{1}H NMR (500 MHz, $CDCl_3$) δ_H = 7.41 – 7.30 (m, 5H, Cbz ArCH), 7.25 – 7.20 (m, 2H, ArCH), 7.18 – 7.13 (m, 1H, ArCH), 7.07 – 7.00 (m, 2H, ArCH), 5.19 (s, 2H, Cbz CH_2), 4.94 (d, *J* = 3.6 Hz, 1H, C**4**-H), 3.68 (ddd, *J* = 12.9, 7.5, 3.3 Hz, 1H, C**1**-H), 3.43 (ddd, *J* = 12.9, 8.6, 3.1 Hz, 1H, C**1**-H'), 3.06 – 2.96 (m, 2H, C**3**-H + C**6**-H), 2.75 (dt, *J* = 14.2, 7.6 Hz, 1H, C**6**-H'), 2.70 – 2.59 (m, 2H, C**7**-H_2), 2.09 (dddd, *J* = 13.3, 7.5, 5.4, 3.1 Hz, 1H, C**2**-H), 2.02 (s, 3H, C**9**-H_3), 1.77 (dddd, *J* = 13.3, 8.6, 7.0, 3.3 Hz, 1H, C**2**-H'); ^{13}C NMR (125 MHz, $CDCl_3$)

δ_C = 207.6 (C**8**), 154.0 (Cbz C=O), 141.4 (ArC), 140.9 (C**5**), 136.3 (Cbz ArC), 128.7 (Cbz ArCH), 128.6 (Cbz ArCH), 128.4 (ArCH), 128.4 (ArCH), 128.3 (Cbz ArCH), 126.0 (ArCH), 109.3 (C**4**), 67.8 (Cbz CH_2), 47.5 (C**3**), 43.9 (C**1**), 37.5 (C**6**), 34.3 (C**7**), 27.9 (C**9**), 24.6 (C**2**); HRMS (ESI$^+$) calculated for $C_{23}H_{25}NNaO_3$ $[M+Na]^+$ = 386.1727, found 386.1727.

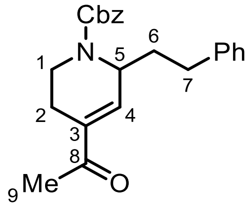

IR (thin film) ν_{max}/cm^{-1}: 2972, 2901, 1671, 1424, 1241, 892; ^{1}H NMR (500 MHz, $CDCl_3$) δ_H = 7.41 – 7.33 (m, 5H, Cbz ArCH), 7.27 – 7.02 (m, 5H, ArCH), 6.81 – 6.62 (m, 1H, C**4**-H), 5.23 – 5.12 (m, 2H, Cbz CH_2), 4.92 – 4.61 (m, 1H, C**5**-H), 4.48 – 4.16 (m, 1H, C**1**-H), 2.95 – 2.83 (m, 1H, C**1**-H'), 2.79 – 2.66 (m, 2H, C**7**-H_2), 2.52 – 2.42 (m, 1H, C**2**-H), 2.26 (s, 3H, C**9**-H_3), 2.24 – 2.13 (m, 1H, C**2**-H'), 2.05 – 1.91 (m, 2H, C**6**-H_2); ^{13}C NMR (125 MHz, $CDCl_3$) δ_C = 197.9 (C**8**), 155.3 (Cbz C=O), 141.5 (ArC), 139.9 (C**4**), 137.7 (Cbz ArC), 136.7 (C**3**), 128.7 (Cbz ArCH), 128.6 (Cbz ArCH), 128.4 (ArCH), 128.3 (Cbz ArCH), 128.0 (ArCH), 126.3 (ArCH), 67.5 (Cbz CH_2), 52.4 (C**5**), 37.5 (C**1**), 35.1 (C**6**), 32.6 (C**7**), 25.3 (C**9**), 23.4 (C**2**); HRMS (ESI$^+$) calculated for $C_{23}H_{25}NNaO_3$ $[M+Na]^+$ = 386.1727, found 386.1727.

Some signal broadening was observed due to amide-like resonance resulting in weak signal intensities in ^{13}C NMR spectrum.

Methyl (4*S*)-4-((*tert*-butyldimethylsilyl)oxy)-2-(prop-1-en-2-yl)pentanoate

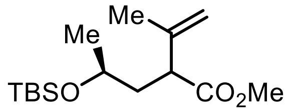

HMPA (1.68 g, 1.63 mL, 9.36 mmol) was added to a stirring solution of LDA (1.0 M in THF, 9.40 mL, 9.36 mmol) in THF (7.80 mL) at 0 °C, and the mixture was stirred for 15 min at 0 °C. After cooling to −78 °C, a mixture of methyl senecioate (890 mg, 947 μL, 7.80 mmol) in THF (7.80 mL) was added slowly, and the reaction was stirred for 1 h at −78 °C. The reaction was allowed to warm to 0 °C and stirred for 30 min. Then (*S*)-*tert*-butyl((1-iodopropan-2-yl)oxy)dimethylsilane (2.81 g, 9.36 mmol) which was freshly made from (*S*)-propylene oxide[31] was added dropwise at −78 °C. The resulting mixture was warmed to 0 °C and stirred for 30 min before warming to room temperature for stirring another 30 min. Upon completion, the mixture was poured into water (20 mL) and extracted with Et_2O (3 × 20 mL). The combined organic layer was washed with brine, dried over Na_2SO_4, filtered and concentrated in *vacuo*. The residue was purified by flash column chromatography (Hexane/EtOAc = 100/0 to 95/5) to afford the title compound (1.57 g, 70%) as a mixture of two diastereomers (1.7:1) and colorless oils. IR (thin film) ν_{max}/cm^{-1}: 2954, 2933, 2860, 1738, 1433, 1255, 836; *Diastereomer 1:* ^{1}H

NMR (500 MHz, $CDCl_3$) δ_H = 4.92 – 4.89 (m, 1H), 4.89 – 4.86 (m, 1H), 3.78 – 3.72 (m, 1H), 3.66 (s, 3H), 3.22 (ddd, *J* = 7.5, 6.7, 0.7 Hz, 1H), 2.01 – 1.94 (m, 1H), 1.73 – 1.70 (m, 3H), 1.69 – 1.62 (m, 1H), 1.13 (d, *J* = 6.1 Hz, 3H), 0.88 (s, 9H), 0.04 (s, 3H), 0.03 (s, 3H); ^{13}C NMR (125 MHz, $CDCl_3$) δ_C = 174.4, 142.5, 114.1, 66.4, 52.0, 49.4, 39.9, 26.0, 24.1, 20.4, 18.2, -4.0, -4.5; *Diastereomer 2:* ^{1}H NMR (500 MHz, $CDCl_3$) δ_H = 4.86 – 4.84 (m, 1H), 4.84 – 4.82 (m, 1H), 3.78 – 3.72 (m, 1H), 3.66 (s, 3H), 3.27 (ddd, *J* = 10.3, 4.3, 0.7 Hz, 1H), 2.01 – 1.94 (m, 1H), 1.76 – 1.73 (m, 3H), 1.57 – 1.51 (m, 1H), 1.13 (d, *J* = 6.0 Hz, 3H), 0.87 (s, 9H), 0.02 (s, 2H), 0.01 (s, 2H); ^{13}C NMR (125 MHz, $CDCl_3$) δ_C = 174.2, 143.4, 113.1, 66.6, 51.8, 49.3, 40.8, 26.0, 24.3, 20.8, 18.2, -4.1, -4.9; HRMS (ESI$^+$) calculated for $C_{15}H_{30}NaO_3Si$ $[M+Na]^+$ = 309.1856, found 309.1850.

(5*S*)-5-((*tert*-Butyldimethylsilyl)oxy)-3-(prop-1-en-2-yl)hexan-2-one

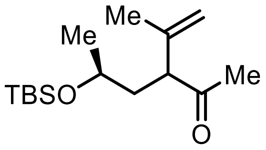

<u>General Procedure L</u>: The preceding ester (860 mg, 3.00 mmol) was employed with MeMgBr (3.0 M in Et_2O, 8.00 mL, 24.0 mmol), HN(OMe)Me·HCl (351 mg, 3.60 mmol) and NaOMe (40.5 mg, 0.75 mmol). Purification by flash column chromatography (Hexane/EtOAc = 100/0 to 95/5) afforded the title compound (644 mg, 79%) as a mixture of two diastereomers (1.7:1) and colorless oils. IR (thin film) ν_{max}/cm^{-1}: 2960, 2929, 2857, 1645, 1468, 1255, 836; *Diastereomer 1:* ^{1}H NMR (500 MHz, $CDCl_3$) δ_H = 4.99 – 4.97 (m, 1H), 4.94 – 4.92 (m, 1H), 3.76 – 3.69 (m, 1H), 3.40 – 3.32 (m, 1H), 2.13 (s, 3H), 1.90 (ddd, *J* = 14.1, 8.1, 5.8 Hz, 1H), 1.64 (s, 2H), 1.57 (ddd, *J* = 14.1, 8.0, 4.0 Hz, 1H), 1.13 – 1.09 (m, 2H), 0.88 (s, 9H), 0.04 (s, 3H), 0.04 (s, 3H); ^{13}C NMR (125 MHz, $CDCl_3$) δ_C = 208.9, 142.7, 115.3, 66.4, 57.9, 38.4, 28.2, 26.1, 24.3, 20.1, 18.2, -3.9, -4.5; *Diastereomer 2:* ^{1}H NMR (500 MHz, $CDCl_3$) δ_H = 4.92 – 4.90 (m, 1H), 4.87 – 4.84 (m, 1H), 3.76 – 3.69 (m, 1H), 3.40 – 3.32 (m, 1H), 2.12 (s, 3H), 2.01 (ddd, *J* = 9.4, 9.4, 4.7 Hz, 1H), 1.66 (s, 2H), 1.45 – 1.40 (m, 1H), 1.13 – 1.09 (m, 2H), 0.88 (s, 9H), 0.02 (s, 3H), -0.00 (s, 3H); ^{13}C NMR (125 MHz, $CDCl_3$) δ_C = 208.5, 143.6, 114.4, 66.8, 57.5, 39.2, 28.7, 26.0, 24.2, 20.4, 18.2, -4.1, -4.7; HRMS (ESI$^+$) calculated for $C_{15}H_{30}NaO_2Si$ $[M+Na]^+$ = 293.1907, found 293.1913.

(5*S*)-5-Hydroxy-3-(prop-1-en-2-yl)hexan-2-one

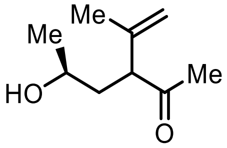

<u>General Procedure M</u>: The preceding silyl ether (406 mg, 1.50 mmol) was employed with TBAF (1 M in THF, 1.95 mL, 1.95 mmol). The residue was purified quickly by flash column chromatography

(Pentane/Et_2O = 50/50 to 25/75) giving a crude product which was taken through to the next step without further purification.

Benzyl ((2*R*)-4-acetyl-5-methylhex-5-en-2-yl)((perfluorobenzoyl)oxy)carbamate (3g)

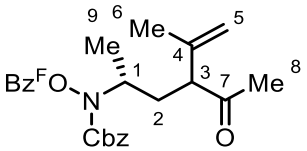

<u>General procedure N</u>: The preceding crude alcohol (172 mg, 1.10 mmol) was employed with $CbzNHO^FBz$ (361 mg, 1.00 mmol), triphenylphosphine (328 mg, 1.25 mmol) and diisopropyl azodicarboxylate (243 mg, 238 µL, 1.20 mmol). Purification by flash column chromatography (Hexane/EtOAc =95/5 to 90/10) afforded the title compound (120 mg, 16% over 2 steps) as a mixture of two diastereomers and colorless oils. IR (thin film) ν_{max}/cm^{-1}: 2958, 2923, 1714, 1524, 1327, 1182, 820; ^{1}H NMR (500 MHz, $CDCl_3$) (*diastereomer 1, rotamer A +B*) δ_H = 7.41 – 7.28 (m, 5H, Cbz ArC<u>H</u>), 5.22 (s, 2H, Cbz C<u>H</u>$_2$), 5.10 – 4.95 (m, 1H, C**5**-<u>H</u>), 4.94 – 4.82 (m, 1H, C**5**-<u>H</u>'), 4.39 – 4.23 (m, 1H, C**1**-<u>H</u>), 3.68 – 3.06 (m, 1H, C**3**-<u>H</u>), 2.22 – 1.85 (m, 4H, C**8**-<u>H</u>$_3$ + C**2**-<u>H</u>), 1.73 – 1.37 (m, 1H, C**2**-<u>H</u>'), 1.63 (s, 3H, C**6**-<u>H</u>$_3$), 1.22 (d, J = 6.6 Hz, 3H, C**9**-<u>H</u>$_3$); (*diastereomer 2, rotamer A +B*) δ_H = 7.41 – 7.28 (m, 5H, Cbz ArC<u>H</u>), 5.21 (s, 2H, Cbz C<u>H</u>$_2$), 5.10 – 4.95 (m, 1H, C**5**-<u>H</u>), 4.94 – 4.82 (m, 1H, C**5**-<u>H</u>'), 4.39 – 4.23 (m, 1H, C**1**-<u>H</u>), 3.68 – 3.06 (m, 1H, C**3**-<u>H</u>), 2.22 – 1.85 (m, 4H, C**8**-<u>H</u>$_3$ + C**2**-<u>H</u>), 1.73 – 1.37 (m, 1H, C**2**-<u>H</u>'), 1.61 (s, 3H, C**6**-<u>H</u>$_3$), 1.23 (d, J = 6.6 Hz, 3H, C**9**-<u>H</u>$_3$); ^{19}F NMR (471 MHz, $CDCl_3$) δ_F = -135.6 – -136.4 (m, 2F), -145.8 – -146.5 (m, 1F), -158.5 – -160.1 (m, 2F); ^{13}C NMR (125 MHz, $CDCl_3$) δ_C = (*diastereomer 1 and 2, rotamer A +B*) ^{13}C NMR (125 MHz, $CDCl_3$) δ 208.6 (C**7**), 208.4 (C**7**), 157.5 (Cbz <u>C</u>=O), 157.3 (Cbz <u>C</u>=O), 142.7 (C**4**), 141.4 (C**4**), 135.3 (Cbz ArC), 128.7 (Cbz ArCH), 128.6 (Cbz ArCH), 128.6 (Cbz ArCH), 128.6 (Cbz ArCH), 128.2 (Cbz ArCH), 117.5 (C**5**), 115.2 (C**5**), 68.9 (Cbz <u>C</u>H$_2$), 57.8 (C**3**), 57.0 (C**3**), 55.6 (C**1**), 54.6 (C**1**), 33.3 (C**2**), 31.1 (C**2**), 29.1 (C**8**), 28.2 (C**8**), 20.1 (C**6**), 19.5 (C**6**), 17.8 (C**9**), 17.7 (C**9**); HRMS (ESI$^+$) calculated for $C_{24}H_{22}F_5NNaO_5$ $[M+Na]^+$ = 522.1310, found 522.1310.

The carbon signals corresponding to the pentafluorobenzoyl group could not be resolved due to their weak intensity.

Benzyl (2*R*)-4-acetyl-2,6-dimethyl-3,4-dihydropyridine-1(2*H*)-carboxylate and benzyl (2*R*)-4-acetyl-2,6-dimethyl-3,6-dihydropyridine-1(2*H*)-carboxylate (4g)

<u>General procedure O</u>: The preceding substrate (49.9 mg, 0.10 mmol) was employed with $Pd_2(dba)_3$ (2.29 mg, 0.0025 mmol), CgP(2-benzofuryl) (**L3**) (8.31 mg, 0.025 mmol), CsOAc (19.2 mg, 0.10 mmol), Et_3N (14.0 µL, 0.10 mmol) and dibutyl ether (1.0 mL). The reaction was stirred at 110 °C for 42 h. Purification by flash column chromatography (Hexane/EtOAc = 97/3 to 85/15) afforded the title compounds (benzyl (2*R*)-4-acetyl-2,6-dimethyl-3,4-dihydropyridine-1(2*H*)-carboxylate: 4.20 mg, 6:1

dr, 15%; benzyl (2*R*)-4-acetyl-2,6-dimethyl-3,6-dihydropyridine-1(2*H*)-carboxylate: 13.0 mg, >15:1 dr, 45%) as colorless oils.

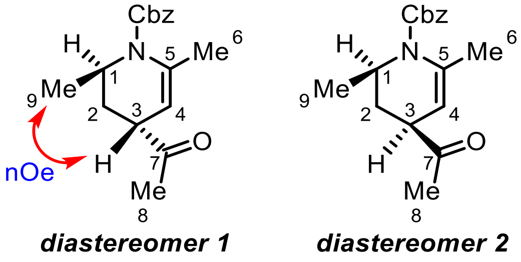

IR (thin film) ν_{max}/cm^{-1}: 2972, 2931, 1659, 1452, 1296, 761; *Diastereomer 1 (major):* ^{1}H NMR (500 MHz, CDCl$_3$) δ_H = 7.39 – 7.30 (m, 5H, Cbz ArCH), 5.18 (d, *J* = 12.2 Hz, 1H, Cbz CH$_2$), 5.13 (d, *J* = 12.2 Hz, 1H, Cbz CH'), 4.94 – 4.89 (m, 1H, C**4**-H), 4.69 (qdd, *J* = 6.8, 4.4, 2.8 Hz, 1H, C**1**-H), 3.24 – 3.12 (m, 1H, C**3**-H), 2.16 (s, 3H, C**8**-H$_3$), 2.11 (dd, *J* = 2.2, 1.3 Hz, 3H, C**6**-H$_3$), 1.94 (ddd, *J* = 13.3, 11.7, 4.4 Hz, 1H, C**2**-H), 1.82 (dddd, *J* = 13.3, 6.8, 2.8, 1.3 Hz, 1H, C**2**-H'), 1.16 (d, *J* = 6.8 Hz, 3H, C**9**-H$_3$); ^{13}C NMR (125 MHz, CDCl$_3$) δ_C = 209.1 (C**7**), 153.9 (Cbz C=O), 136.3 (Cbz ArC), 134.9 (C**5**), 128.7 (Cbz ArCH), 128.3 (Cbz ArCH), 128.2 (Cbz ArCH), 106.6 (C**4**), 67.6 (Cbz CH$_2$), 48.5 (C**1**), 45.3 (C**3**), 30.1 (C**2**), 27.6 (C**8**), 23.5 (C**6**), 16.6 (C**9**); *Diastereomer 2 (minor):* ^{1}H NMR (500 MHz, CDCl$_3$) δ_H = 7.39 – 7.30 (m, 5H, Cbz ArCH), 5.26 – 5.24 (m, 1H, C**4**-H), 5.19 (d, *J* = 12.5 Hz, 1H, Cbz ArCH), 5.13 (d, *J* = 12.5 Hz, 1H, Cbz ArCH'), 4.58 – 4.54 (m, 1H, C**1**-H), 2.92 – 2.87 (m, C**3**-H), 2.23 (s, 3H, C**8**-H$_3$), 2.13 – 2.09 (s, 3H, C**6**-H$_3$), 2.03 – 1.99 (m, 1H, C**2**-H), 1.62 – 1.61 (m, 1H, C**2**-H'), 0.98 (d, *J* = 6.7 Hz, 3H, C**9**-H$_3$); ^{13}C NMR (125 MHz CDCl$_3$) δ_C = 207.7 (C**7**), 153.9 (Cbz C=O), 136.4 (Cbz ArC), 134.4 (C**5**), 128.7 (Cbz ArCH), 128.3 (Cbz ArCH), 128.2 (Cbz ArCH), 108.6 (C**4**), 67.6 (Cbz CH$_2$), 48.1 (C**1**), 45.2 (C**3**), 29.5 (C**2**), 27.9 (C**8**), 23.1 (C**6**), 17.9 (C**9**); HRMS (ESI$^+$) calculated for $C_{17}H_{21}NNaO_3$ [M+Na]$^+$ = 310.1414, found 310.1406.

The relative stereochemistry of major diastereomer (diastereomer 1) was assigned on the basis of the nOe enhancement outlined above.

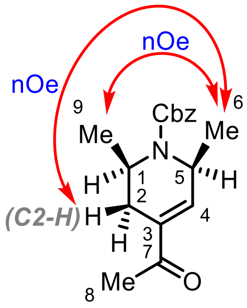

IR (thin film) ν_{max}/cm^{-1}: 2983, 2972, 1669, 1334, 1298, 878; ^{1}H NMR (500 MHz, CDCl$_3$) δ_H = 7.44 – 7.28 (m, 5H, Cbz ArCH), 6.68 (dd, *J* = 3.1, 3.1 Hz, C**4**-H), 5.19 (d, *J* = 12.5 Hz, 1H, Cbz CH), 5.16 (d, *J* = 12.5 Hz, 1H, Cbz CH'), 4.85 – 4.70 (m, 1H, C**1**-H), 4.70 – 4.58 (m, 1H, C**5**-H), 2.49 (ddd, *J* = 17.2, 1.3, 1.3 Hz, 1H, C**2**-H), 2.35 (s, 3H, C**8**-H$_3$), 2.24 (dddd, *J* = 17.2, 6.6, 3.1, 3.1 Hz, 1H, C**2**-H'), 1.39 (d, *J* = 7.1 Hz, 3H, C**6**-H$_3$), 1.11 (d, *J* = 7.0 Hz, 3H, C**9**-H$_3$); ^{13}C NMR (125 MHz CDCl$_3$) δ_C = 198.1 (C**7**),

154.8 (Cbz C=O), 139.5 (C**4**), 136.8 (Cbz ArC), 134.3 (C**3**), 128.7 (Cbz ArCH), 128.2(Cbz ArCH), 128.0 (Cbz ArCH), 67.3 (Cbz CH_2), 47.8 (C**5**), 44.1 (C**1**), 27.9 (C**2**), 25.3 (C**8**), 20.9 (C**9**), 20.6 (C**6**); HRMS (ESI^+) calculated for $C_{17}H_{21}NNaO_3$ $[M+Na]^+$ = 310.1414, found 310.1410.

Some signal broadening was observed due to amide-like resonance resulting in weak signal intensities in ^{13}C NMR spectrum.

The relative stereochemistry was assigned on the basis of the nOe enhancements outlined above.

Studies on the homologue of 1a

5-Methyleneheptan-1-ol

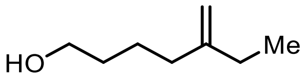

A mixture of cuprous bromide-dimethyl sulfide complex (2.30 g, 11.2 mmol) in Et_2O (10.4 mL) and dimethyl sulfide (12.5 mL) was stirred until the solution became homogeneous. The solution was cooled to -45 °C and treated with EtMgBr (3.0 M in Et_2O, 3.73 mL, 11.2 mmol) over 10 min. The mixture was stirred for 2 h at -45 °C and a solution of 6-(1-ethoxyethoxy)hex-1-yne (1.36 g, 8.00 mmol) in Et_2O (4.80 mL) was added slowly. The reaction mixture was stirred for 2 h at -45 °C, warmed to 0 °C, and quenched by addition of sat. NH_4Cl. The aqueous phase was extracted with Et_2O (3 × 30 mL). The combined organic phase was washed with brine (30 mL) and concentrated in *vacuo* to give a crude product. A solution of this crude product in acetone (12.8 mL), water (4.50 mL) and 2 drops of concentrated sulfuric acid was heated at reflux for 2 h. Upon completion, acetone was evaporated in *vacuo*, and 12 mL of water was added. The aqueous layer was extracted with (3 × 30 mL). The combined organic phase was washed with sat. $NaHCO_3$ solution, brine (30 mL), dried over Na_2SO_4, filtered and concentrated in *vacuo*. The residue was purified by flash column chromatography (Hexane/Et_2O = 50/50 to 40/60) to afford the title compound (366 mg, 36%, over 2 steps) as a colorless oil. ^1H NMR (500 MHz, $CDCl_3$) δ_H = 4.79 – 4.58 (m, 2H), 3.64 (t, J = 6.4 Hz, 2H), 2.10 – 1.94 (m, 4H), 1.73 (br s, 1H), 1.60 – 1.41 (m, 4H), 1.02 (t, J = 7.4 Hz, 3H); ^{13}C NMR (125 MHz, $CDCl_3$) δ_C = 151.4, 107.8, 63.0, 36.1, 32.6, 28.8, 24.0, 12.5.

The spectroscopic properties were consistent with the data available in the literature.[32]

Benzyl (5-methyleneheptyl)((perfluorobenzoyl)oxy)carbamate

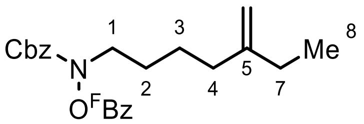

General procedure N: The preceding alcohol (256 mg, 2.00 mmol) was employed with $CbzNHO^FBz$ (867 mg, 2.40 mmol), triphenylphosphine (629 mg, 2.40 mmol) and diisopropyl azodicarboxylate (485 mg, 476 µL, 2.40 mmol). Purification by flash column chromatography (Hexane/EtOAc = 100/0 to

98/2) afforded the title compound (370 mg, 39%) as a colorless oil. IR (thin film) ν_{max}/cm^{-1}: 2966, 2939, 1784, 1650, 1326, 906; ^{1}H NMR (500 MHz, $CDCl_3$) δ_H = 7.41 – 7.30 (m, 5H, Cbz ArCH), 5.22 (s, 2H, Cbz CH_2), 4.75 – 4.69 (m, 1H, C**6**-H), 4.69 – 4.62 (m, 1H, C**6**-H'), 3.76 (t, J = 7.1 Hz, 2H, C**1**-H_2), 2.08 – 1.97 (m, 4H, C**4**-H_2 + C**7**-H_2), 1.69 – 1.61 (m, 2H, C**2**-H_2), 1.56 – 1.46 (m, 2H, C**3**-H_2), 1.01 (t, J = 7.4 Hz, 3H, C**8**-H_3). ^{19}F NMR (377 MHz, $CDCl_3$) δ_F = -135.5 – -136.8 (m, 2F), -146.2 (tt, J = 21.1, 5.3 Hz), -158.3 – -160.3 (m, 2F); ^{13}C NMR (125 MHz, $CDCl_3$) δ_C = 155.5 (C=O), 150.9 (C**5**), 135.4 (Cbz ArC), 128.7 (Cbz ArCH), 128.6 (Cbz ArCH), 128.3 (Cbz ArCH), 108.1 (C**6**), 68.9 (Cbz CH_2), 51.2 (C**1**), 35.8 (C**4**), 28.7 (C**7**), 26.6 (C**2**), 24.6 (C**3**), 12.4 (C**8**); HRMS (ESI$^+$) calculated for $C_{23}H_{22}F_5NNaO_4$ $[M+Na]^+$ = 494.1361, found 494.1370.

The carbon signals corresponding to the pentafluorobenzoyl group could not be resolved due to their weak intensity.

Catalysis reaction and results for benzyl (5-methyleneheptyl)((perfluorobenzoyl)oxy)carbamate

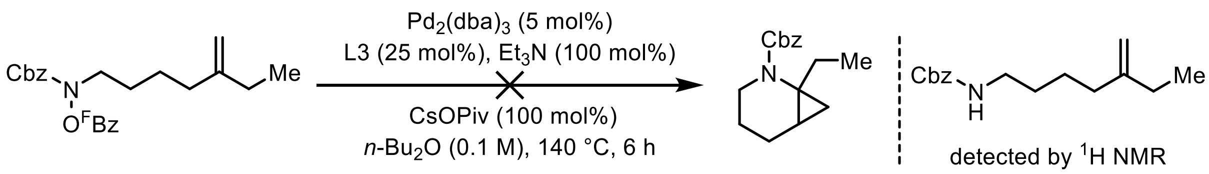

<u>General procedure O</u>: The preceding substrate (47.1 mg, 0.10 mmol) was employed with $Pd_2(dba)_3$ (4.58 mg, 0.005 mmol), CgP(2-benzofuryl) (**L3**) (8.31 mg, 0.025 mmol), CsOPiv (23.4 mg, 0.10 mmol), Et_3N (14.0 µL, 0.10 mmol) and dibutyl ether (1.0 mL). The reaction was stirred at 140 °C for 6 h. Unpon completion, the reaction mixture was passed through a plug of silica, washed with EtOAc (3.00 mL) and concentrated in *vacuo*. The ^{1}H NMR spectrum of the crude mixture indicated the formation of the NH carbamate shown above.

Deuterium-labeling experiment for 1k to *iso*-2k

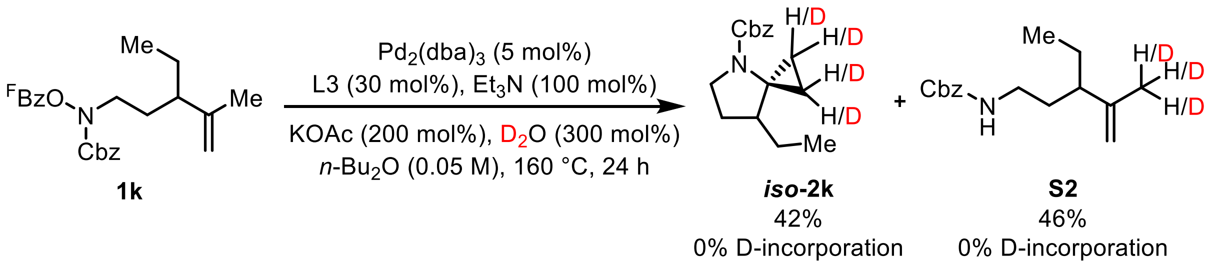

<u>General procedure O</u>: **1k** (47.1 mg, 0.10 mmol) was employed with $Pd_2(dba)_3$ (4.58 mg, 0.005 mmol), CgP(2-benzofuryl) (**L3**) (9.97 mg, 0.03 mmol), KOAc (19.6 mg, 0.20 mmol), Et_3N (14.0 µL, 0.10 mmol) D_2O (6.00 mg, 0.20 mmol) and dibutyl ether (2.0 mL). The reaction was stirred at 160 °C for 24 h. Purification by flash column chromatography (Hexane/EtOAc = 100/0 to 90/10) afforded ***iso*-1k** (11.0 mg, 42%) as a colorless oil and **S2** (11.9 mg, 46%) as a colorless oil.

__Representative 2D NMR studies__

Assigned COSY, HSQC (blue refers to CH_2, red refers to CH/CH_3) and HMBC for benzyl 7-ethyl-4-azaspiro[2.4]heptane-4-carboxylate (*iso*-2k)

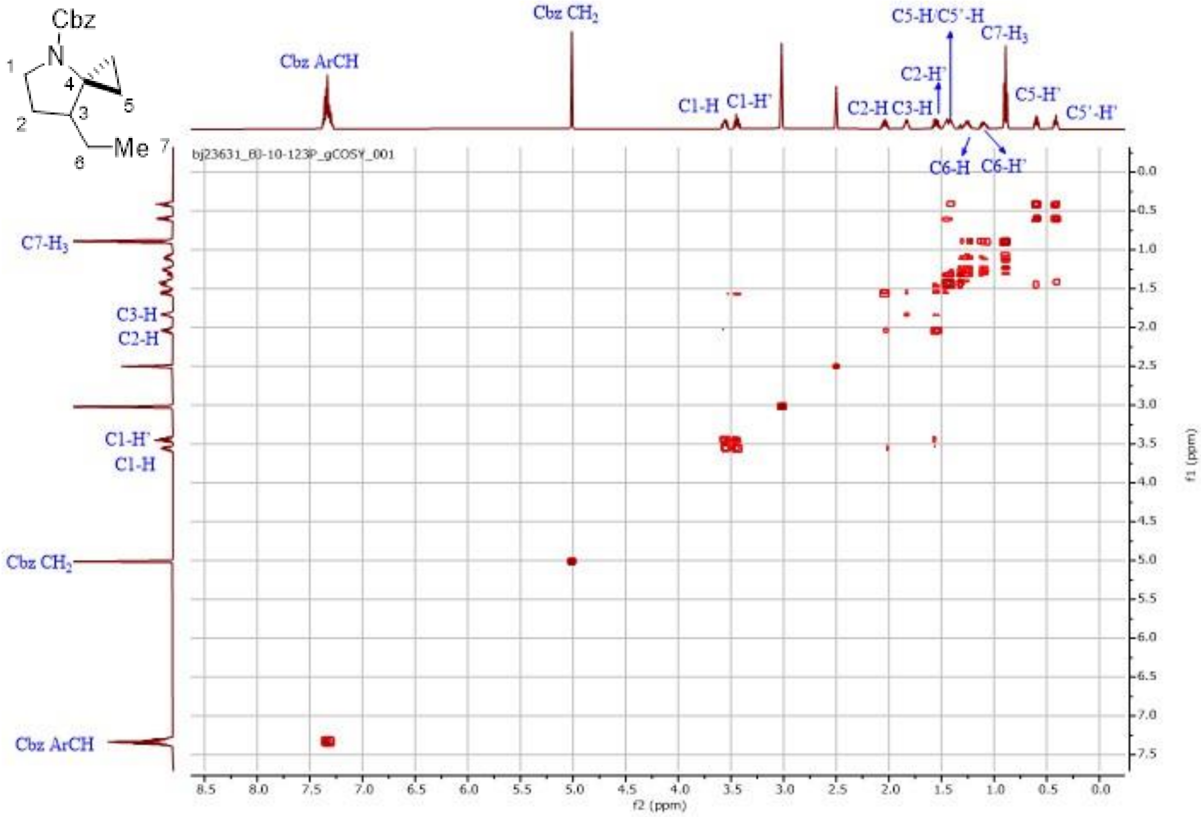

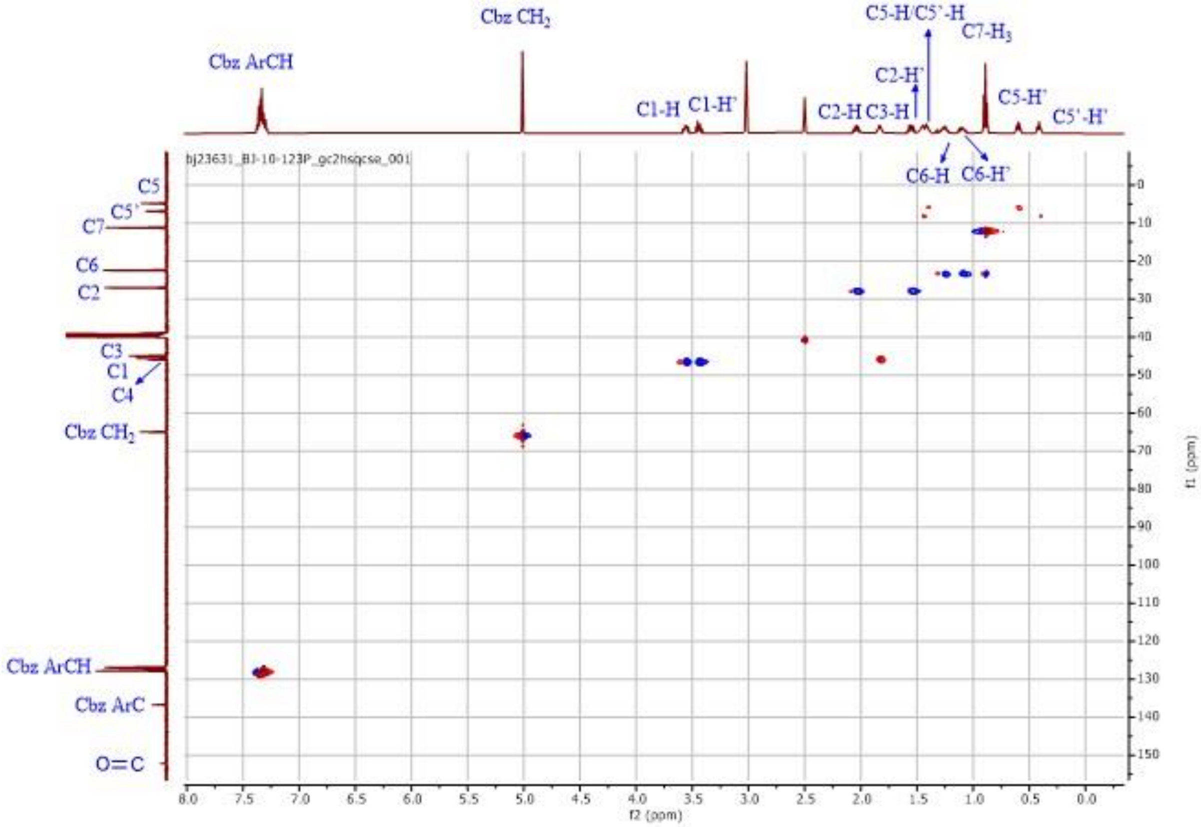

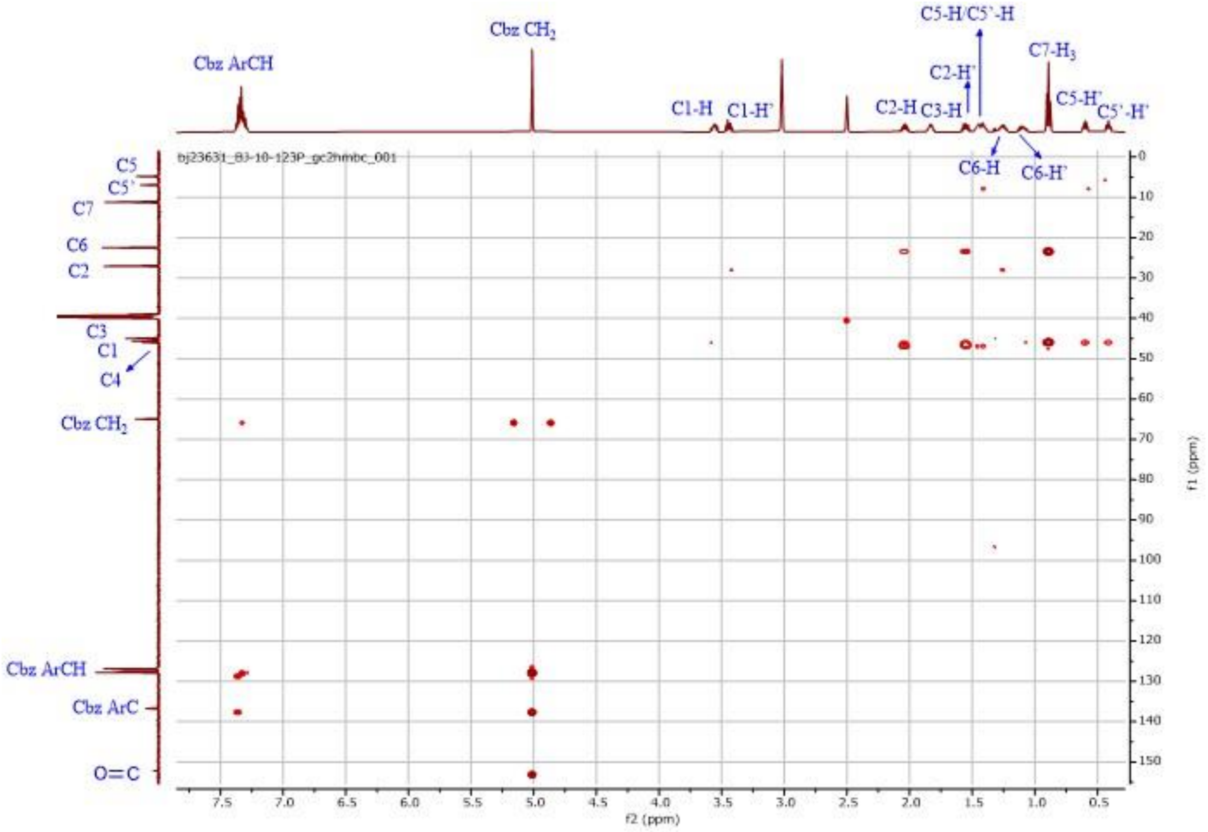

Assigned COSY, HSQC (blue refers to CH/CH$_3$, red refers to CH$_2$) and HMBC for benzyl 1a-ethyl-1,1a,3,7b-tetrahydro-2*H*-cyclopropa[*c*]isoquinoline-2-carboxylate (2n)

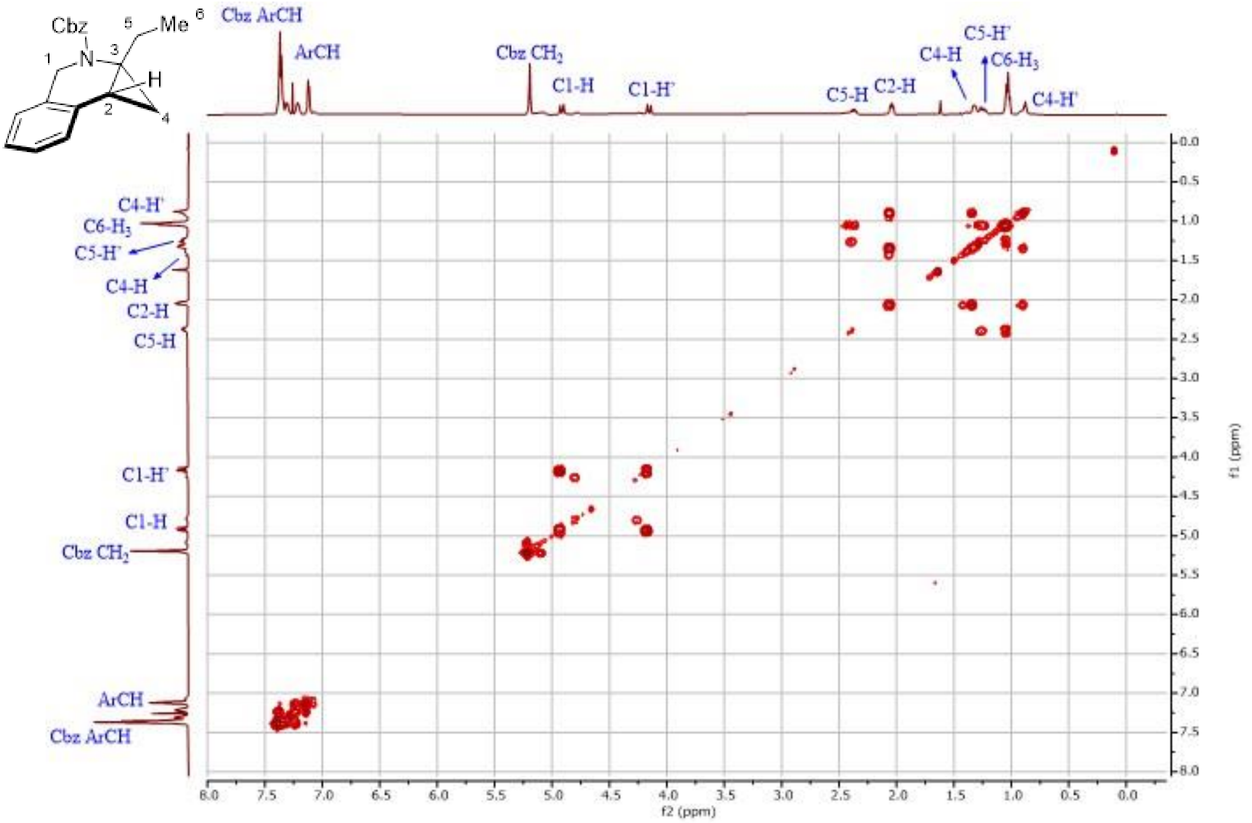

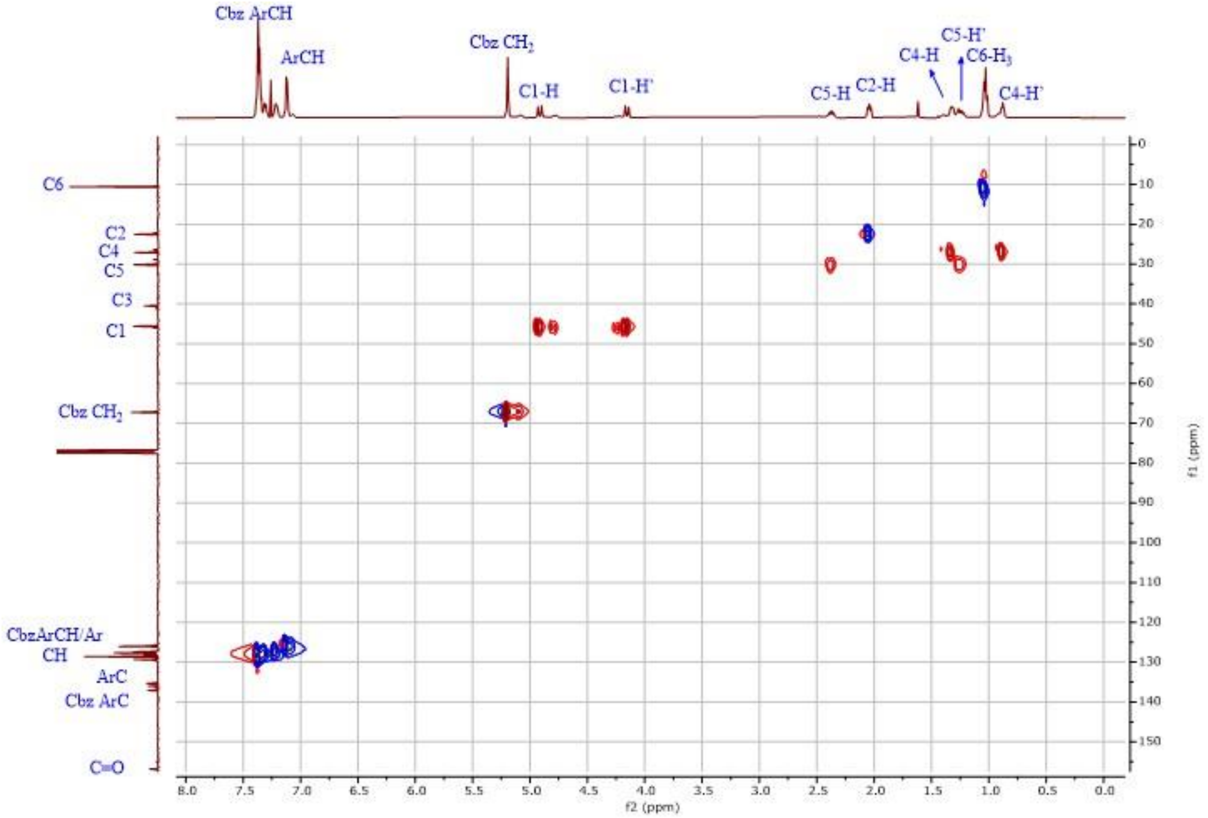
Cbz ArCH
ArCH
Cbz CH2
C1-H
C1-H'
C5-H
C2-H
C4-H
C5-H'
C6-H3
C4-H'
C6
C2
C4
C5
C3
C1
Cbz CH2
CbzArCH/Ar
CH
ArC
Cbz ArC
C=O
f2 (ppm)
f1 (ppm)

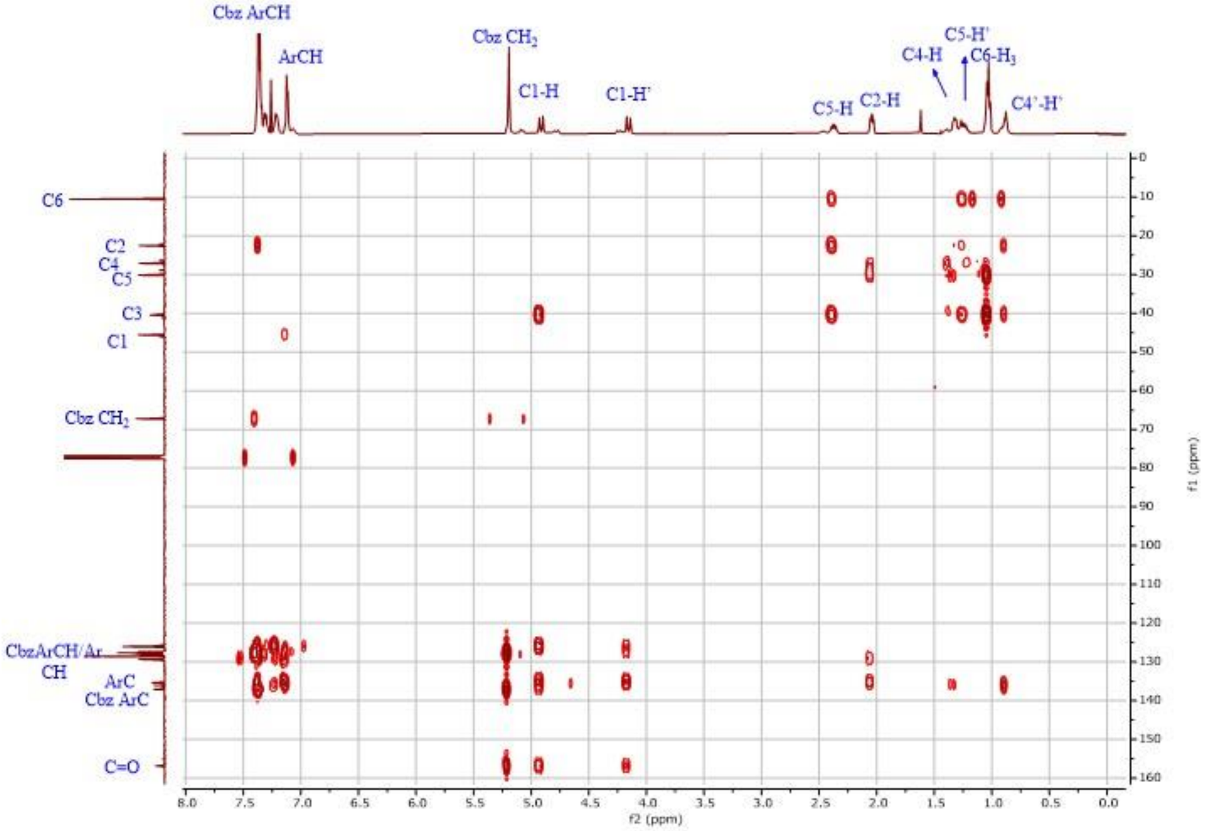
Cbz ArCH
ArCH
Cbz CH2
C1-H
C1-H'
C5-H
C2-H
C4-H
C5-H'
C6-H3
C4'-H'
C6
C2
C4
C5
C3
C1
Cbz CH2
CbzArCH/Ar
CH
ArC
Cbz ArC
C=O
f2 (ppm)
f1 (ppm)

Assigned COSY, HSQC (blue refers to CH_2, red refers to CH/CH_3) and HMBC for benzyl 4-acetyl-6-methyl-3,4-dihydropyridine-1(2*H*)-carboxylate and benzyl 4-acetyl-6-methyl-3,6-dihydropyridine-1(2*H*)-carboxylate (4a)

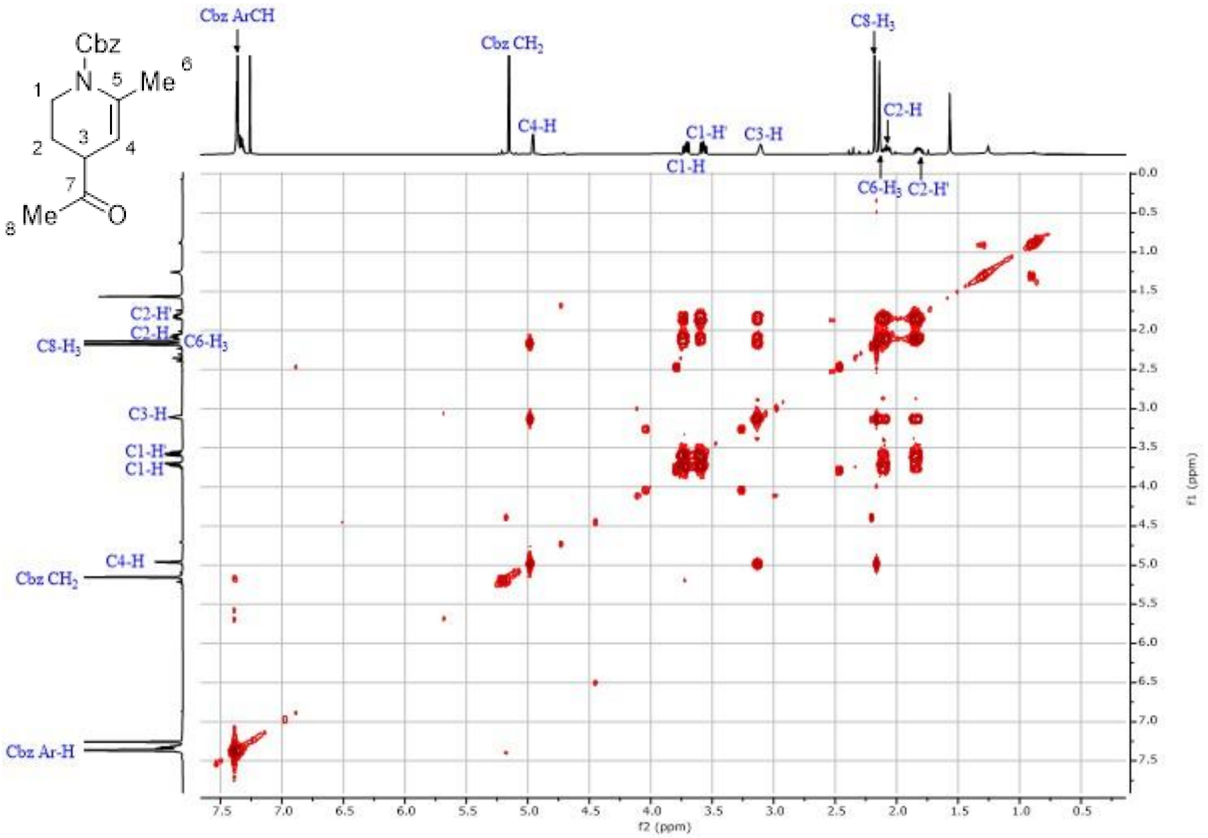

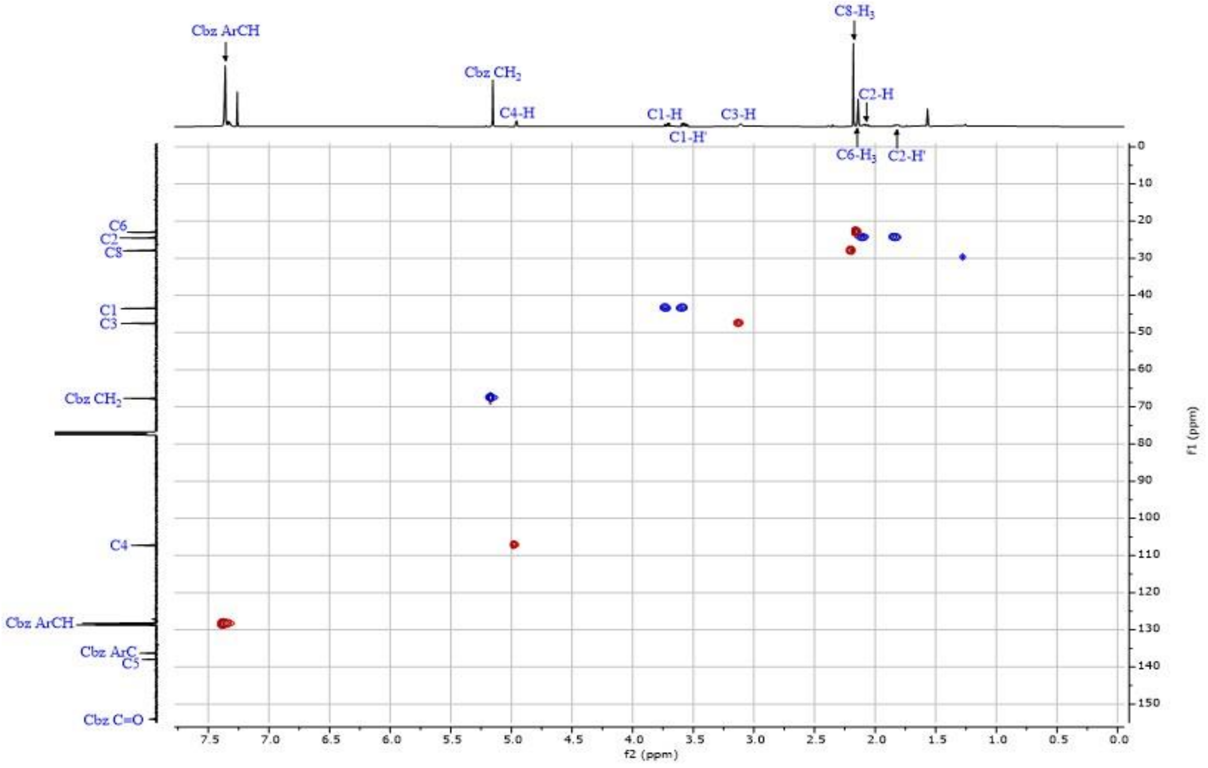

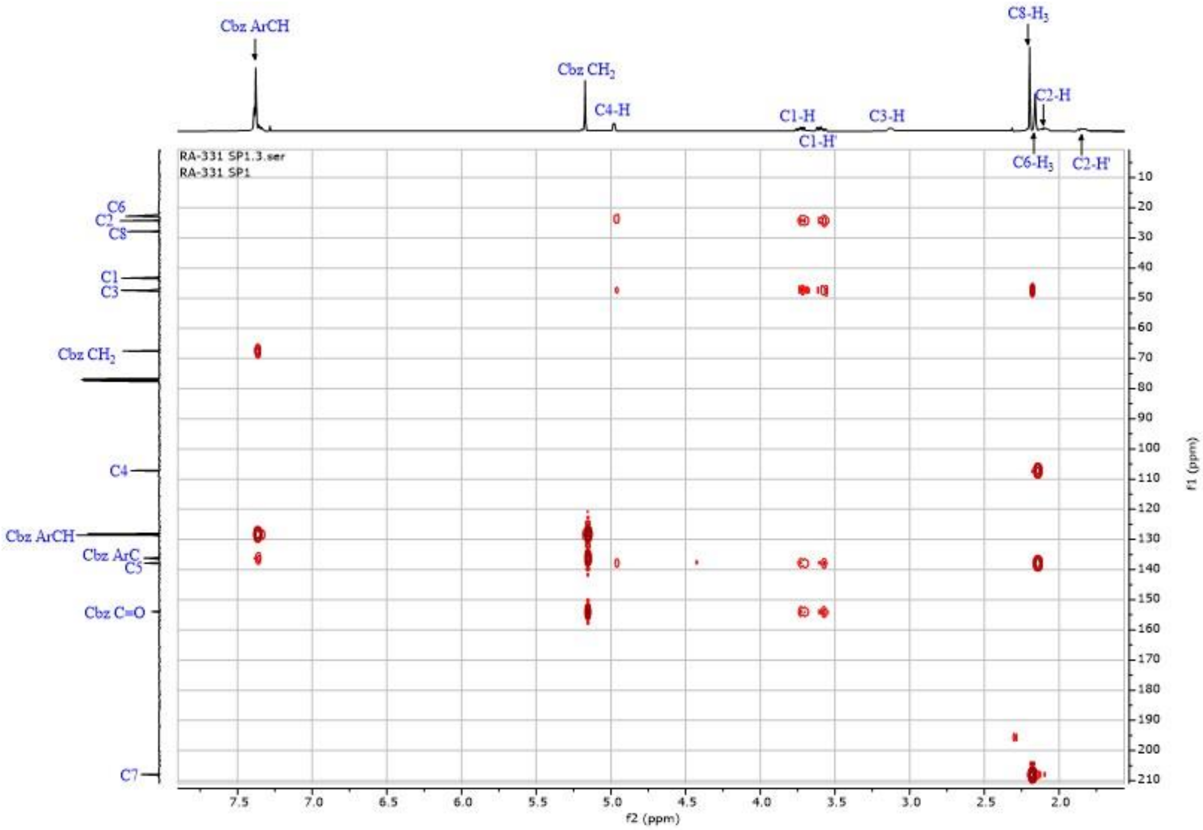
RA-331 SP1.3.ser
RA-331 SP1
Cbz ArCH
Cbz CH2
C4-H
C1-H
C1-H'
C3-H
C8-H3
C2-H
C6-H3
C2-H'
C6
C2
C8
C1
C3
Cbz CH2
C4
Cbz ArCH
Cbz ArC
C5
Cbz C=O
C7
f2 (ppm)
f1 (ppm)

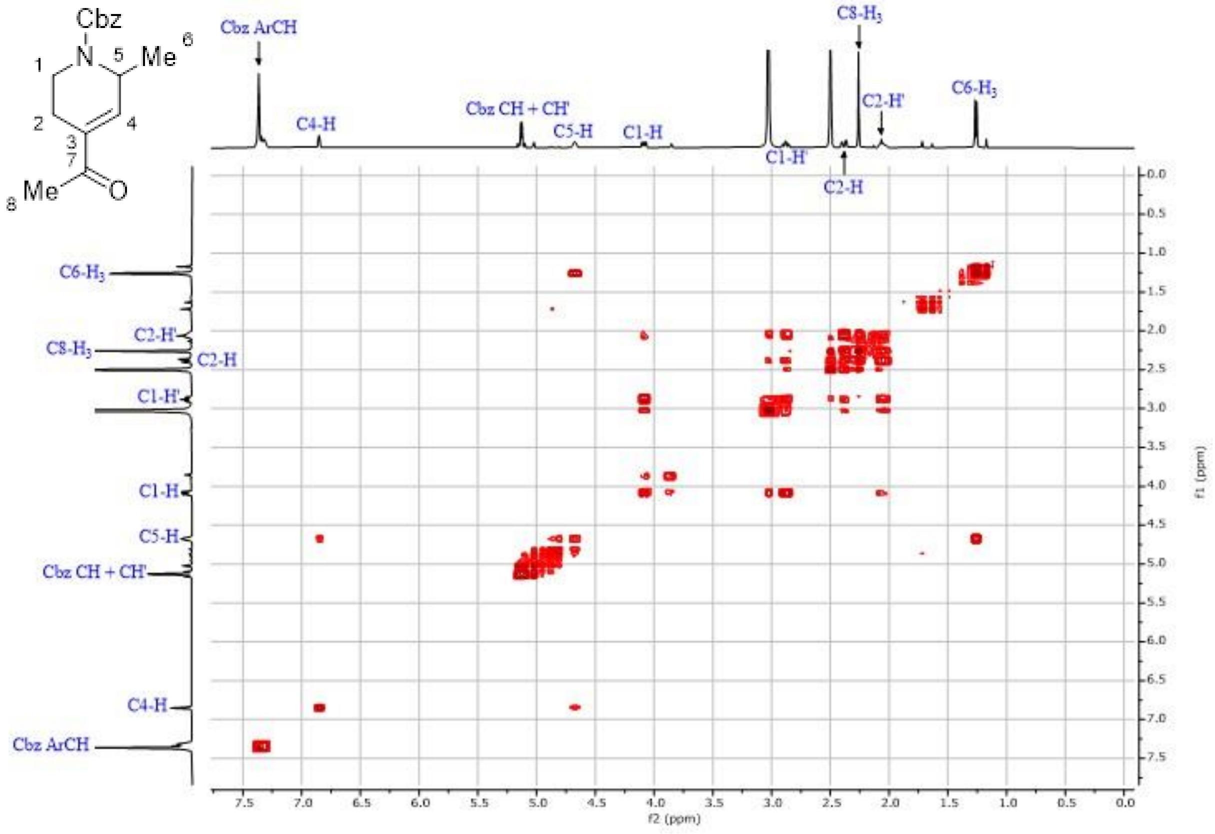
Cbz
N
Me
Me
O
1
2
3
4
5
6
7
8
Cbz ArCH
C4-H
Cbz CH + CH'
C5-H
C1-H
C1-H'
C2-H
C8-H3
C2-H'
C6-H3
f2 (ppm)
f1 (ppm)

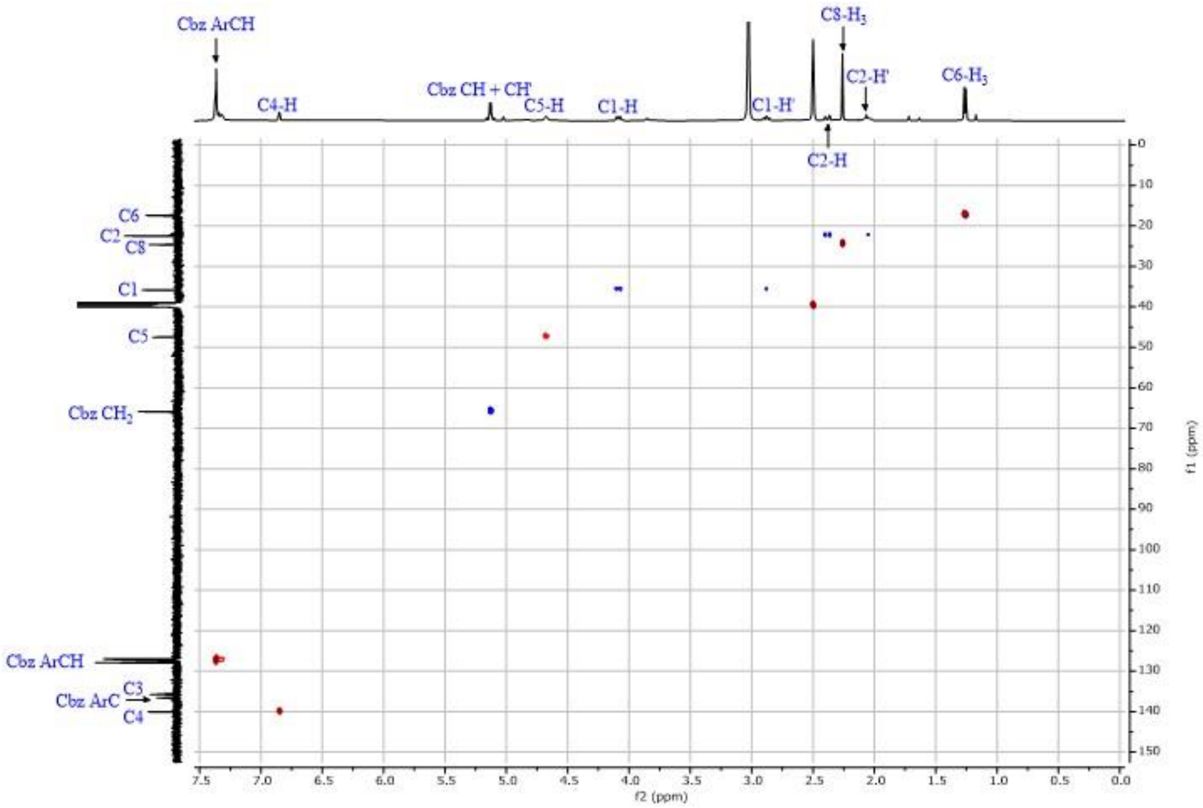
Cbz ArCH
C4-H
Cbz CH + CH'
C5-H
C1-H
C1-H'
C8-H3
C2-H'
C2-H
C6-H3
C6
C2
C8
C1
C5
Cbz CH2
Cbz ArCH
C3
Cbz ArC
C4
f2 (ppm)
f1 (ppm)

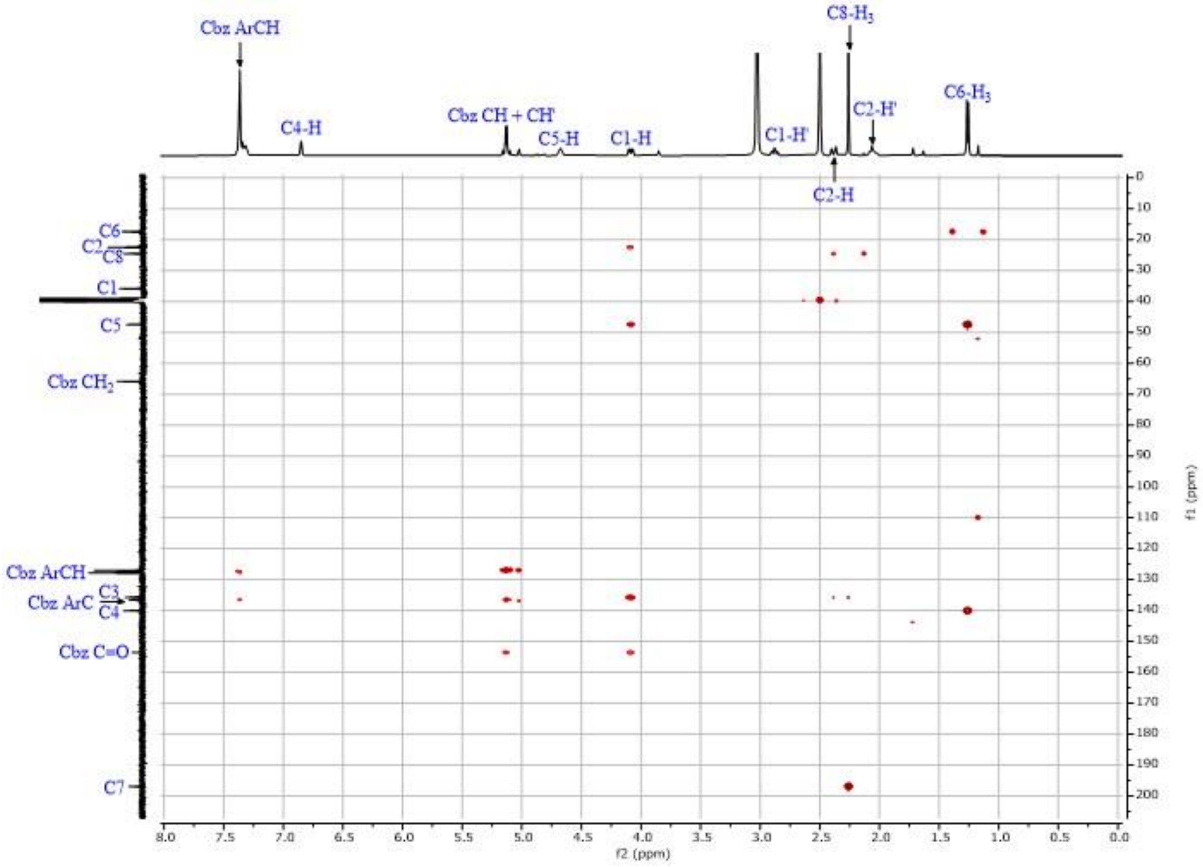
Cbz ArCH
C4-H
Cbz CH + CH'
C5-H
C1-H
C1-H'
C8-H3
C2-H'
C2-H
C6-H3
C6
C2
C8
C1
C5
Cbz CH2
Cbz ArCH
C3
Cbz ArC
C4
Cbz C=O
C7
f2 (ppm)
f1 (ppm)

NMR Spectra for Novel Compounds

1,3,5,7-Tetramethyl-8-(thiophen-2-yl)-2,4,6-trioxa-8-phosphaadamantane

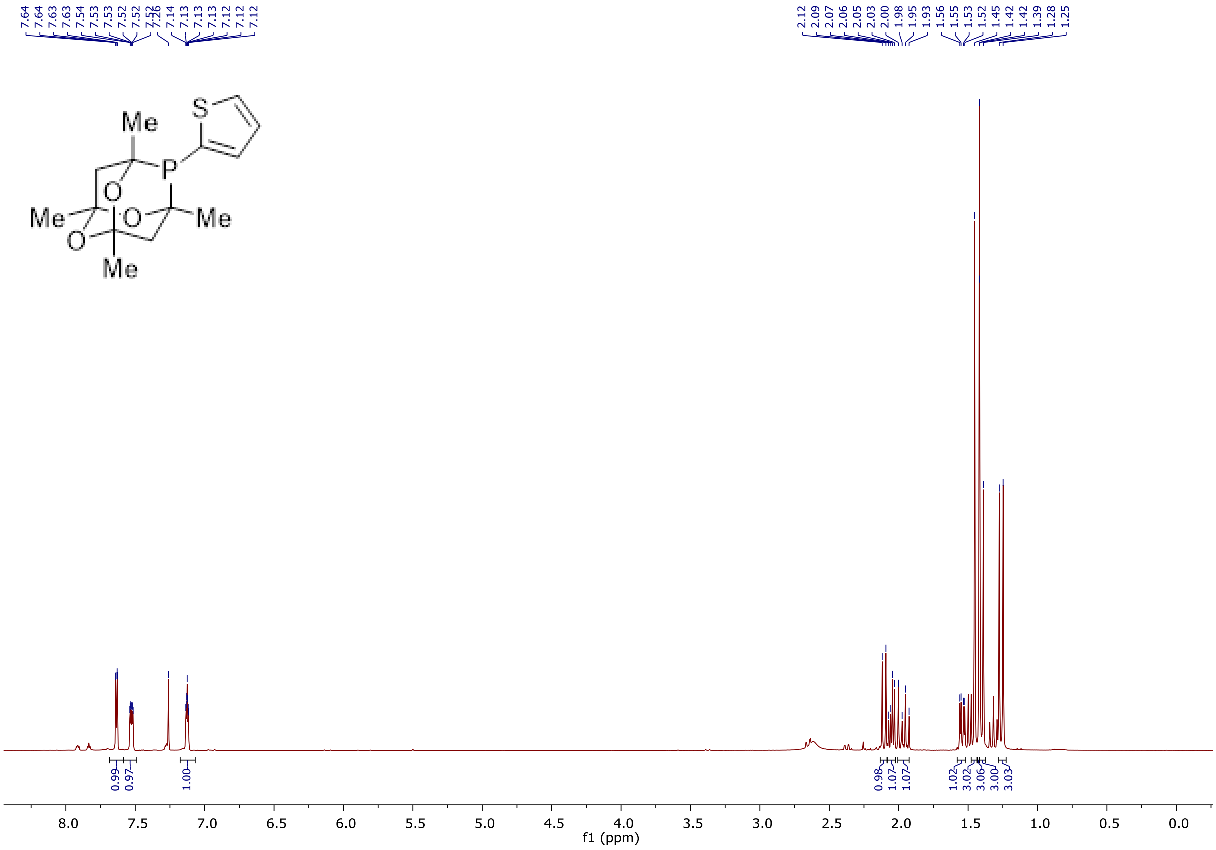

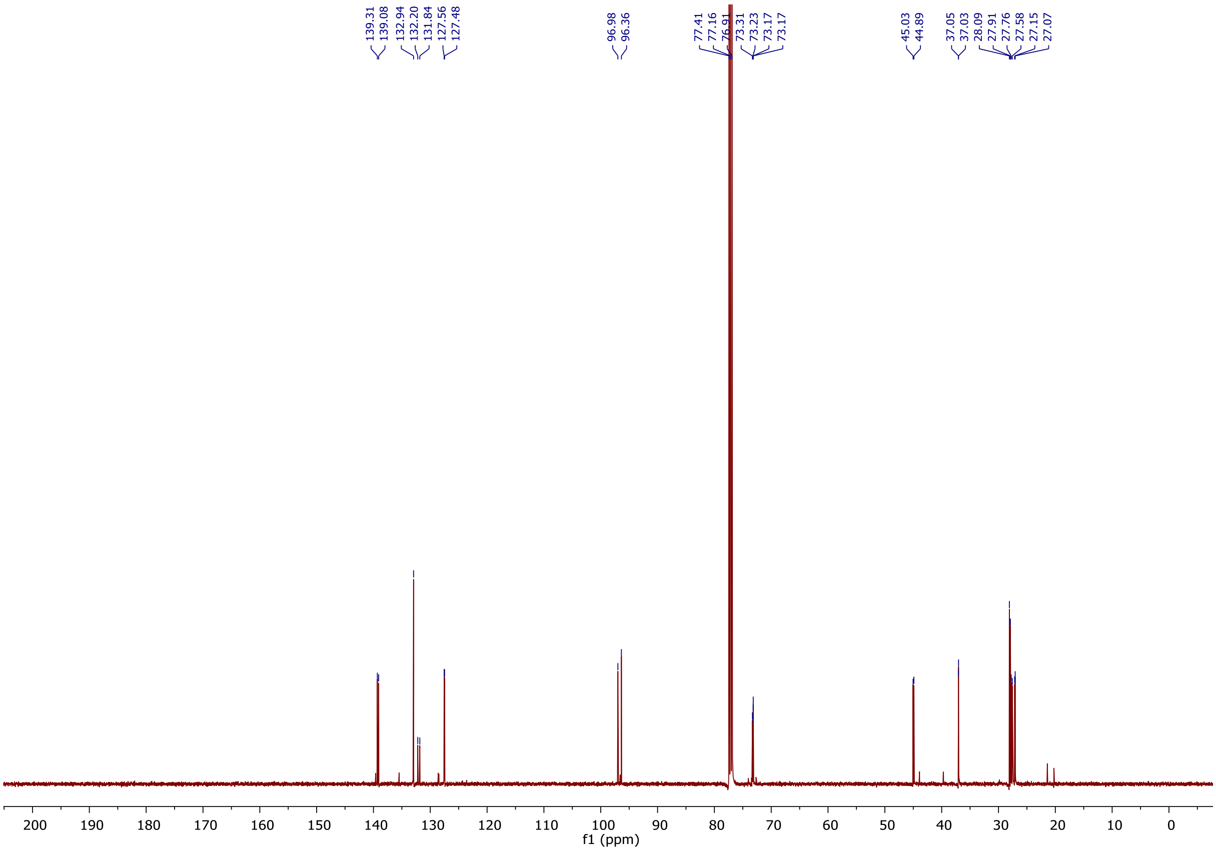

1,3,5,7-Tetramethyl-8-(5-acetylthiophen-2-yl)-2,4,6-trioxa-8-phosphaadamantane

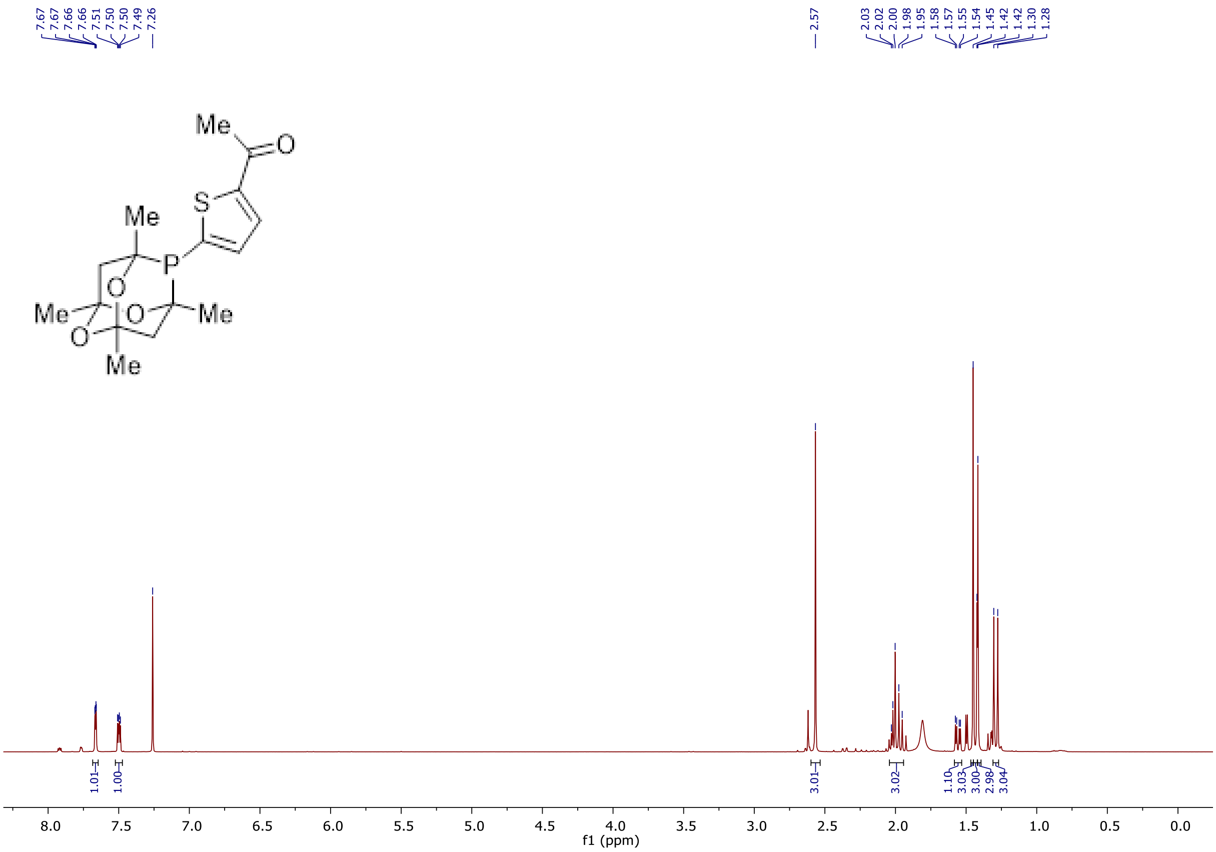

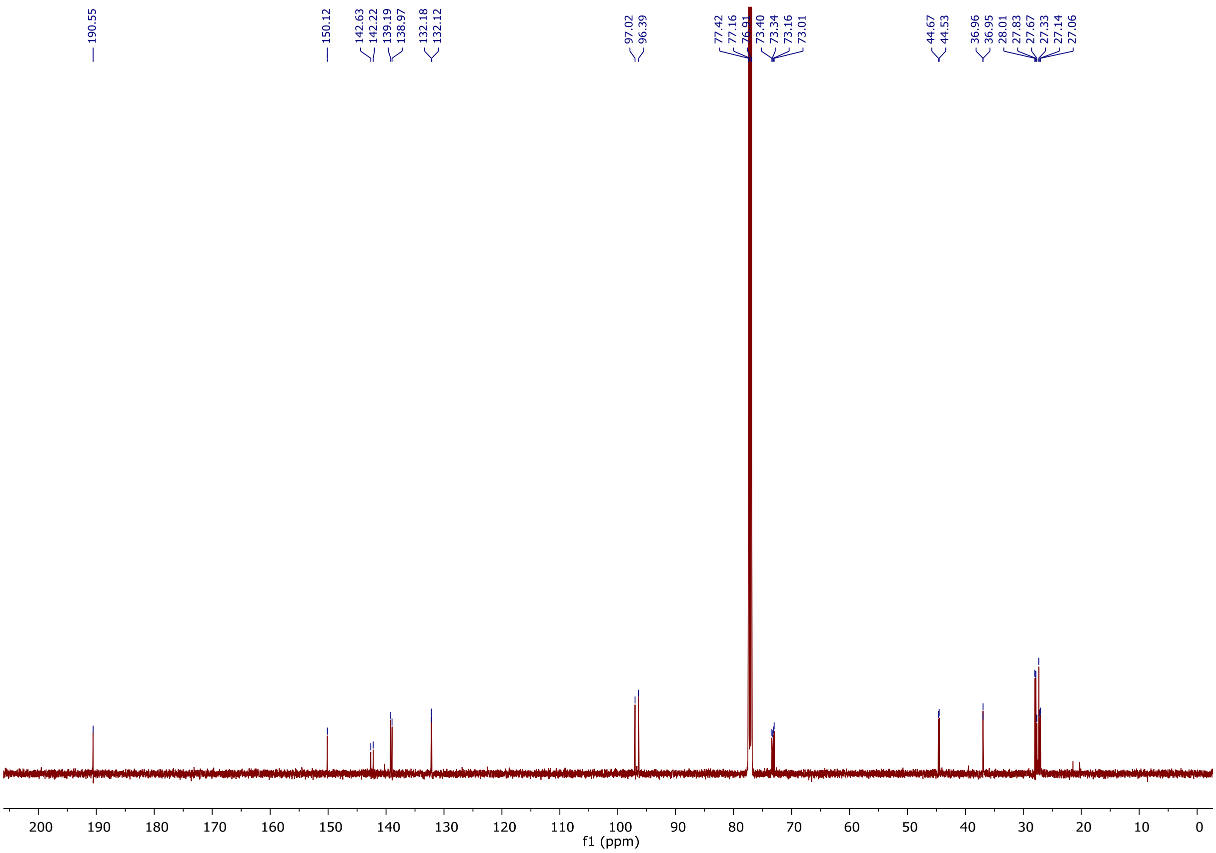

8-(Furan-2-yl)-1,3,5,7-tetramethyl-2,4,6-trioxa-8-phosphaadamantane

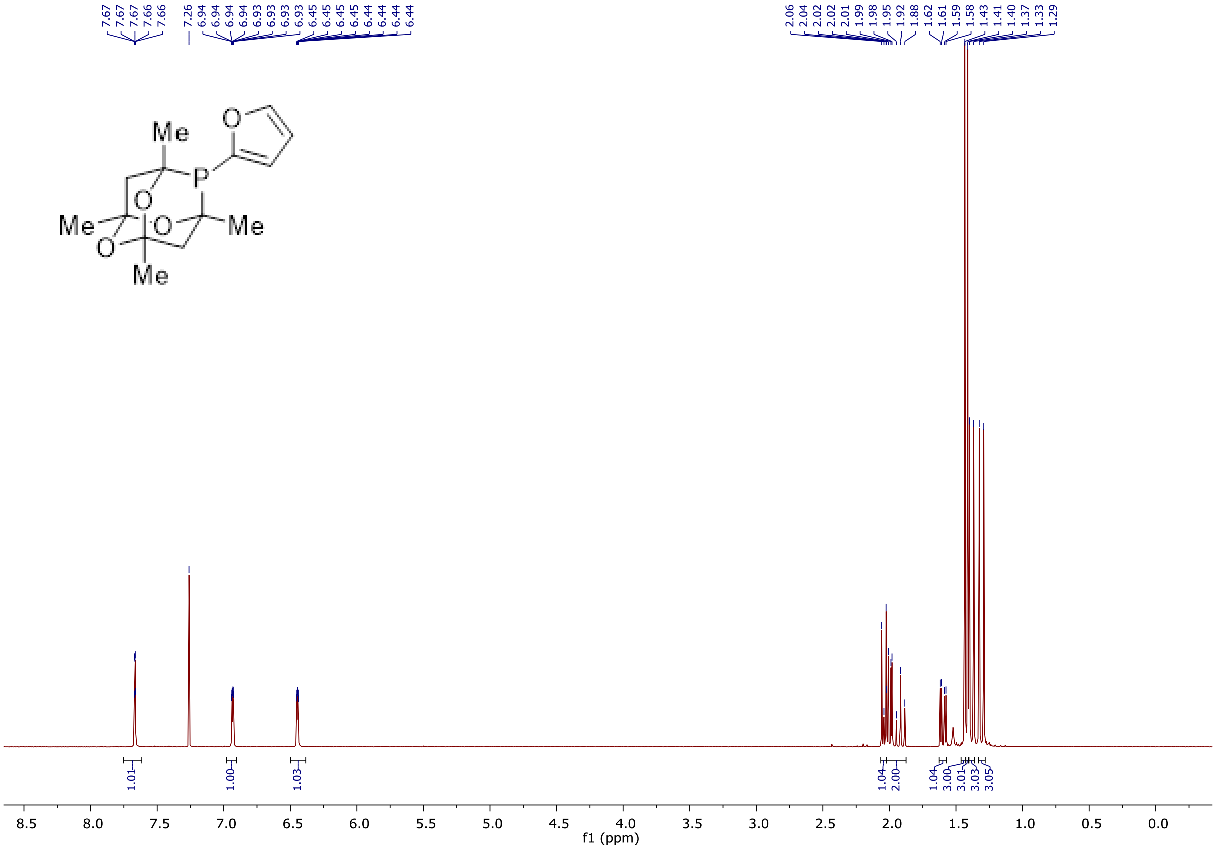

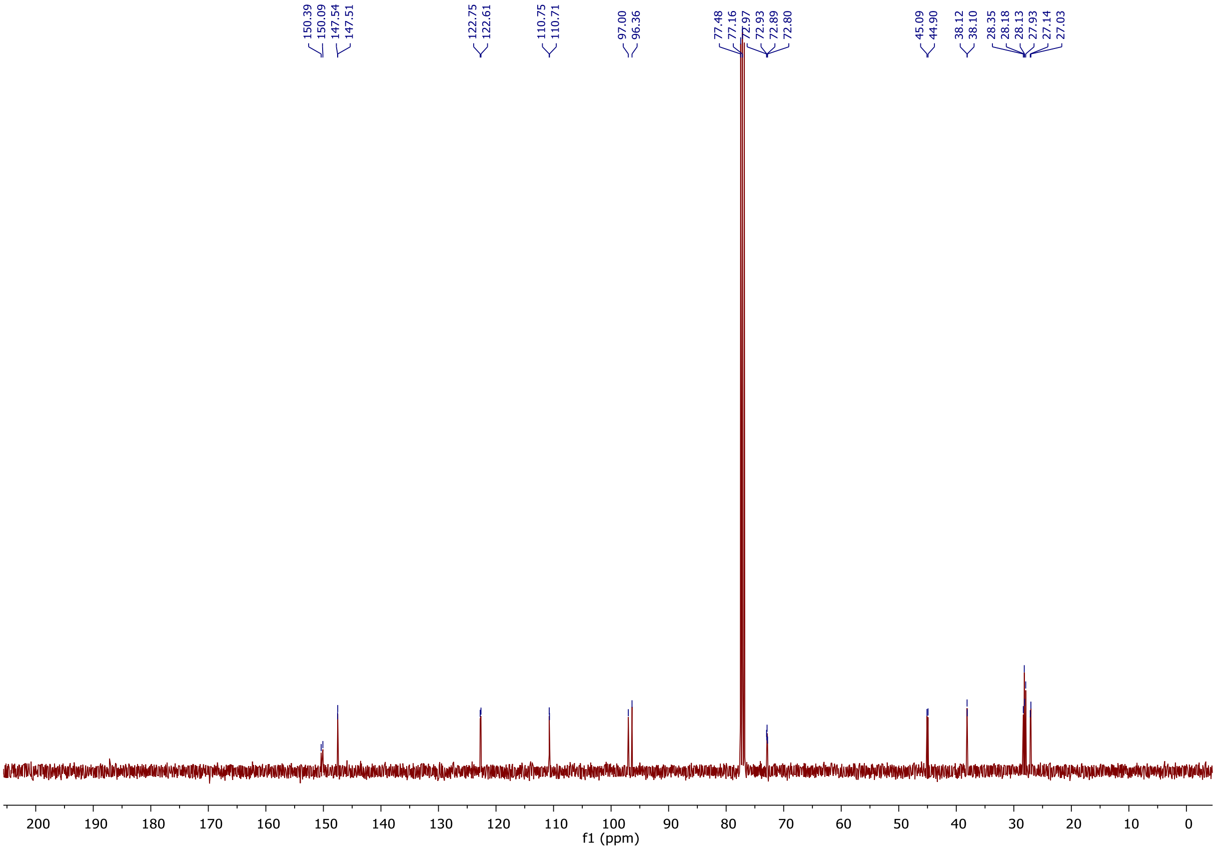

8-(5-Ethylfuran-2-yl)-1,3,5,7-tetramethyl-2,4,6-trioxa-8-phosphaadamantane

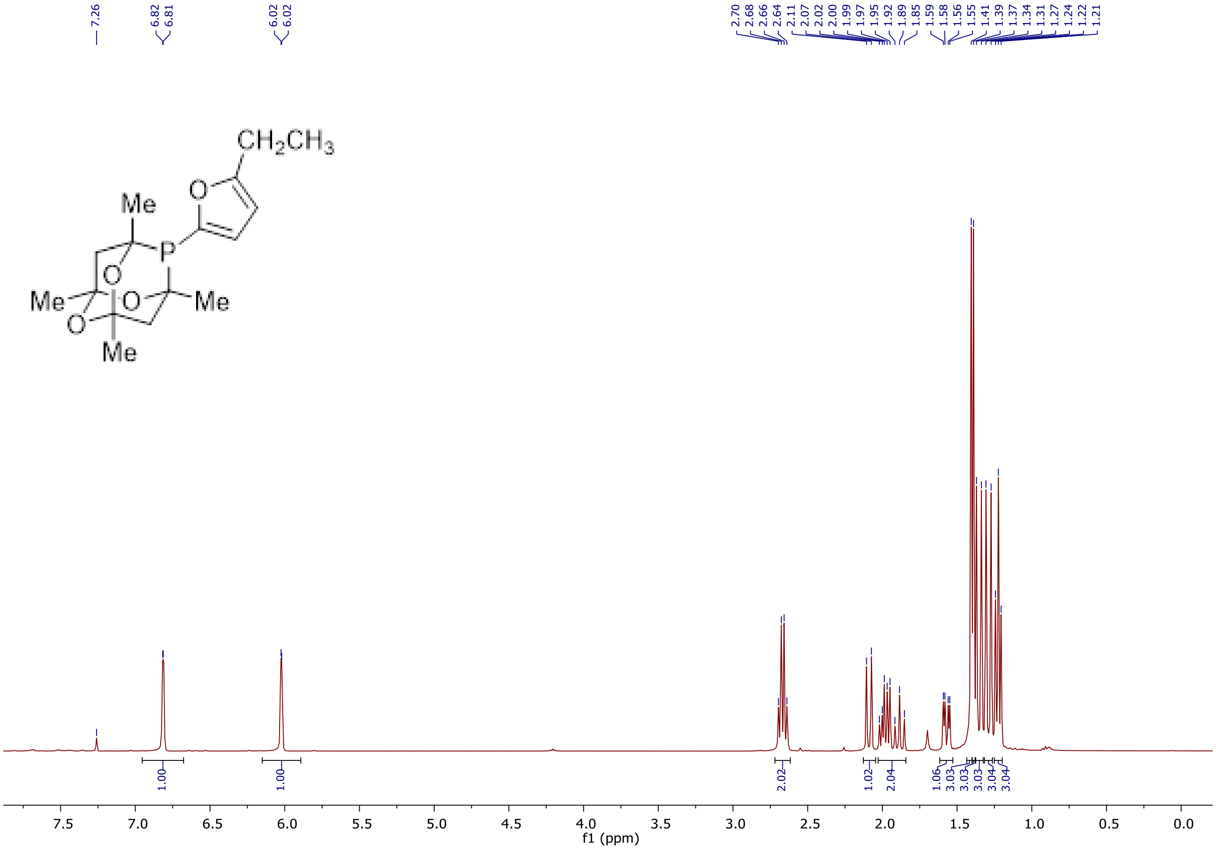

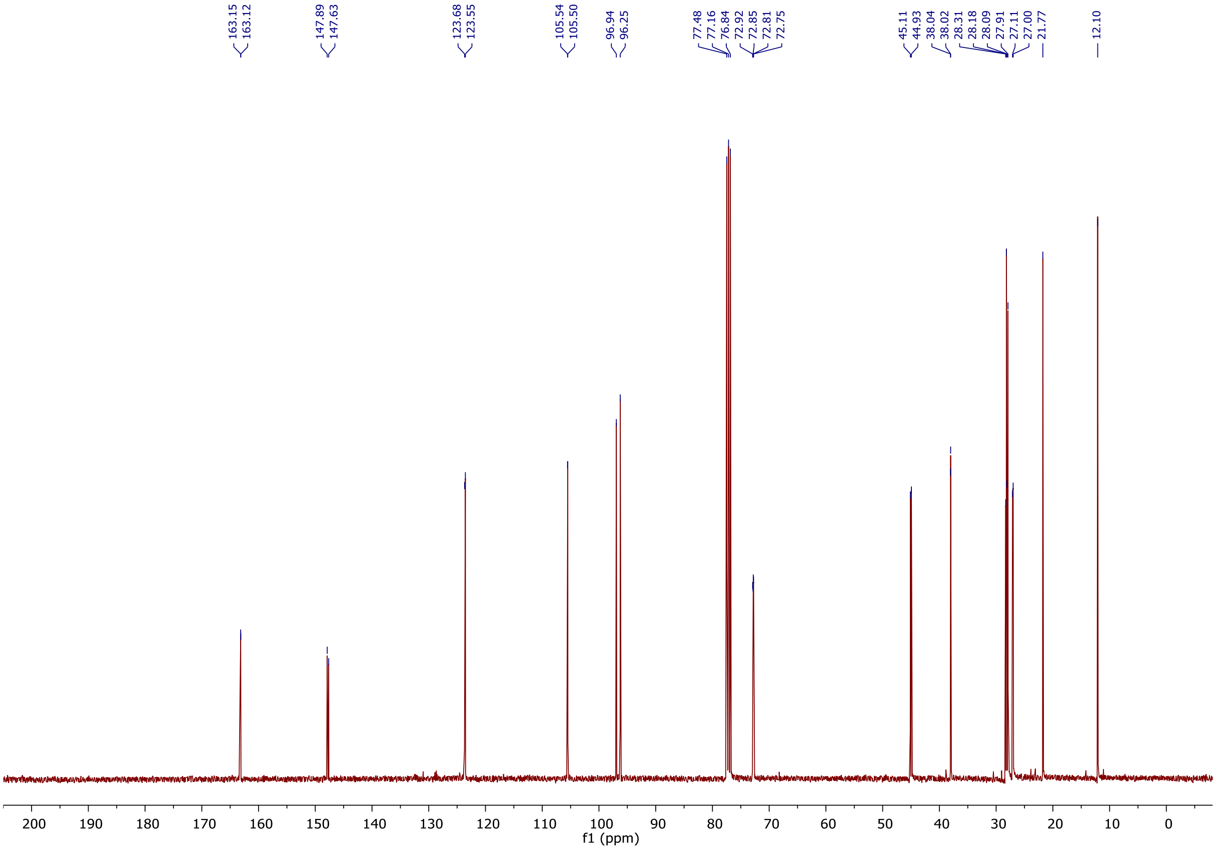

1-(5-(1,3,5,7-Tetramethyl-2,4,6-trioxa-8-phosphaadamantan-8-yl)furan-2-yl)ethan-1-one

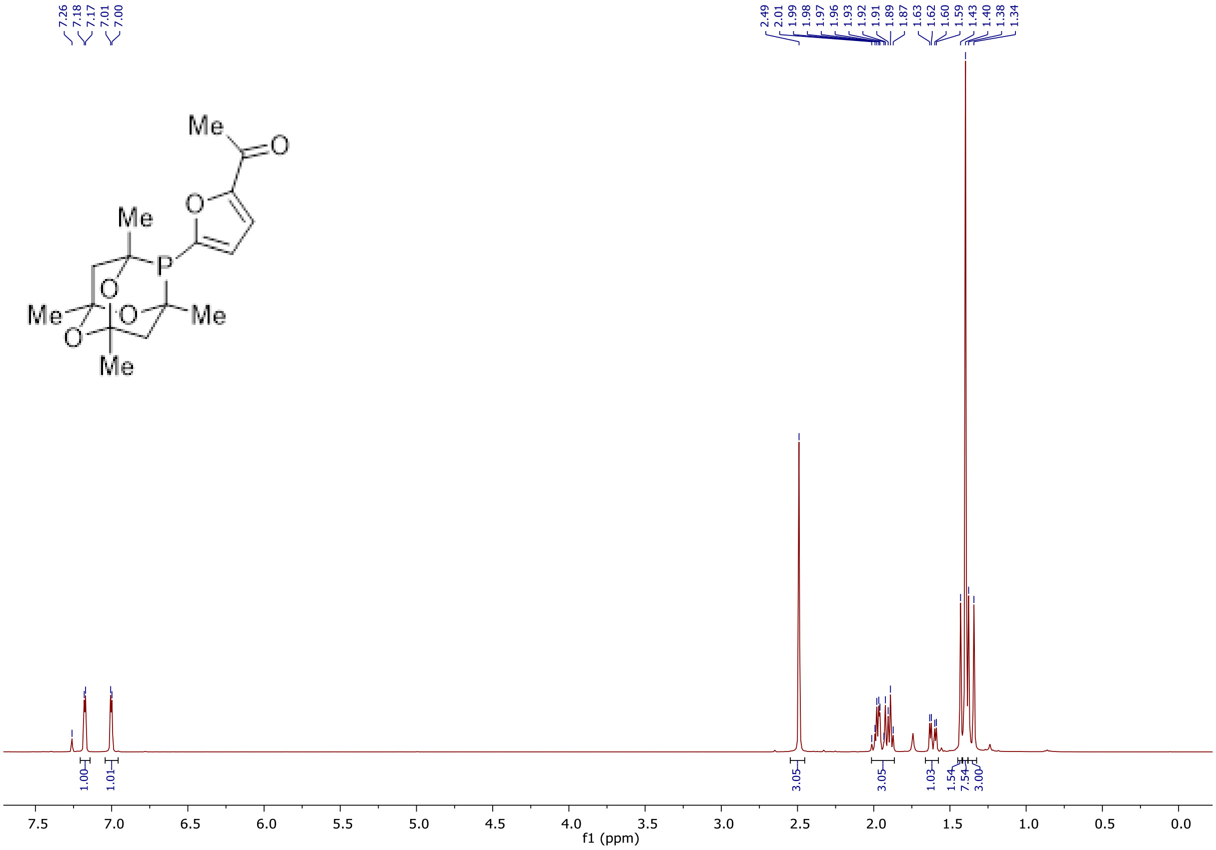

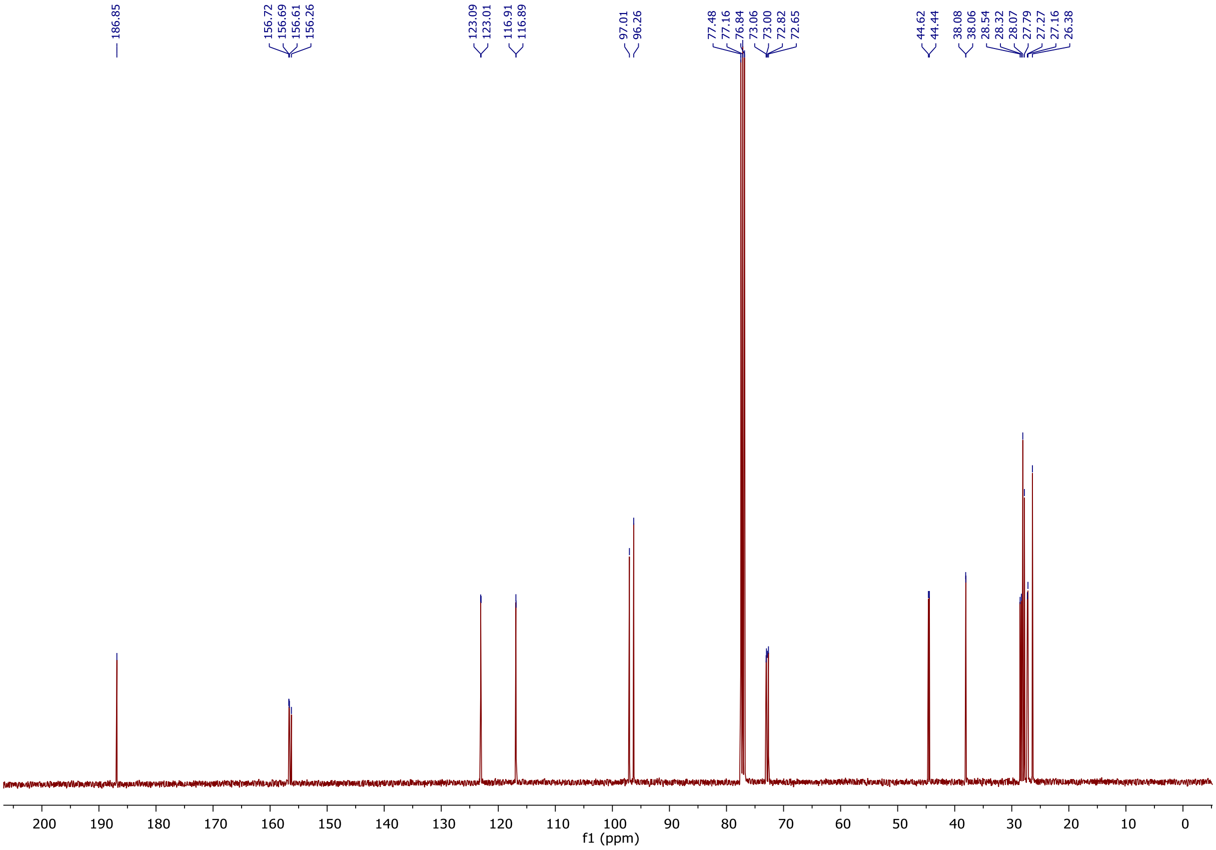

Methyl 5-(1,3,5,7-tetramethyl-2,4,6-trioxa-8-phosphaadamantan-8-yl)furan-2-carboxylate

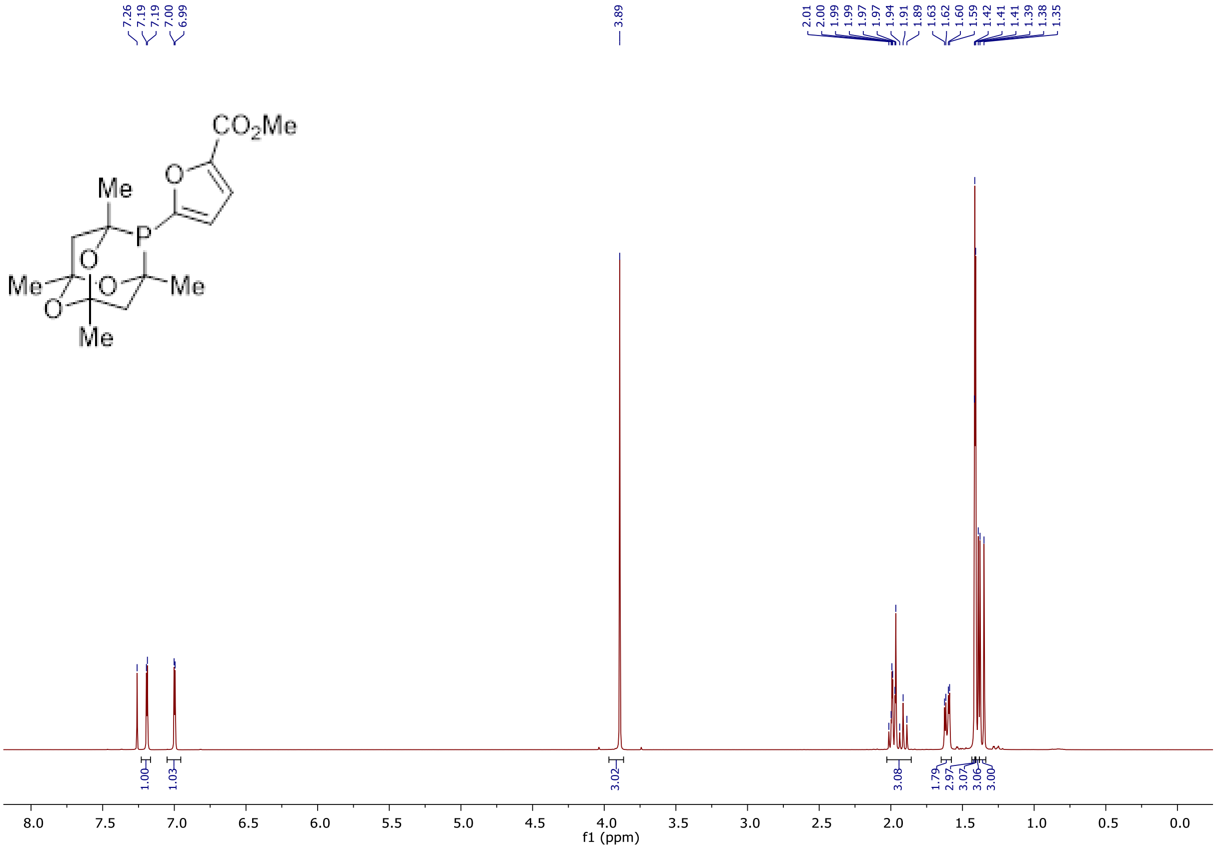

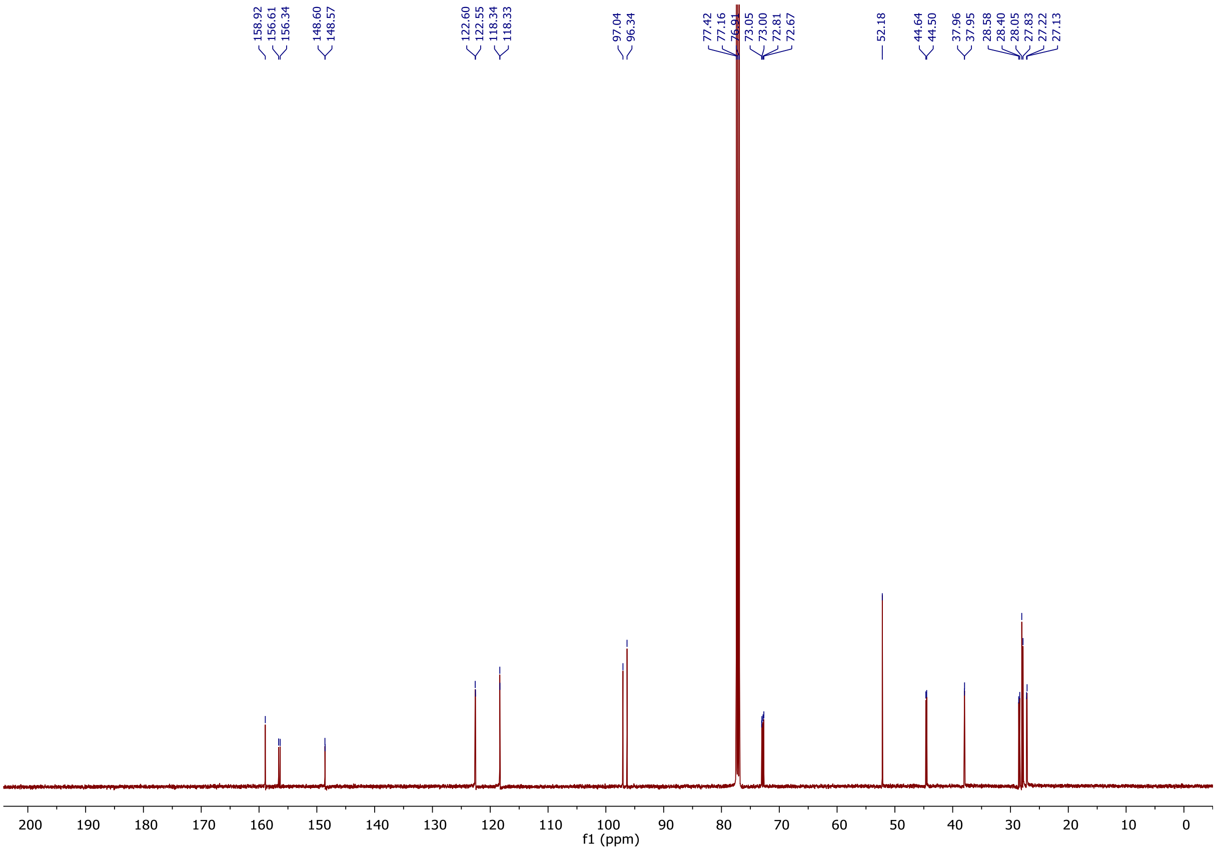

8-(Benzofuran-2-yl)-1,3,5,7-tetramethyl-2,4,6-trioxa-8-phosphaadamantane

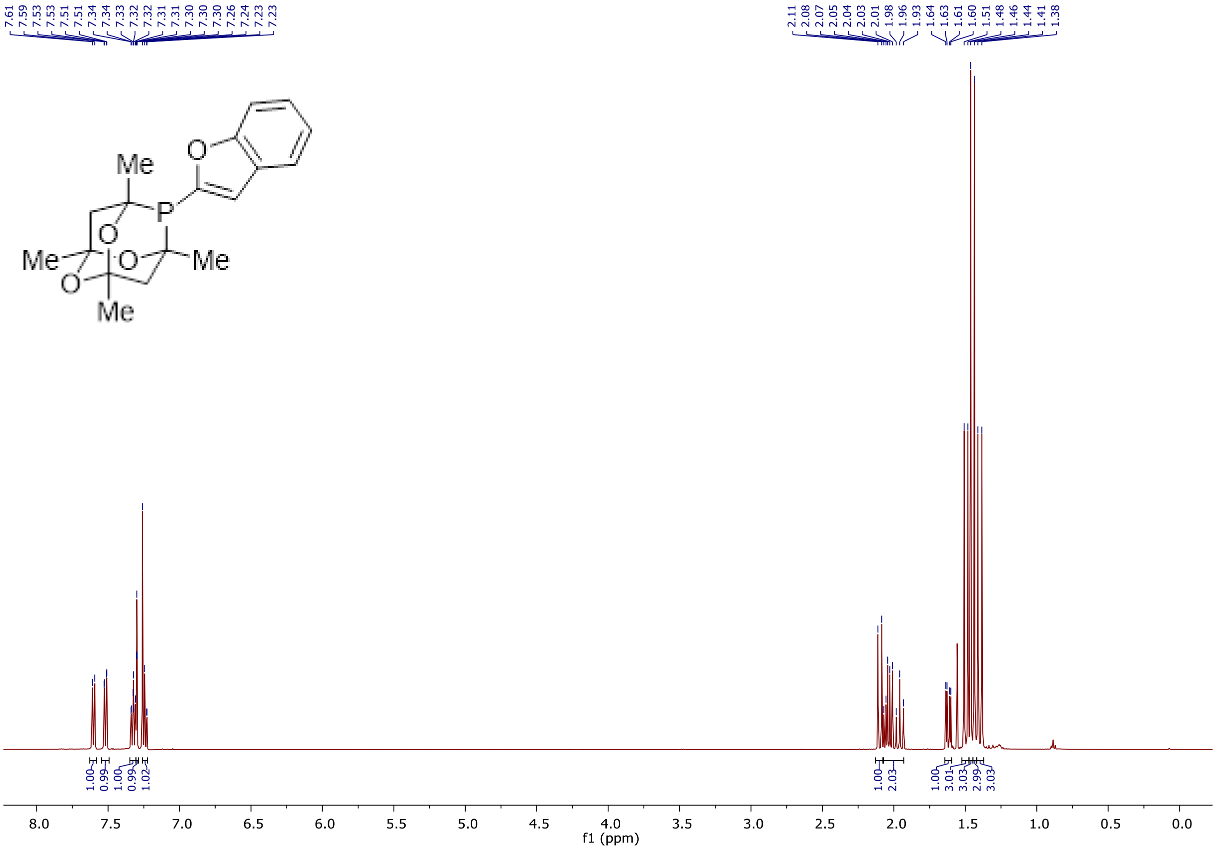

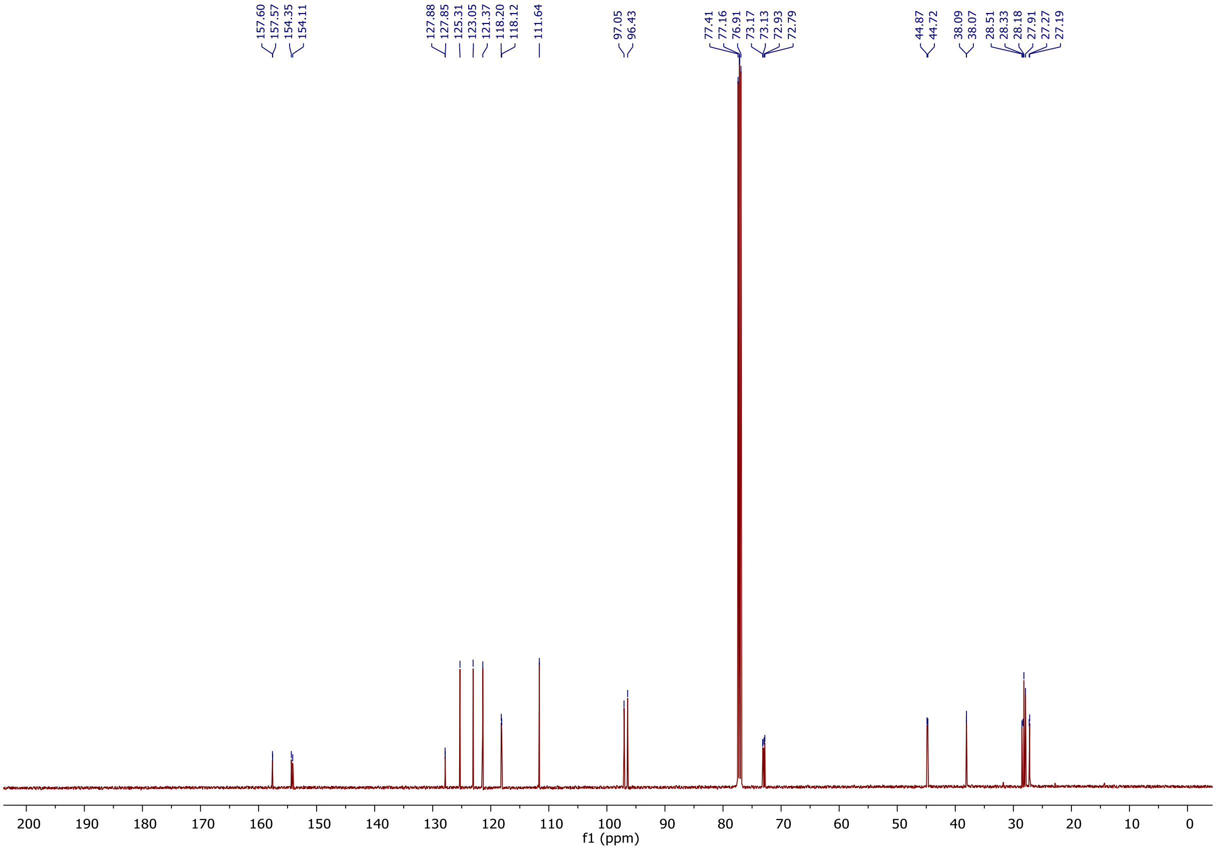

8-(Benzo[*b*]thiophen-2-yl)-1,3,5,7-tetramethyl-2,4,6-trioxa-8-phosphaadamantane

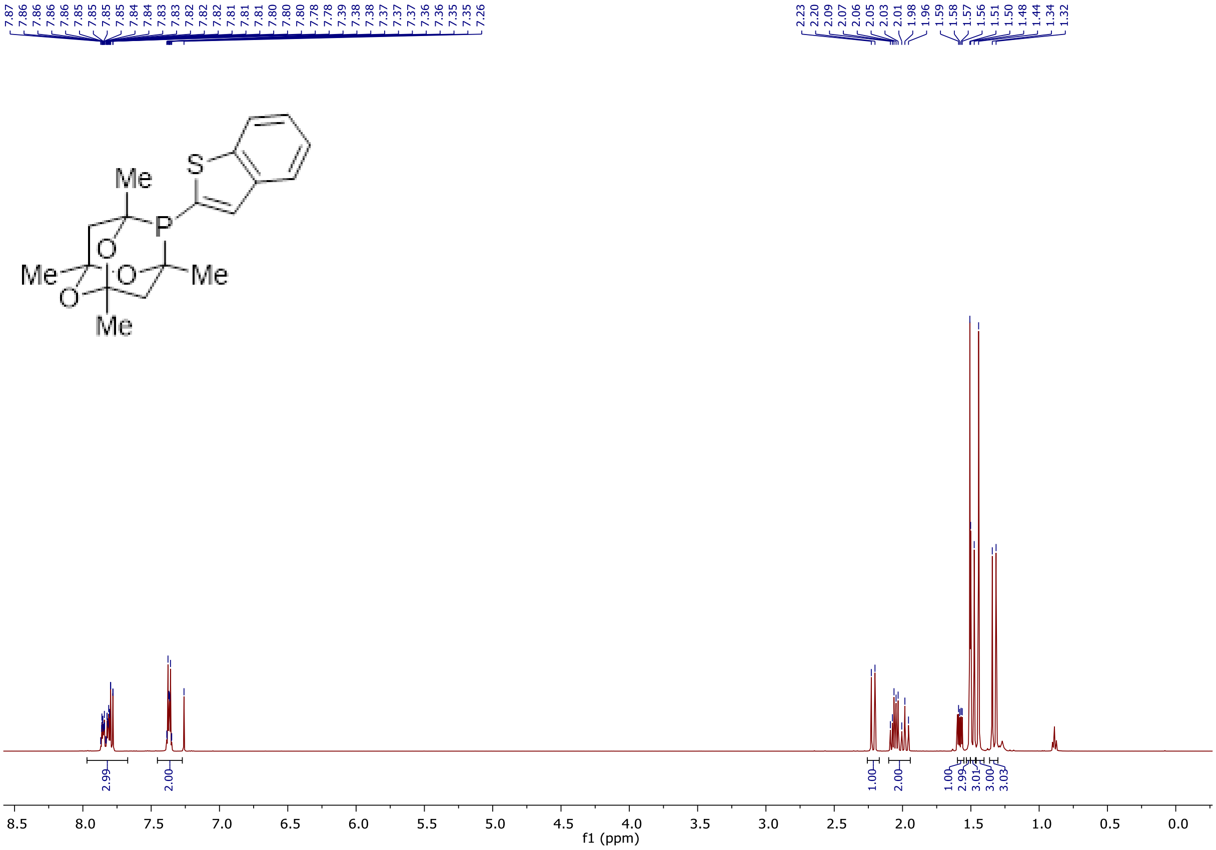

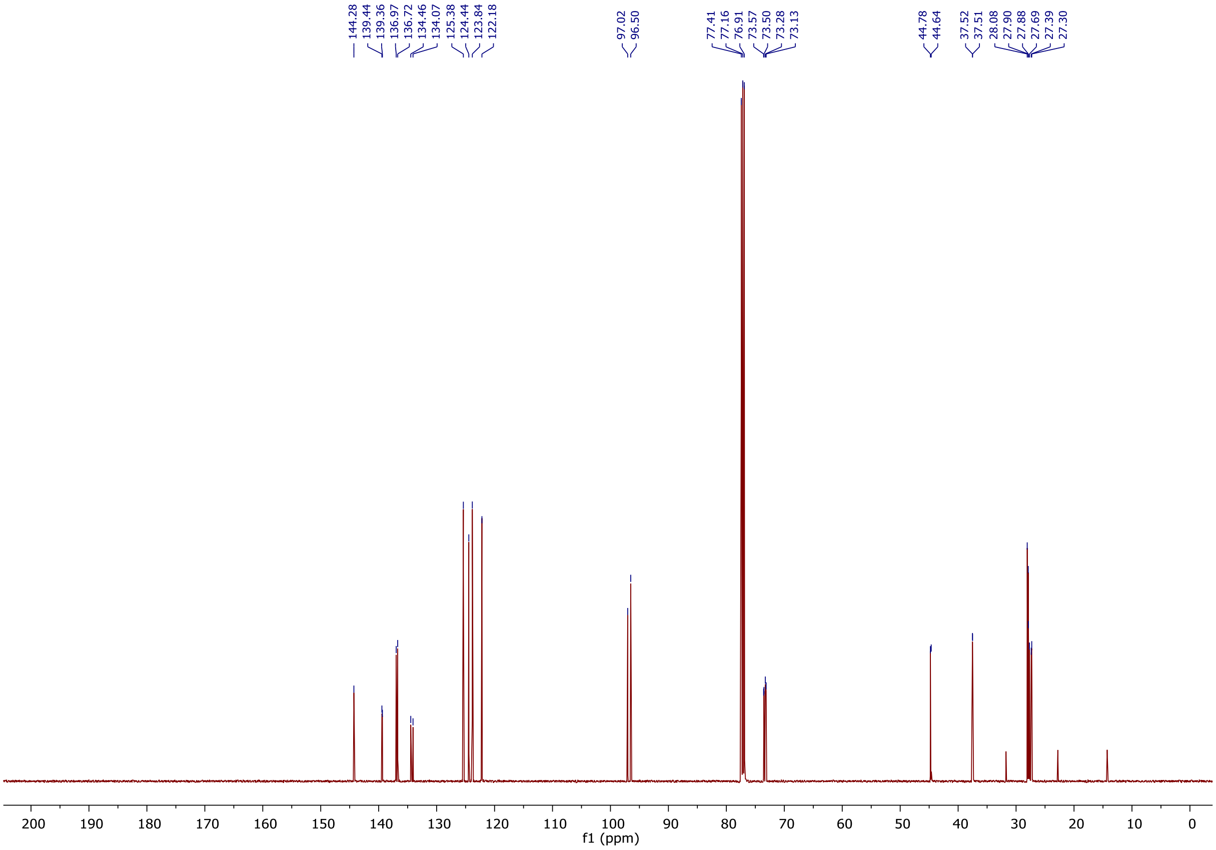

Benzyl (4-methylenehexyl)(tosyloxy)carbamate (1a’)

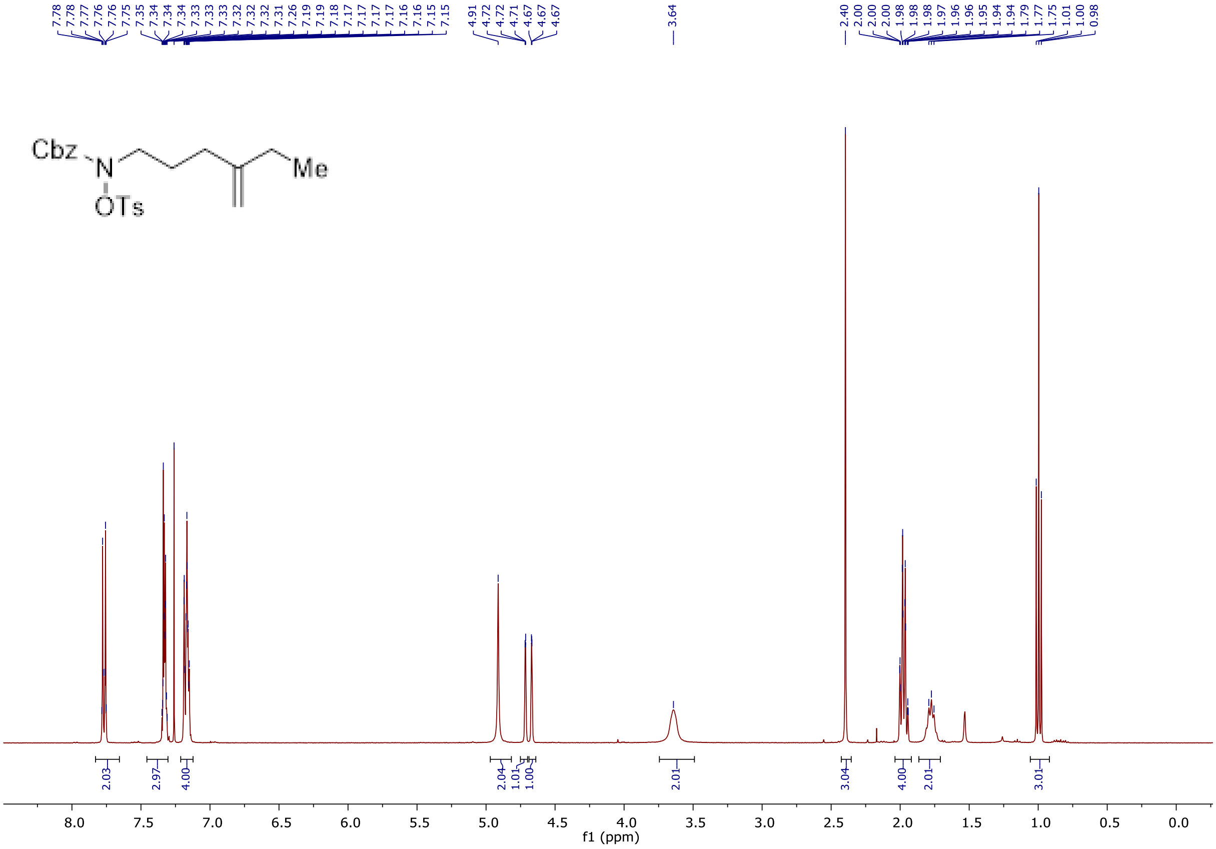

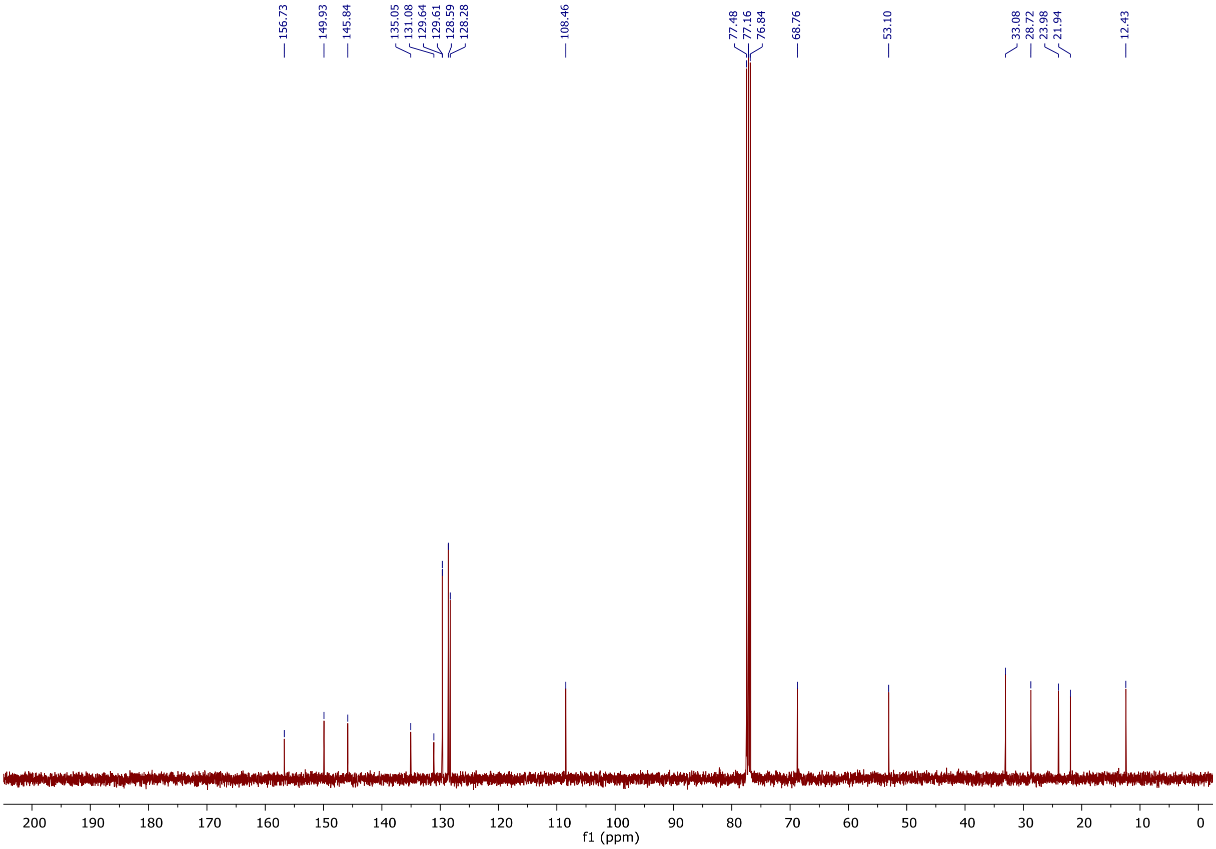

Benzyl (4-methylenehexyl)(pivaloyloxy)carbamate (1a'')

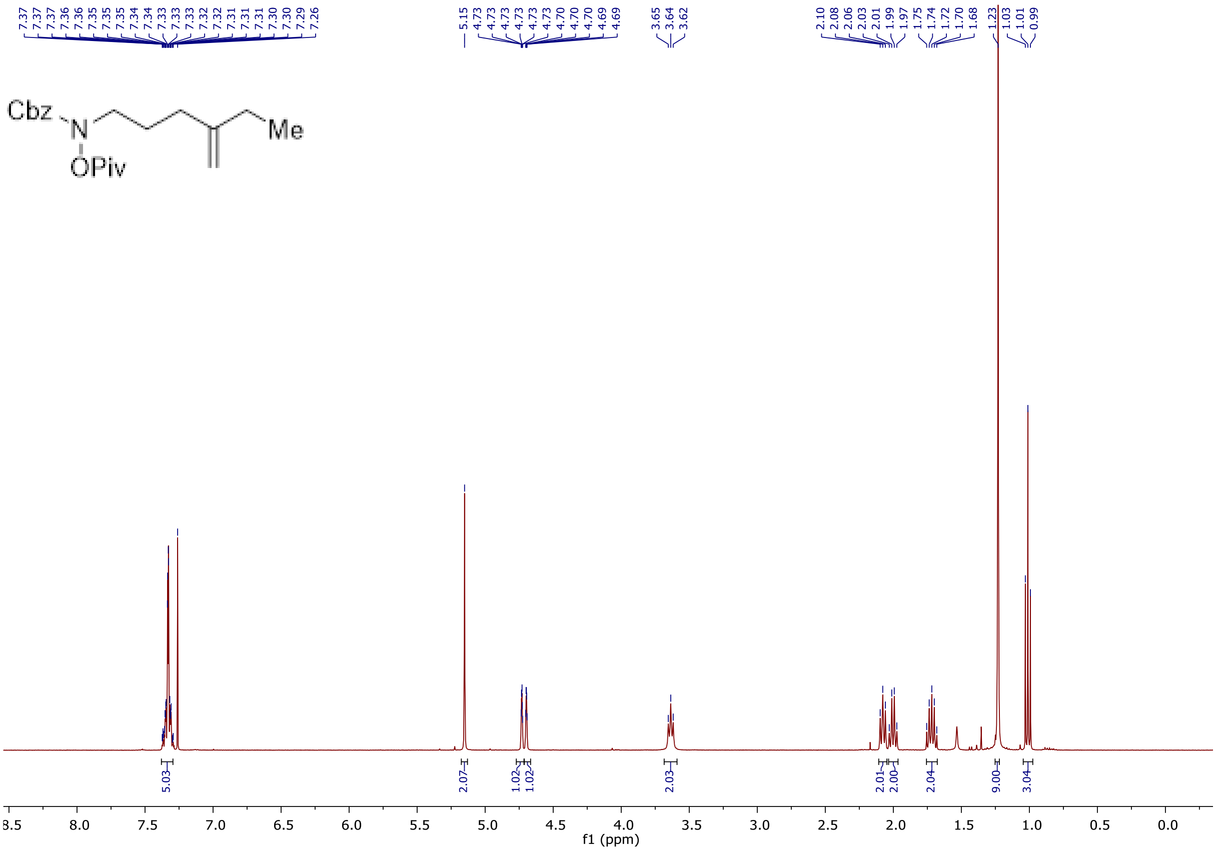

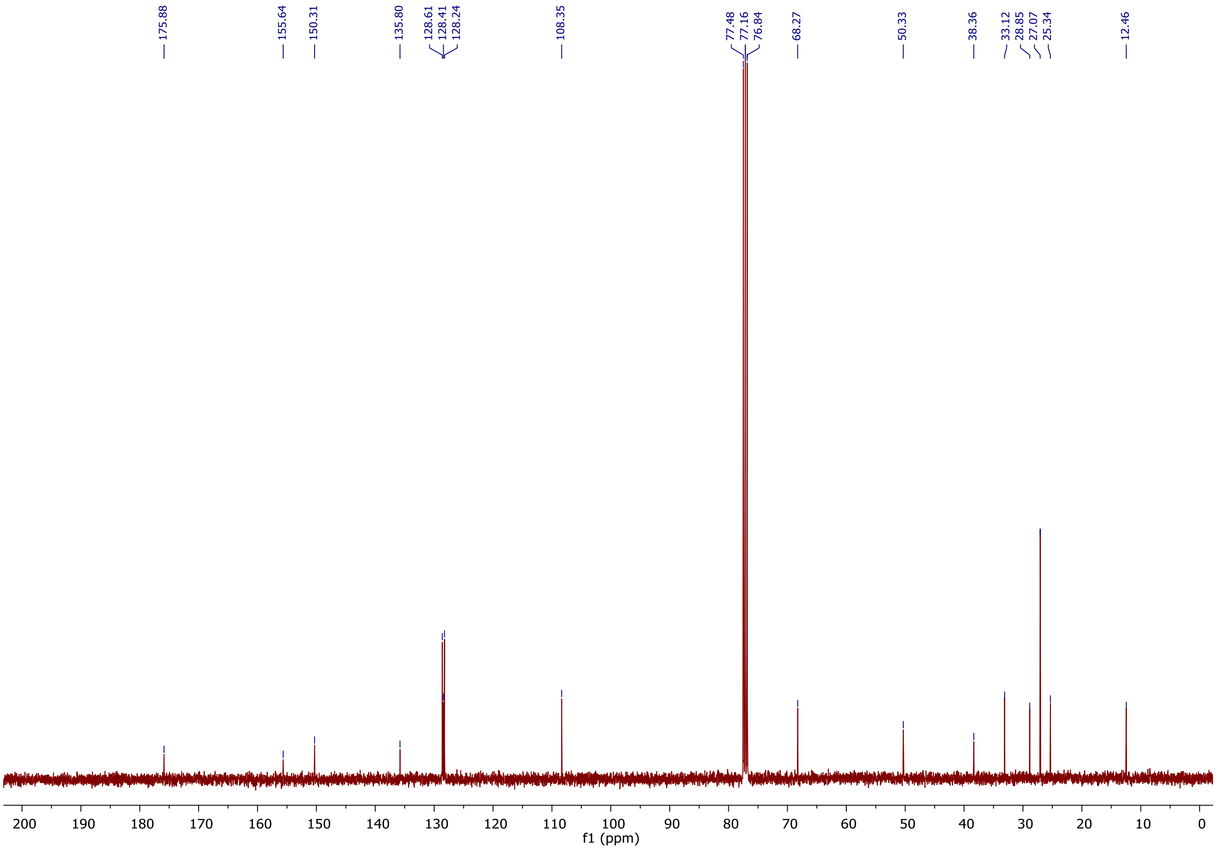

Benzyl (4-methylenehexyl)((perfluorobenzoyl)oxy)carbamate (1a)

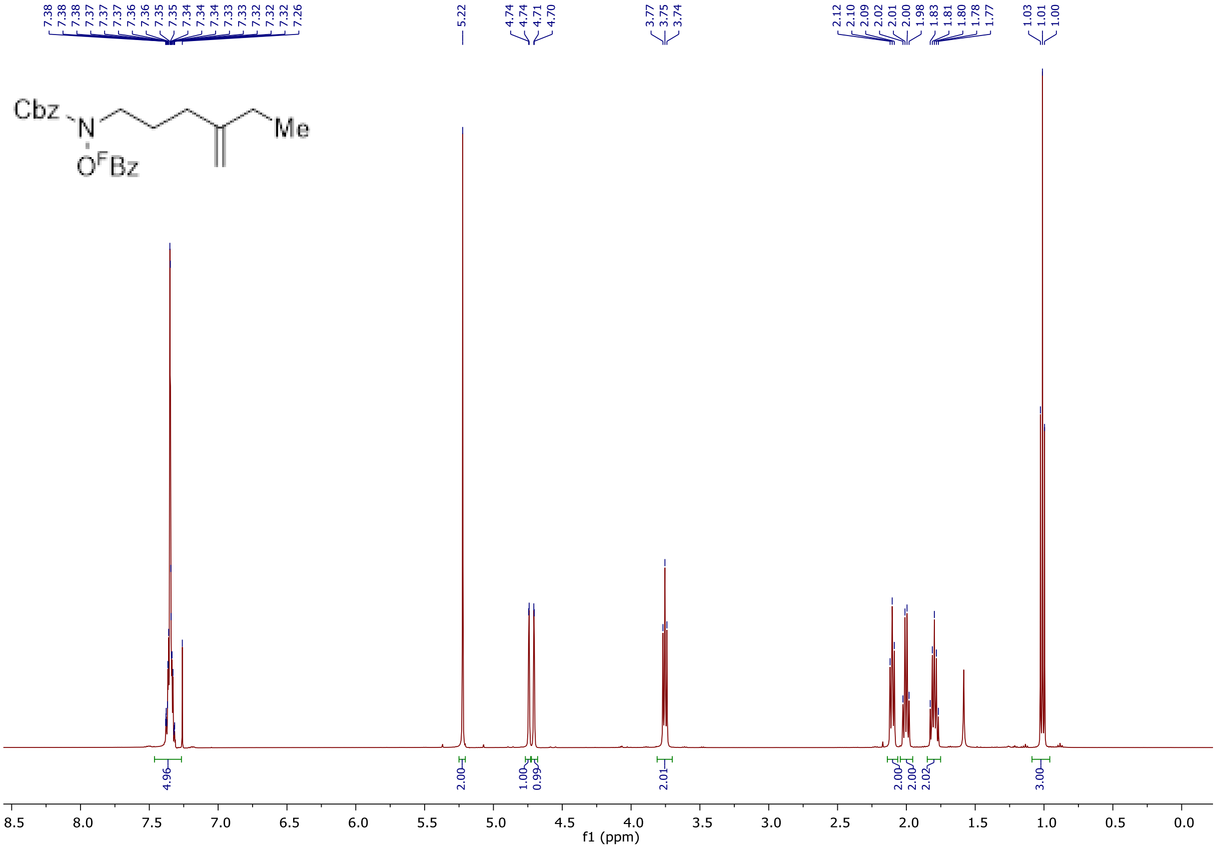

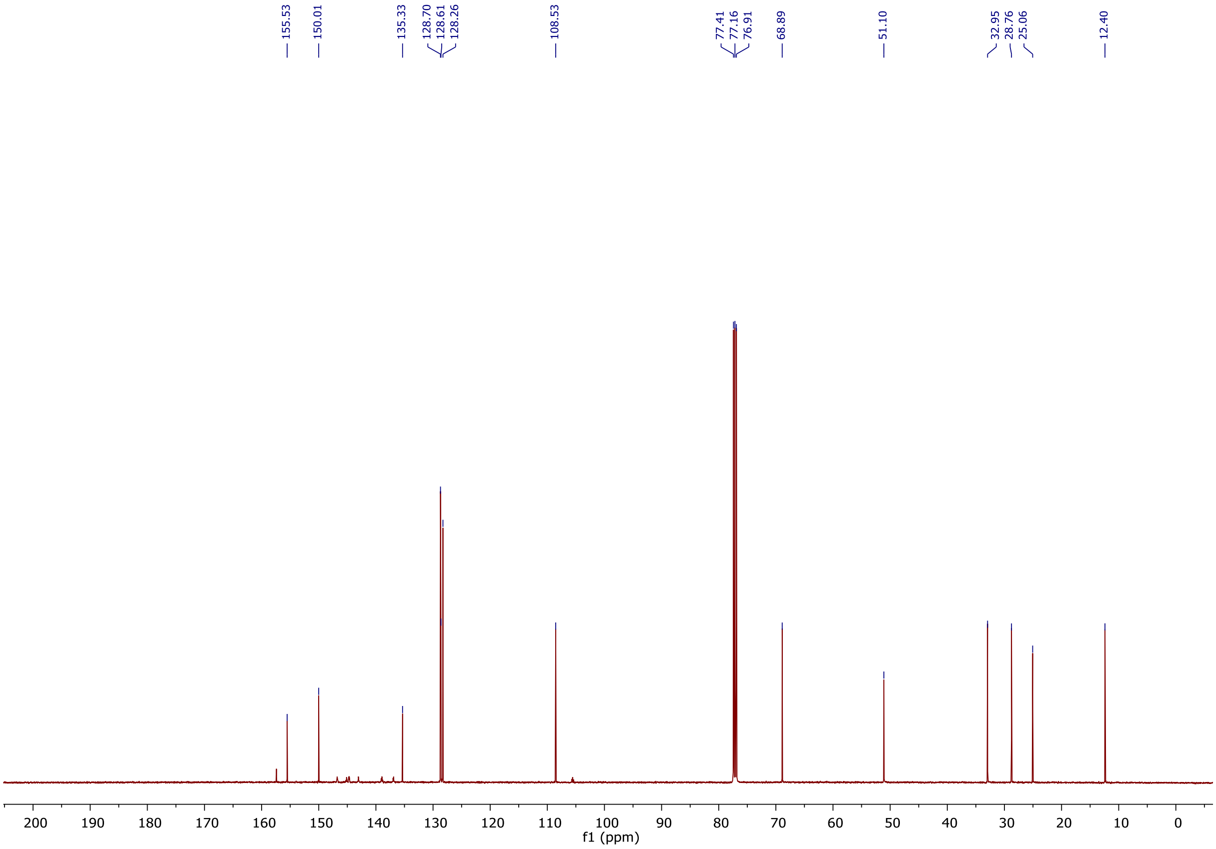

Benzyl 1-ethyl-2-azabicyclo[3.1.0]hexane-2-carboxylate (2a)

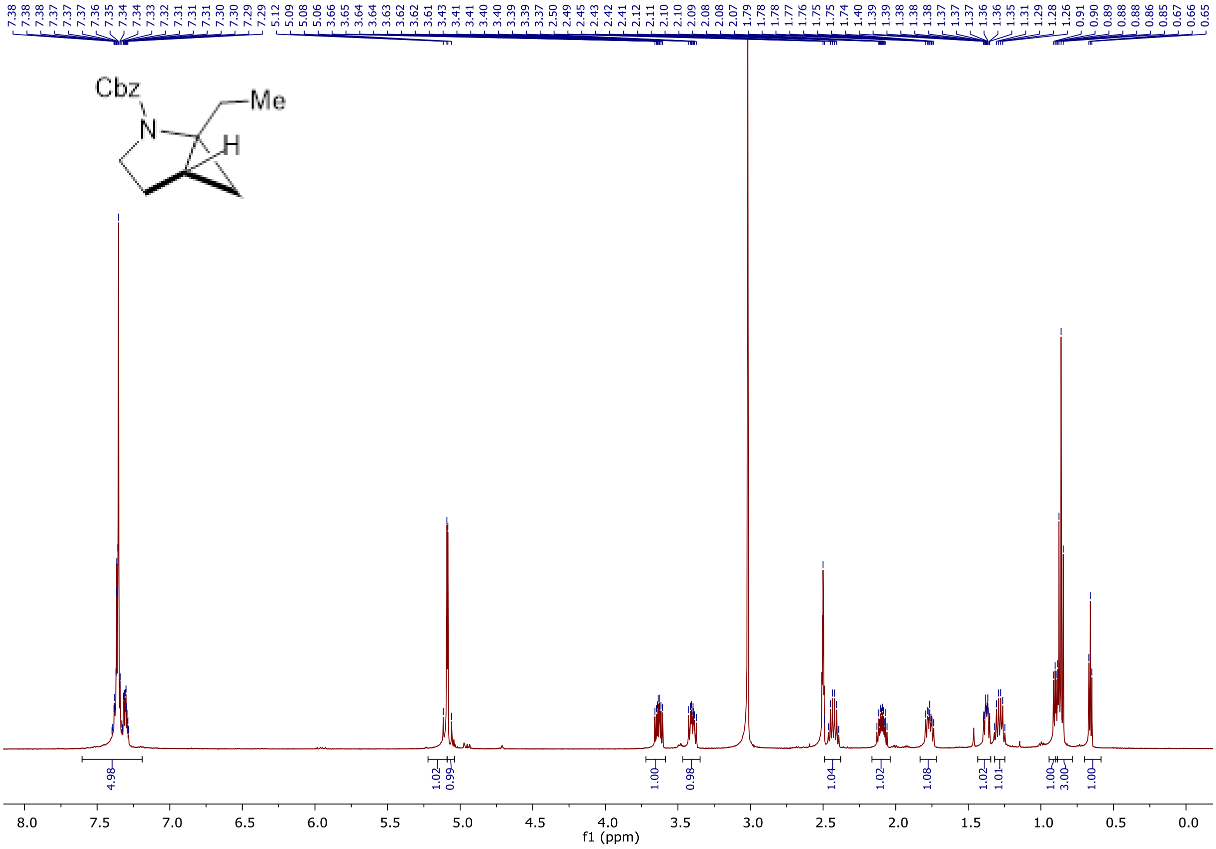

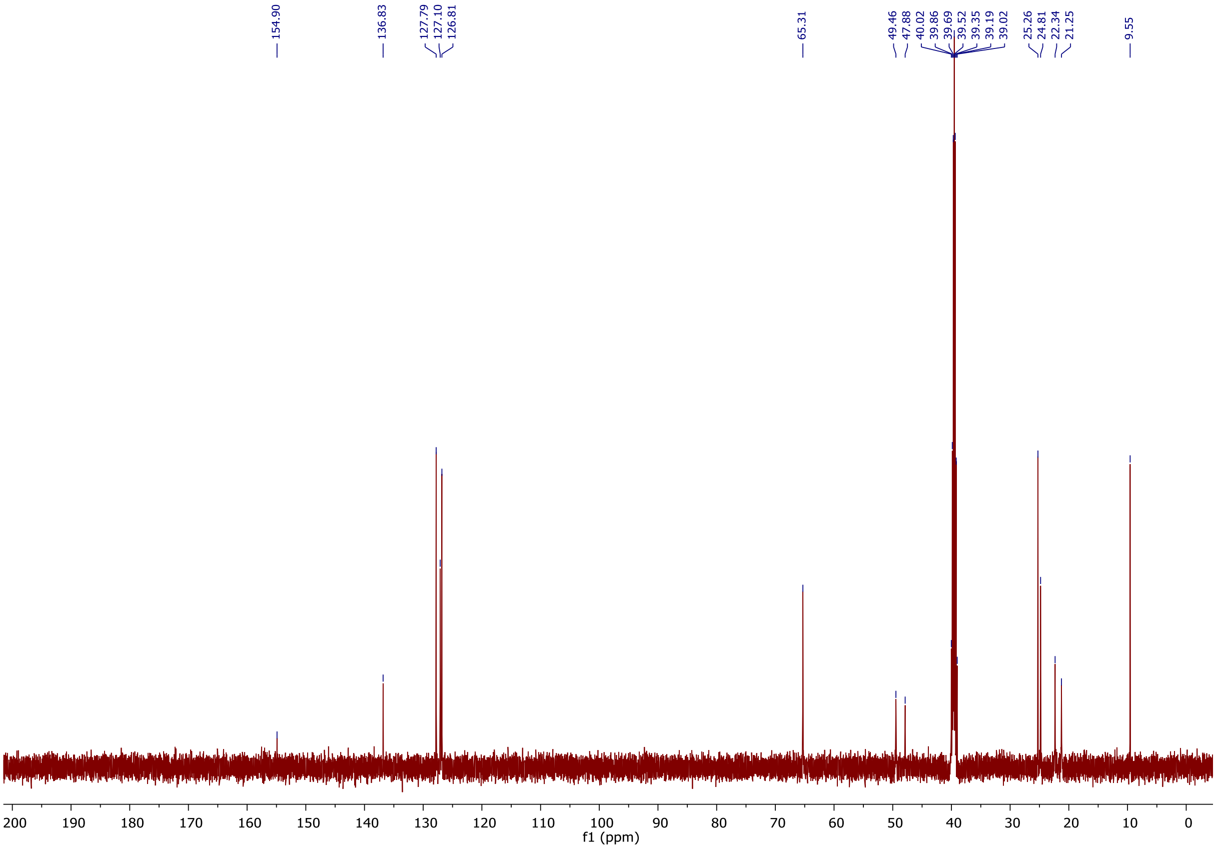

5-Methyl-4-methylenehexan-1-ol

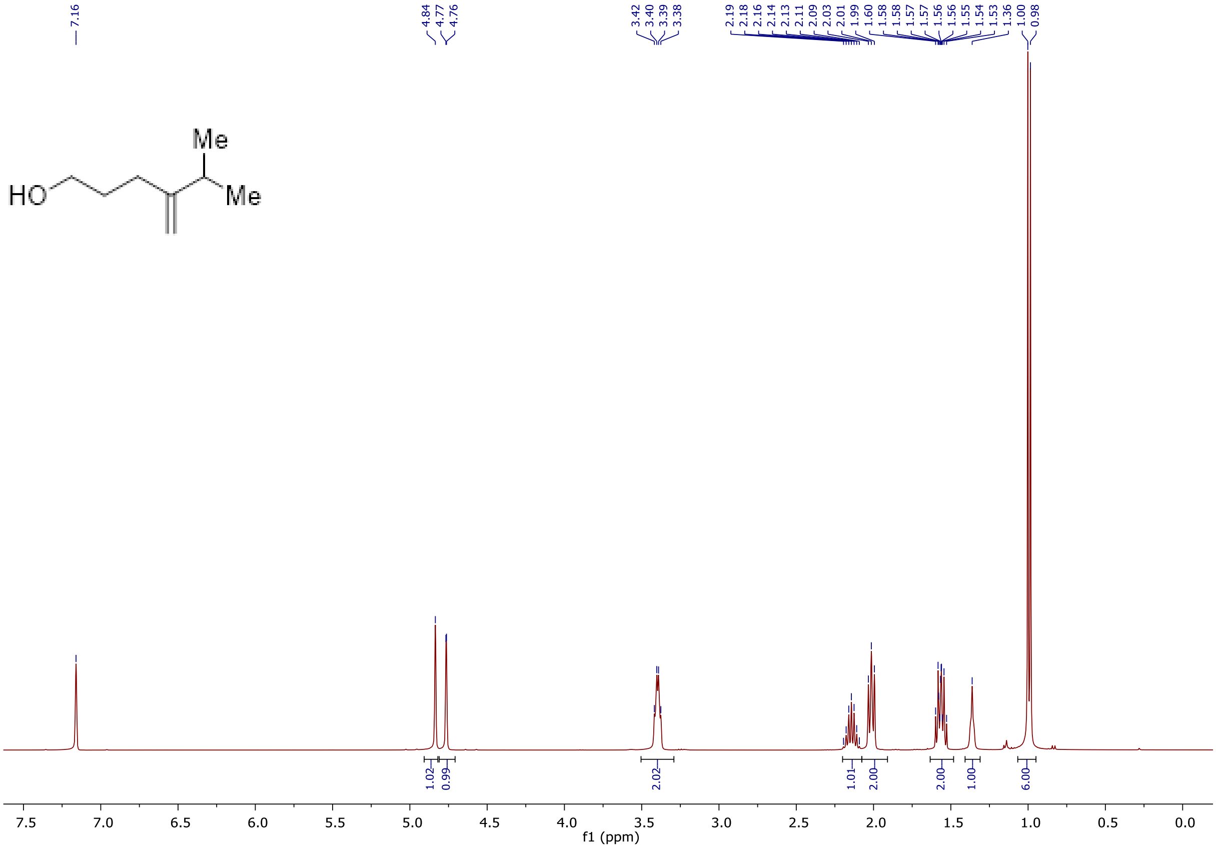

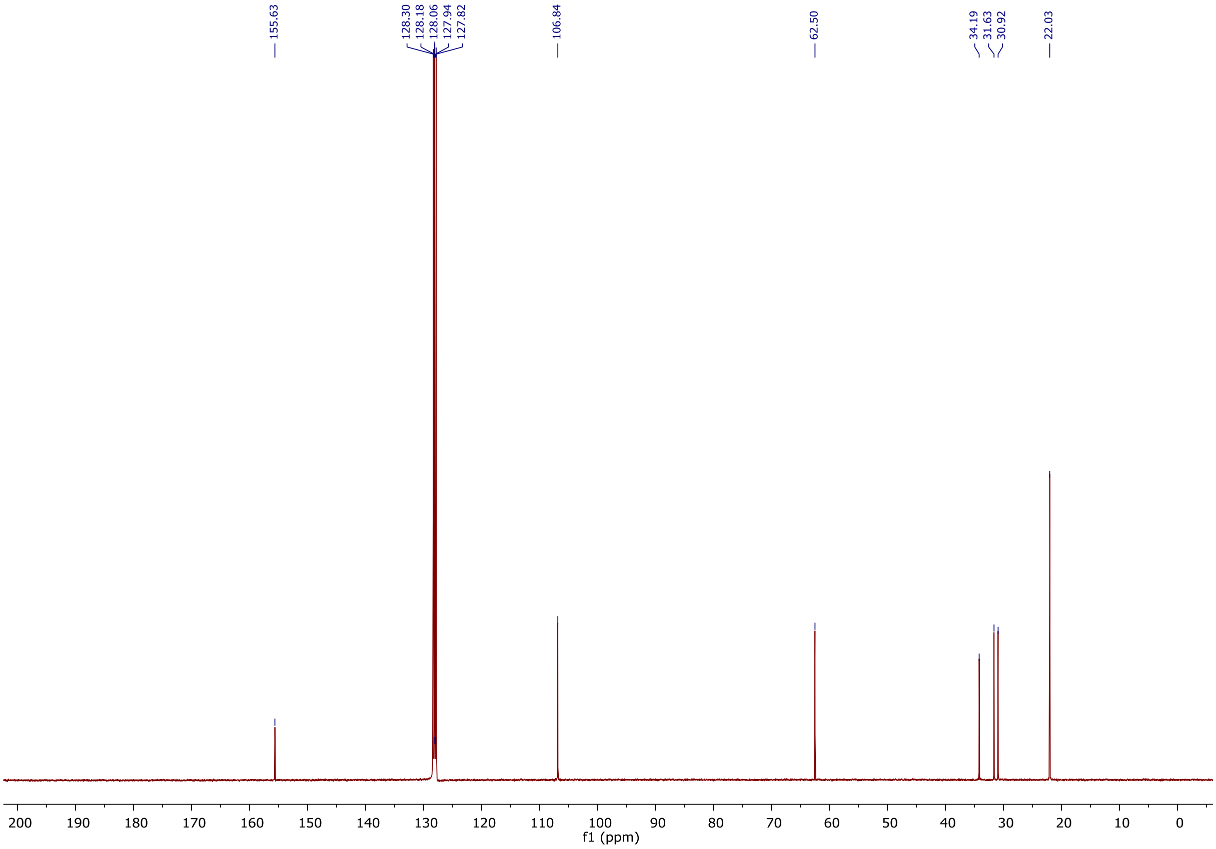

Benzyl (5-methyl-4-methylenehexyl)((perfluorobenzoyl)oxy)carbamate (1b)

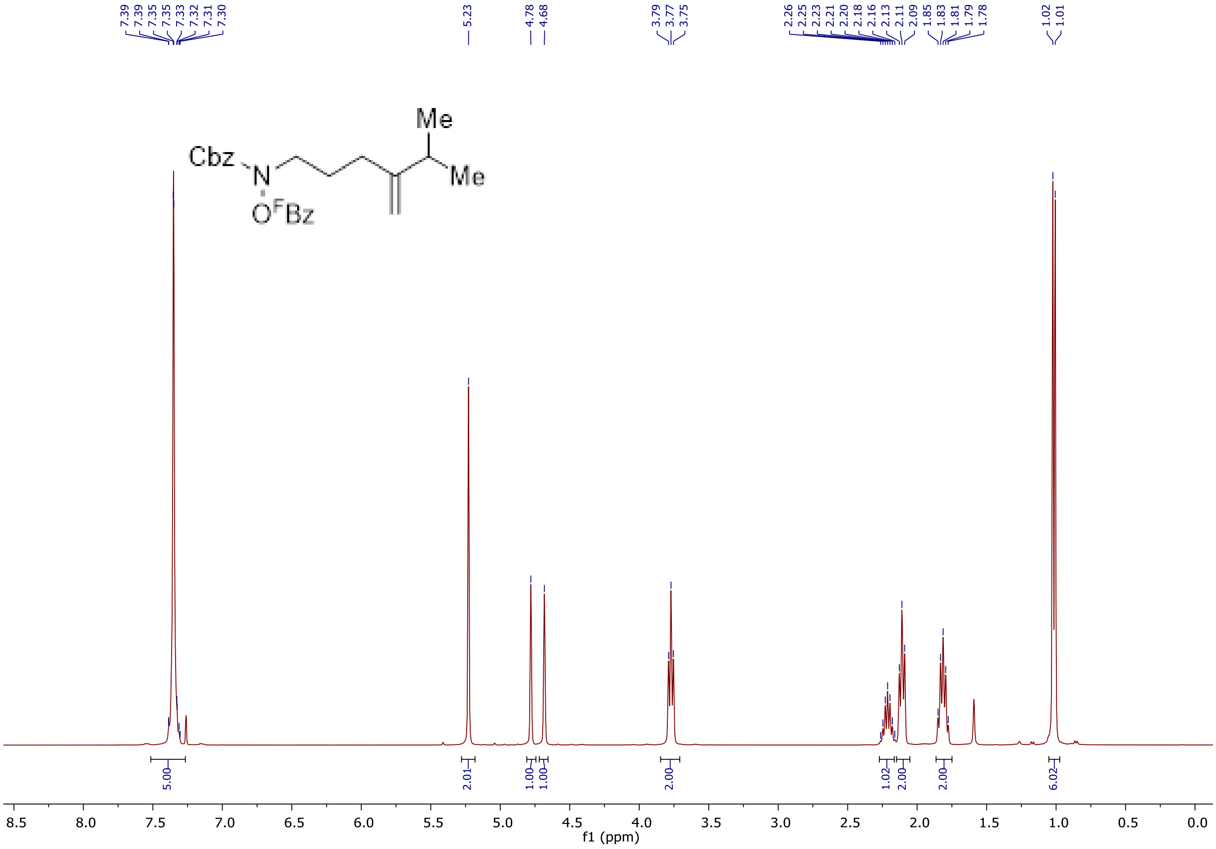

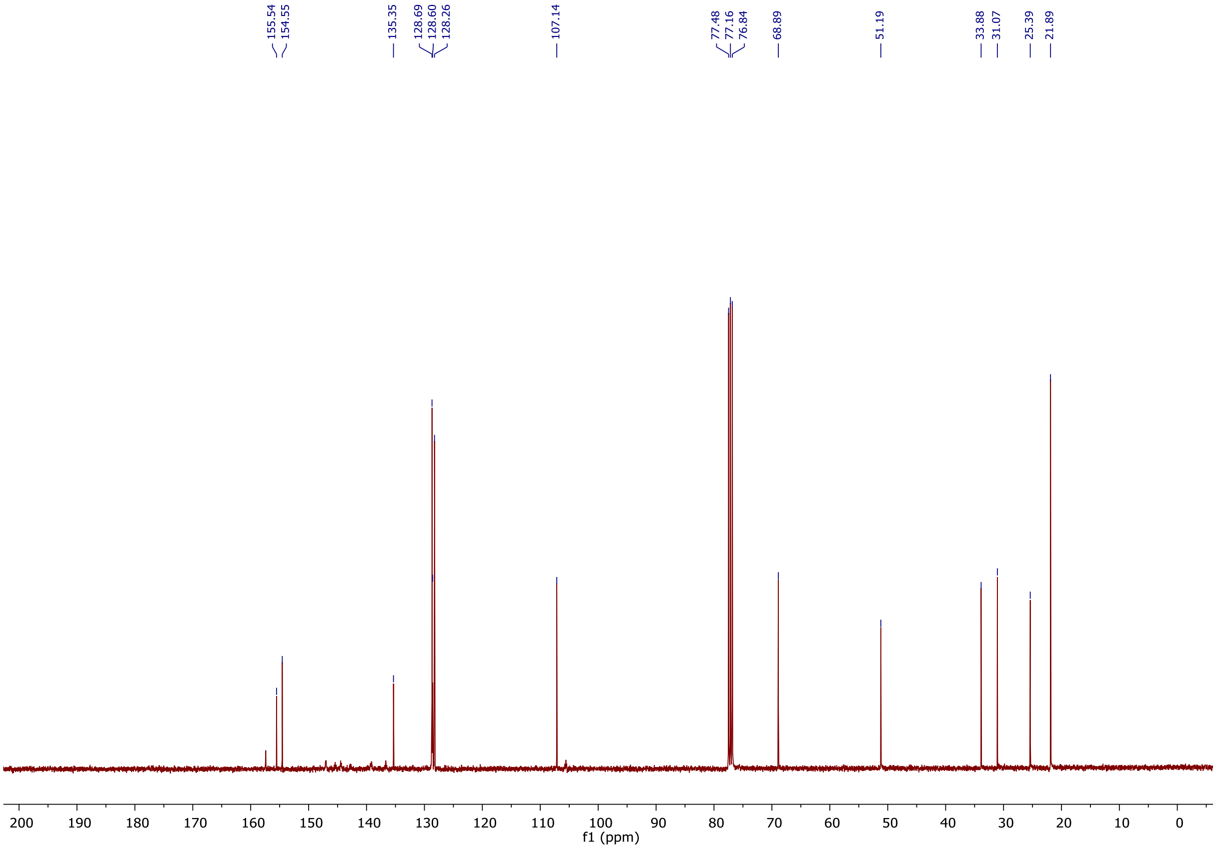

Benzyl 1-isopropyl-2-azabicyclo[3.1.0]hexane-2-carboxylate (2b)

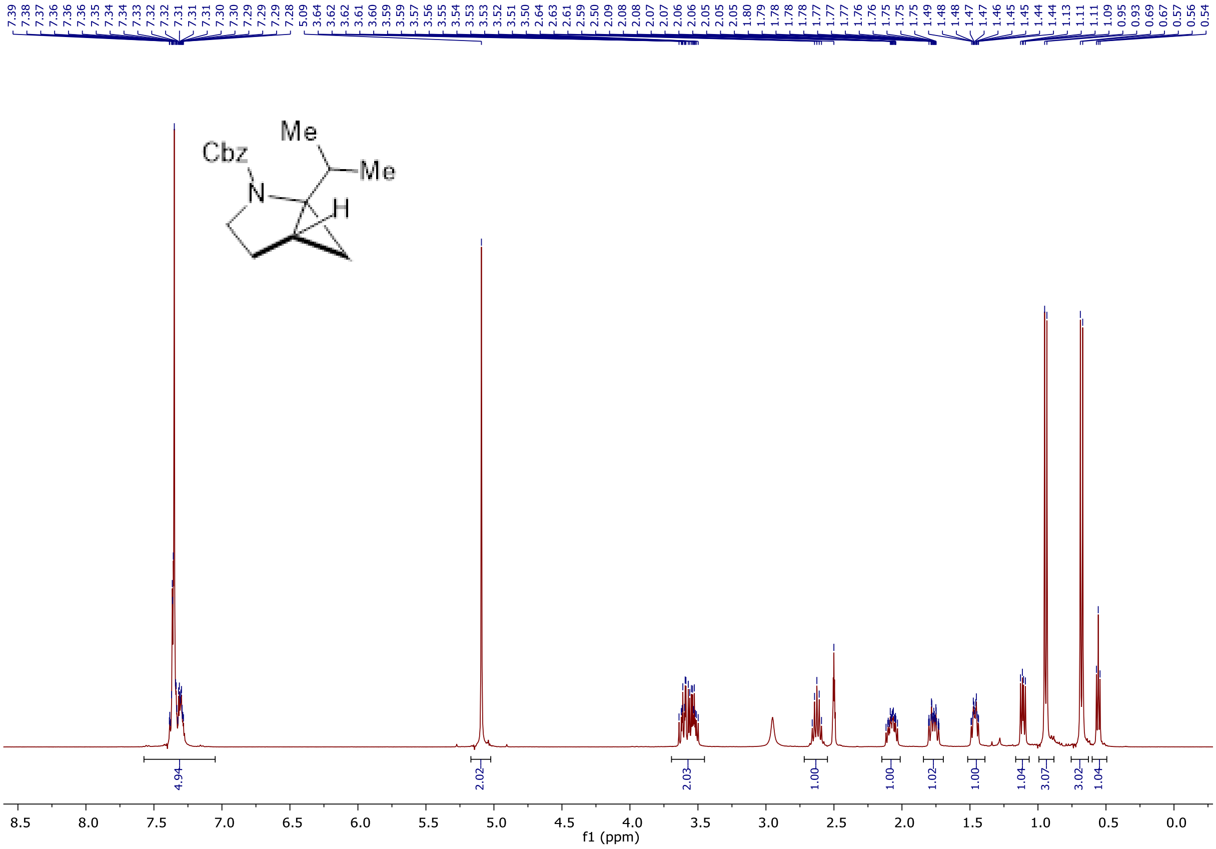

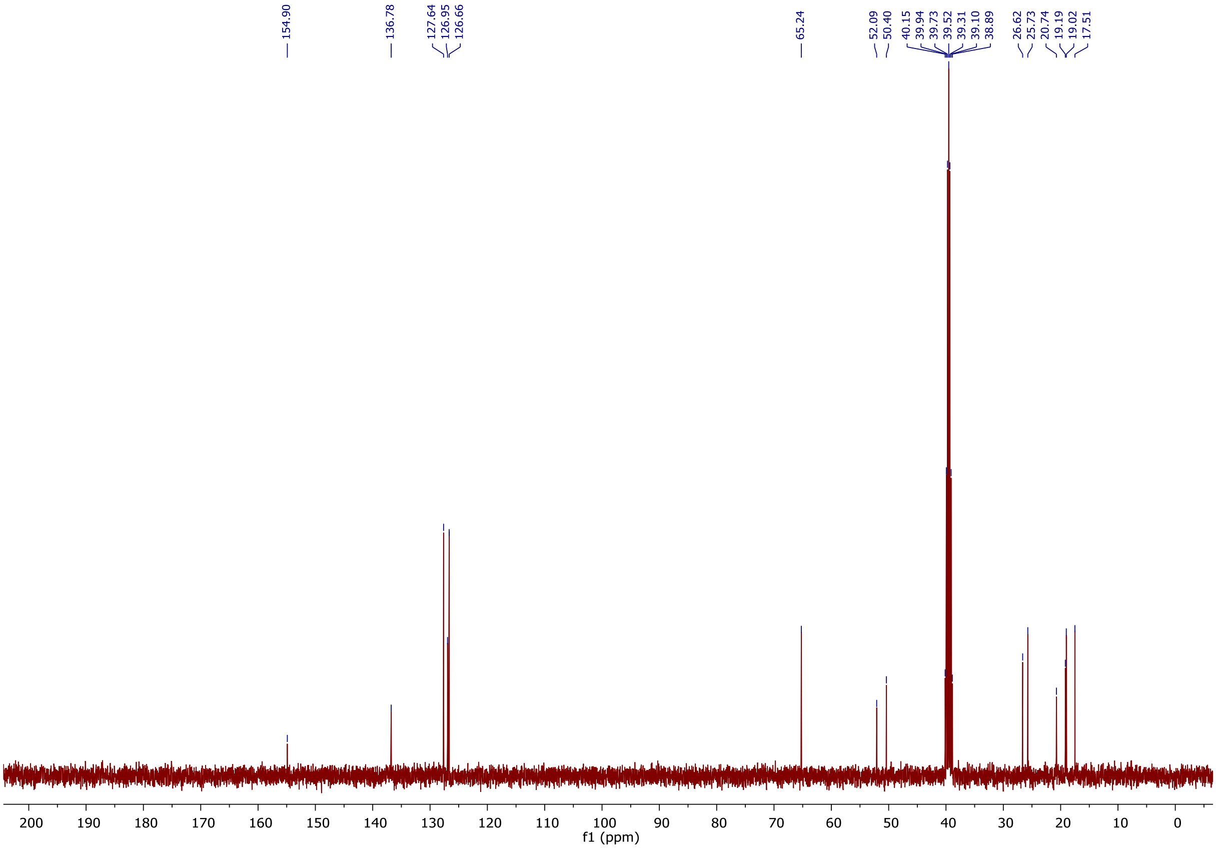

Benzyl (4-methylenedecyl)((perfluorobenzoyl)oxy)carbamate (1c)

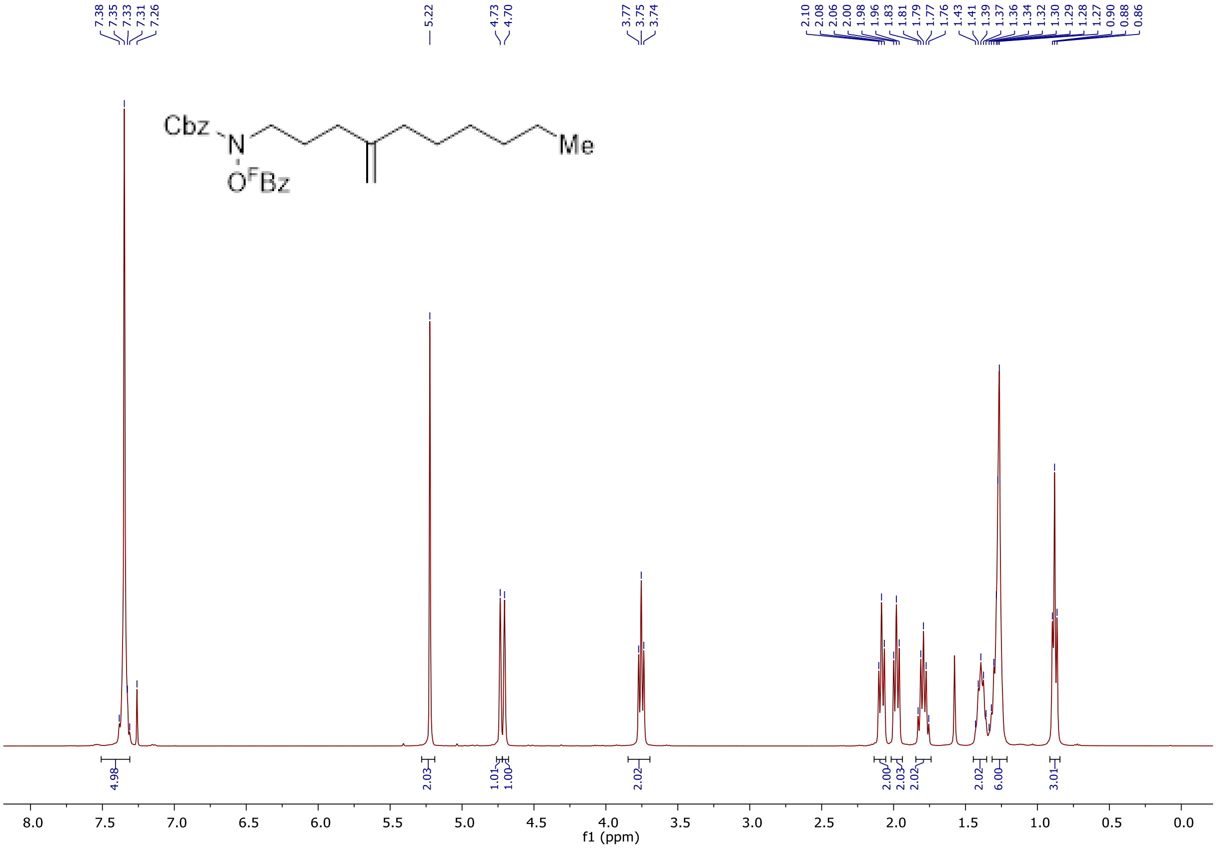

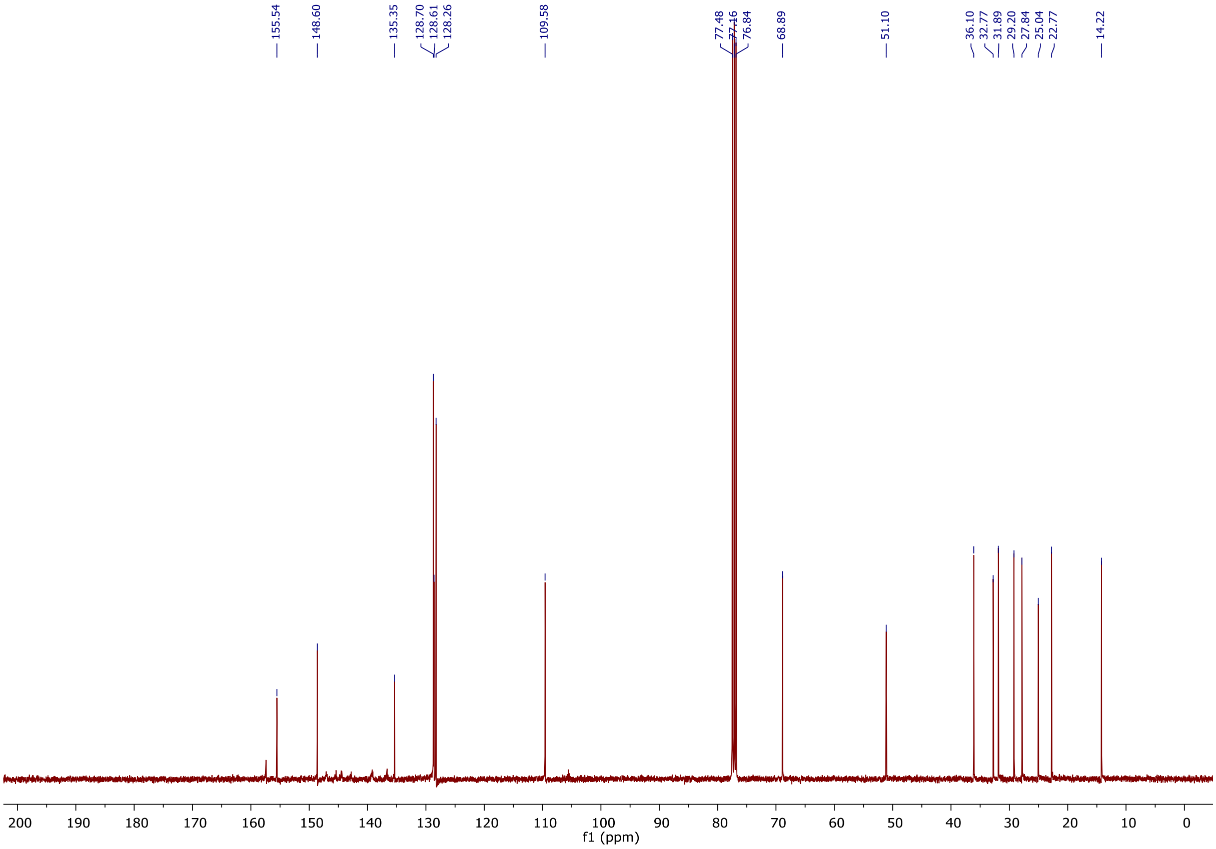

Benzyl 1-hexyl-2-azabicyclo[3.1.0]hexane-2-carboxylate (2c)

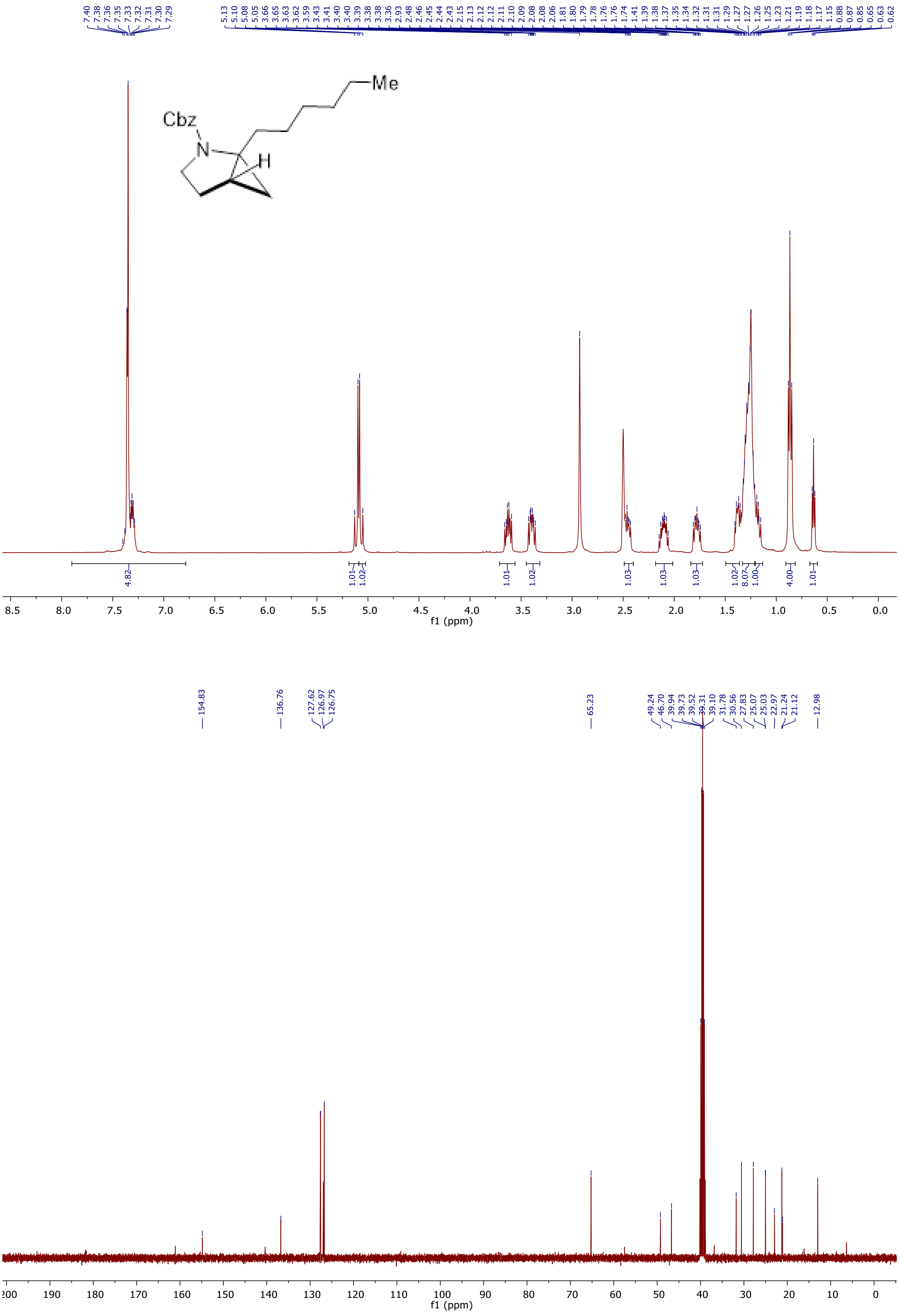

4-Ethoxy-2-methylenebutanal

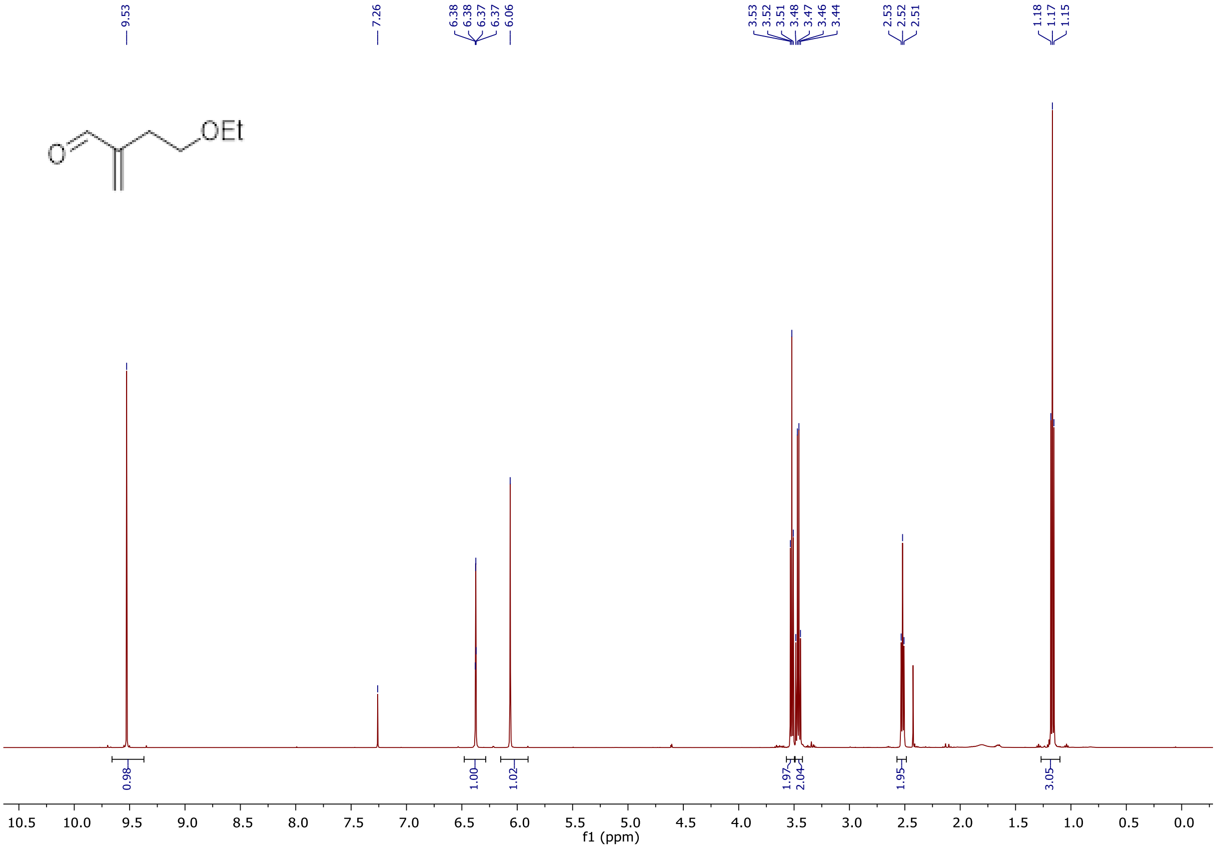

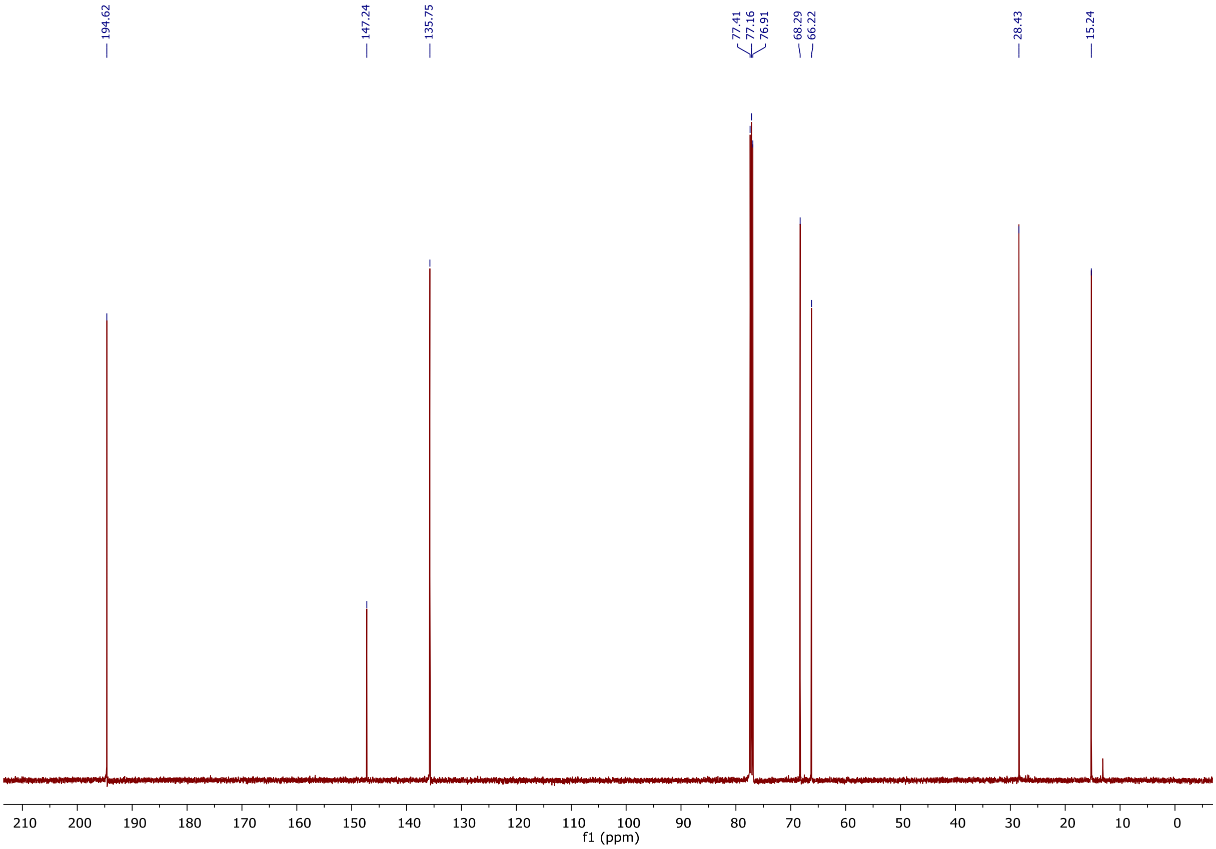

4-Ethoxy-2-methylenebutan-1-ol

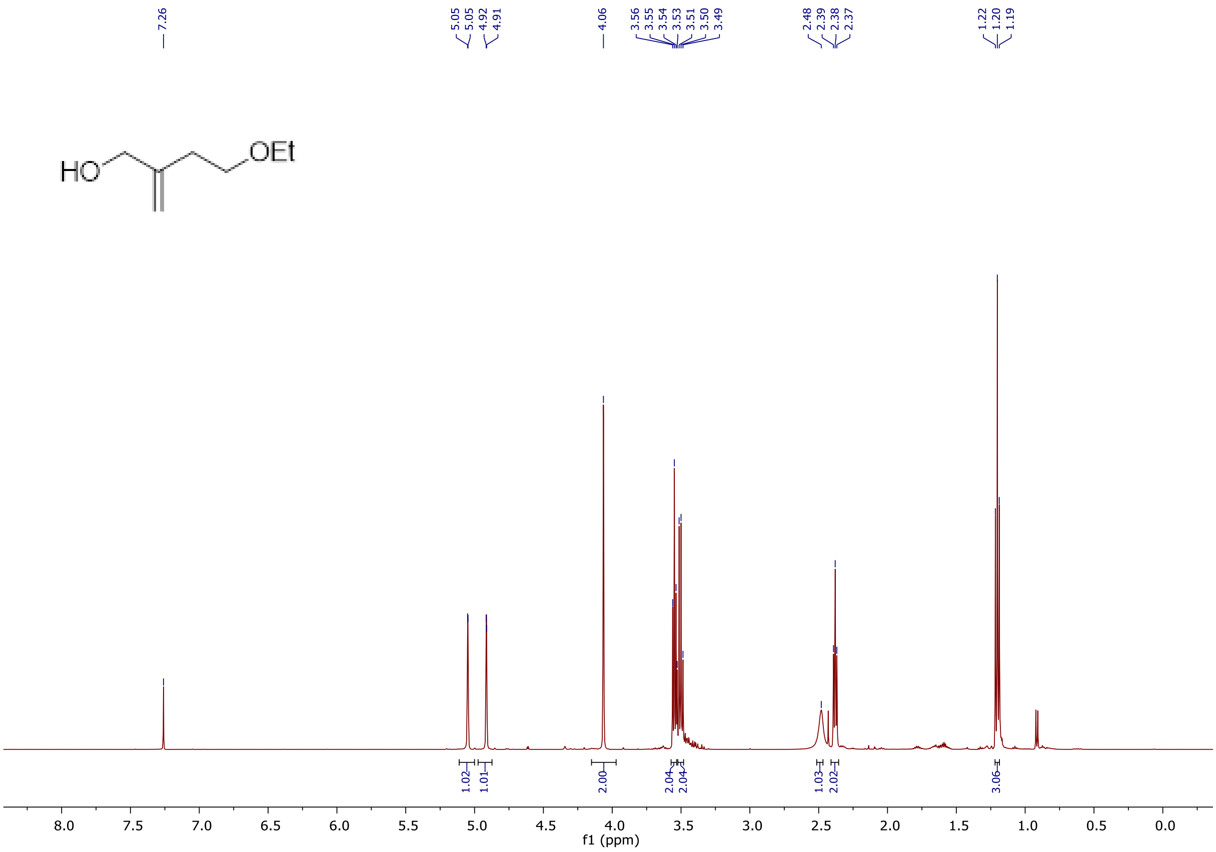

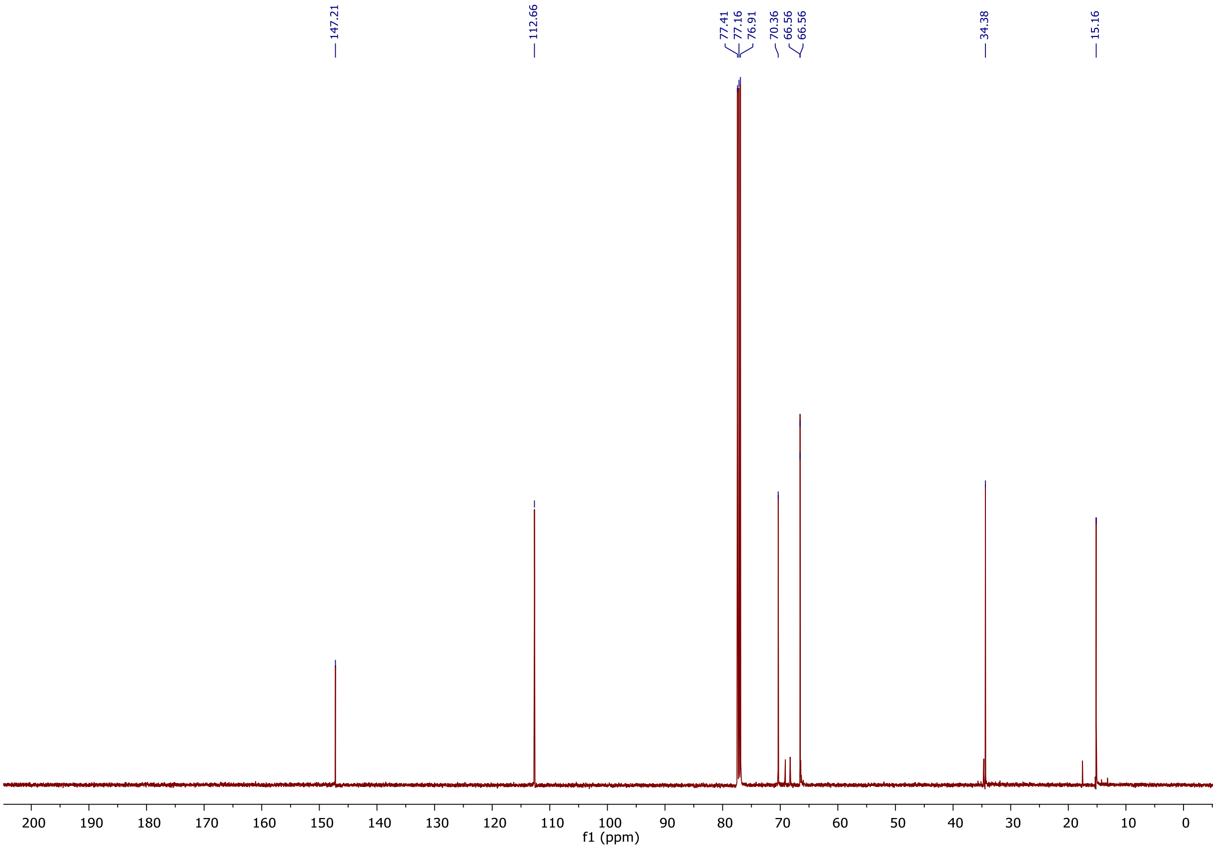

6-Ethoxy-4-methylenehexanoic acid

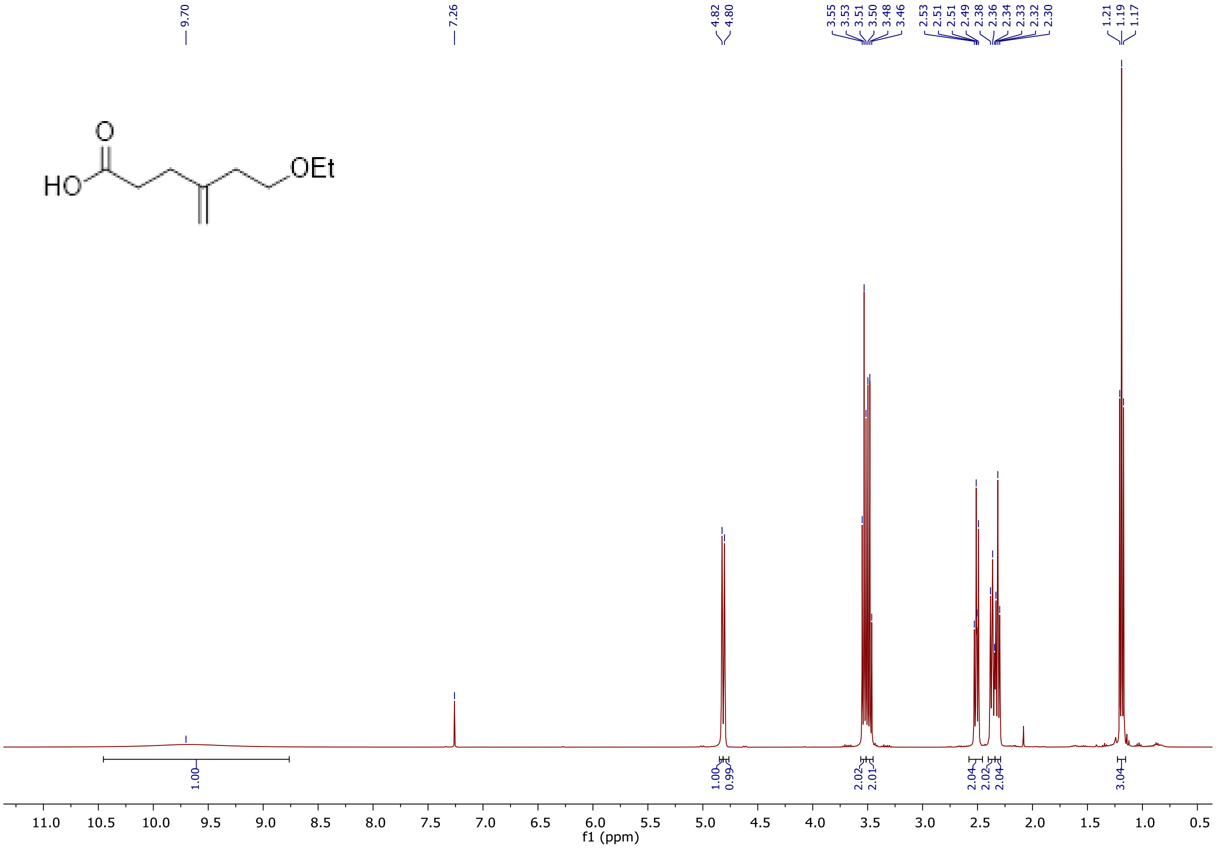

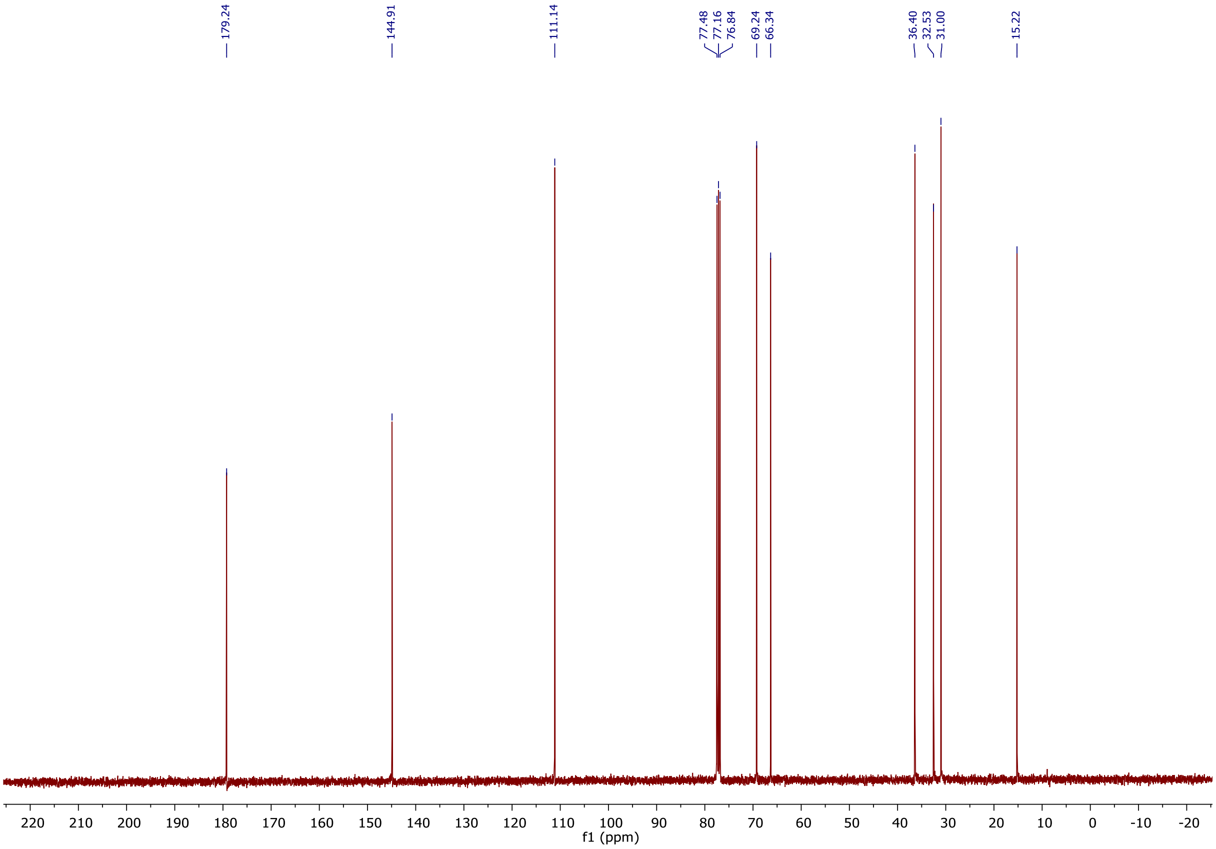

6-Ethoxy-4-methylenehexan-1-ol

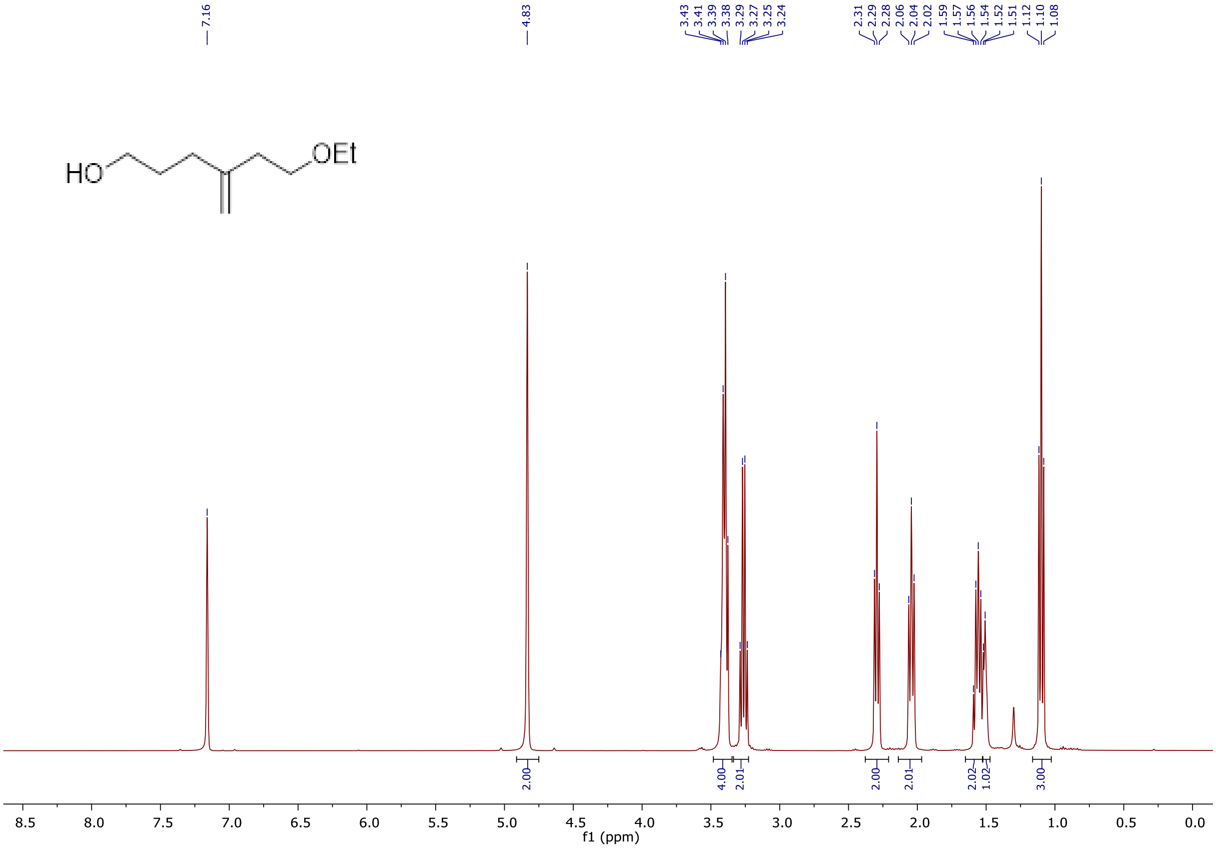

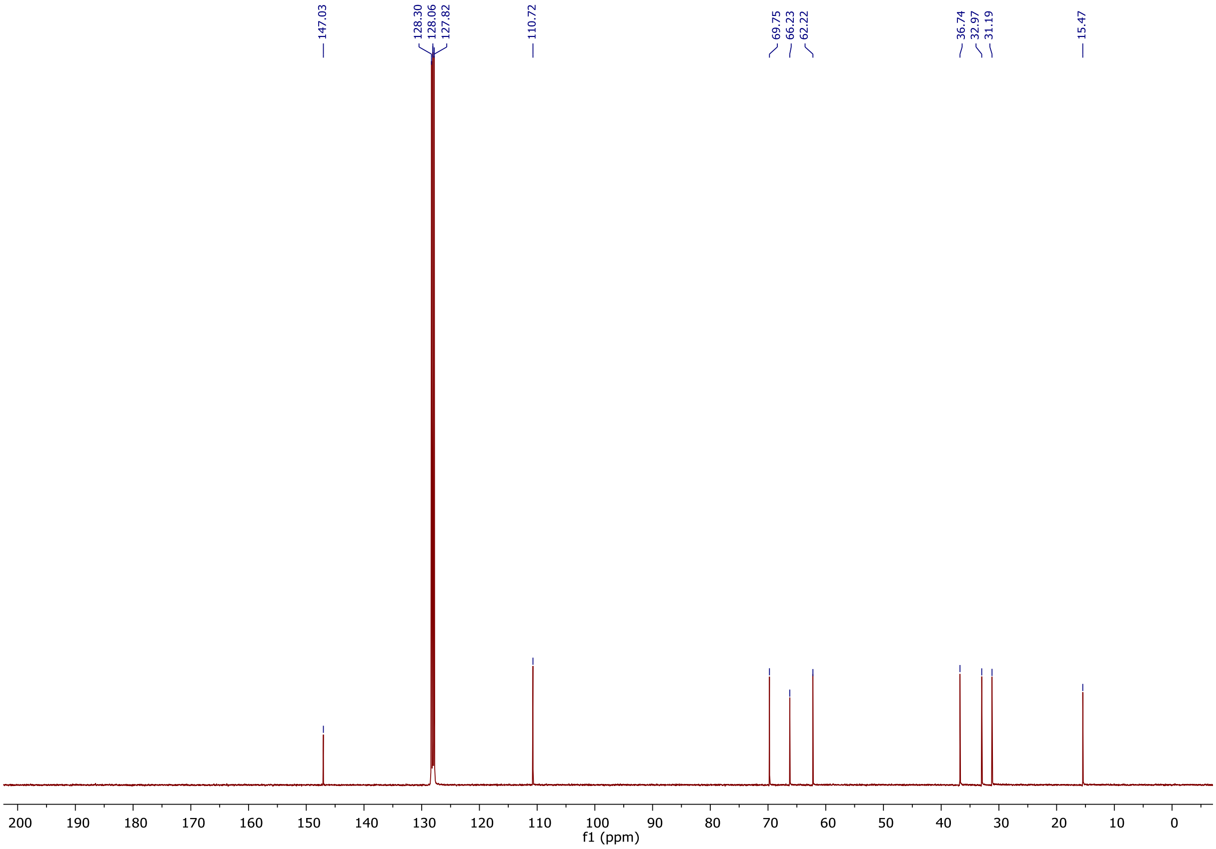

Benzyl (6-ethoxy-4-methylenehexyl)((perfluorobenzoyl)oxy)carbamate (1d)

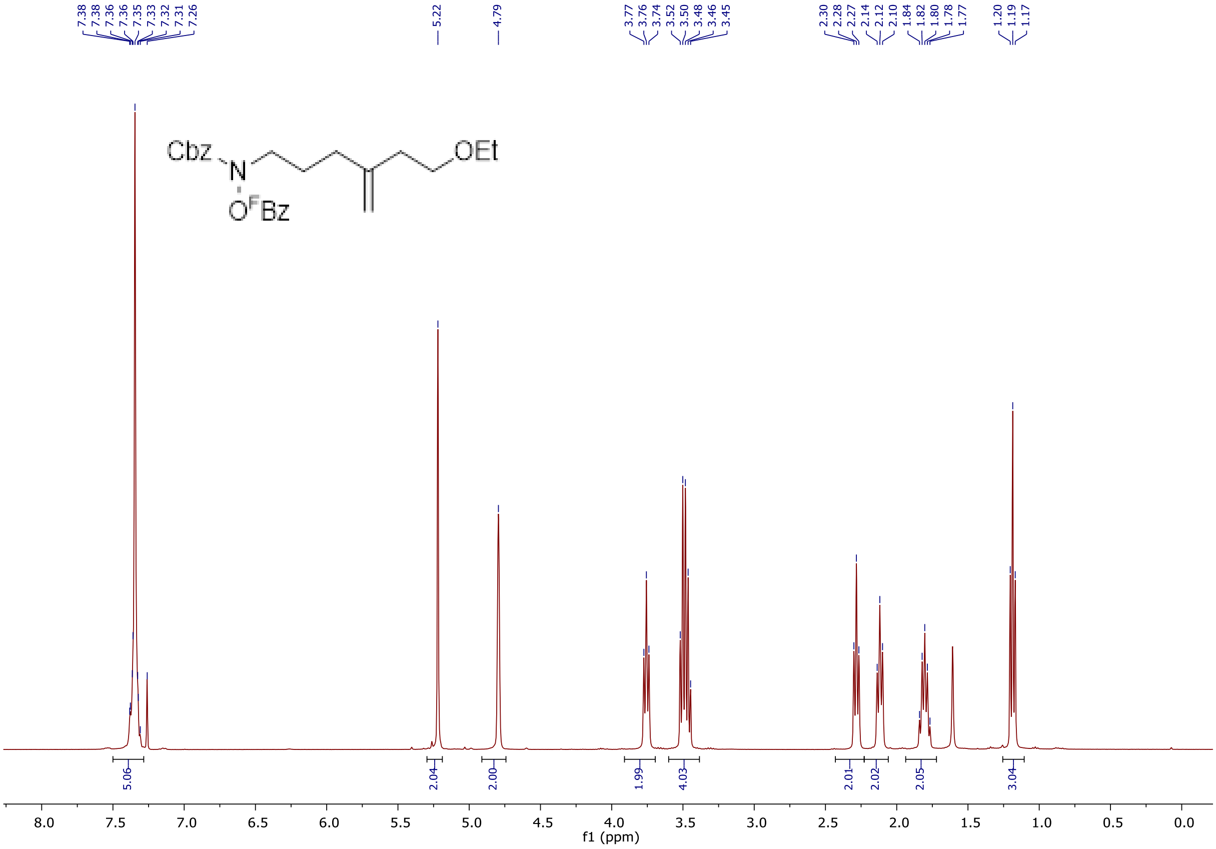

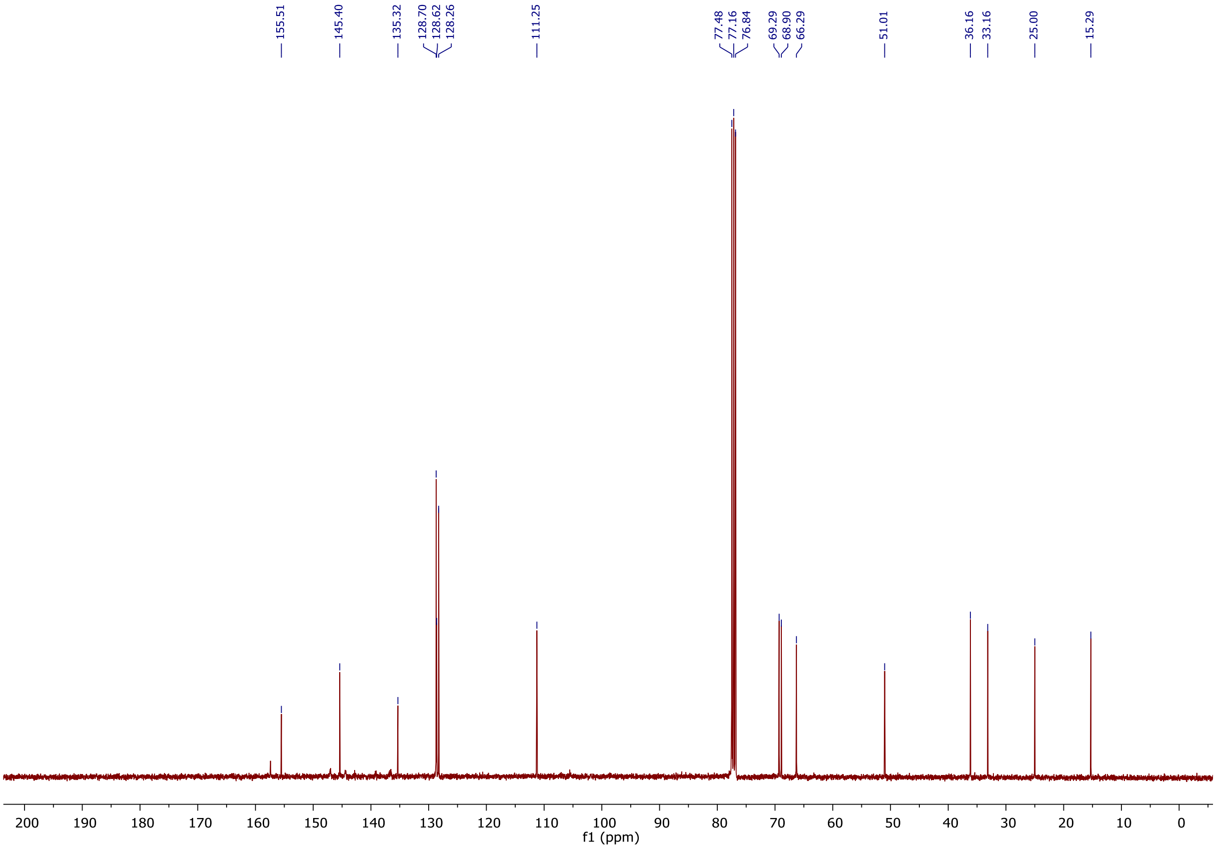

Benzyl 1-(2-ethoxyethyl)-2-azabicyclo[3.1.0]hexane-2-carboxylate (2d)

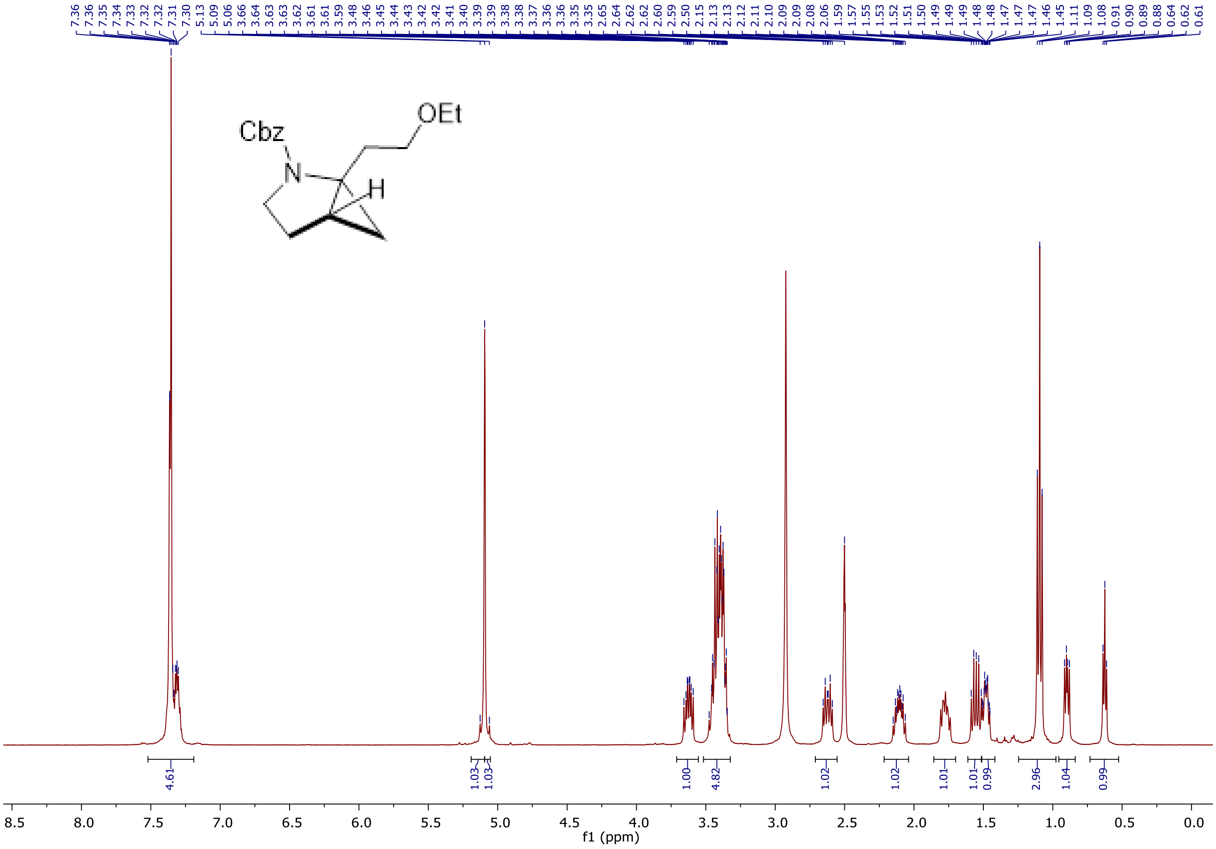

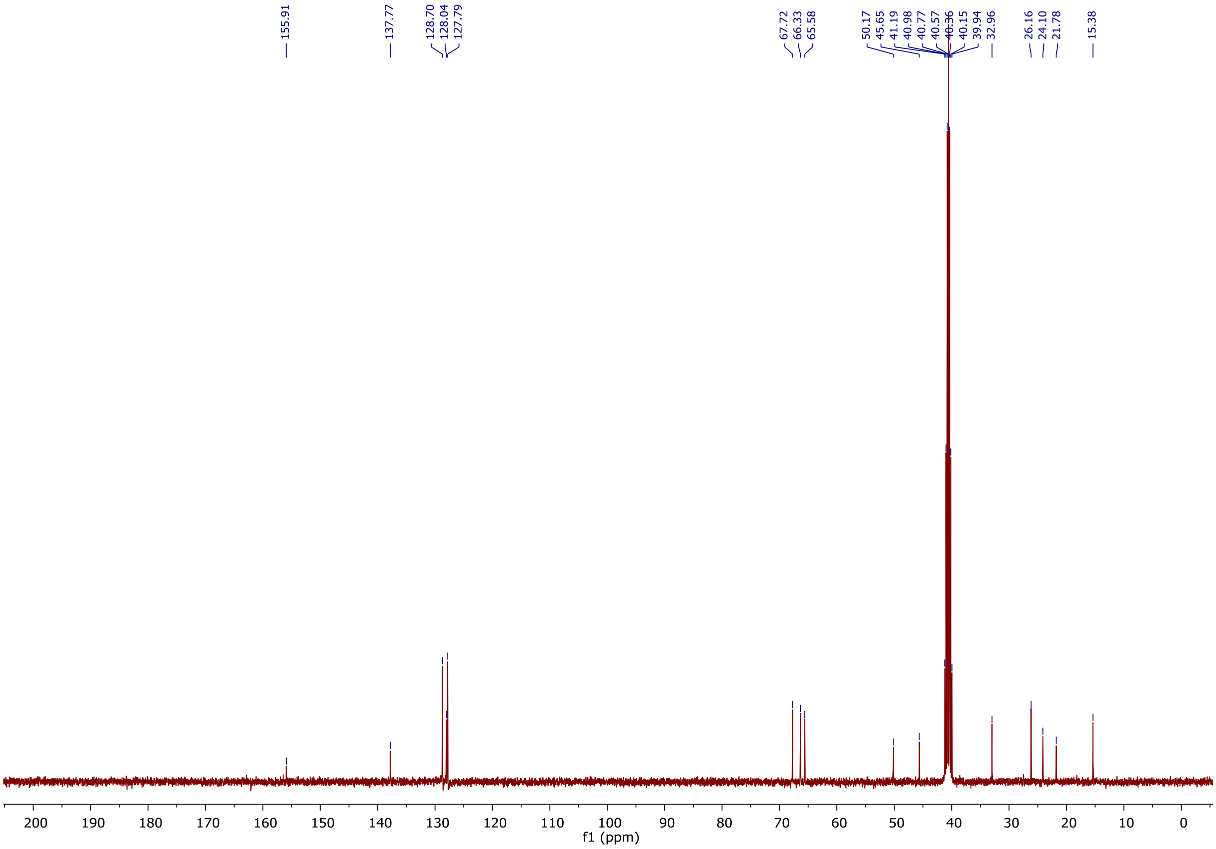

4-Methylene-7-phenylheptan-1-ol

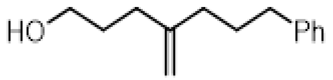

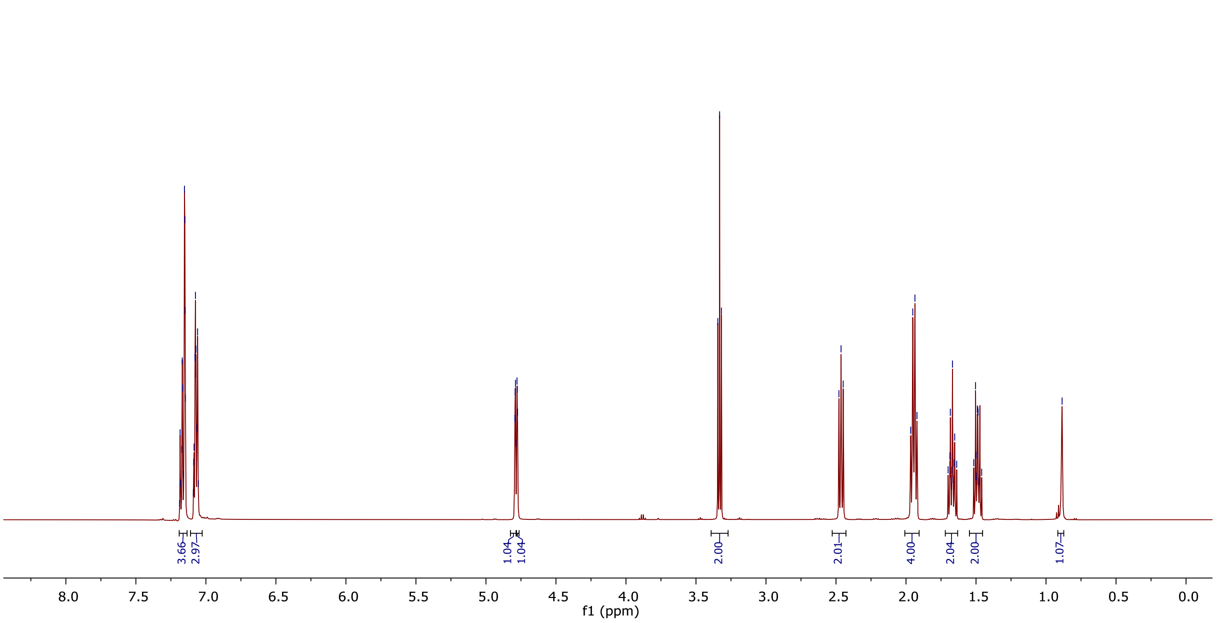

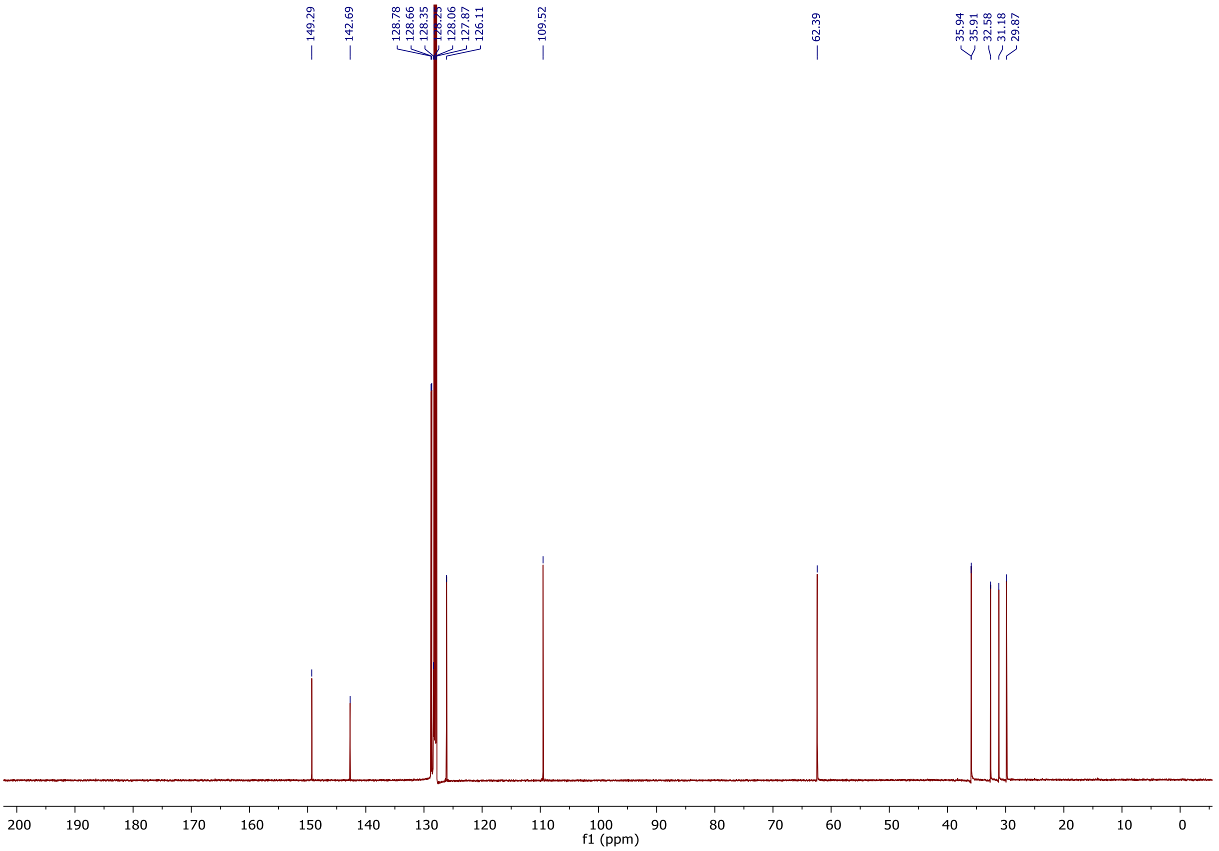

Benzyl (4-methylene-7-phenylheptyl)((perfluorobenzoyl)oxy)carbamate (1e)

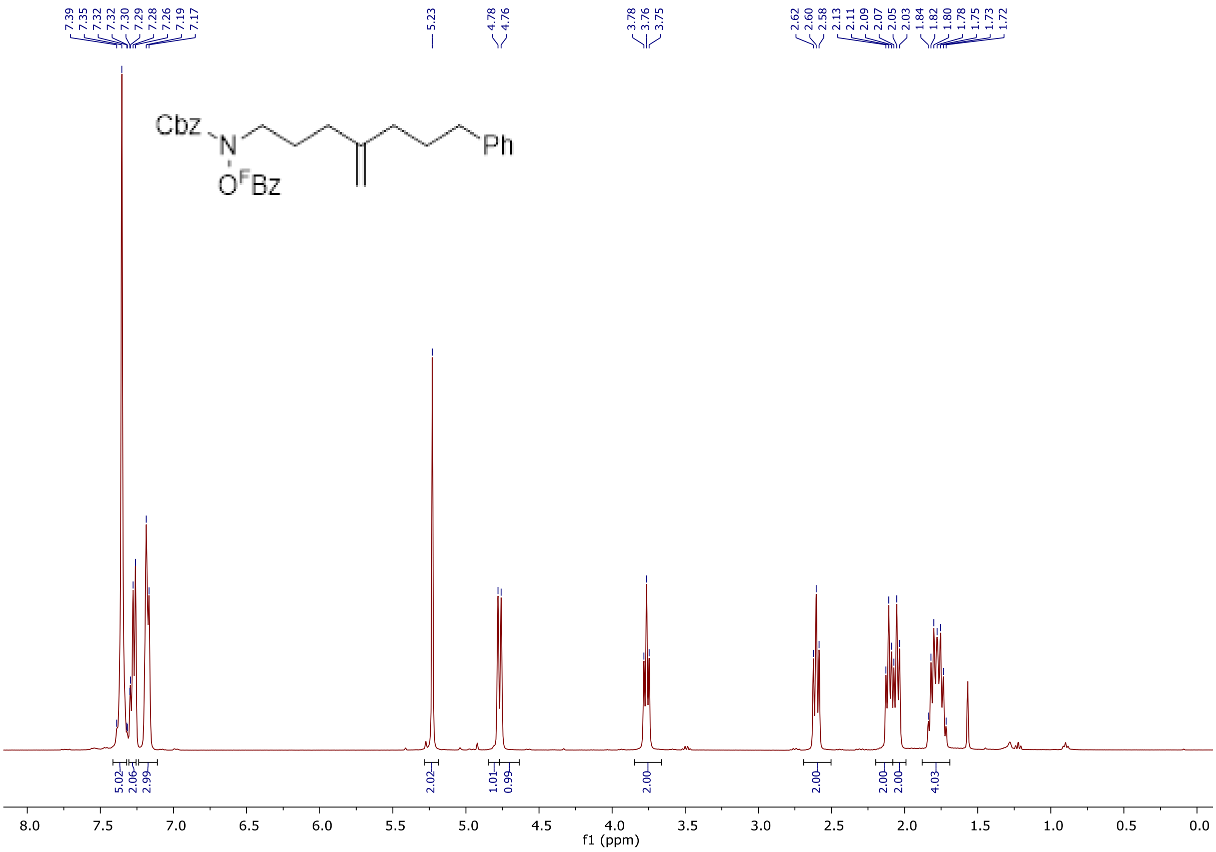

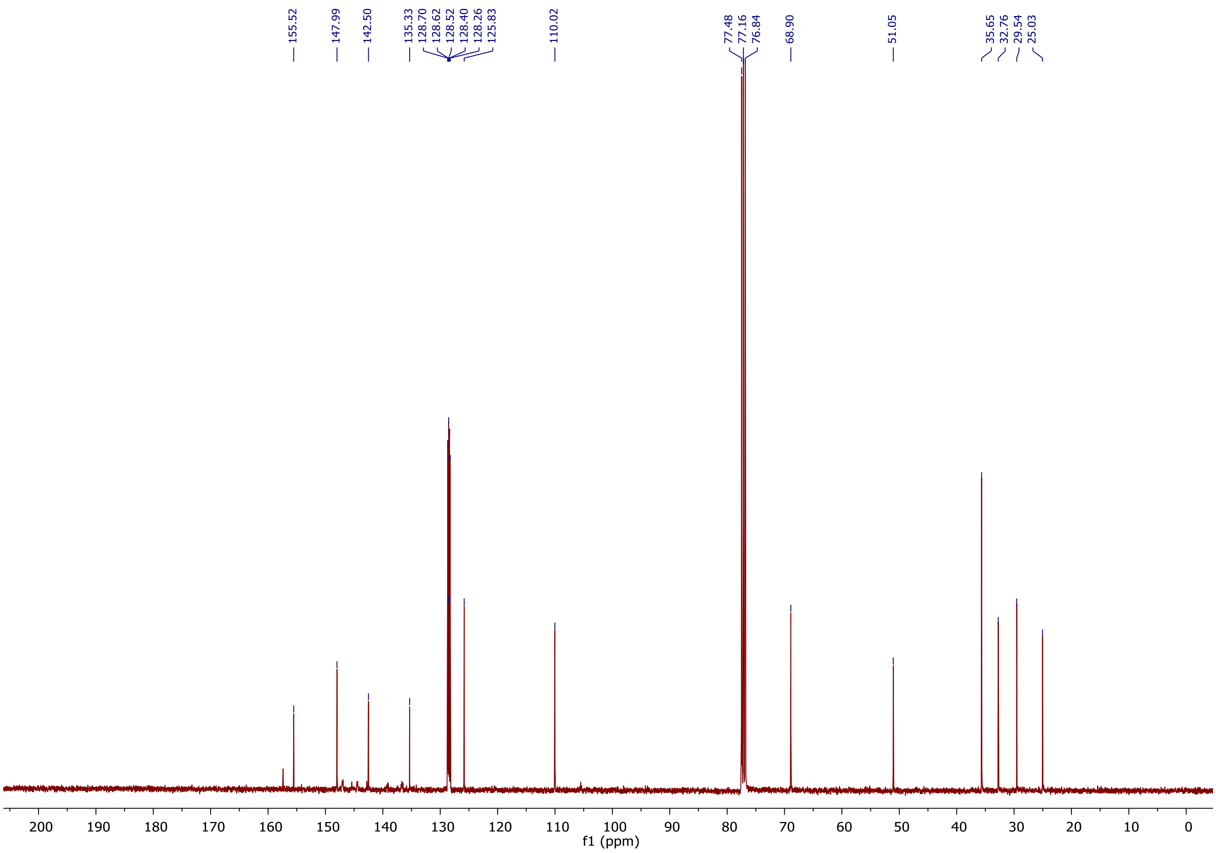

Benzyl 1-(3-phenylpropyl)-2-azabicyclo[3.1.0]hexane-2-carboxylate (2e)

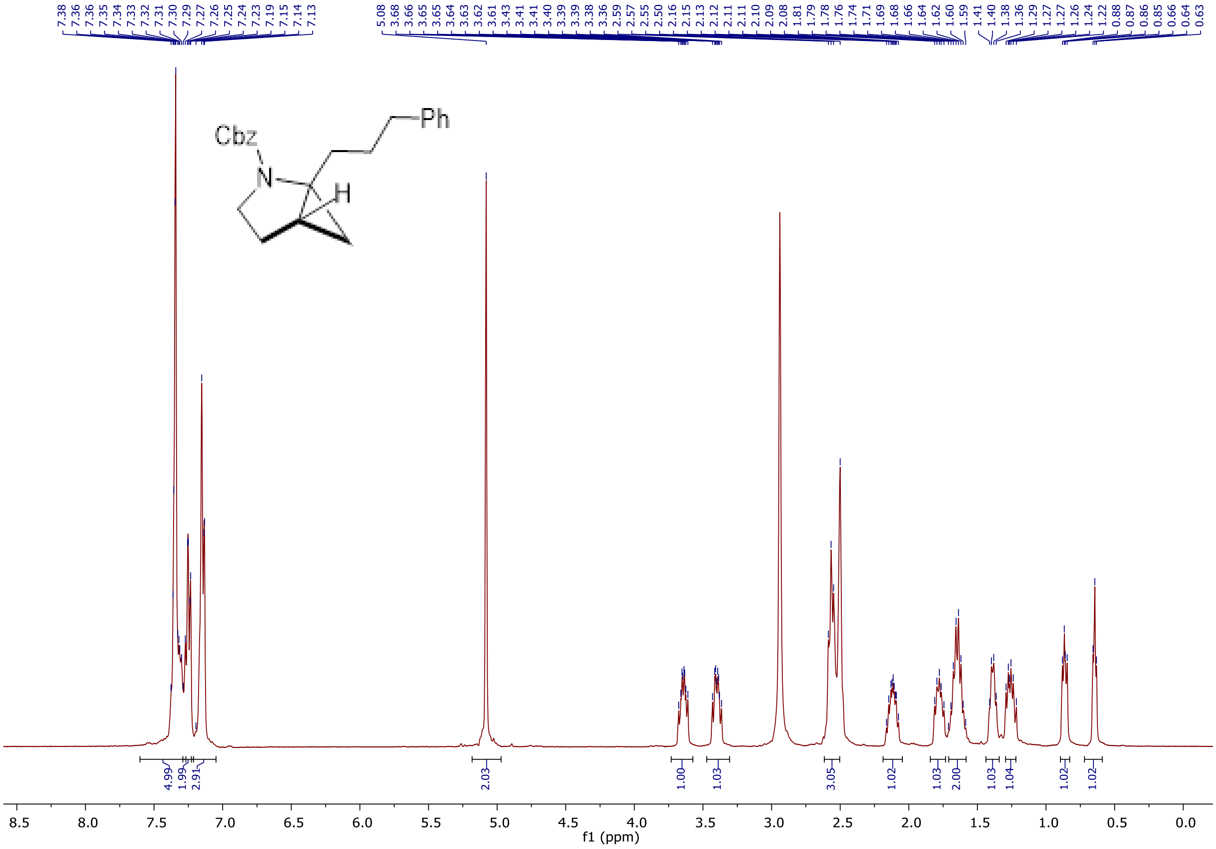

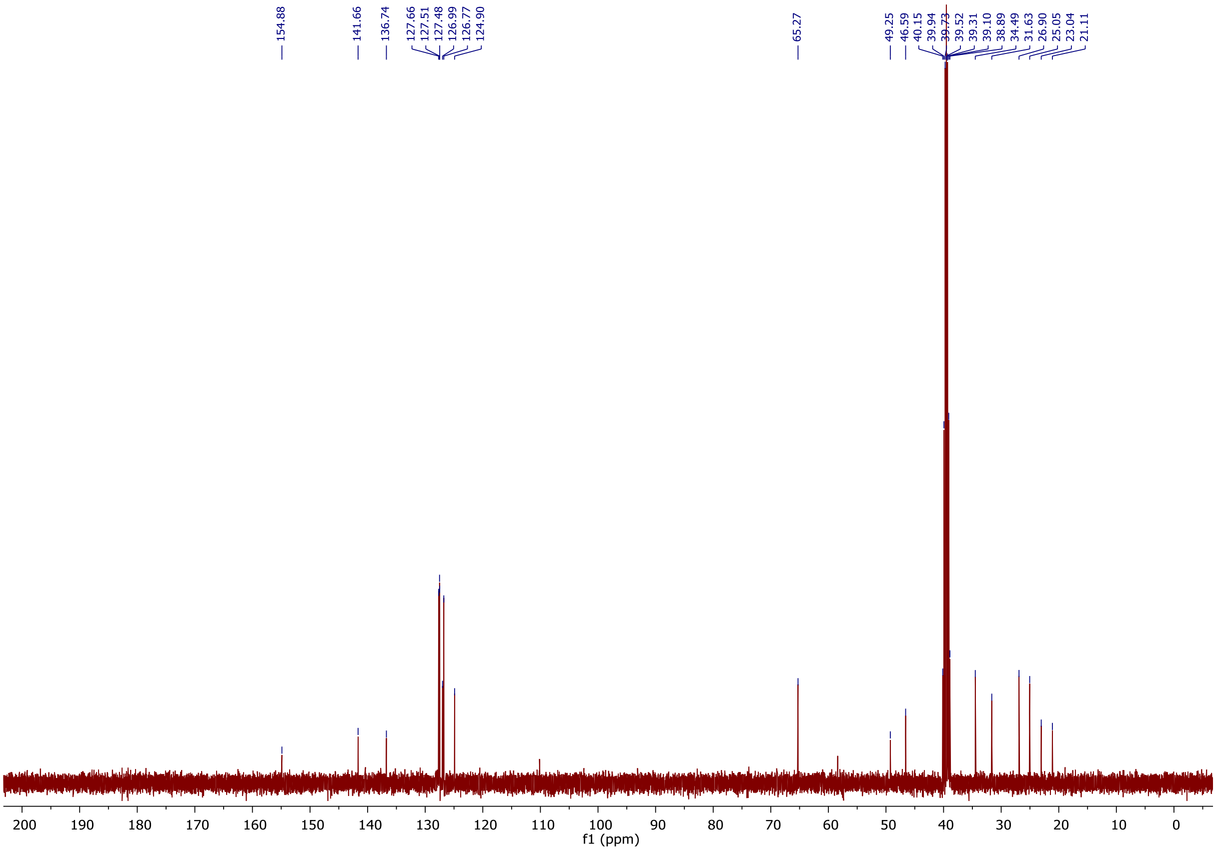

4-Cyclopentylpent-4-enoic acid

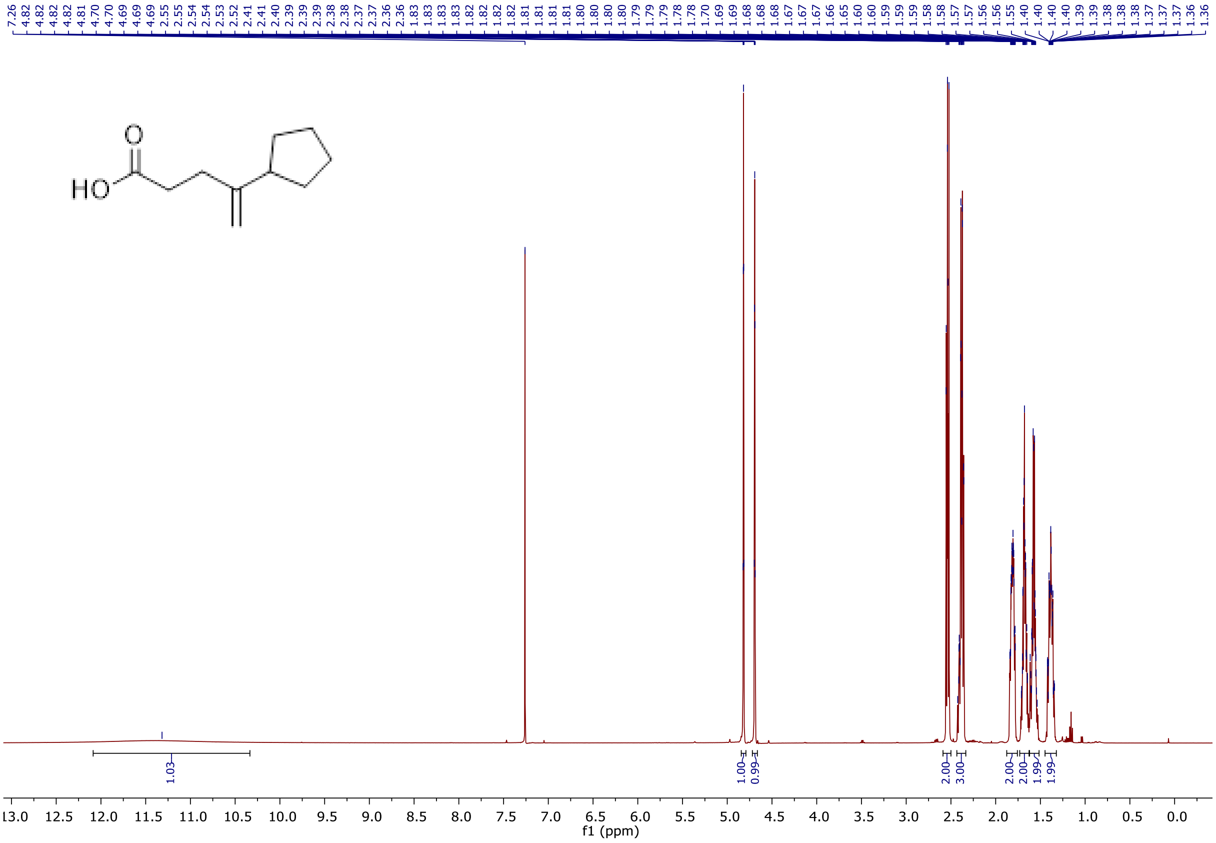

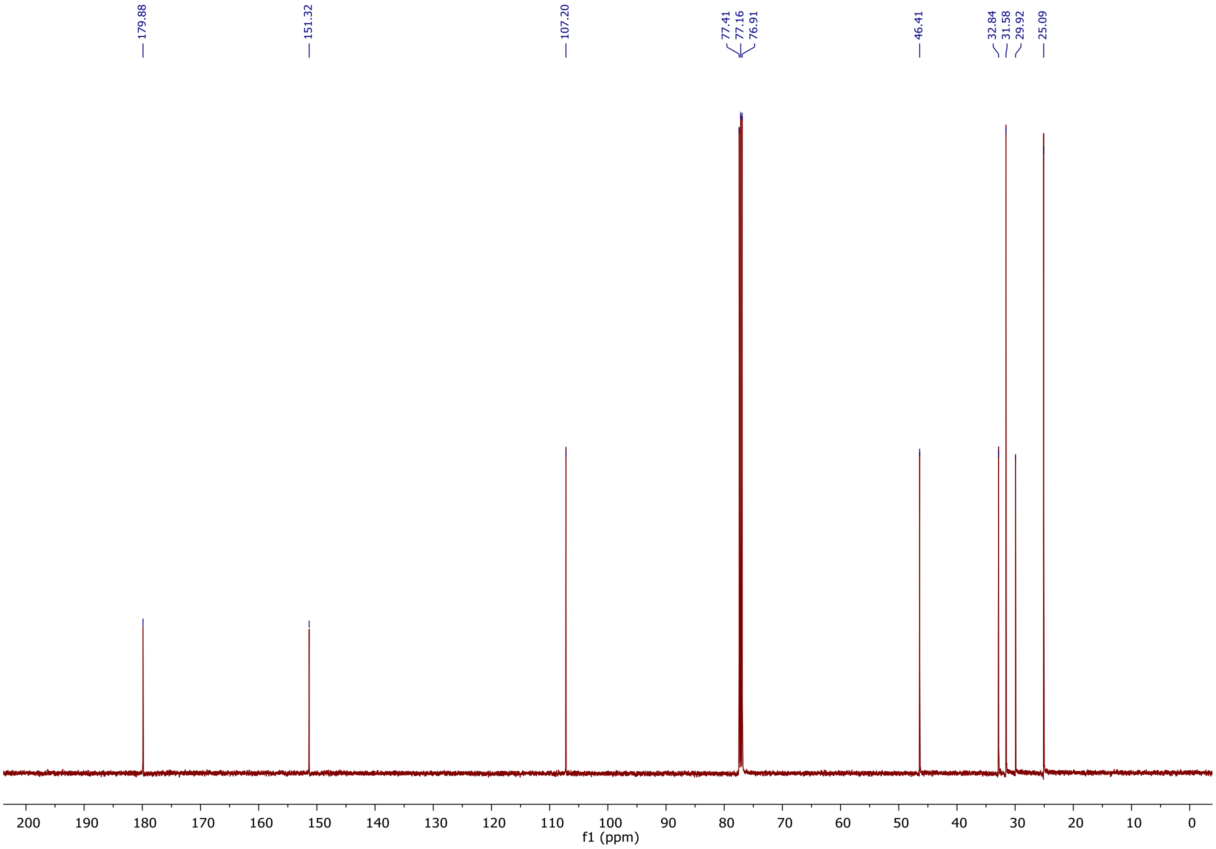

4-Cyclopentylpent-4-en-1-ol

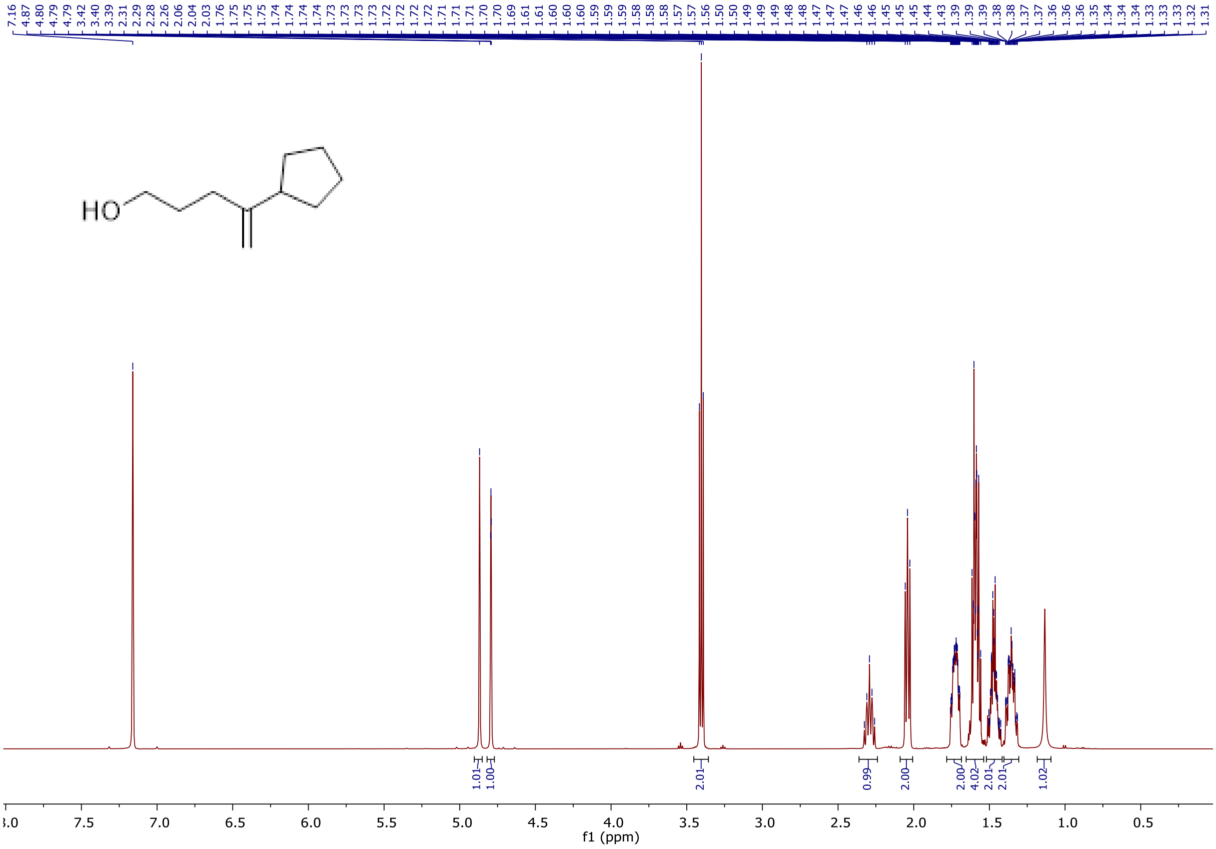

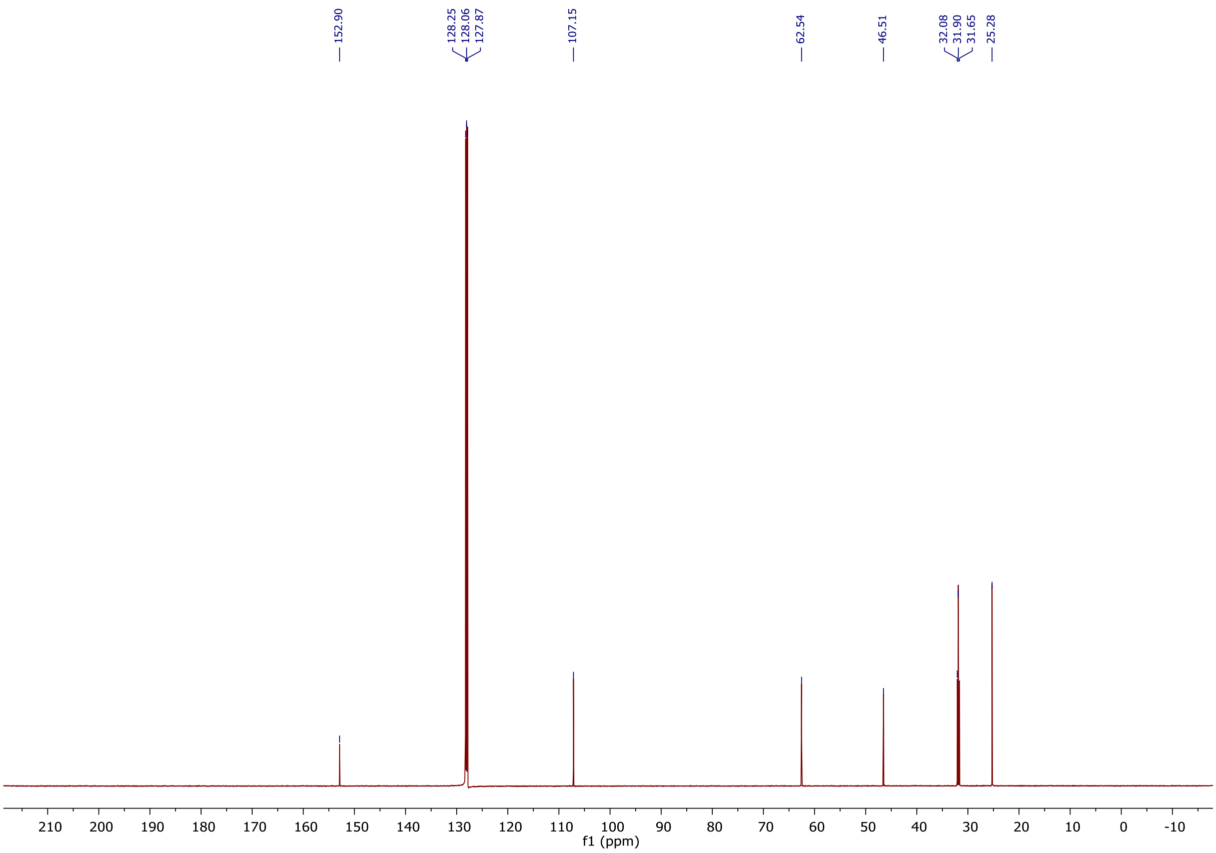

Benzyl (4-cyclopentylpent-4-en-1-yl)((perfluorobenzoyl)oxy)carbamate (1f)

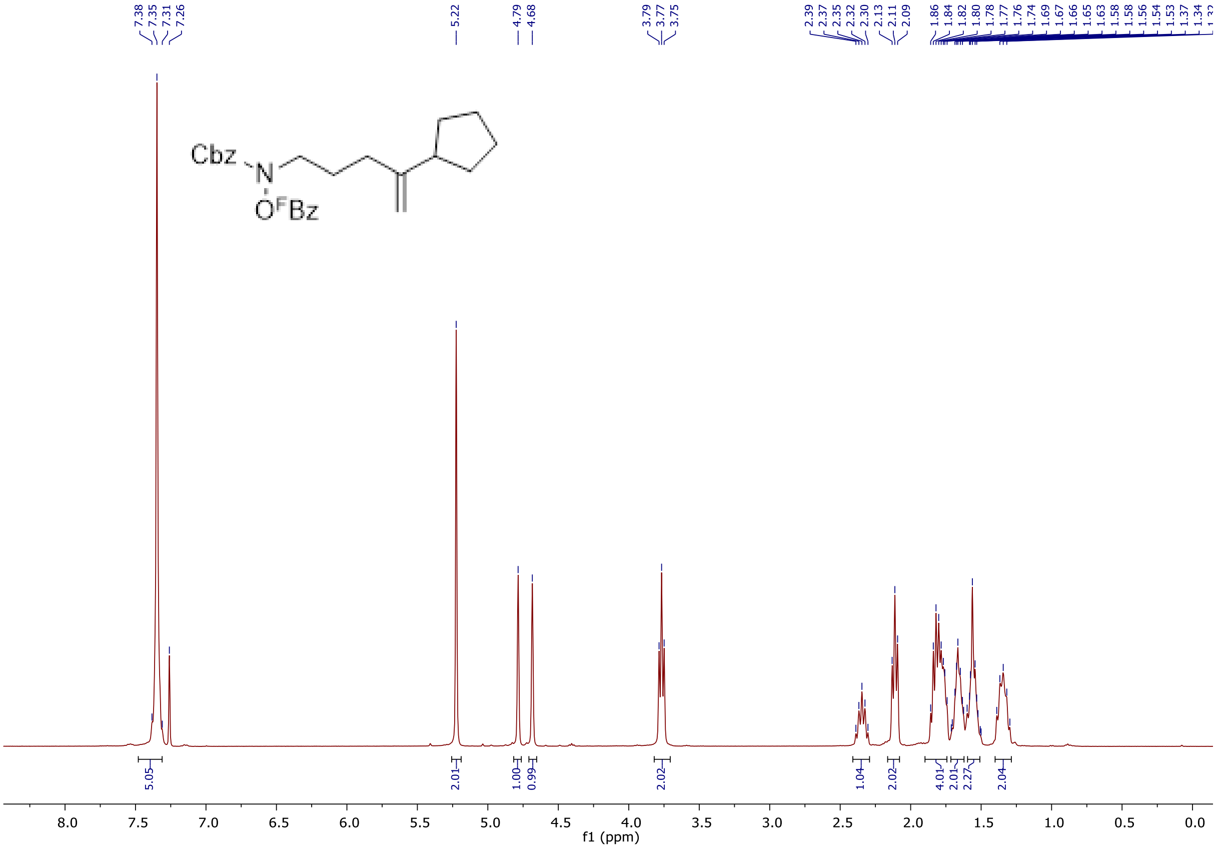

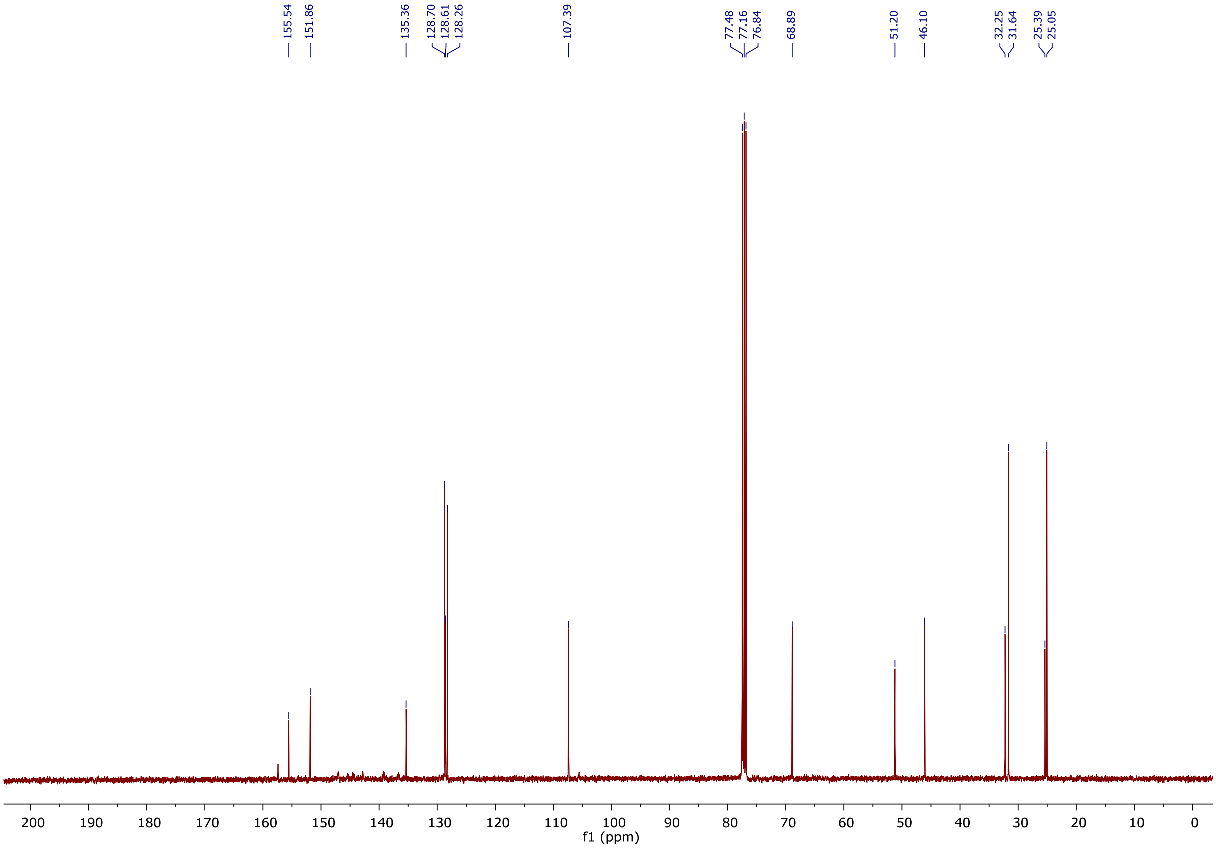

Benzyl 1-cyclopentyl-2-azabicyclo[3.1.0]hexane-2-carboxylate (2f)

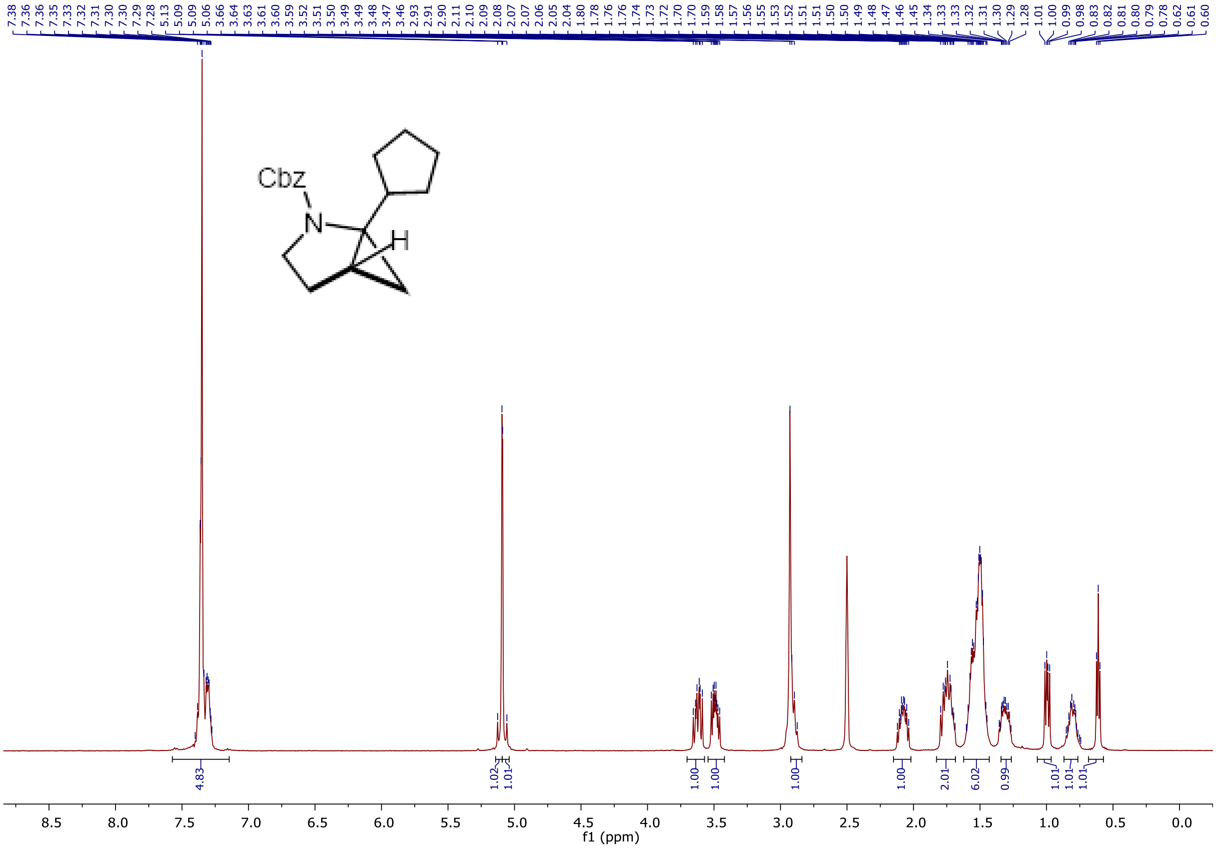

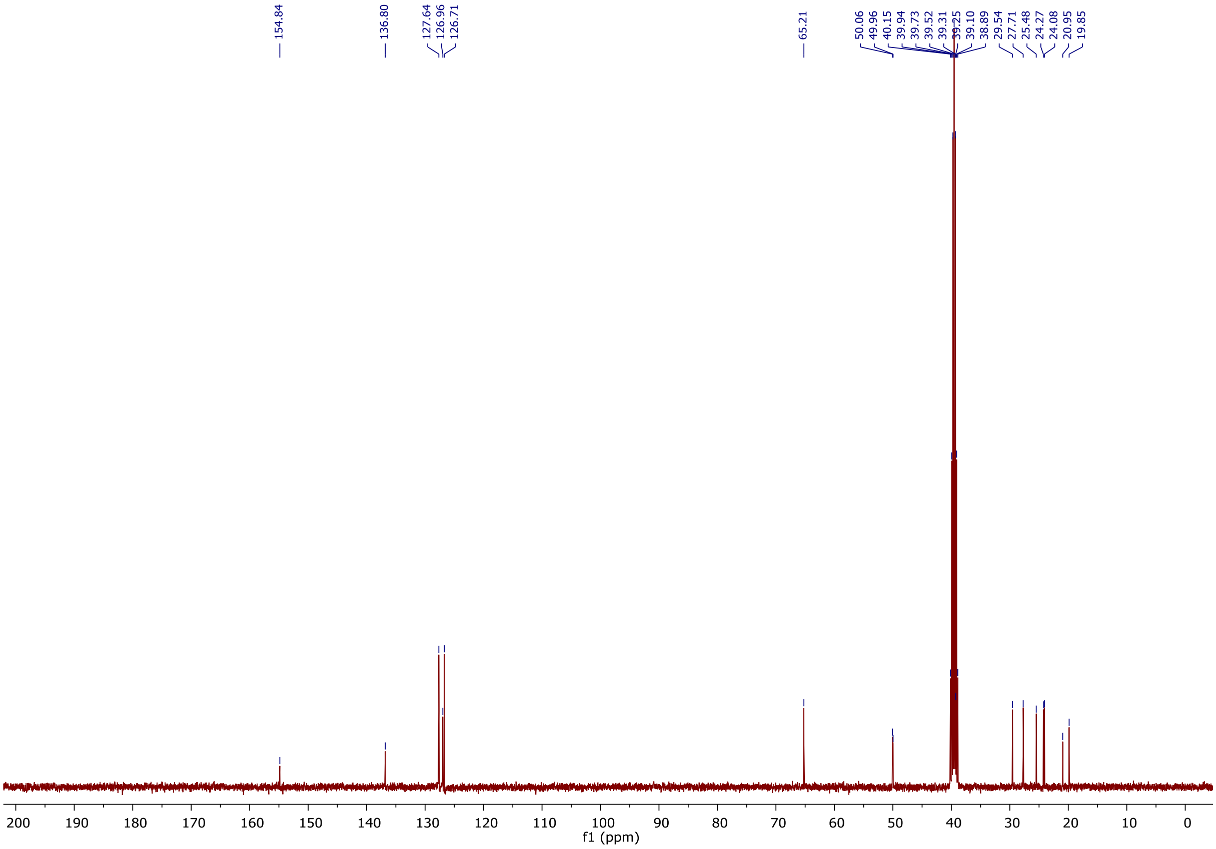

Benzyl ((perfluorobenzoyl)oxy)(4-phenylpent-4-en-1-yl)carbamate (1g)

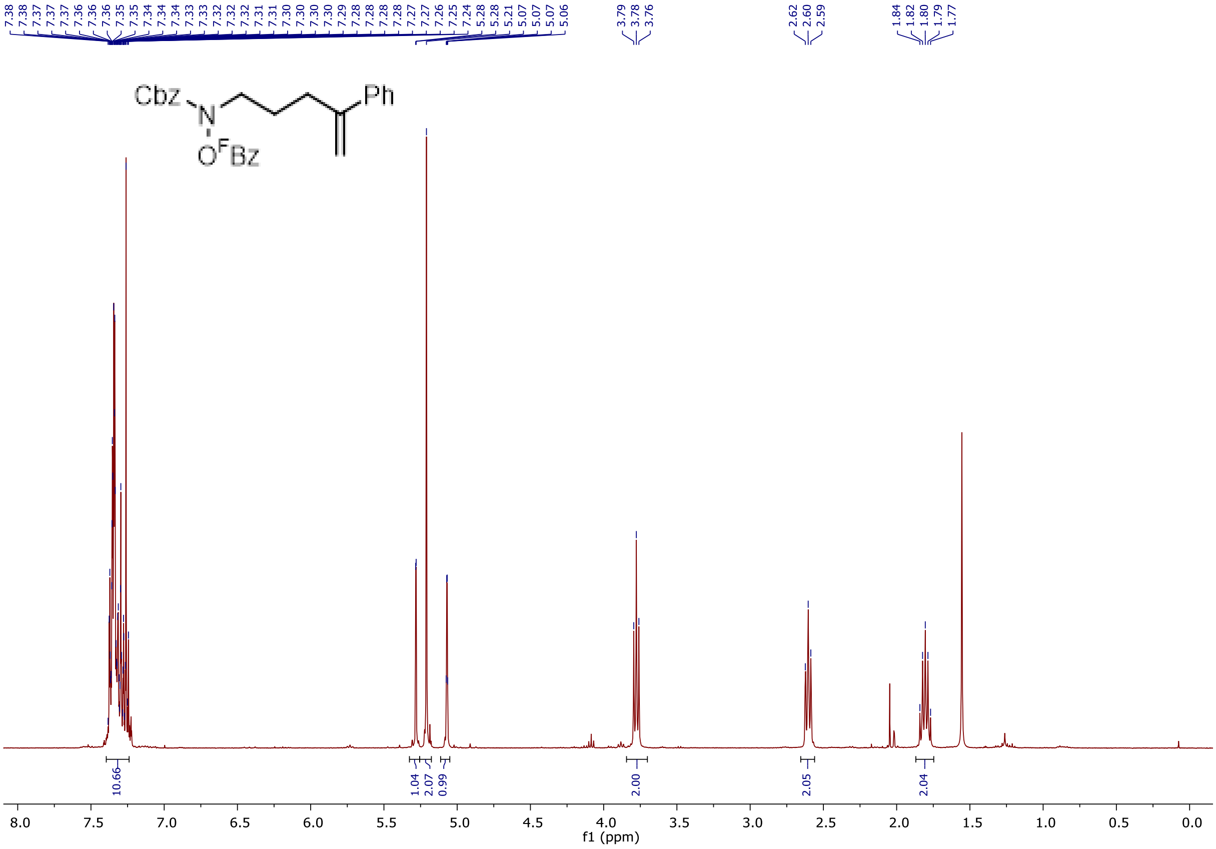

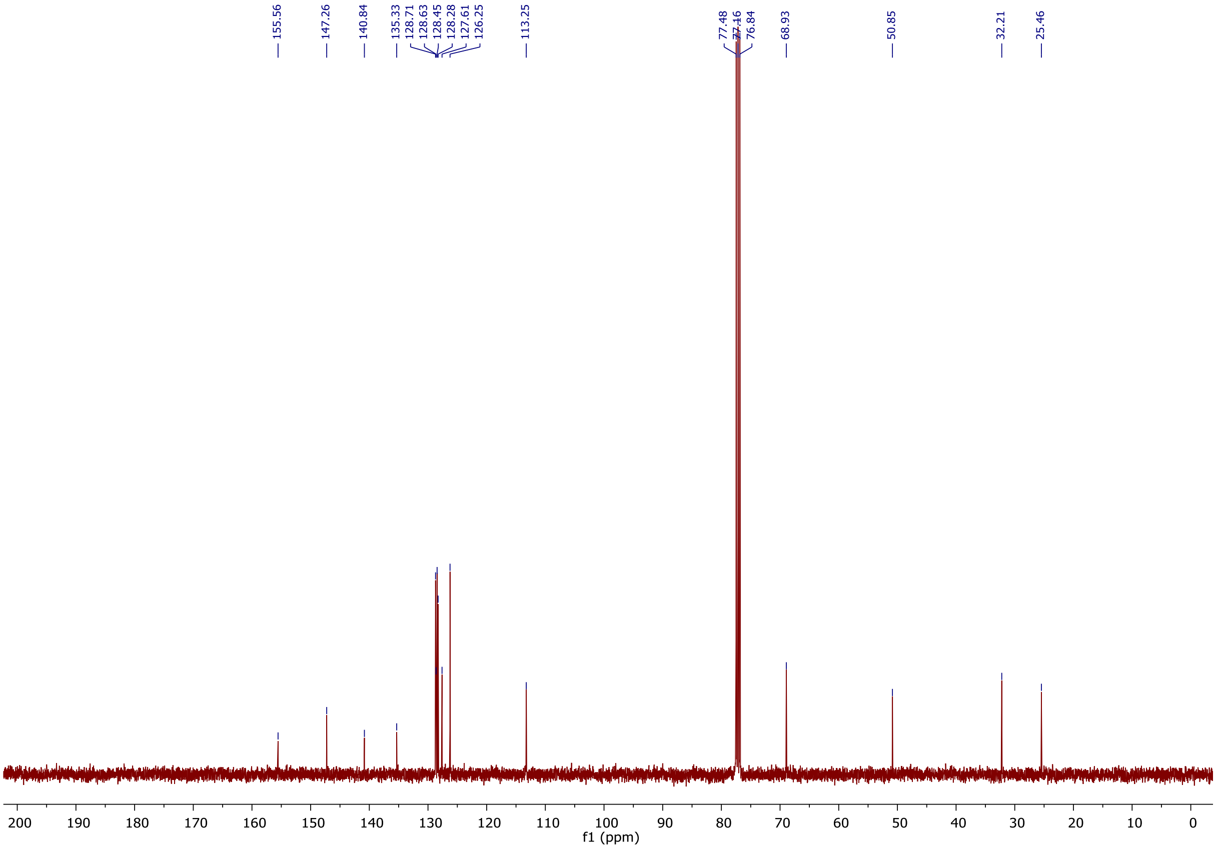

Benzyl 1-phenyl-2-azabicyclo[3.1.0]hexane-2-carboxylate (2g)

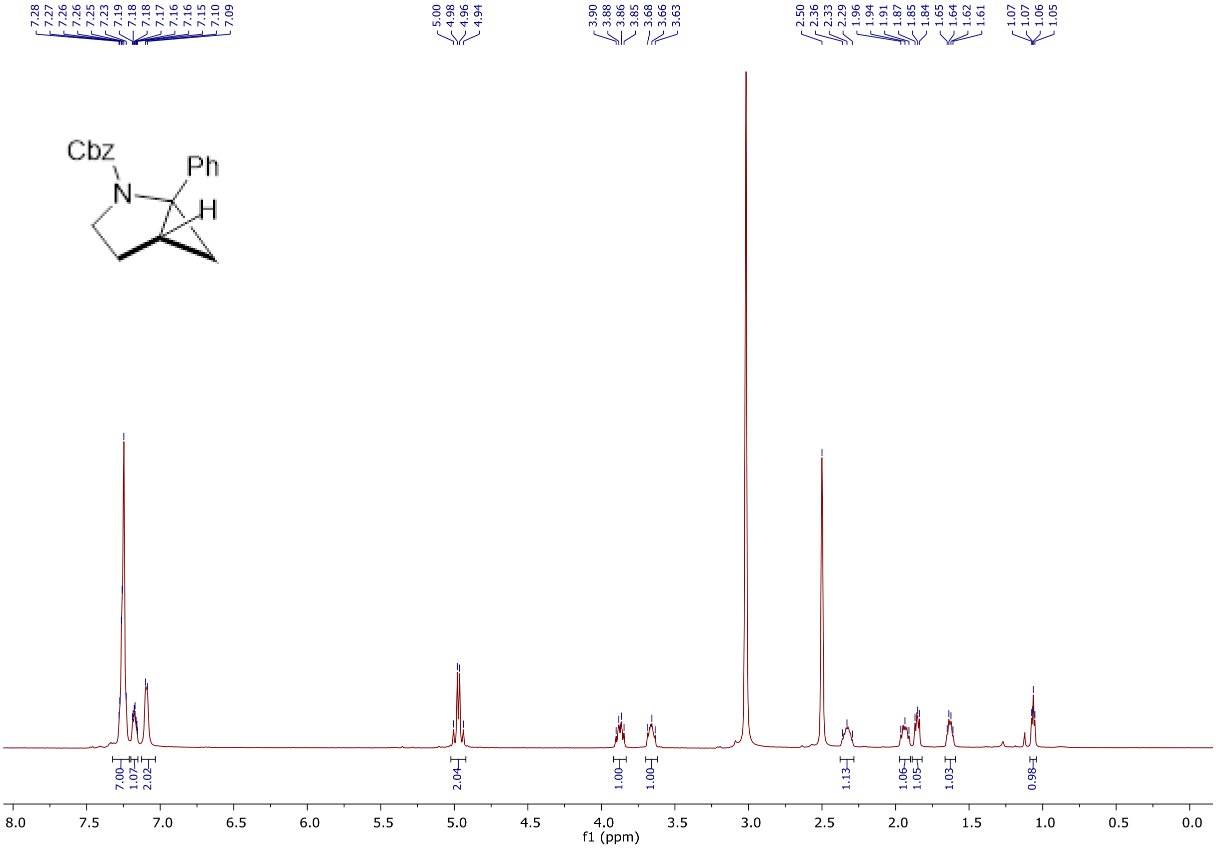

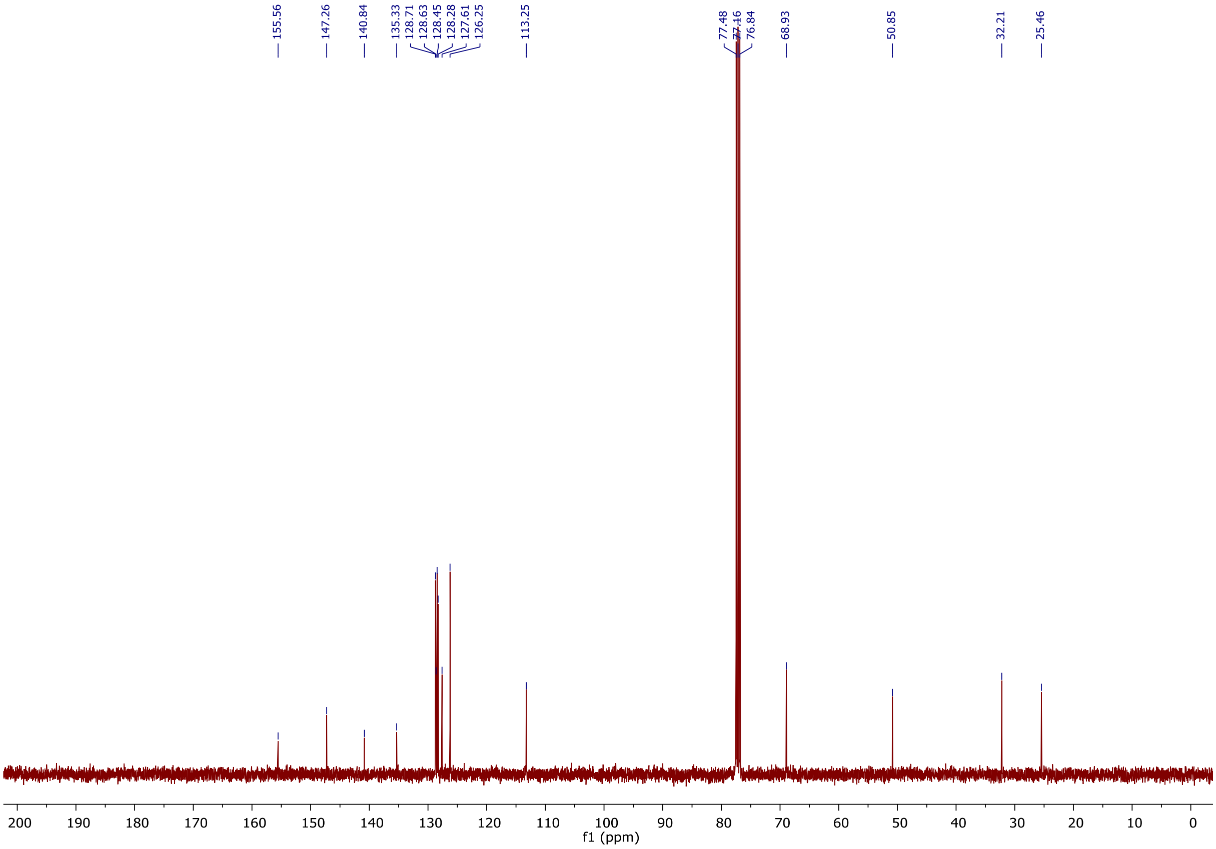

(*S*)-4-Benzyl-3-((*R*)-2-ethyl-4-methylenehexanoyl)oxazolidin-2-one

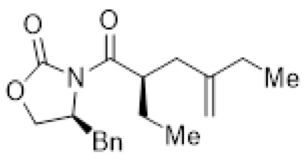

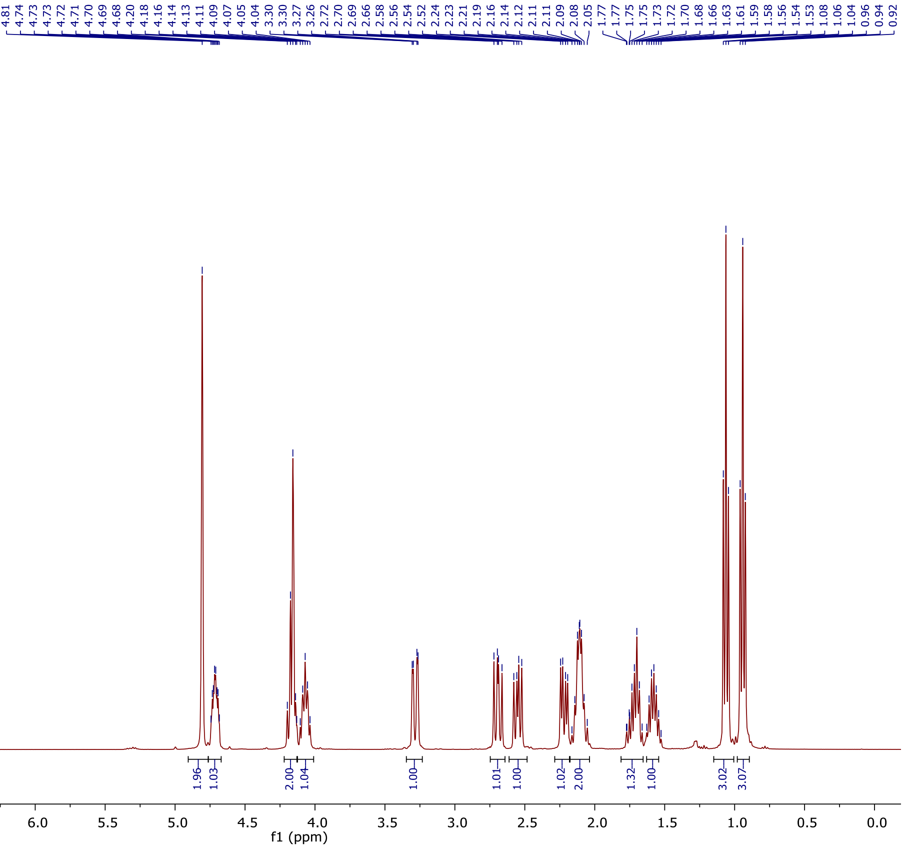

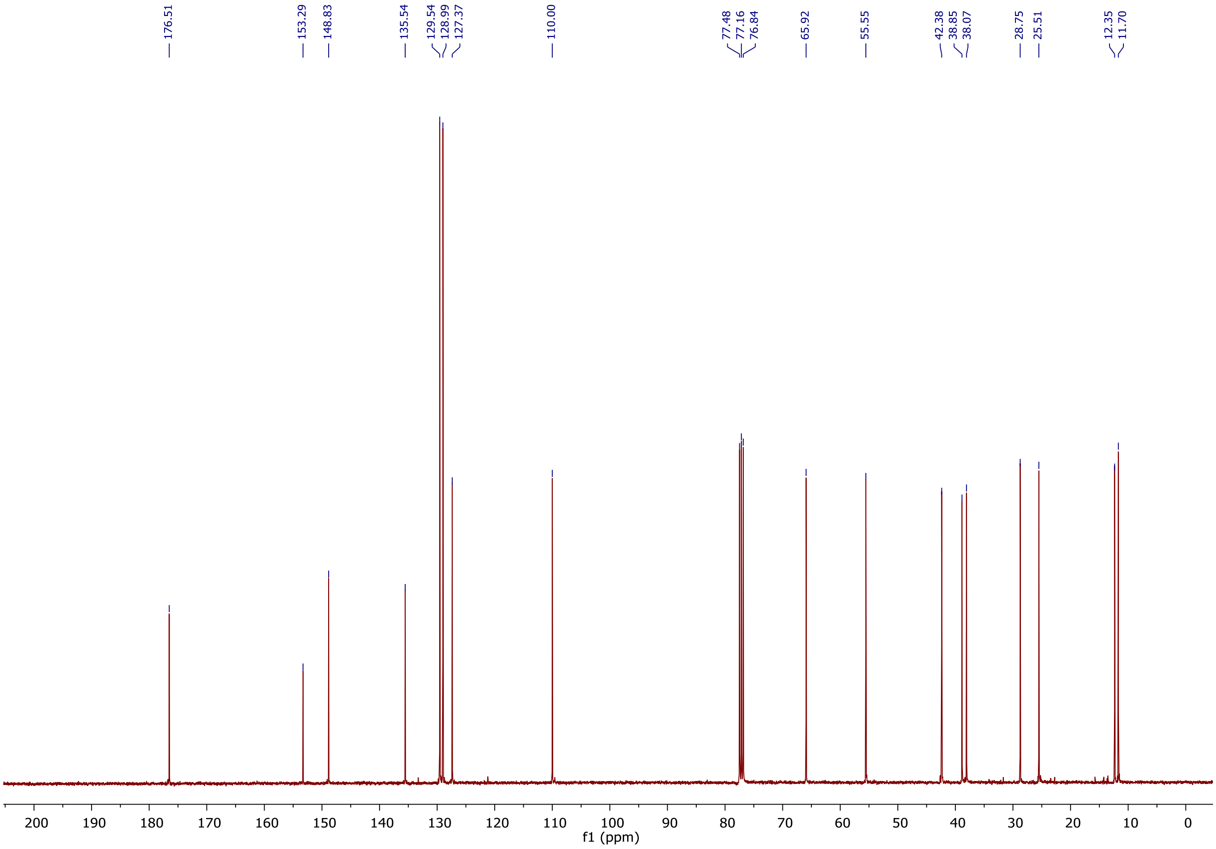

(*R*)-2-Ethyl-4-methylenehexan-1-ol

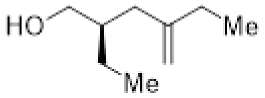

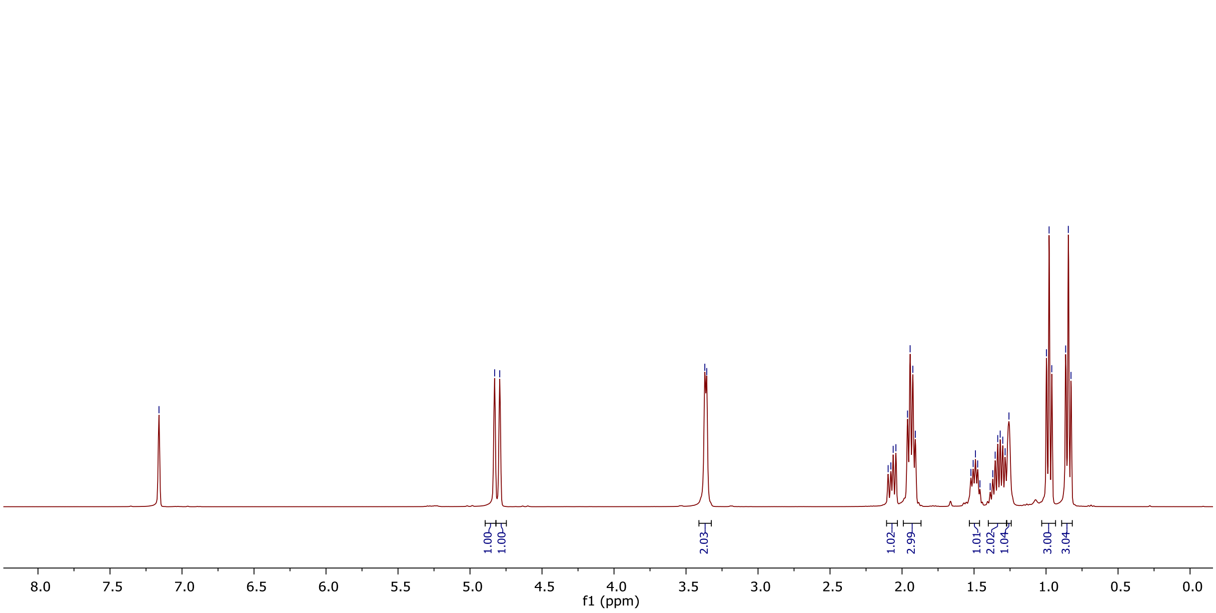

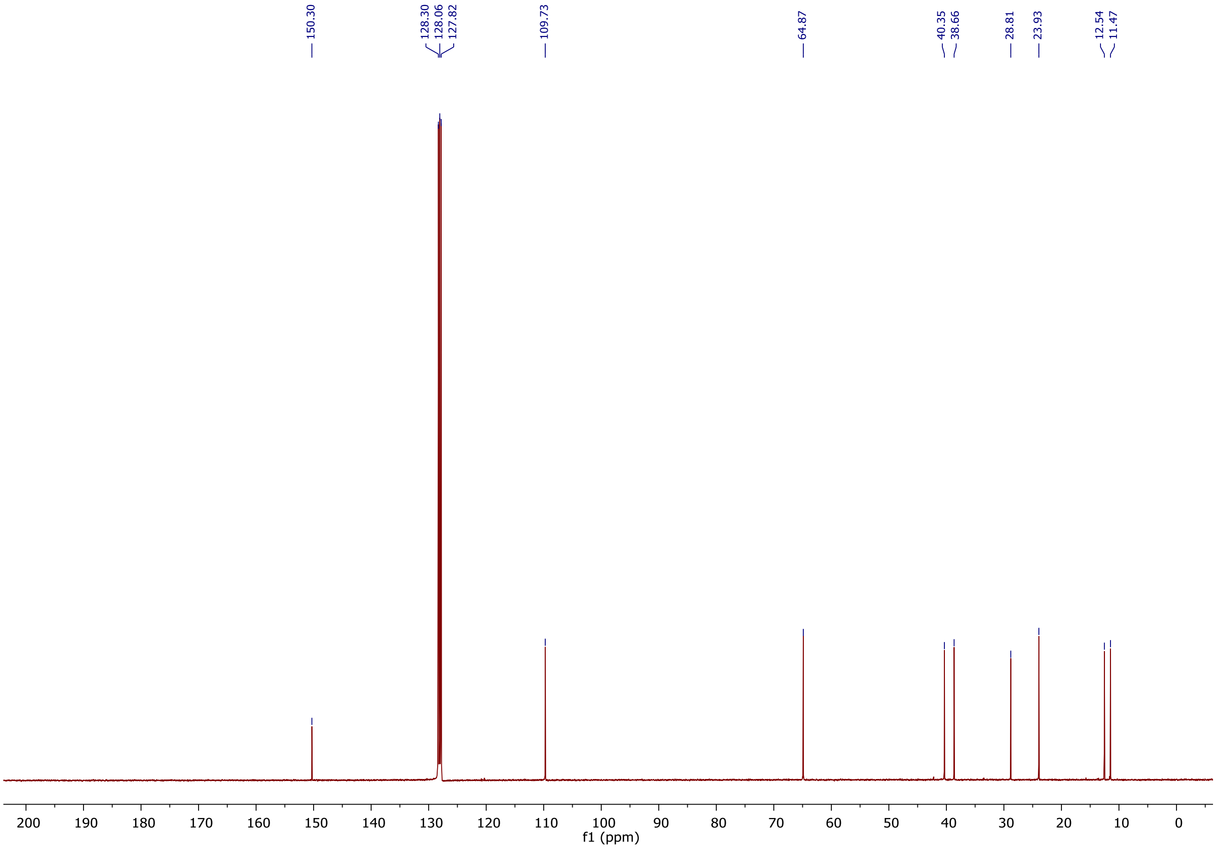

Benzyl (*R*)-(2-ethyl-4-methylenehexyl)((perfluorobenzoyl)oxy)carbamate (1h)

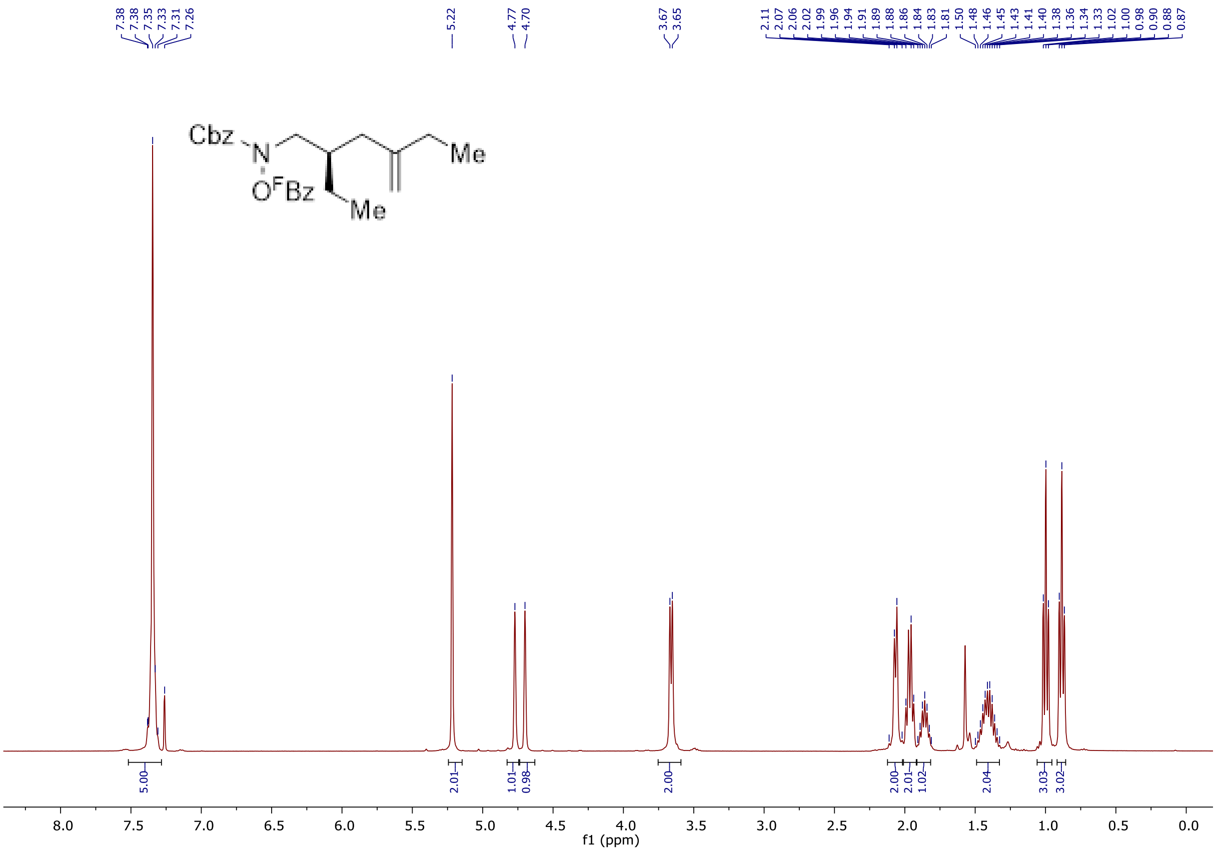

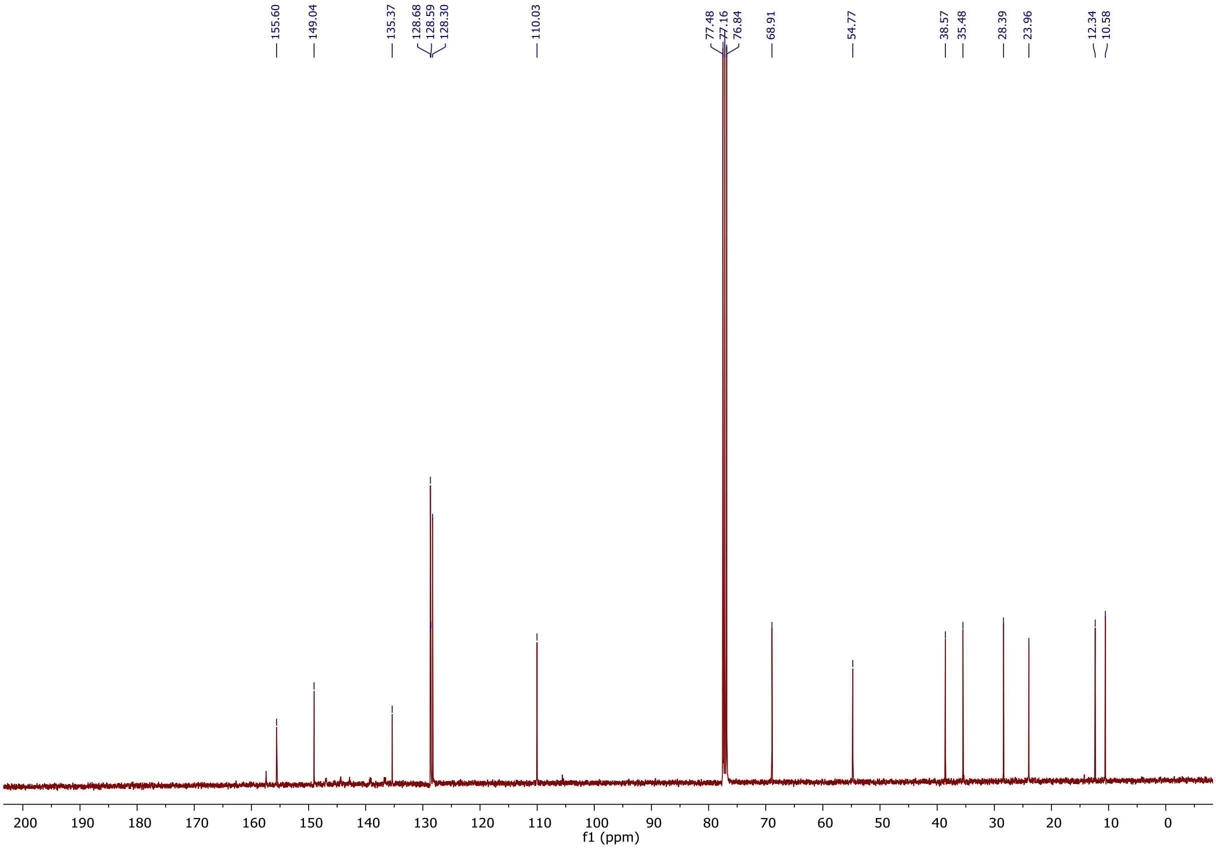

Benzyl (4*R*)-1,4-diethyl-2-azabicyclo[3.1.0]hexane-2-carboxylate (2h)

Isomer 1

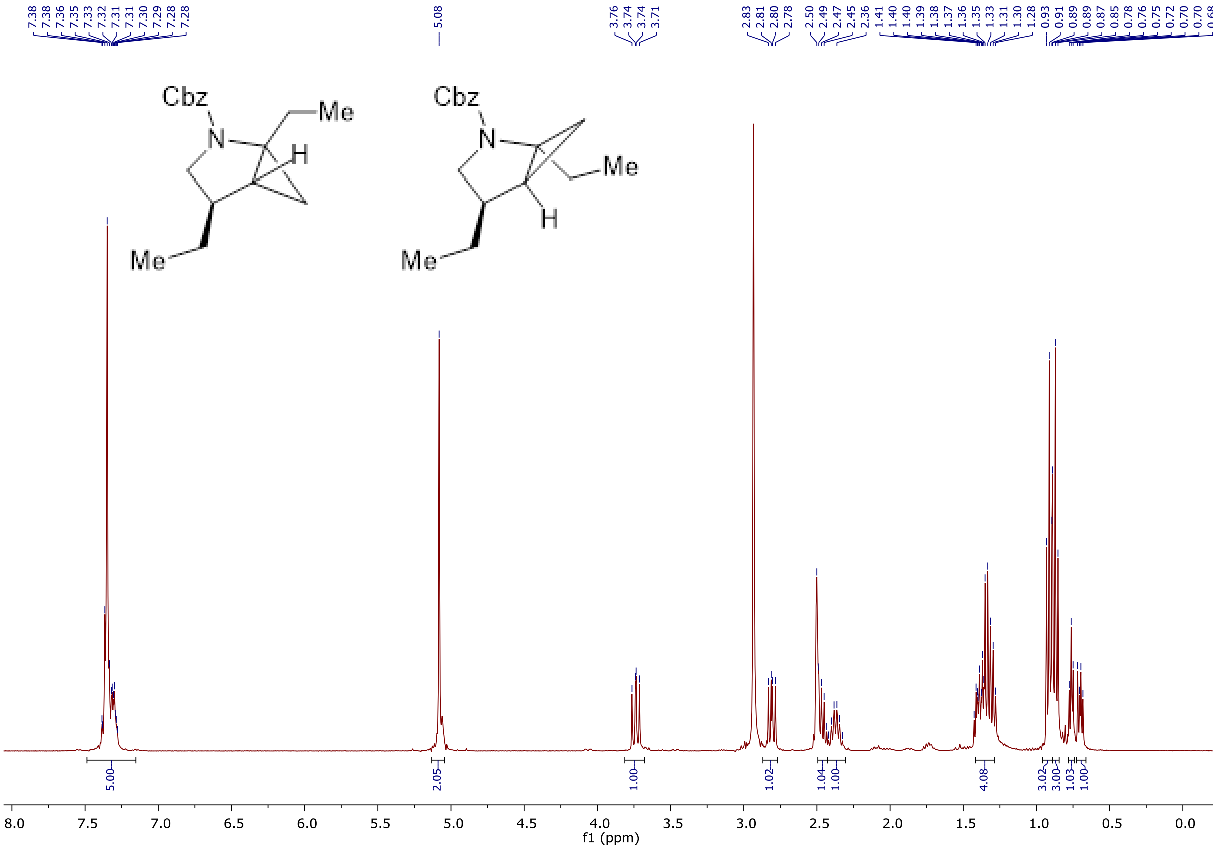

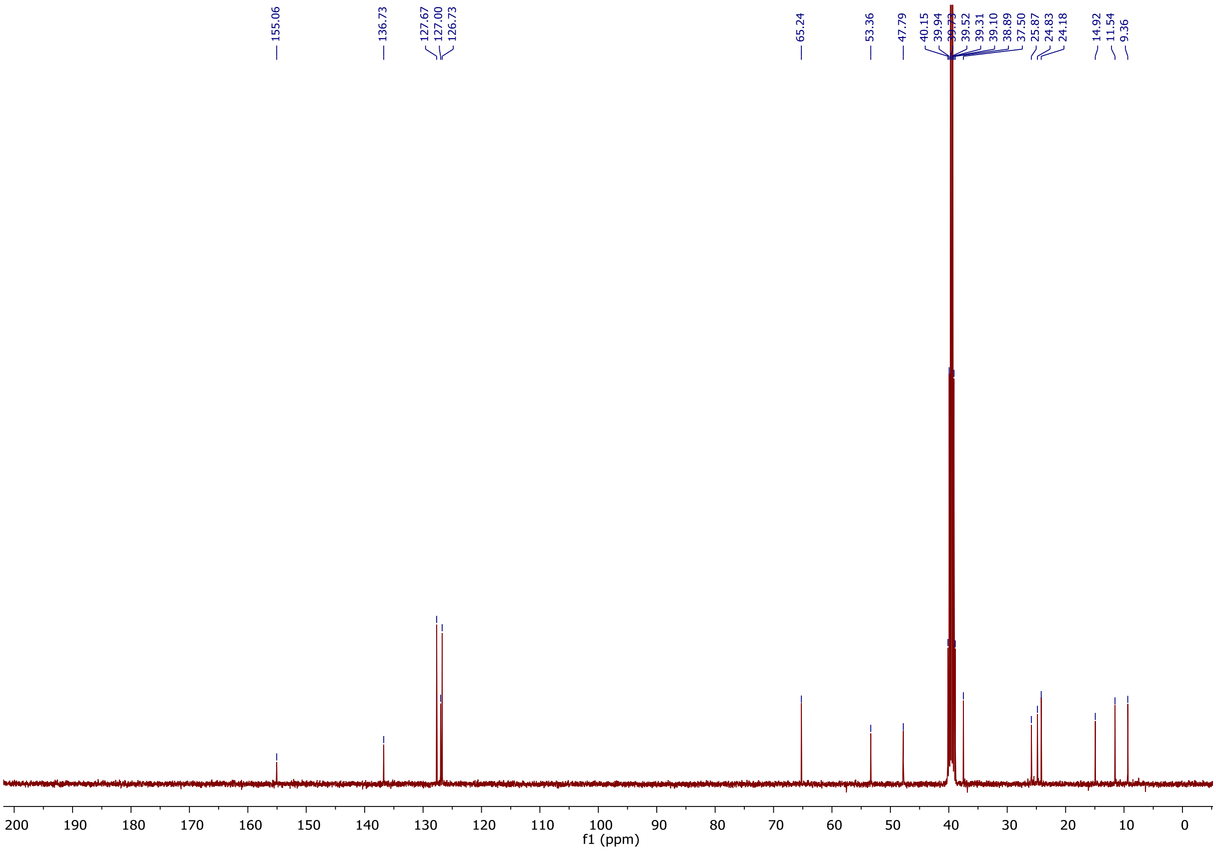

Isomer 2

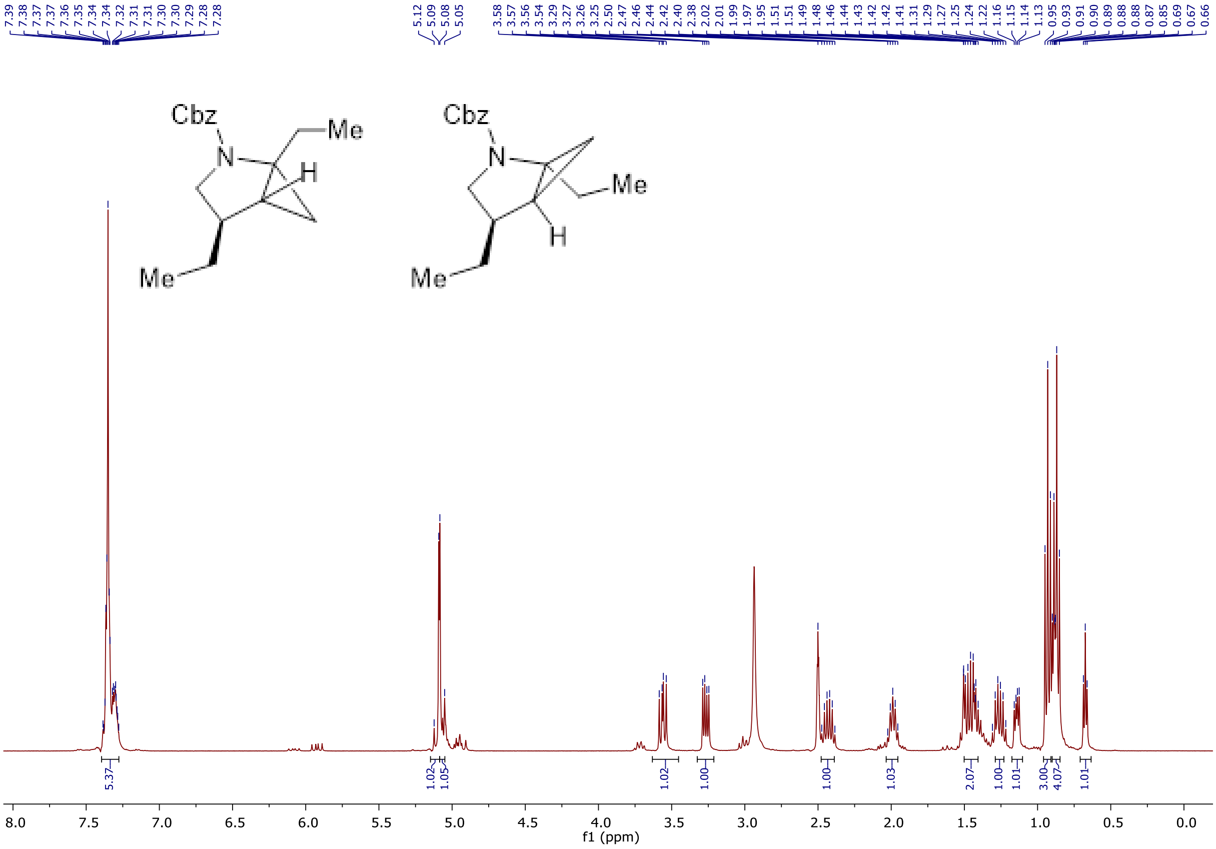
7.39 7.38 7.37 7.37 7.36 7.35 7.34 7.34 7.32 7.31 7.31 7.30 7.29 7.28 7.28
5.12 5.09 5.08 5.05
3.58 3.57 3.56 3.54 3.29 3.27 3.26 3.25 2.50 2.47 2.46 2.44 2.42 2.40 2.38 2.02 2.01 1.99 1.97 1.95 1.51 1.51 1.49 1.48 1.46 1.44 1.43 1.42 1.42 1.41 1.31 1.29 1.27 1.25 1.24 1.22 1.16 1.15 1.14 1.13 0.95 0.93 0.91 0.90 0.89 0.88 0.88 0.87 0.85 0.69 0.67 0.66
Cbz
N
Me
H
5.37
1.02
1.05
1.02
1.00
1.00
1.03
2.07
1.00
1.01
3.00
4.07
1.01
f1 (ppm)

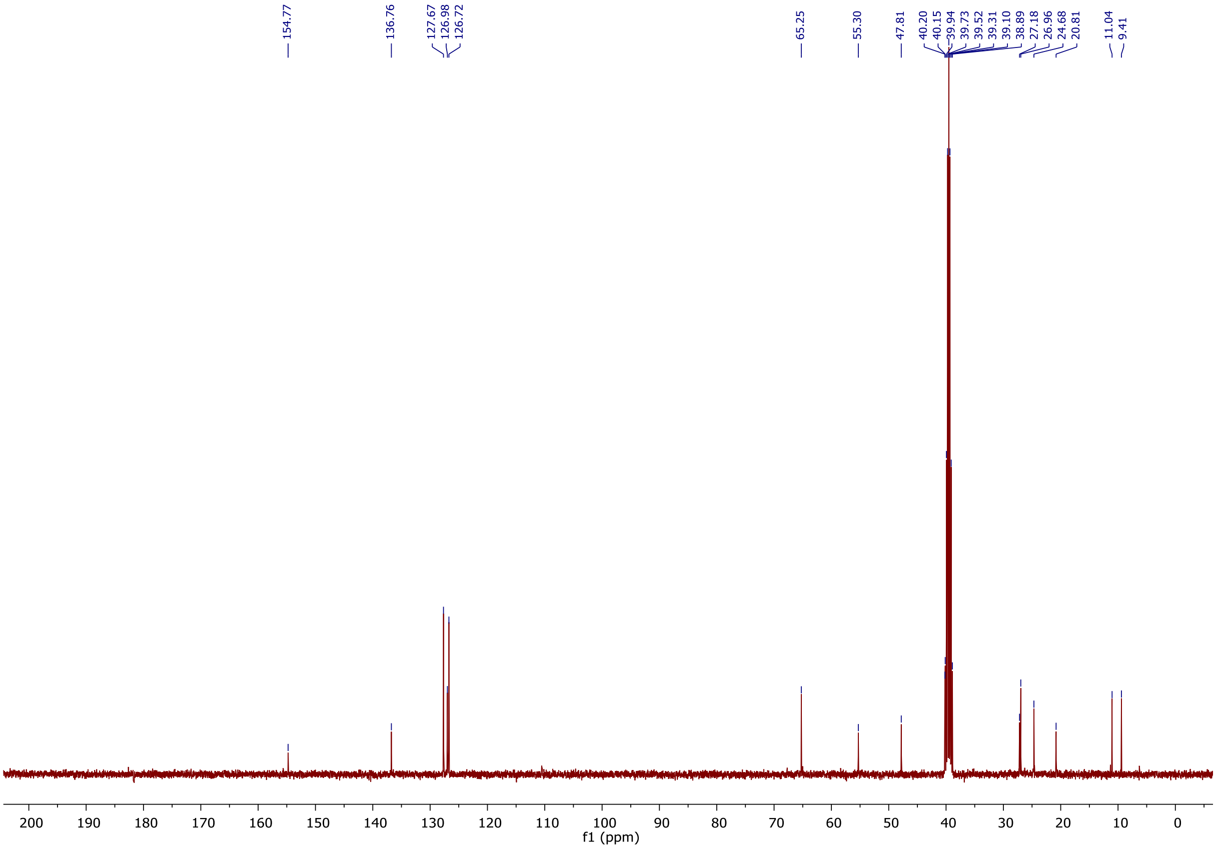
154.77
136.76
127.67 126.98 126.72
65.25
55.30
47.81
40.20 40.15 39.94 39.73 39.52 39.31 39.10 38.89 27.18 26.96 24.68 20.81
11.04 9.41
f1 (ppm)

5-Methyleneheptan-2-ol

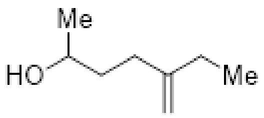

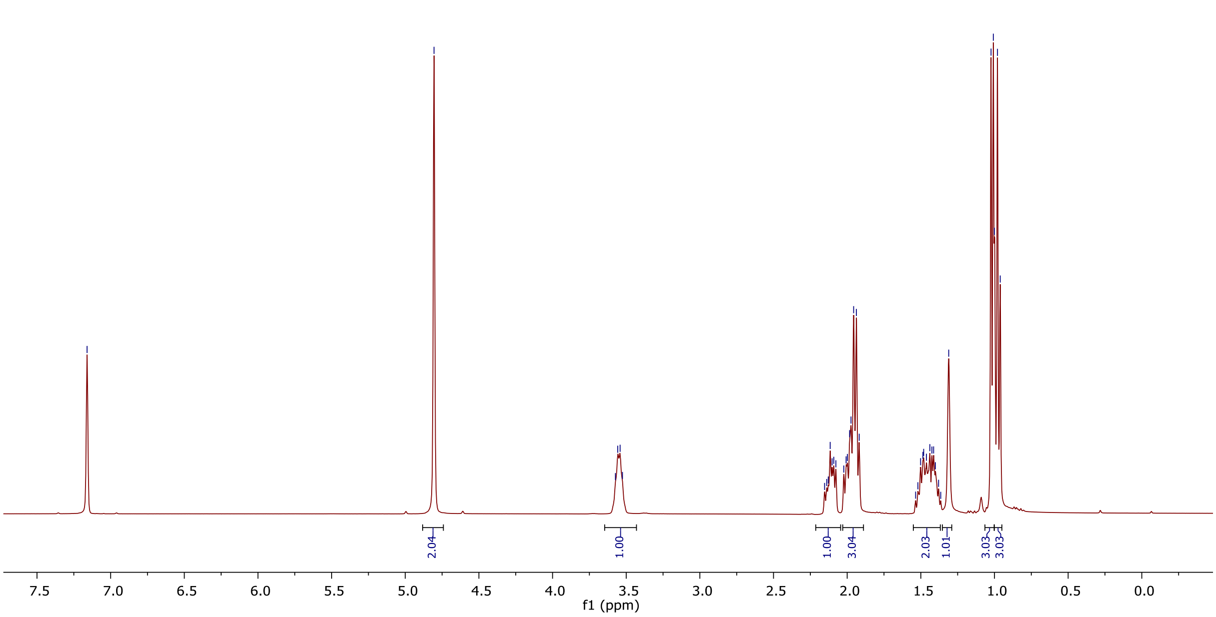

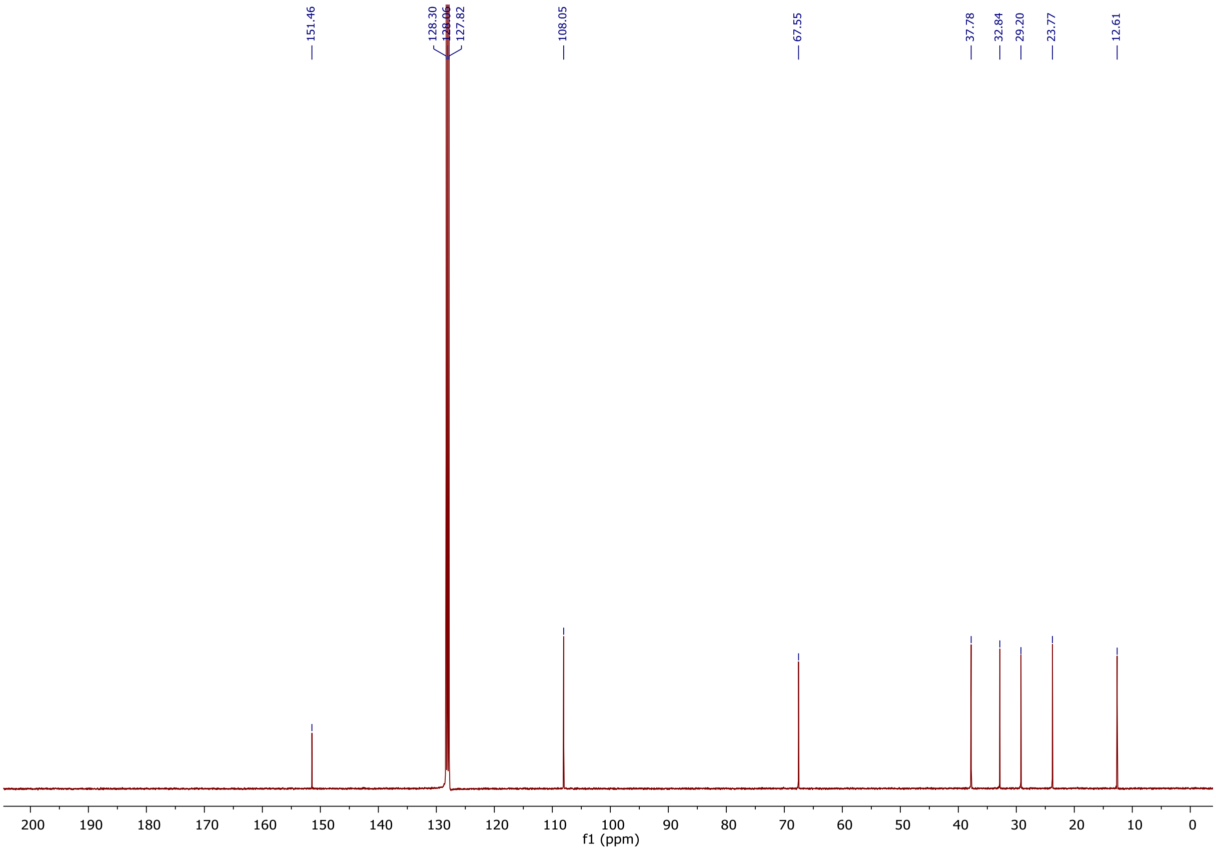

Benzyl (5-methyleneheptan-2-yl)((perfluorobenzoyl)oxy)carbamate (1i)

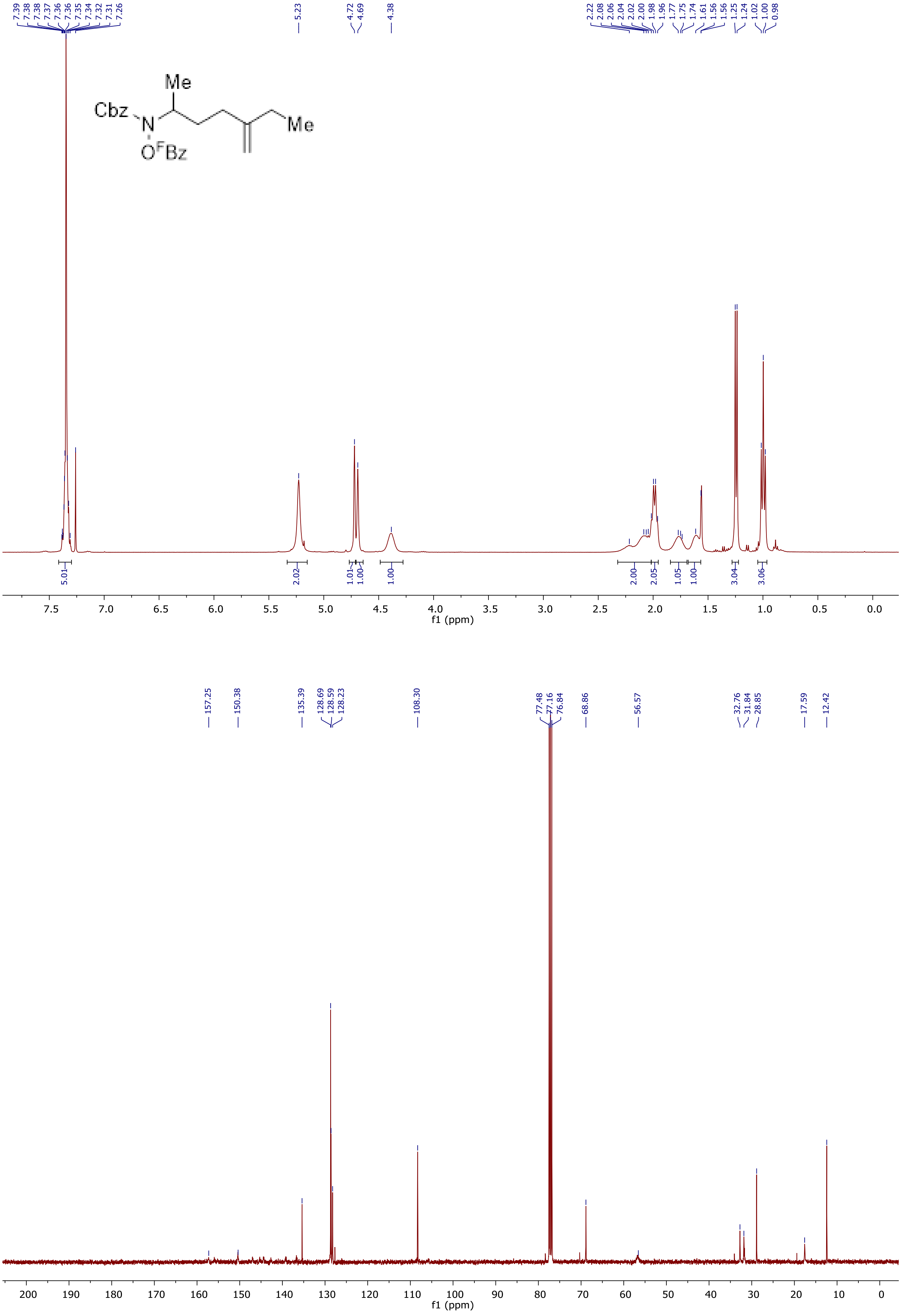

Benzyl 1-ethyl-3-methyl-2-azabicyclo[3.1.0]hexane-2-carboxylate (2i)

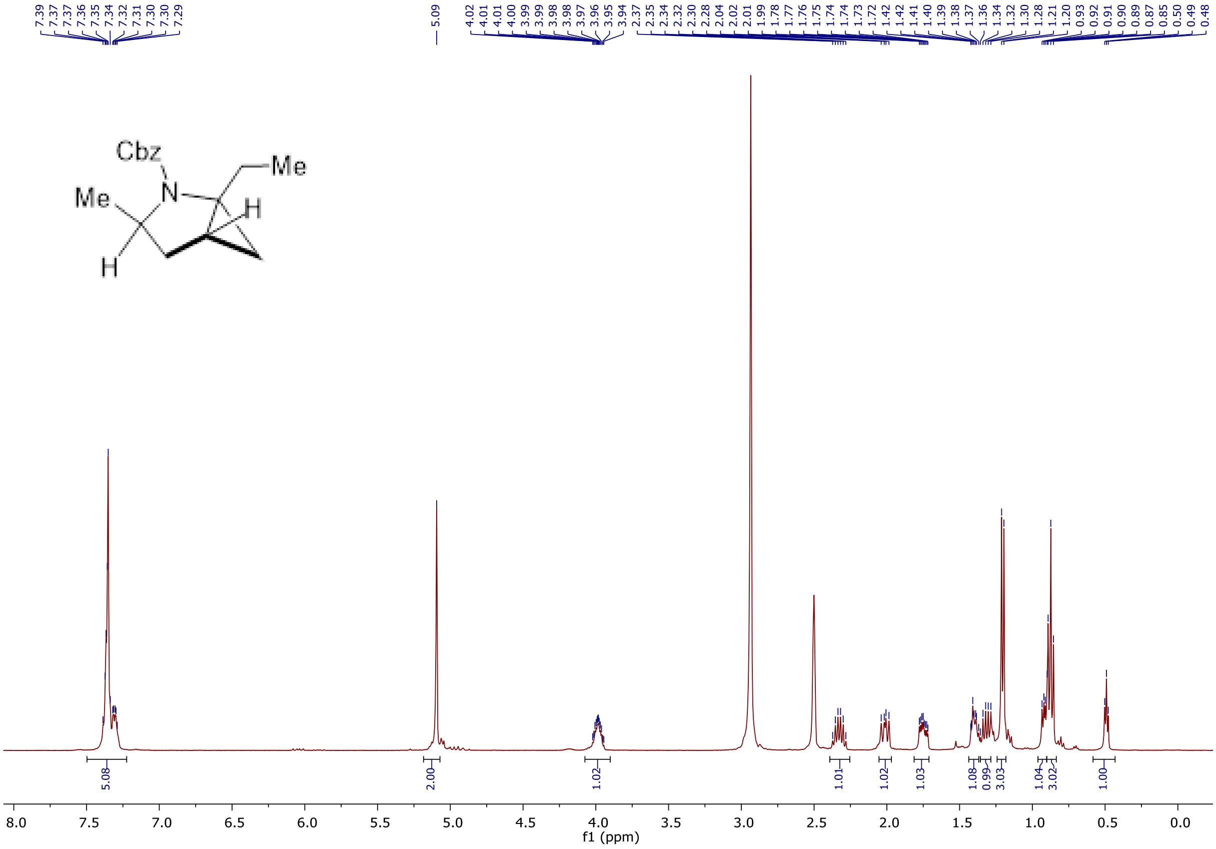

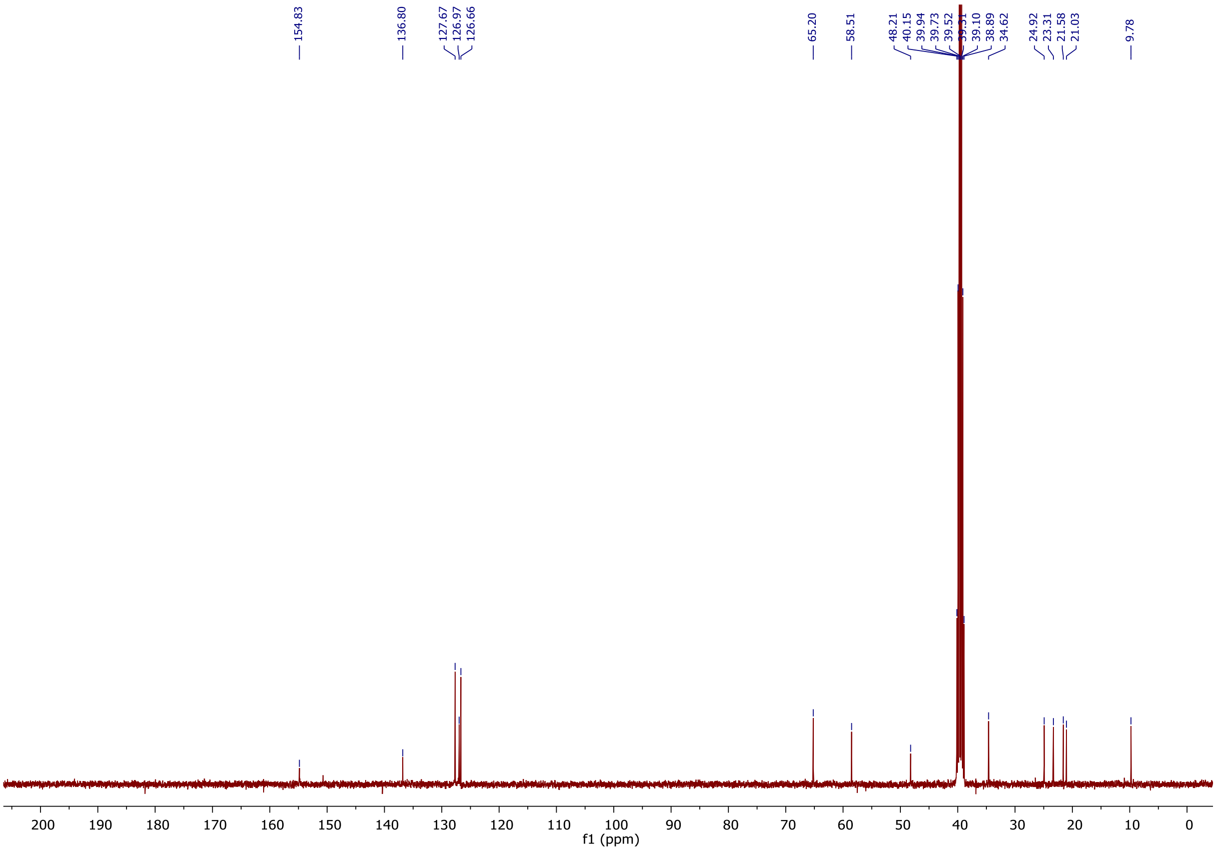

2-((2,4-Dinitrophenyl)sulfonyl)-1-ethyl-3-methyl-2-azabicyclo[3.1.0]hexane (2i')

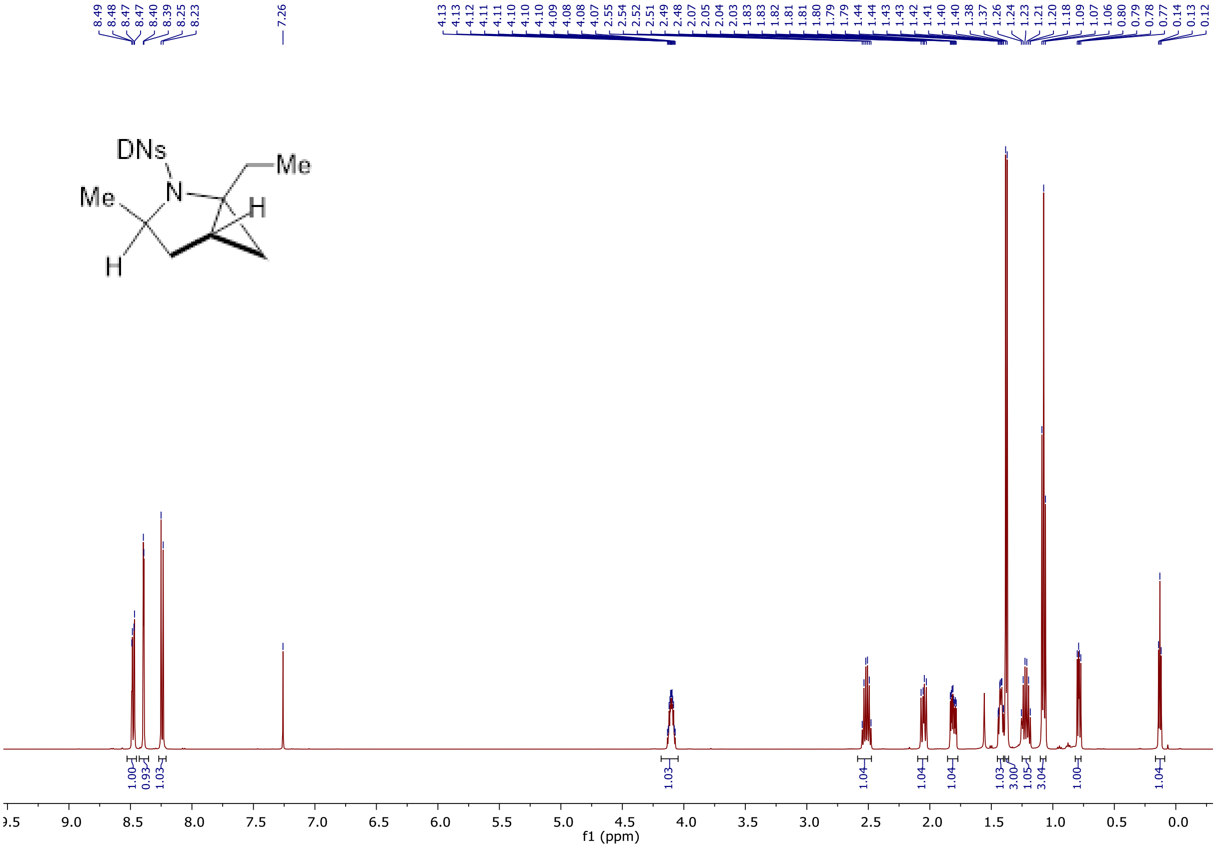

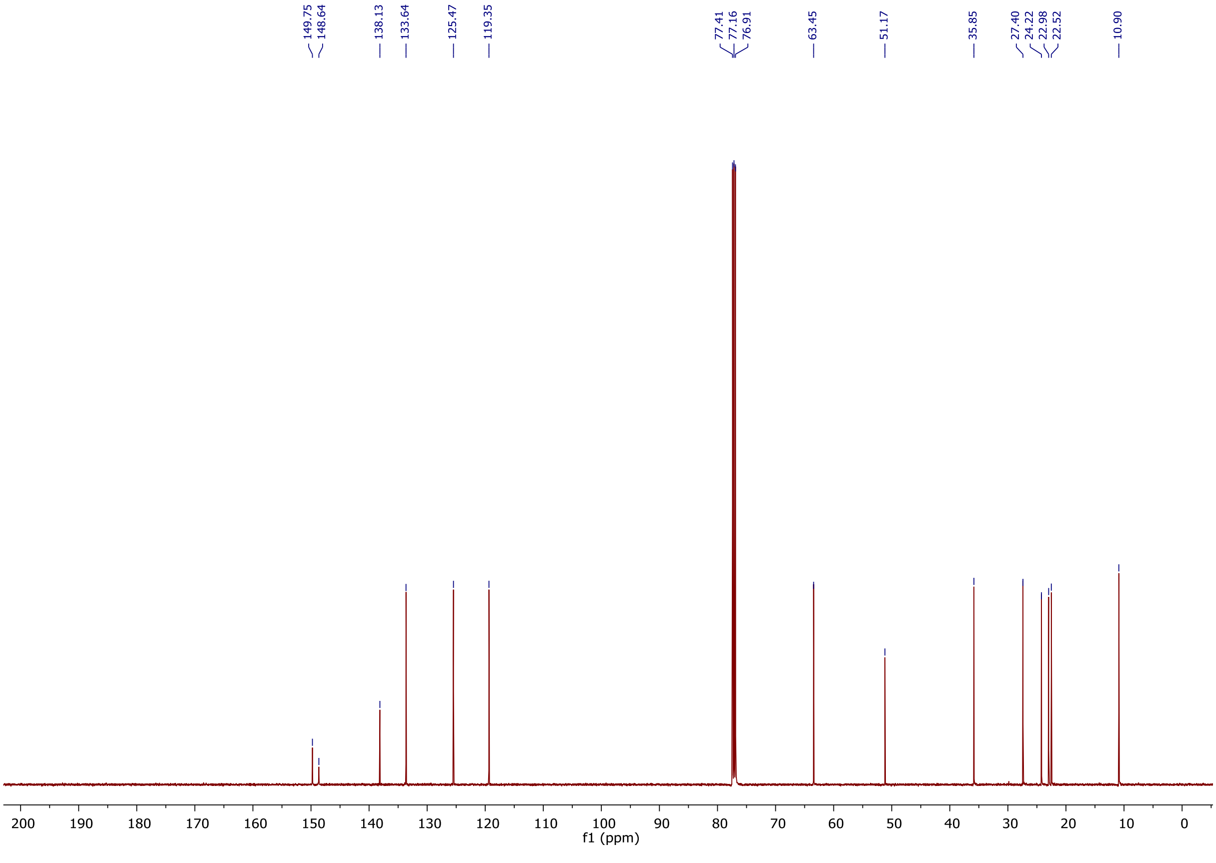

Benzyl (4-methylpent-4-en-1-yl)((perfluorobenzoyl)oxy)carbamate (1j)

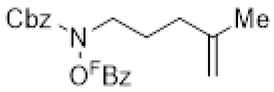

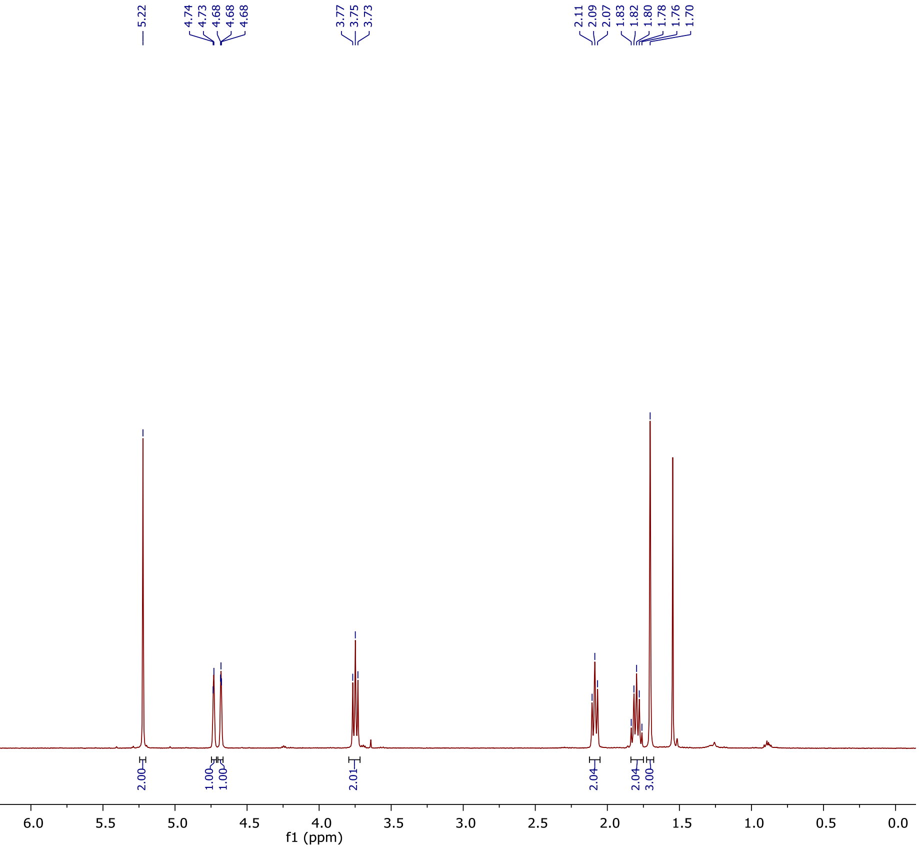

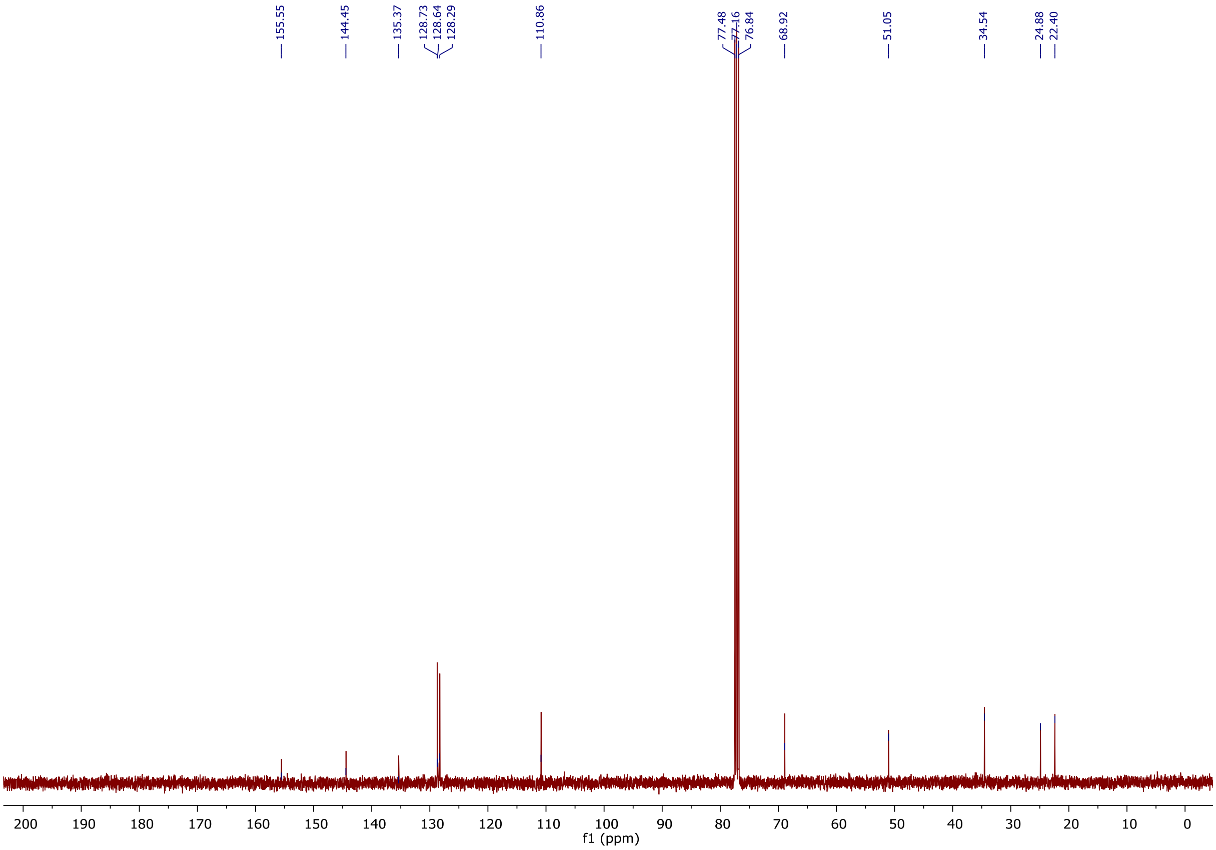

Benzyl 1-methyl-2-azabicyclo[3.1.0]hexane-2-carboxylate (2j)

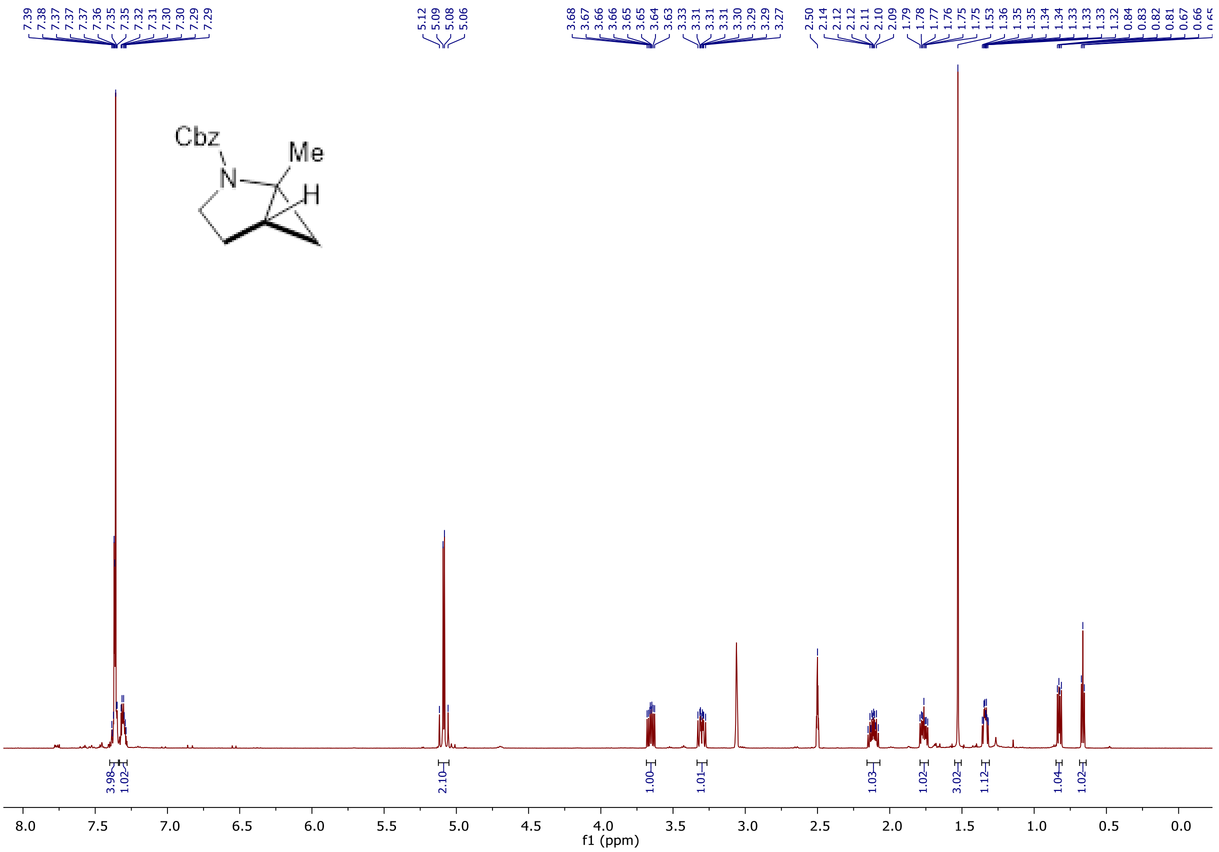

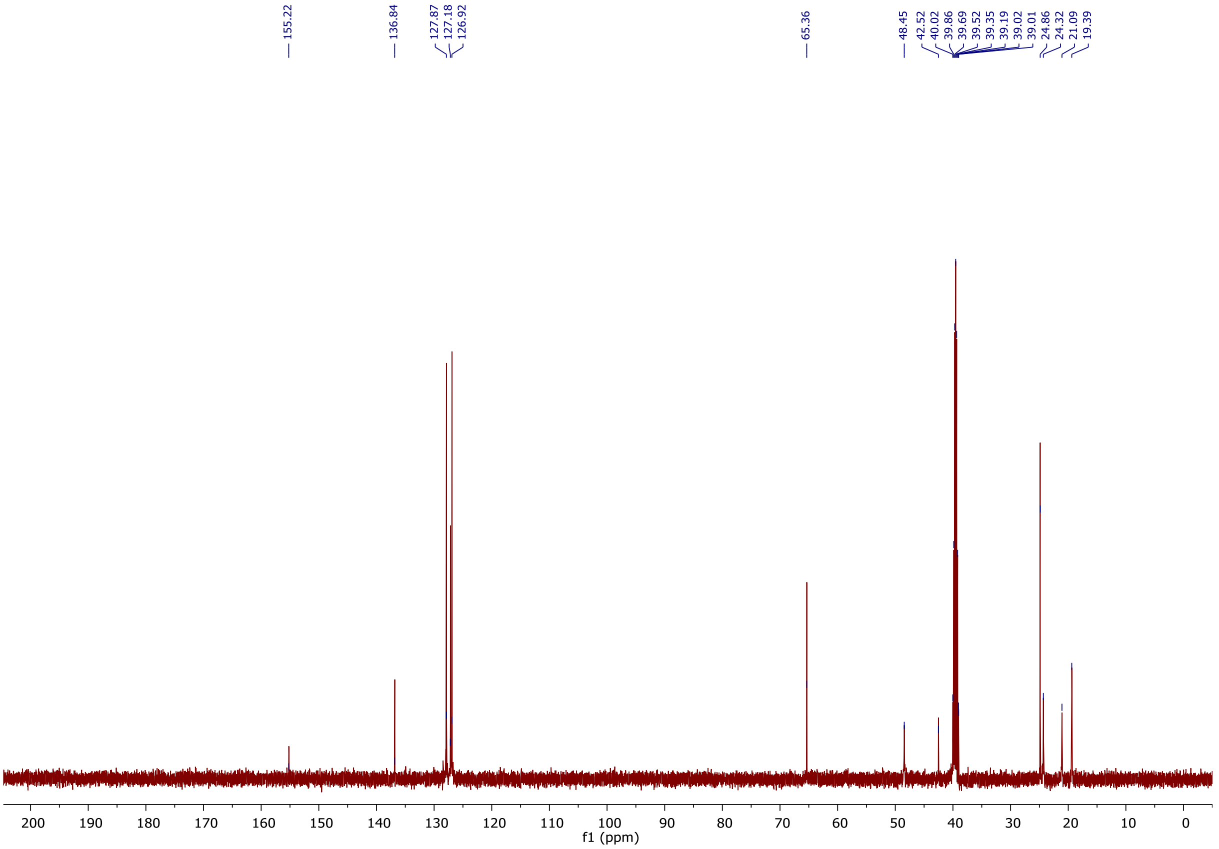

Benzyl 4-azaspiro[2.4]heptane-4-carboxylate (*iso*-2j)

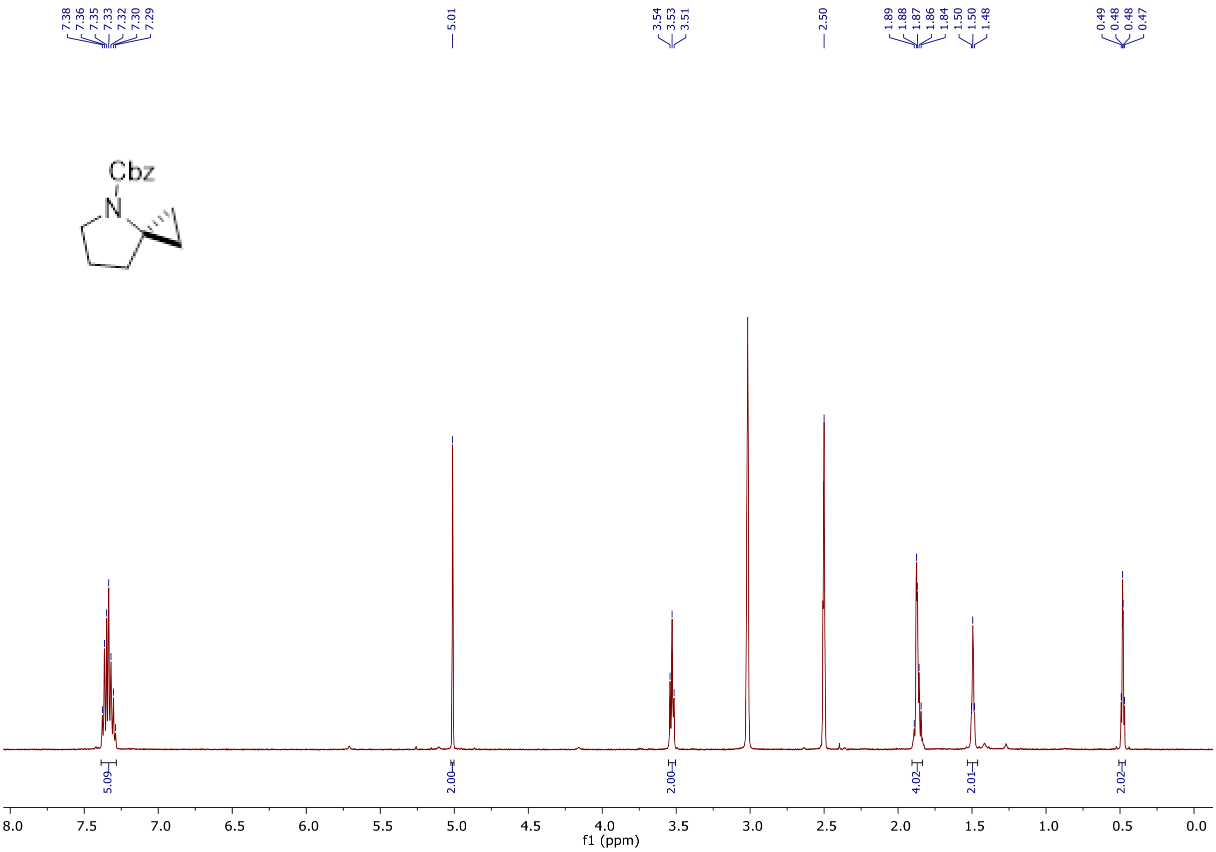

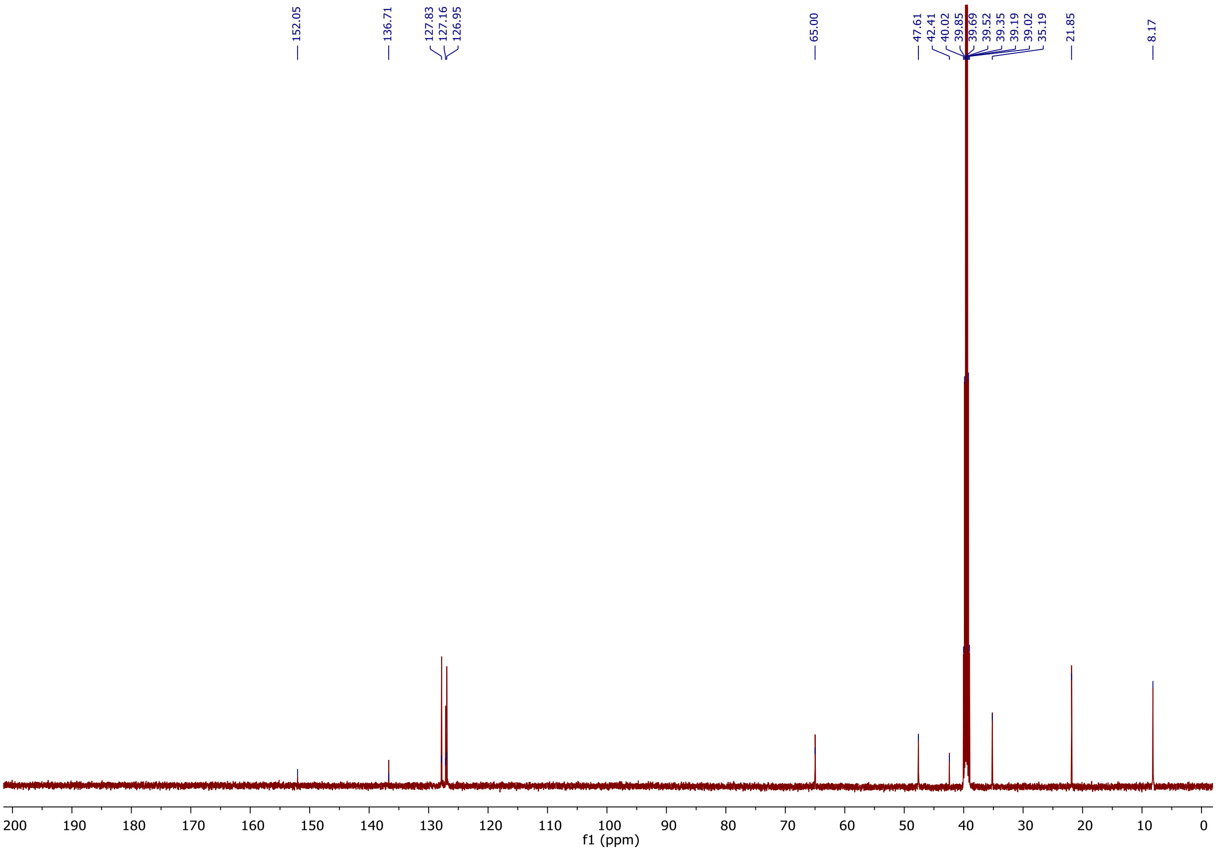

3-Ethyl-4-methylpent-4-enoic acid

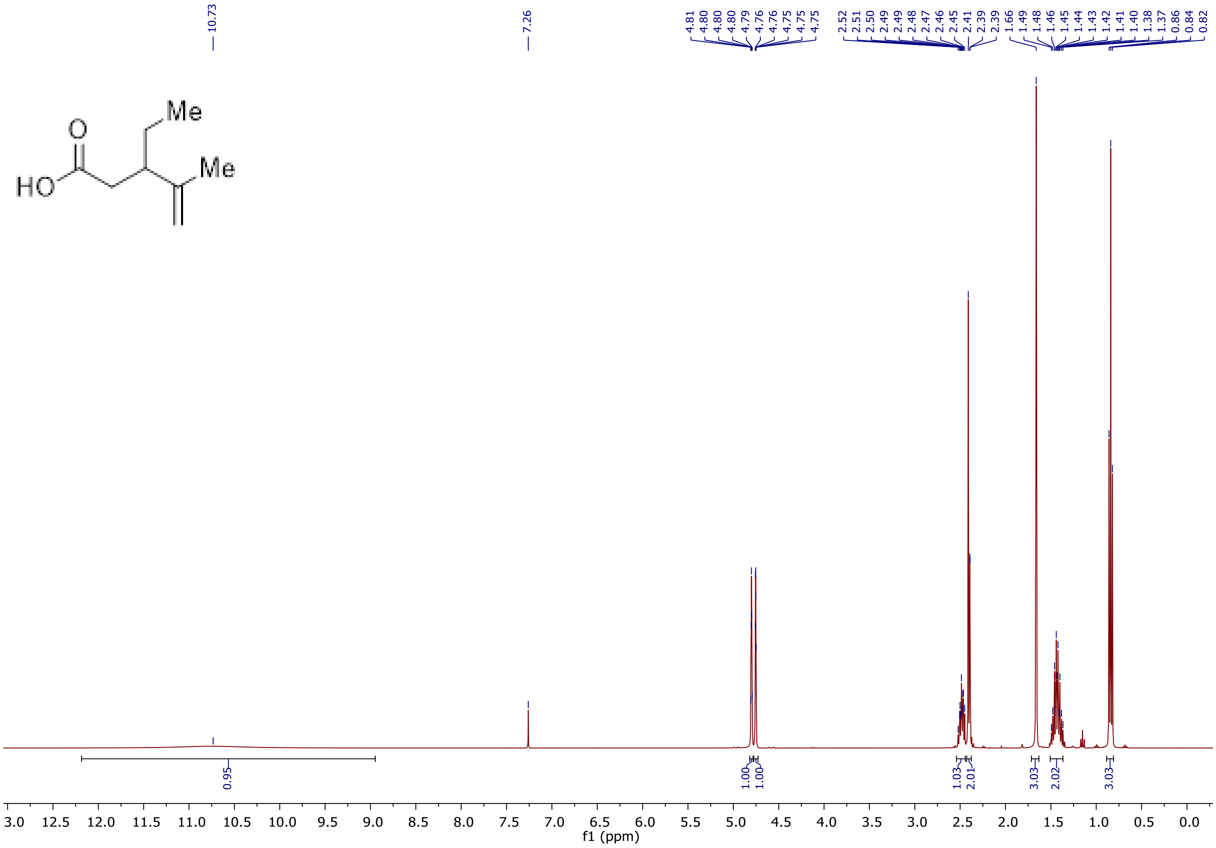

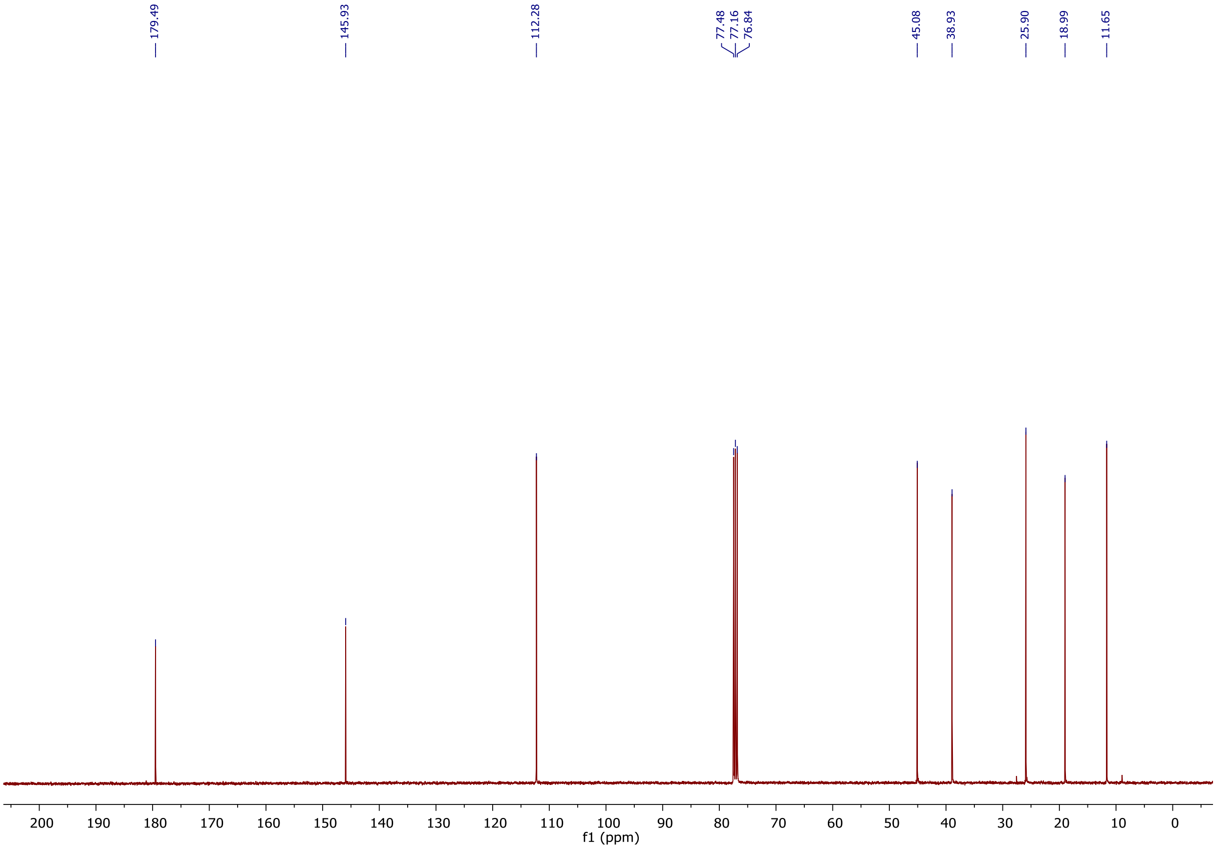

3-Ethyl-4-methylpent-4-en-1-ol

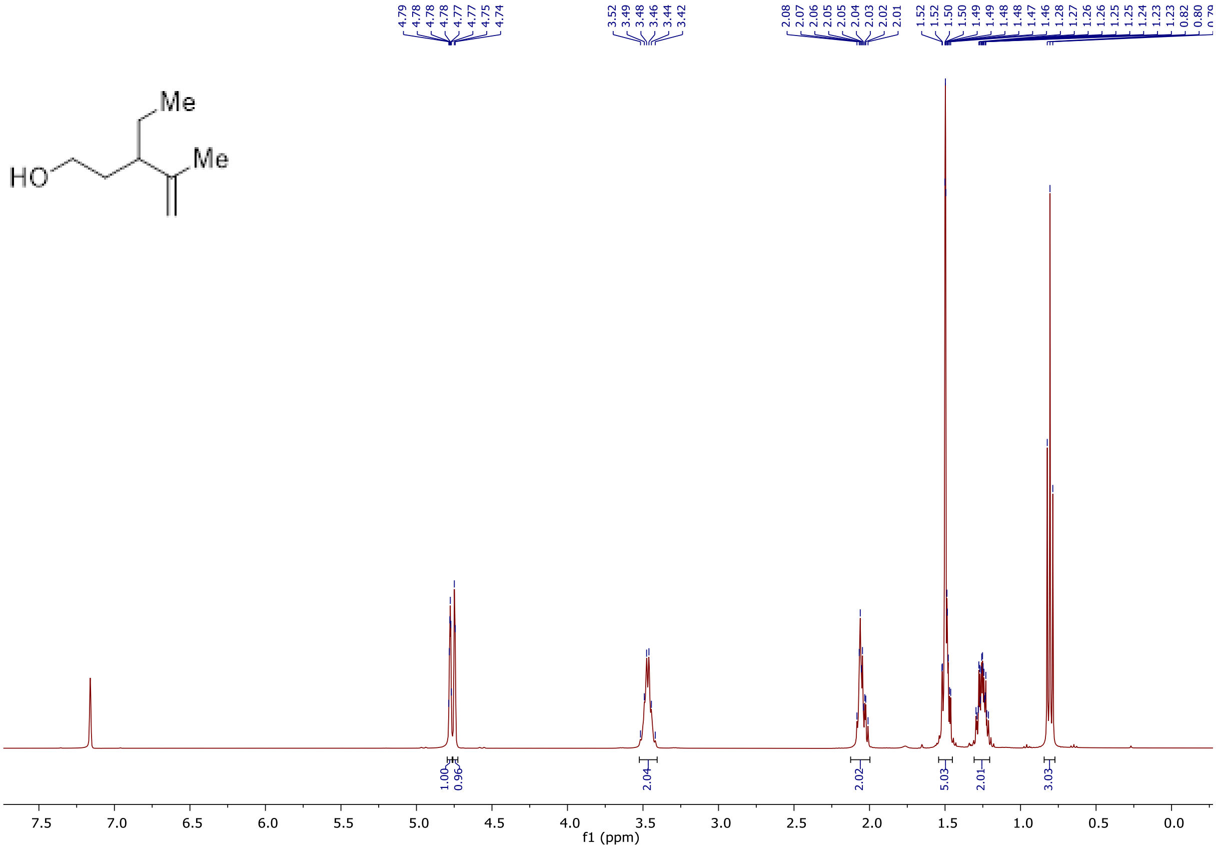

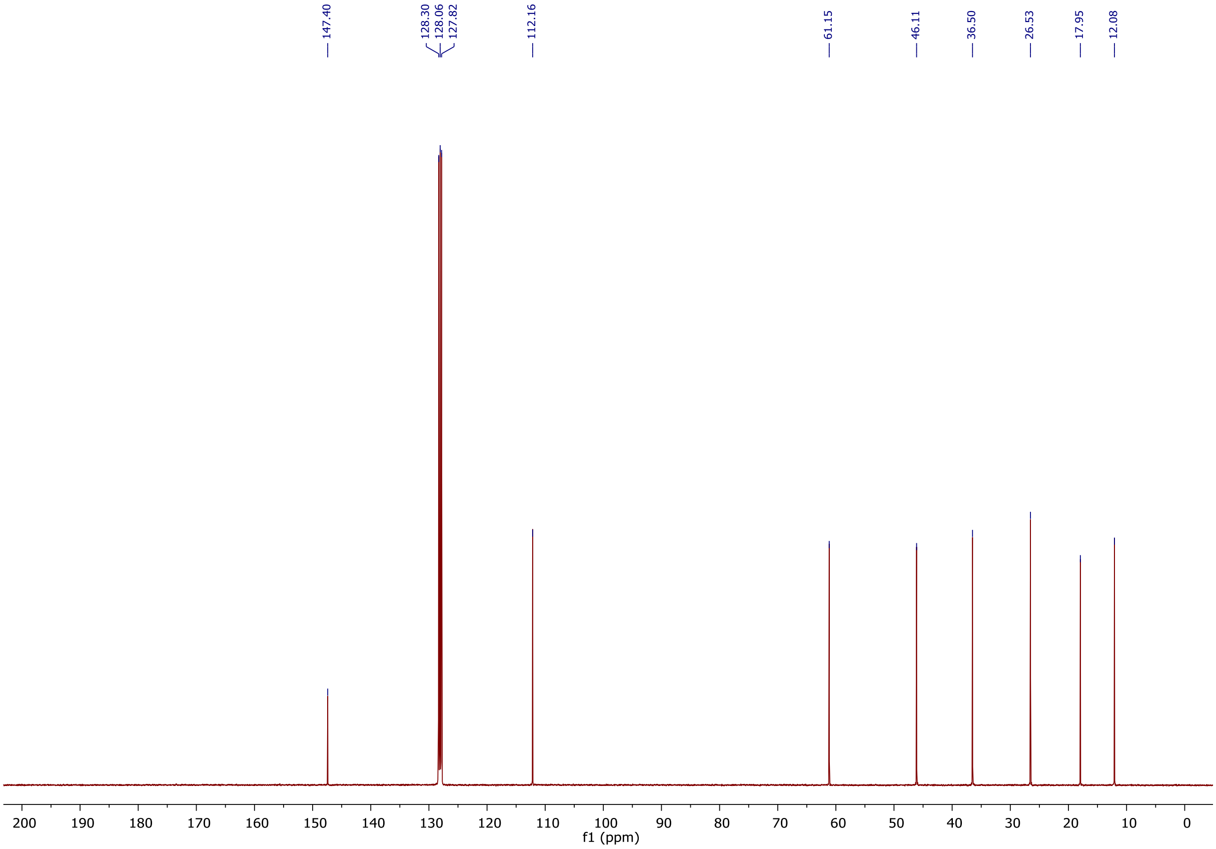

Benzyl (3-ethyl-4-methylpent-4-en-1-yl)((perfluorobenzoyl)oxy)carbamate (1k)

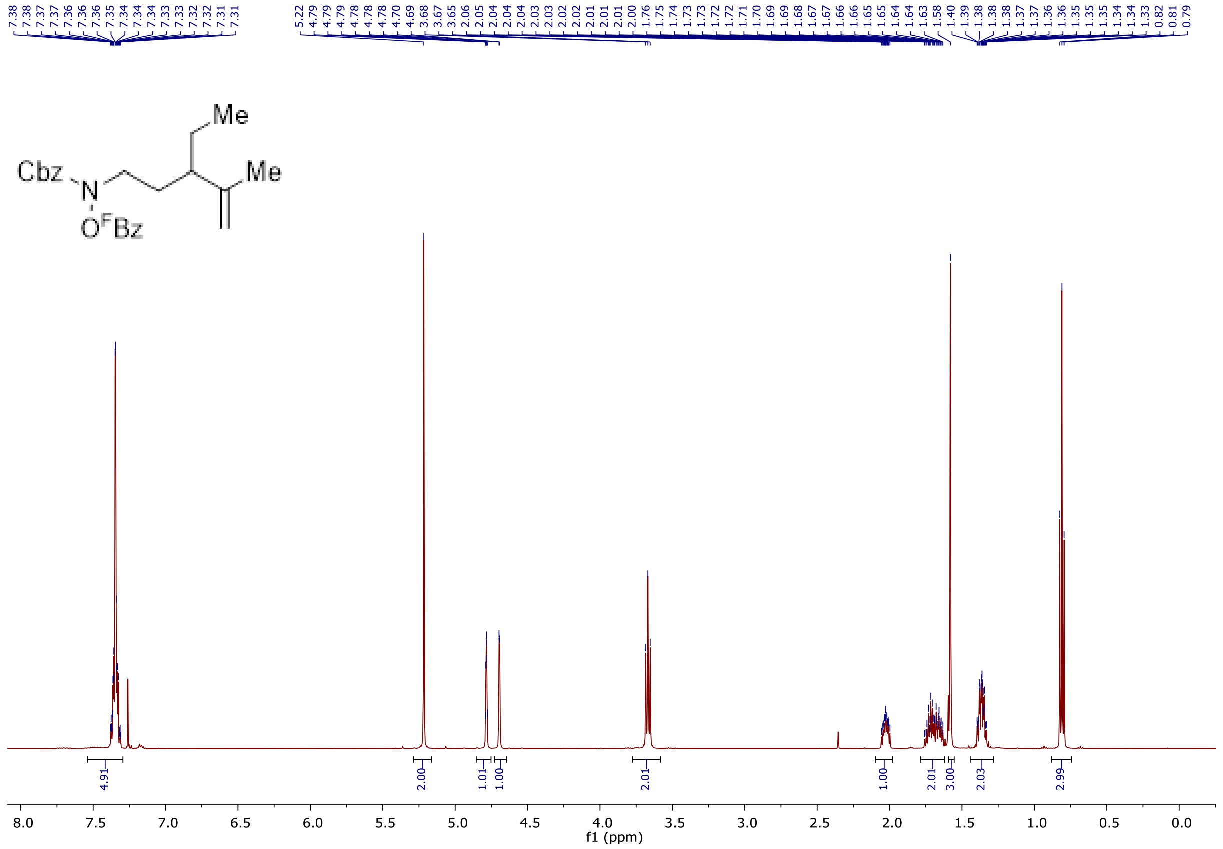

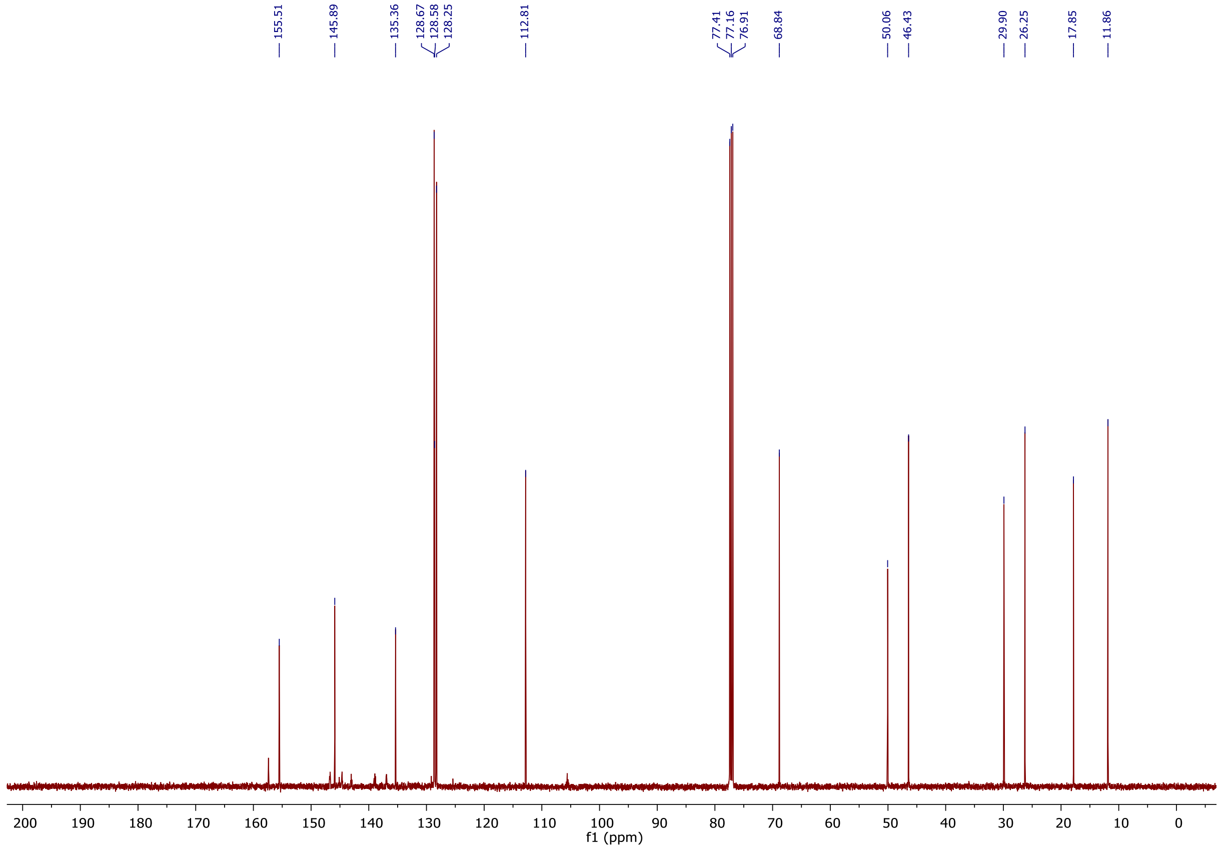

Benzyl 7-ethyl-4-azaspiro[2.4]heptane-4-carboxylate (*iso*-2k)

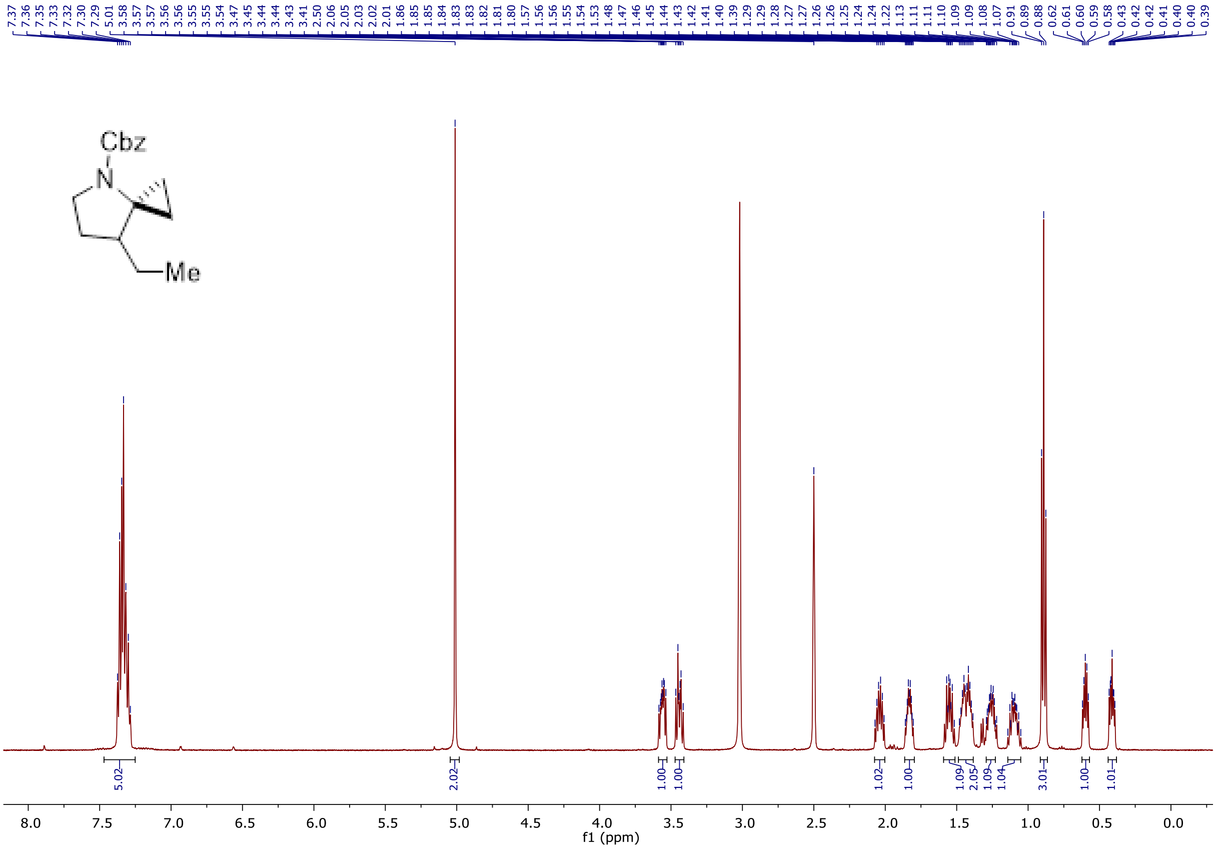

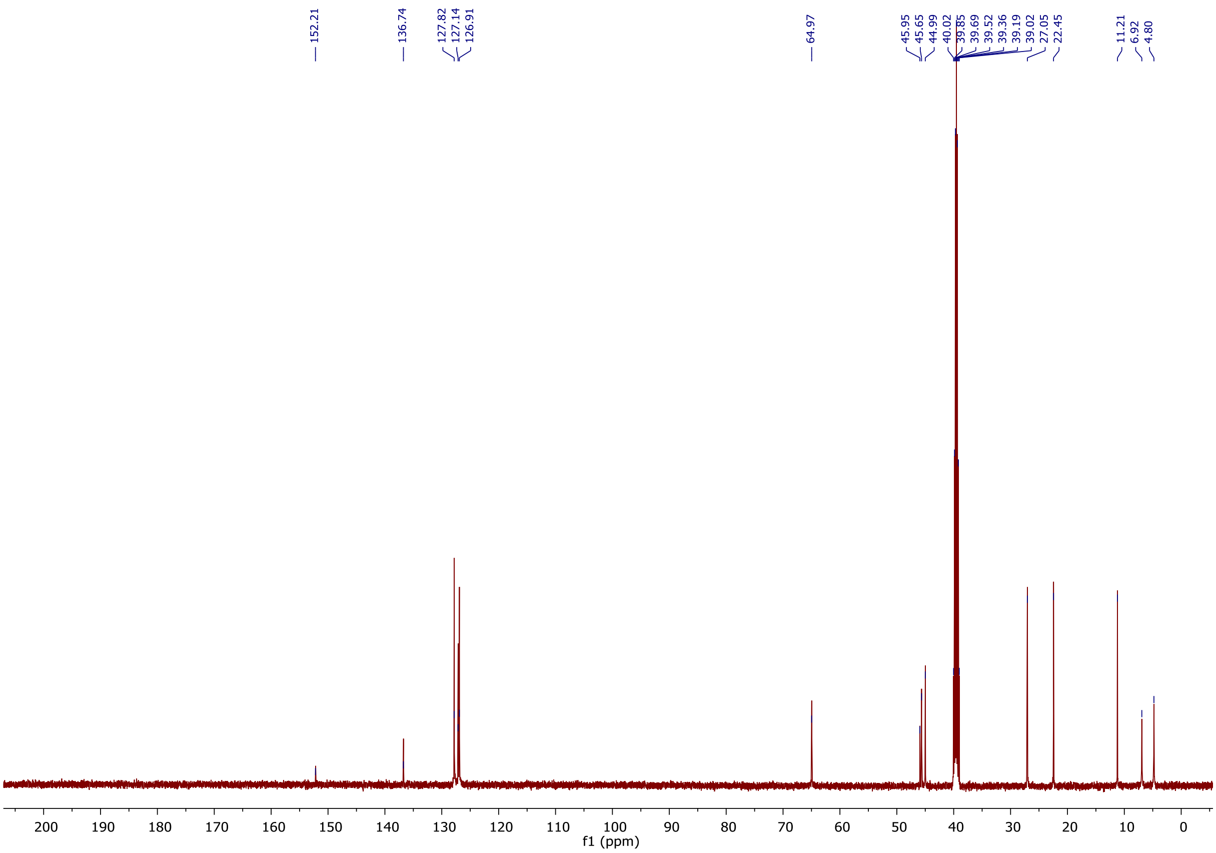

3-Isopropyl-4-methylpent-4-en-1-ol

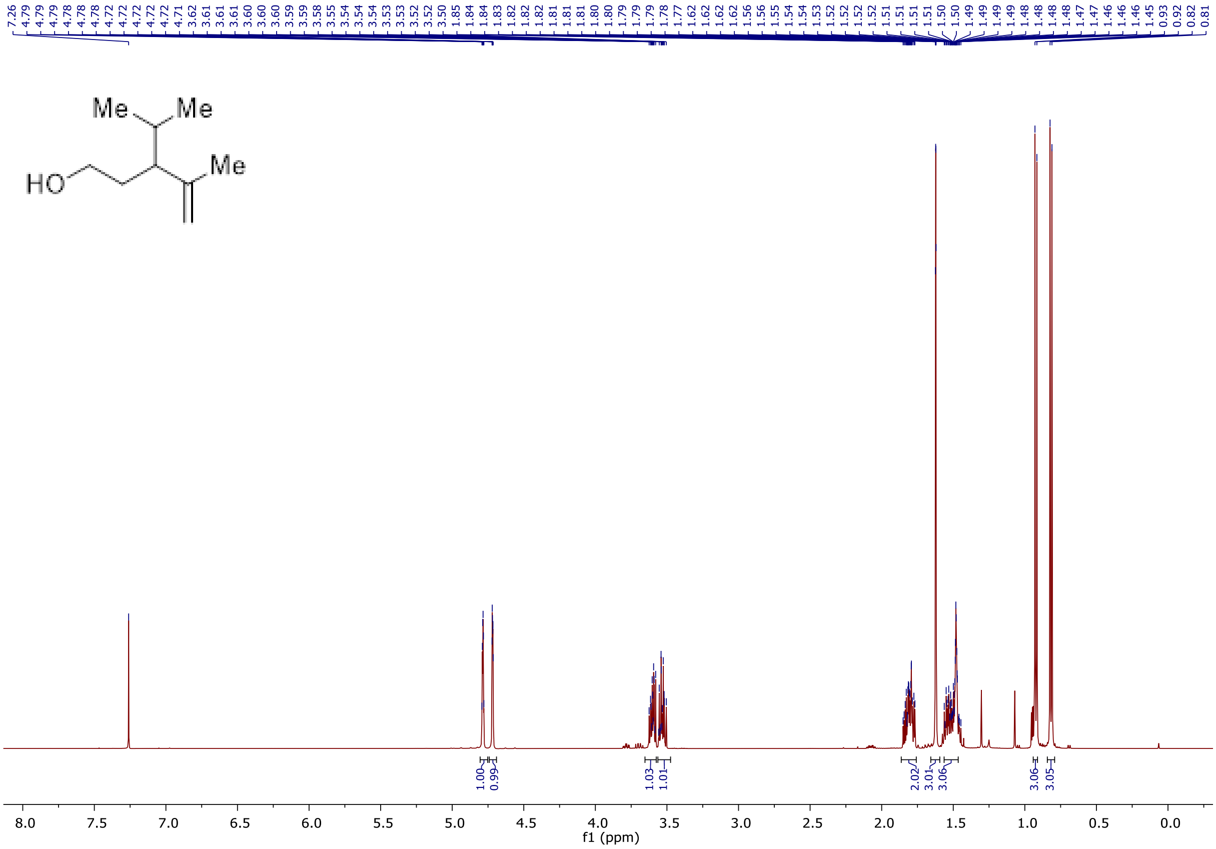

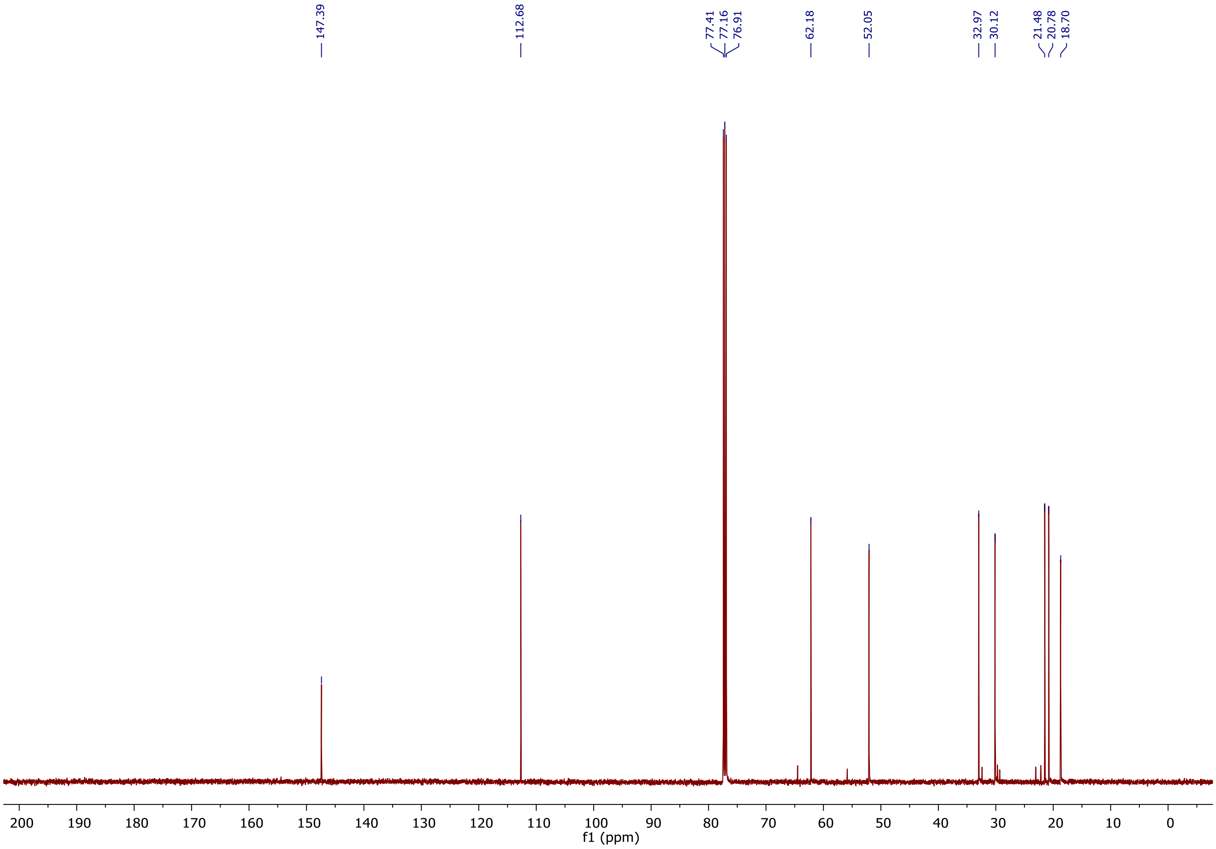

Benzyl (3-isopropyl-4-methylpent-4-en-1-yl)((perfluorobenzoyl)oxy)carbamate (2l)

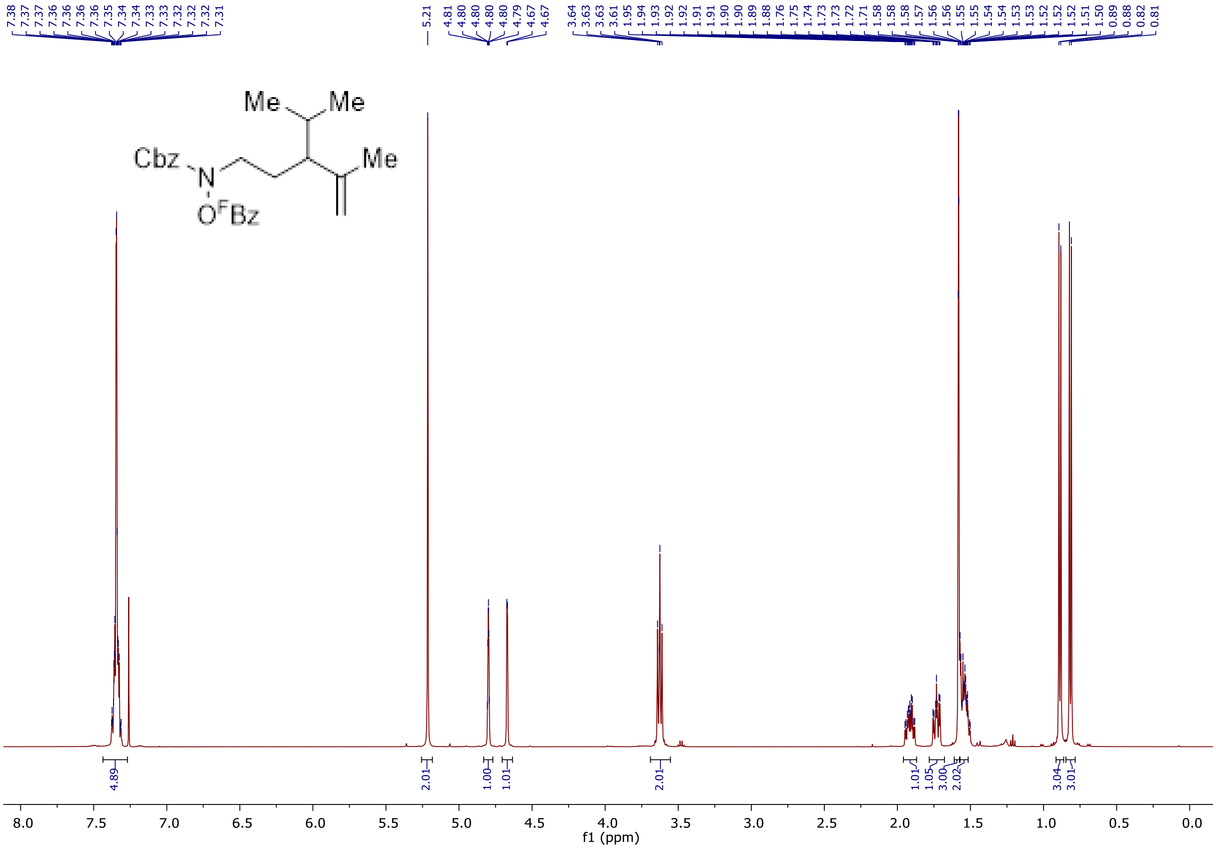

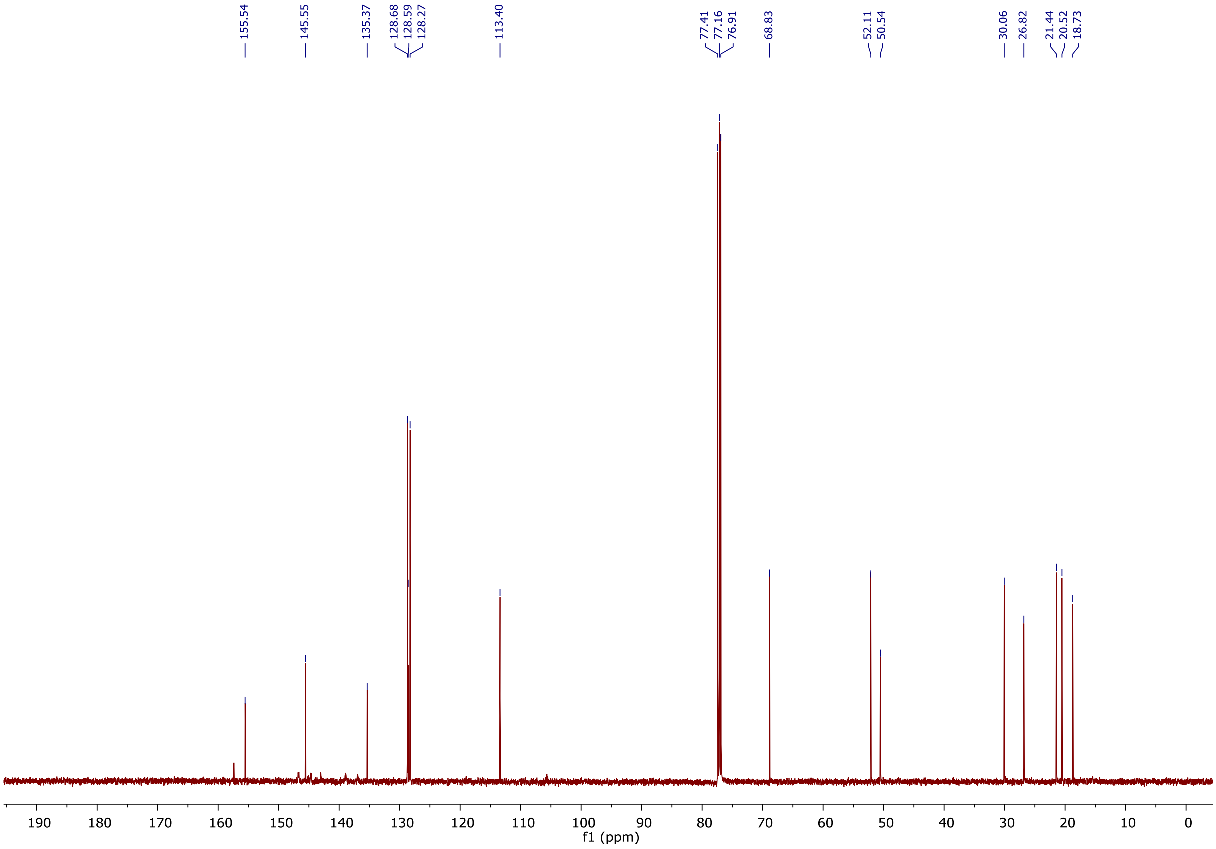

Benzyl 7-isopropyl-4-azaspiro[2.4]heptane-4-carboxylate (*iso*-2l)

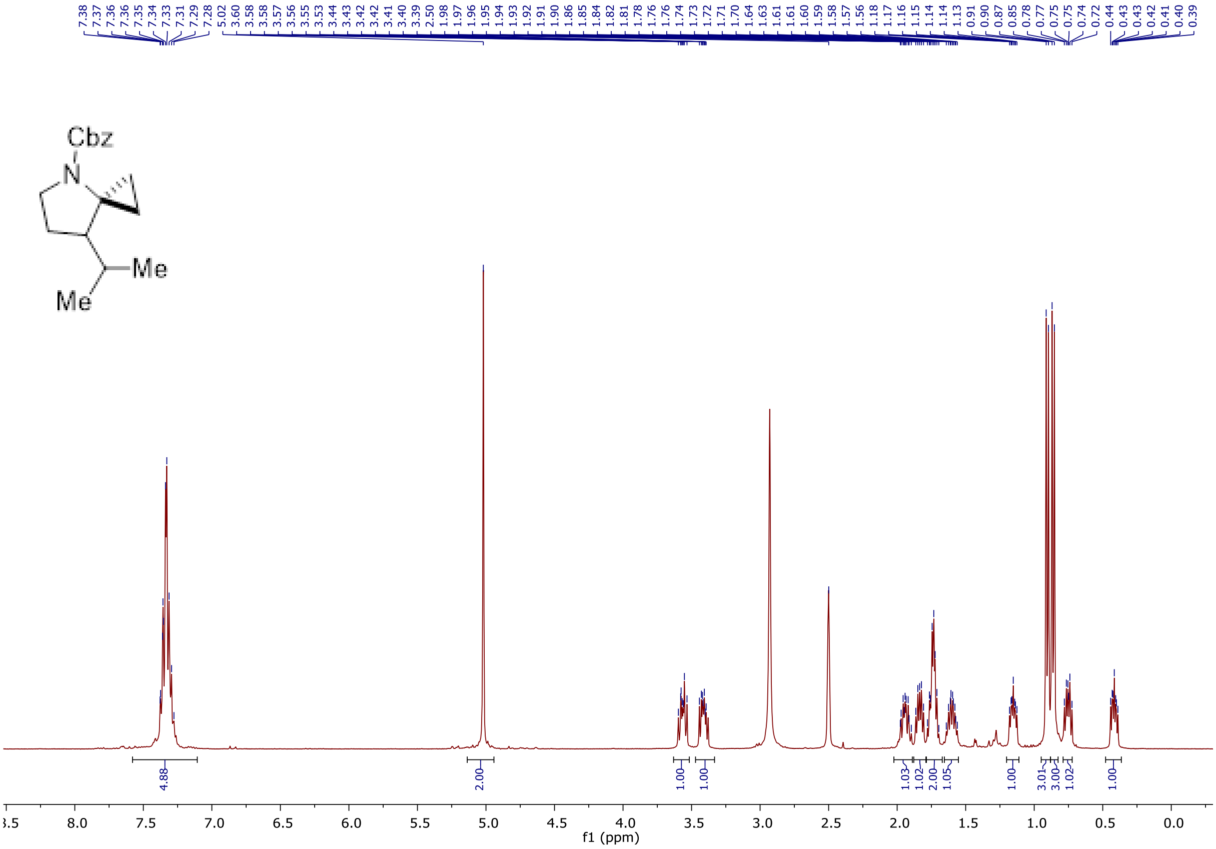

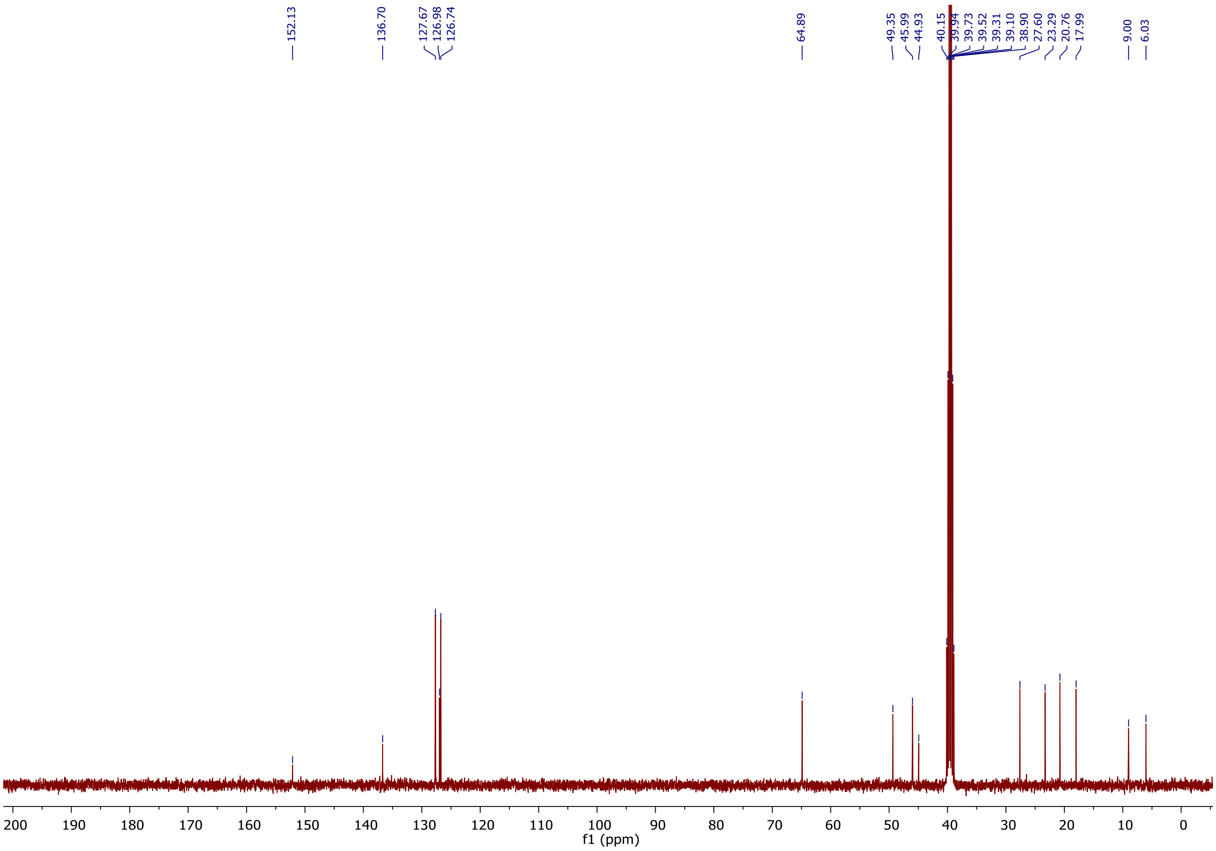

2-(1-(Prop-1-en-2-yl)cyclopentyl)acetic acid

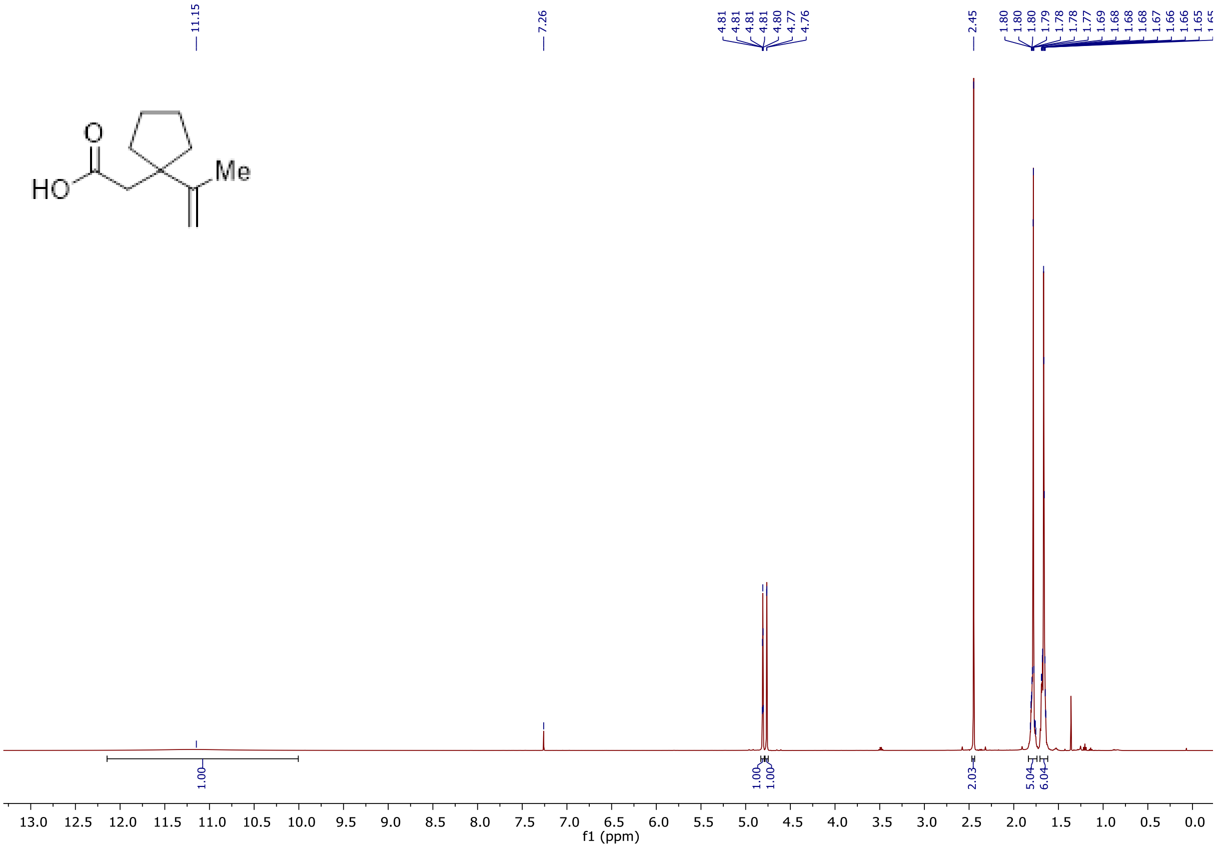

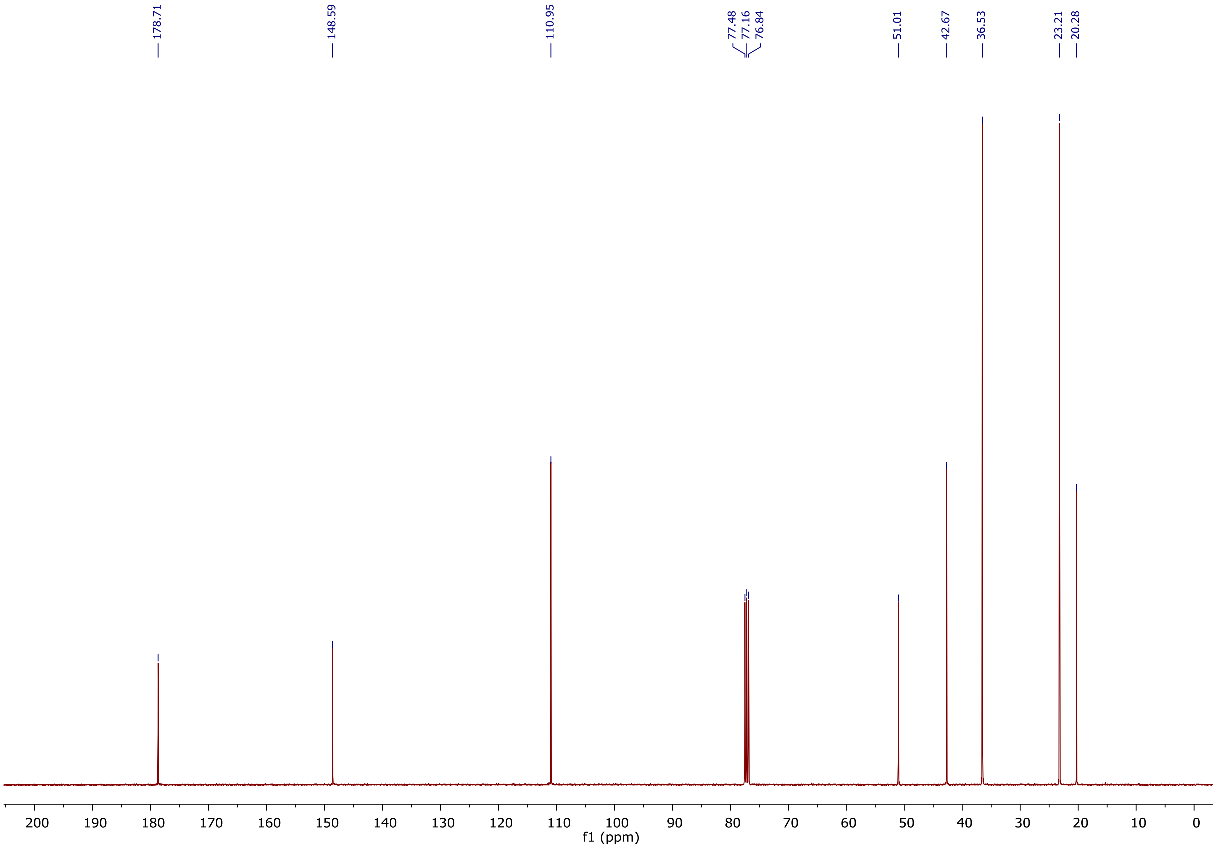

2-(1-(Prop-1-en-2-yl)cyclopentyl)ethan-1-ol

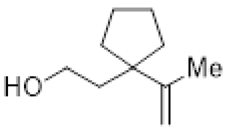

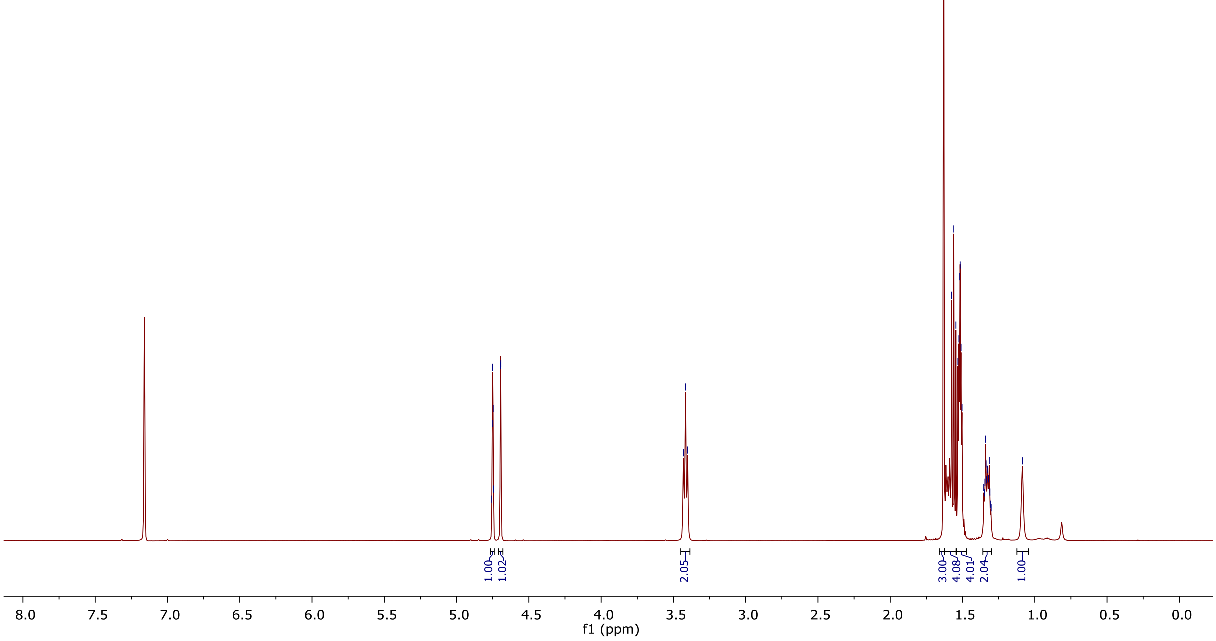

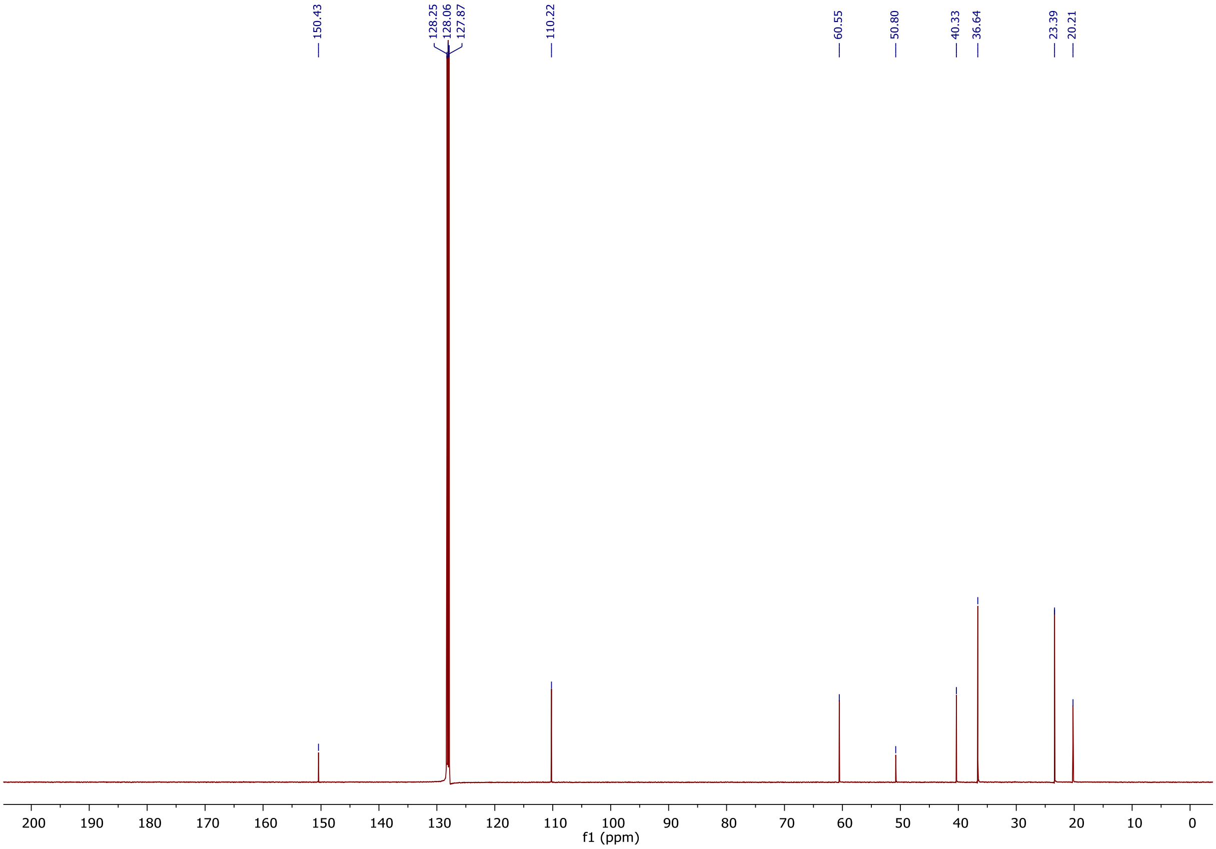

Benzyl ((perfluorobenzoyl)oxy)(2-(1-(prop-1-en-2-yl)cyclopentyl)ethyl)carbamate (2m)

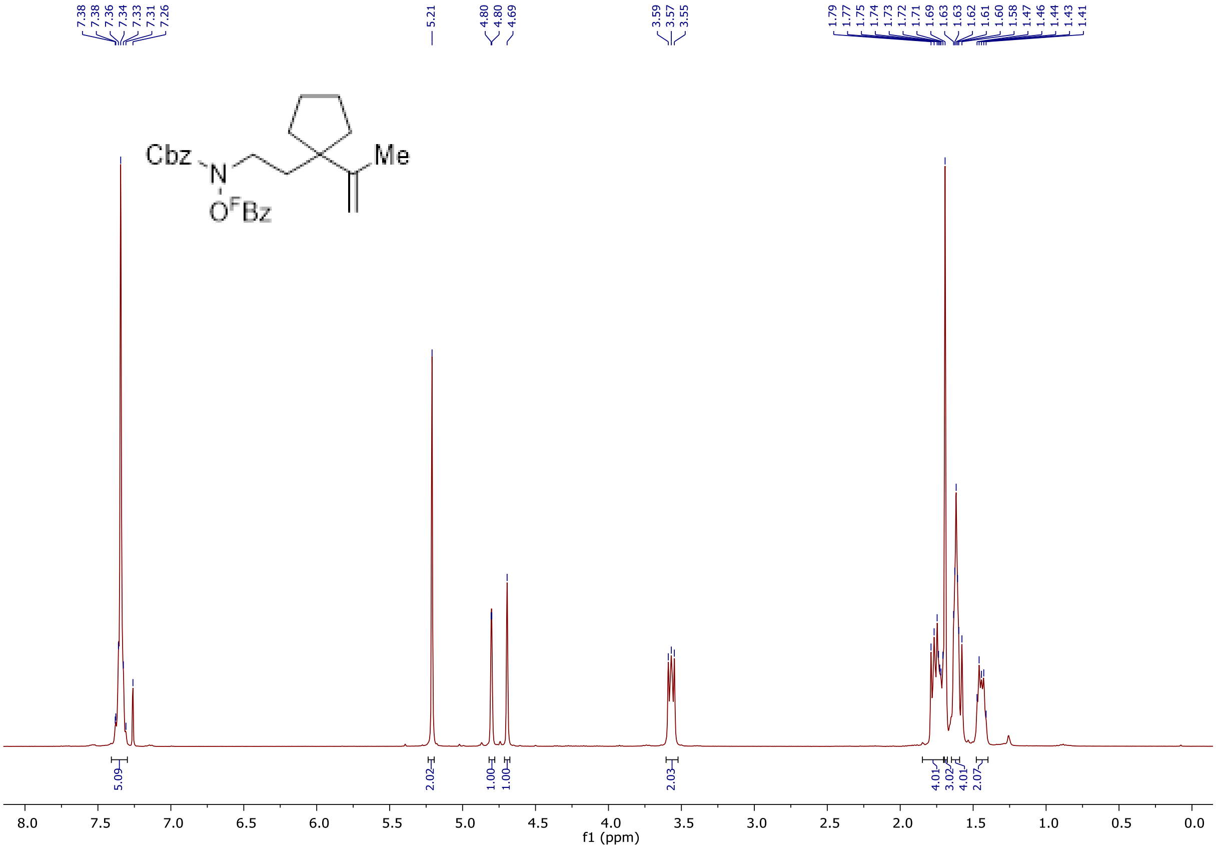

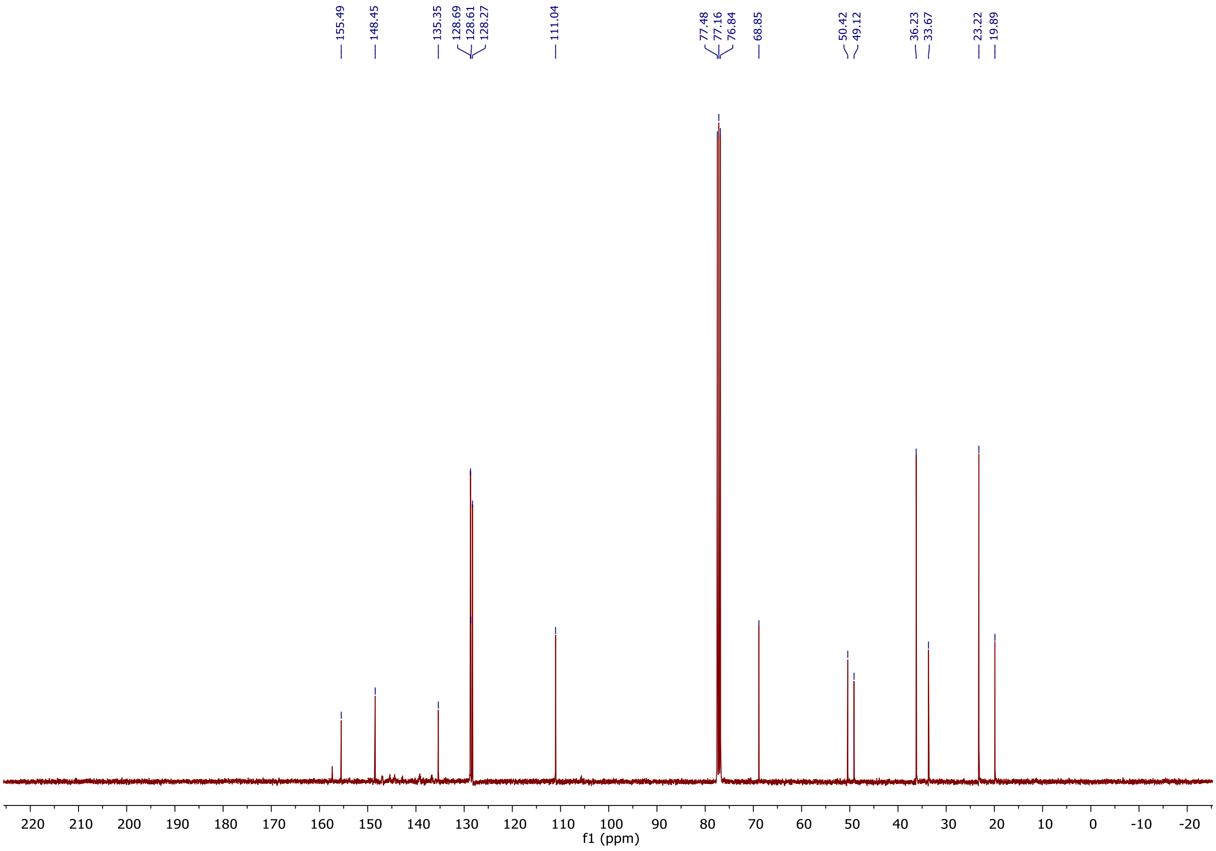

Benzyl 11-azadispiro[2.0.4^4.3^3]undecane-11-carboxylate (*iso*-2m)

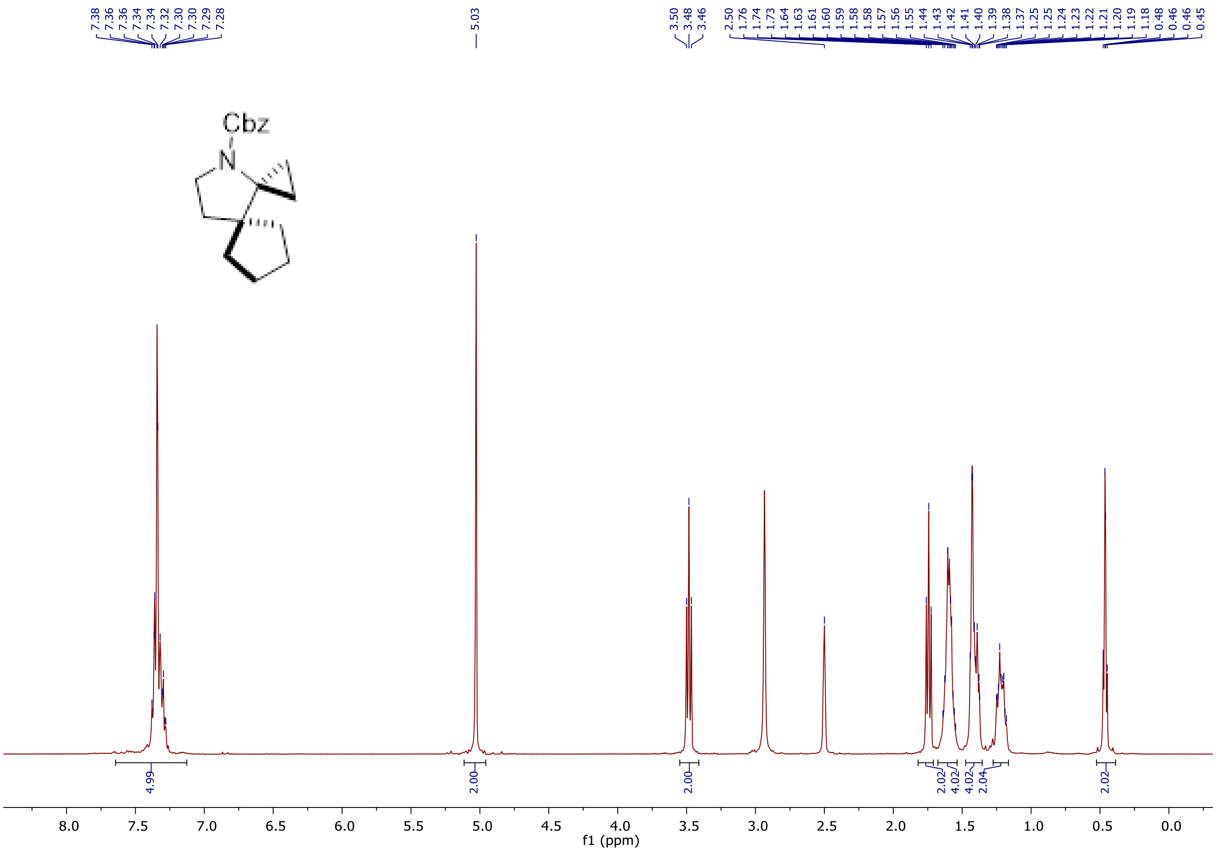

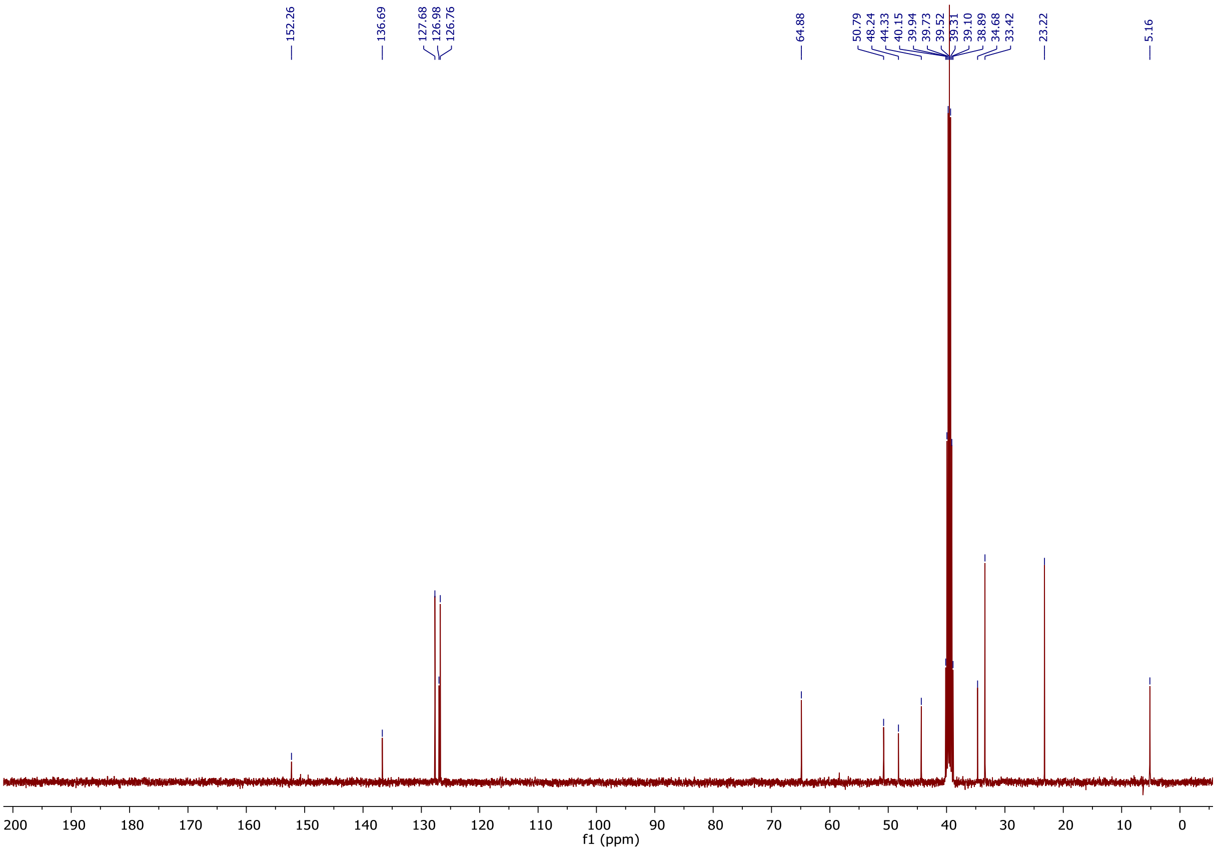

Methyl 2-(2-methylenebutyl)benzoate

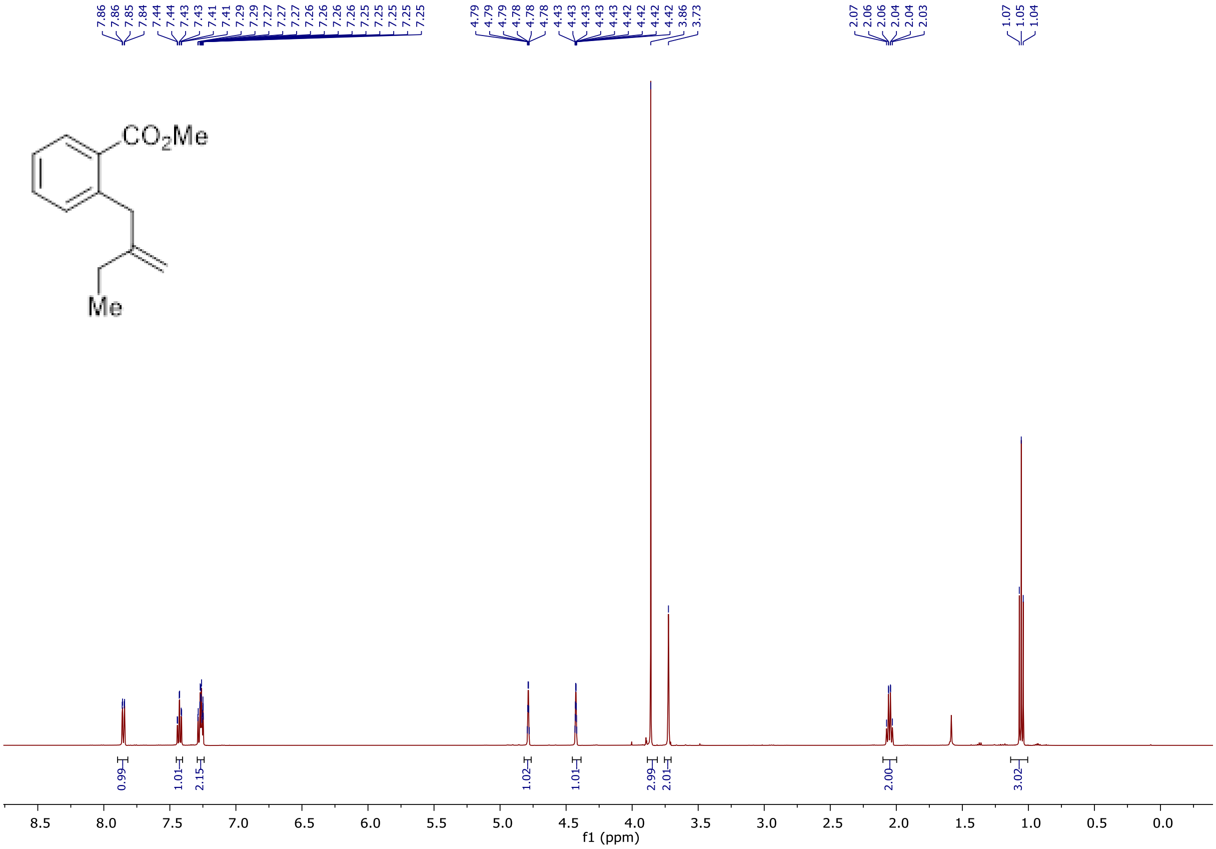

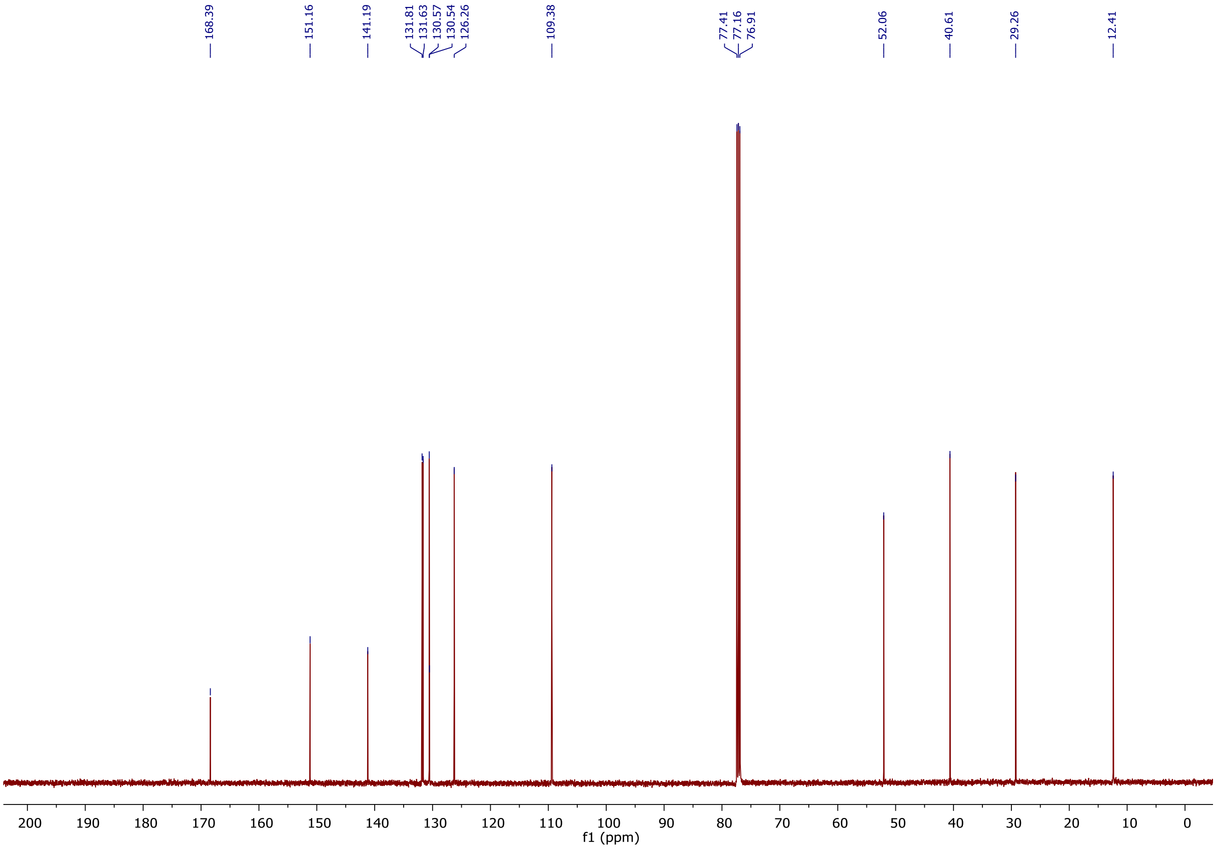

(2-(2-Methylenebutyl)phenyl)methanol

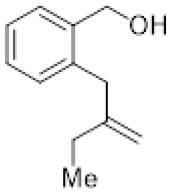

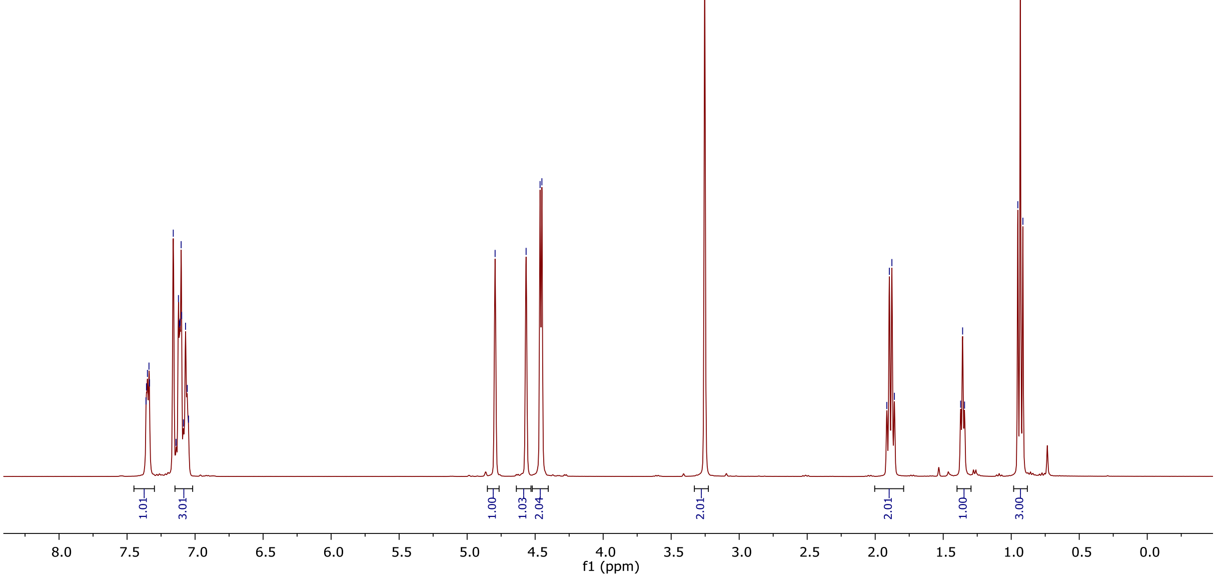

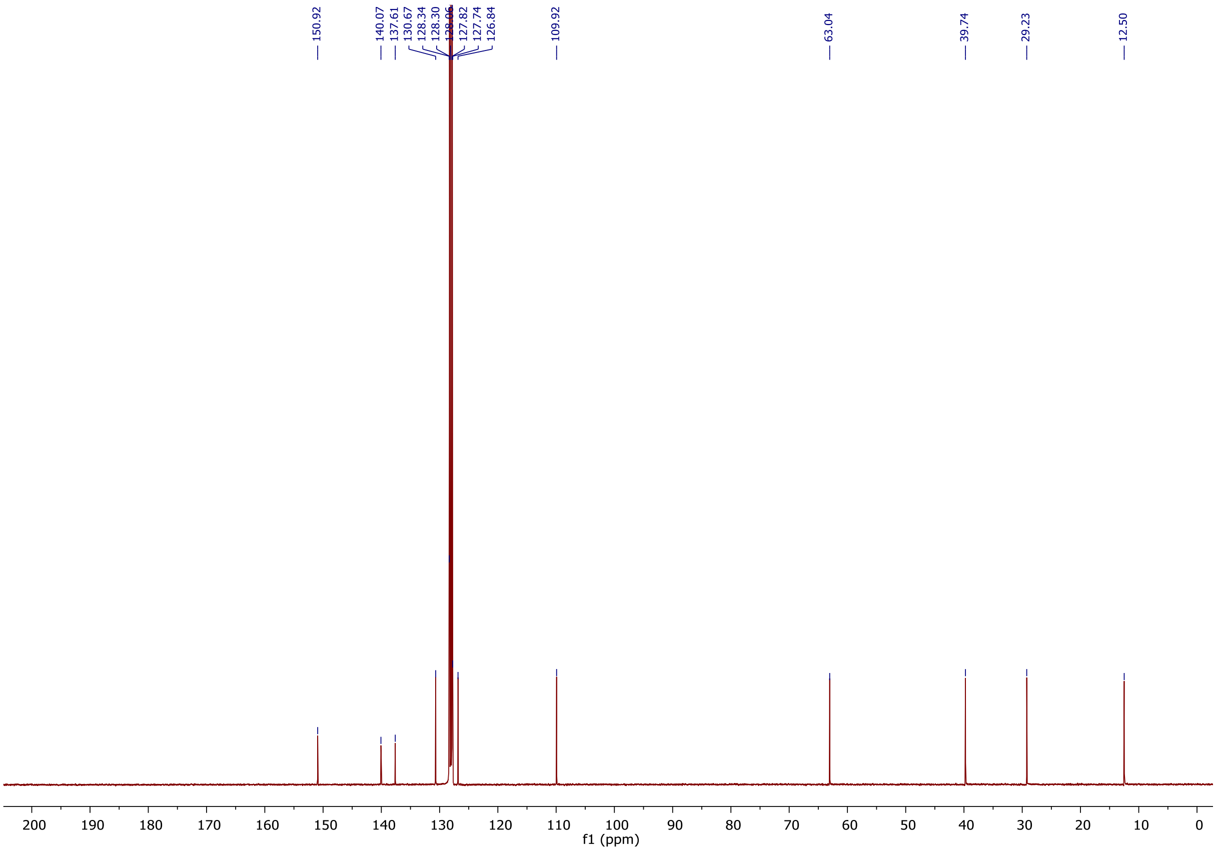

Benzyl (2-(2-methylenebutyl)benzyl)((perfluorobenzoyl)oxy)carbamate (1n)

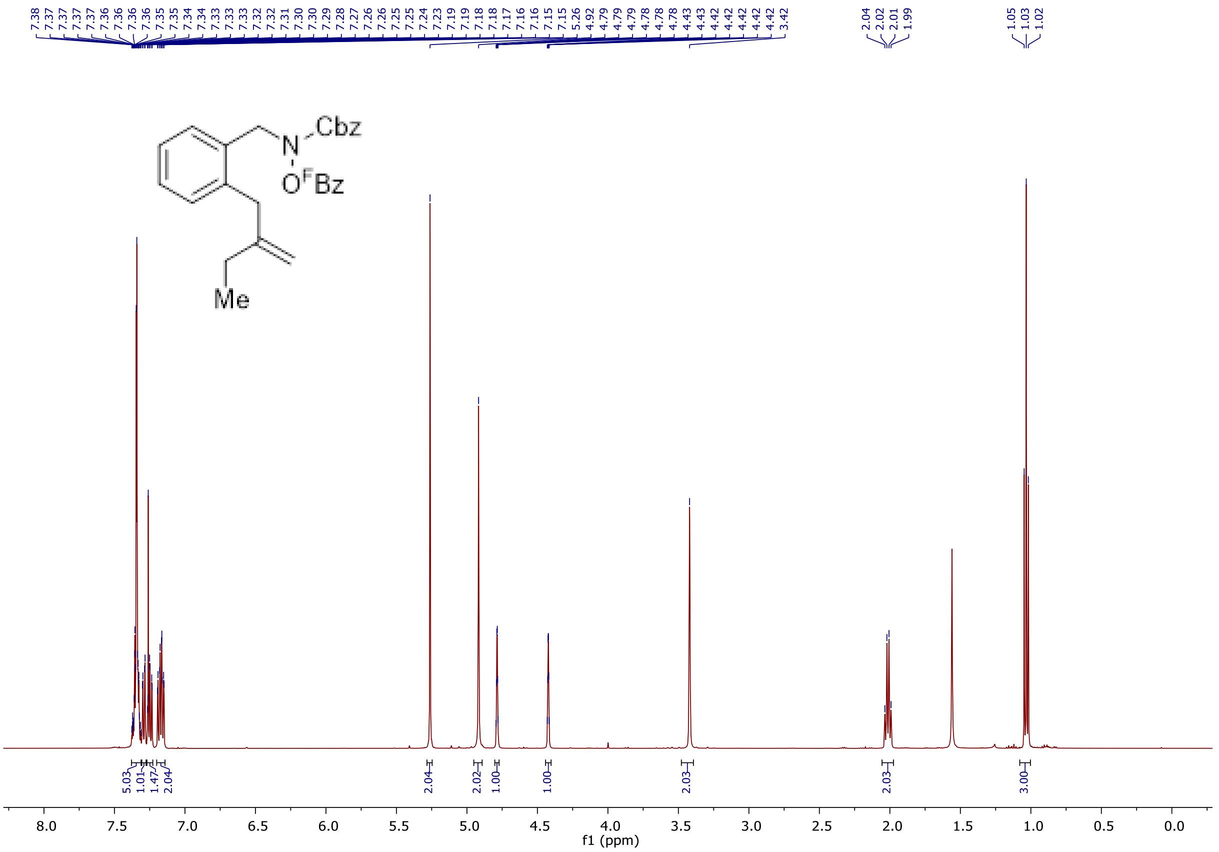

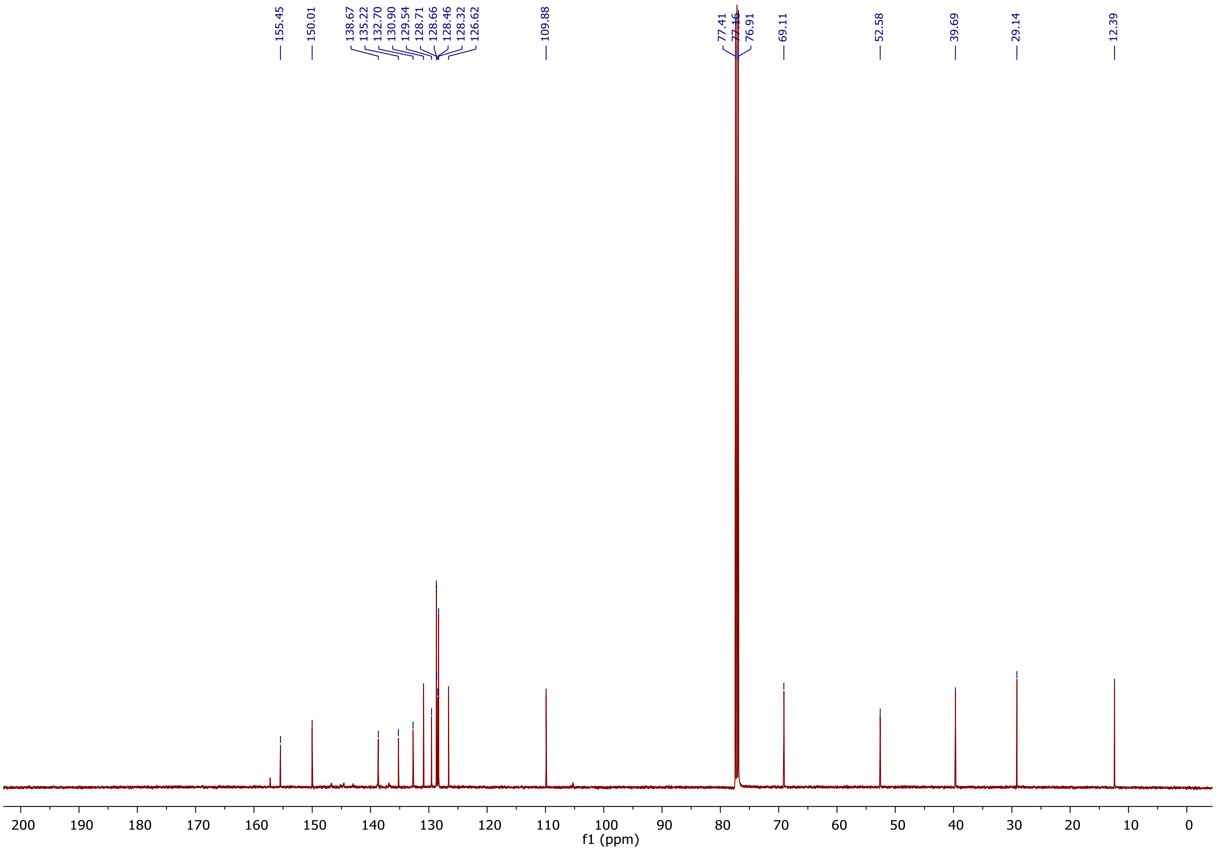

Benzyl 1a-ethyl-1,1a,3,7b-tetrahydro-2*H*-cyclopropa[*c*]isoquinoline-2-carboxylate (2n)

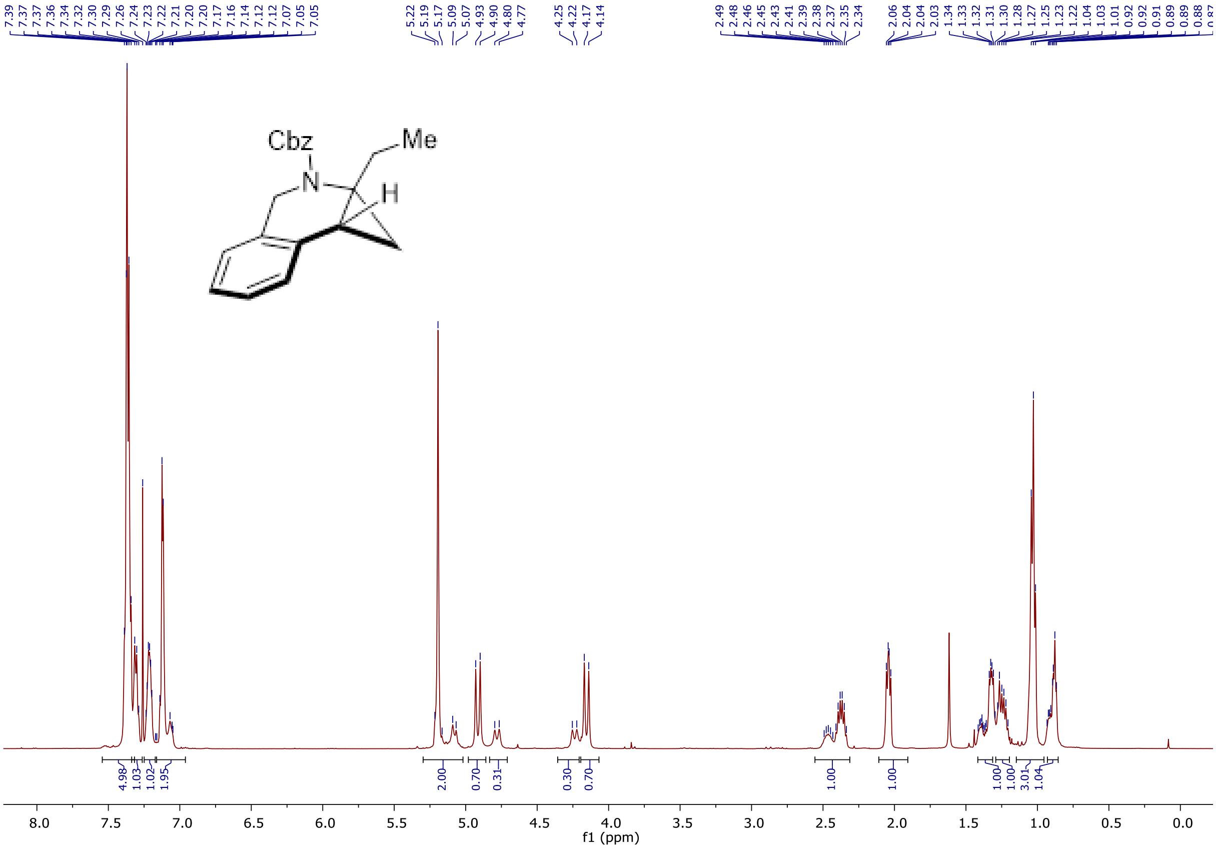

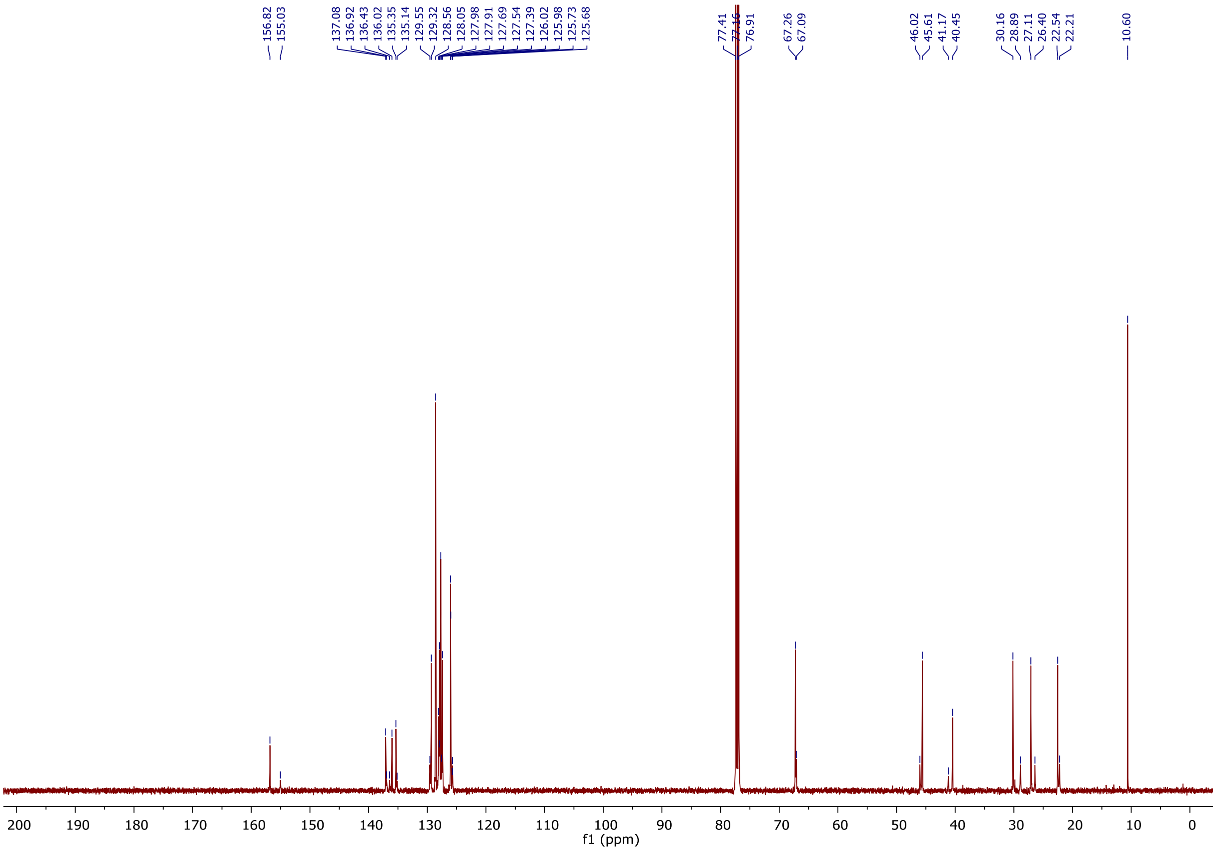

Methyl 2-(2-methyleneoctyl)benzoate

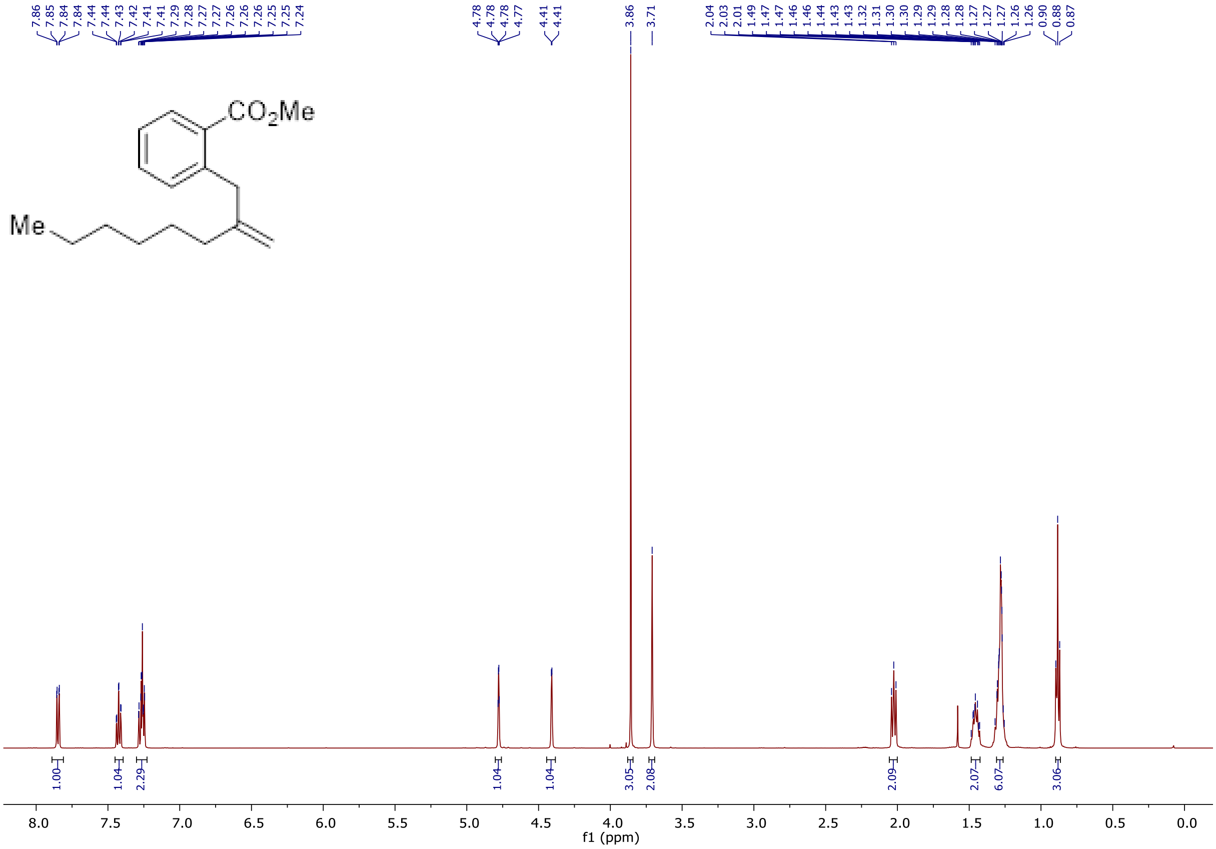

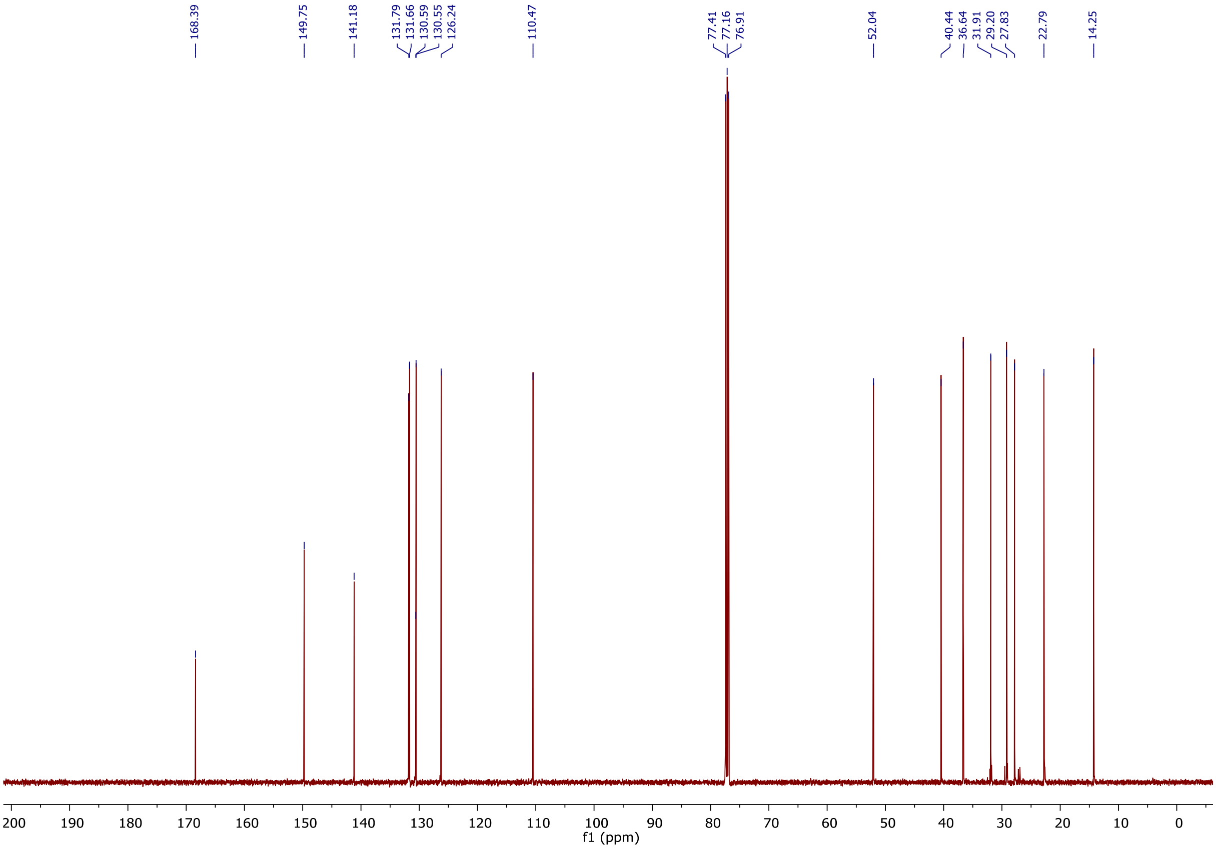

(2-(2-Methyleneoctyl)phenyl)methanol

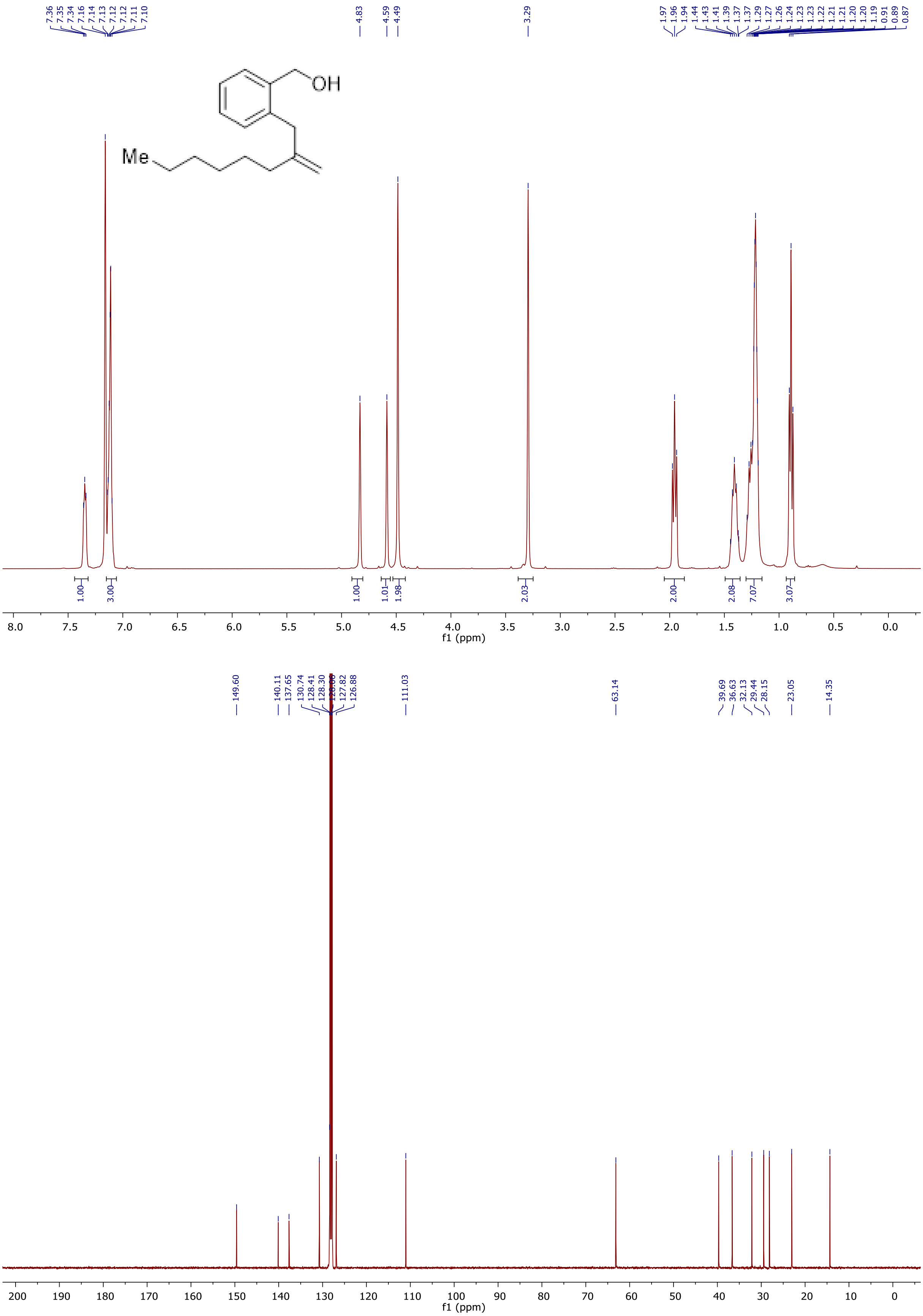

Benzyl (2-(2-methyleneoctyl)benzyl)((perfluorobenzoyl)oxy)carbamate (1o)

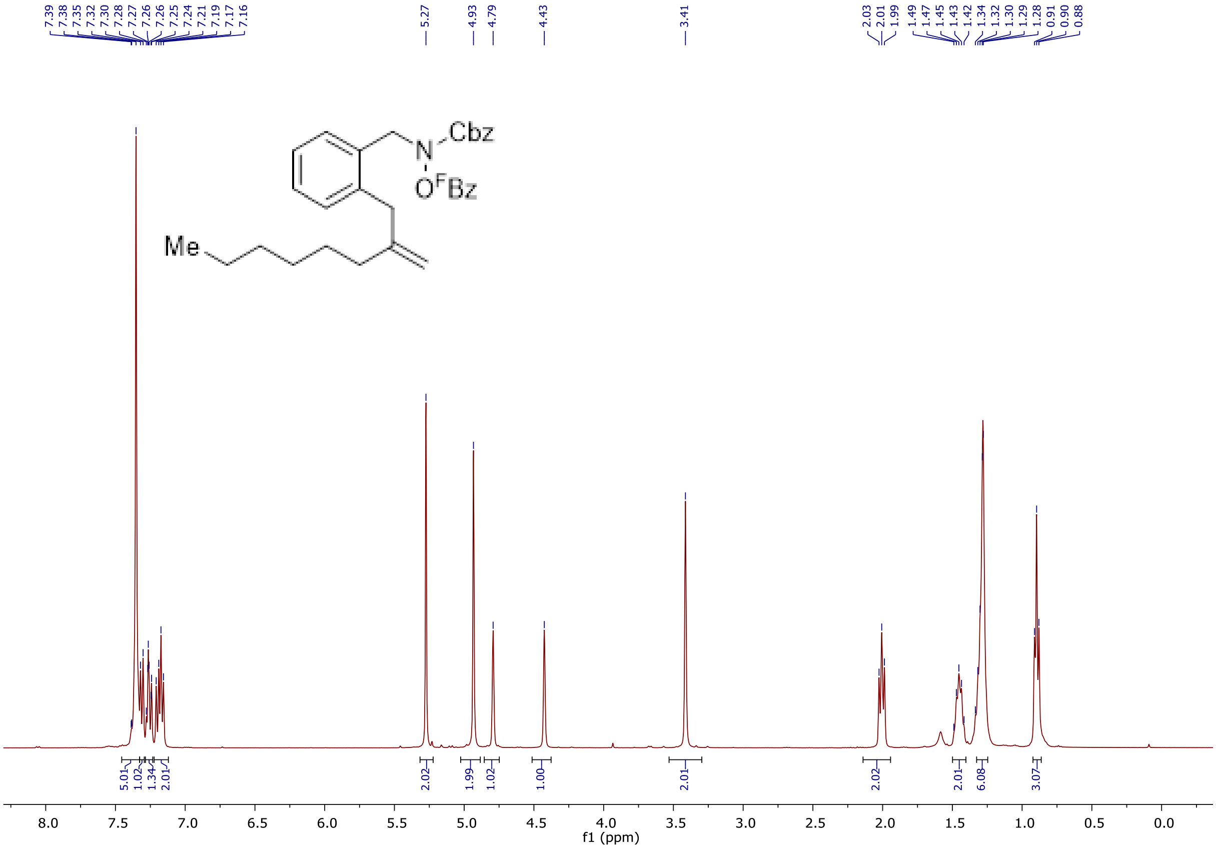

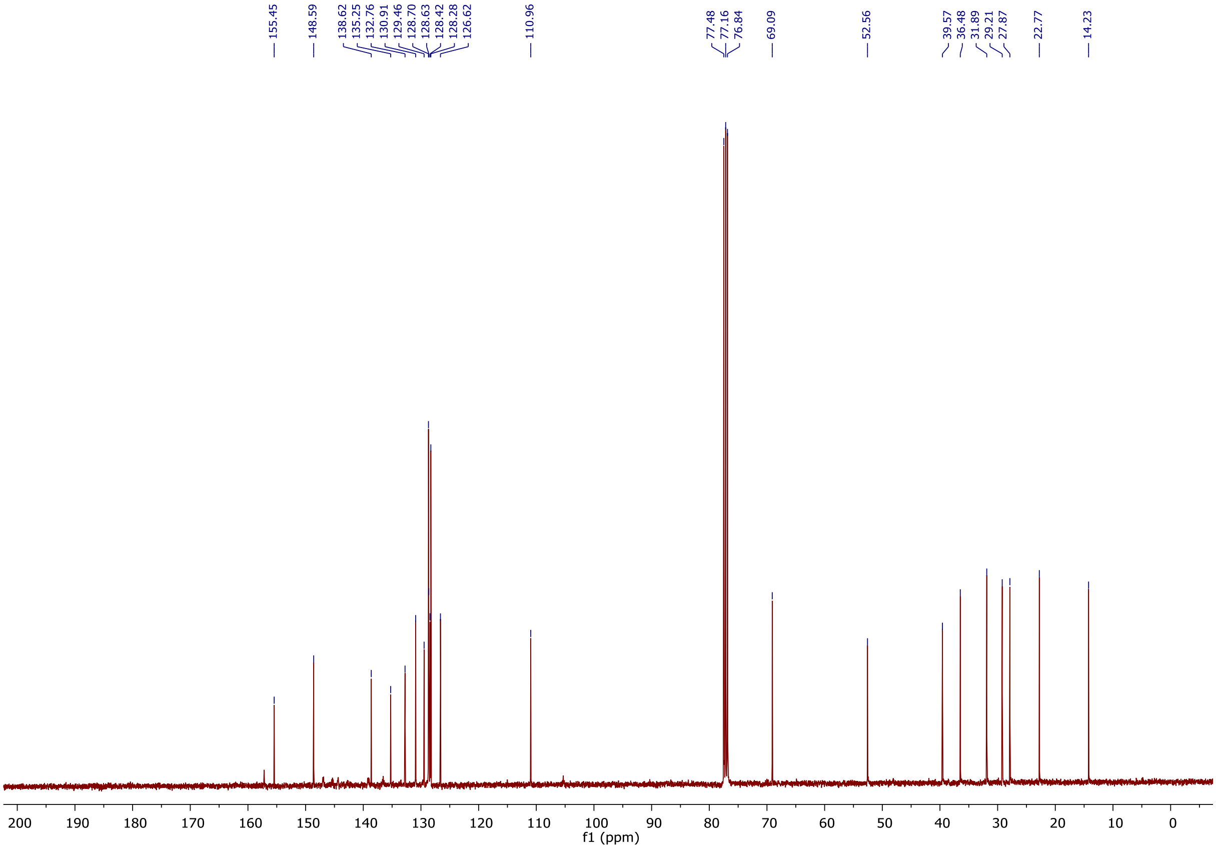

Benzyl 1a-hexyl-1,1a,3,7b-tetrahydro-2*H*-cyclopropa[*c*]isoquinoline-2-carboxylate (2o)

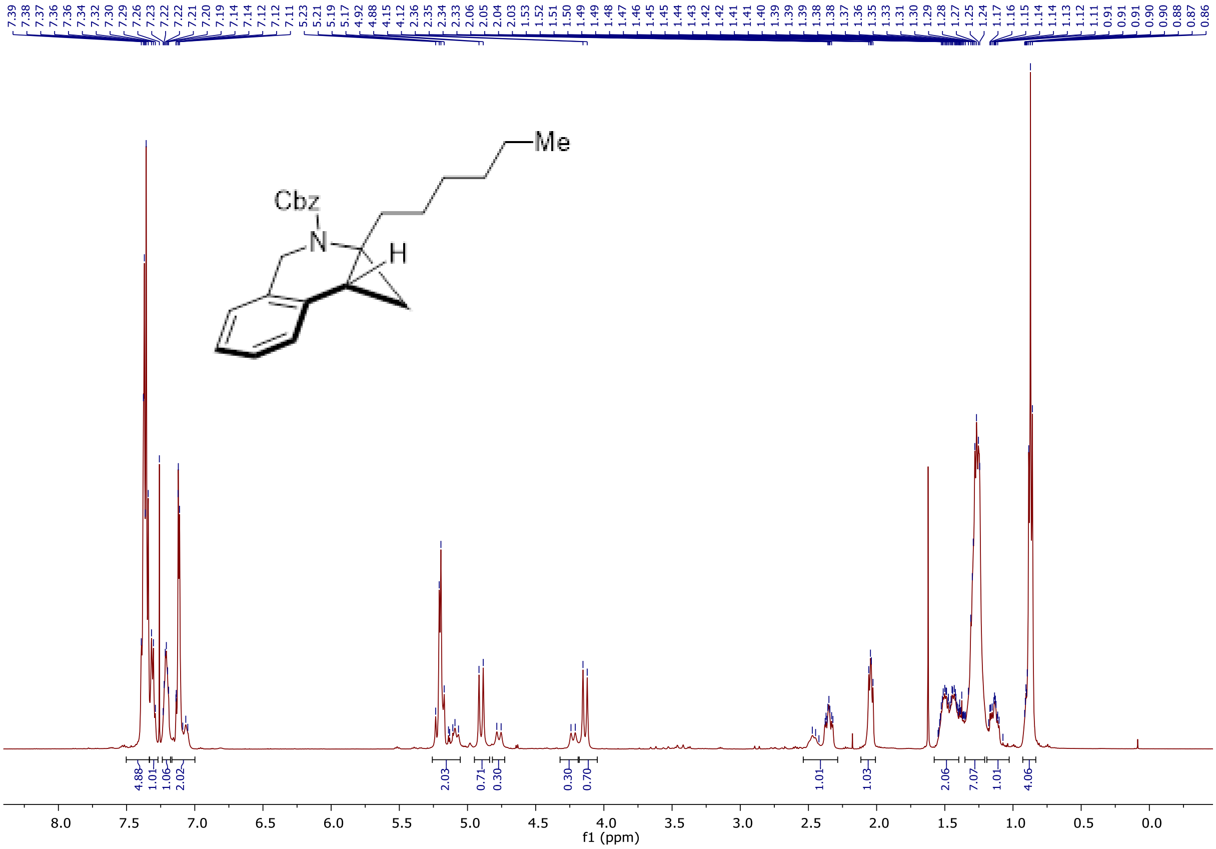

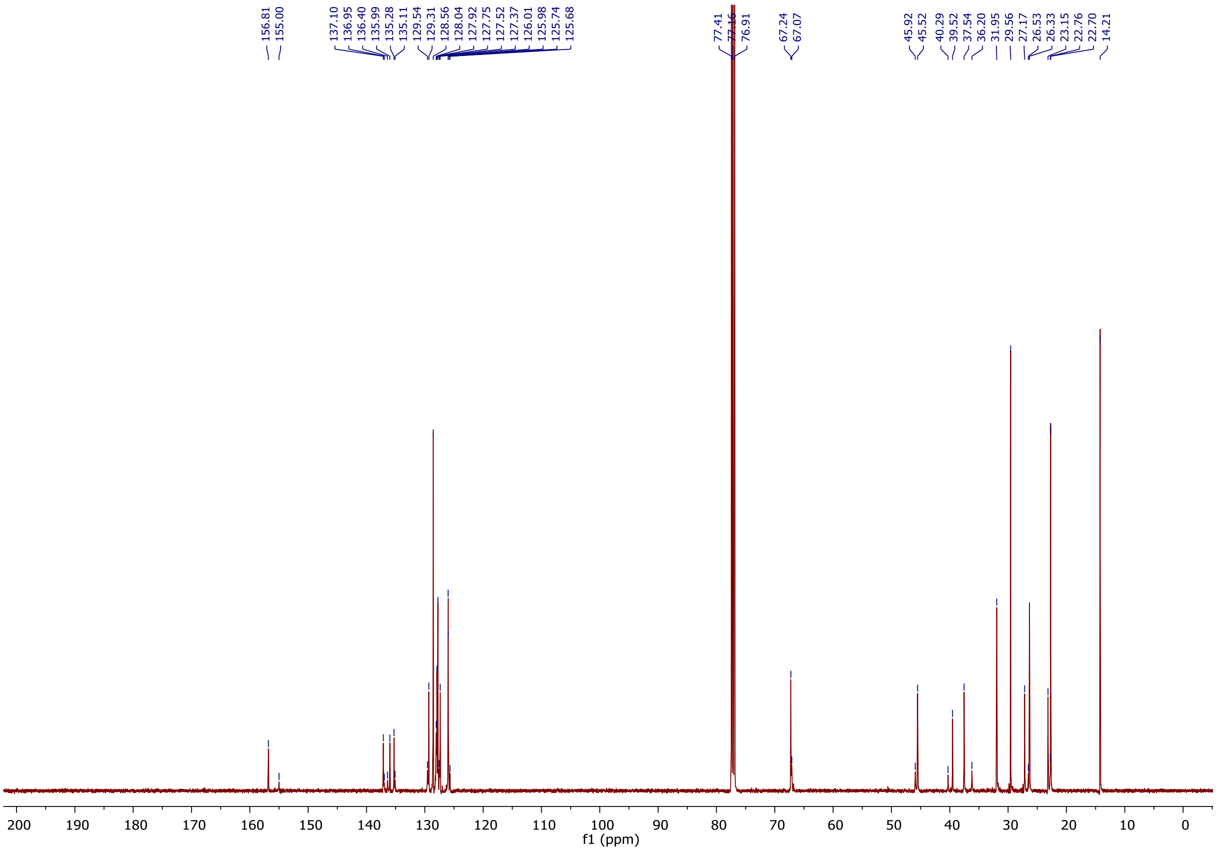

Methyl 2-(3-methyl-2-methylenebutyl)benzoate

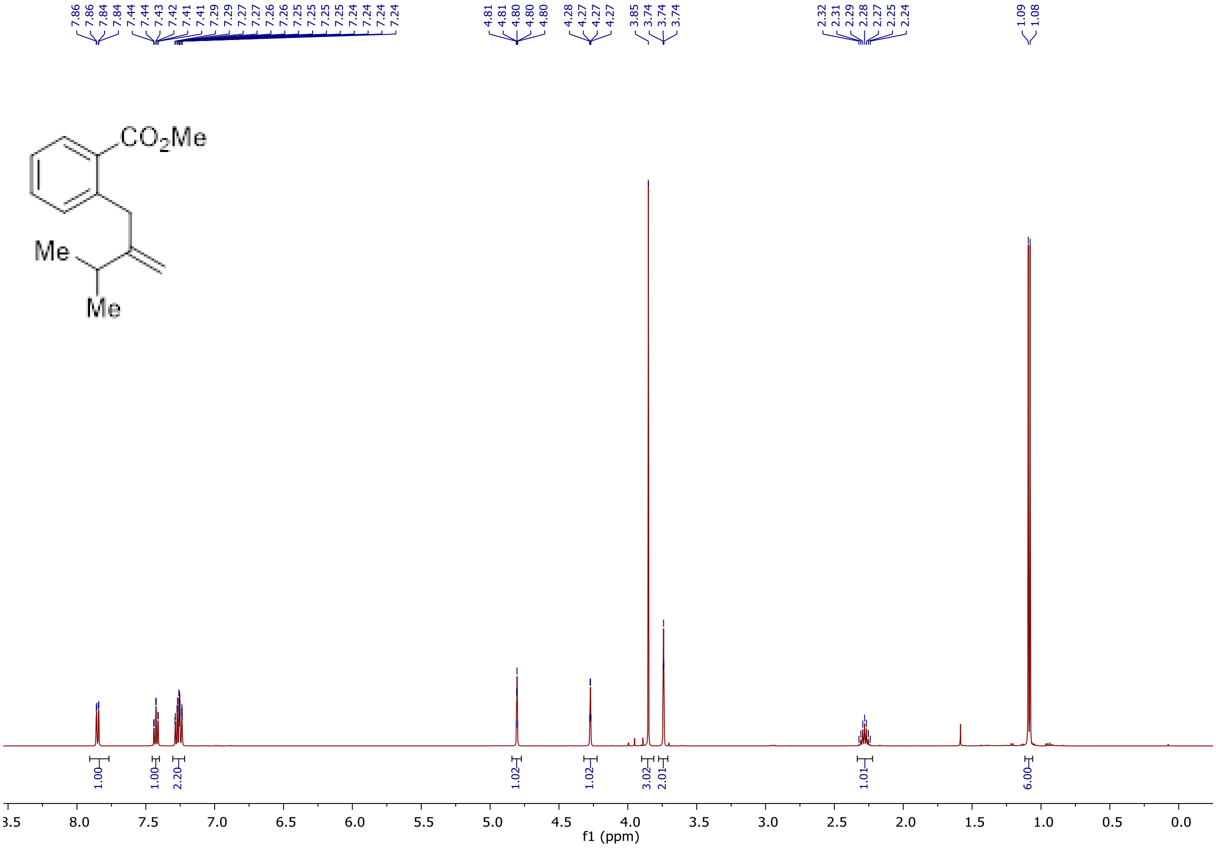

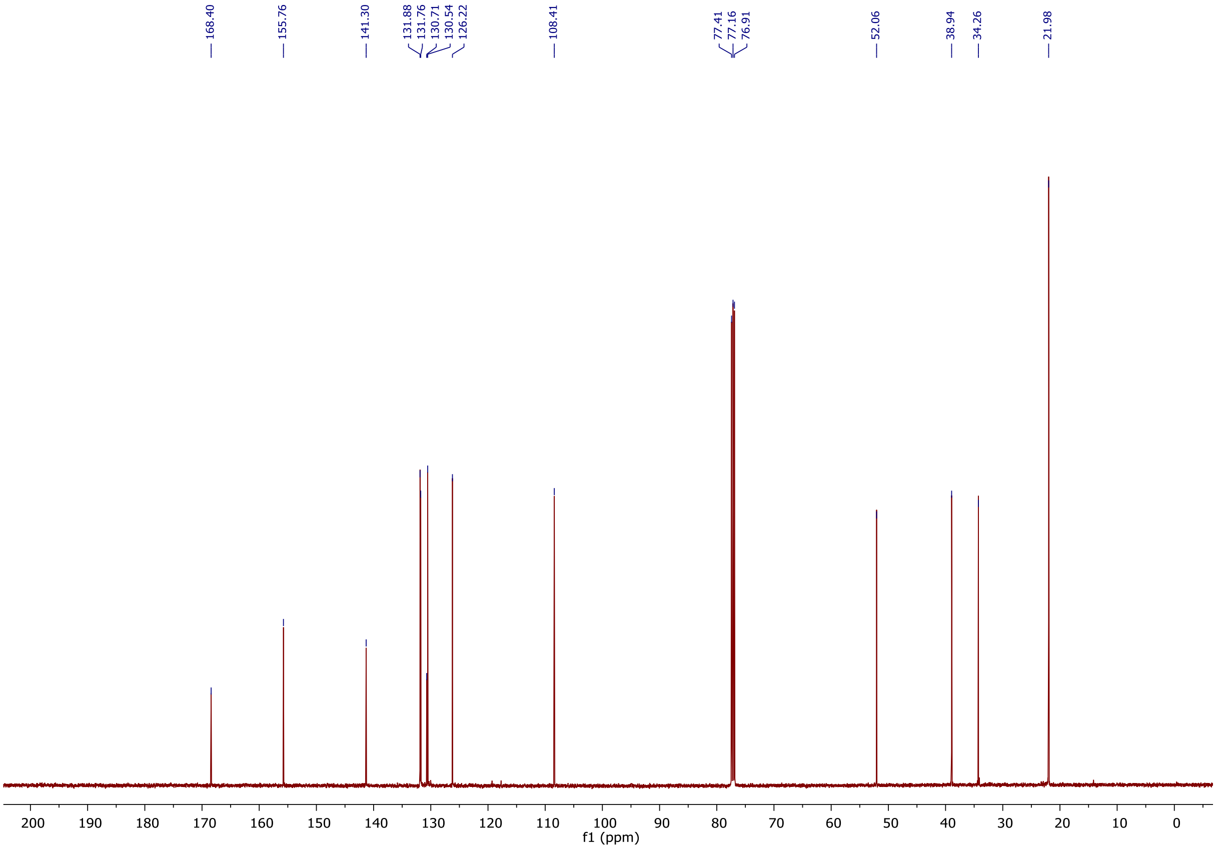

(2-(3-Methyl-2-methylenebutyl)phenyl)methanol

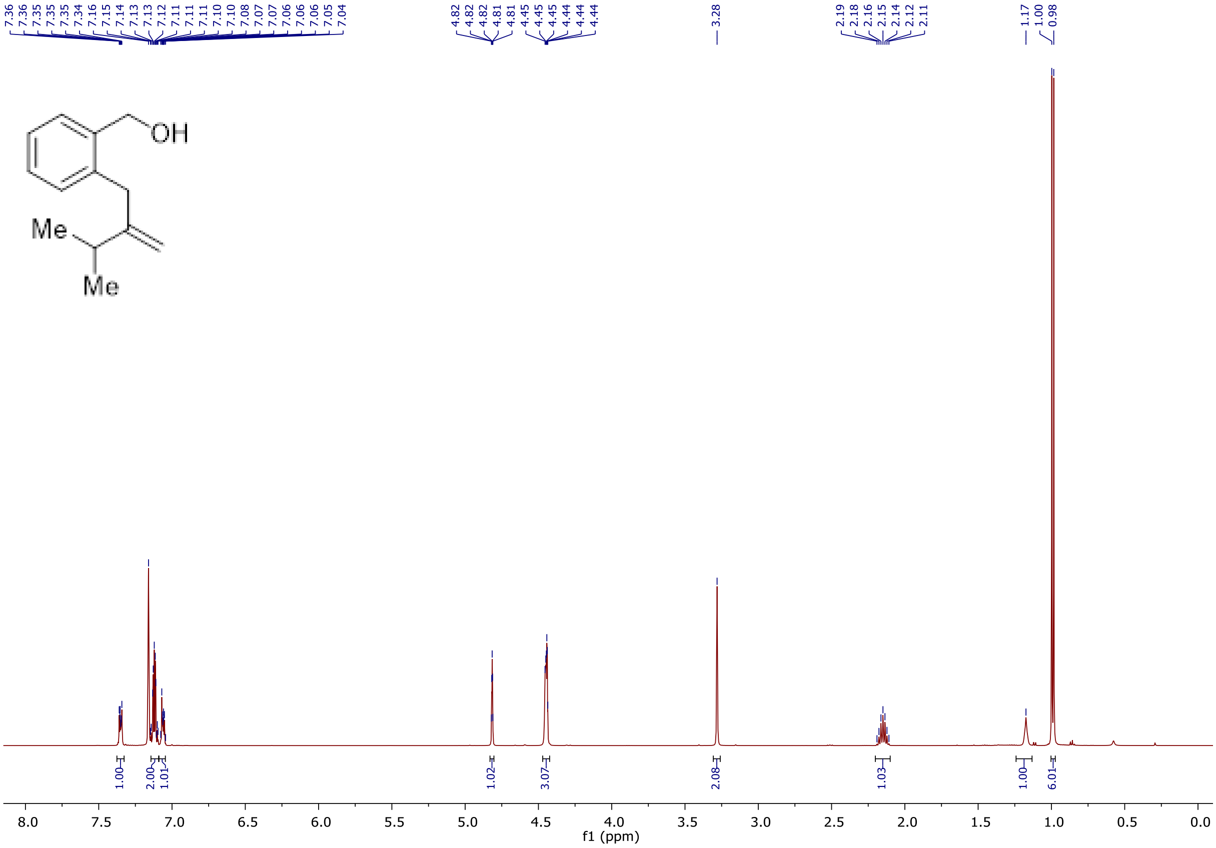

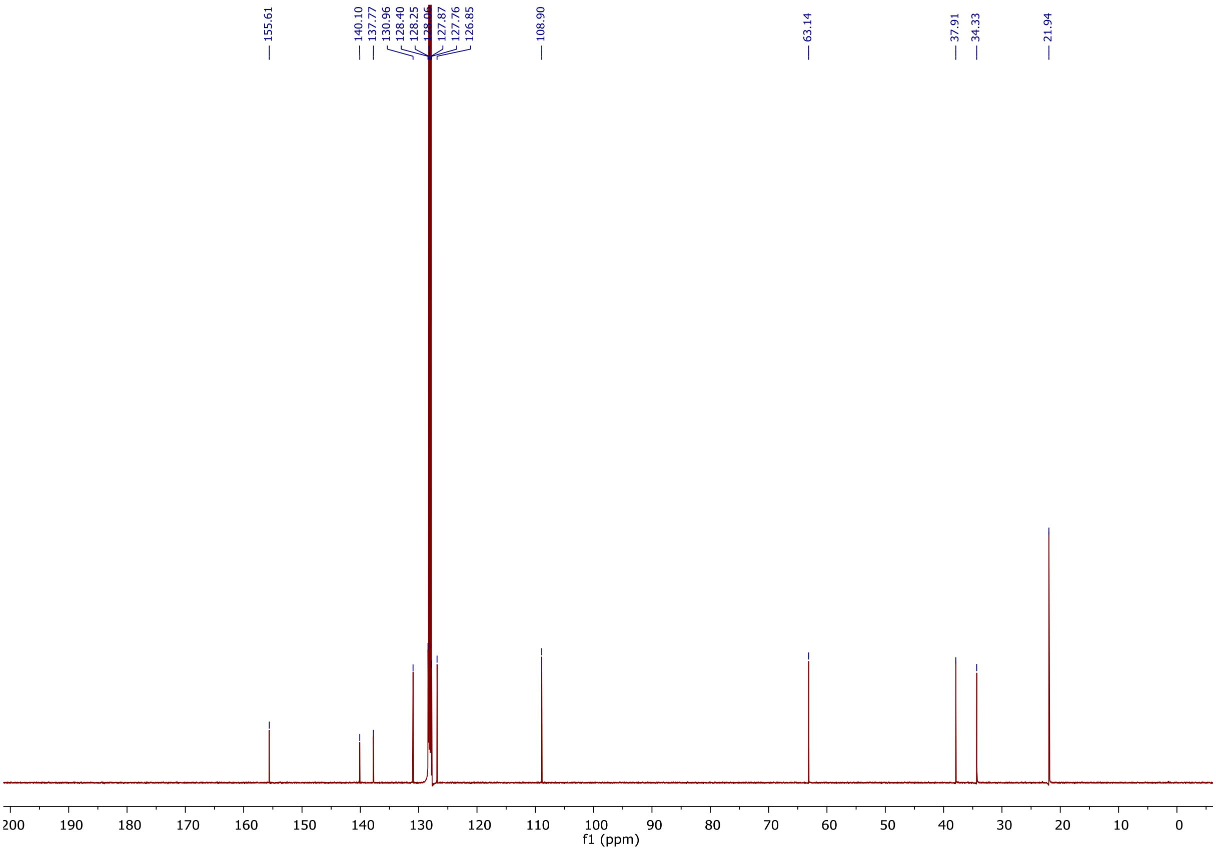

Benzyl (2-(3-methyl-2-methylenebutyl)benzyl)((perfluorobenzoyl)oxy)carbamate (1p)

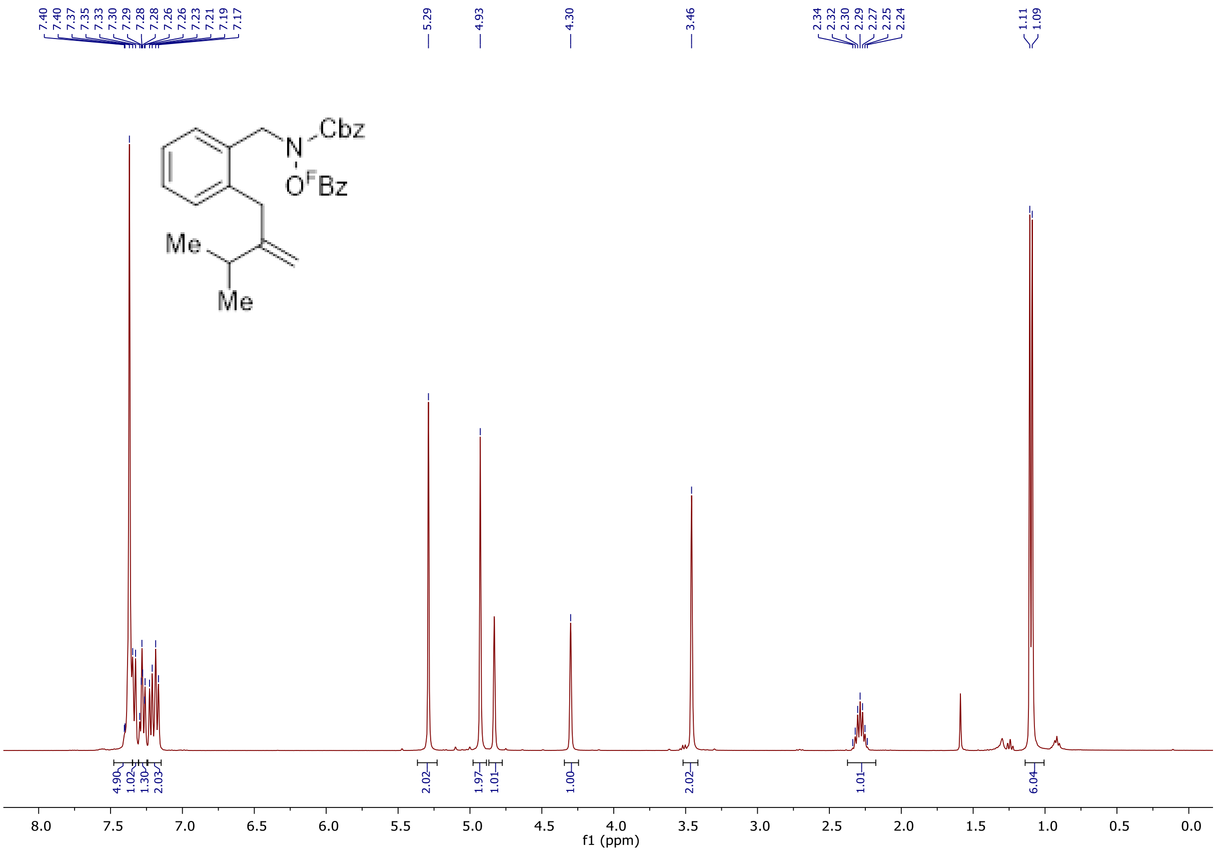

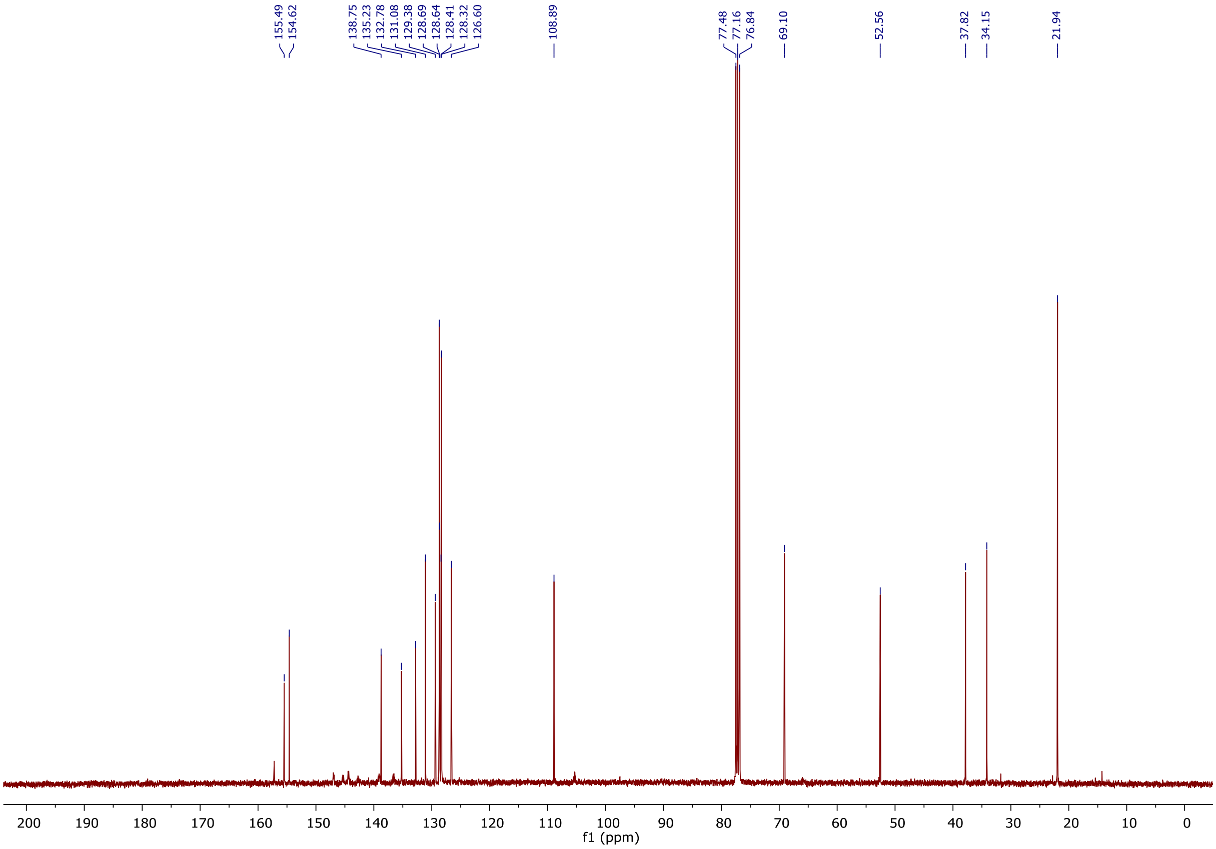

Benzyl 1a-isopropyl-1,1a,3,7b-tetrahydro-2*H*-cyclopropa[*c*]isoquinoline-2-carboxylate (2p)

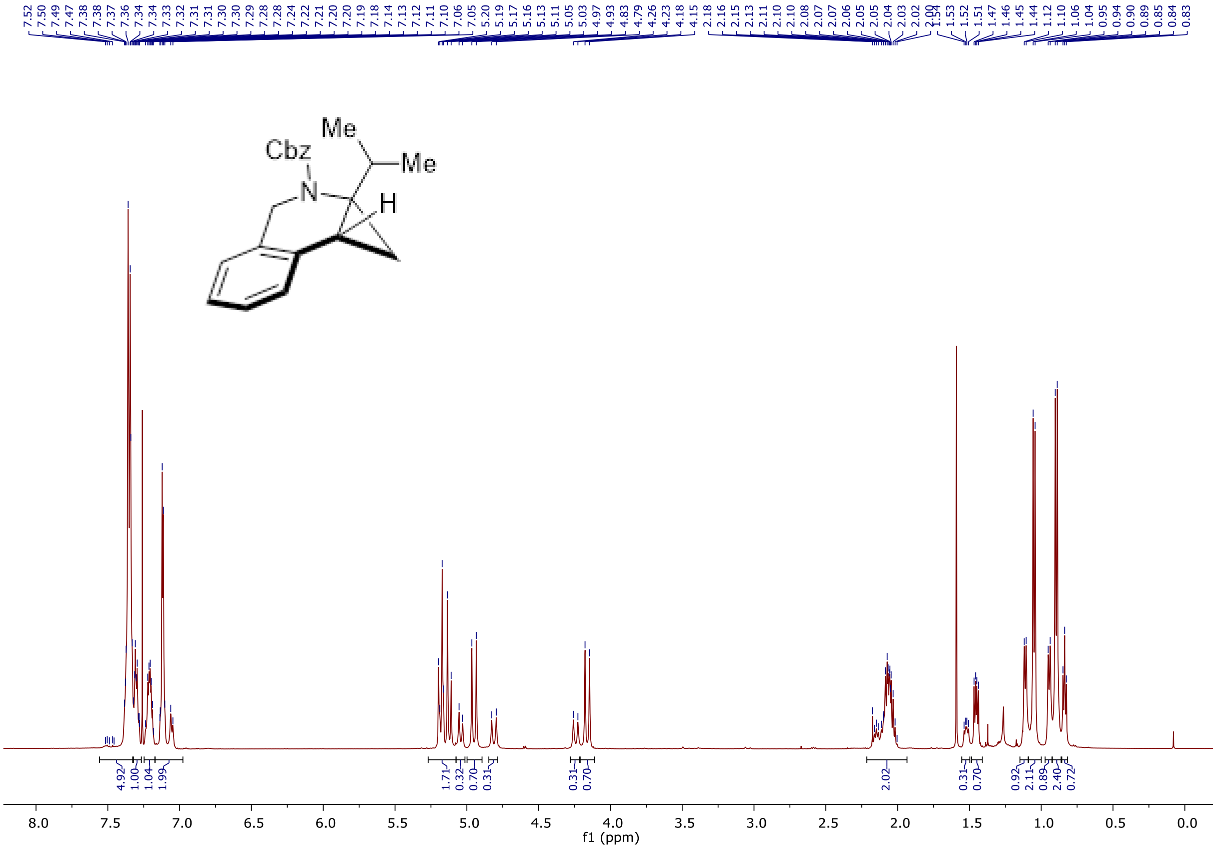

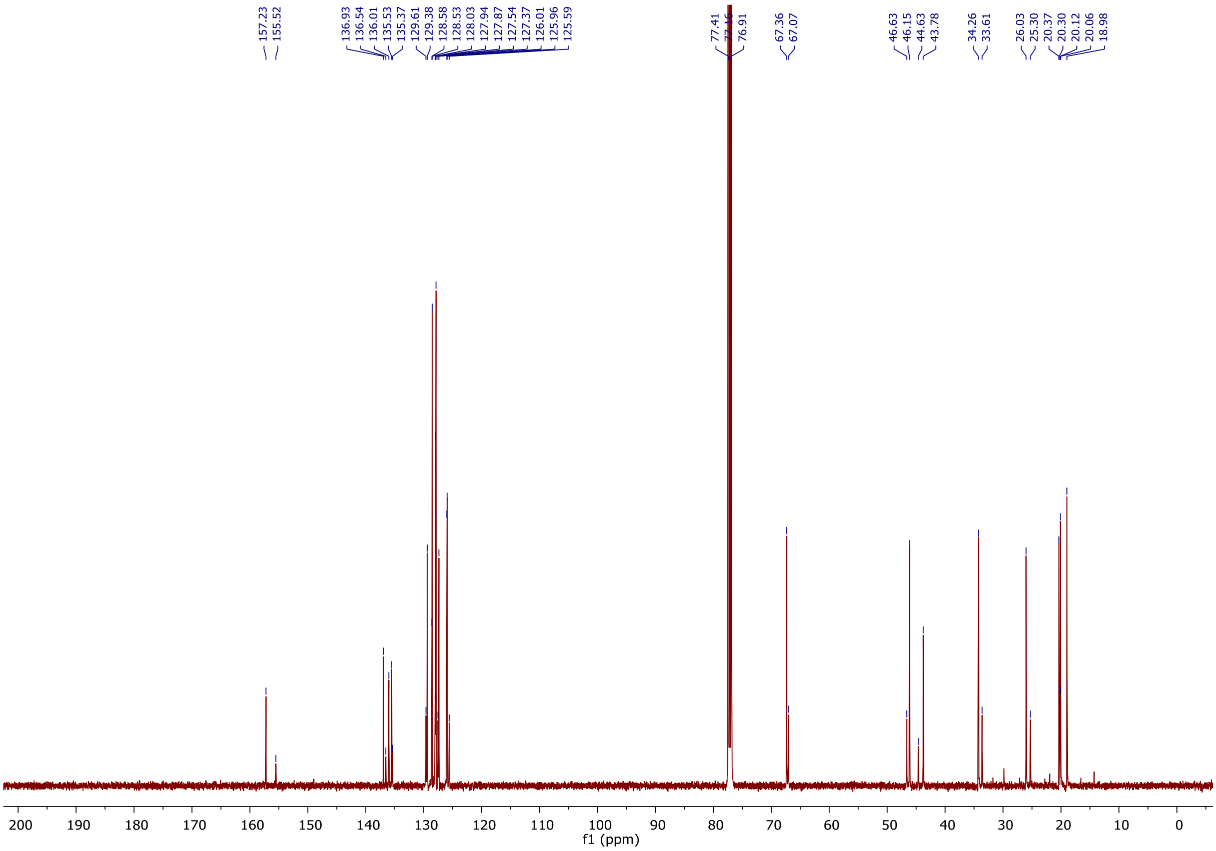

Methyl 5-methoxy-2-(2-methylenebutyl)benzoate

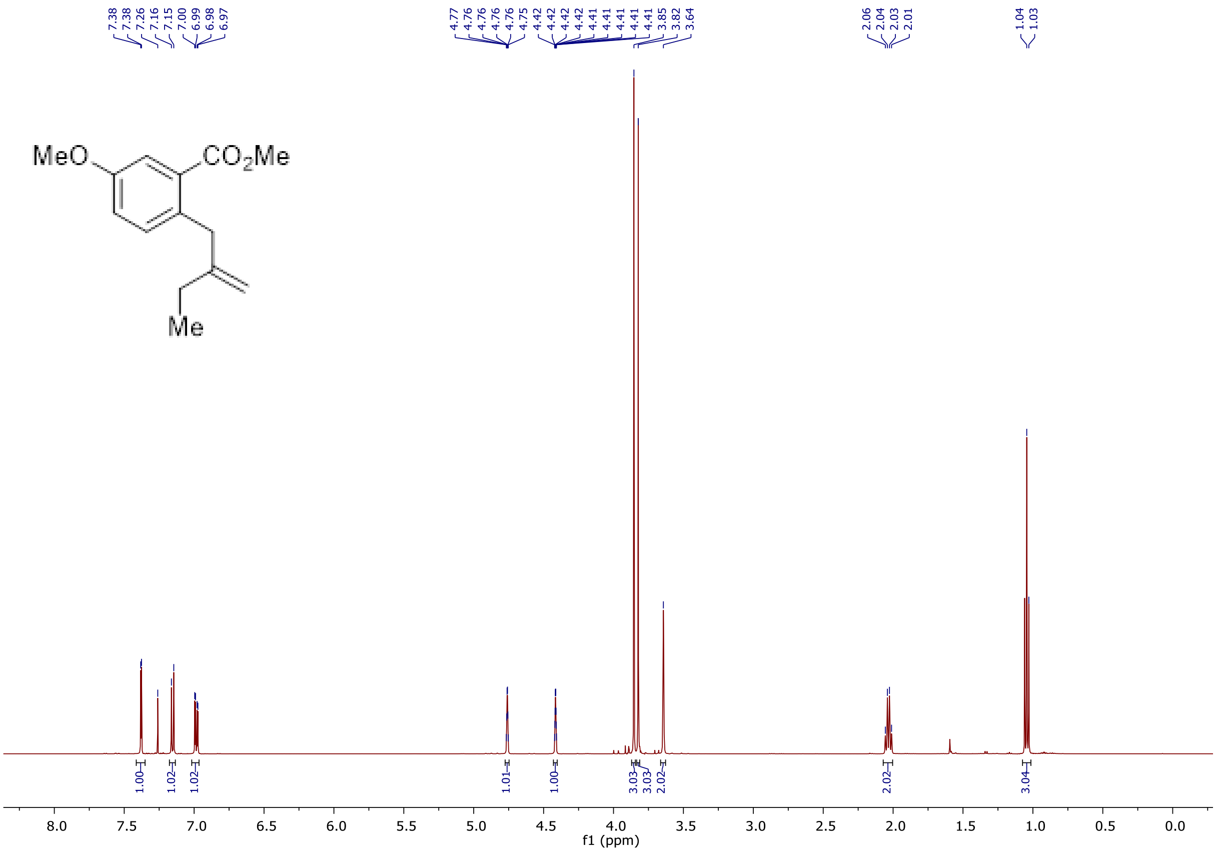

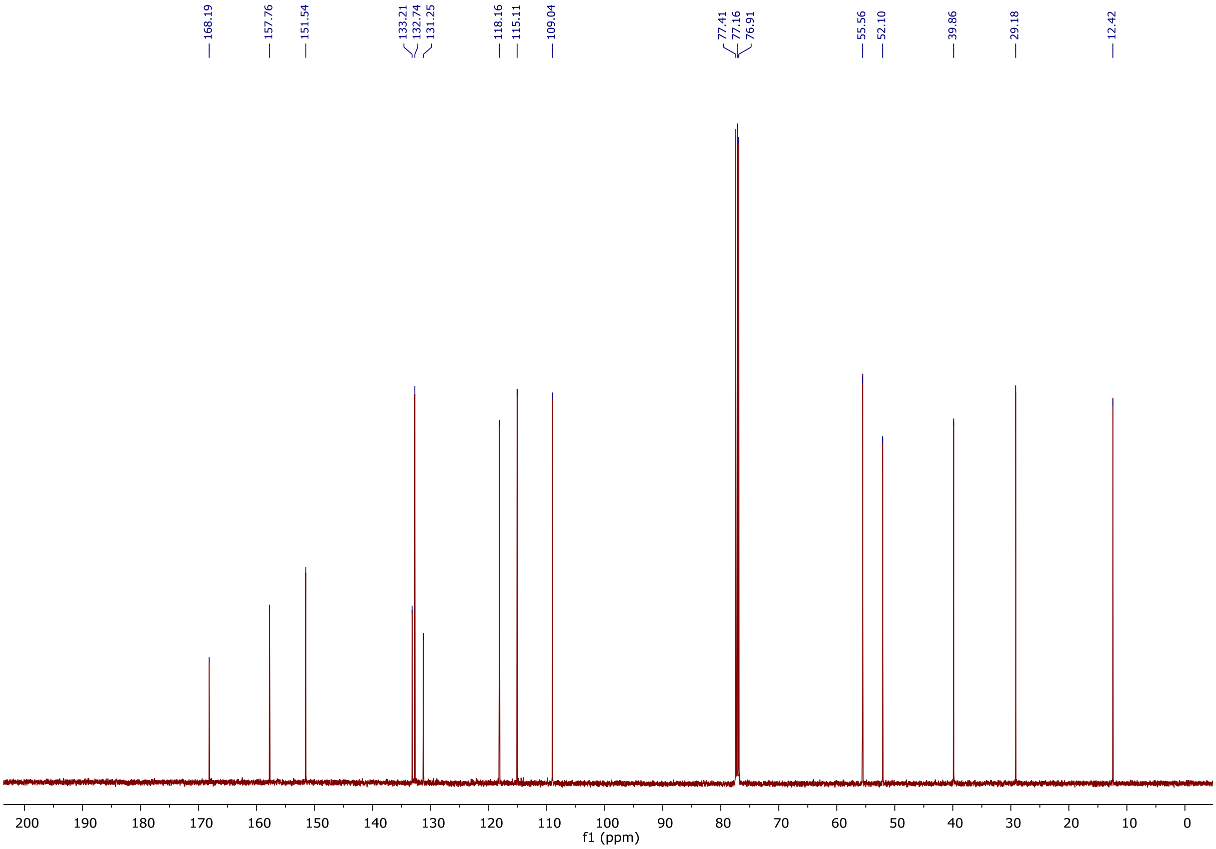

(5-Methoxy-2-(2-methylenebutyl)phenyl)methanol

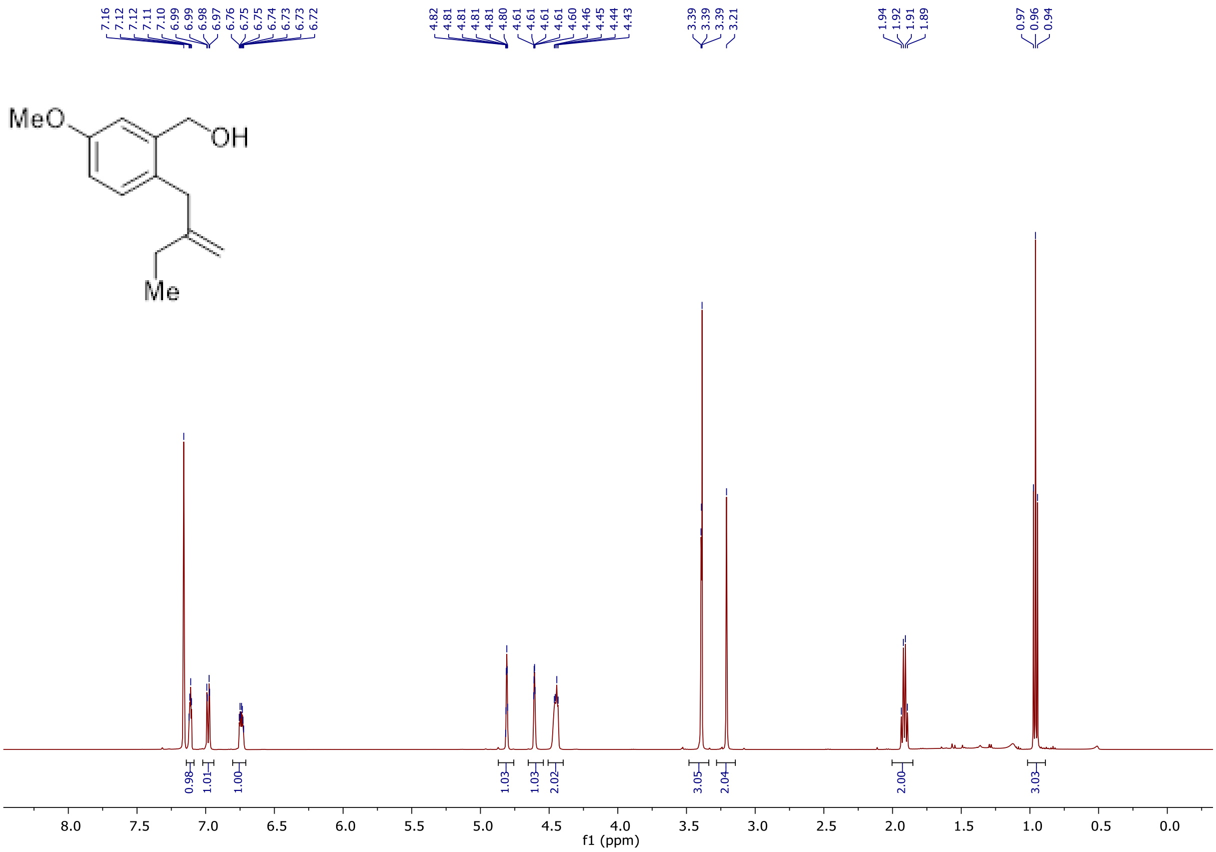

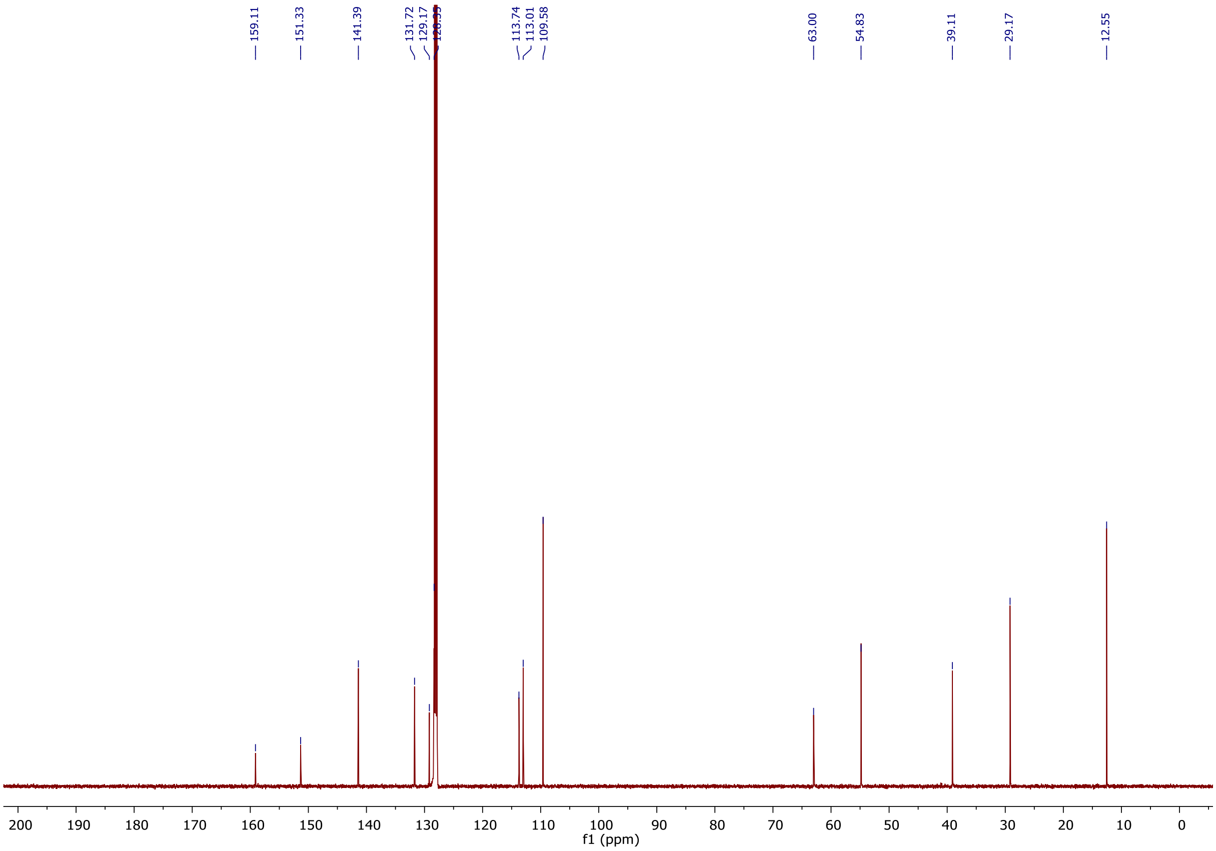

Benzyl (5-methoxy-2-(2-methylenebutyl)benzyl)((perfluorobenzoyl)oxy)carbamate (1q)

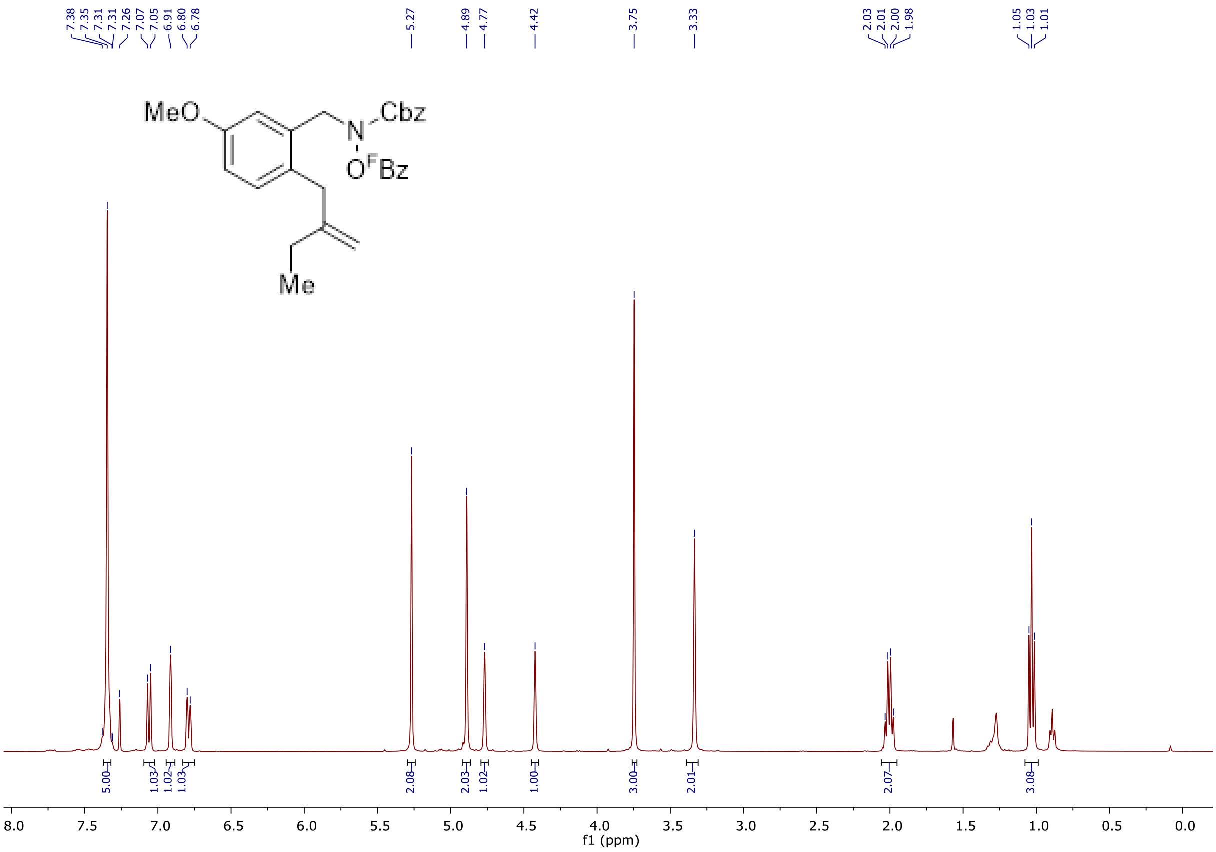

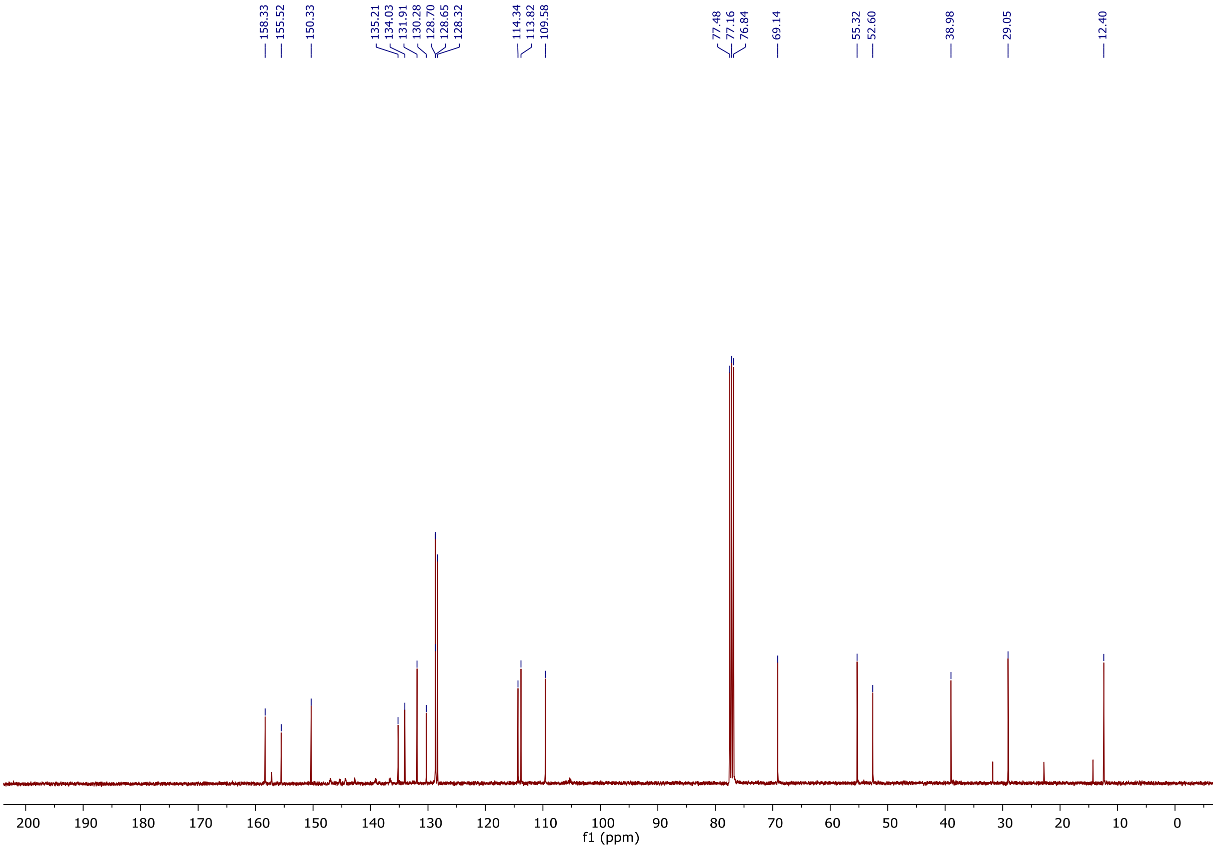

Benzyl 1a-ethyl-5-methoxy-1,1a,3,7b-tetrahydro-2*H*-cyclopropa[*c*]isoquinoline-2-carboxylate (2q)

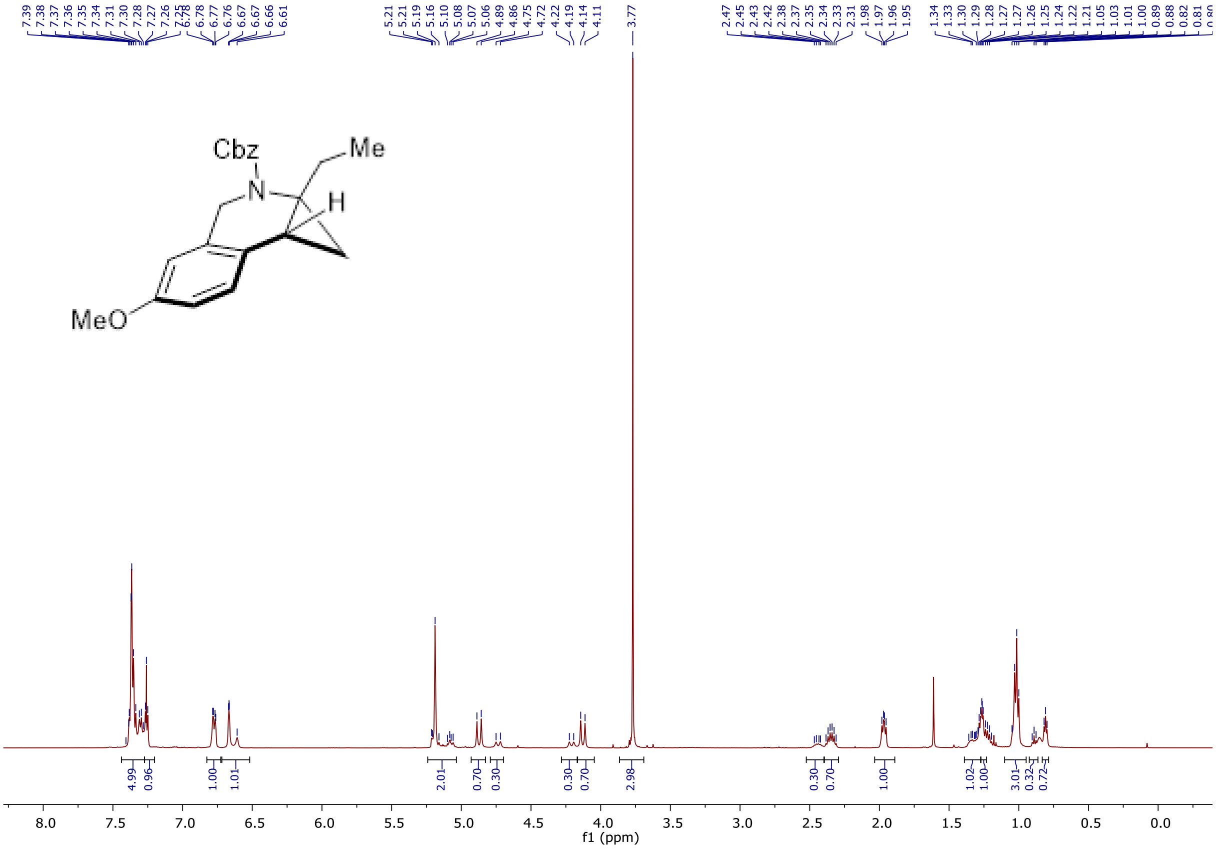

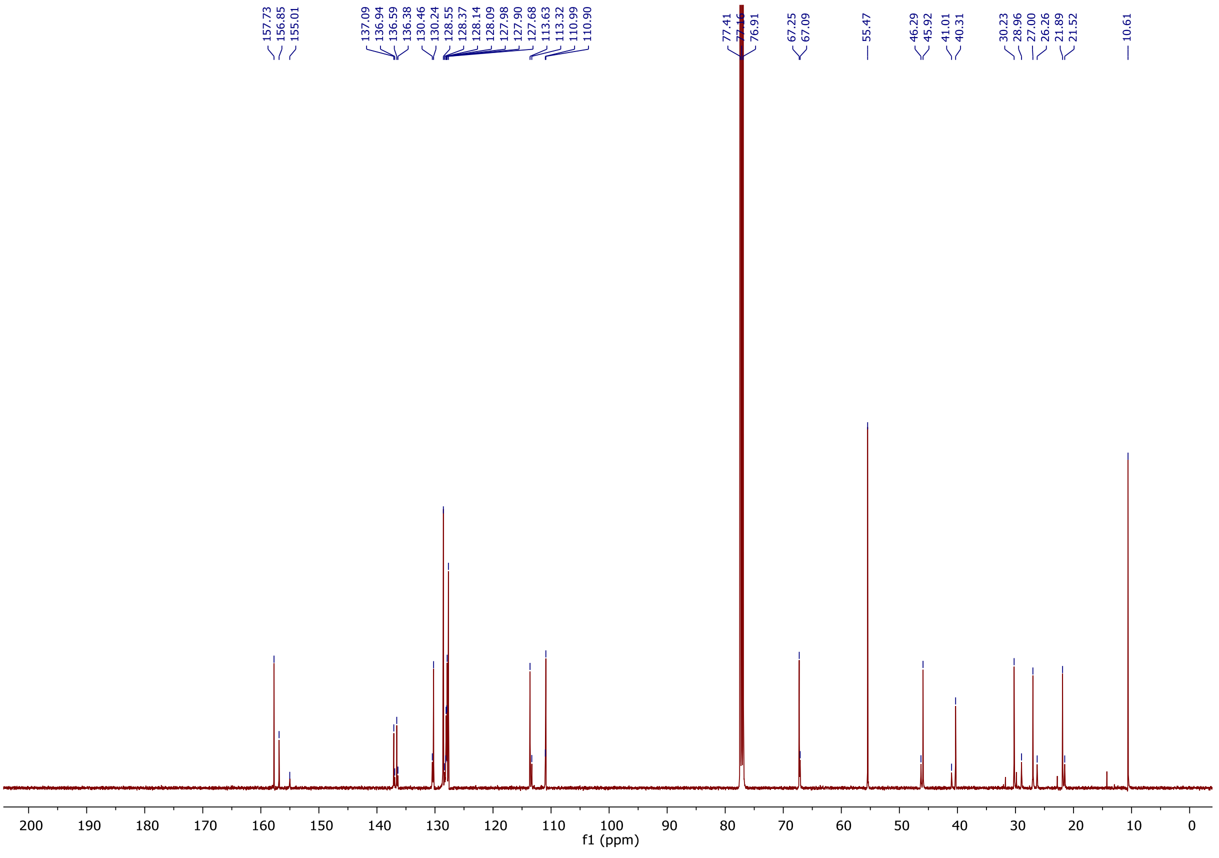

Methyl 4-methoxy-2-(2-methylenebutyl)benzoate

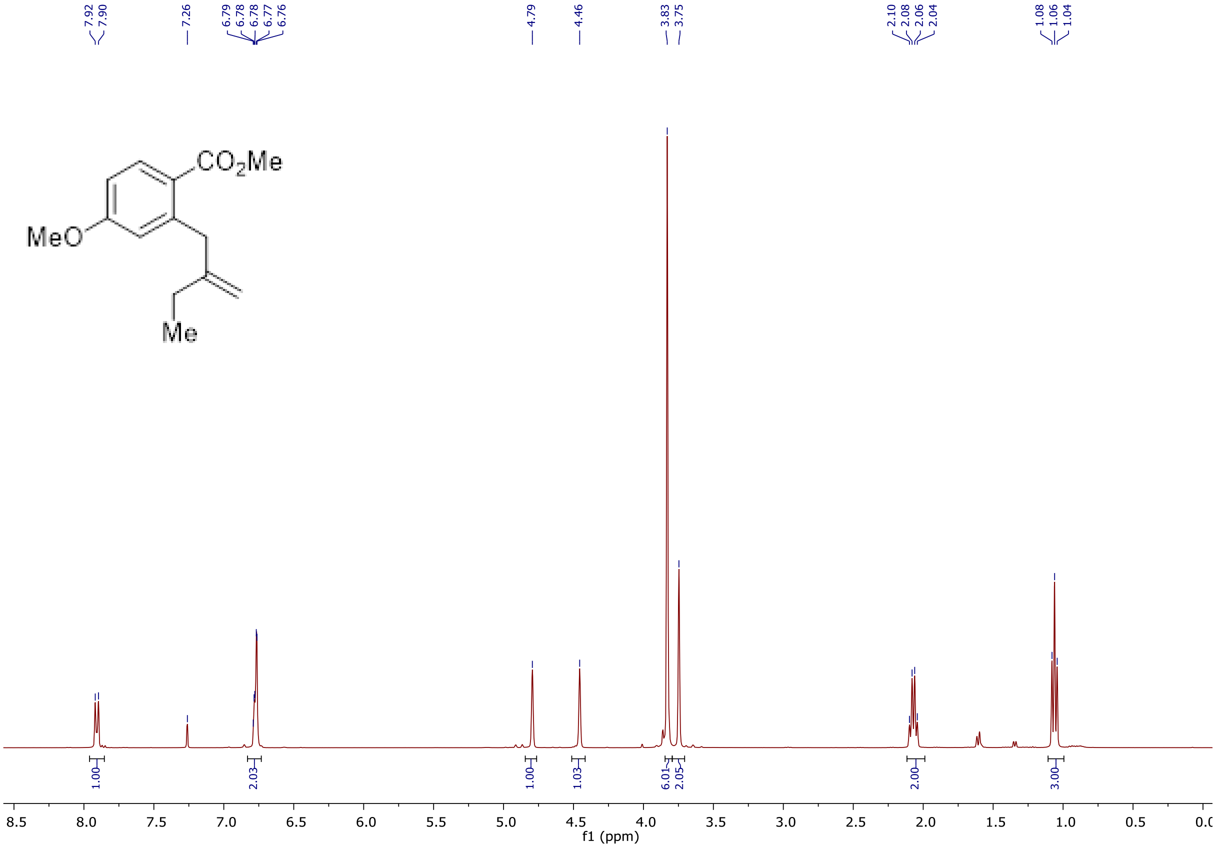

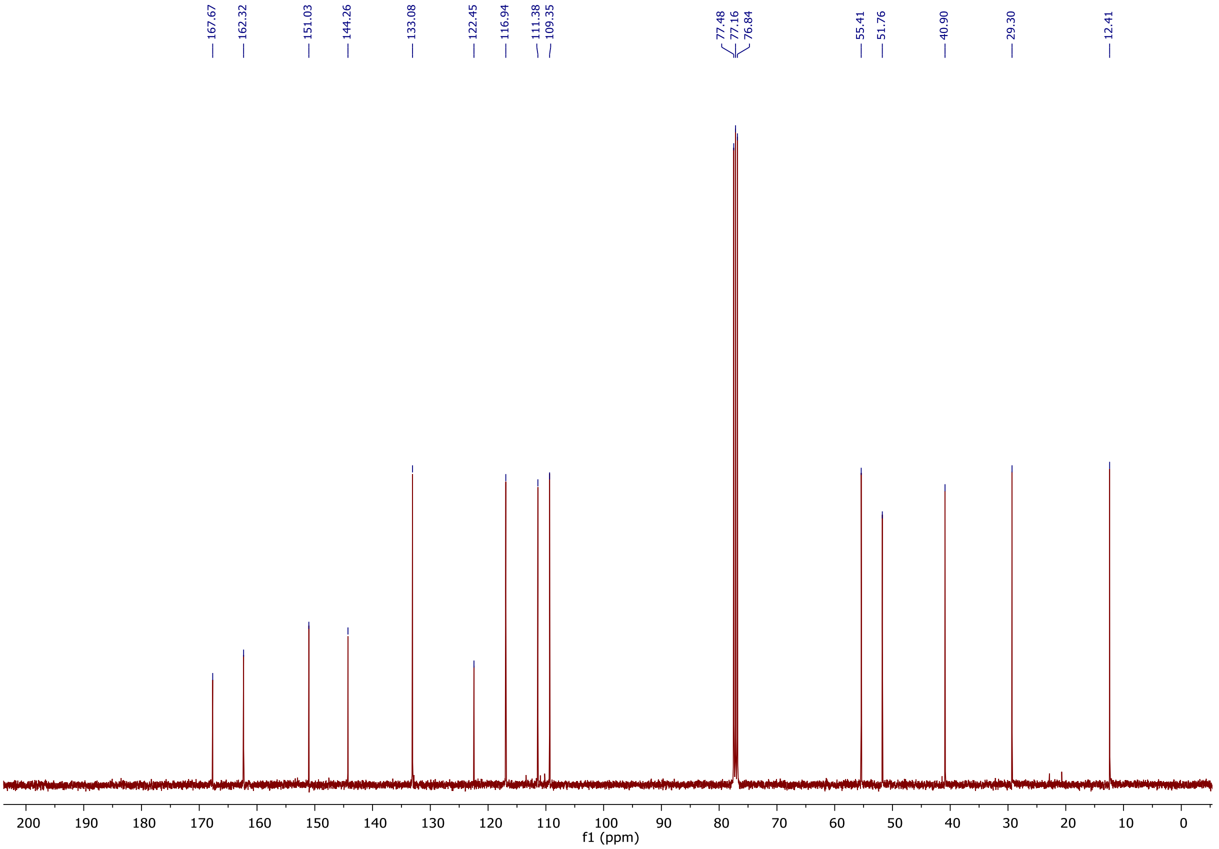

(4-Methoxy-2-(2-methylenebutyl)phenyl)methanol

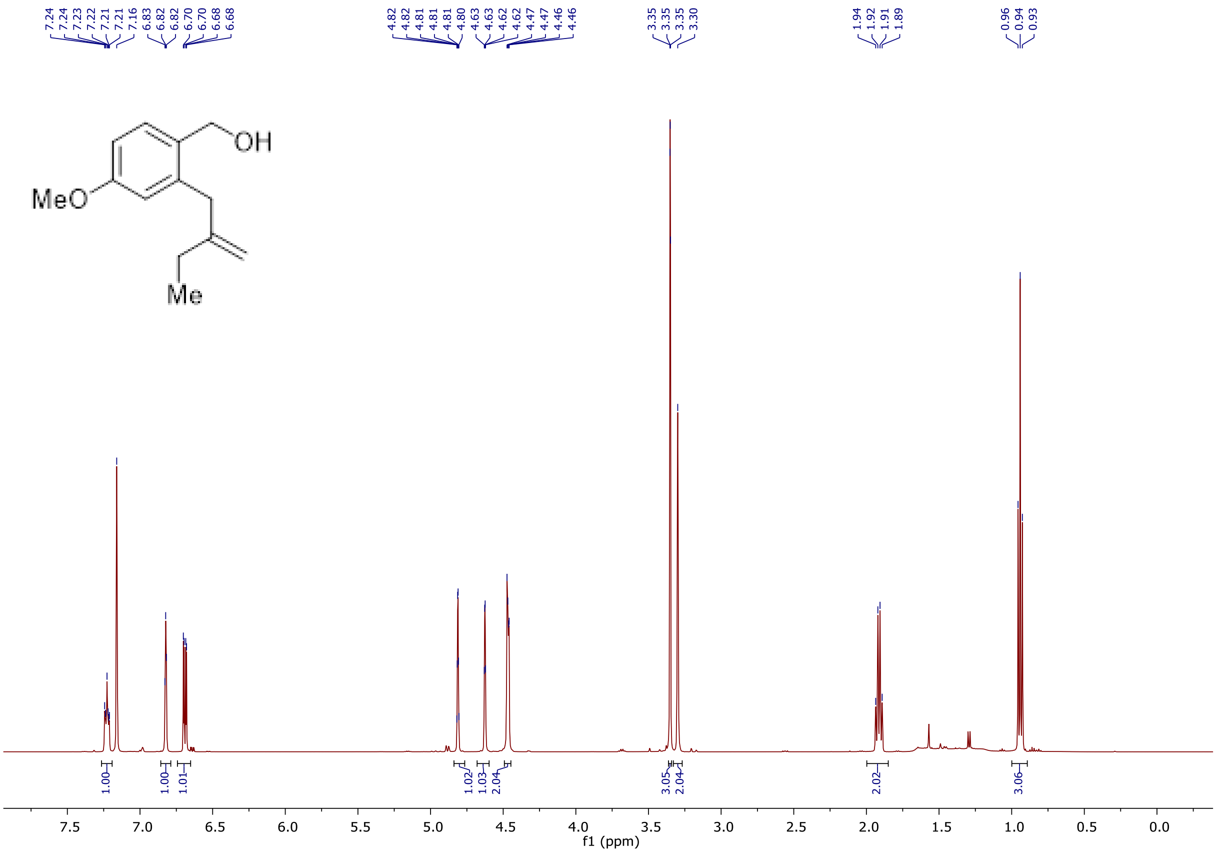

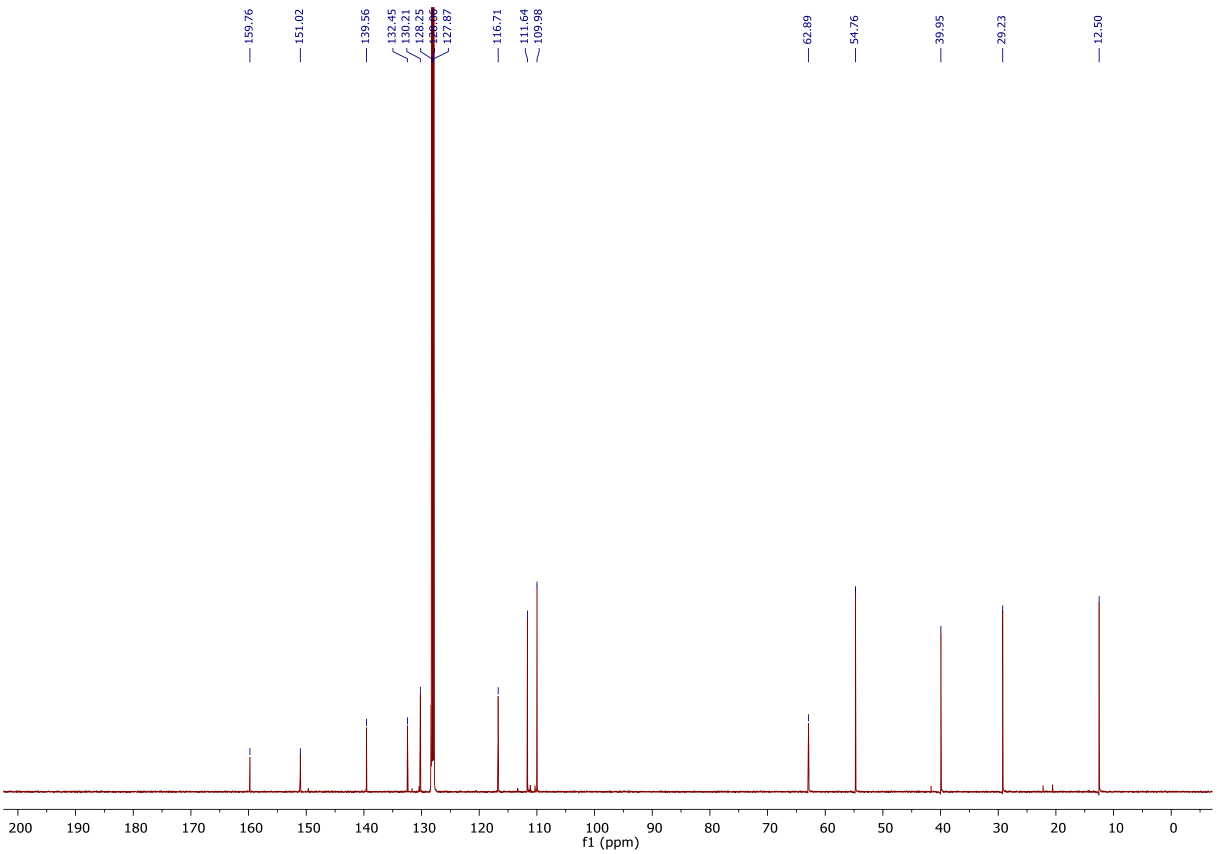

Benzyl (4-methoxy-2-(2-methylenebutyl)benzyl)((perfluorobenzoyl)oxy)carbamate (1r)

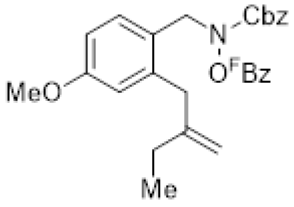

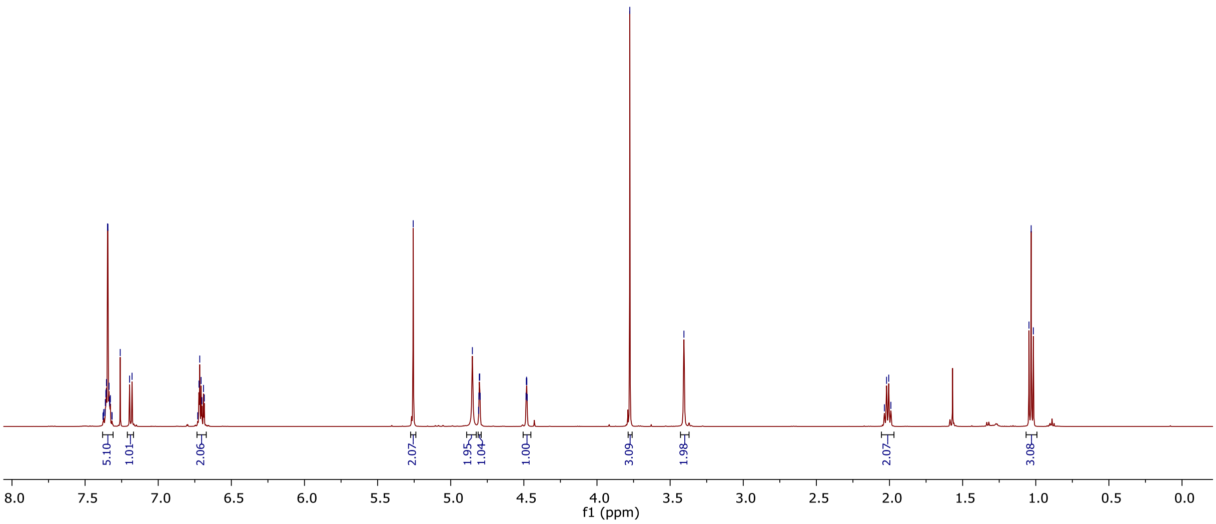

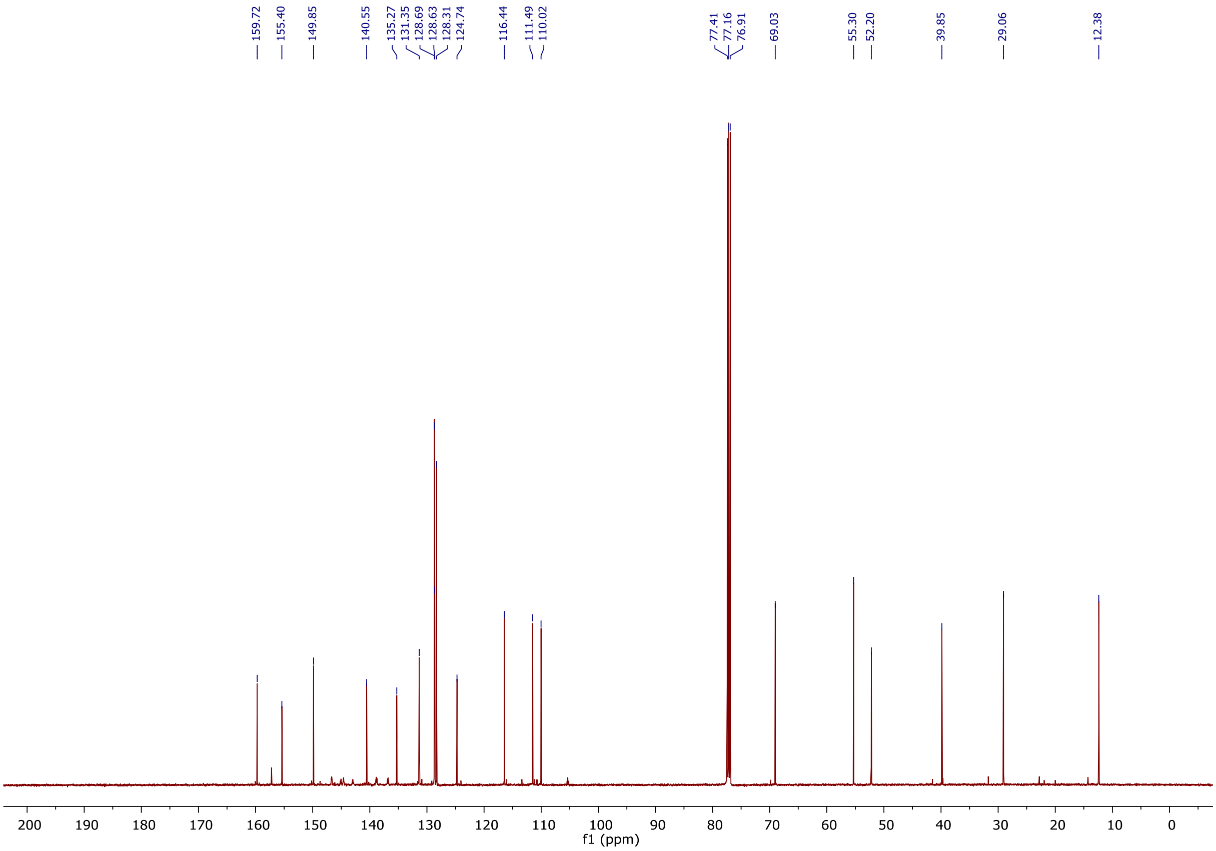

Benzyl 1a-ethyl-6-methoxy-1,1a,3,7b-tetrahydro-2*H*-cyclopropa[*c*]isoquinoline-2-carboxylate (2r)

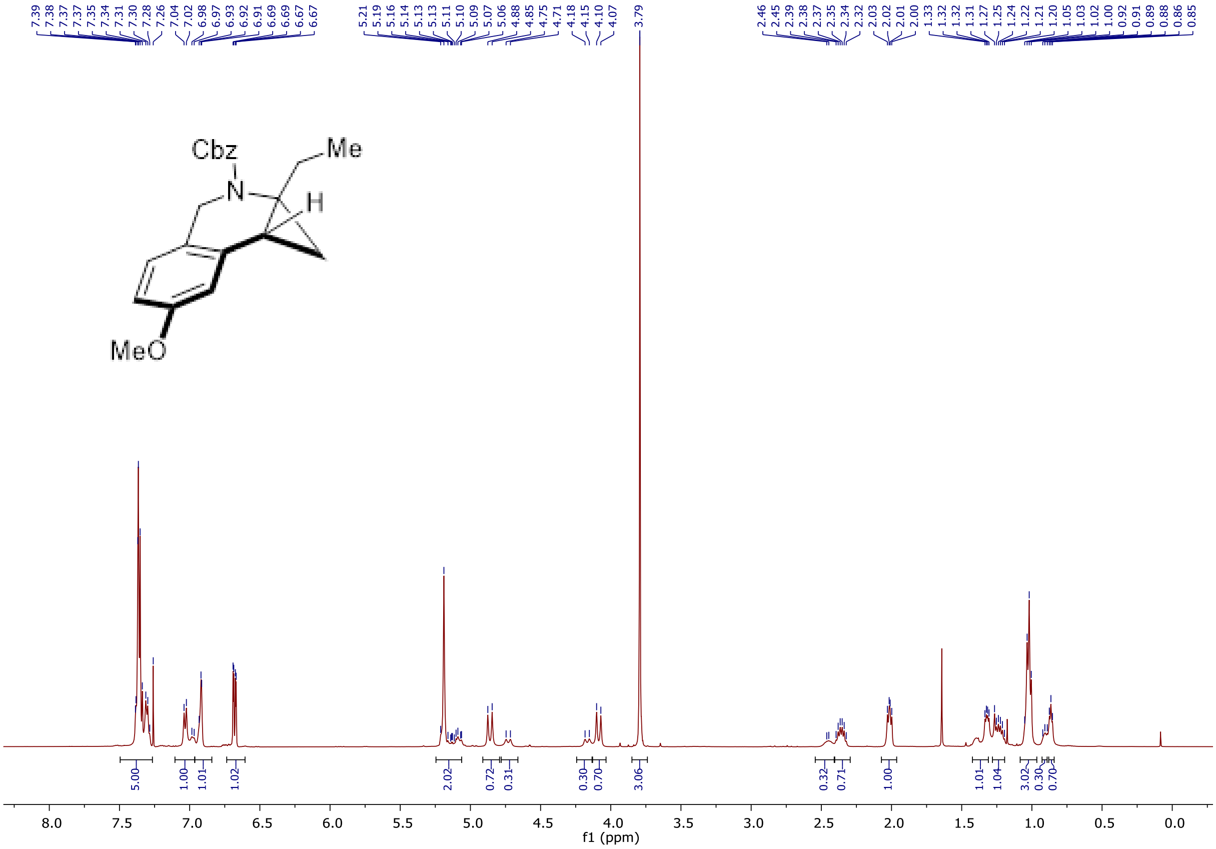

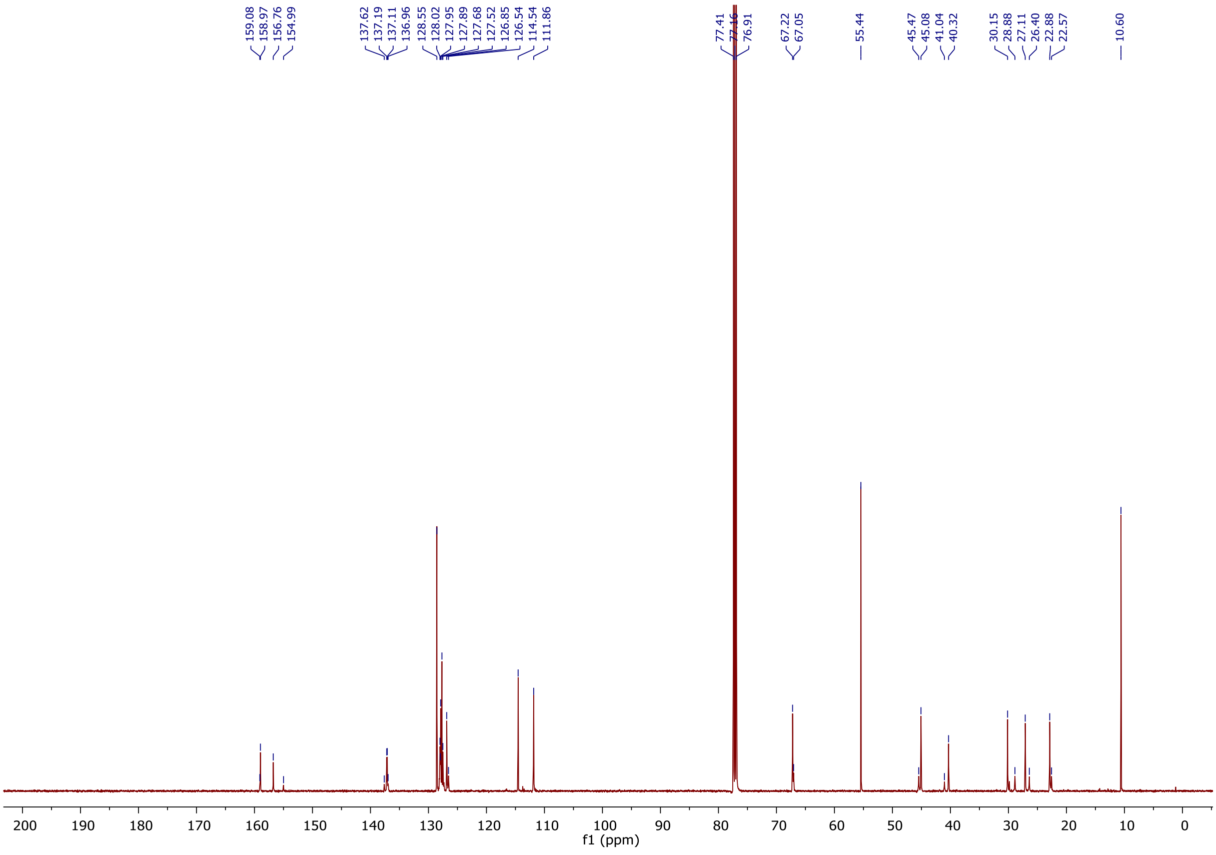

Methyl 2-(2-methylenebutyl)-4-(trifluoromethyl)benzoate

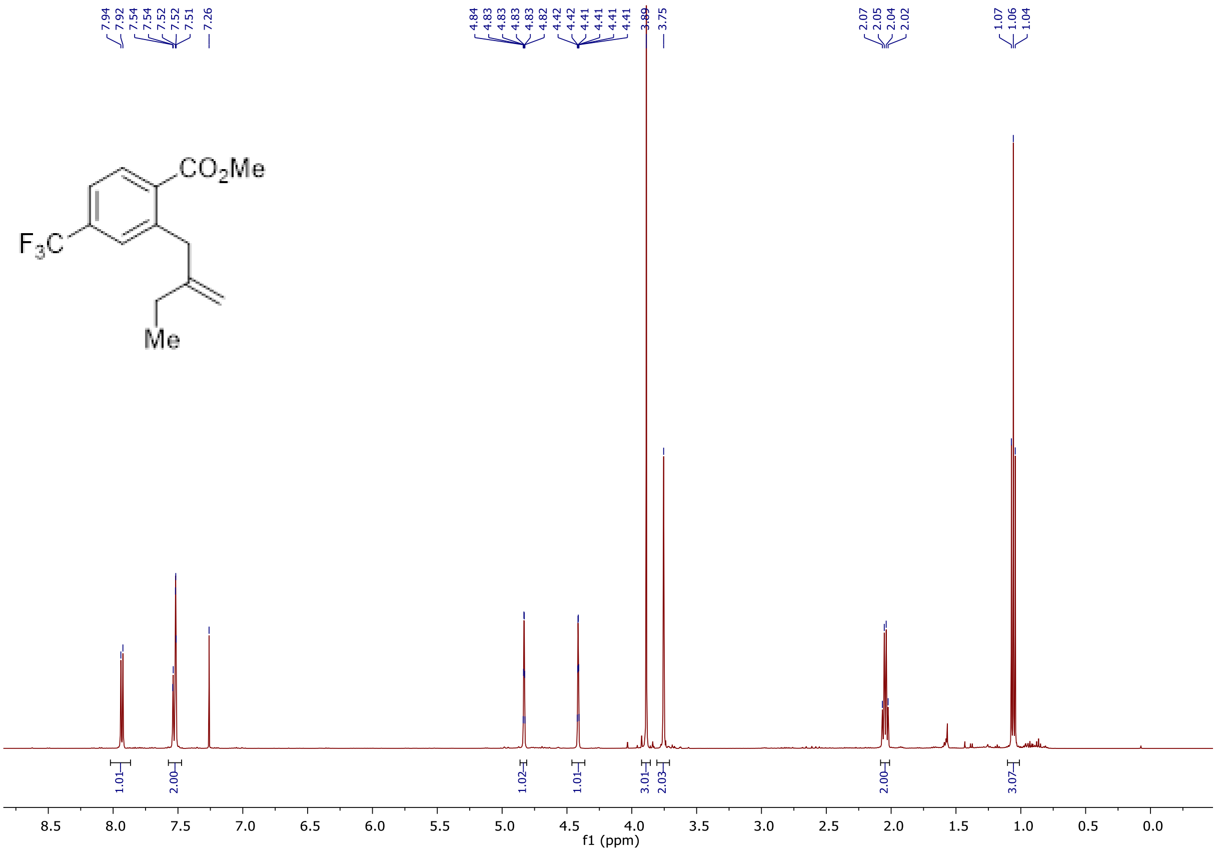

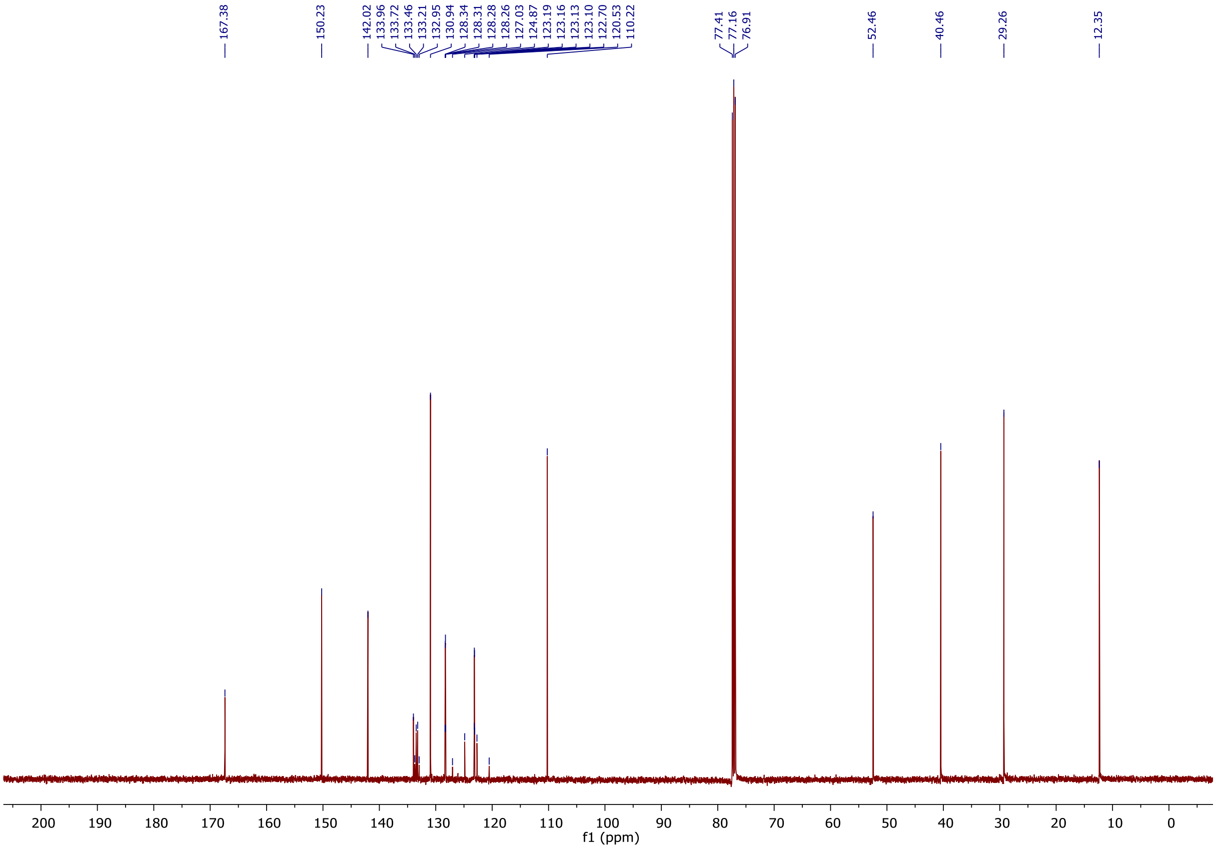

(2-(2-Methylenebutyl)-4-(trifluoromethyl)phenyl)methanol

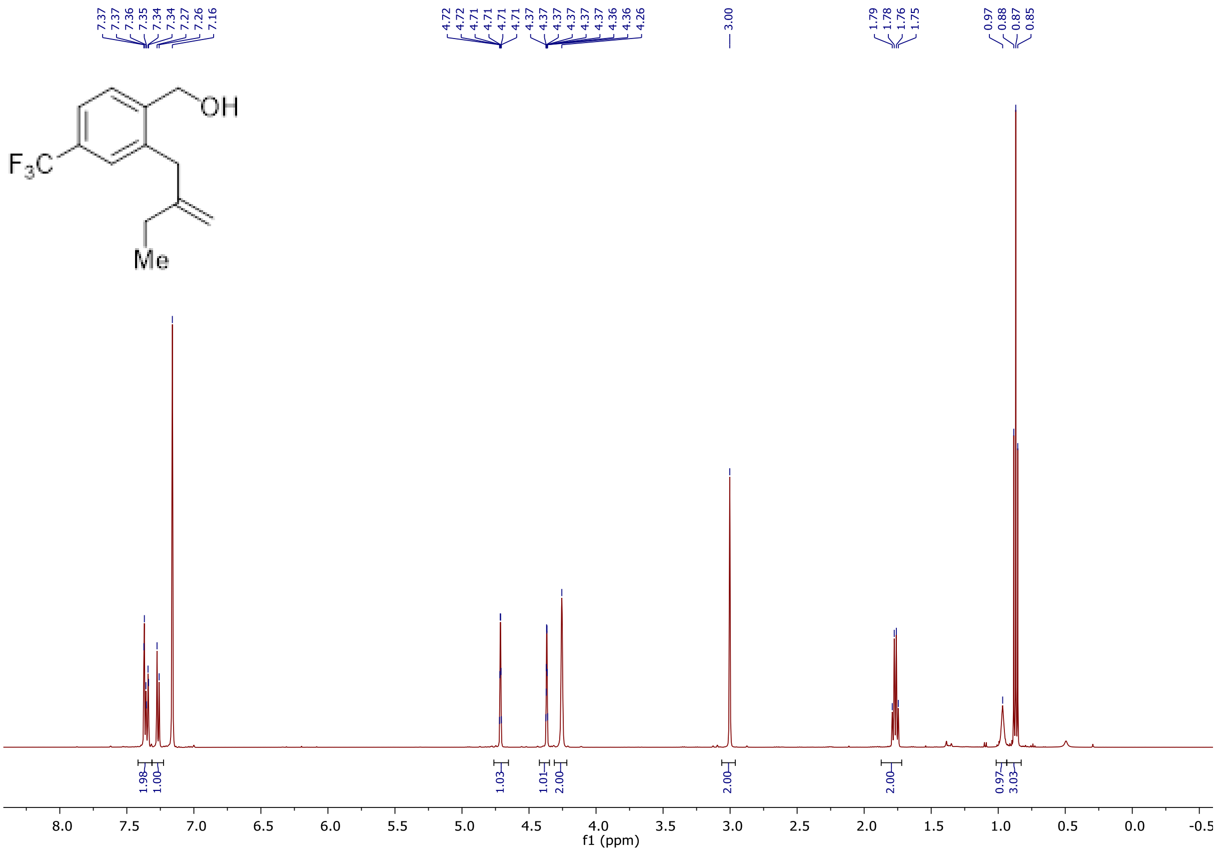

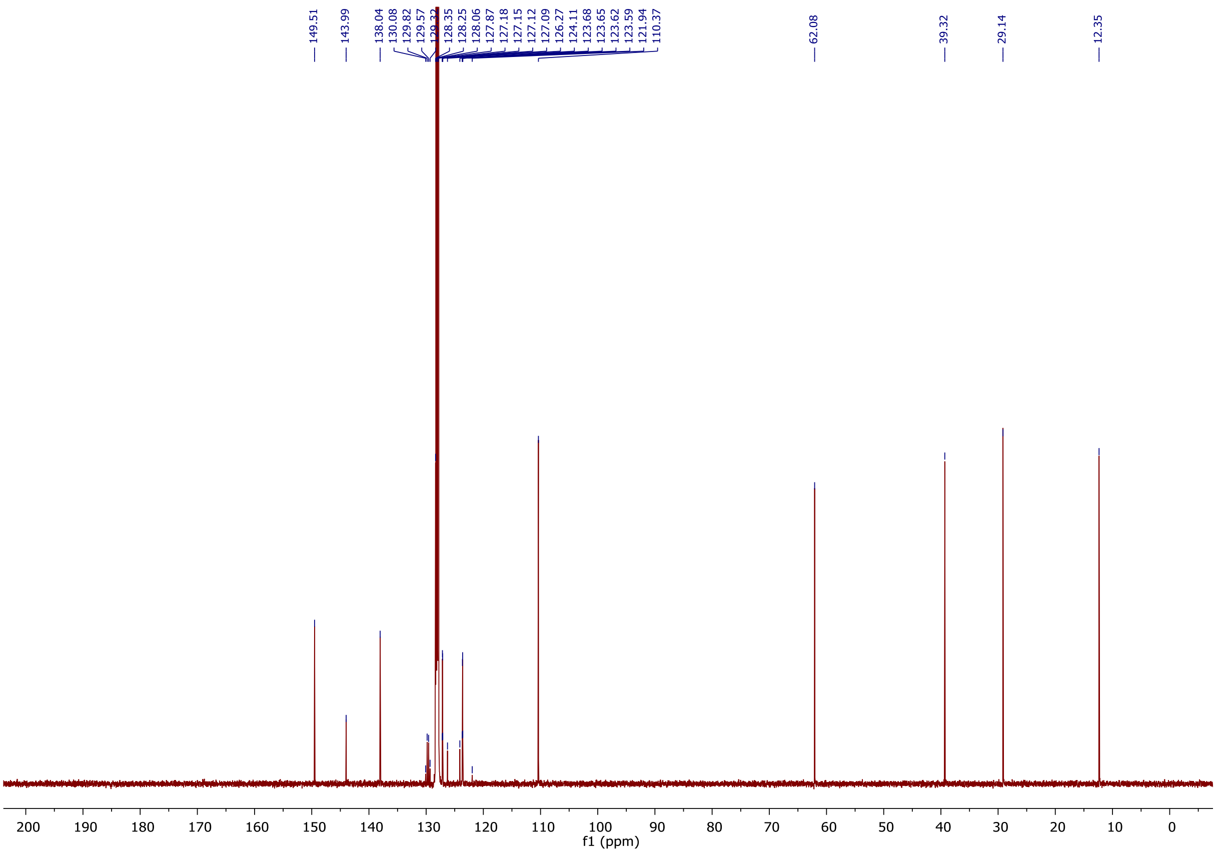

Benzyl (4- trifluoromethyl-2-(2-methylenebutyl)benzyl)((perfluorobenzoyl)oxy)carbamate (1s)

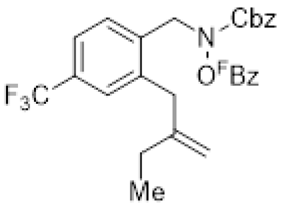

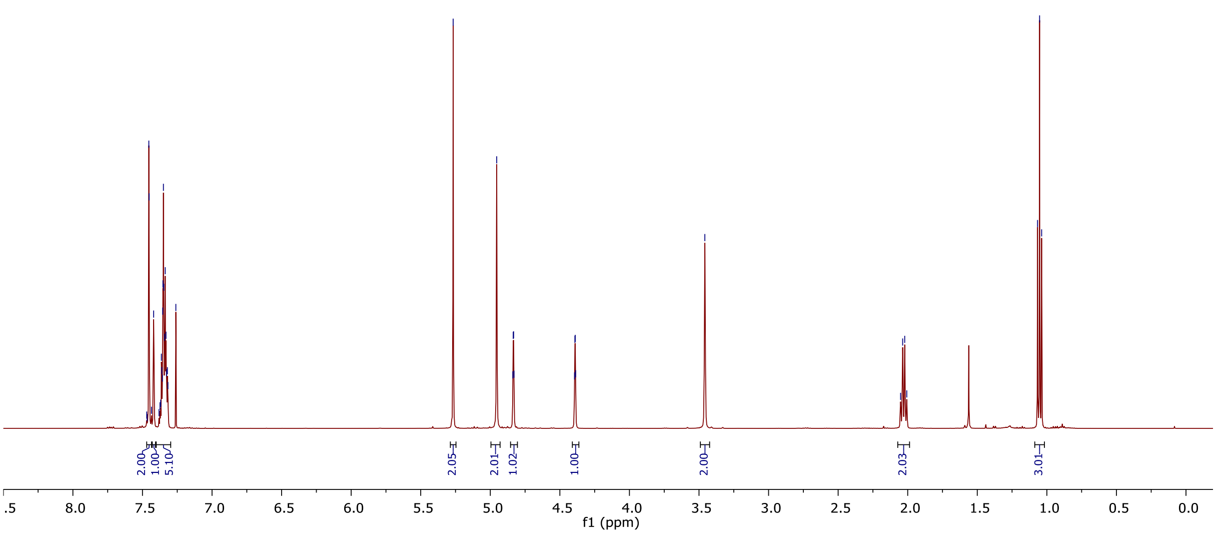

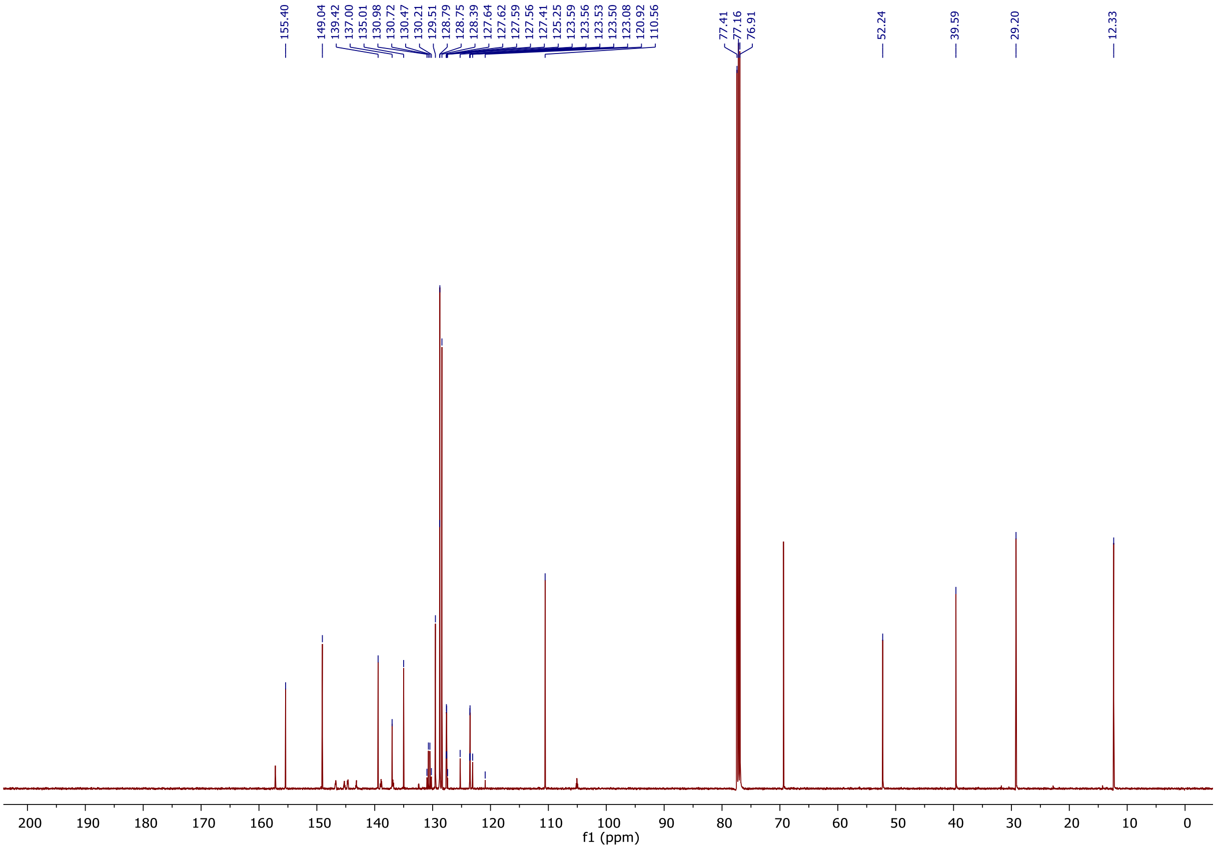

Benzyl 1a-ethyl-6-(trifluoromethyl)-1,1a,3,7b-tetrahydro-2*H*-cyclopropa[*c*]isoquinoline-2-carboxylate (2s)

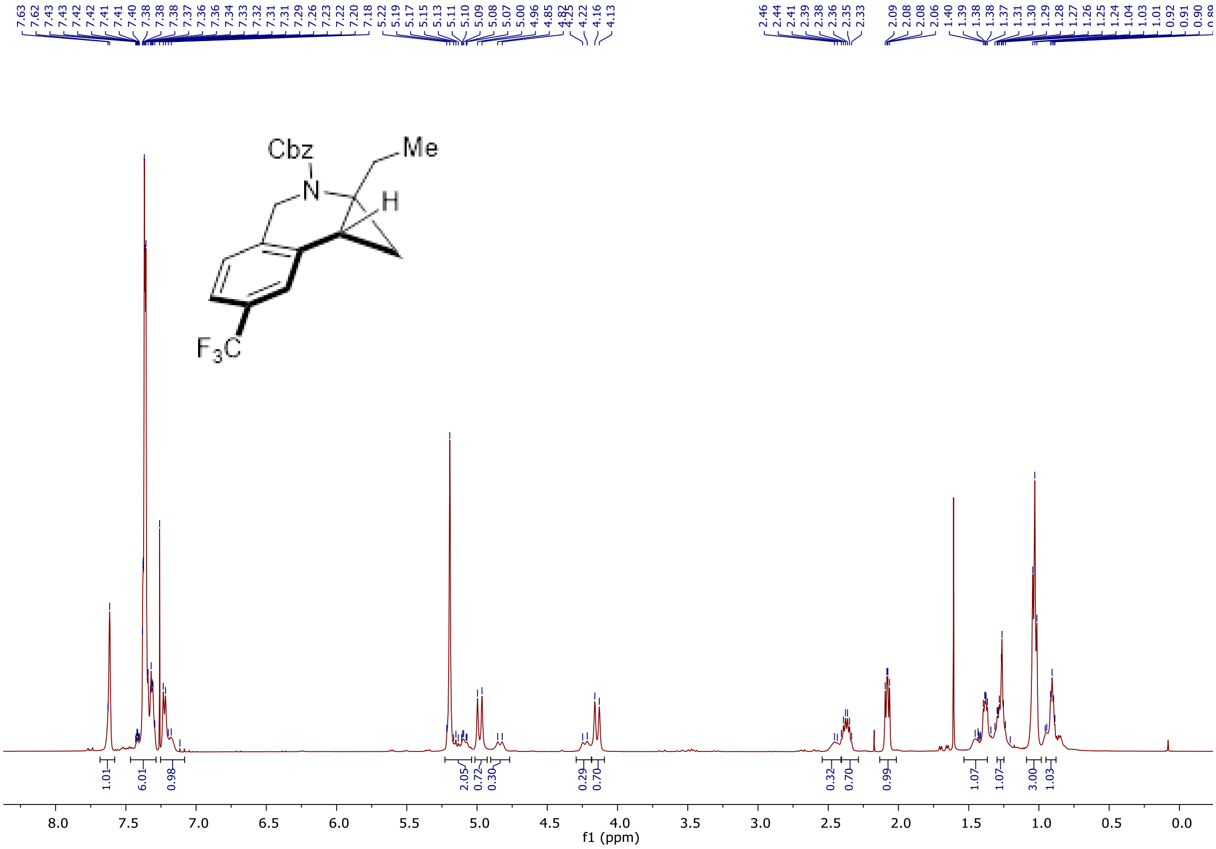

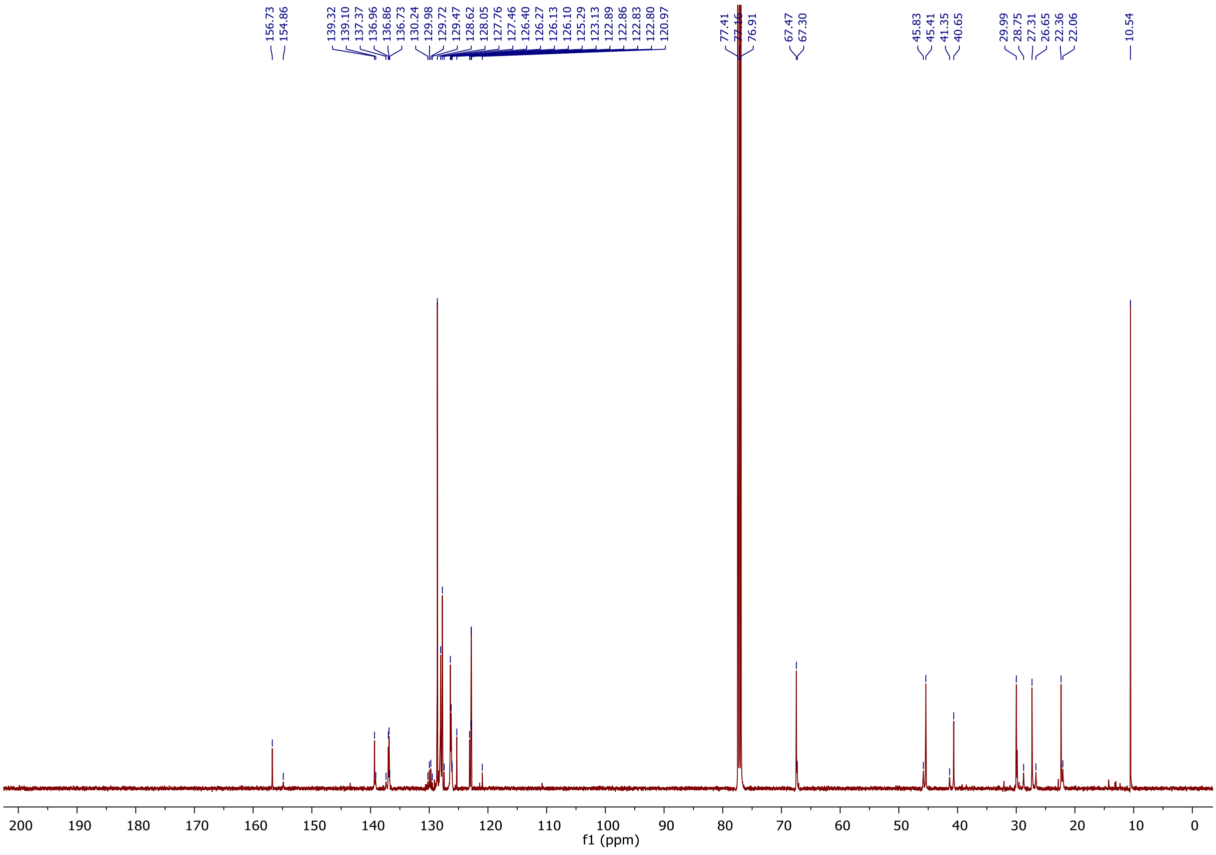

Methyl 3-(2-methylenebutyl)-2-naphthoate

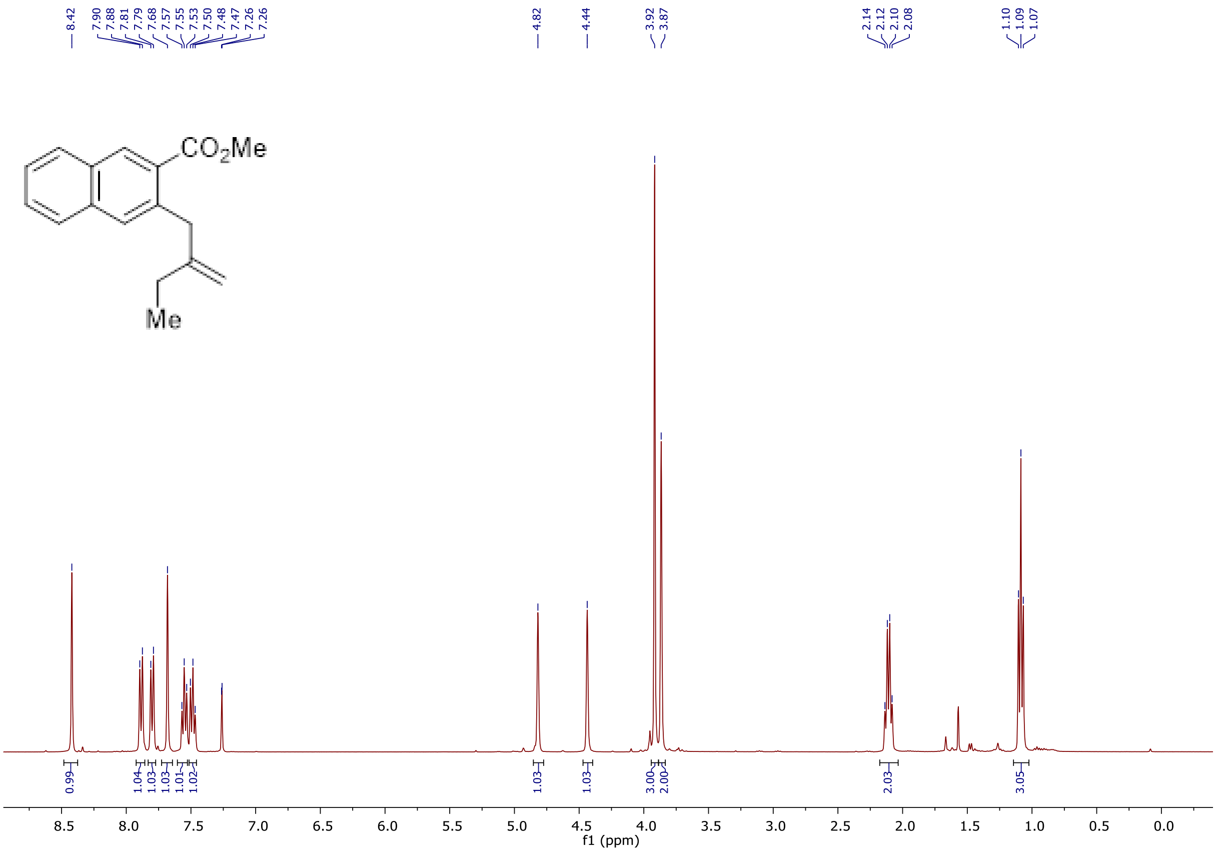

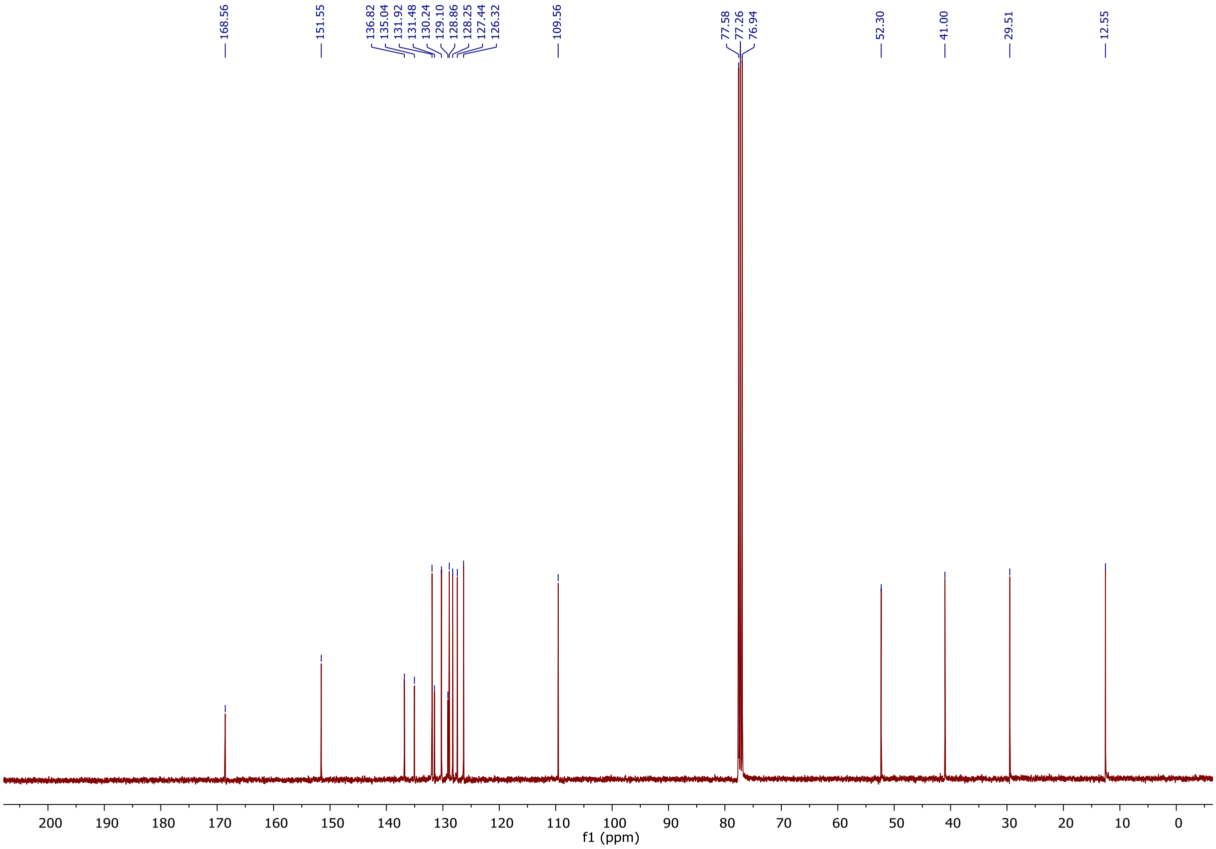

(3-(2-Methylenebutyl)naphthalen-2-yl)methanol

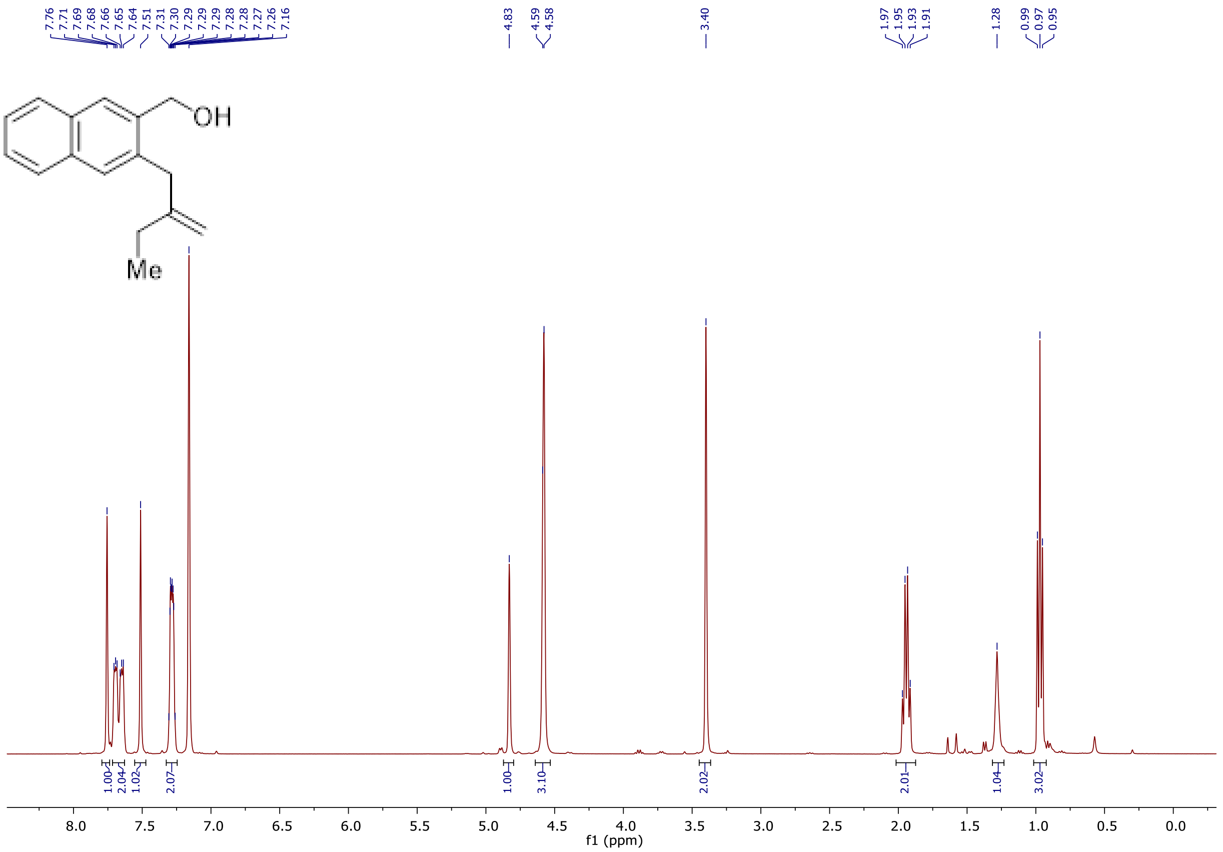

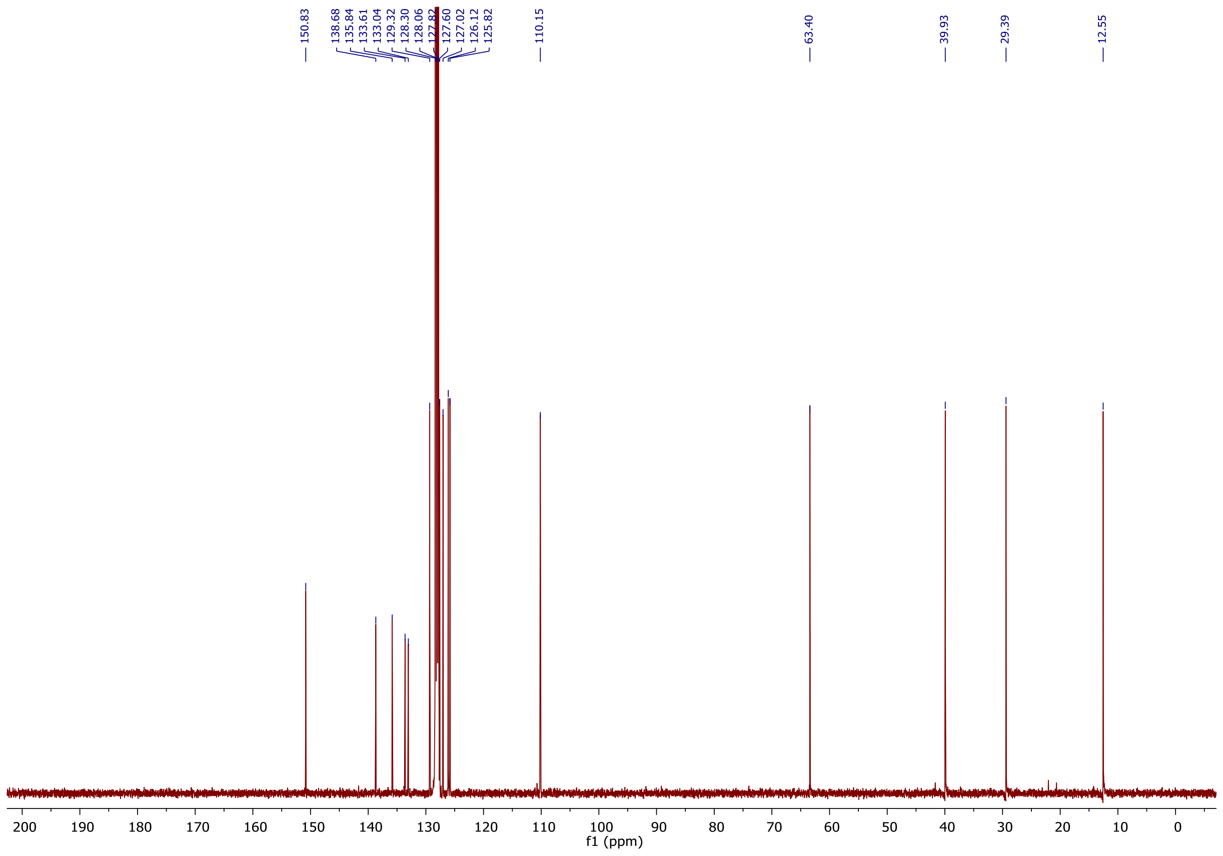

Benzyl ((3-(2-methylenebutyl)naphthalen-2-yl)methyl)((perfluorobenzoyl)oxy)carbamate (1t)

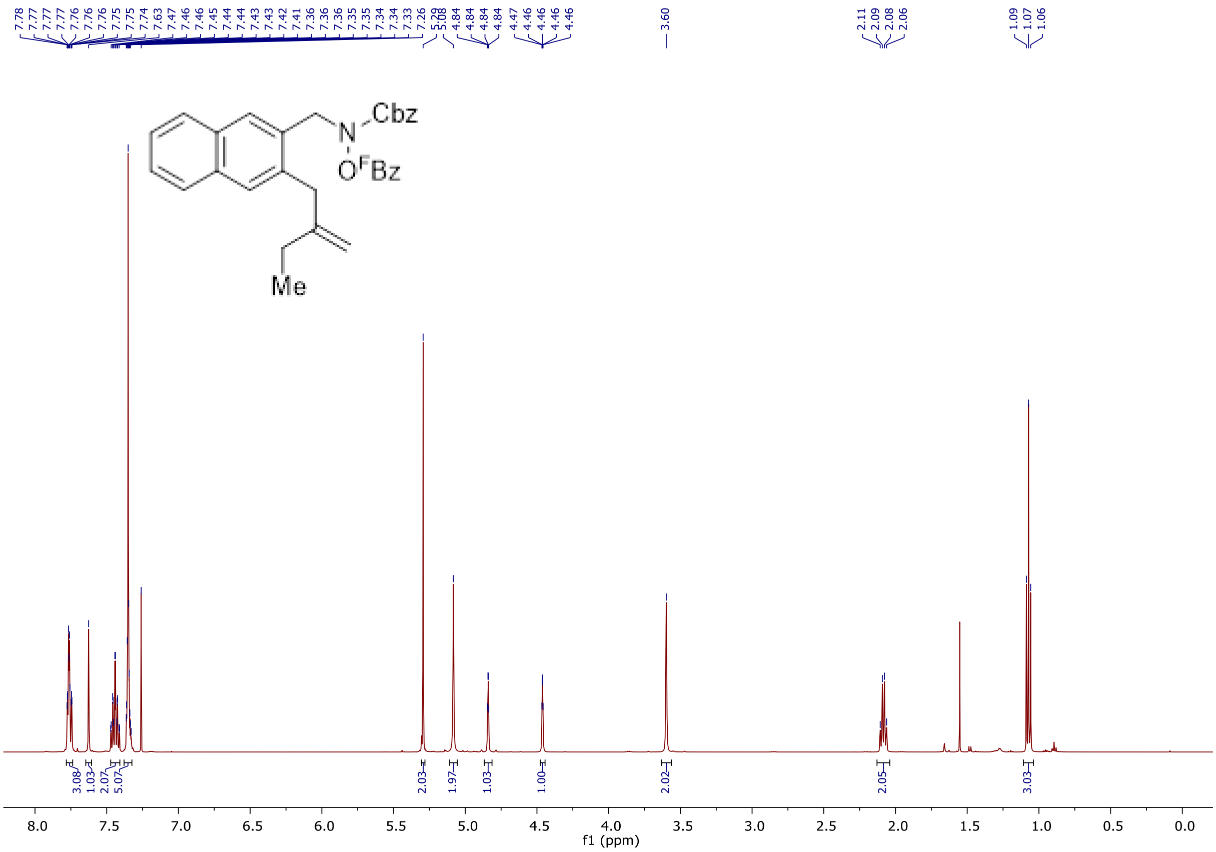

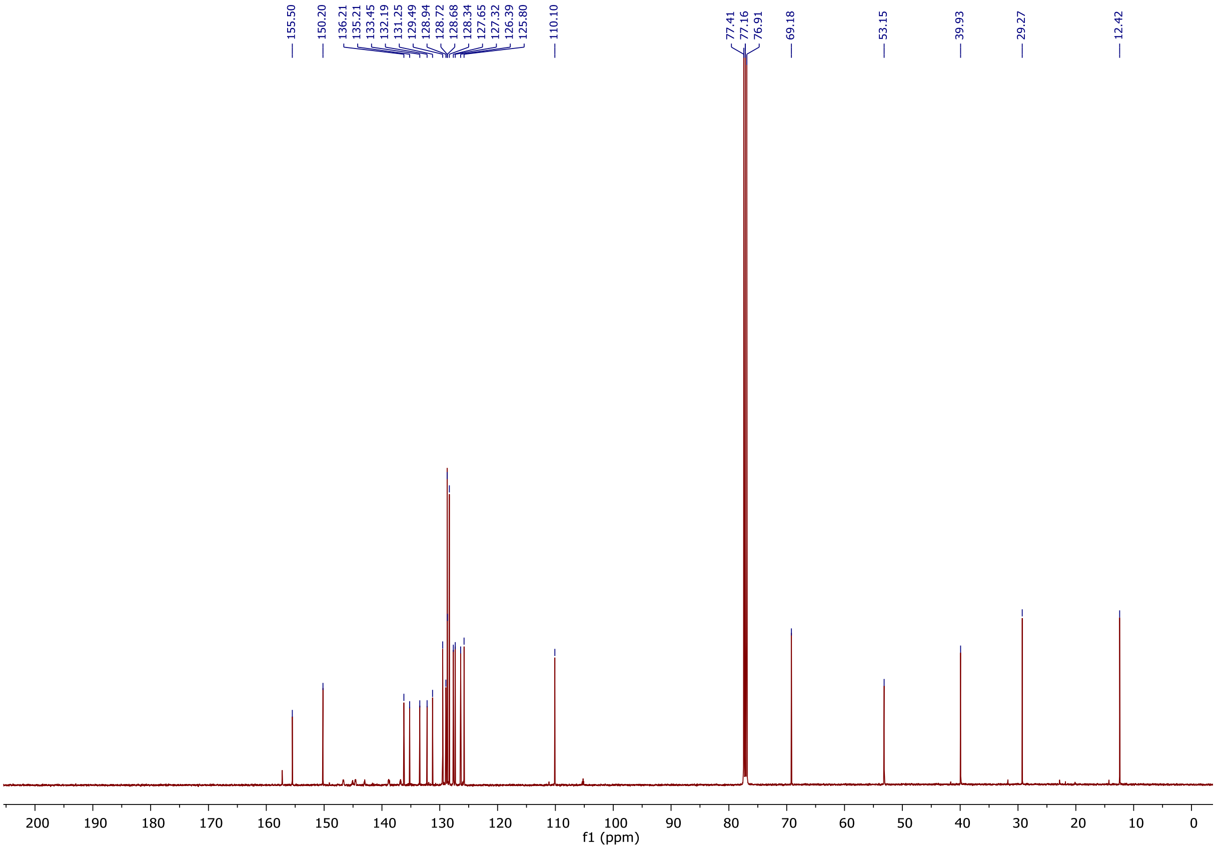

Benzyl 1a-ethyl-1,1a,3,9b-tetrahydro-2*H*-benzo[*g*]cyclopropa[*c*]isoquinoline-2-carboxylate (2t)

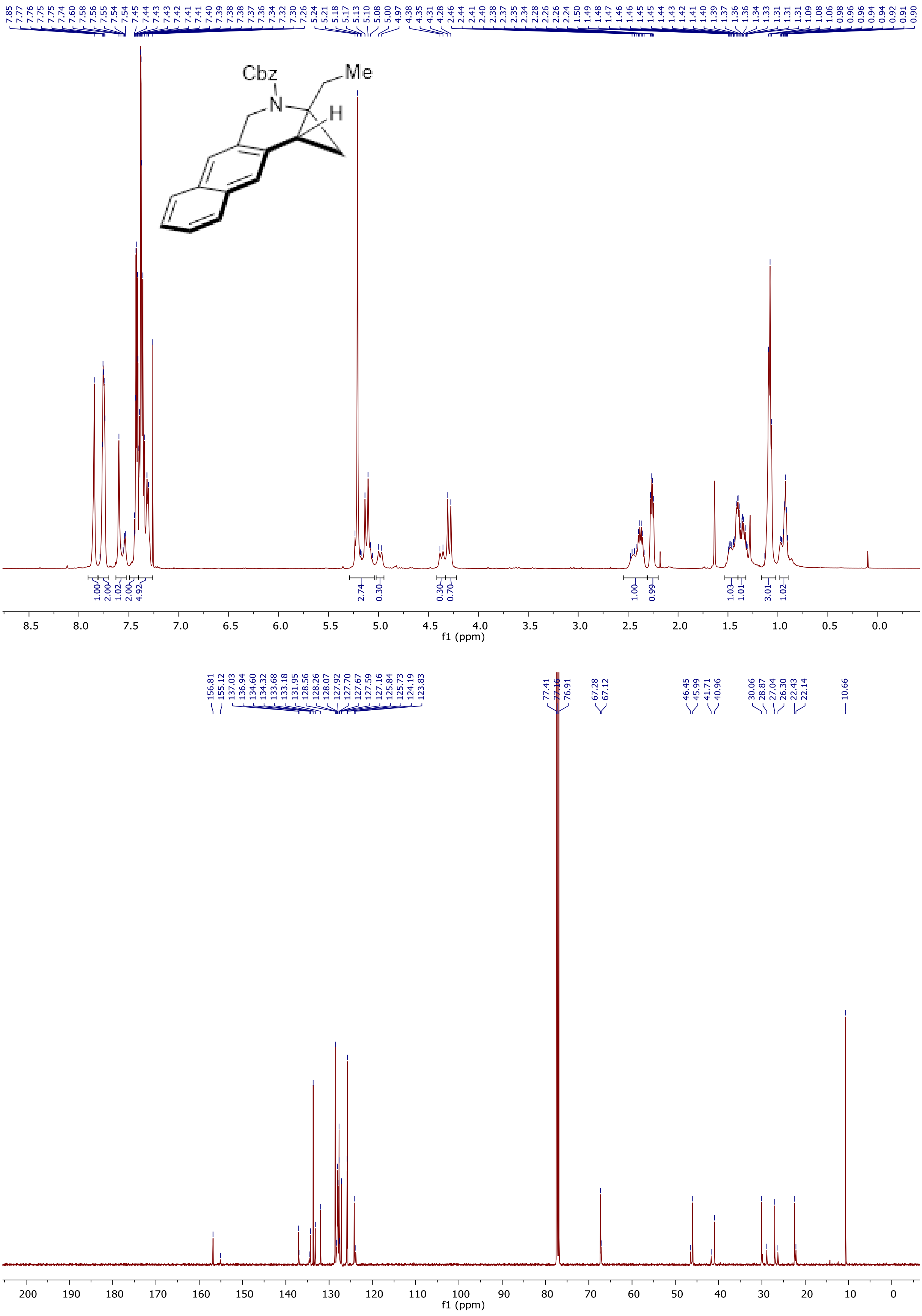

1-(2-(2-Methylenebutyl)phenyl)ethan-1-ol

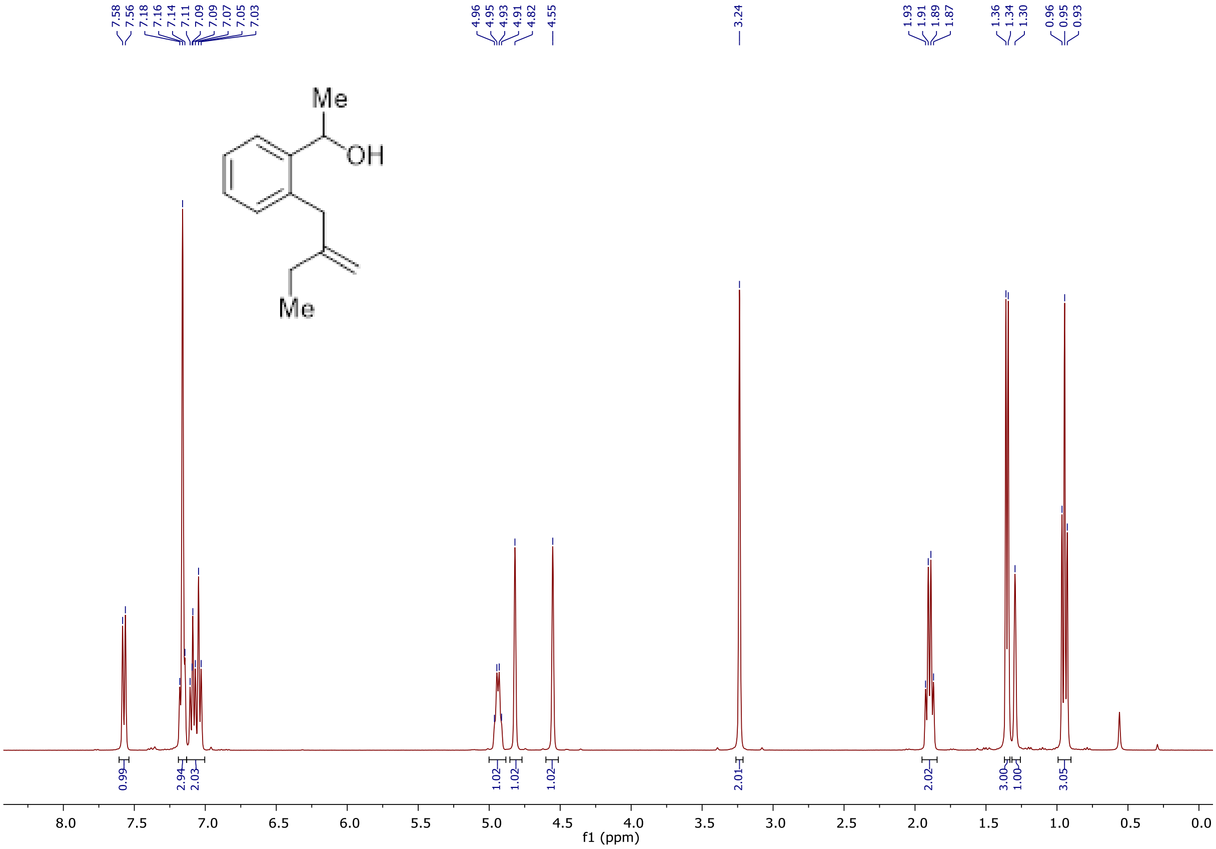

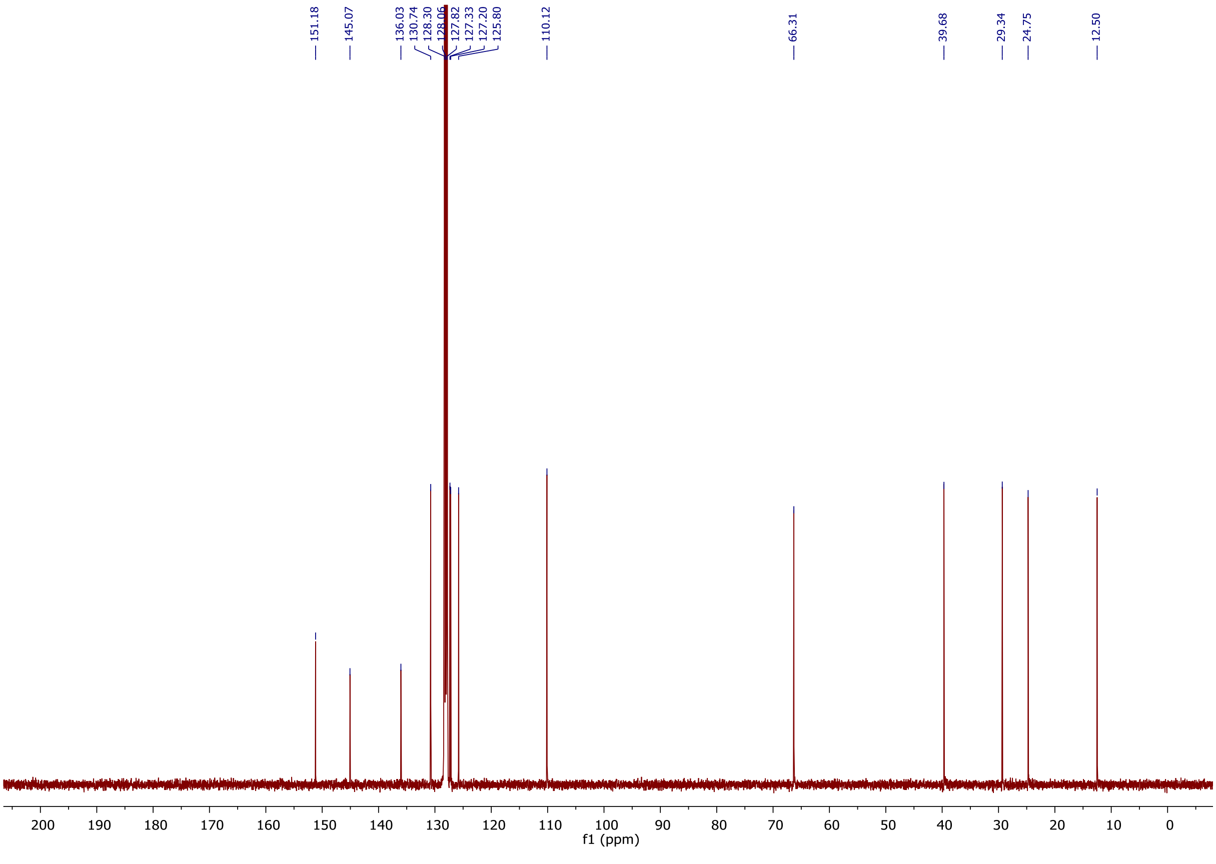

Benzyl (1-(2-(2-methylenebutyl)phenyl)ethyl)((perfluorobenzoyl)oxy)carbamate (1u)

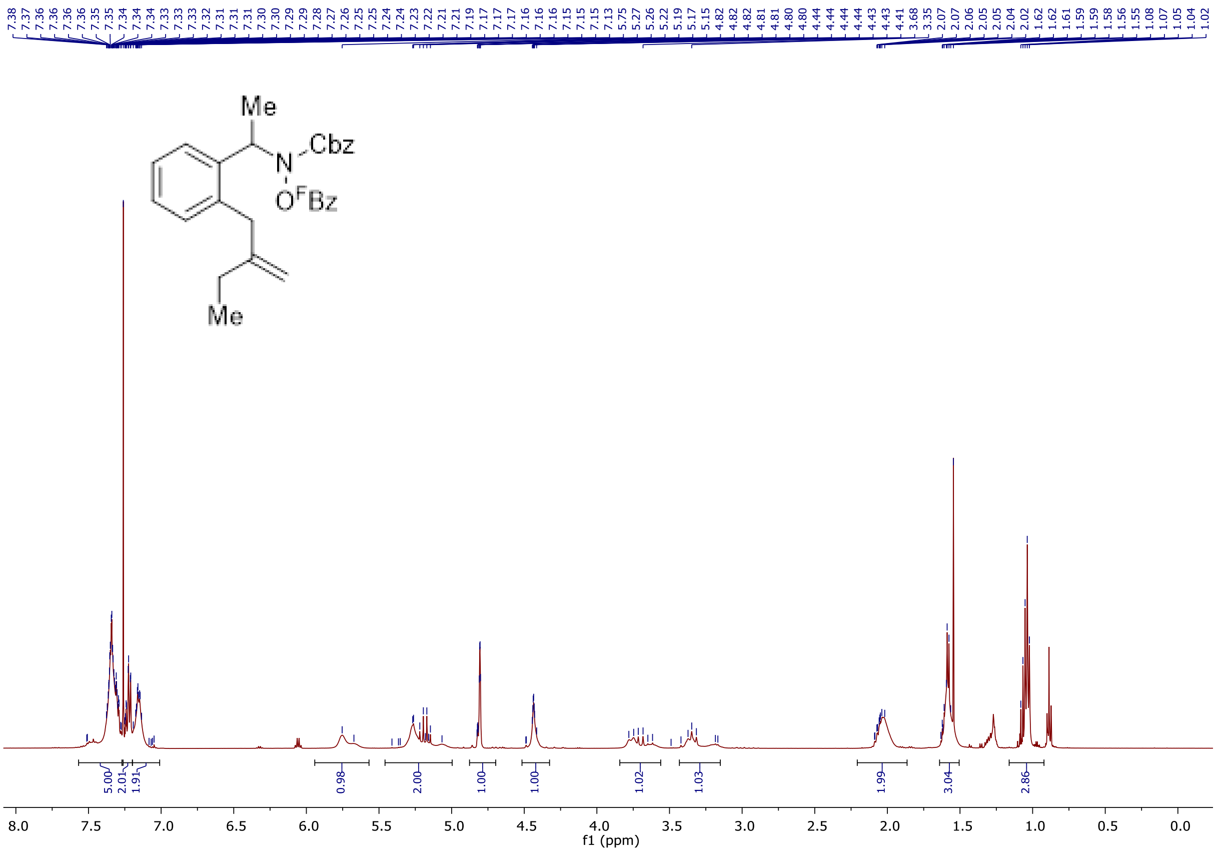

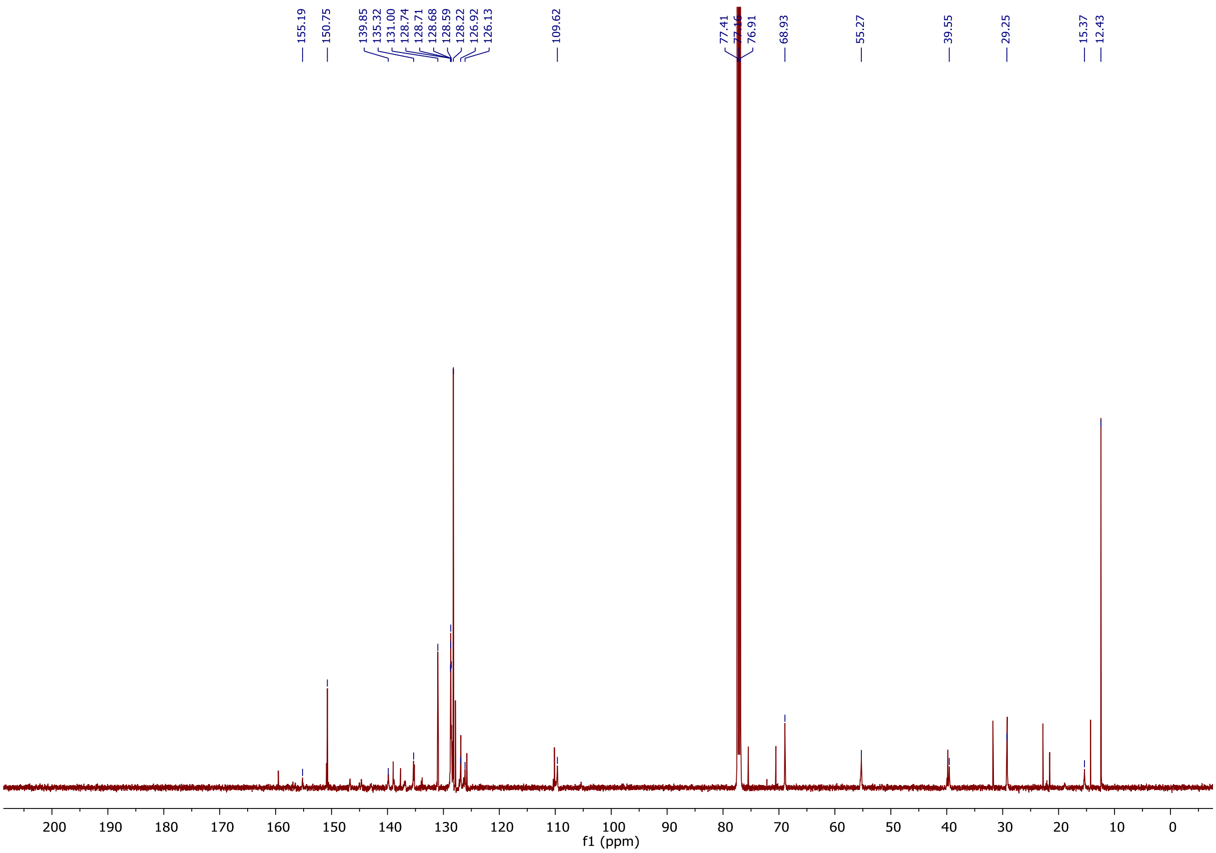

Benzyl 1a-ethyl-3-methyl-1,1a,3,7b-tetrahydro-2*H*-cyclopropa[*c*]isoquinoline-2-carboxylate (2u)

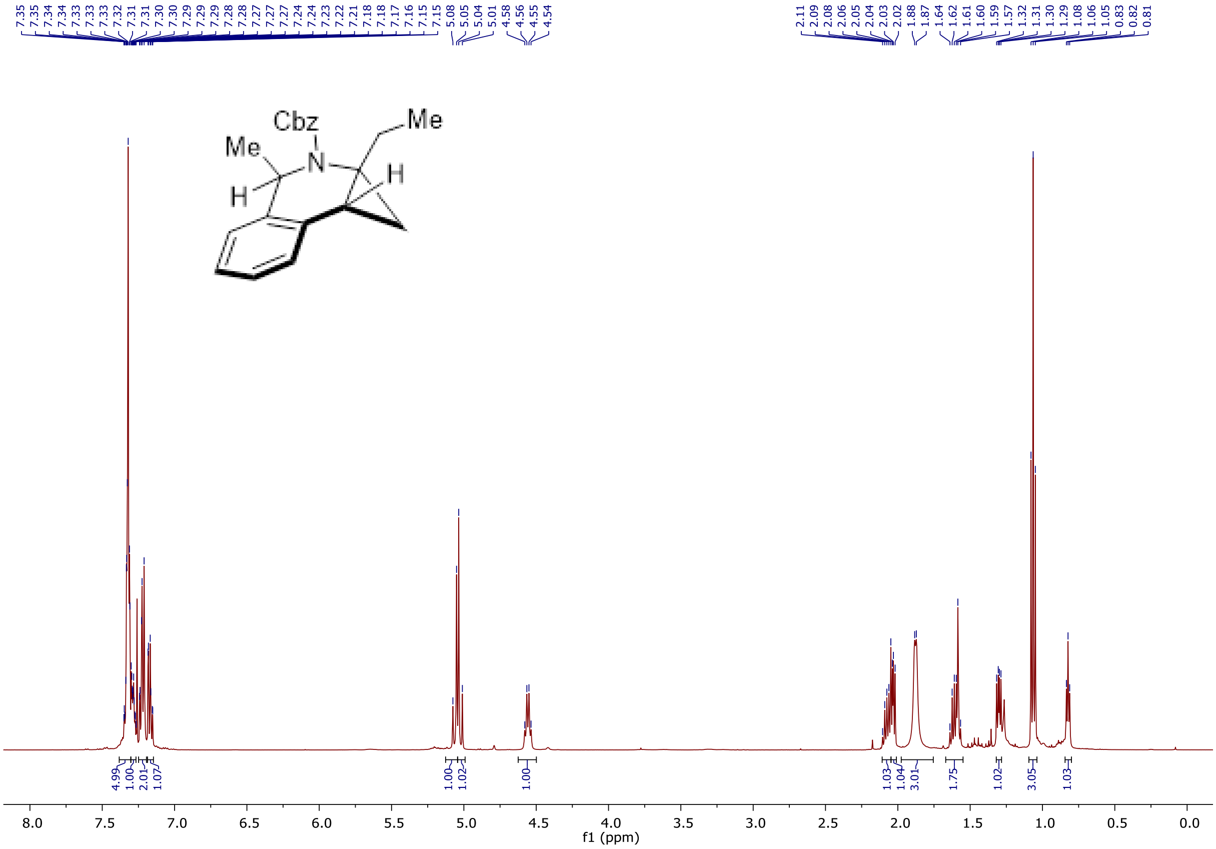

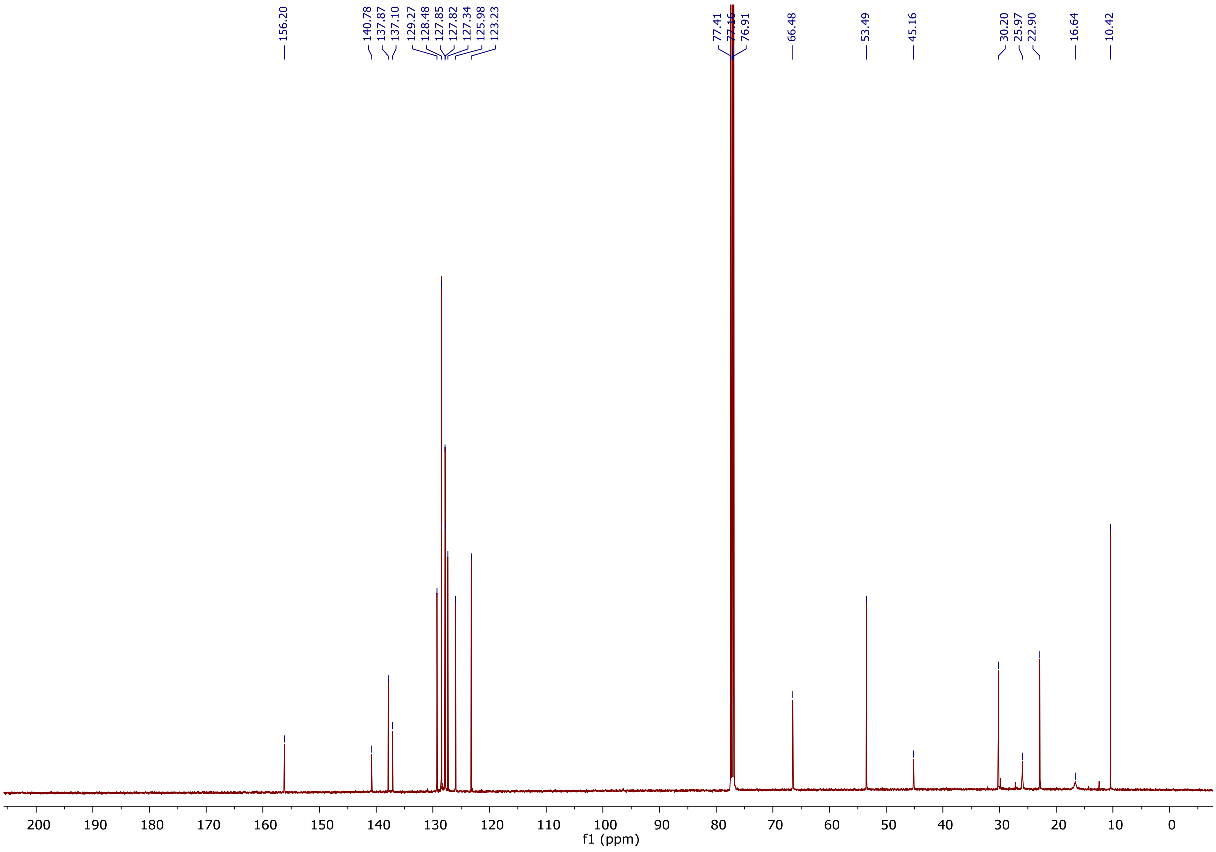

3-(2-Hydroxy-1-(trimethylsilyl)propan-2-yl)dihydrofuran-2(3*H*)-one

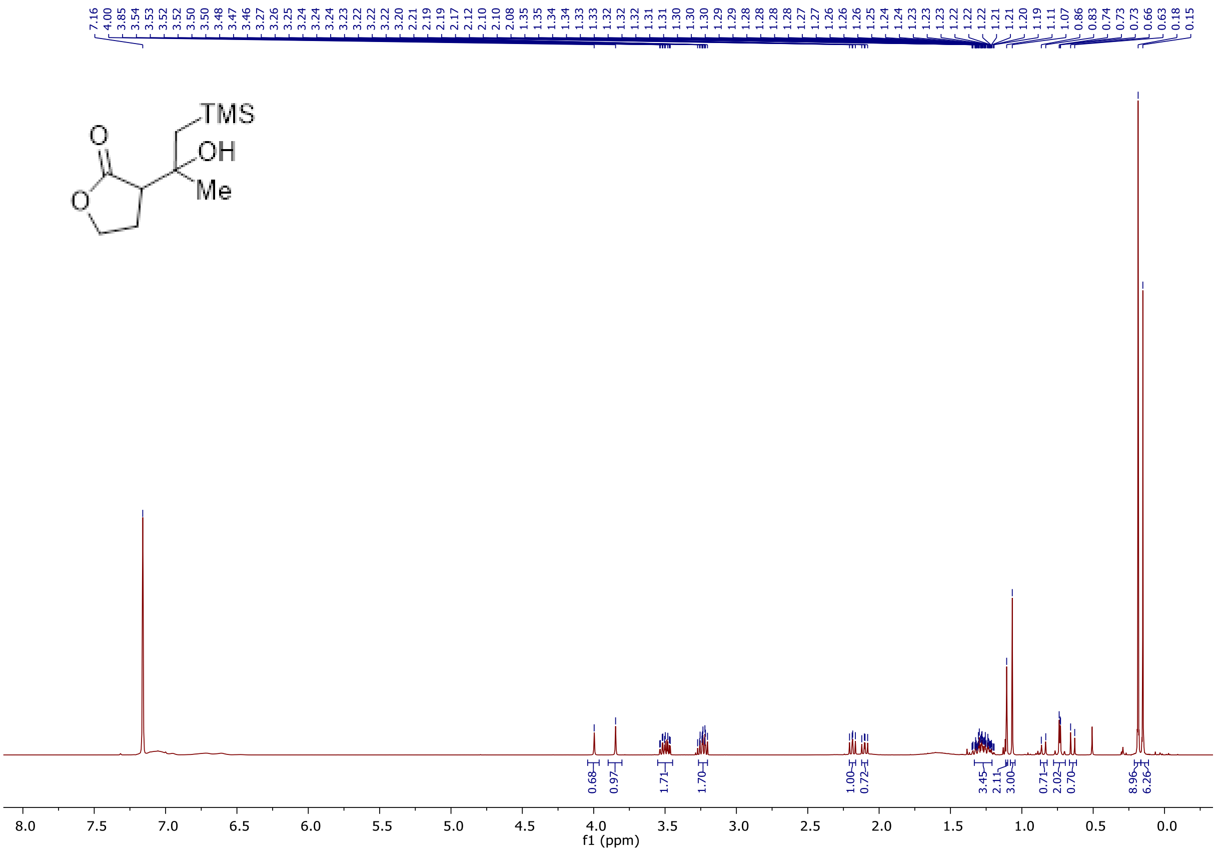

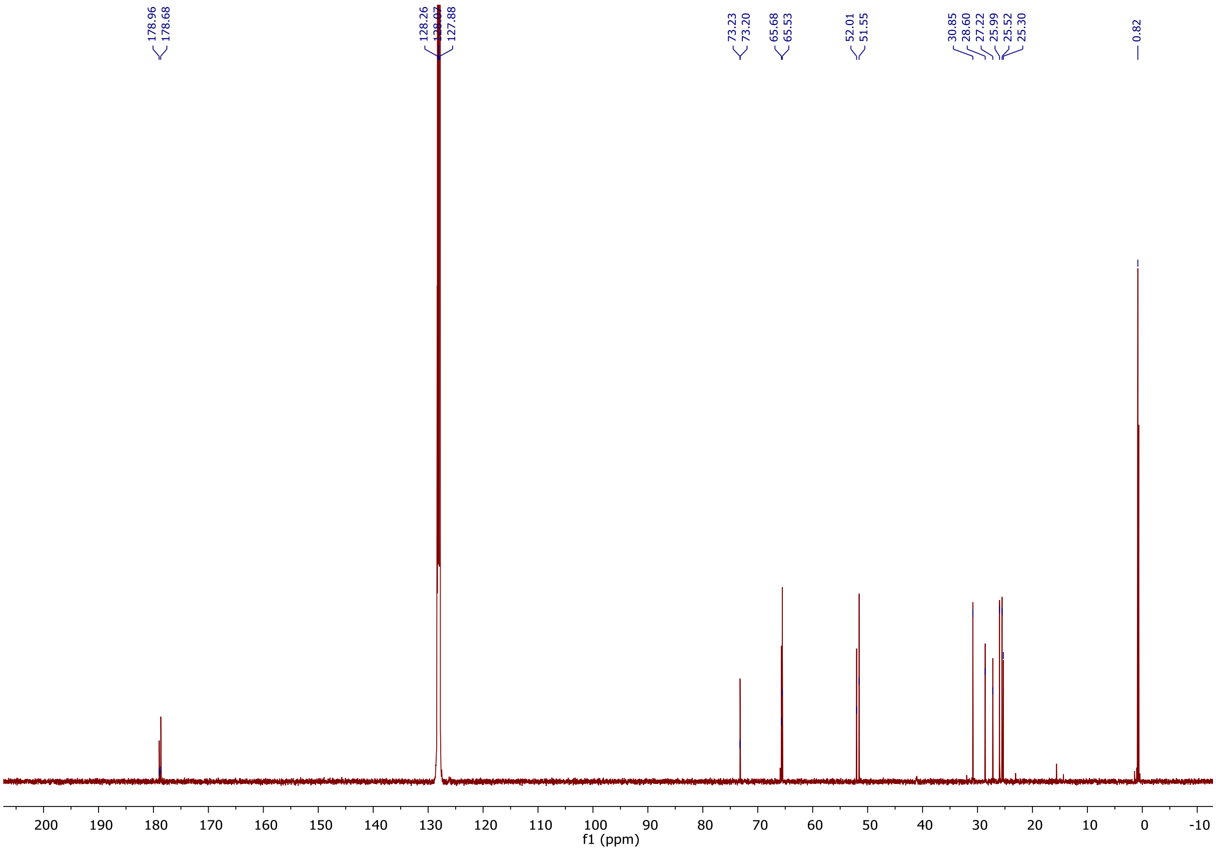

3-(Prop-1-en-2-yl)dihydrofuran-2(3*H*)-one

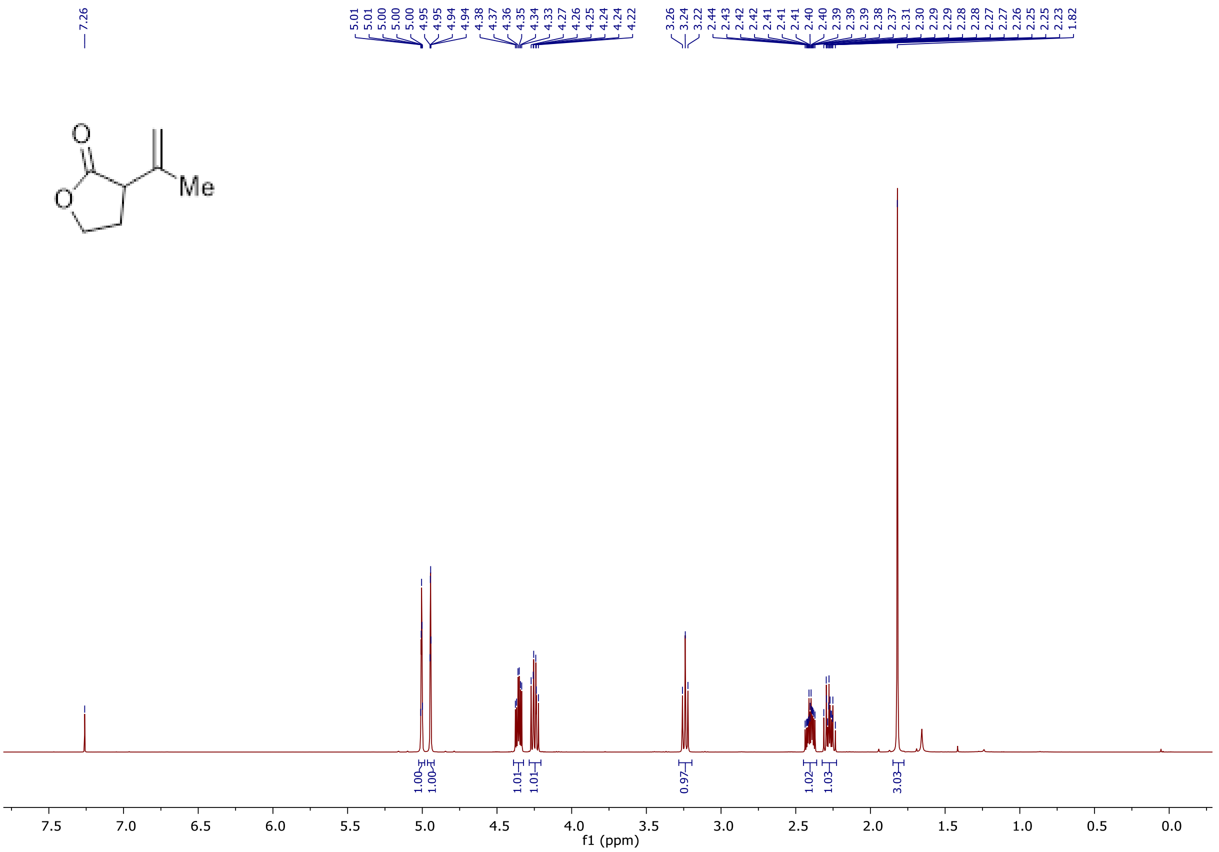

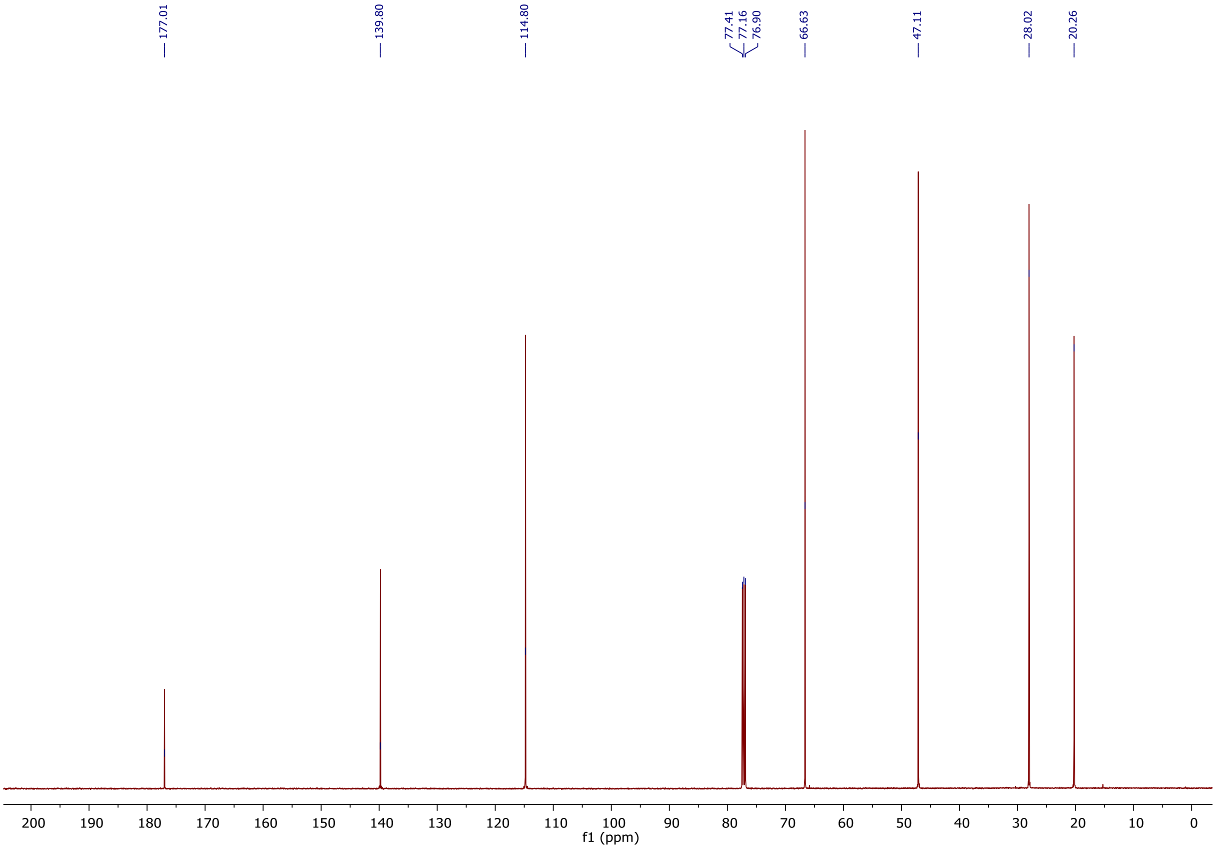

Benzyl (3-acetyl-4-methylpent-4-en-1-yl)((perfluorobenzoyl)oxy)carbamate (3a)

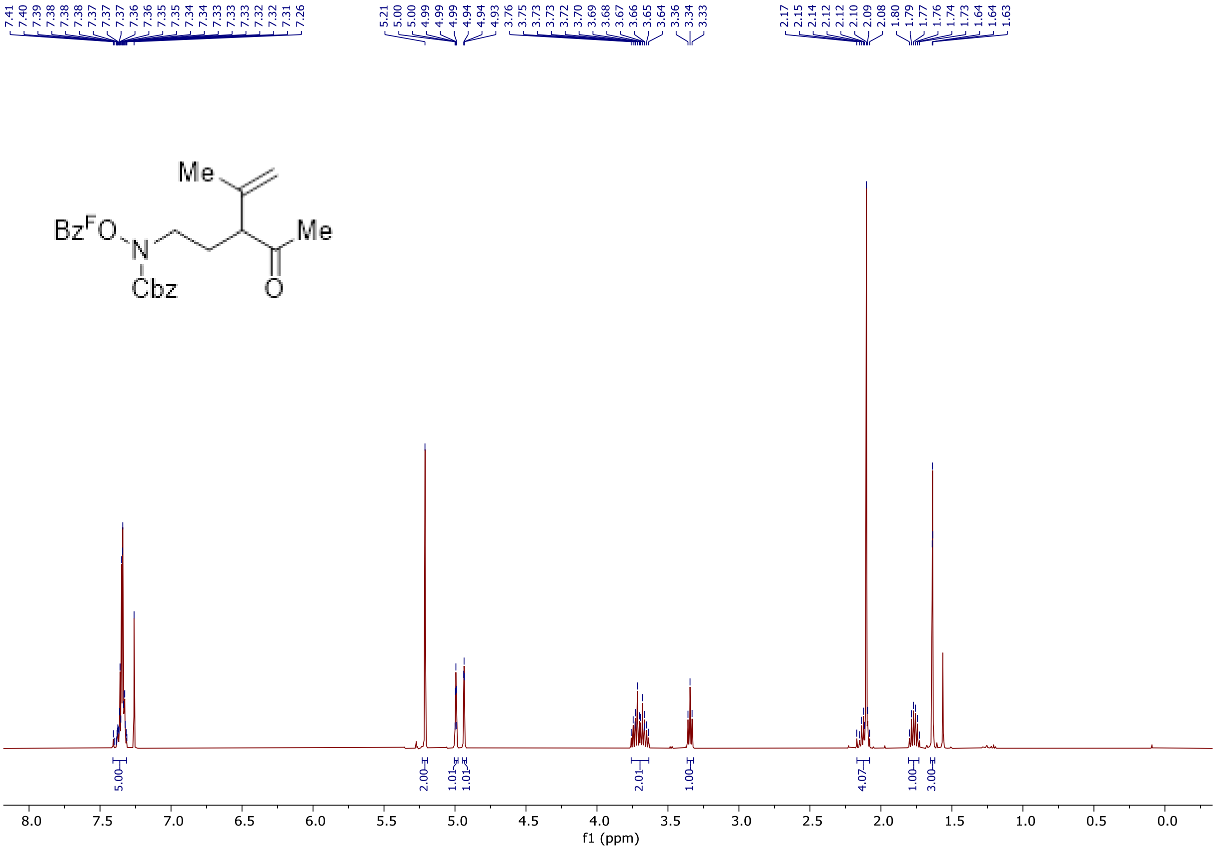

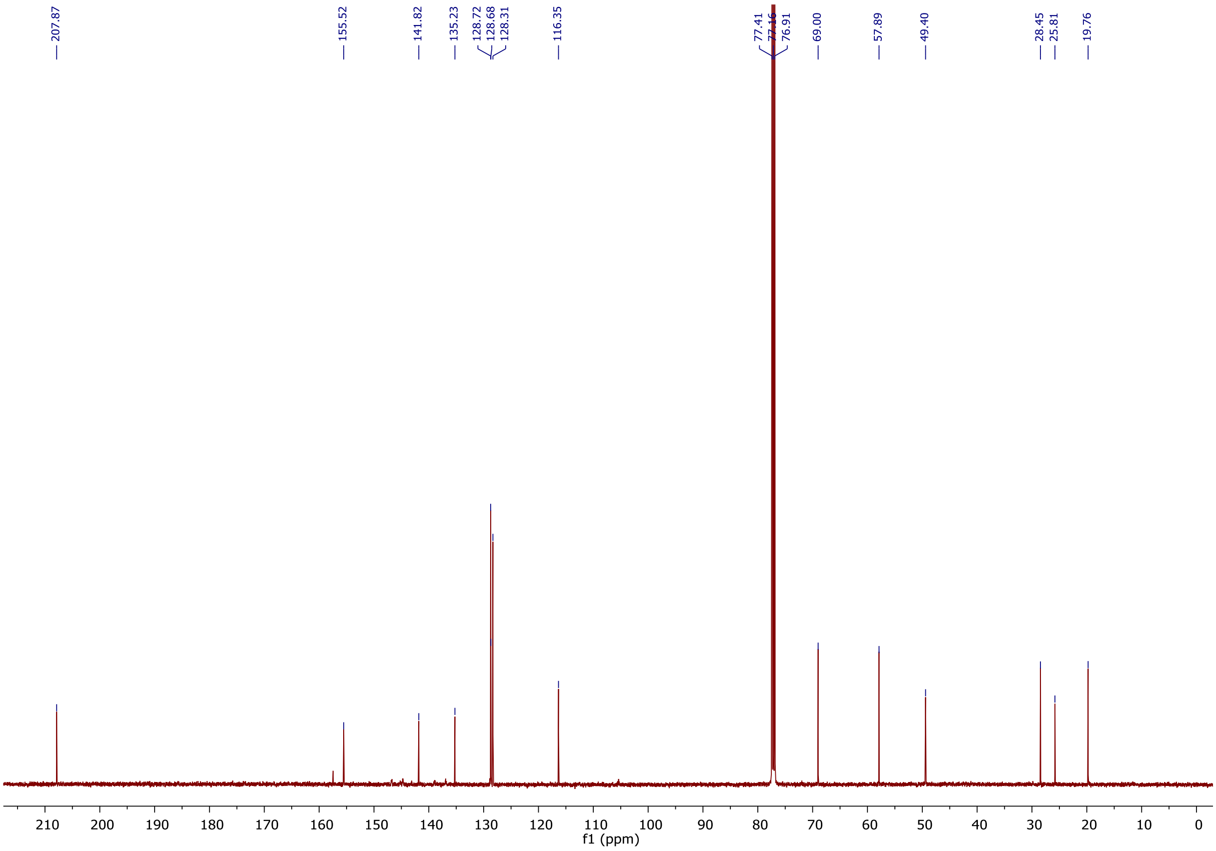

Benzyl 4-acetyl-6-methyl-3,4-dihydropyridine-1(2*H*)-carboxylate (4a-C2-C3)

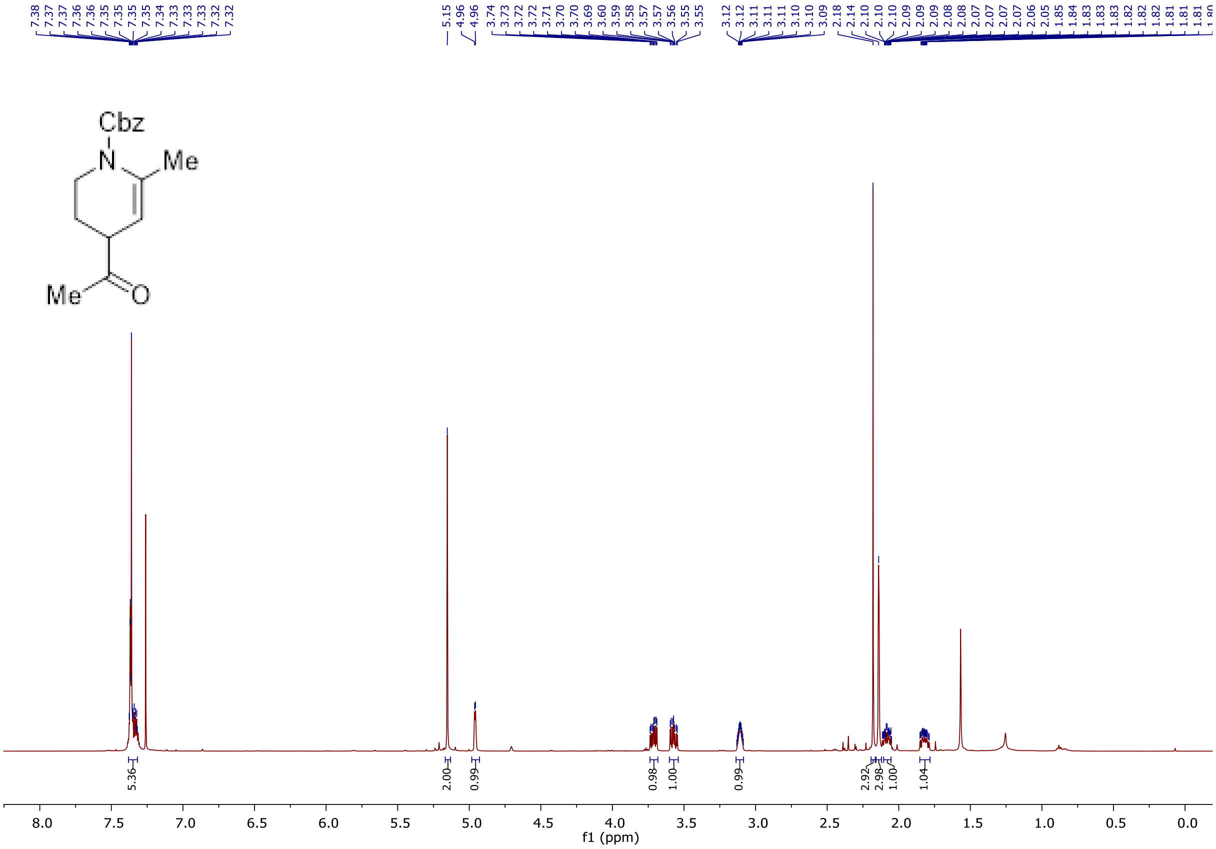

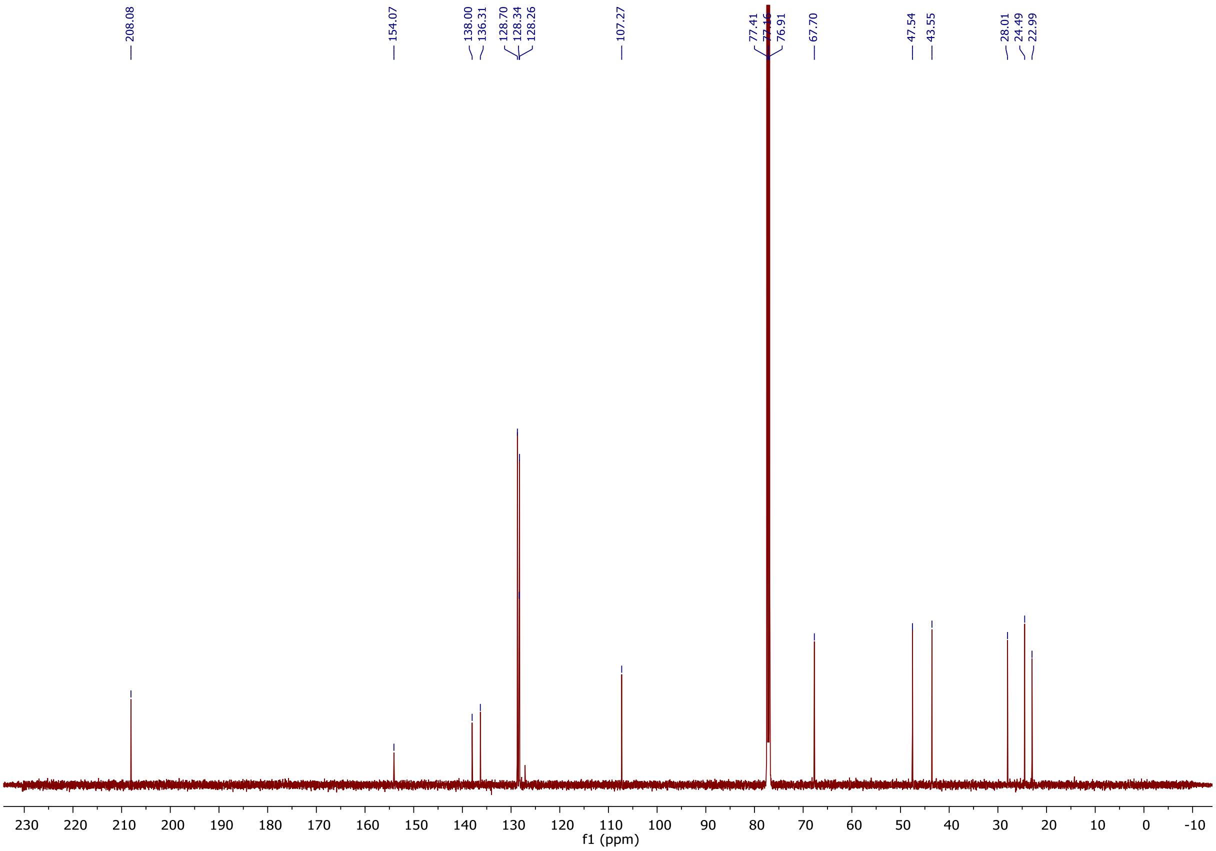

Benzyl 4-acetyl-6-methyl-3,6-dihydropyridine-1(2*H*)-carboxylate (4a-C3-C4)

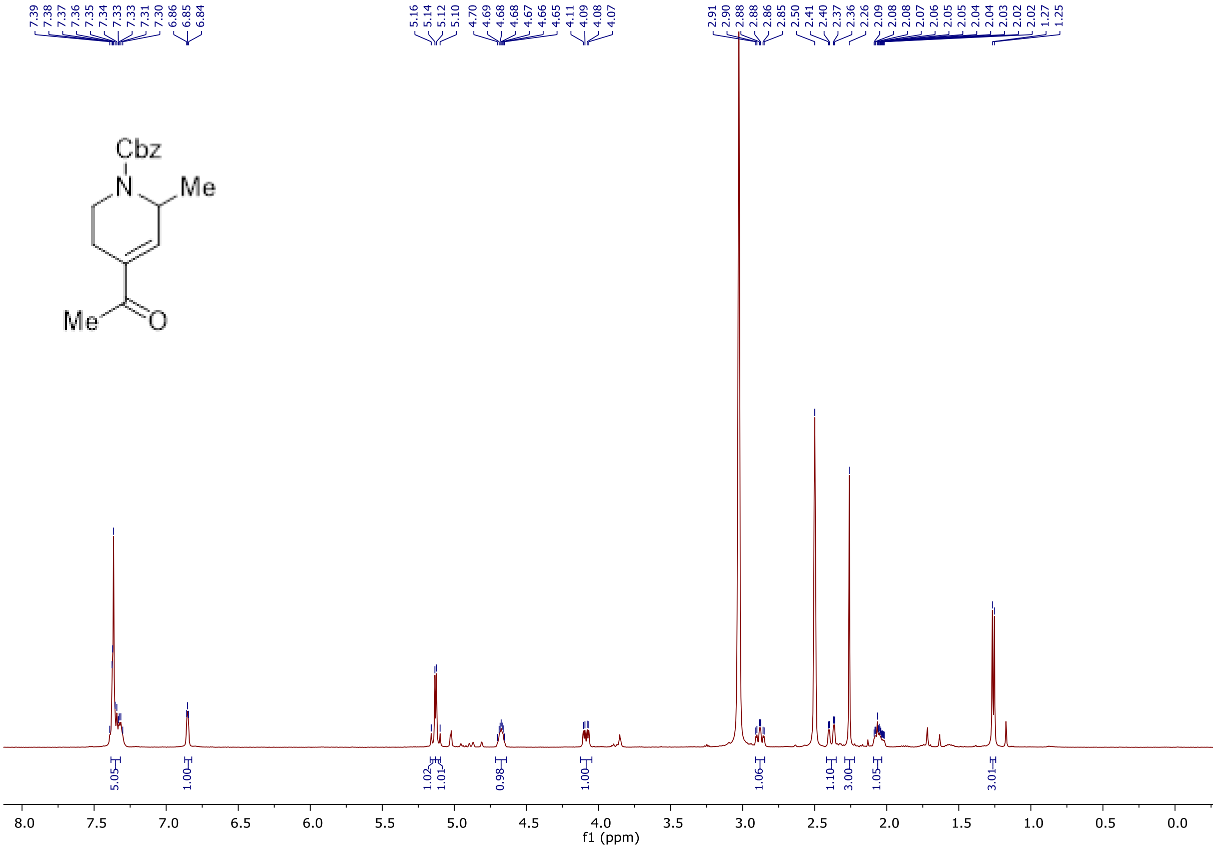

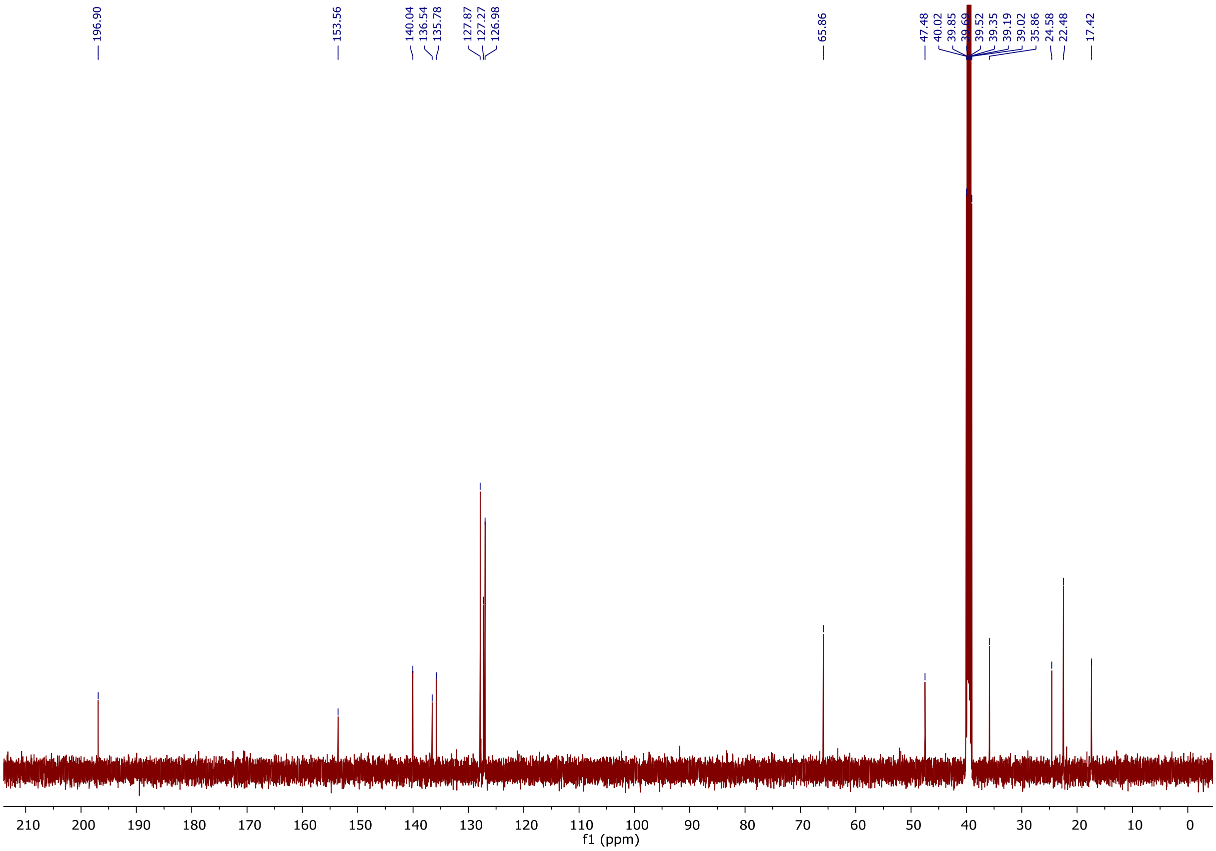

Methyl 2-(2-((*tert*-butyldimethylsilyl)oxy)ethyl)-3-methylbut-3-enoate

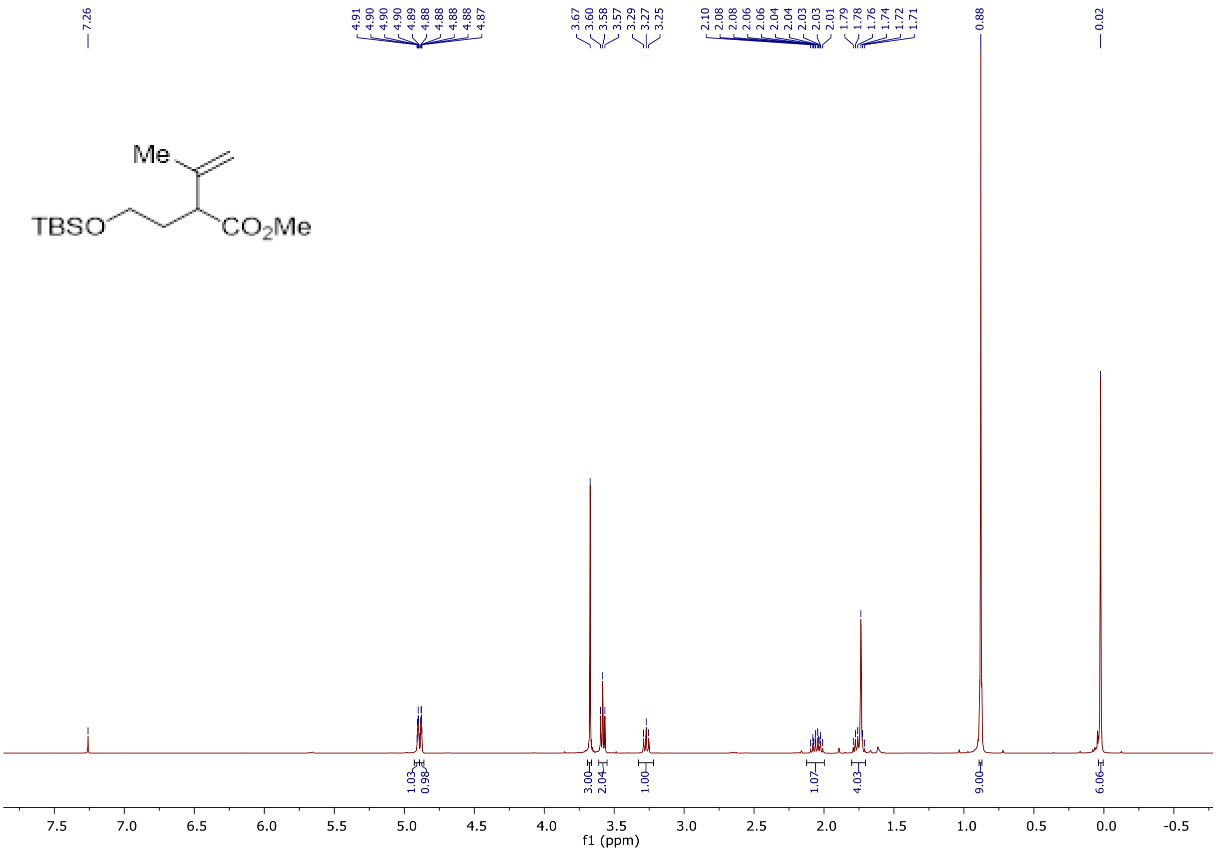

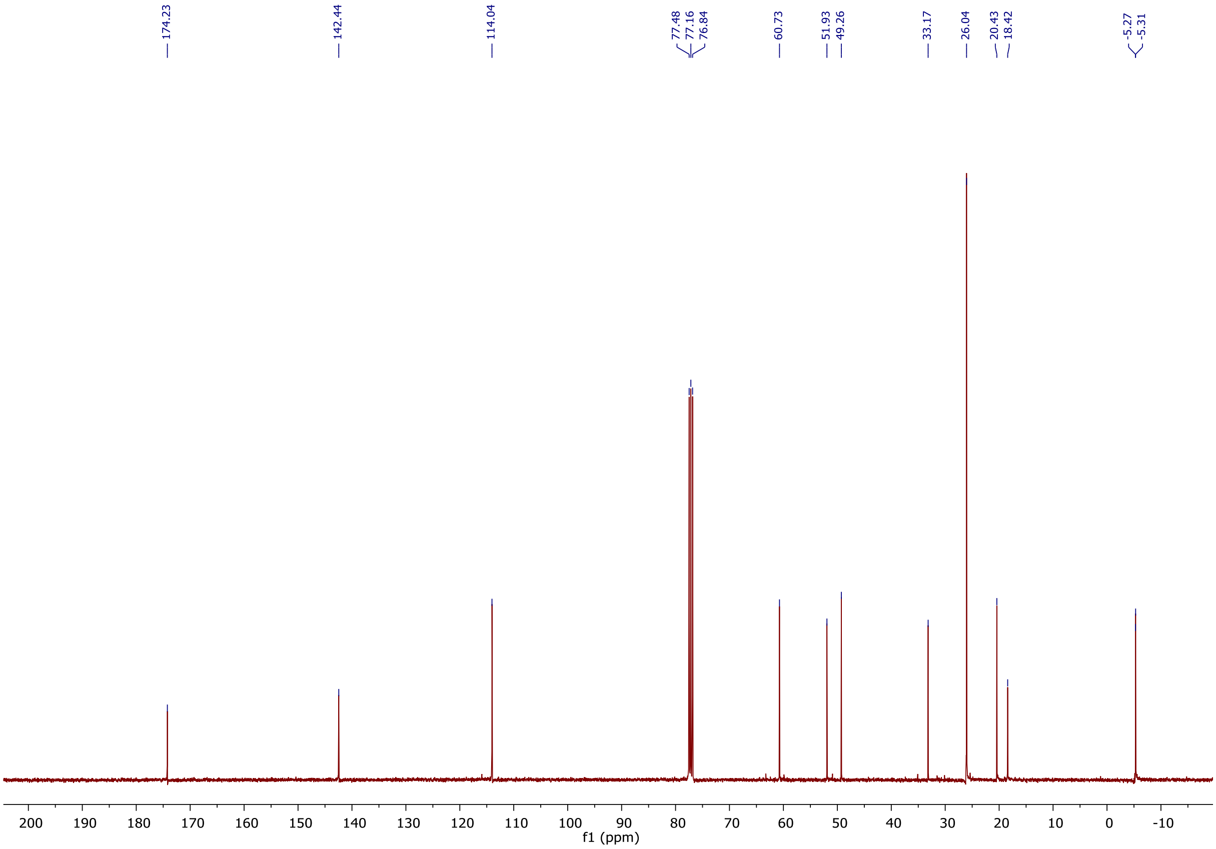

3-(2-((*tert*-Butyldimethylsilyl)oxy)ethyl)-2-methyloct-1-en-4-one

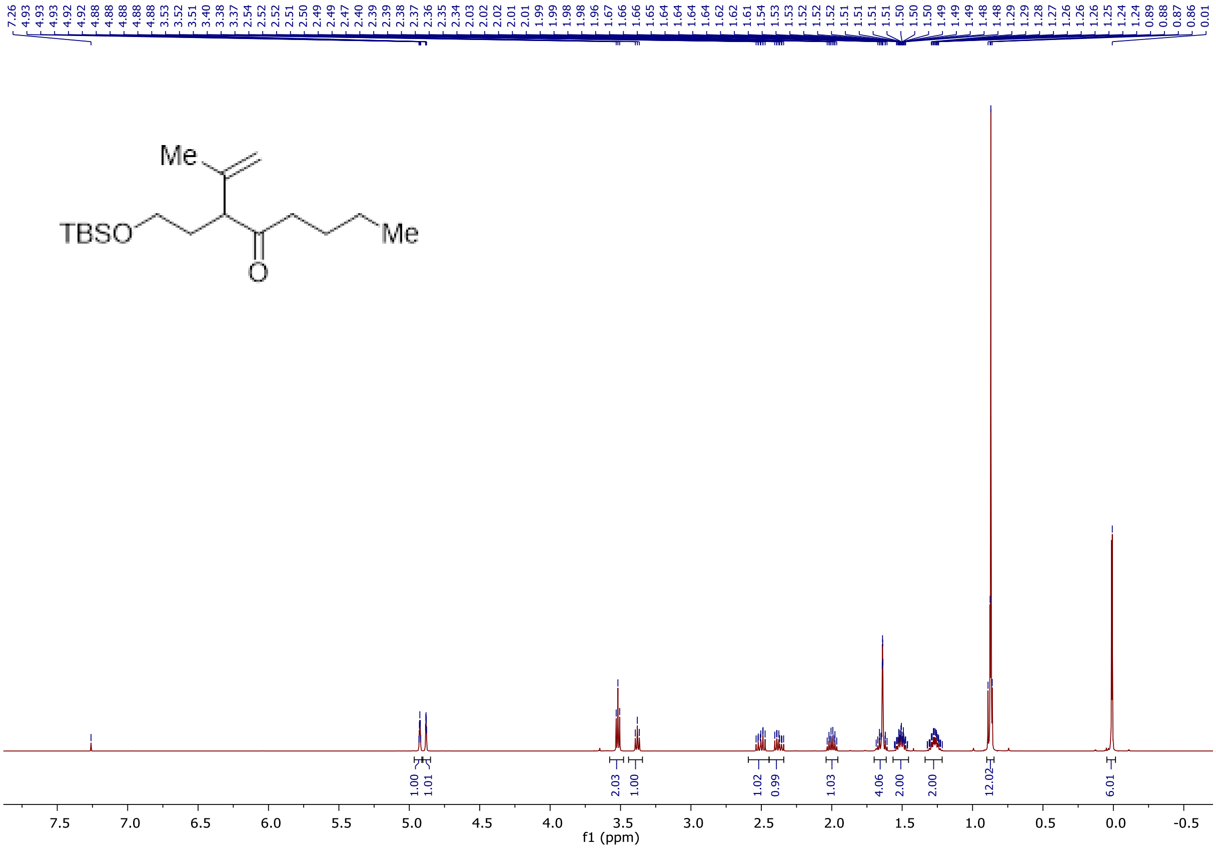

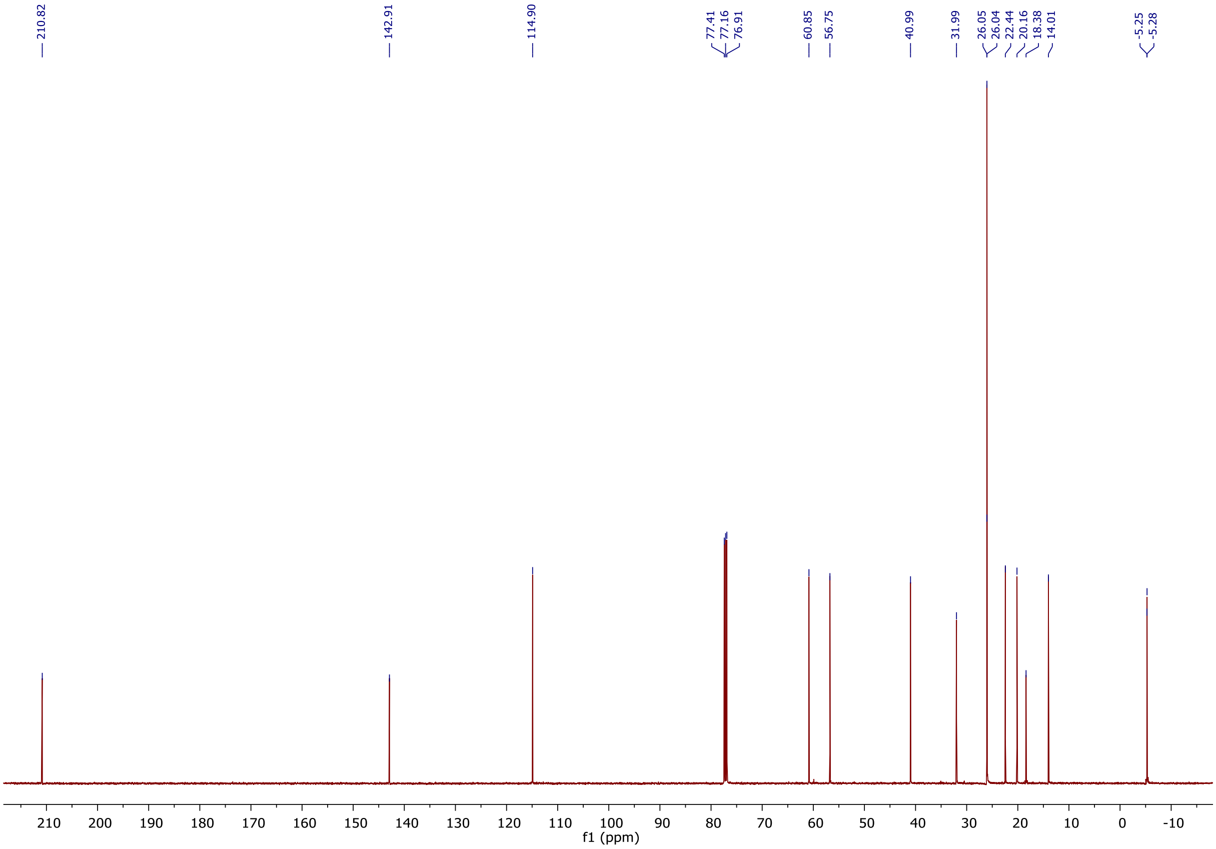

3-(2-Hydroxyethyl)-2-methyloct-1-en-4-one

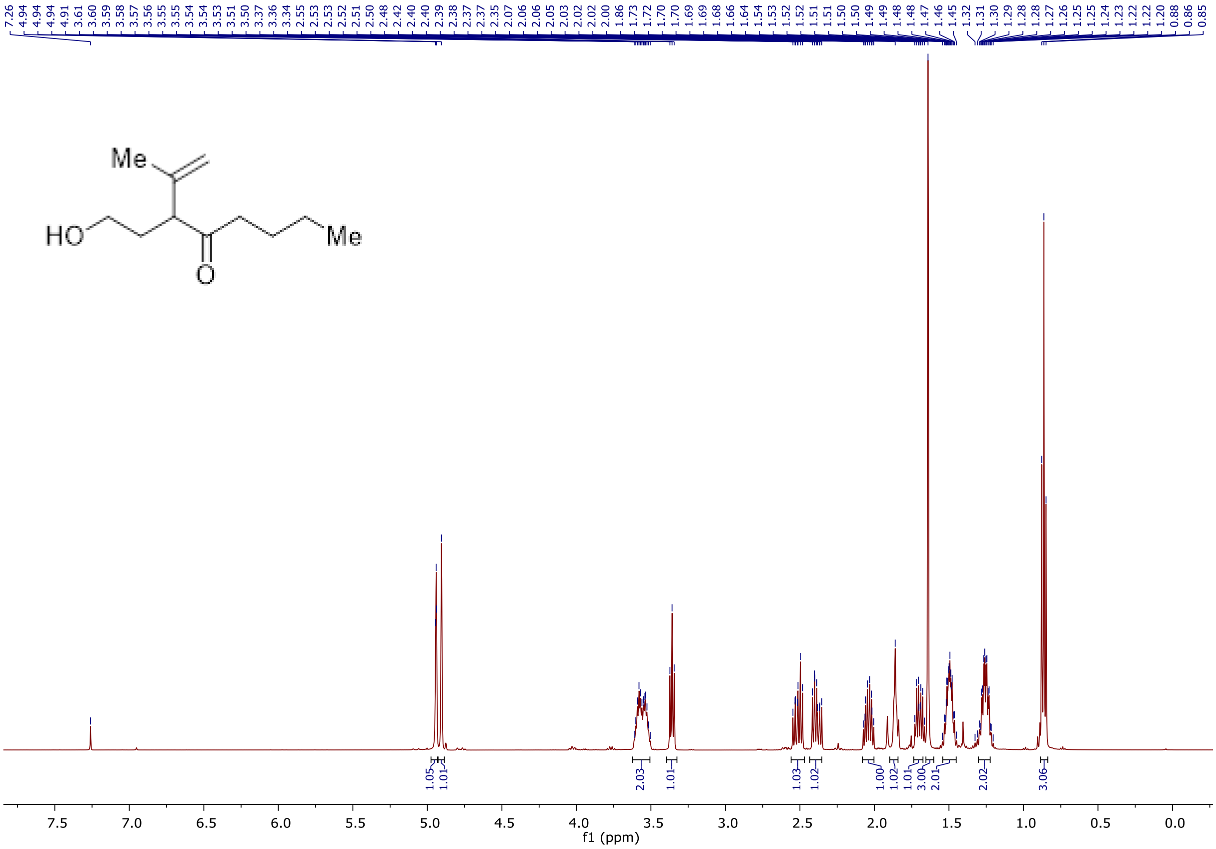

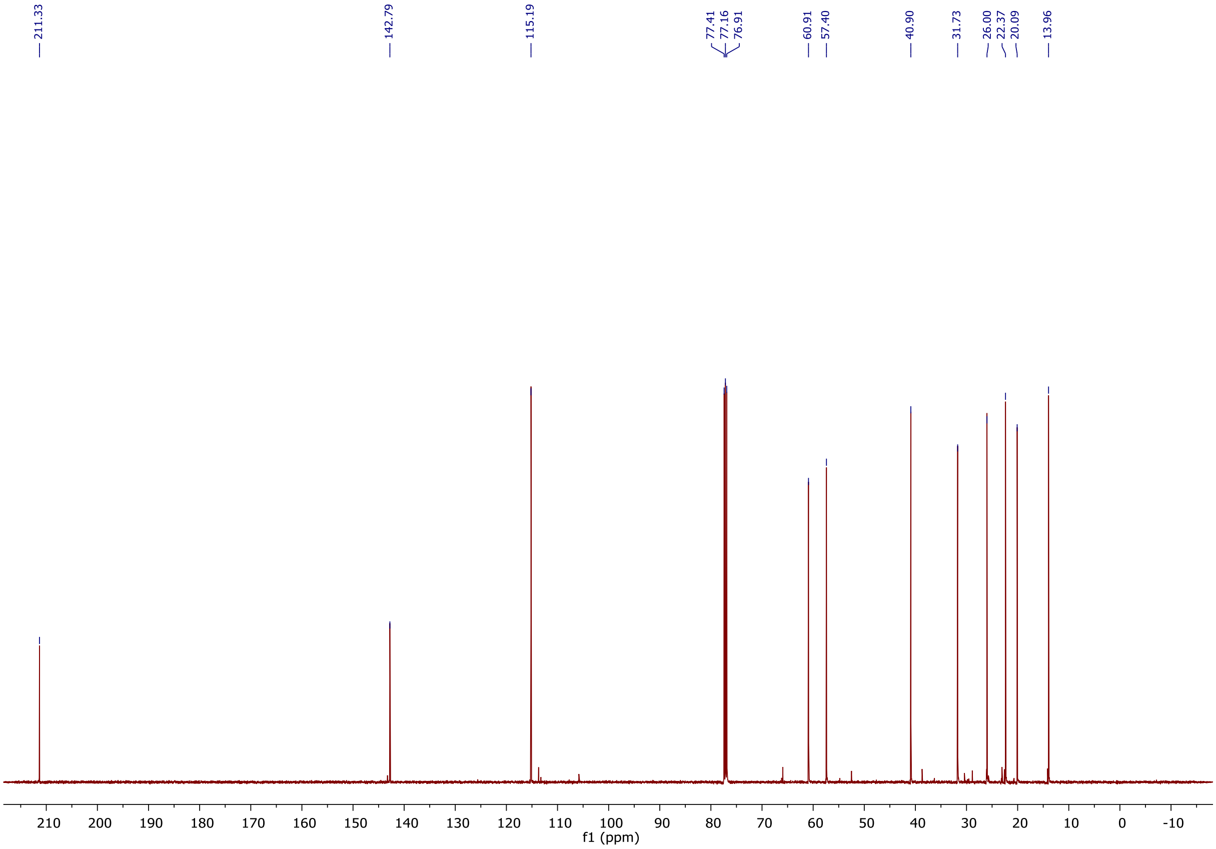

Benzyl (4-oxo-3-(prop-1-en-2-yl)octyl)((perfluorobenzoyl)oxy)carbamate (3b)

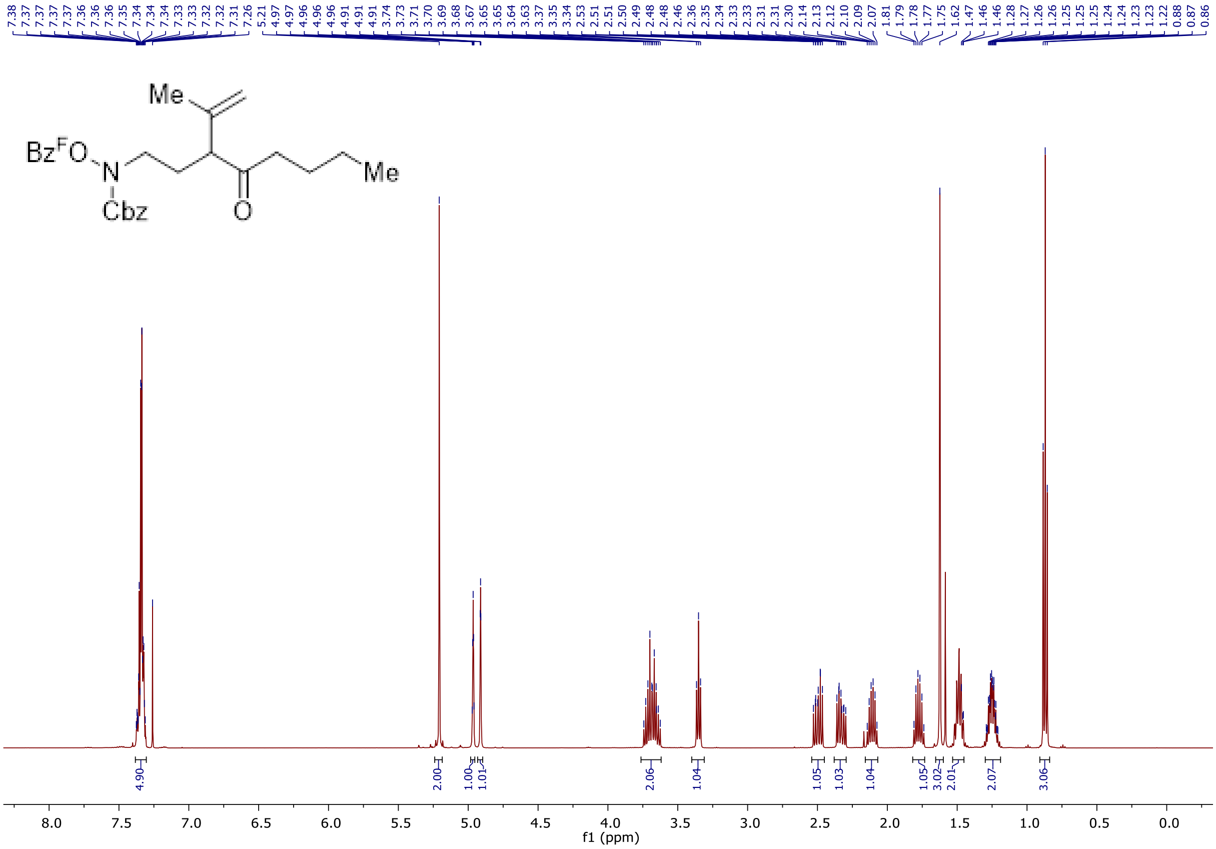

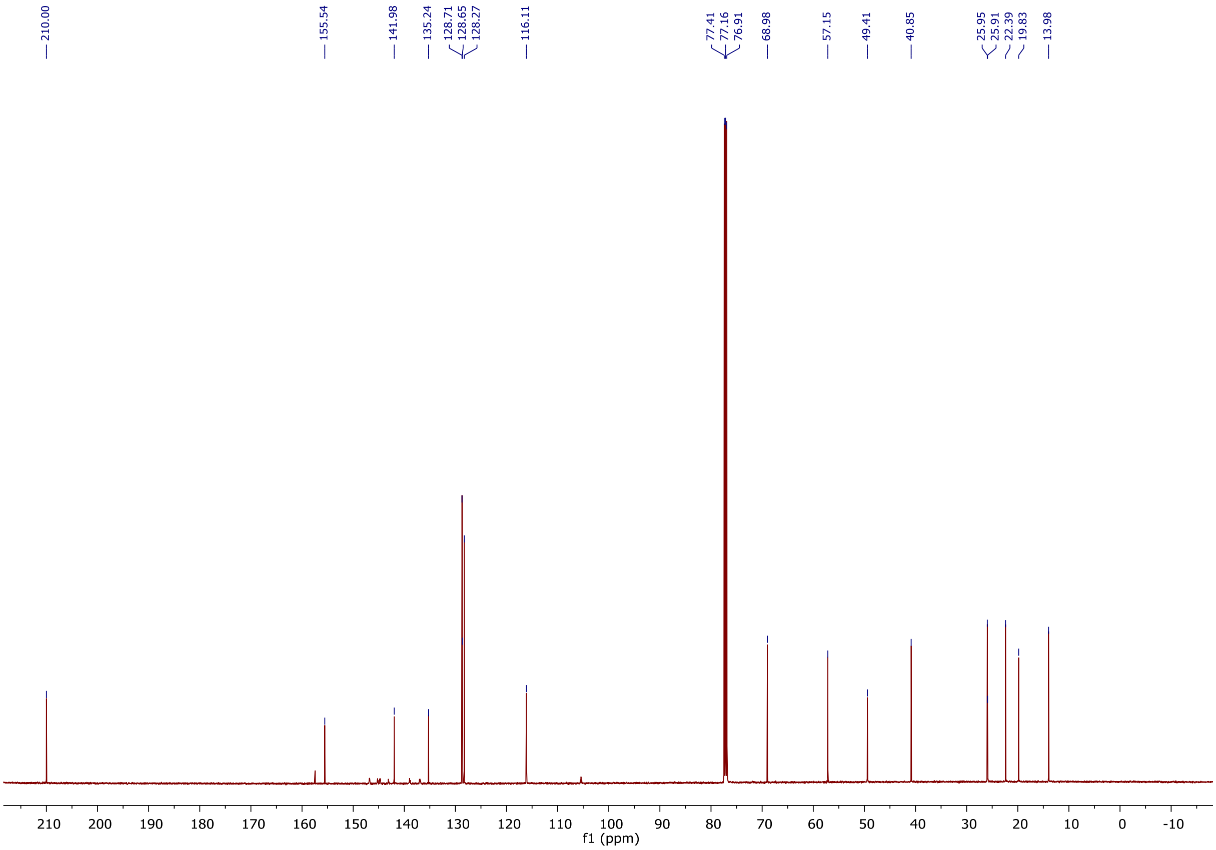

Benzyl 6-methyl-4-pentanoyl-3,4-dihydropyridine-1(2*H*)-carboxylate (4b-C2-C3)

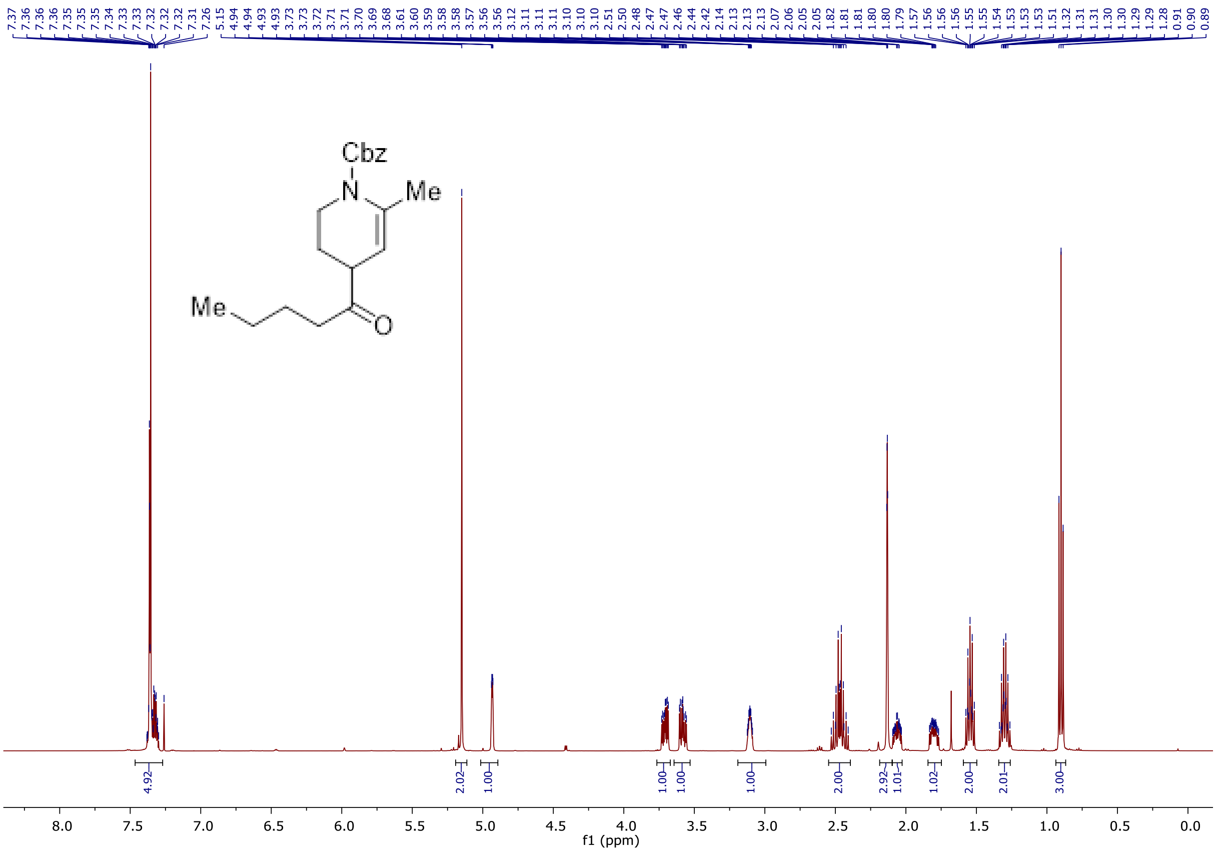

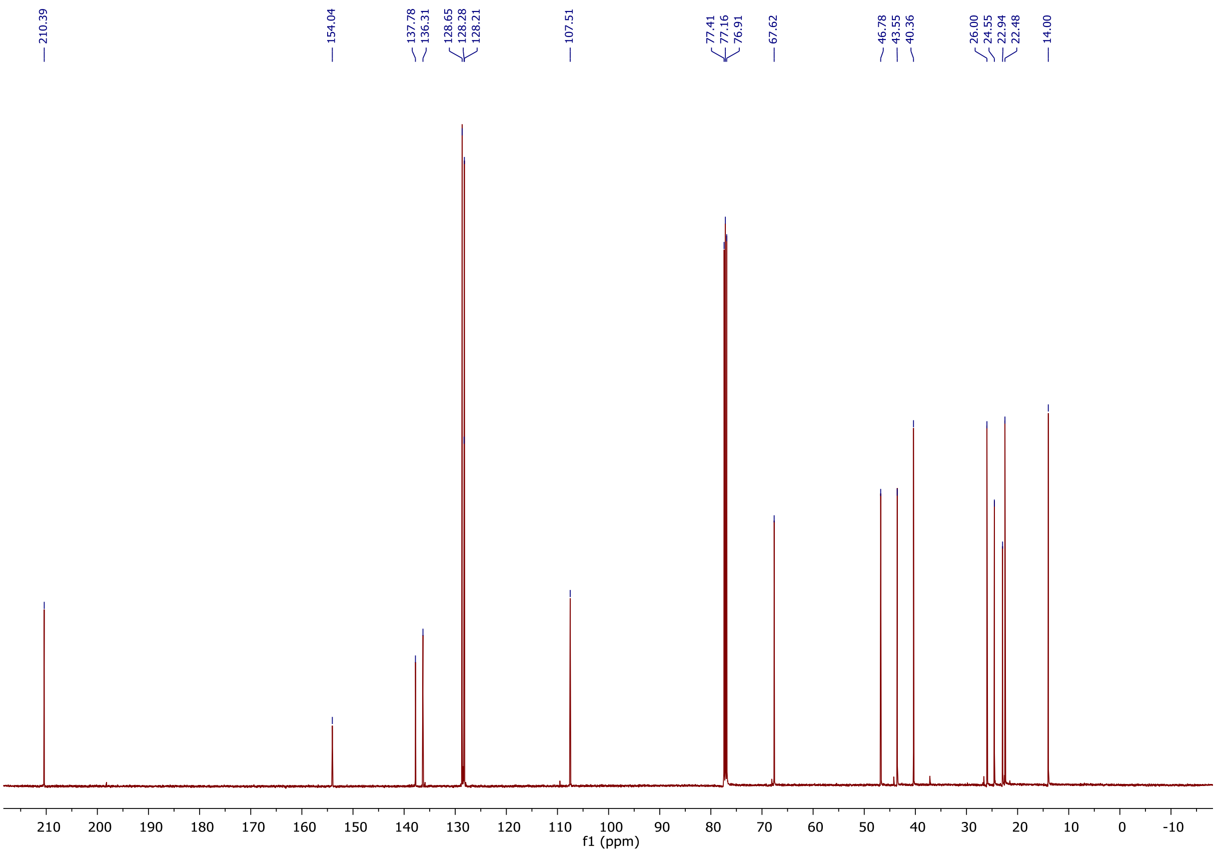

Benzyl 6-methyl-4-pentanoyl-3,6-dihydropyridine-1(2*H*)-carboxylate (4b-C3-C4)

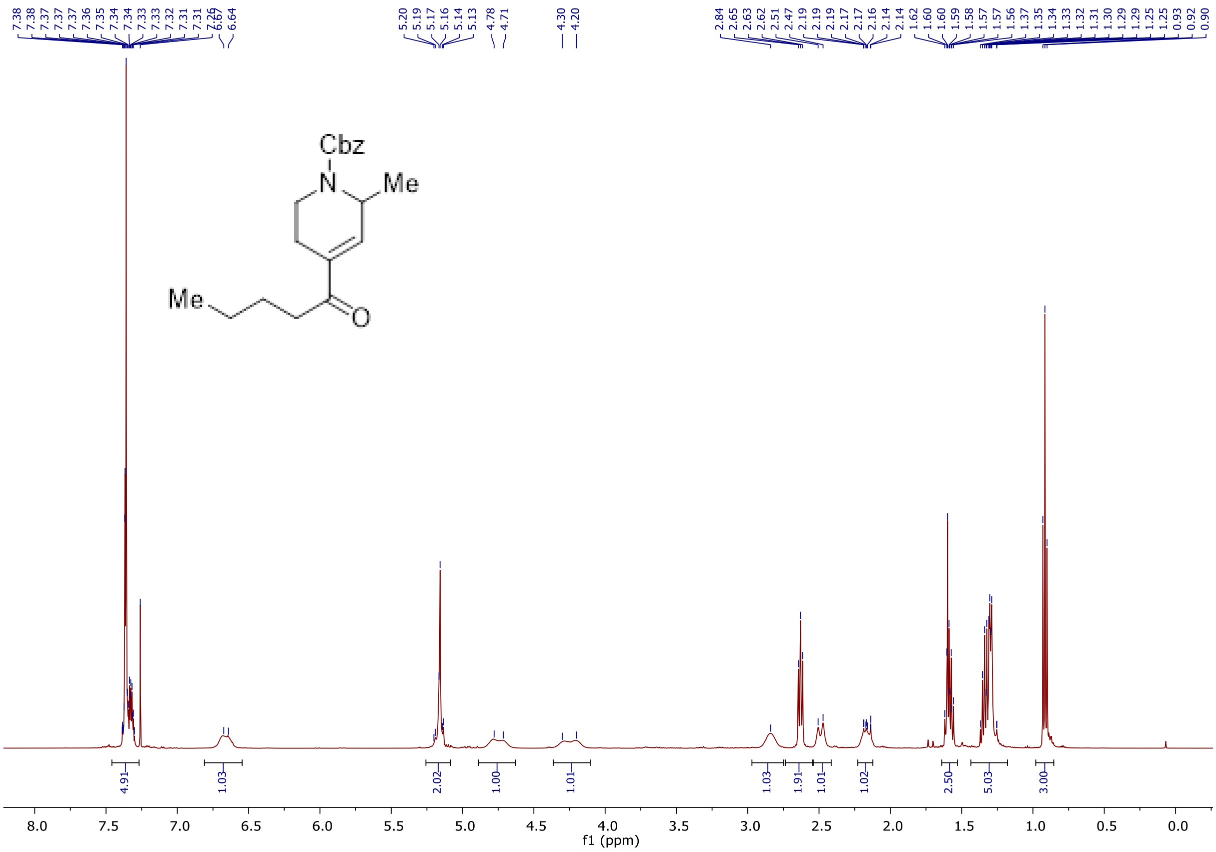

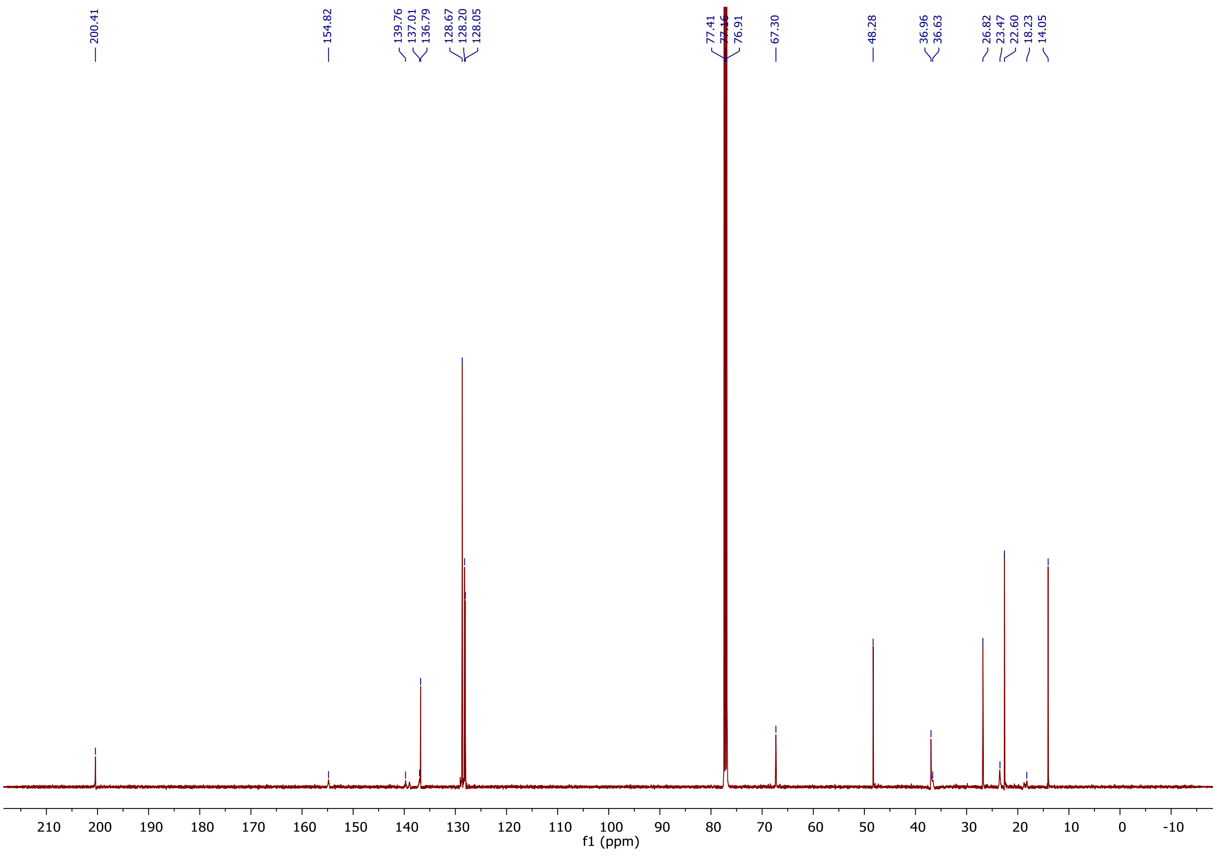

2-(2-((*tert*-Butyldimethylsilyl)oxy)ethyl)-3-methyl-1-phenylbut-3-en-1-one

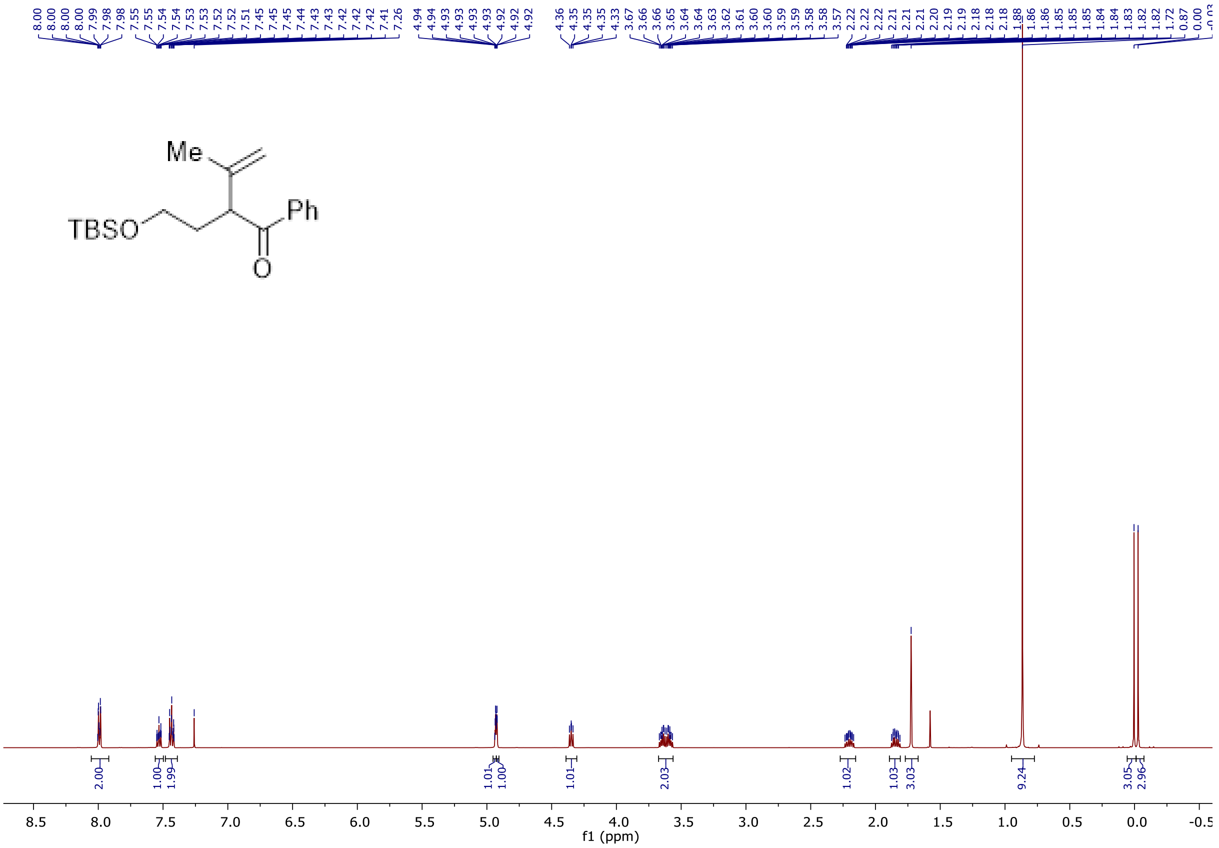

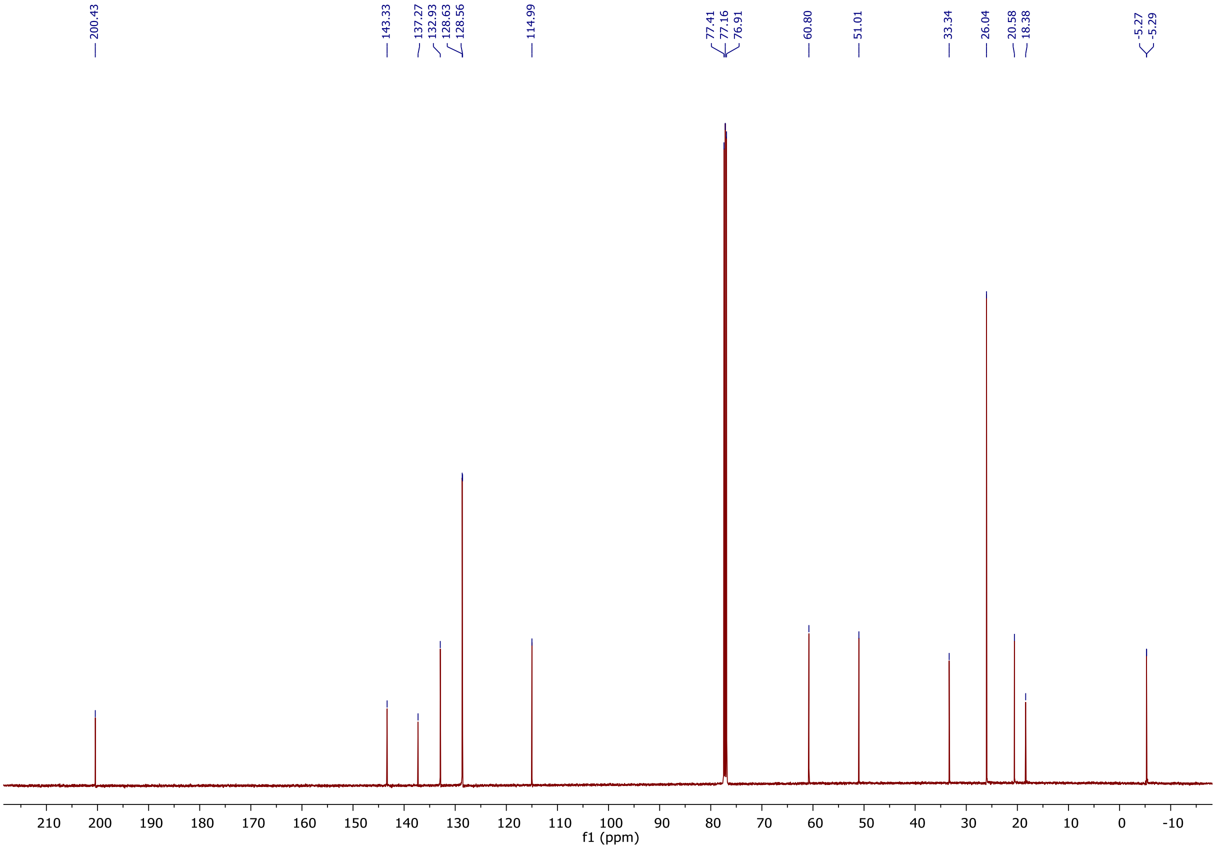

2-(2-Hydroxyethyl)-3-methyl-1-phenylbut-3-en-1-one

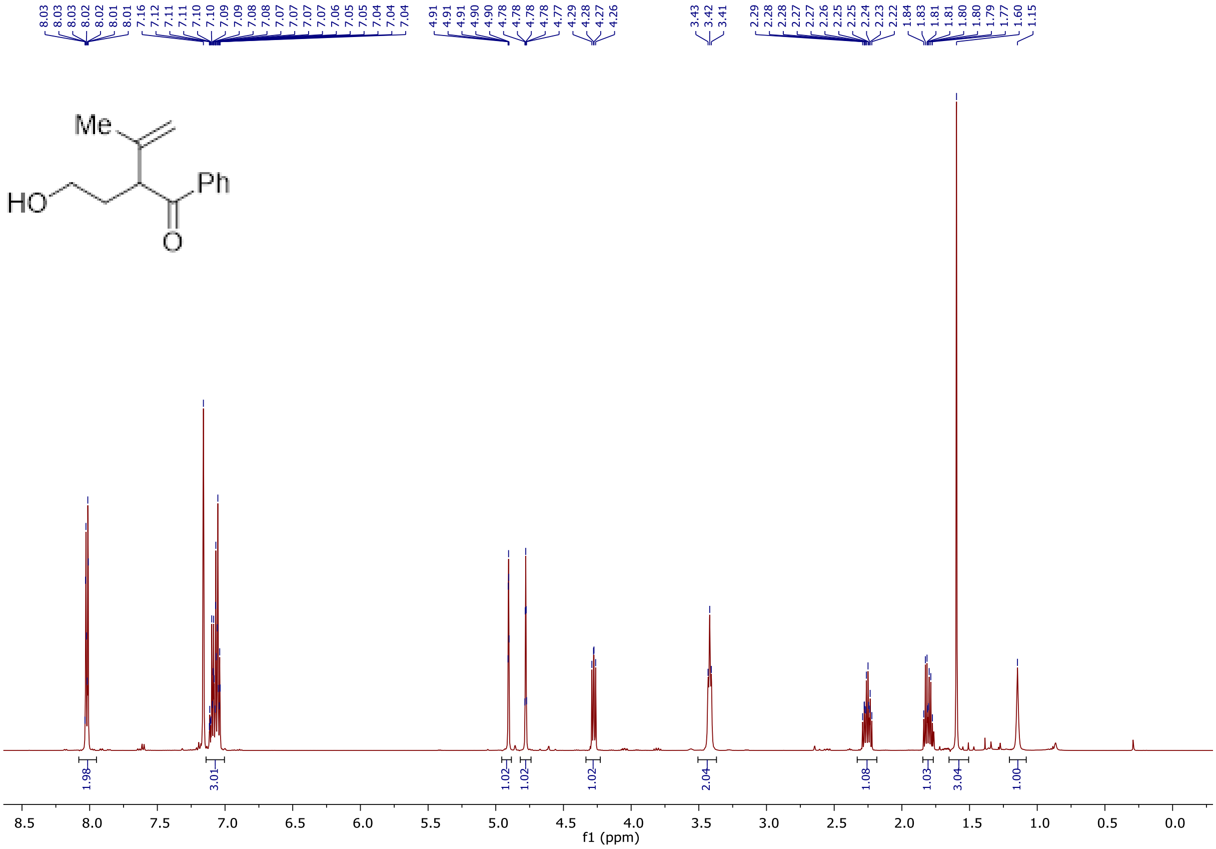

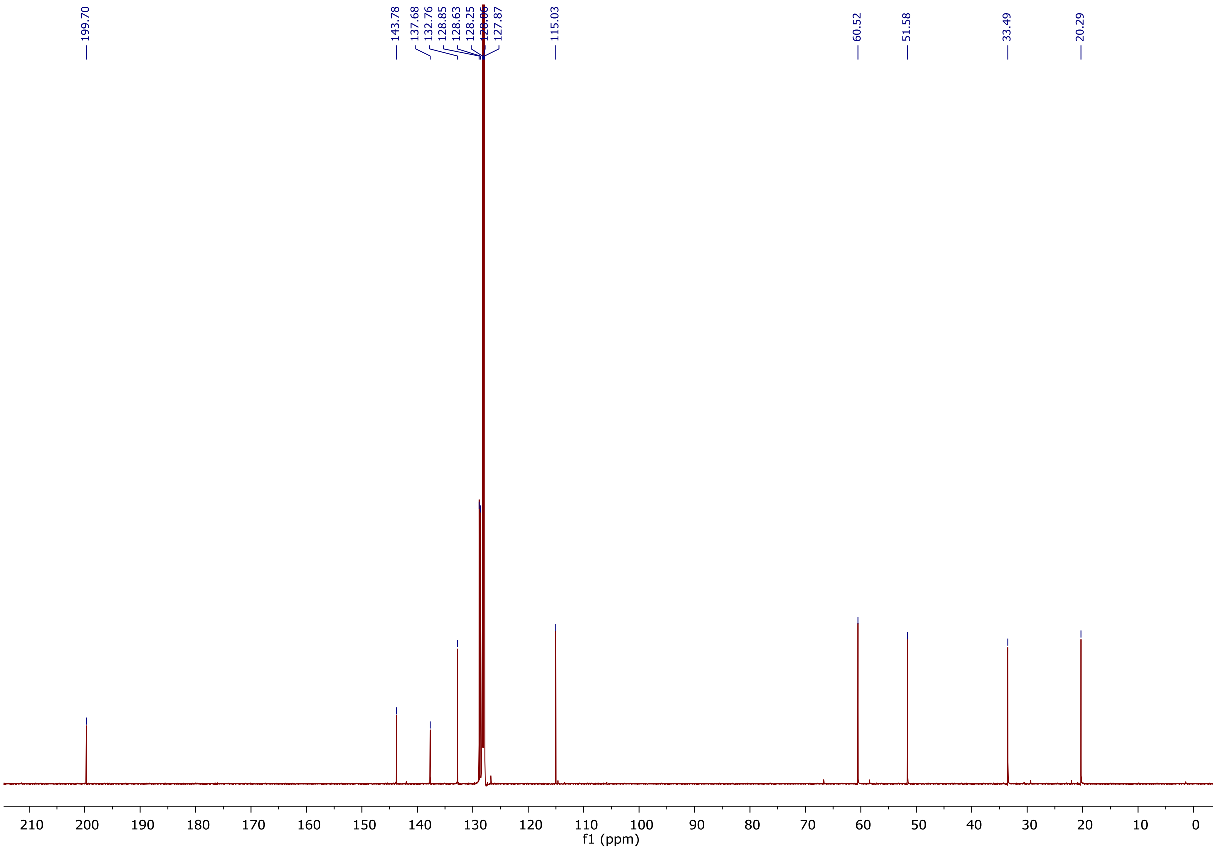

Benzyl (3-benzoyl-4-methylpent-4-en-1-yl)((perfluorobenzoyl)oxy)carbamate (3c)

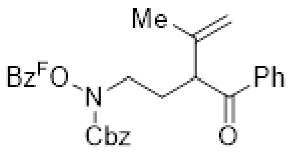

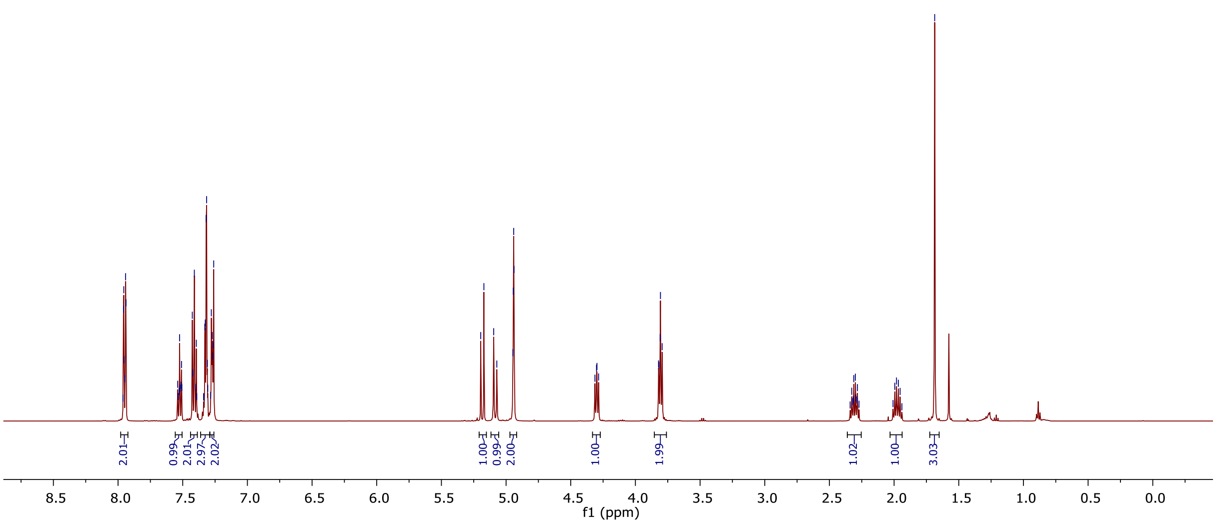

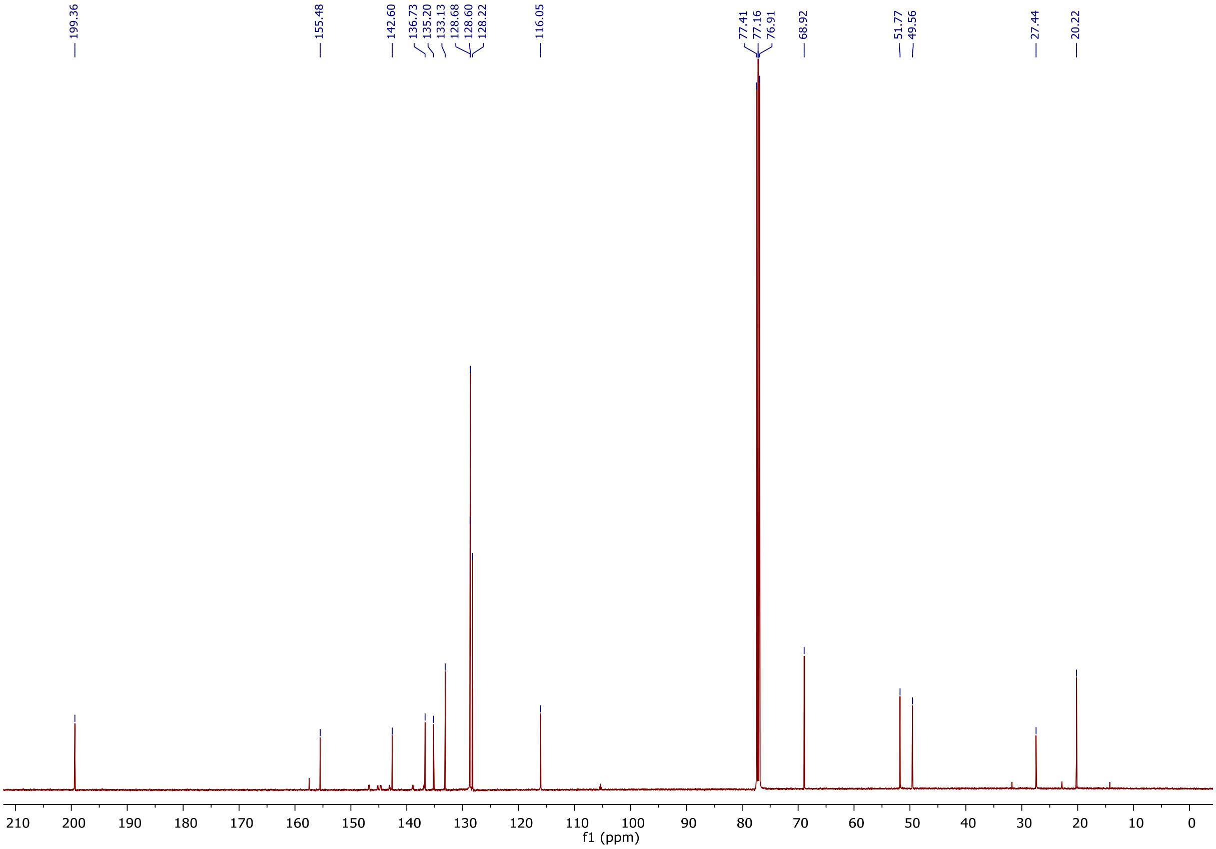

Benzyl 4-benzoyl-6-methyl-3,4-dihydropyridine-1(2*H*)-carboxylate (4c-C2-C3)

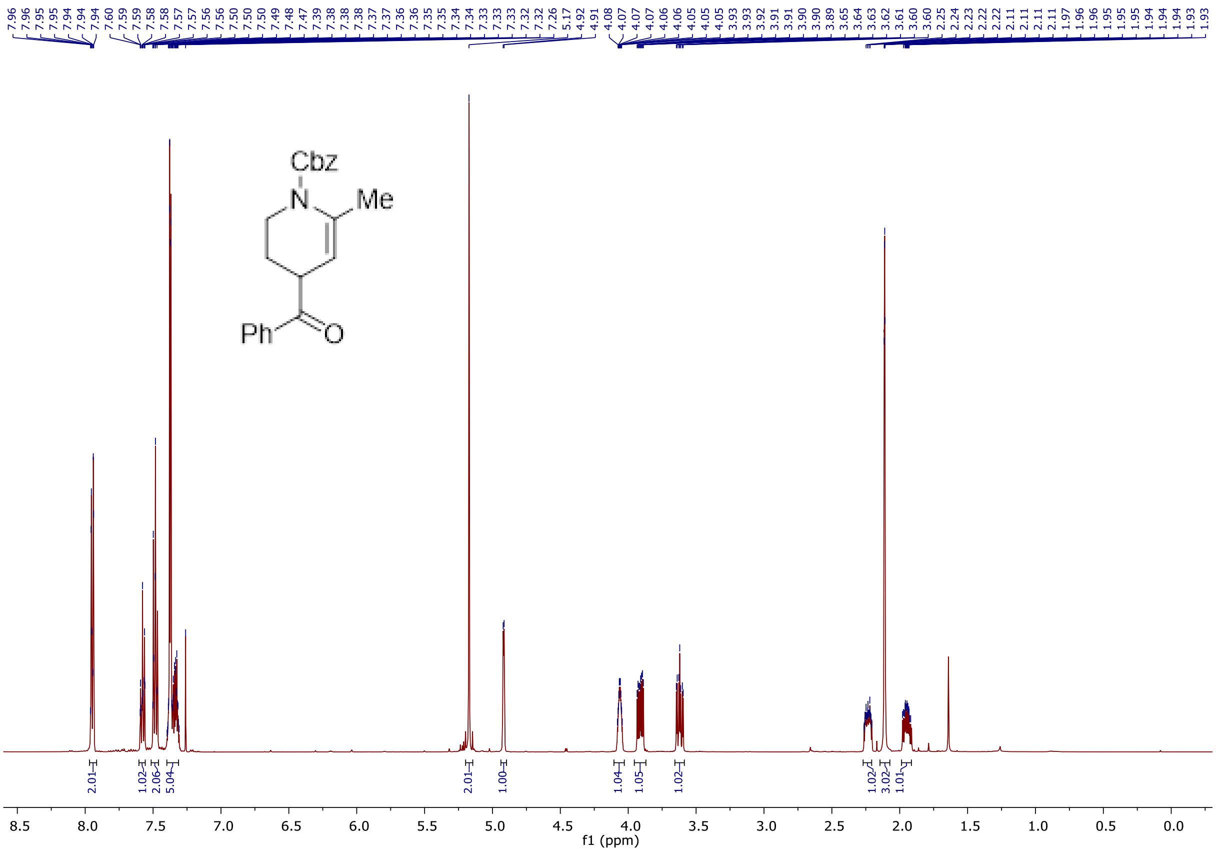

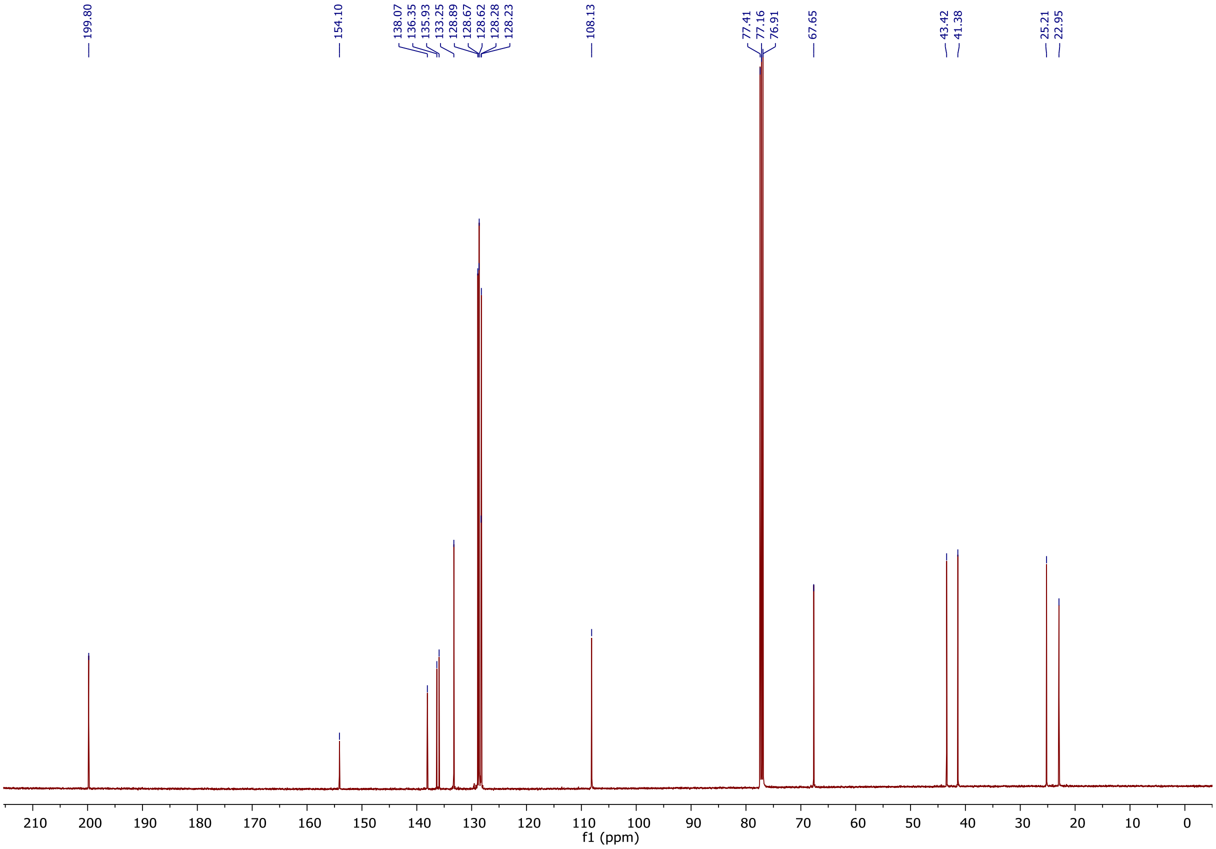

Benzyl 4-benzoyl-6-methyl-3,6-dihydropyridine-1(2*H*)-carboxylate (4c-C3-C4)

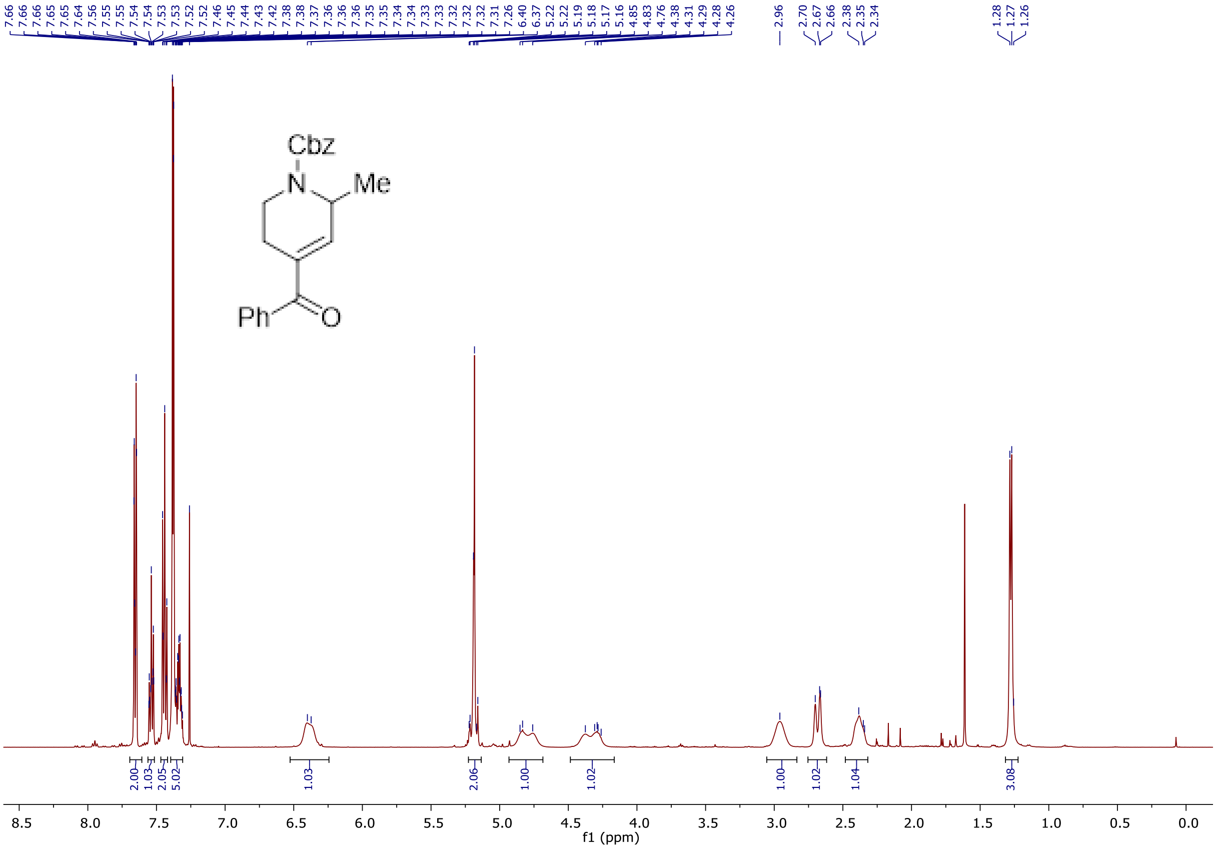

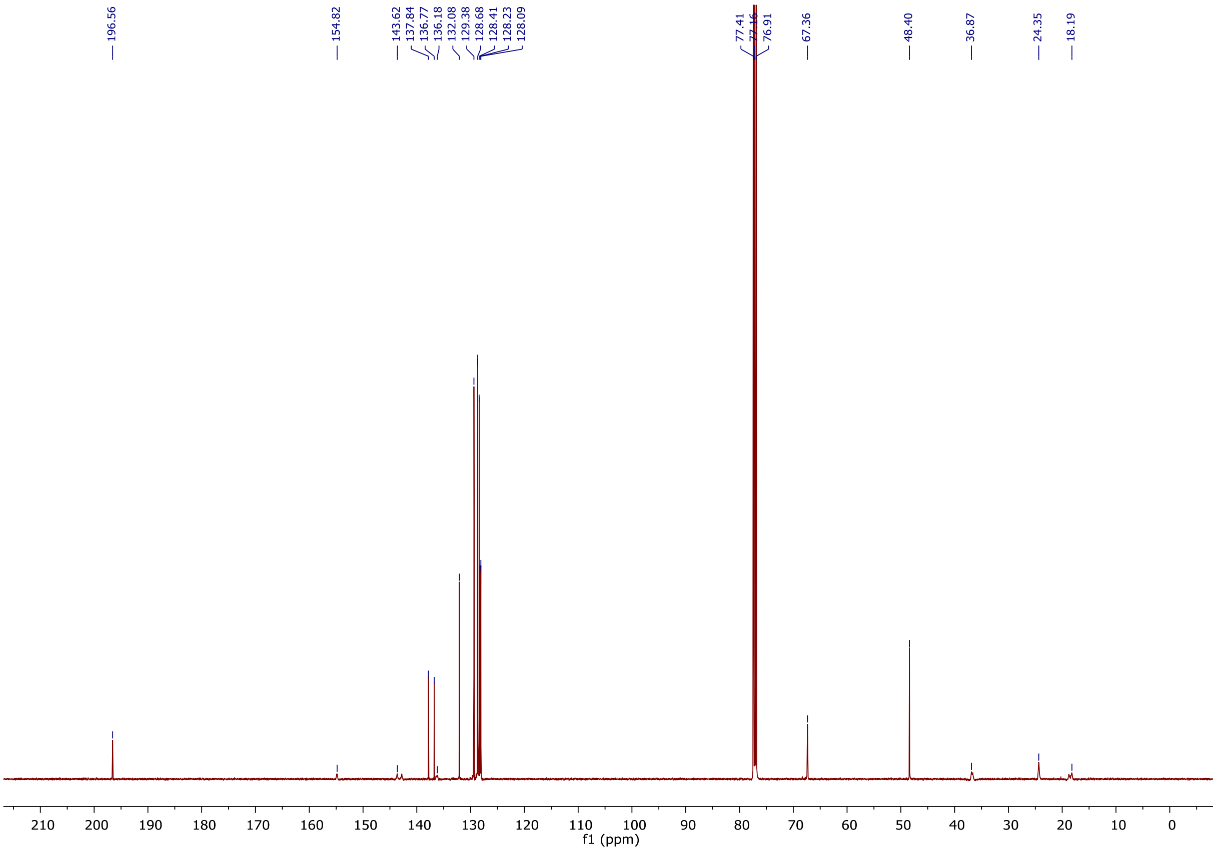

3-(2-((*tert*-Butyldimethylsilyl)oxy)ethyl)-2-methylnona-1,8-dien-4-one

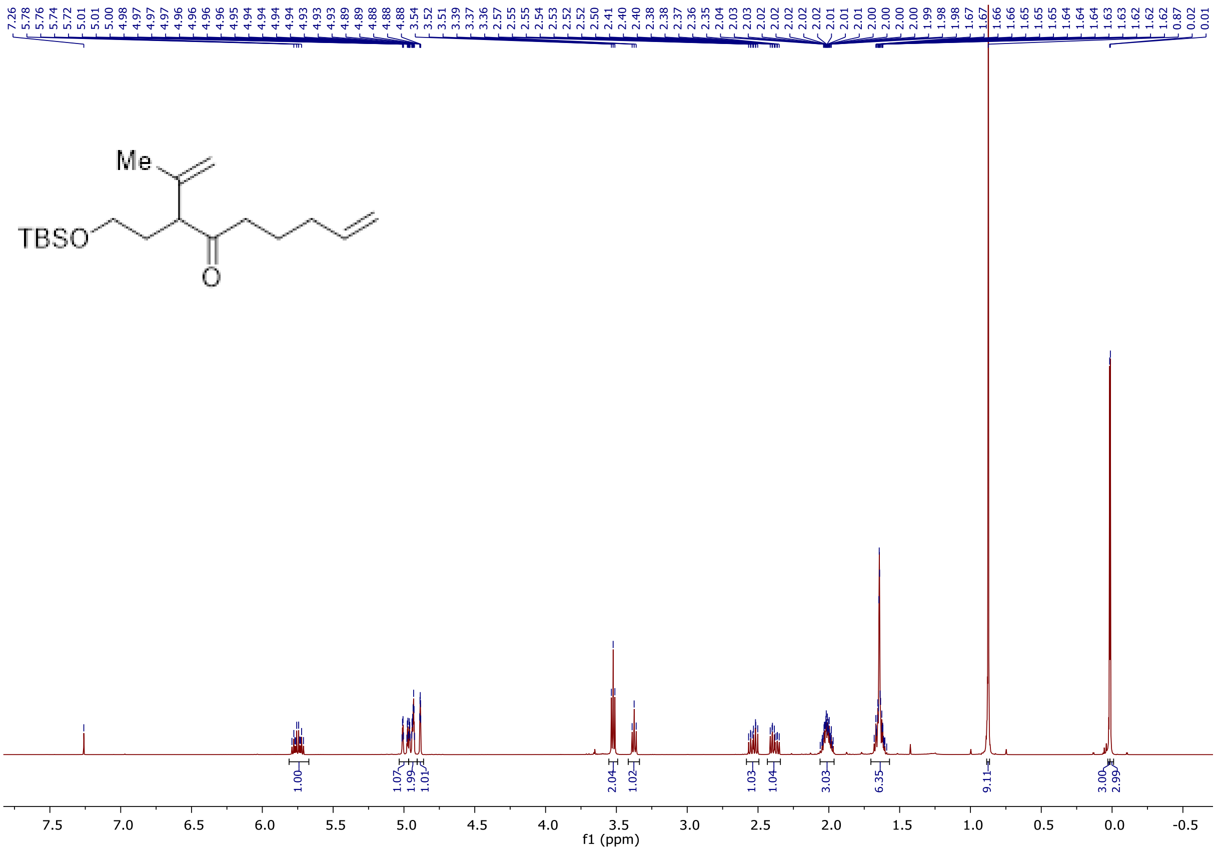

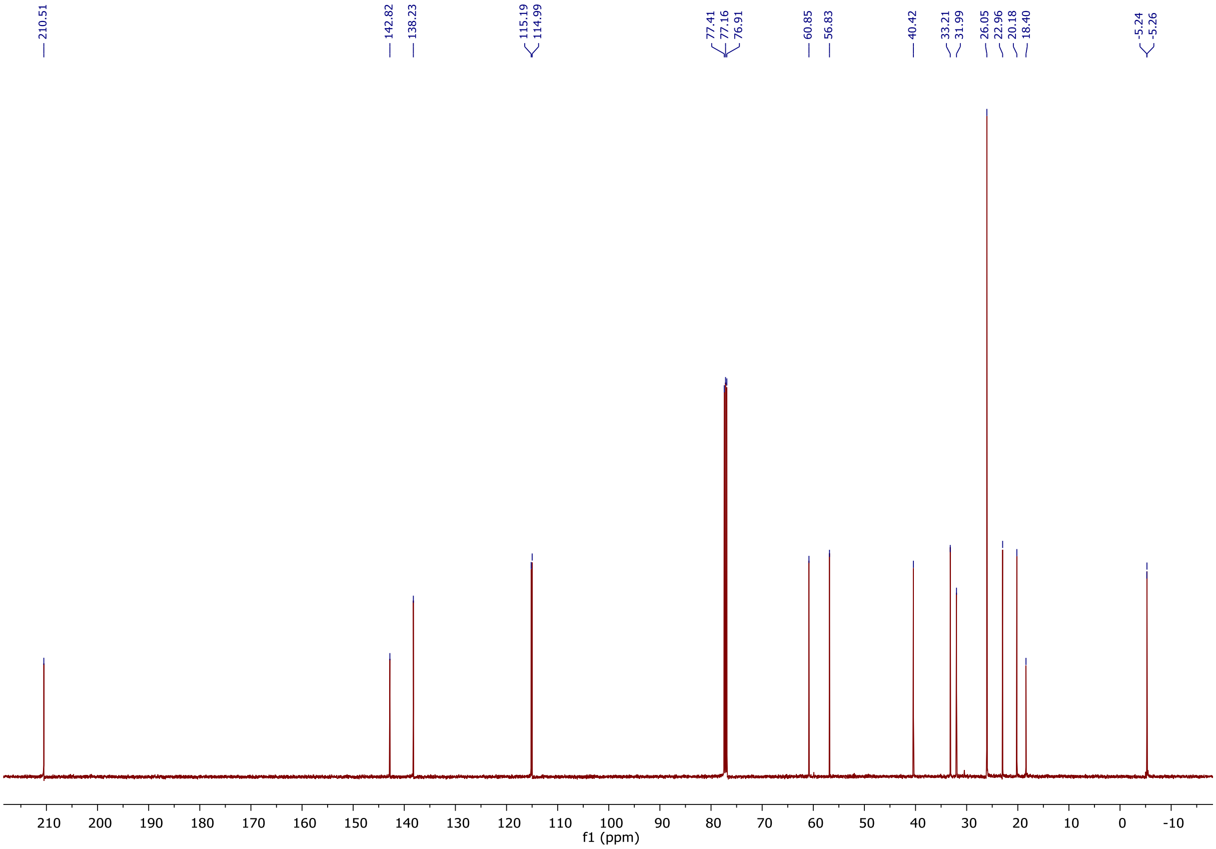

Methyl (*E*)-8-(2-((*tert*-butyldimethylsilyl)oxy)ethyl)-9-methyl-7-oxodeca-2,9-dienoate

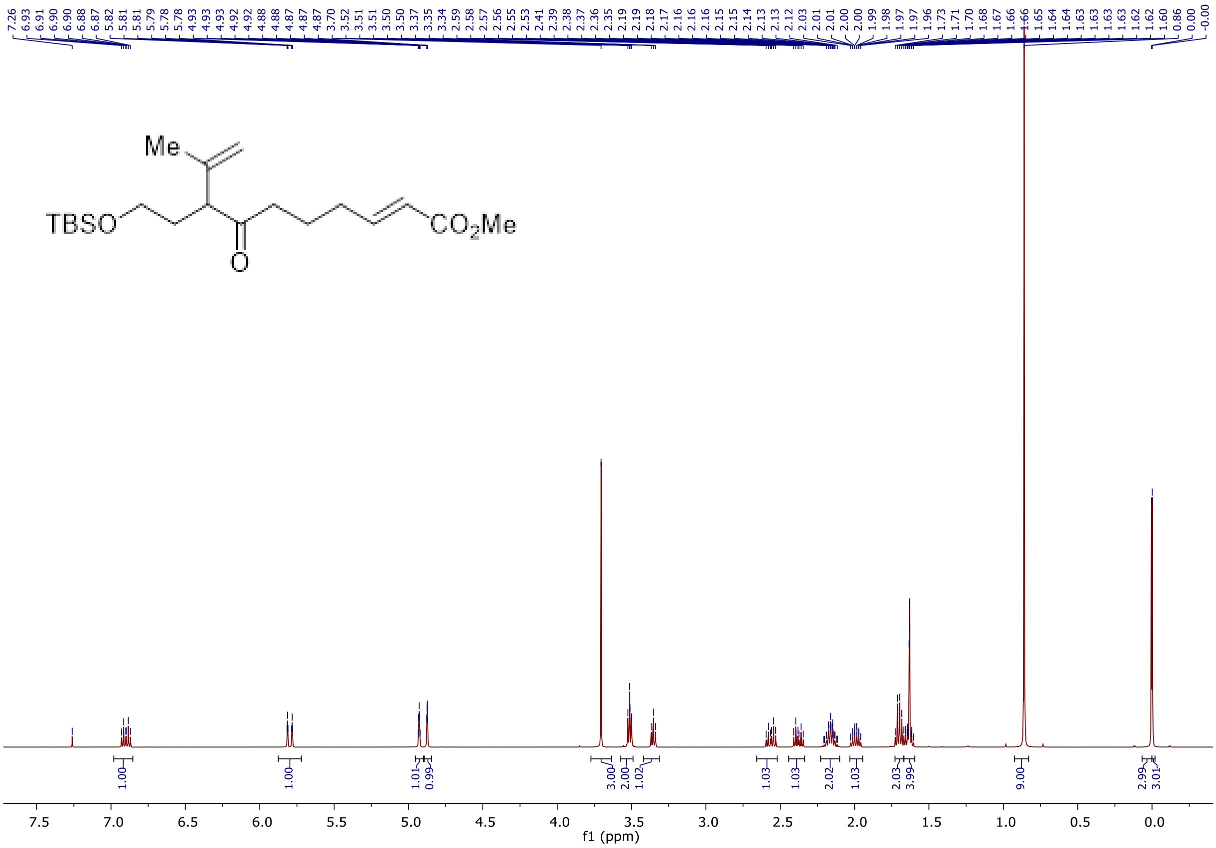

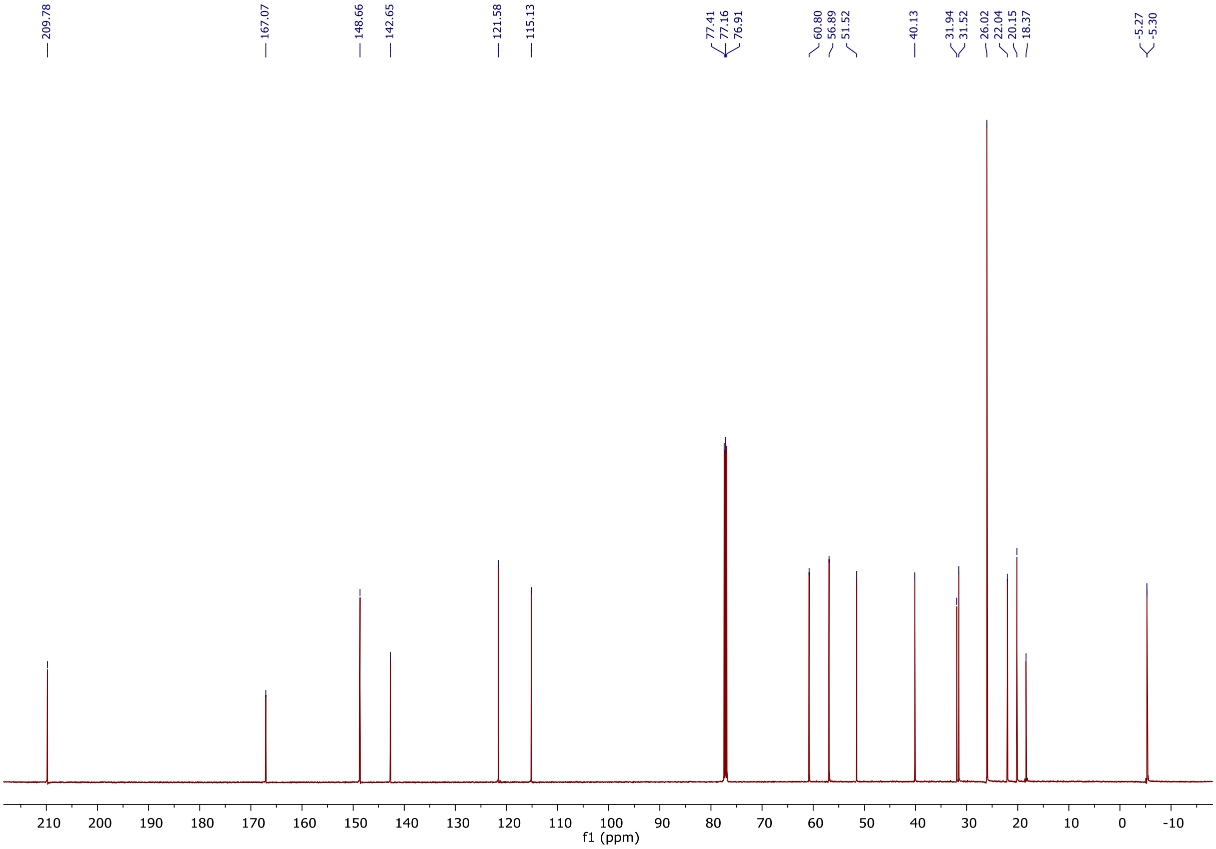

Methyl (*E*)-8-(2-hydroxyethyl)-9-methyl-7-oxodeca-2,9-dienoate

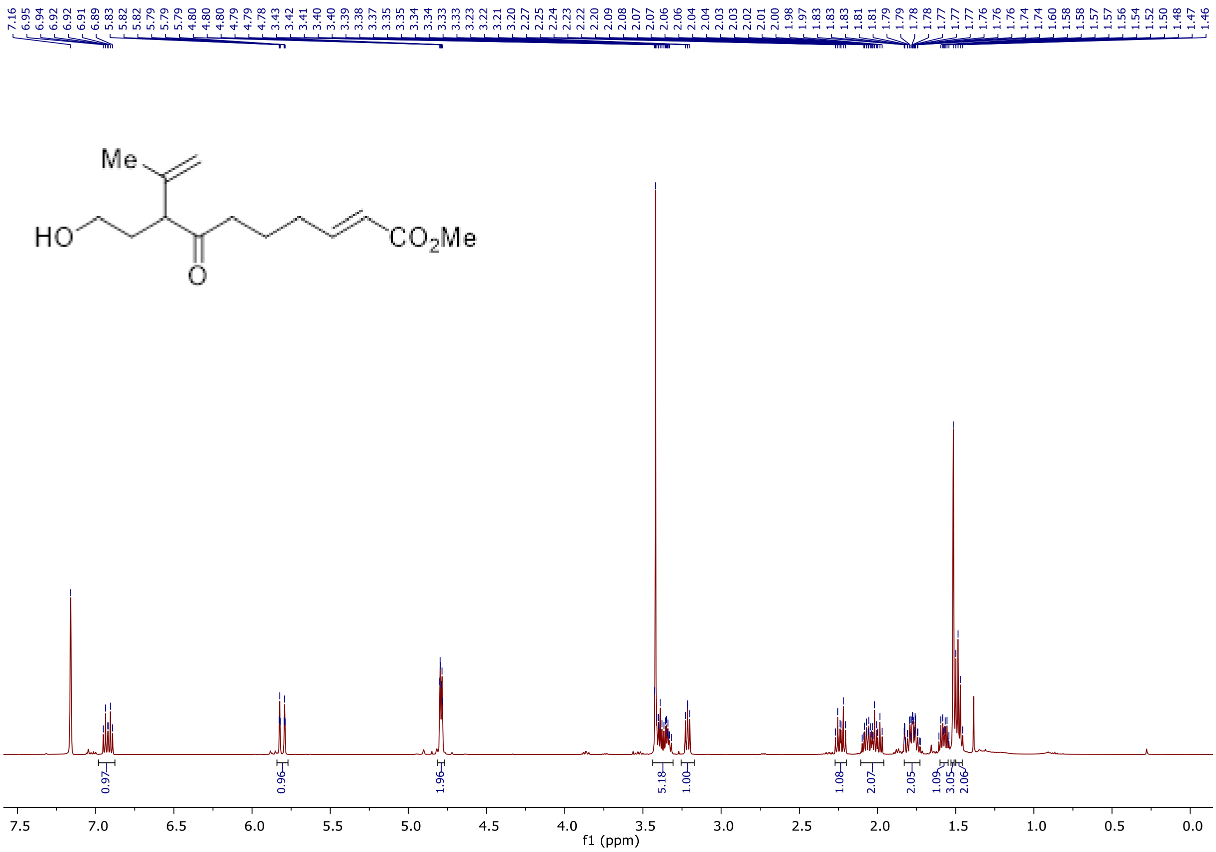

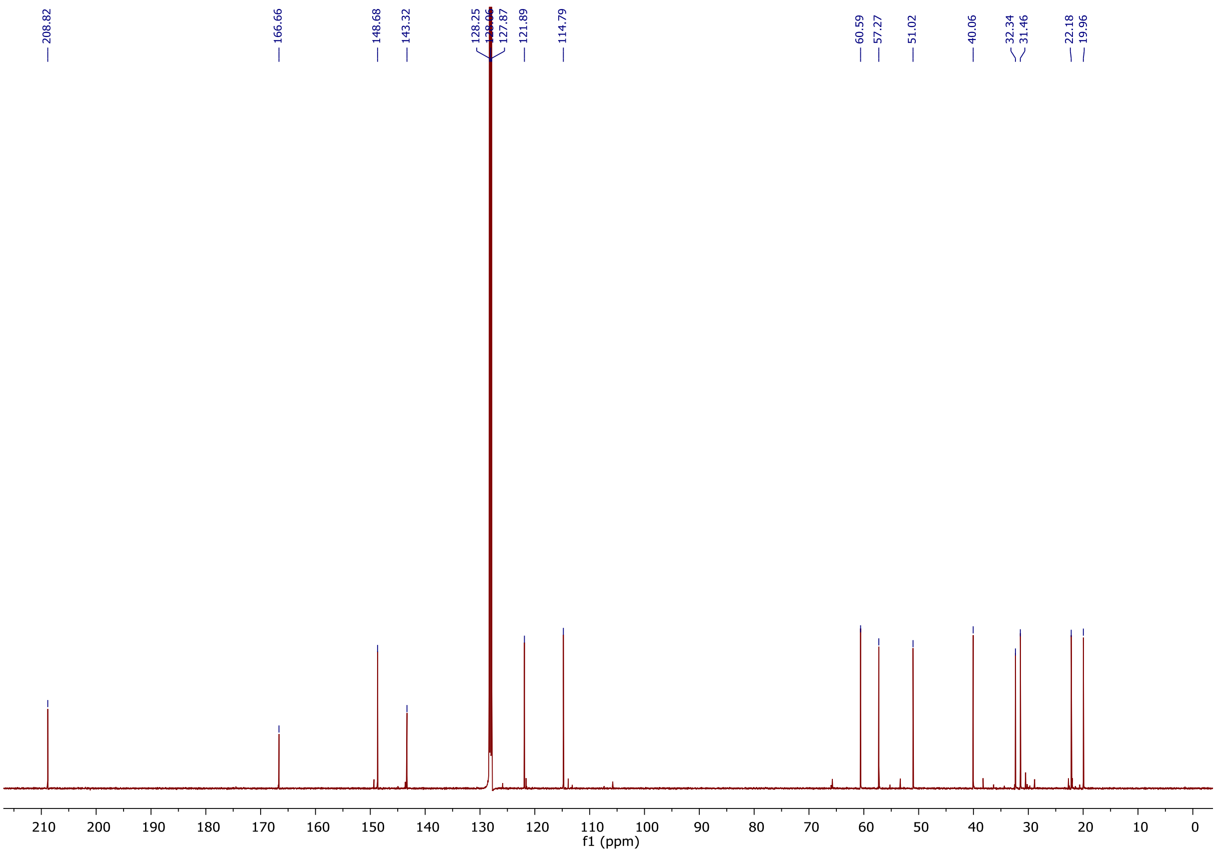

Methyl (*E*)-8-(2-(((benzyloxy)carbonyl)((perfluorobenzoyl)oxy)amino)ethyl)-9-methyl-7-oxodeca-2,9-dienoate (3d)

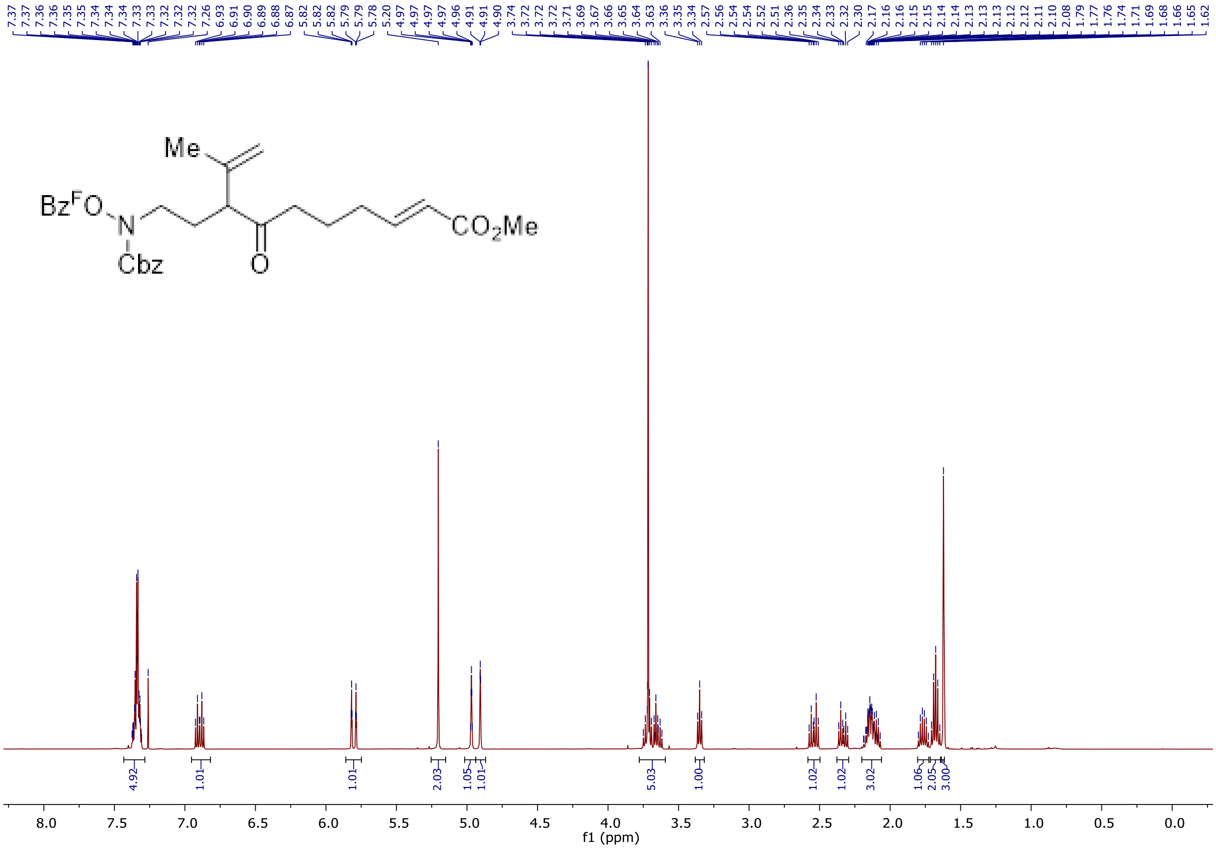

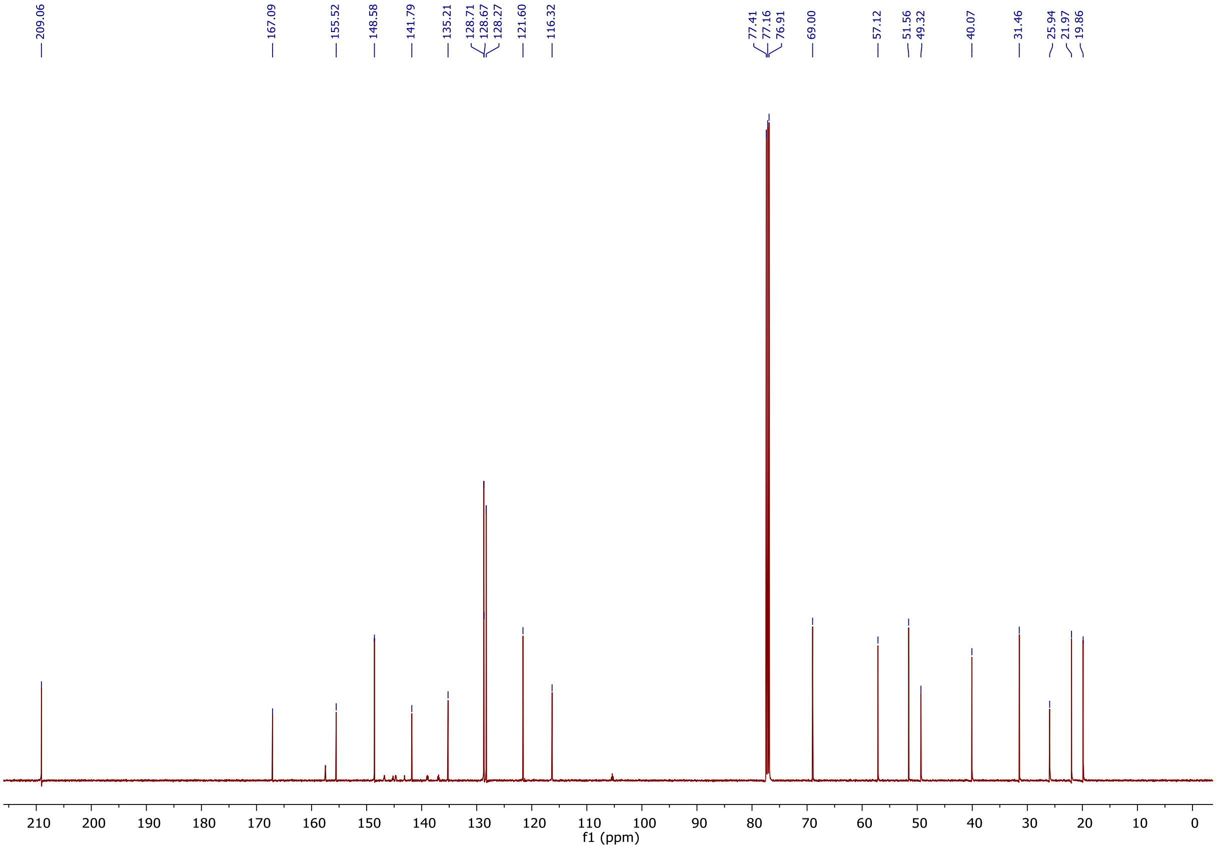

Benzyl (*E*)-4-(7-methoxy-7-oxohept-5-enoyl)-6-methyl-3,4-dihydropyridine-1(2*H*)-carboxylate (4d-C2-C3)

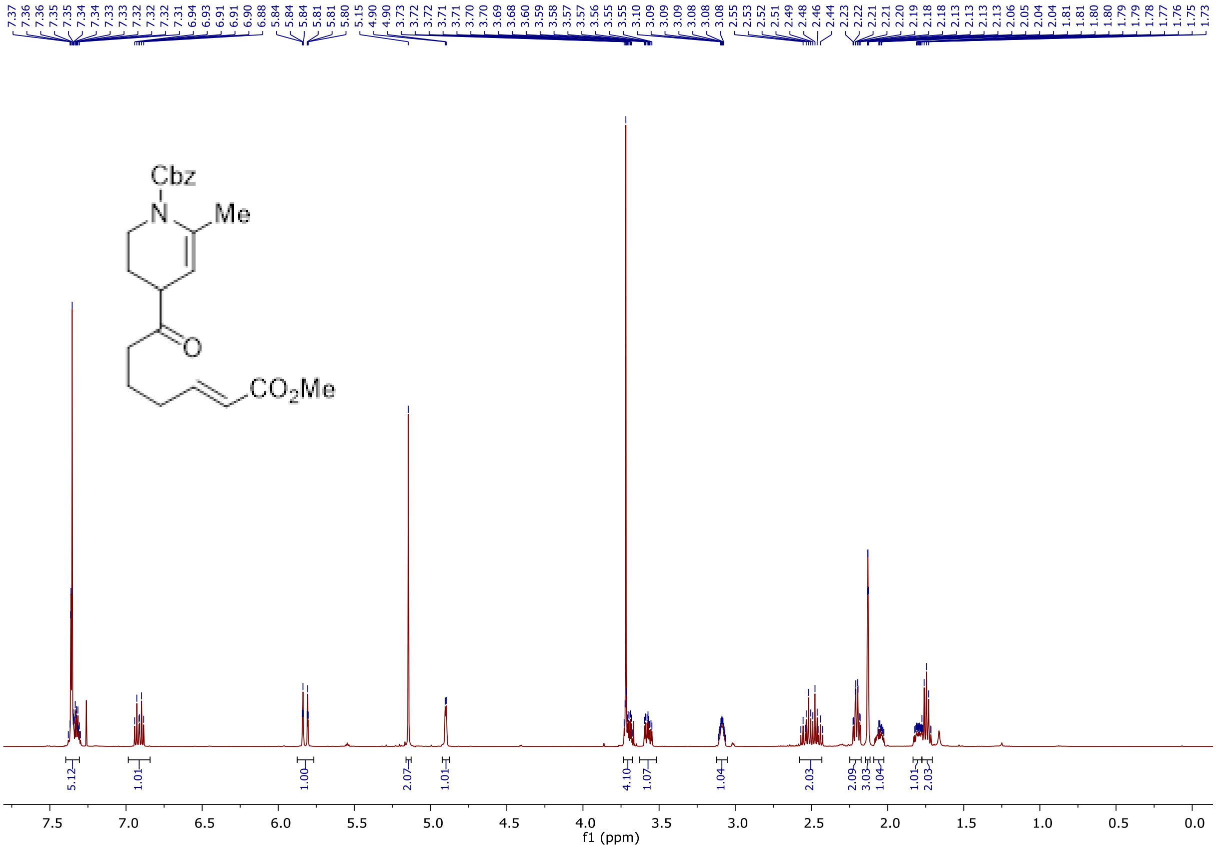

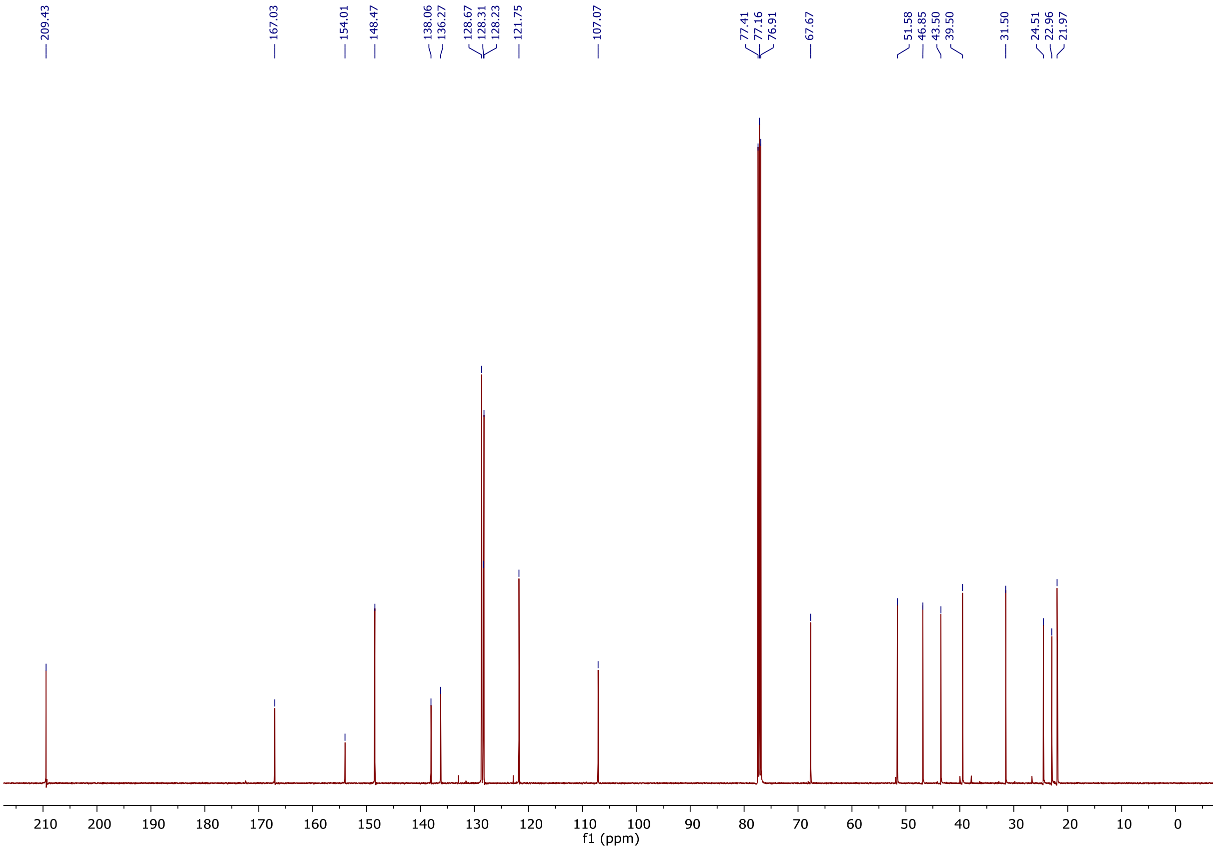

Benzyl (*E*)-4-(7-methoxy-7-oxohept-5-enoyl)-6-methyl-3,6-dihydropyridine-1(2*H*)-carboxylate (4d-C3-C4)

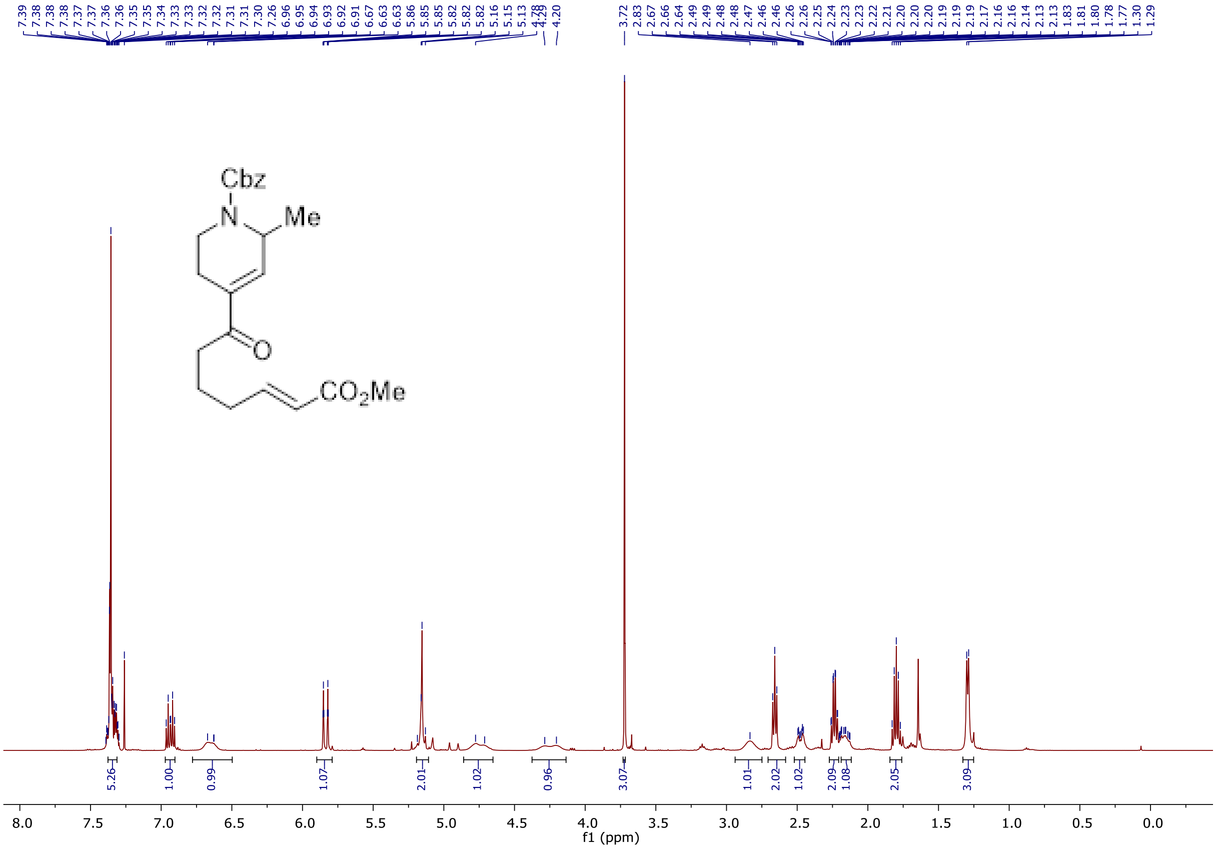

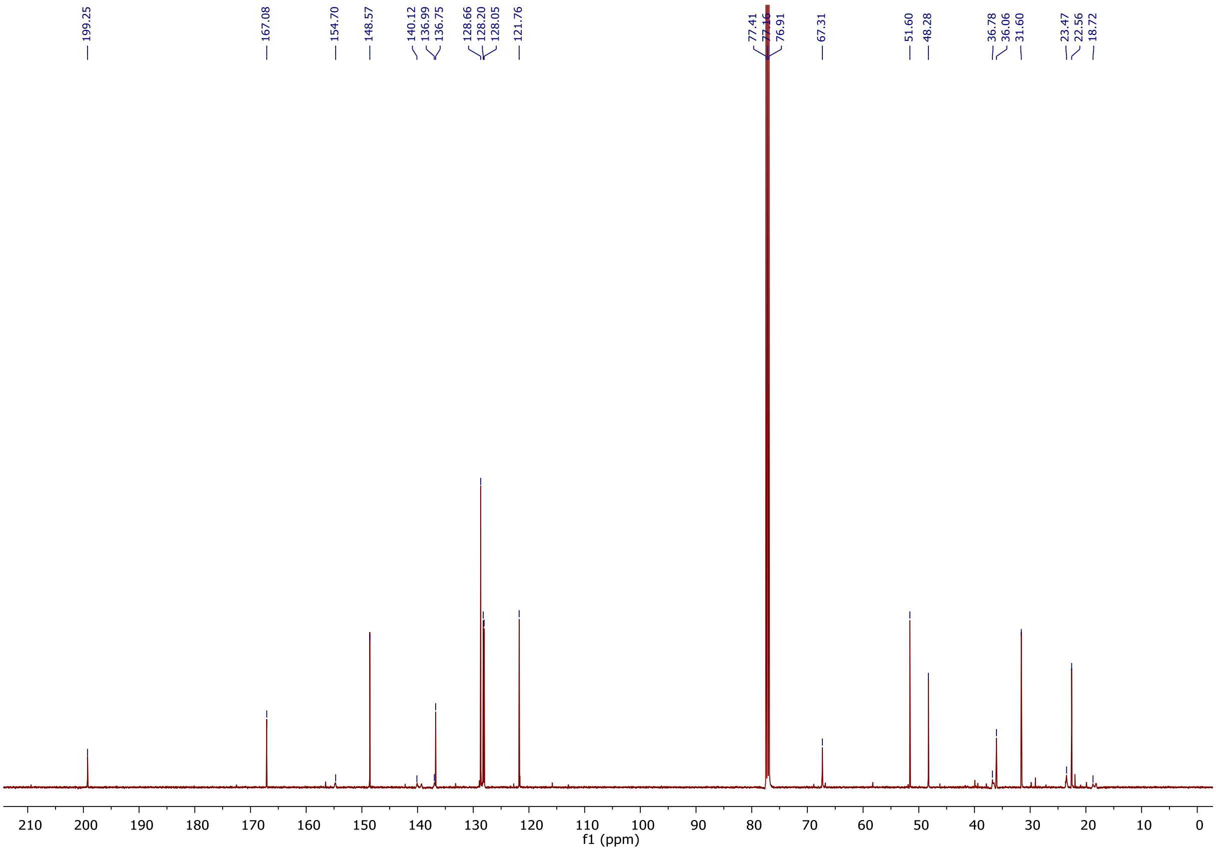

3-(1-Cyclopropyl-1-hydroxy-2-(trimethylsilyl)ethyl)dihydrofuran-2(3*H*)-one

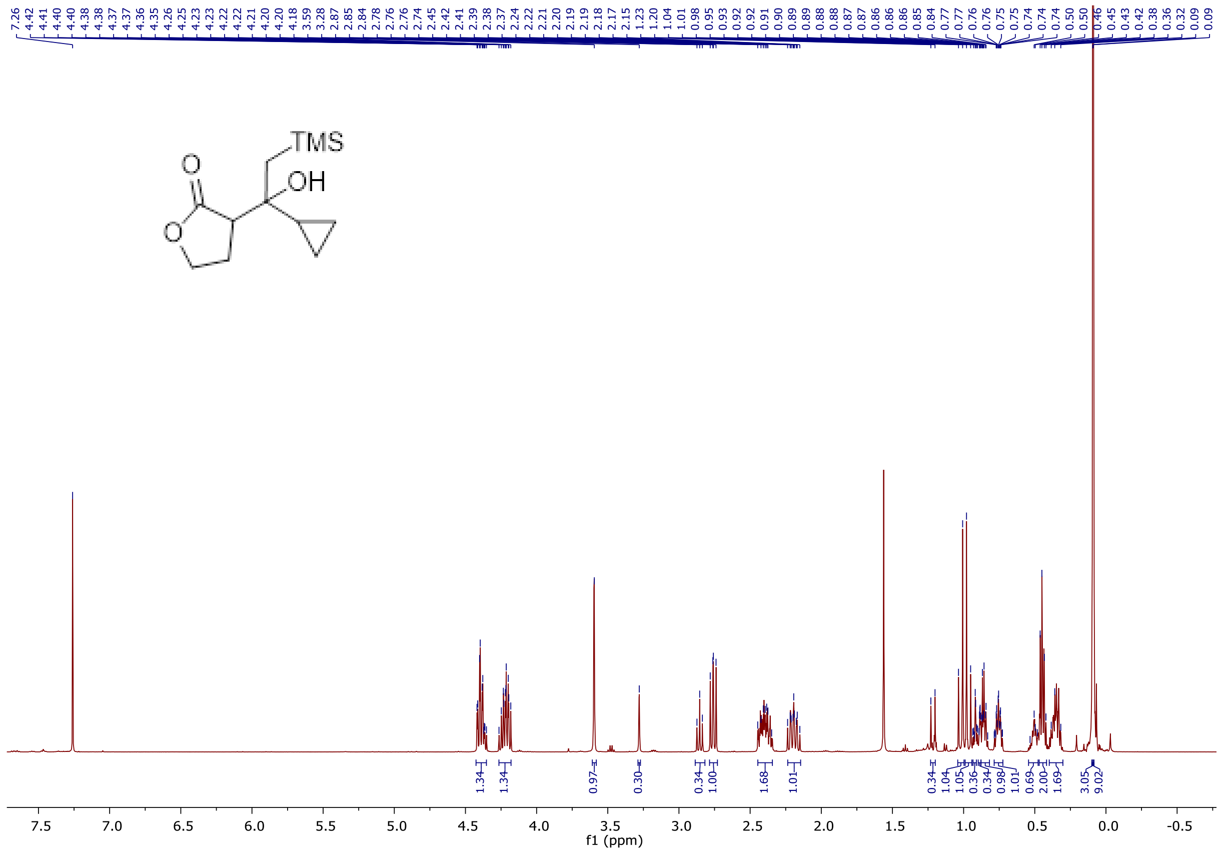

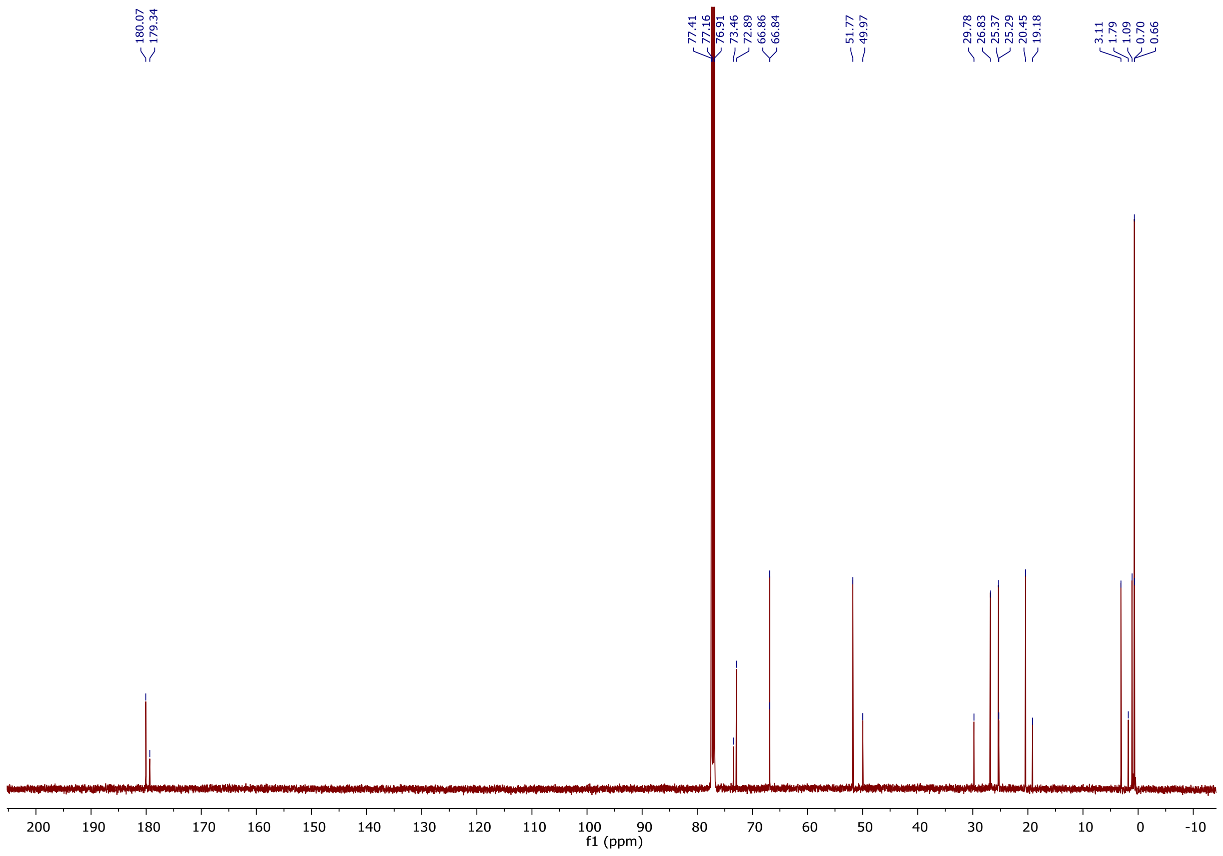

3-(1-Cyclopropylvinyl)dihydrofuran-2(3*H*)-one

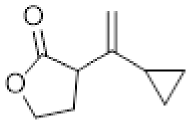

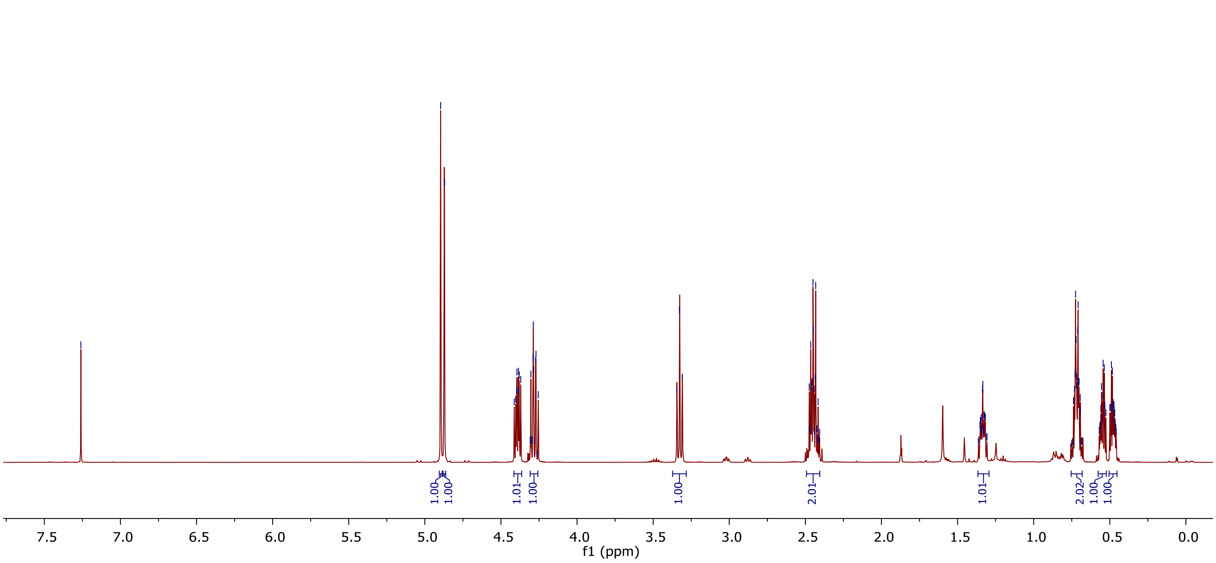

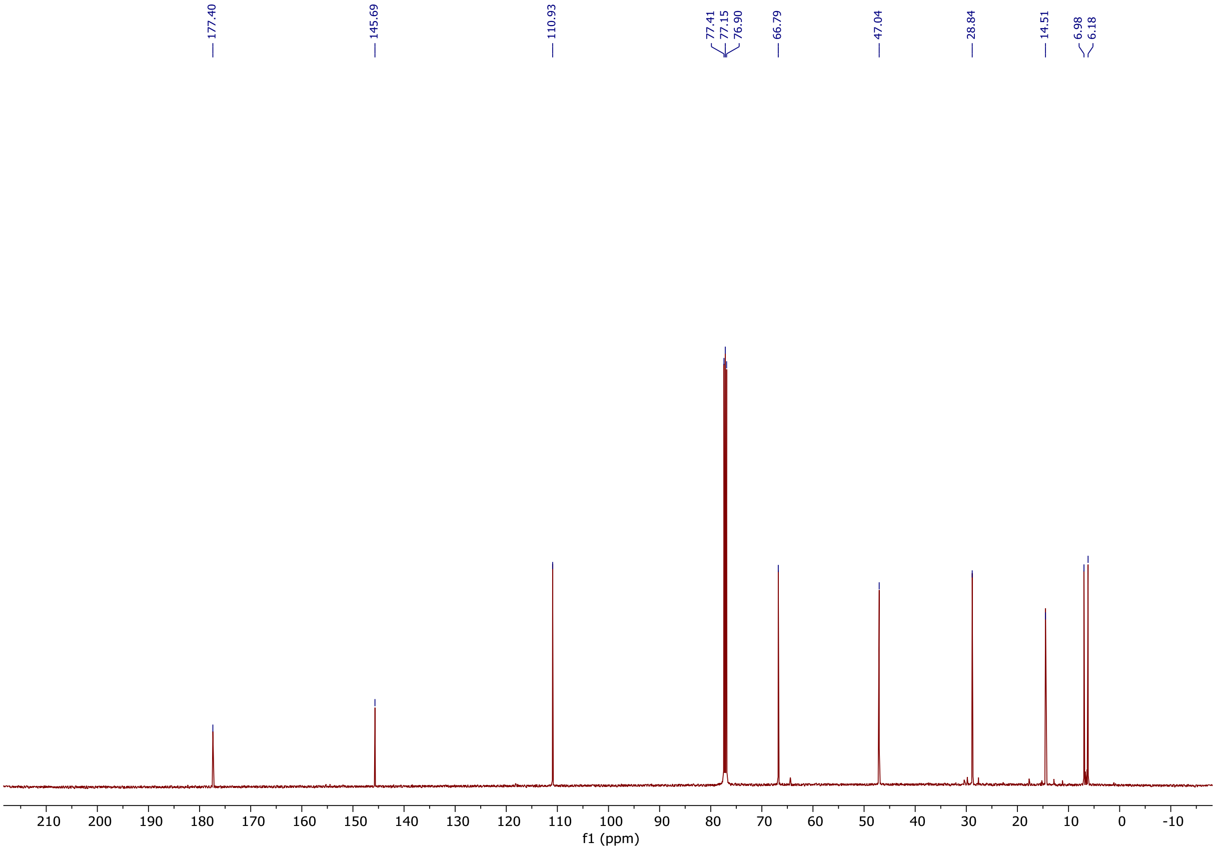

Benzyl (3-acetyl-4-cyclopropylpent-4-en-1-yl)((perfluorobenzoyl)oxy)carbamate (3e)

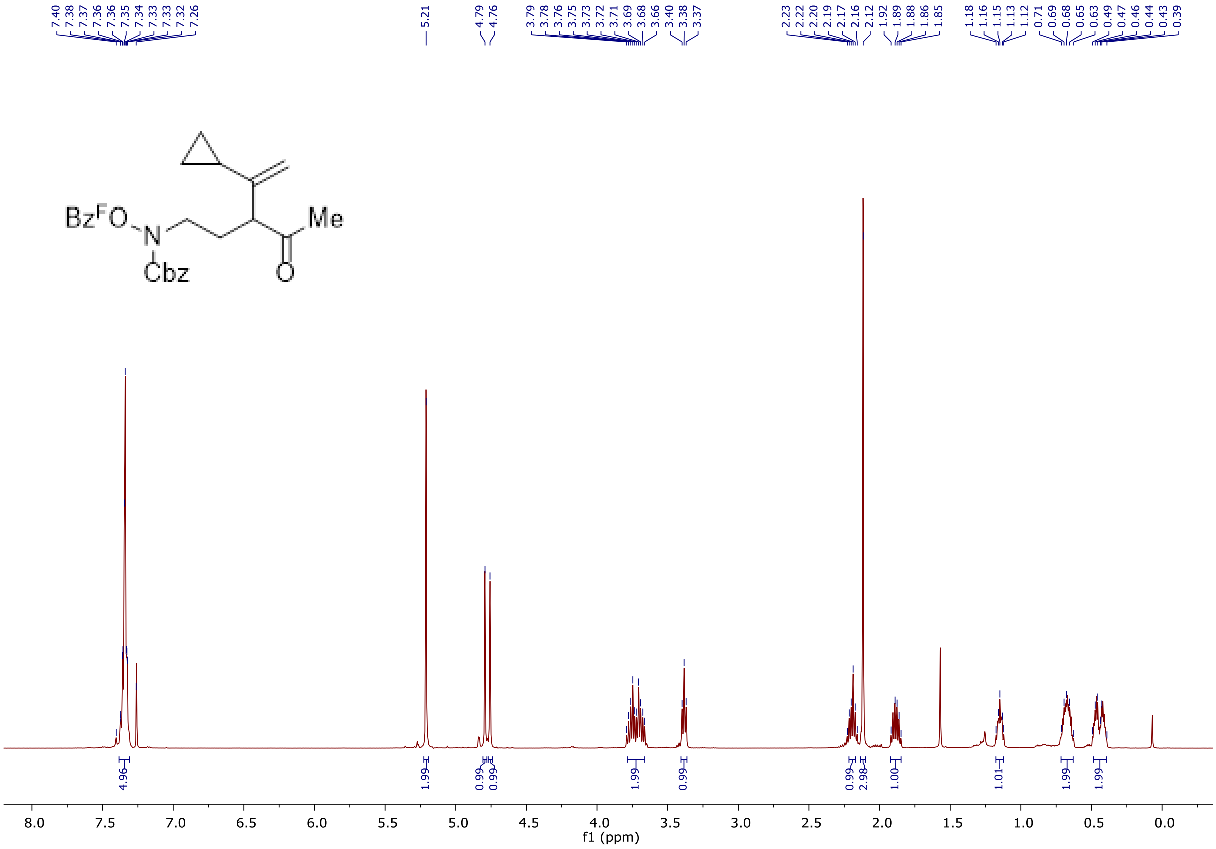

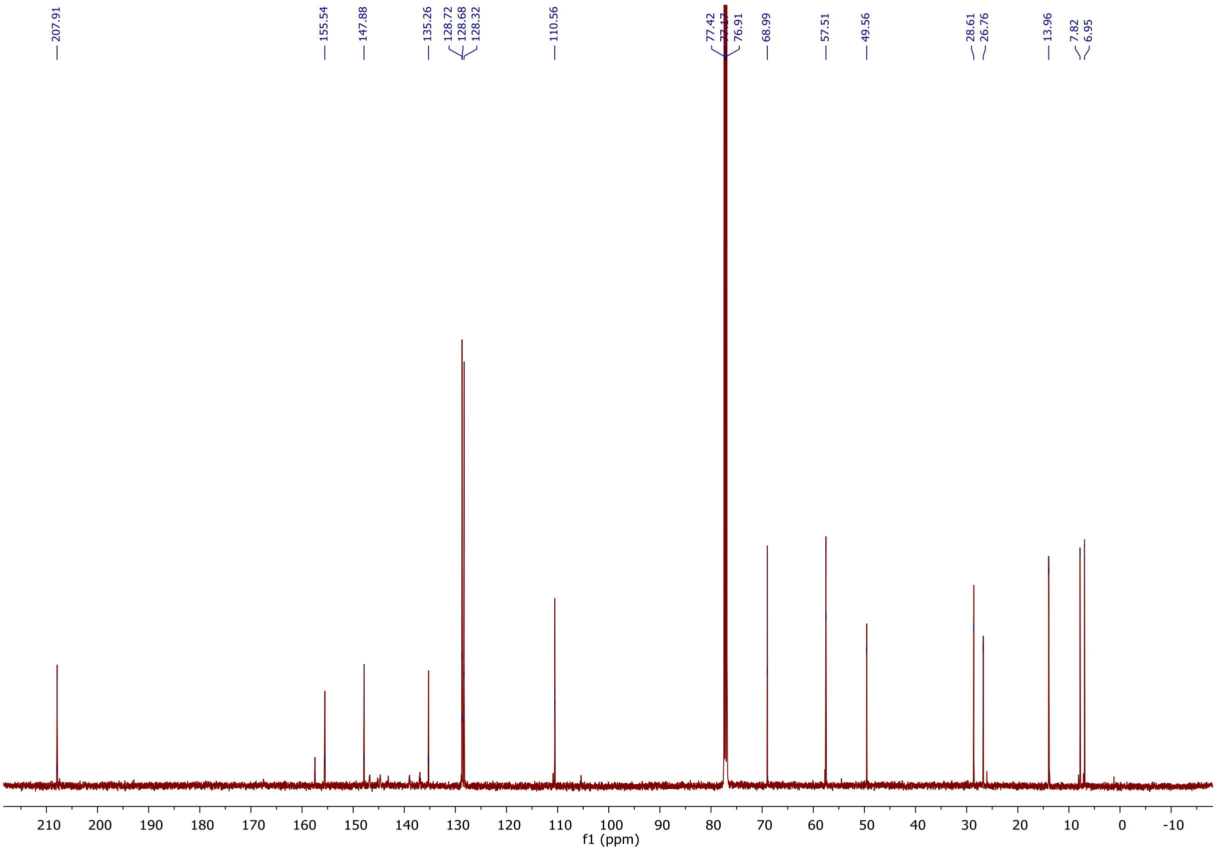

Benzyl 4-acetyl-6-cyclopropyl-3,4-dihydropyridine-1(2*H*)-carboxylate (4e-C2-C3)

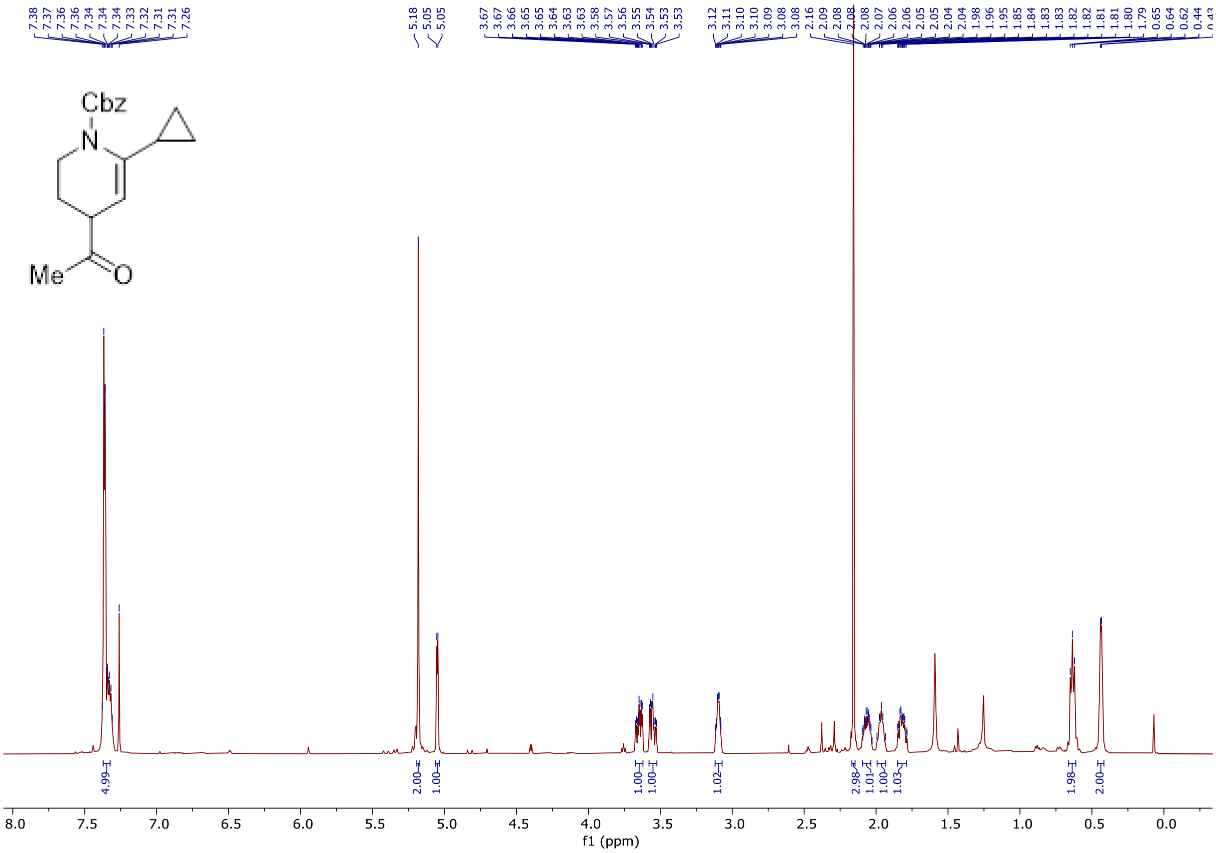

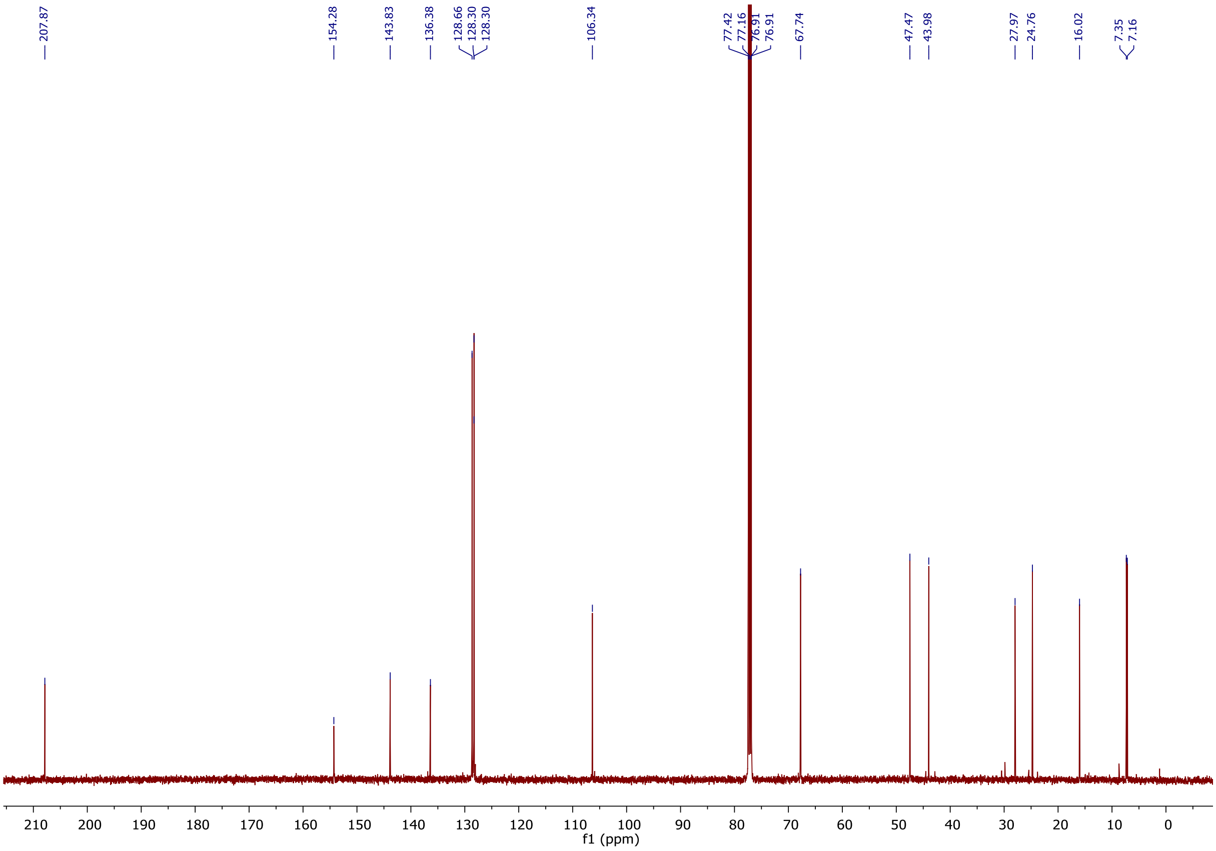

Benzyl 4-acetyl-6-cyclopropyl-3,6-dihydropyridine-1(2*H*)-carboxylate (4e-C3-C4)

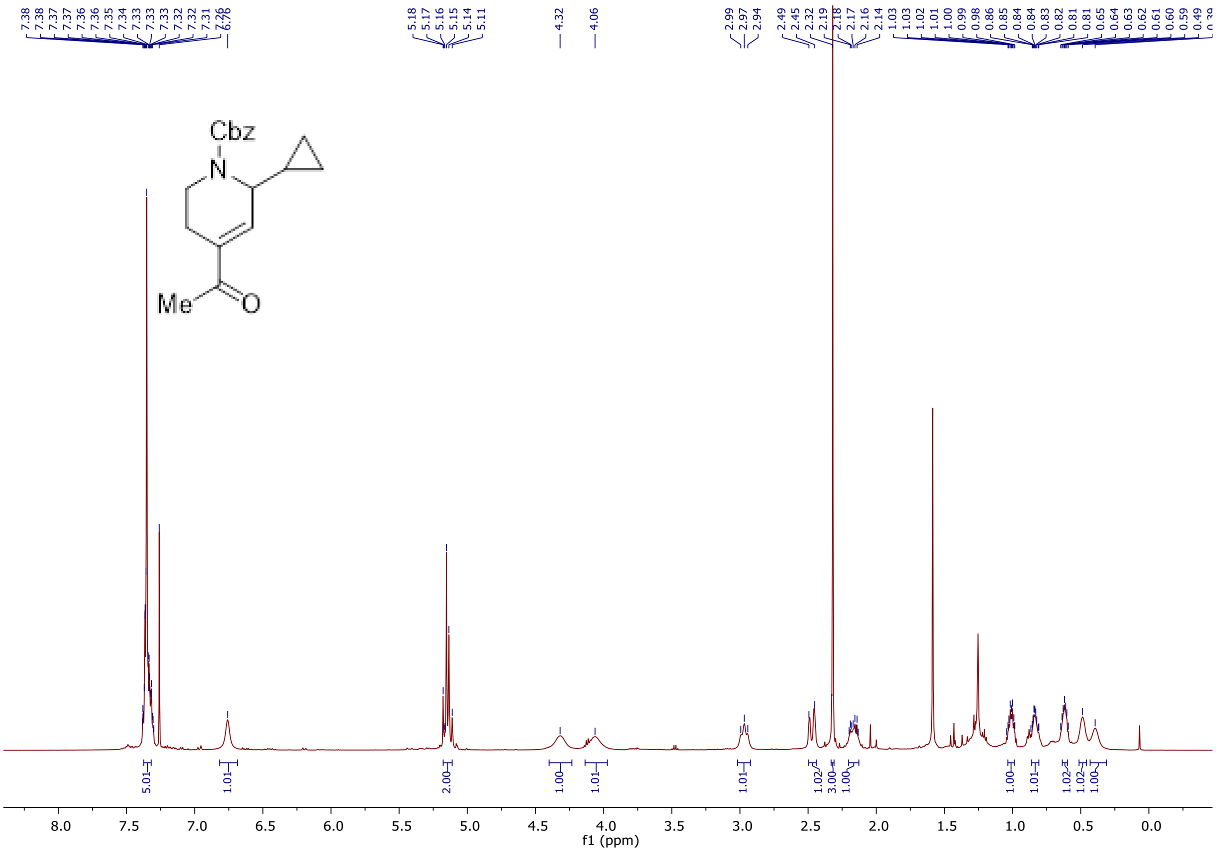

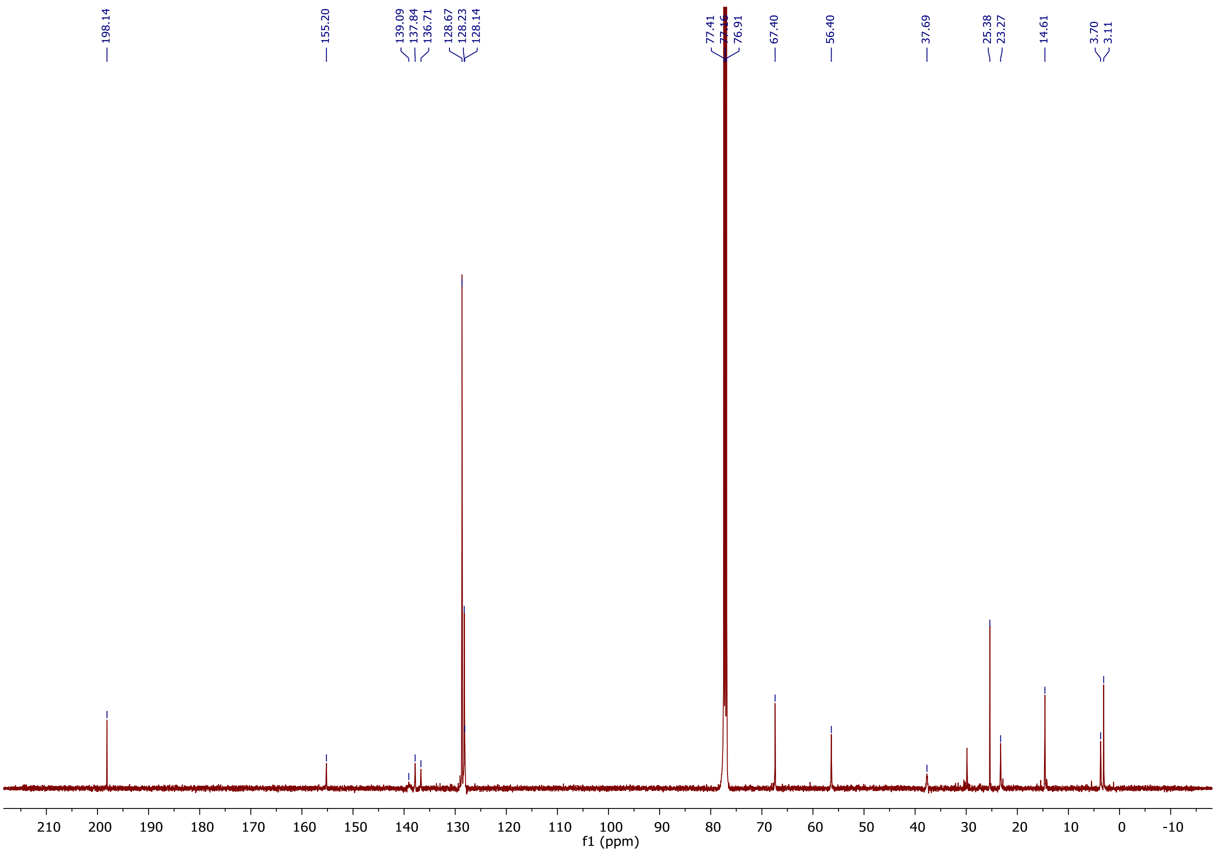

4-Phenyl-1-(trimethylsilyl)butan-2-one

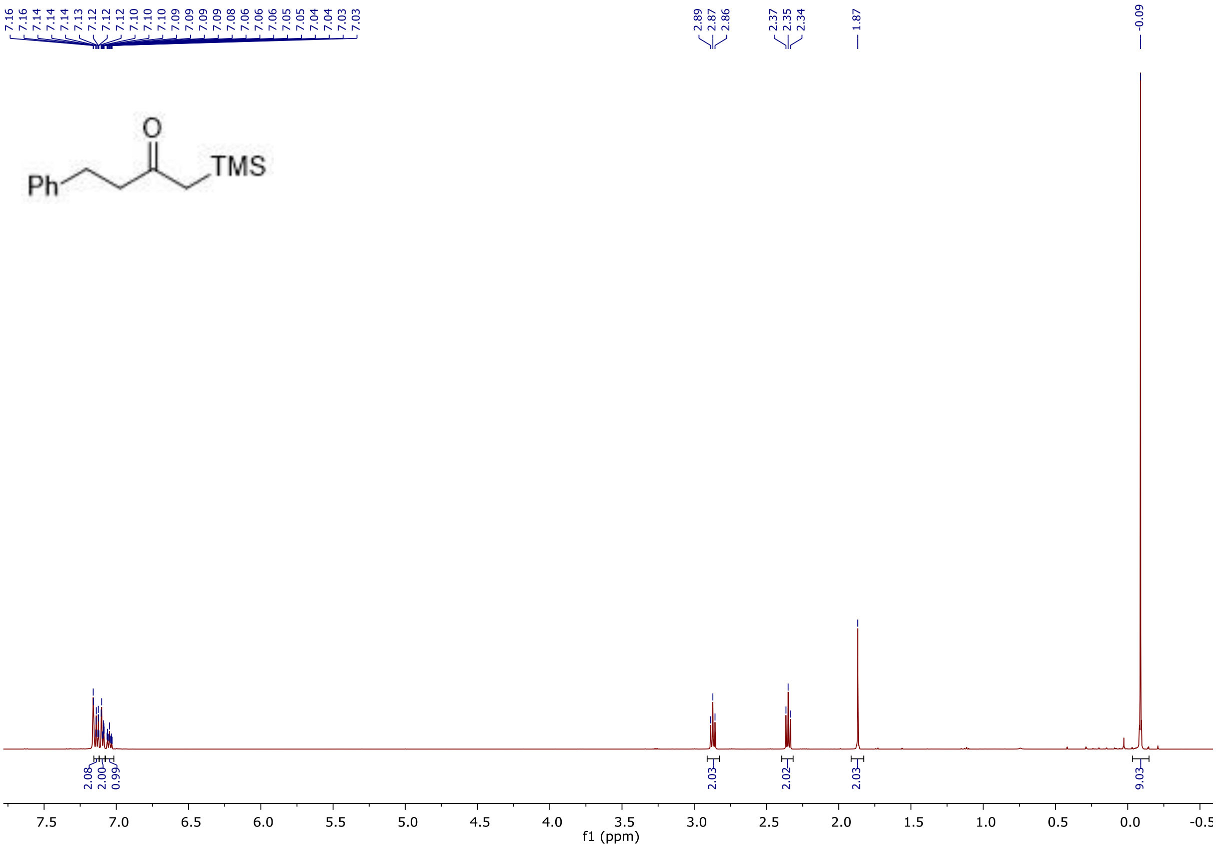

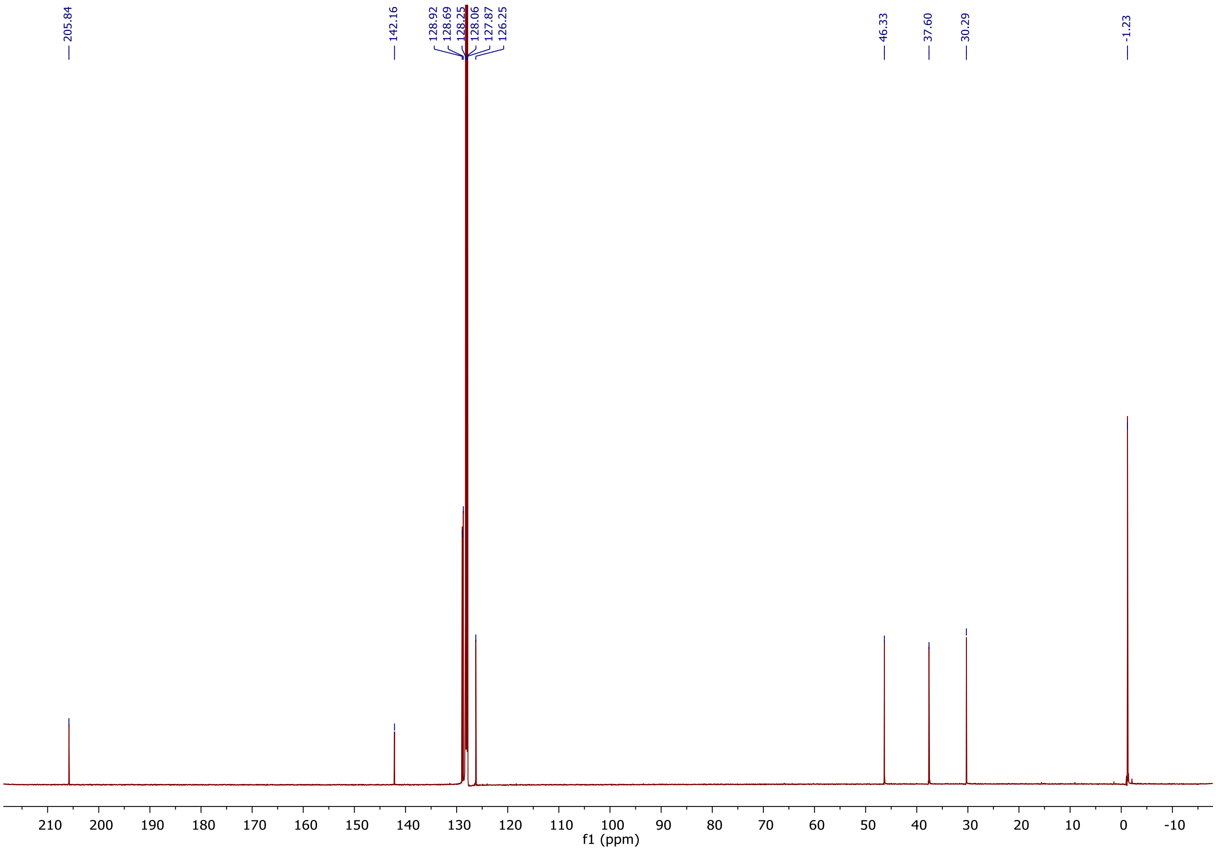

3-(2-Hydroxy-4-phenyl-1-(trimethylsilyl)butan-2-yl)dihydrofuran-2(3*H*)-one

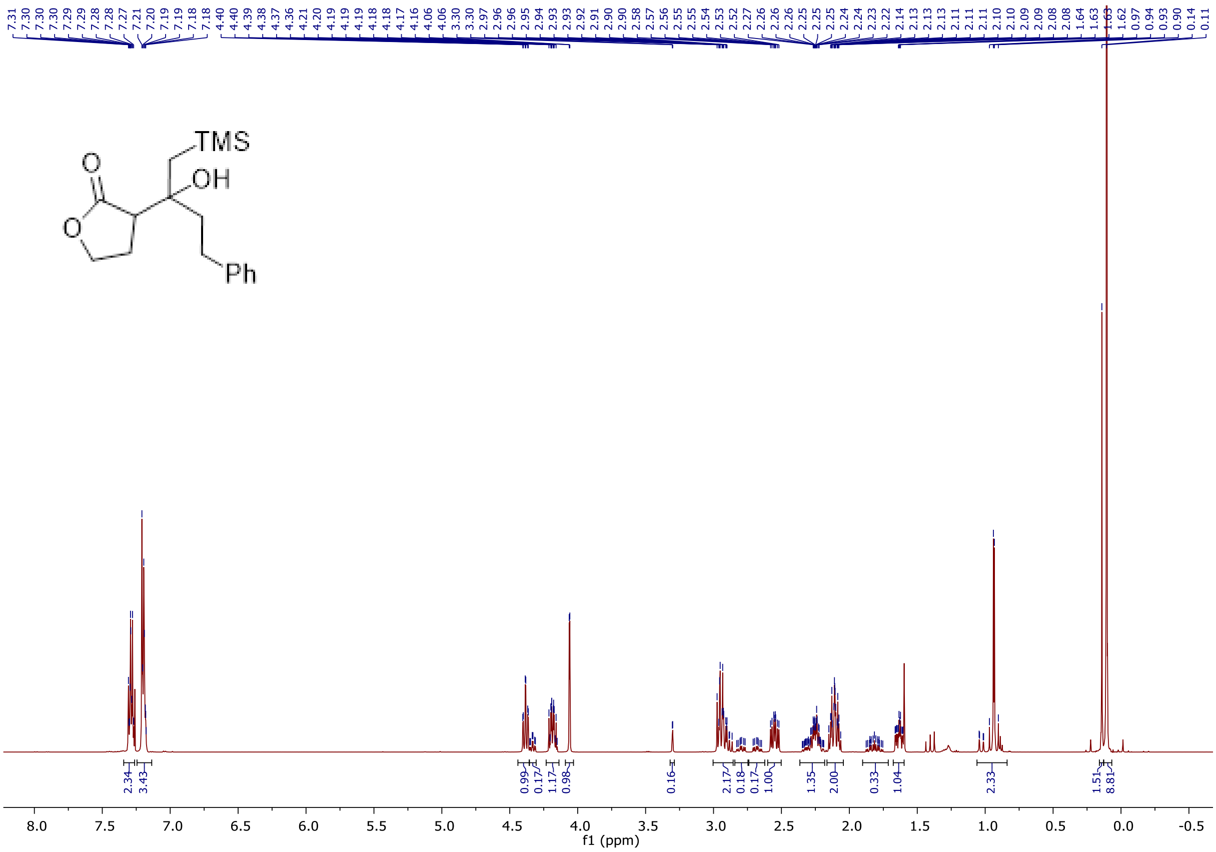

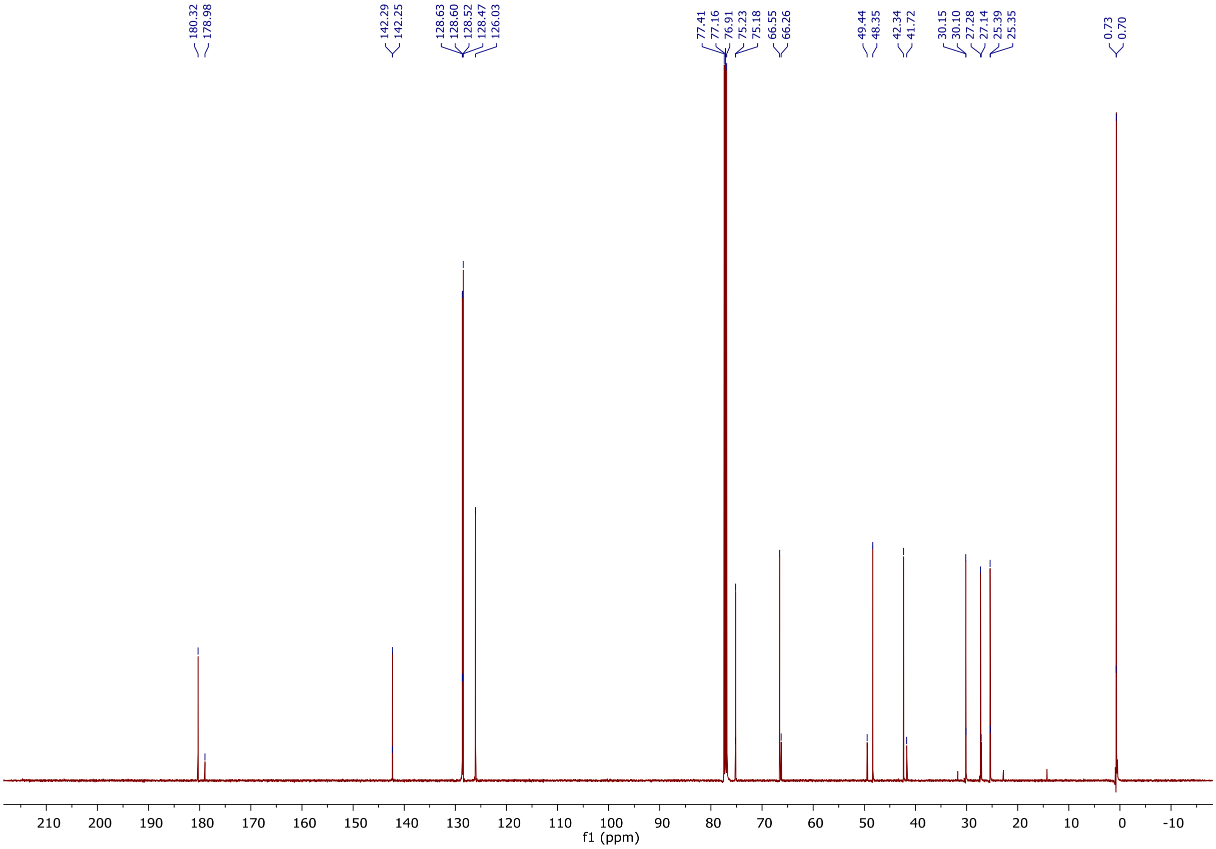

3-(4-Phenylbut-1-en-2-yl)dihydrofuran-2(3*H*)-one

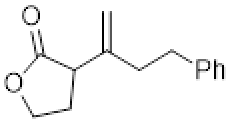

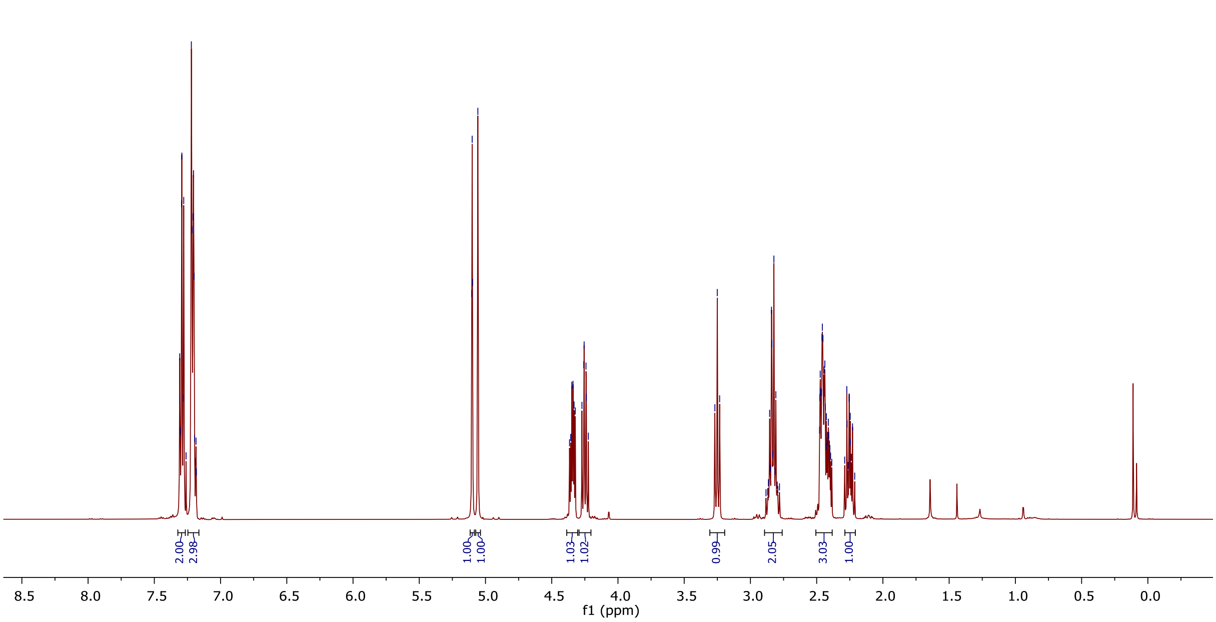

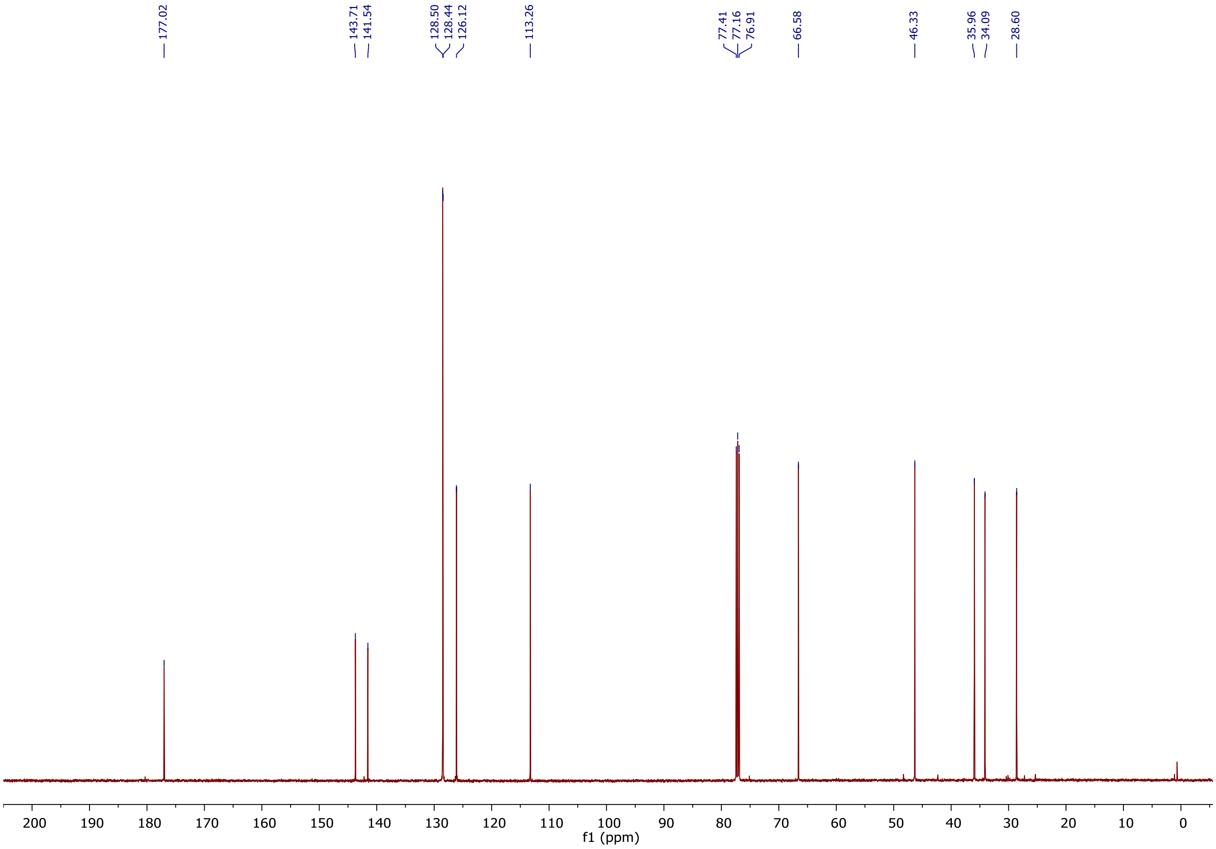

3-(2-Hydroxyethyl)-4-methylene-6-phenylhexan-2-one

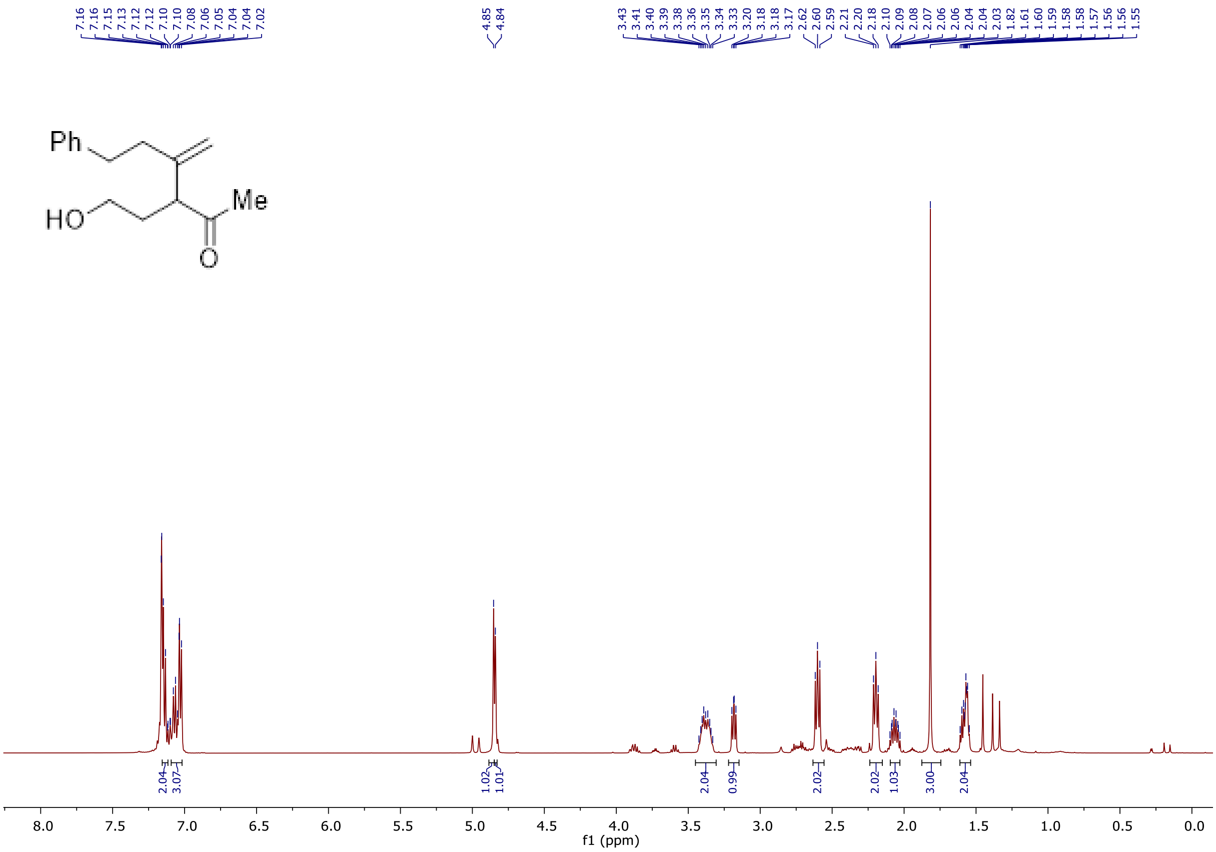

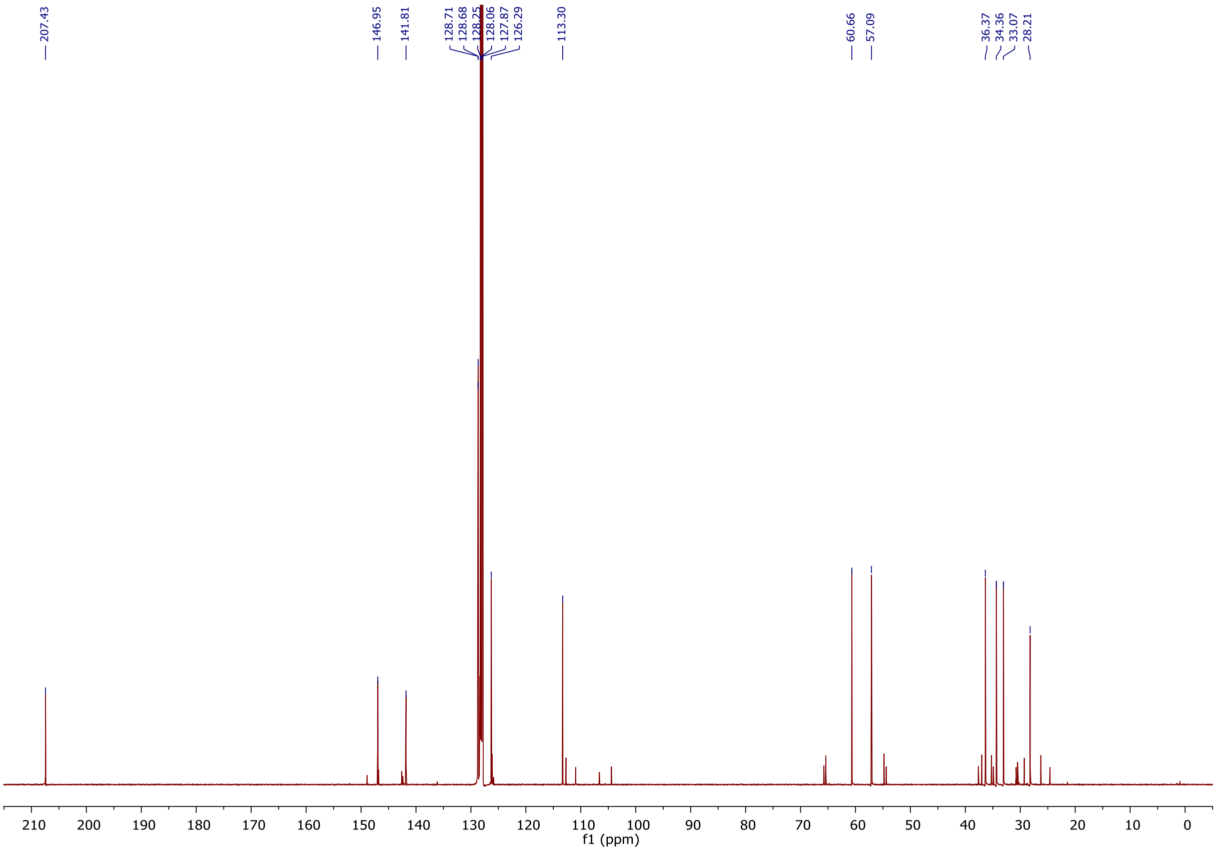

Benzyl (3-acetyl-4-methylene-6-phenylhexyl)((perfluorobenzoyl)oxy)carbamate (3f)

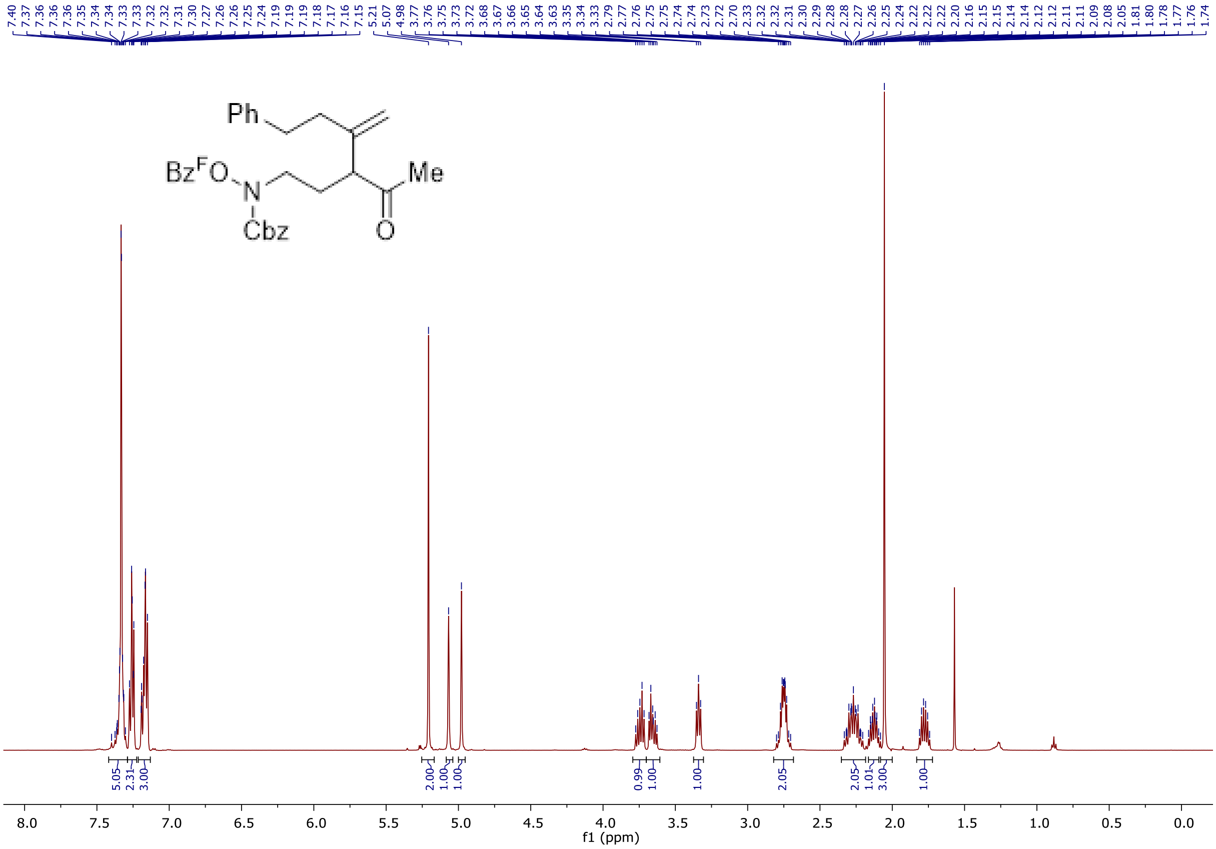

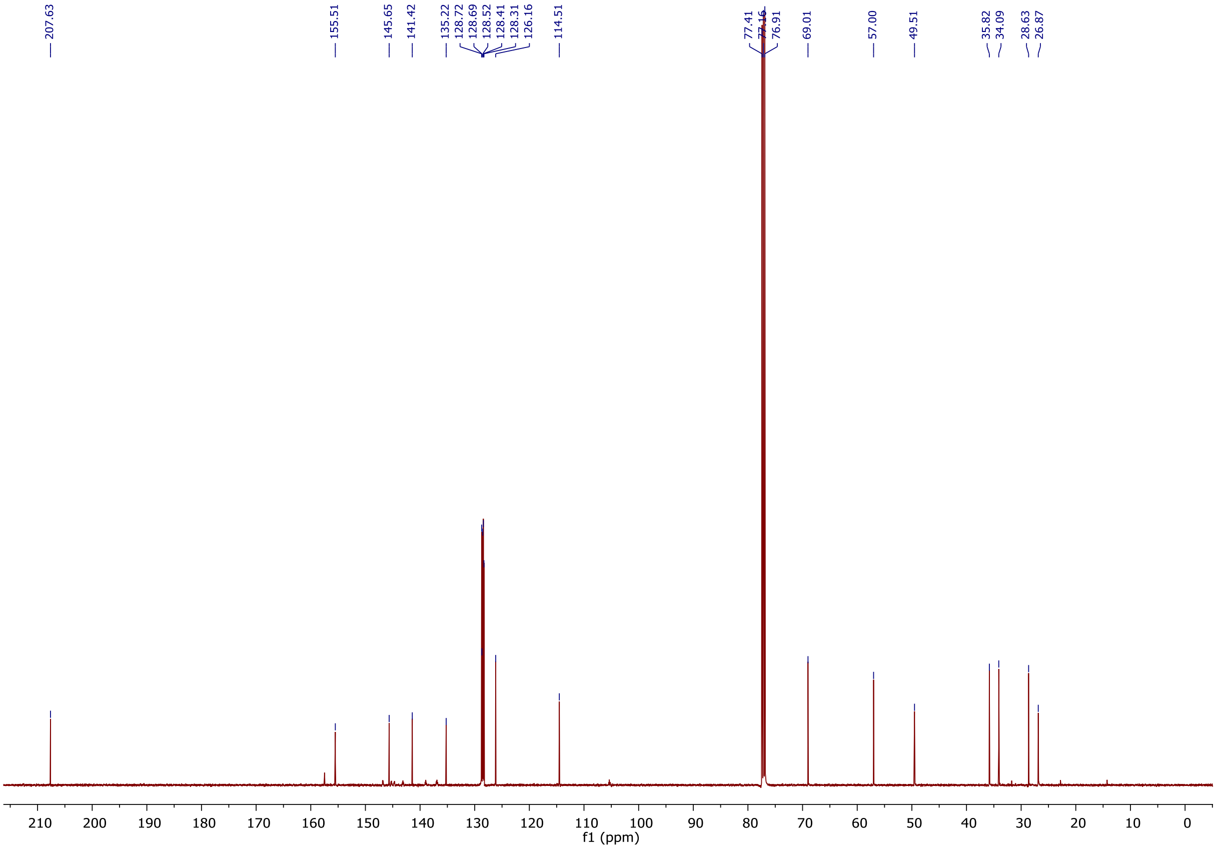

Benzyl 4-acetyl-6-phenethyl-3,4-dihydropyridine-1(2*H*)-carboxylate (4f-C2-C3)

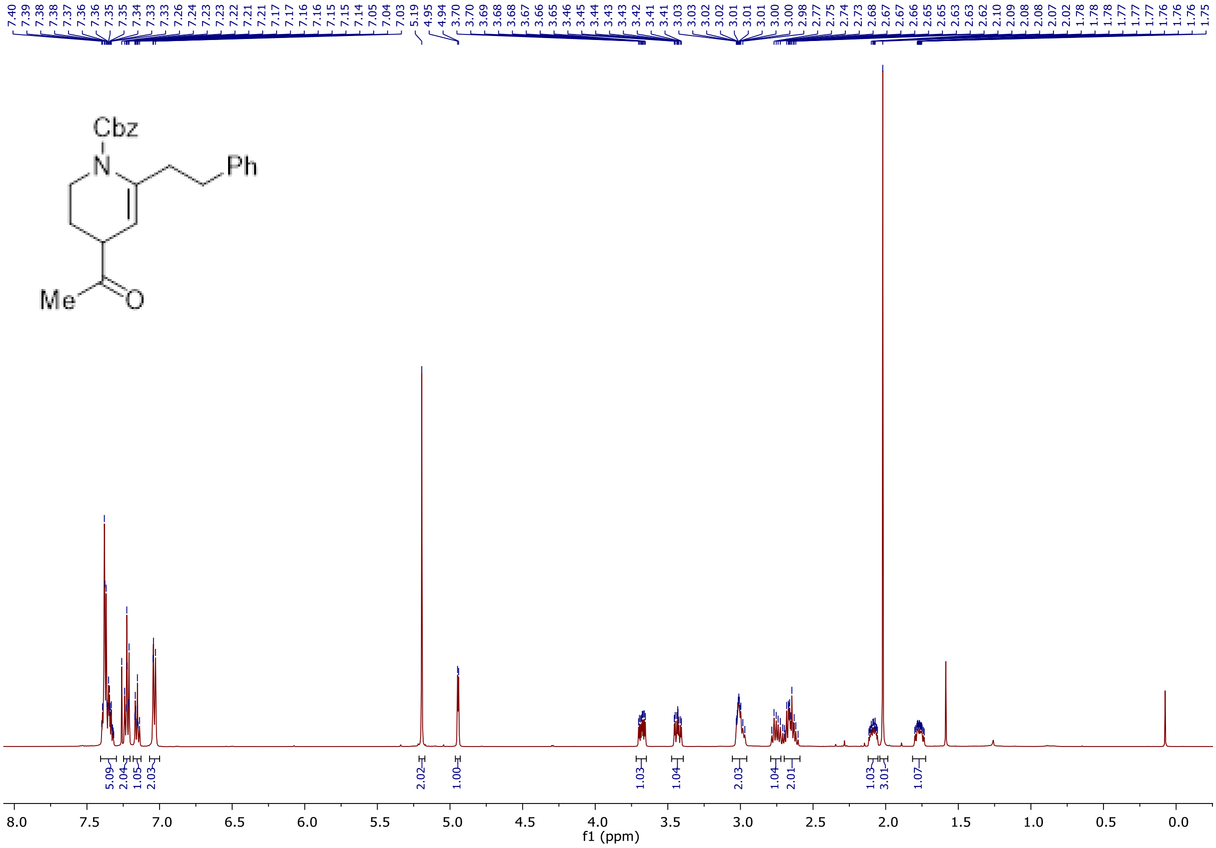

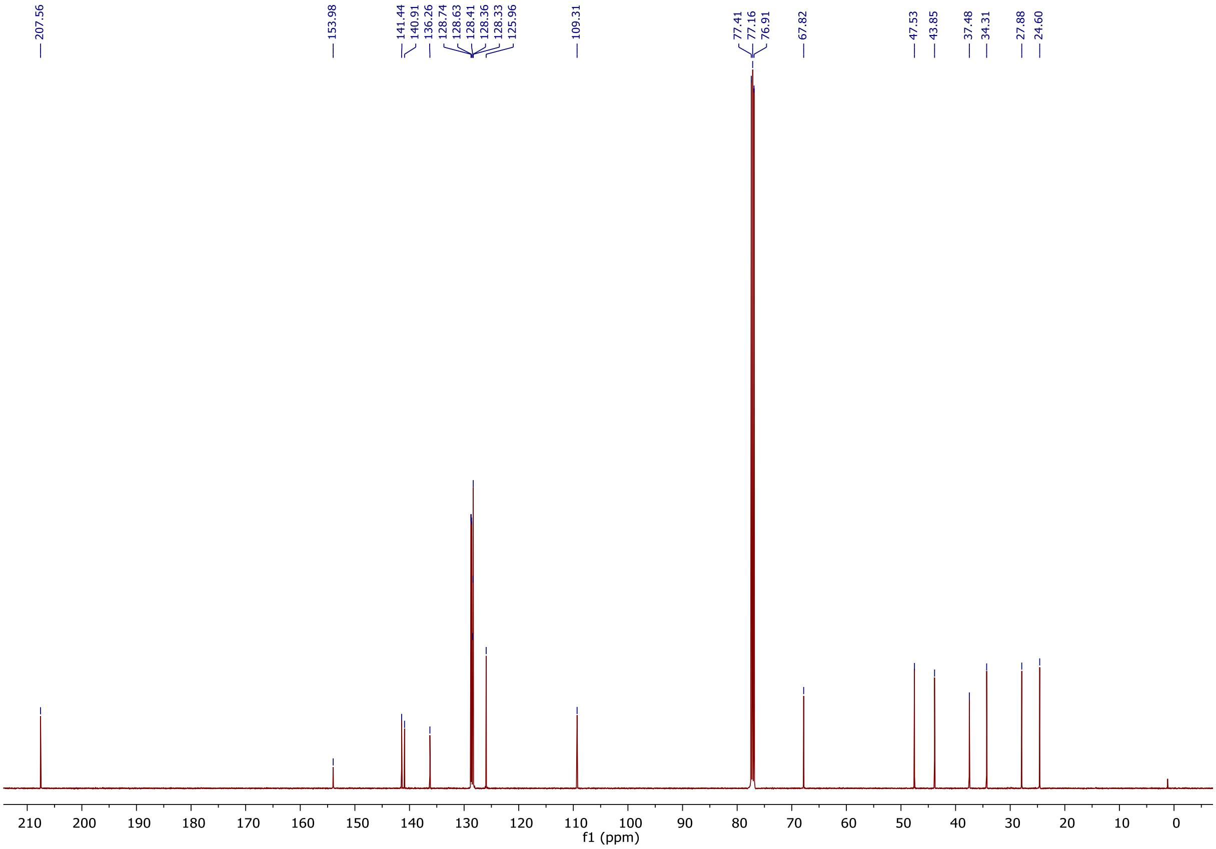

Benzyl 4-acetyl-6-phenethyl-3,6-dihydropyridine-1(2*H*)-carboxylate (4f-C3-C4)

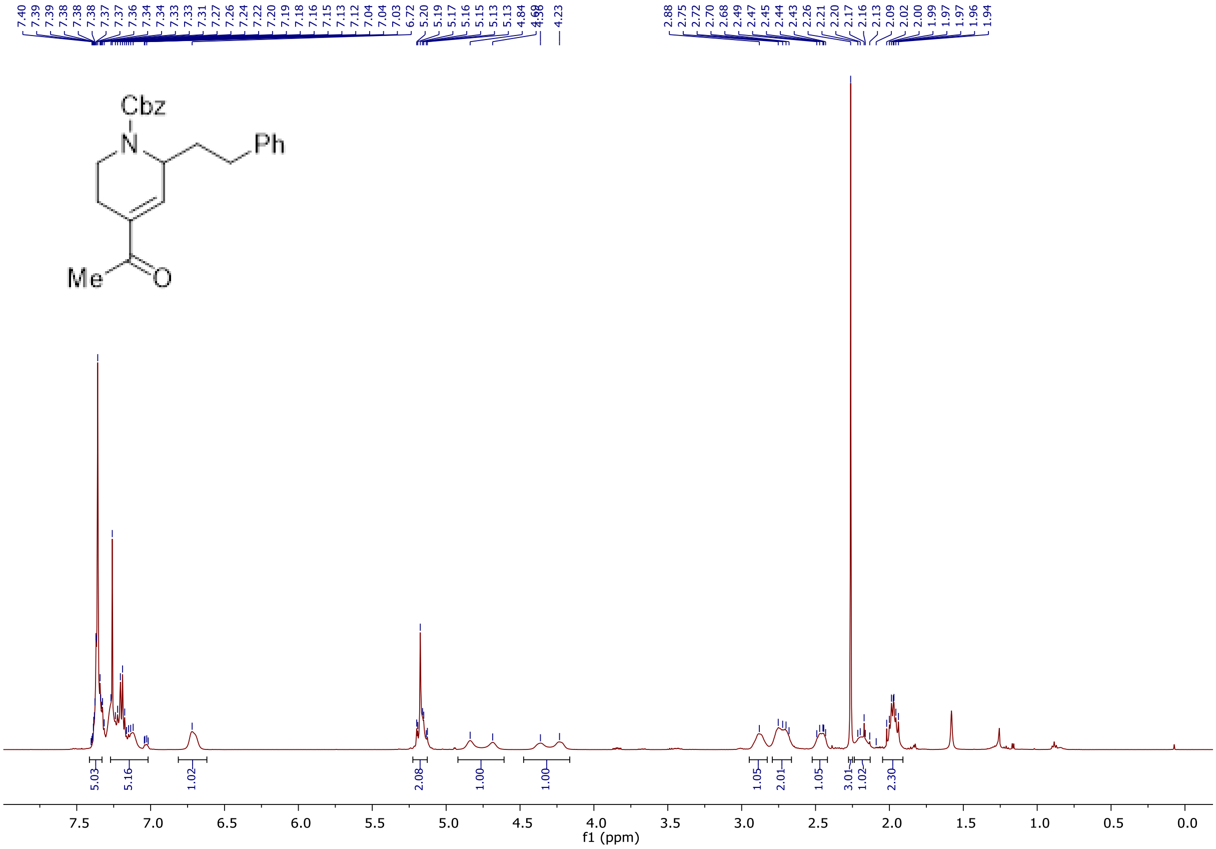

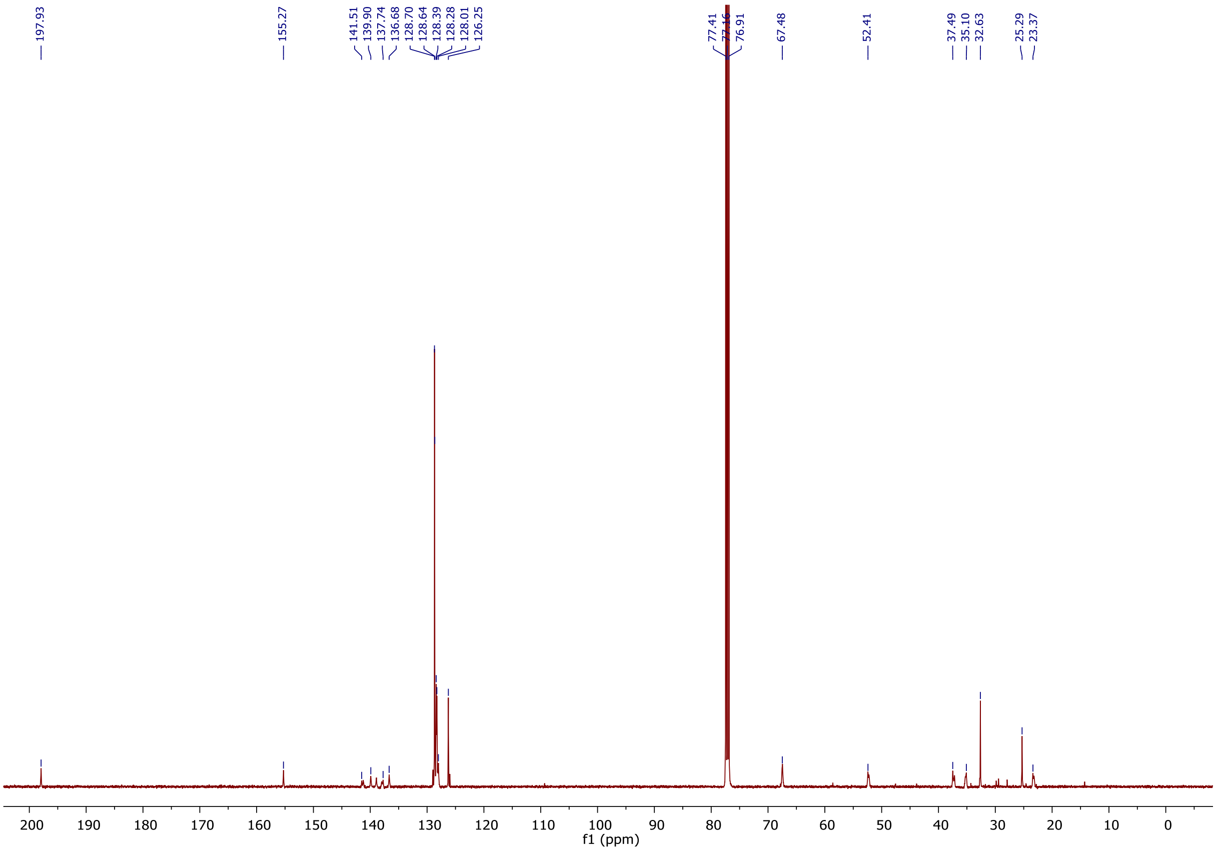

Methyl (4*S*)-4-((*tert*-butyldimethylsilyl)oxy)-2-(prop-1-en-2-yl)pentanoate

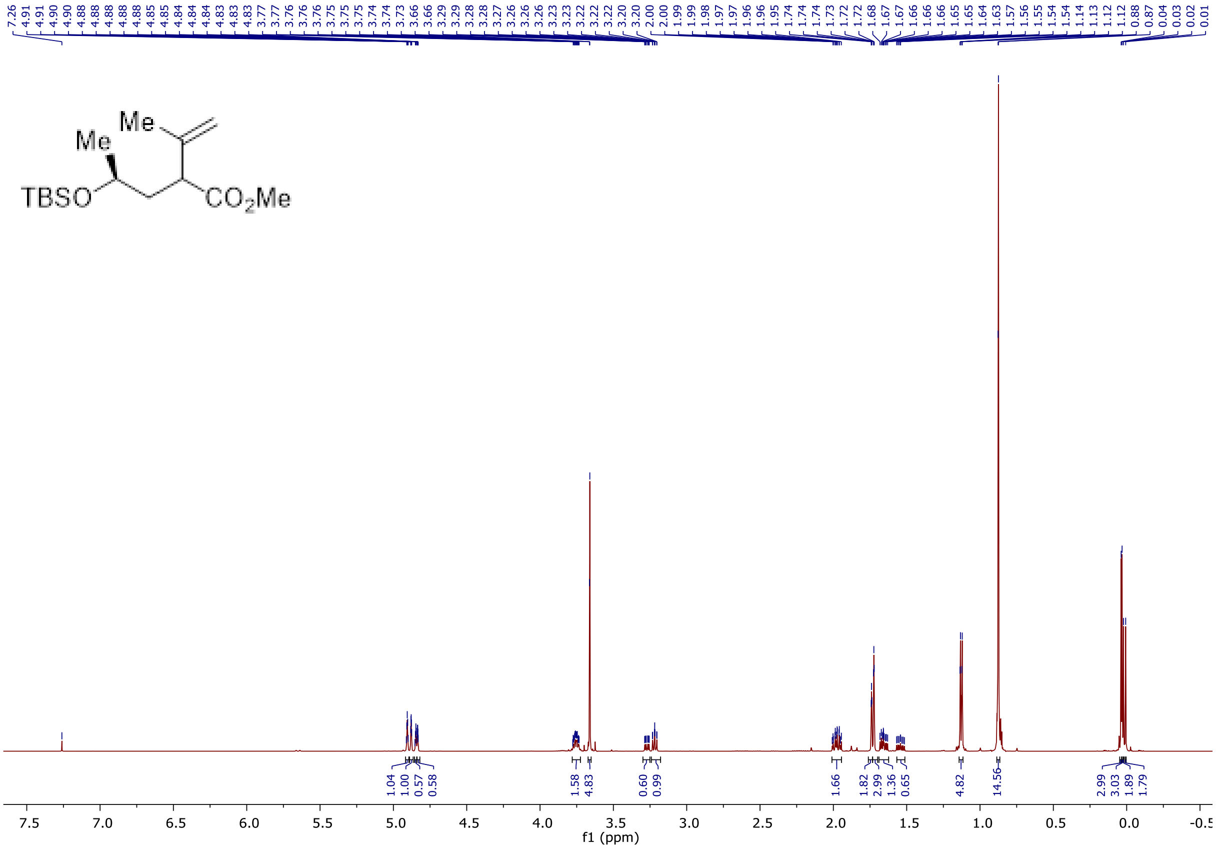

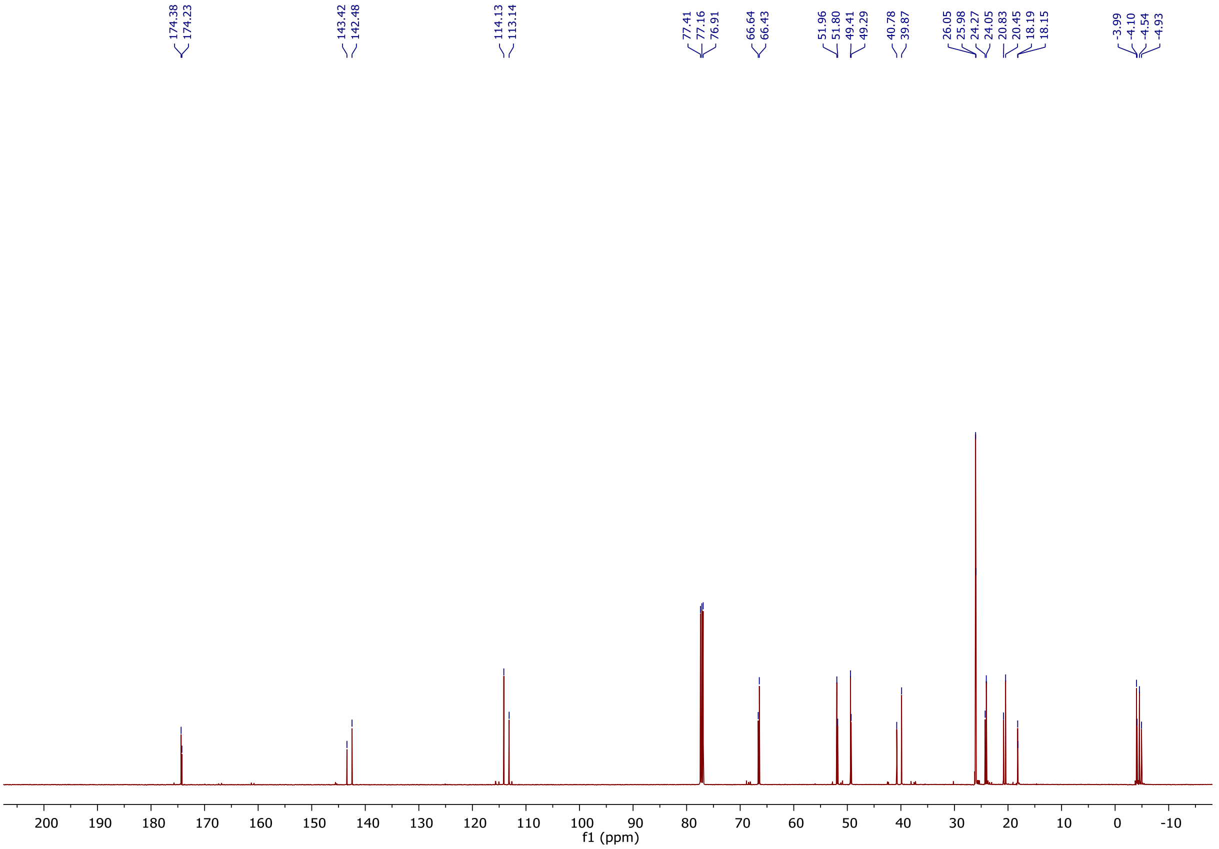

(5*S*)-5-((*tert*-Butyldimethylsilyl)oxy)-3-(prop-1-en-2-yl)hexan-2-one

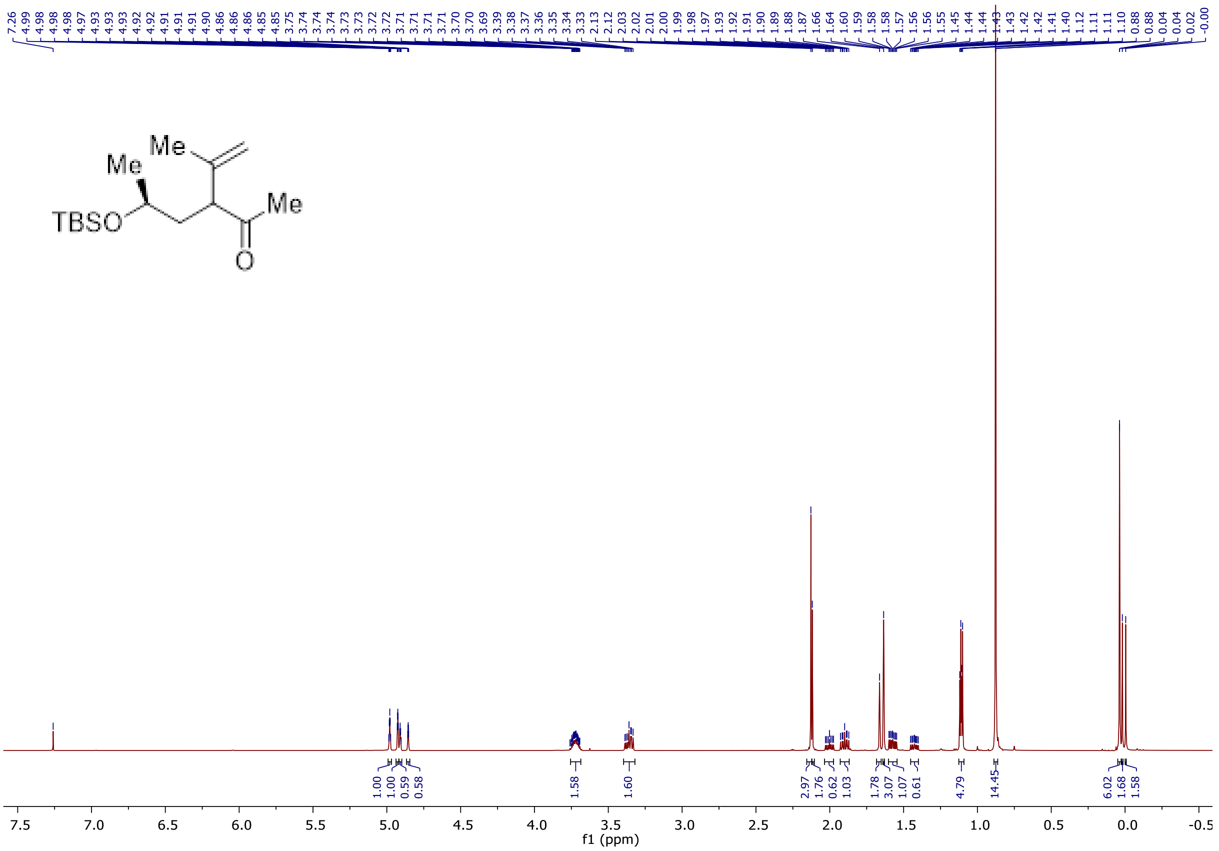

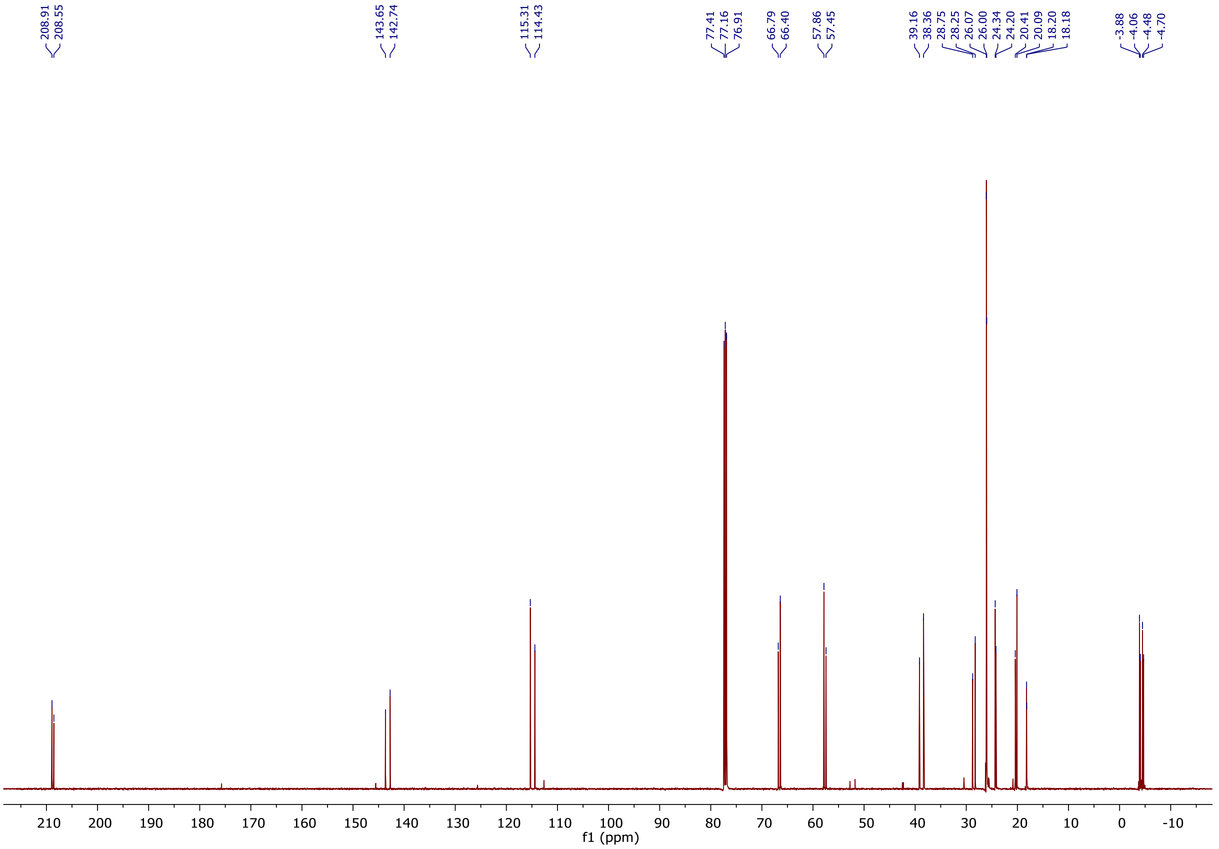

Benzyl ((2*R*)-4-acetyl-5-methylhex-5-en-2-yl)((perfluorobenzoyl)oxy)carbamate (3g)

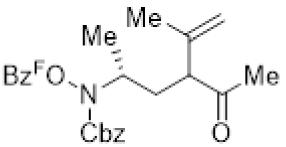

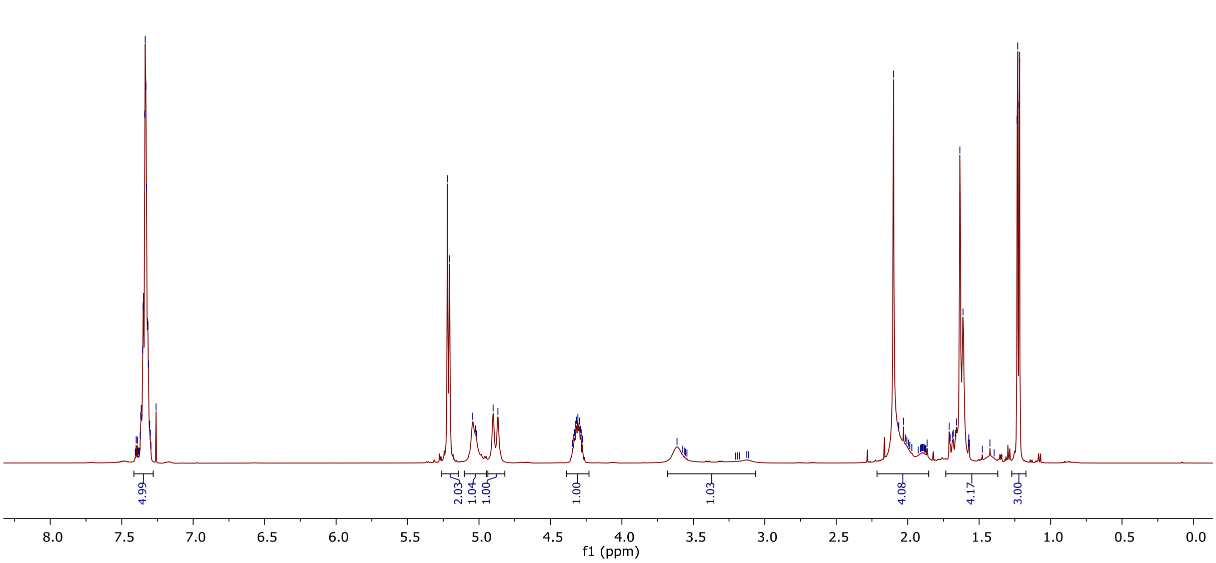

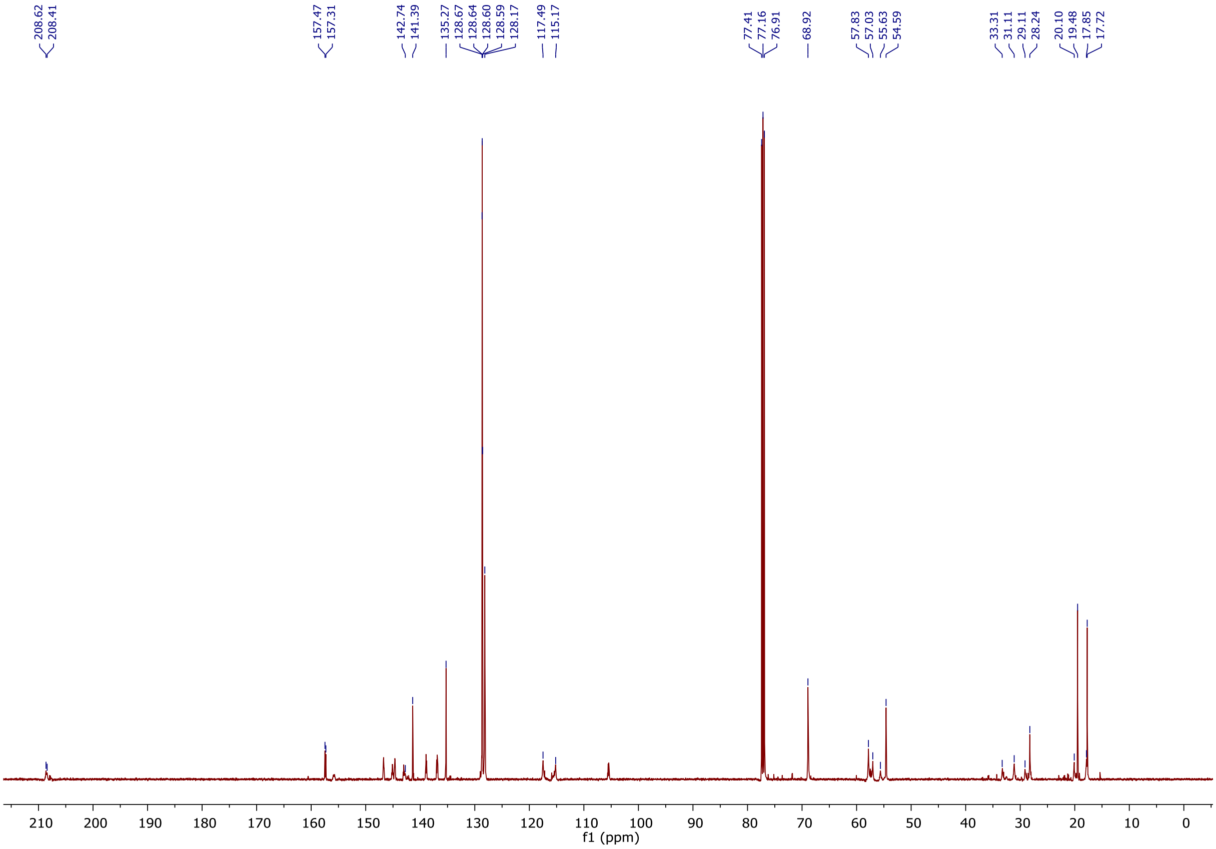

Benzyl (2*R*)-4-acetyl-2,6-dimethyl-3,4-dihydropyridine-1(2*H*)-carboxylate (4g-C2-C3)

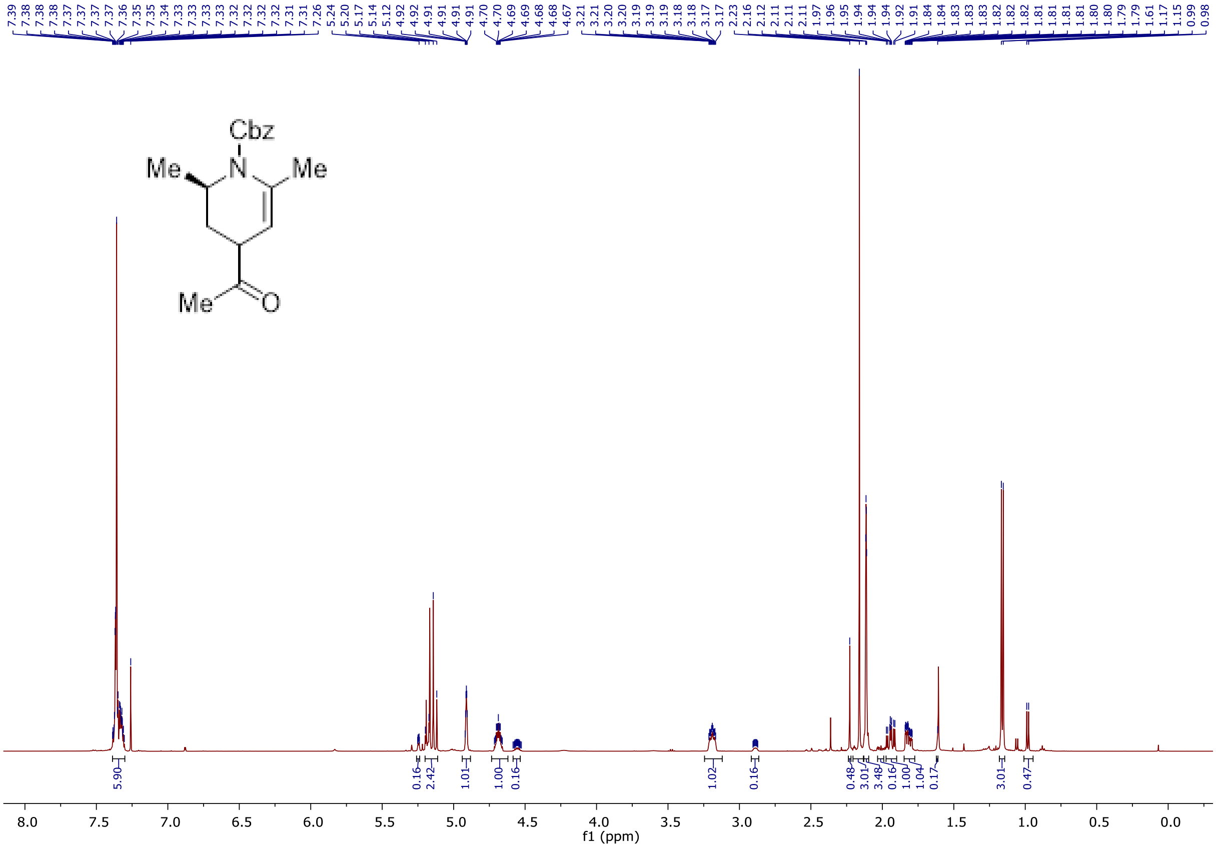

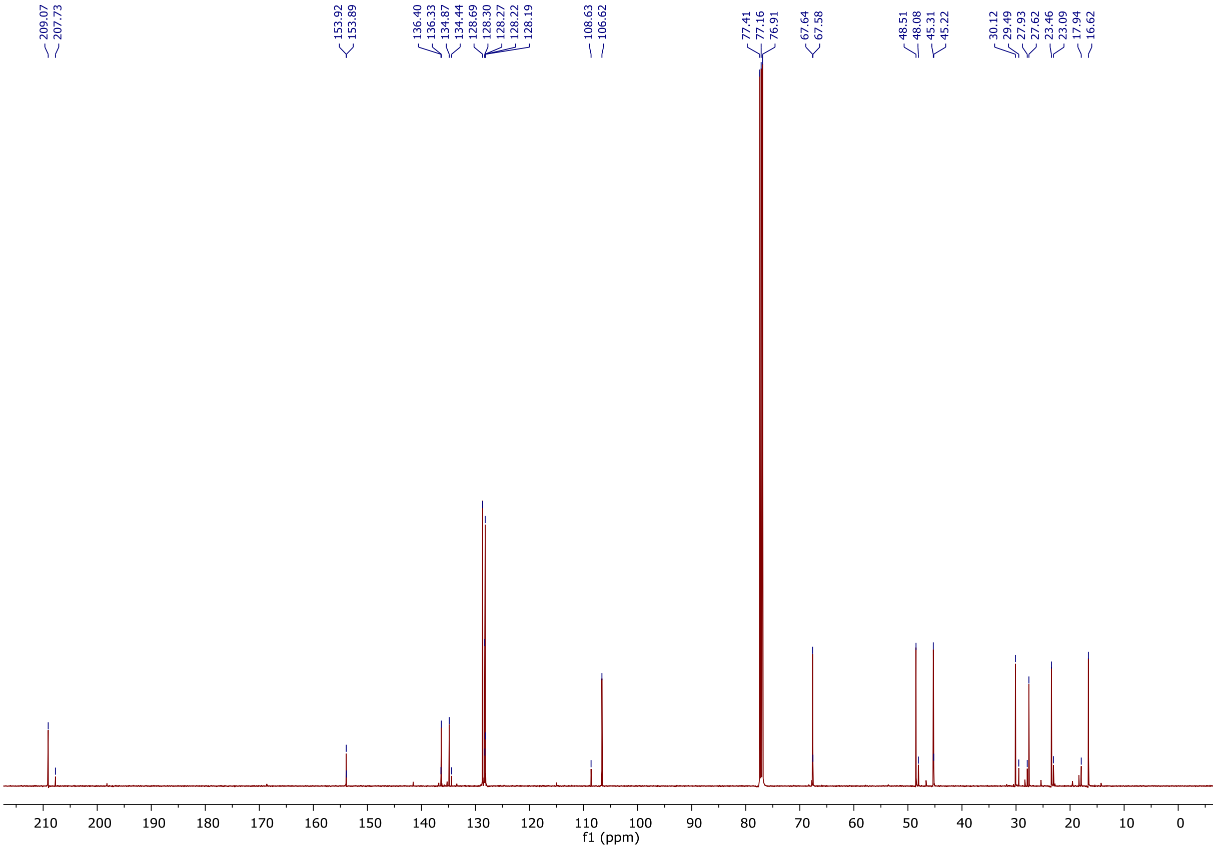

Benzyl (2*R*)-4-acetyl-2,6-dimethyl-3,6-dihydropyridine-1(2*H*)-carboxylate (4g-C3-C4)

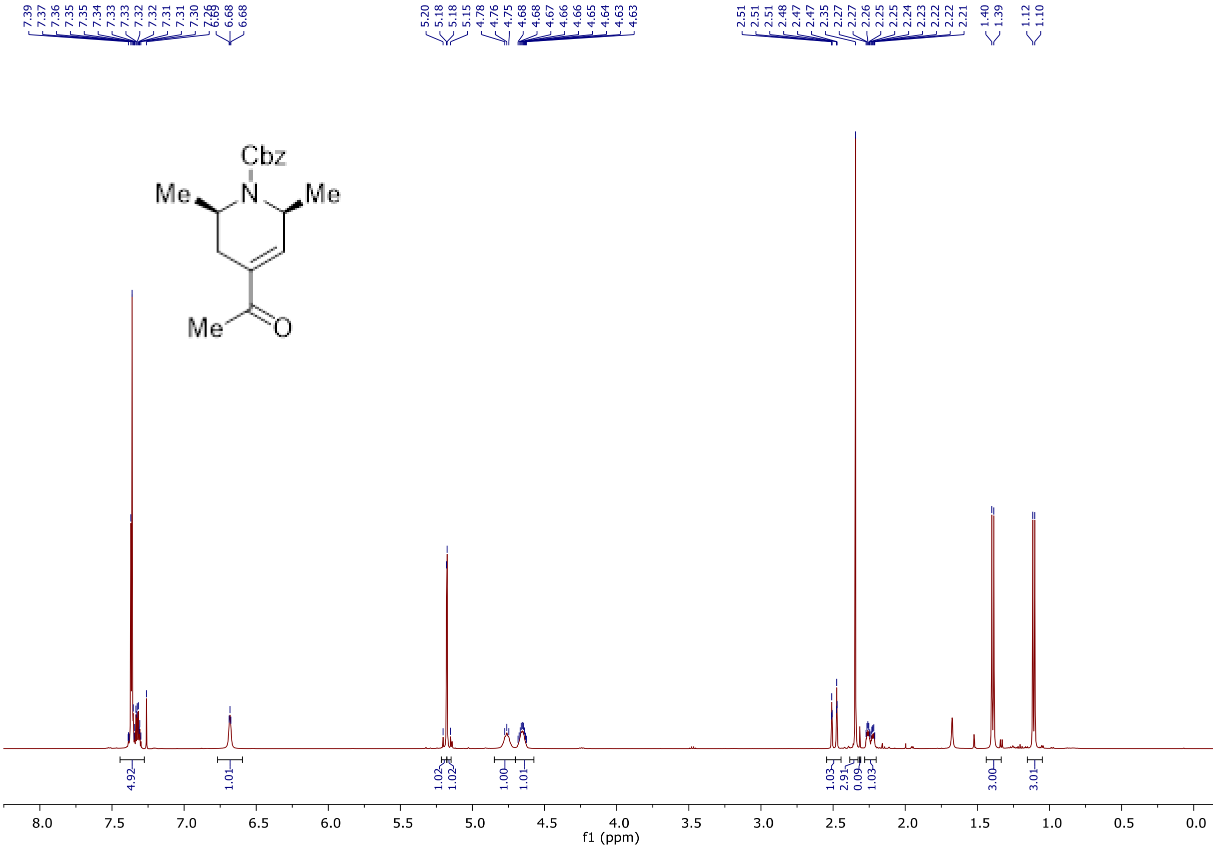

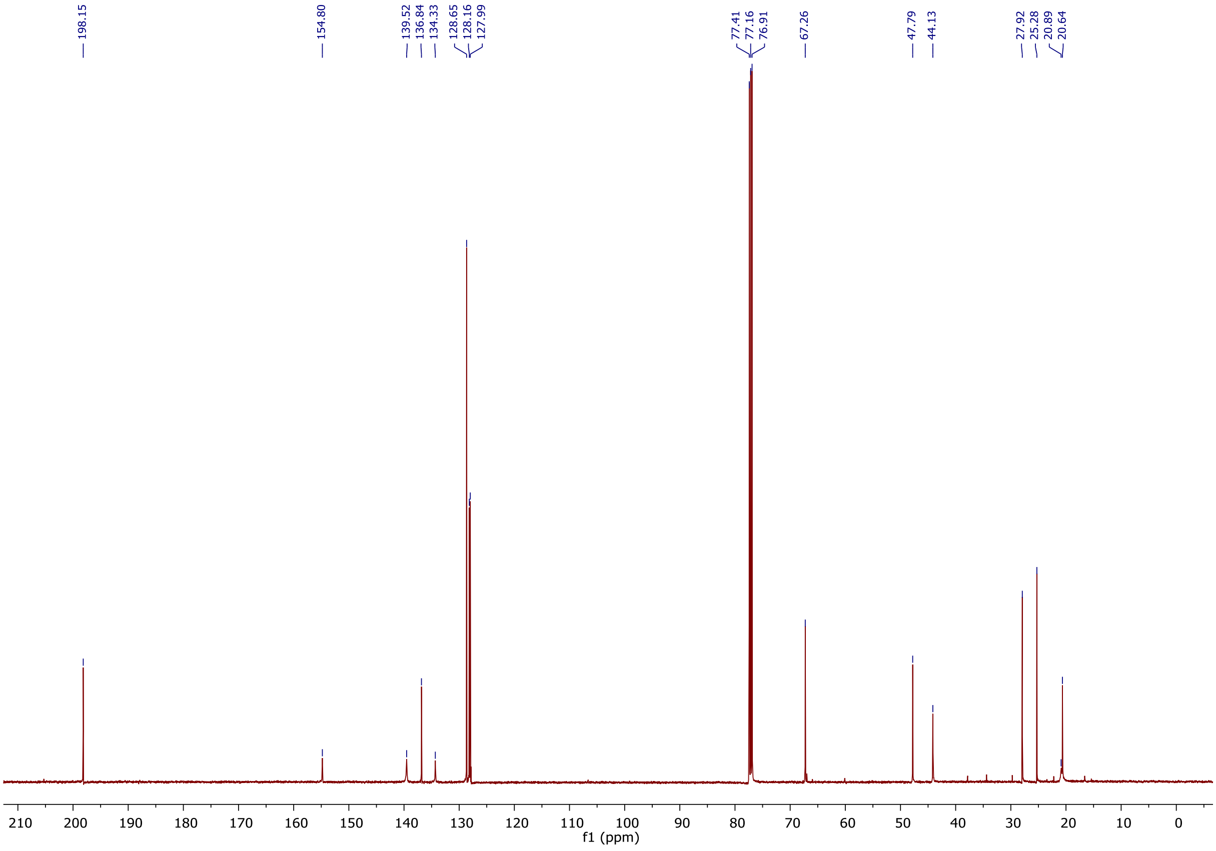

Benzyl (5-methyleneheptyl)((perfluorobenzoyl)oxy)carbamate

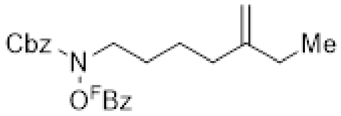

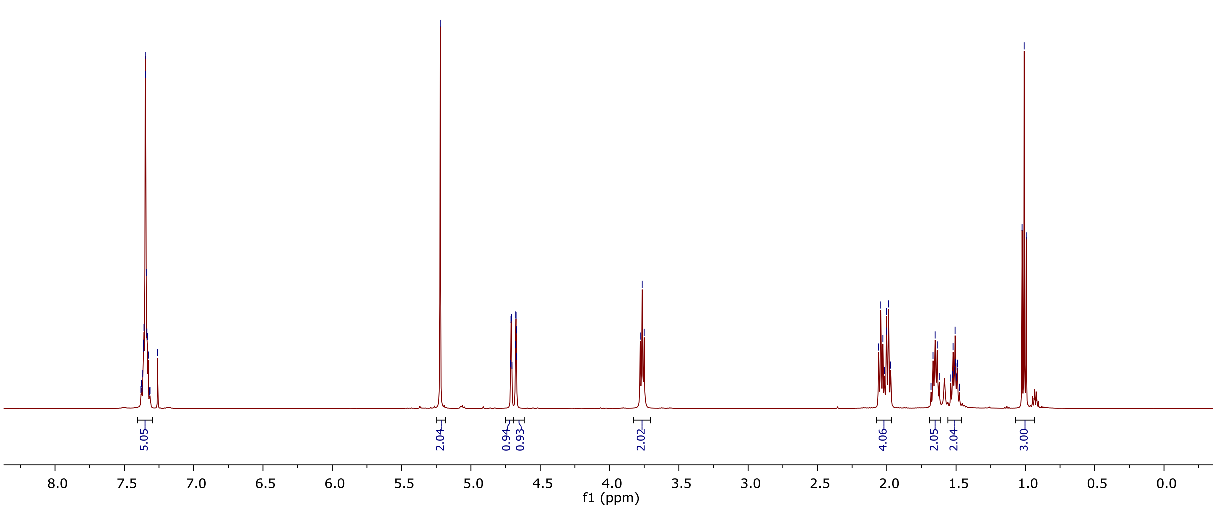

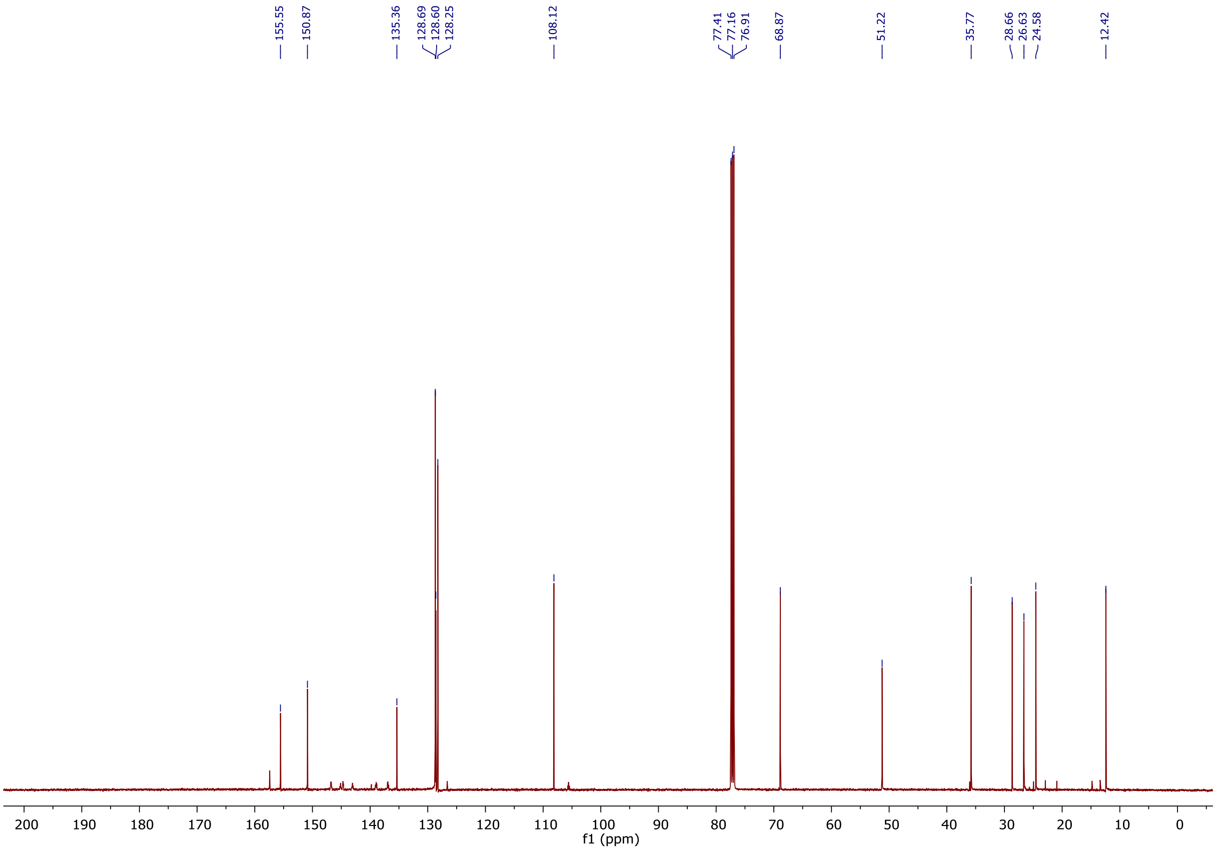

Notes and References

1. Hazelden, I. R.; Carmona, R. C.; Langer, T.; Pringle, P. G.; Bower, J. F. Pyrrolidines and Piperidines by Ligand-Enabled Aza-Heck Cyclizations and Cascades of *N*-(Pentafluorobenzoyloxy)carbamates. *Angew. Chem. Int. Ed.* **2018**, *57*, 5124 – 5128.
2. Ma, X. F.; Hazelden, I. R.; Langer, T.; Munday, R. H.; Bower, J. F. Enantioselective Aza-Heck Cyclizations of *N*-(Tosyloxy)carbamates: Synthesis of Pyrrolidines and Piperidines. *J. Am. Chem. Soc.* **2019**, *141*, 3356 – 3360.
3. Jones, B. T.; García-Cárceles, J.; Caiger, L.; Hazelden, I. R.; Lewis, R. J.; Langer, T.; Bower, J. F. Complex Polyheterocycles and the Stereochemical Reassignment of Pileamartine A via Aza-Heck Triggered Aryl C–H Functionalization Cascades. *J. Am. Chem. Soc.* **2021**, *143*, 15593 – 15598.
4. Shuttleworth, T. A.; Miles-Hobbs, A. M.; Pringle, P. G.; Sparkes, H. A. 2-Pyridyl Substituents Enhance the Activity of Palladium–phospha-adamantane Catalysts for the Methoxycarbonylation of Phenylacetylene, *Dalton Trans.* **2017**, *46*, 125 – 137.
5. Yokoshima, S.; Ueda, T.; Kobayashi, S.; Sato, A.; Kuboyama, T.; Tokuyama, H.; Fukuyama, T. Stereocontrolled Total Synthesis of (+)-Vinblastine. *J. Am. Chem. Soc.* **2002**, *124*, 2137 – 2139.
6. Banwell, M. G.; Edwards, A. J.; Jolliffe, K. A.; Smith, J. A.; Hamel, E.; Verdier-Pinard, P. Total Synthesis of (±)-Rhazinal, an Alkaloidal Spindle Toxin from *Kopsia teoi*. *Org. Biomol. Chem.* **2003**, *1*, 296 – 305.
7. Elliott, D. C.; Beutler, J. A.; Parker, K. A. The Importance of a 4-Alkyl Substituent for Activity in the Englerin Series. *ACS Med. Chem. Lett.* **2017**, *8*, 746 – 750.
8. Lee, J.; Parker, K. A.; A Formal Synthesis of (–)-Englerin A by RRCM and Transannular Etherification. *Org. Lett.* **2012**, *14*, 2682 – 2685.
9. Song, S.; Zhu, S.-F.; Yu, Y.-B.; Zhou, Q.-L. Carboxy-Directed Asymmetric Hydrogenation of 1,1-Diarylethenes and 1,1-Dialkylethenes. *Angew. Chem. Int. Ed.* **2013**, *52*, 1556 – 1559.
10. Erkkilä, A.; Pihko, P. M. Mild Organocatalytic *α*-Methylenation of Aldehydes. *J. Org. Chem.* **2006**, *71*, 2538 – 2541.
11. Yuan, W.; Berman, R. J.; Gelb, M. H. Synthesis and Evaluation of Phospholipid Analogues as Inhibitors of Cobra Venom Phospholipase A_2. *J. Am. Chem. Soc.* **1987**, *109*, 8071 – 8081.
12. Song, S.; Zhu, S.-F.; Yu, Y.-B.; Zhou, Q.-L. Carboxy-Directed Asymmetric Hydrogenation of 1,1-Diarylethenes and 1,1-Dialkylethenes. *Angew. Chem. Int. Ed.* **2013**, *52*, 1556 – 1559.
13. Guria, S.; Daniliuc, C. G.; Hennecke, U. Brønsted Acid-Catalyzed Enantioselective Iodocycloetherification Enabled by Triphenylphosphine Selenide Cocatalysis. *Adv. Synth. Catal.* **2021**, *363*, 3852 – 385.

14. Li, J.; Fu, N.; Zhang, L.; Zhou, P.; Luo, S.; Cheng, J.-P. Chiral Primary Amine Catalyzed Asymmetric Epoxidation of α-Substituted Acroleins. *Eur. J. Org. Chem.* **2010**, 6840 – 6849.
15. Mita, T.; Higuchi, Y.; Sato, Y. Highly Regioselective Palladium-Catalyzed Carboxylation of Allylic Alcohols with CO_2. *Chem. Eur. J.* **2015**, *21*, 16391 – 16394.
16. Alazet, S.; Vaillant, F. L.; Nicolai, S.; Courant, T.; Waser, J. Divergent Access to (1,1) and (1,2)-Azidolactones from Alkenes using Hypervalent Iodine Reagents. *Chem. Eur. J.* **2017**, *23*, 9501 – 9504.
17. Zhao, Y.; Jiang, X.; Yeung, Y.-Y. Catalytic, Enantioselective, and Highly Chemoselective Bromocyclization of Olefinic Dicarbonyl Compounds. *Angew. Chem. Int. Ed.* **2013**, *52*, 8597 – 8601.
18. Zhao, Y.; Jiang, X.; Yeung, Y.-Y. Catalytic, Enantioselective, and Highly Chemoselective Bromocyclization of Olefinic Dicarbonyl Compounds. *Angew. Chem. Int. Ed.* **2013**, *52*, 8597 – 8601.
19. Uetake, Y.; Niwa, T.; Nakad, M. Synthesis of Cycloalkanone-fused Cyclopropanes by Au(I)-catalyzed Oxidative Ene-yne Cyclizations. *Tetrahedron Lett.* **2014**, 55, 6847 – 6850.
20. Rösner, C.; Hennecke, U. Homohalocyclization: Electrophilic Bromine-Induced Cyclizations of Cyclopropanes. *Org. Lett.*, **2015**, 17, 3226 – 3229.
21. Gansäuer, A.; Worgull, D.; Knebel, K.; Huth, I.; Schnakenburg, G. 4-*exo* Cyclizations by Template Catalysis. *Angew. Chem. Int. Ed.* **2009**, *48*, 8882 – 8885.
22. Poplata, S.; Bauer, A.; Storch, G.; Bach, T. Intramolecular [2+2] Photocycloaddition of Cyclic Enones: Selectivity Control by Lewis Acids and Mechanistic Implications. *Chem. Eur. J.* **2019**, *25*, 8135 – 8148.
23. Poplata, S.; Bauer, A.; Storch, G.; Bach, T. Intramolecular [2+2] Photocycloaddition of Cyclic Enones: Selectivity Control by Lewis Acids and Mechanistic Implications. *Chem. Eur. J.* **2019**, *25*, 8135 – 8148.
24. Gampe, C. M.; Carreira, E. M. Cyclohexyne Cycloinsertion in the Divergent Synthesis of Guanacastepenes. *Chem. Eur. J.* **2012**, *18*, 15761 – 15771.
25. Miles, K. C.; Le, C. C.; Stambuli, J. P. Direct Carbocyclizations of Benzoic Acids: Catalyst-Controlled Synthesis of Cyclic Ketones and the Development of Tandem aHH (acyl Heck–Heck) Reactions. *Chem. Eur. J.* **2014**, *20*, 11336 – 11339.
26. Vong, K.; Yamamoto, T.; Chang, T.; Tanaka, K. Bioorthogonal Release of Anticancer Drugs *via* Gold-triggered 2-Alkynylbenzamide Cyclization. *Chem. Sci.* **2020**, *11*, 10933 – 10938.
27. Guo, C.; Huang, K.; Wang, B.; Xie, L.; Xu, X. Palladium-catalyzed Annulation Reactions of Methyl *o*-Halobenzoates with Azabicyclic Alkenes: a general protocol for the construction of benzo[*c*]phenanthridine derivatives. *RSC Adv.* **2013**, *3*, 17271 – 17280.
28. Kadiyala, R. R.; Tilly, D.; Nagaradja, E.; Roisnel, T.; Matulis, V. E.; Ivashkevich, O. A.; Halauko, Y. S.; Chevallier, F.; Gros, P. C.; Mongin, F. Computed CH Acidity of Biaryl

Compounds and Their Deprotonative Metalation by Using a Mixed Lithium/Zinc-TMP Base. *Chem. Eur. J.* **2013**, *19*, 7944 – 7960.

29. Janssen-Müller, D.; Schedler, M.; Fleige, M.; Daniliuc, C. G.; Glorius, F. Enantioselective Intramolecular Hydroacylation of Unactivated Alkenes: An NHC-Catalyzed Robust and Versatile Formation of Cyclic Chiral Ketones. *Angew. Chem. Int. Ed.* **2015**, *54*, 12492 – 12496.
30. Hosomi, A.; Hayashida, H.; Tominaga, Y. Synthesis and Consecutive Double Alkylation Reactions of (2-Siloxyallyl)silanes as the Synthetic Equivalent of Acetone *α*,*α*'-Dianion. *J. Org. Chem.* **1989**, *54*, 3254 – 3256.
31. Koch, K.; Hurley, B.; Yang, W.; Lyssikatos, J.; Blake, J. F.; Marlow, A. L.; Wallace, E. M. Preparation of Fused (is)Oxazoles and Analogs as Inhibitors of MEK Kinase. U.S. Patent US 2006/0030610 Al, February 9, **2006**.
32. Johnston, M. I.; Kwass, J. A.; Beal, R. B.; Snider, B. B. Stereochemical Sudies of Type-II Intramolecular Ene Reactions of *δ,ε*-Unsaturated Aldehydes. *J. Org. Chem.* **1987**, *52*, 5419 – 5424.
